# Insights into long non-coding RNA regulation of anthocyanin carrot root pigmentation

**DOI:** 10.1038/s41598-021-83514-4

**Published:** 2021-02-18

**Authors:** Constanza Chialva, Thomas Blein, Martin Crespi, Diego Lijavetzky

**Affiliations:** 1 Facultad de Ciencias Agrarias, Instituto de Biología Agrícola de Mendoza (IBAM), UNCuyo, CONICET, Almirante Brown 500, M5528AHB Chacras de Coria, Mendoza Argentina; 2 Institute of Plant Sciences Paris-Saclay (IPS2), CNRS, INRA, University Paris-Saclay and University of Paris, Batiment 630, Gif Sur Yvette, France

**Keywords:** Natural variation in plants, Plant genetics, Plant molecular biology

## Abstract

Carrot (*Daucus carota* L.) is one of the most cultivated vegetable in the world and of great importance in the human diet. Its storage organs can accumulate large quantities of anthocyanins, metabolites that confer the purple pigmentation to carrot tissues and whose biosynthesis is well characterized. Long non-coding RNAs (lncRNAs) play critical roles in regulating gene expression of various biological processes in plants. In this study, we used a high throughput stranded RNA-seq to identify and analyze the expression profiles of lncRNAs in phloem and xylem root samples using two genotypes with a strong difference in anthocyanin production. We discovered and annotated 8484 new genes, including 2095 new protein-coding and 6373 non-coding transcripts. Moreover, we identified 639 differentially expressed lncRNAs between the phenotypically contrasted genotypes, including certain only detected in a particular tissue. We then established correlations between lncRNAs and anthocyanin biosynthesis genes in order to identify a molecular framework for the differential expression of the pathway between genotypes. A specific natural antisense transcript linked to the *DcMYB7* key anthocyanin biosynthetic transcription factor suggested how the regulation of this pathway may have evolved between genotypes.

## Introduction

Anthocyanins are flavonoids, a class of phenolic compounds synthesized via the phenylpropanoid pathway, a late branch of the shikimic acid pathway^1^. They are secondary metabolites that confer purple, red, and blue pigmentation to several organs and tissues of many plant species^2^. These water-soluble pigments serve in various roles in the plant, including attracting pollinators to flowers and seed dispersers to fruits, protection against UV radiation, amelioration of different abiotic and biotic stresses, such as drought, wounding, cold temperatures, and pathogen attacks^3,4^, as well as participation in physiological processes such as leaf senescence^5,6^. As dietary components, anthocyanins possess various health-promoting effects, mainly due to their antioxidant and anti-inflammatory properties, including protection against cancer, strokes and other chronic human disorders^7^.

Carrot (*Daucus carota* subsp. *carota* L.; 2n = 2x = 18) is a globally important root crop with yellow and purple as the first documented colors for domesticated carrot in Central Asia approximately 1100 years ago^8^. Orange carrots were not reliably reported until the sixteenth century in Europe^9,10^, where its popularity was fortuitous for modern consumers because the orange pigmentation results from high quantities of α- and β-carotene, making carrots the richest source of provitamin A in the US diet^11^. Additionally, with its great nutrition and economic value, carrot has been well known as a nice model plant for genetic and molecular studies^11^. Carrot is one of the crops that can accumulate large quantities of anthocyanins in its storage roots (up to 17–18 mg/100 g fresh weight)^12^. Purple carrots accumulate almost exclusively derivatives of cyanidin glycosides with five cyanidin pigments reported in most studies^13,14^. The root content of these five anthocyanin pigments vary across carrot genetic backgrounds^12,15^. In addition, anthocyanin pigmentation also varies between root tissues, ranging from fully pigmented roots (i.e., purple color in the root phloem and xylem) to pigmentation only in the outer-most layer of the phloem^16,17^.

Regardless of the plant species, at least two classes of genes are involved in anthocyanin biosynthesis: structural genes encoding the enzymes that directly catalyze the production of anthocyanins, and regulatory genes that control the transcription of structural genes^18,19^. In most cases, the anthocyanin biosynthetic structural genes are regulated by transcription factors (TFs) belonging to the R2R3–MYB, basic helix–loop–helix (bHLH) and WD-repeat protein families, in the form of the ‘MBW’ complex^19,20^. Recent reports pointed out that gene regulation by TFs may play a key role controlling anthocyanin pigmentation in purple carrots^17,21,22^. Moreover, the broad variation observed among purple carrot root genotypes, regarding both anthocyanin concentration and pigment distribution in the phloem and xylem tissues, suggests independent genetic regulation in these two root tissues^23^. In this sense, Xu et al.^16^ found that the expression pattern of a R2R3–MYB TF, *DcMYB6*, is correlated with anthocyanin production in carrot roots and that the overexpression of this gene in *Arabidopsis thaliana* enhanced anthocyanin accumulation in vegetative and reproductive tissues in this heterologous system. Similarly, Kodama et al.^24^ found that a total of 10 *MYB*, *bHLH* and *WD40* genes were consistently up- or downregulated in a purple color-specific manner, including *DcMYB6*. Iorizzo et al.^25^ identified a cluster of MYB TFs, with *DcMYB7* as a candidate gene for root and petiole pigmentation, and *DcMYB11* as a candidate gene for petiole pigmentation. Bannoud et al.^23^ showed that *DcMYB7* and *DcMYB6* participate in the regulation of phloem pigmentation in purple-rooted samples. Finally, Xu et al.^26^, by means of loss- and gain-of-function mutation experiments, demonstrated that *DcMYB7* is the main determinant that controls purple pigmentation in carrot roots.

Non-coding RNAs with a length higher than 200 nucleotides are defined as long noncoding RNAs (lncRNAs). They were originally considered to be transcriptional byproducts, or transcriptional ‘noise’, and were often dismissed in transcriptome analyses due to their low expression and low sequence conservation compared with protein-coding mRNAs. However, specific lncRNAs were shown to be involved in chromatin modification, epigenetic regulation, genomic imprinting, transcriptional control as well as pre- and post-translational mRNA processing in diverse biological processes in plants^27–30^. Certain lncRNAs can be precursors of small interfering RNA (siRNA) or microRNA (miRNAs), triggering the repression of protein-coding genes at the transcription level (transcriptional gene silencing or TGS) or at post-transcriptional level (PTGS)^27,31^. Additionally, other lncRNAs can act as endogenous target mimics of miRNAs, to fine-tune the miRNA-dependent regulation of target genes^32,33^. It has been suggested that lncRNAs can regulate gene expression in both the *cis*- and *trans*-acting mode^35^. The *cis*-acting lncRNAs can be classified by their relative position to annotated genes^27,34,35^ and notably include long noncoding natural antisense (lncNATs) transcribed in opposite strand of a coding gene, overlapping with at least one of its exons^36,37^. Other so-called intronic lncRNAs are transcribed within introns of a protein-coding gene^38^ whereas long intergenic ncRNAs (lincRNAs) are transcripts located farther than 1 kb from protein-coding genes^27,34,35^. Among these *cis*-lncRNAs, NATs are of special interest as they have been shown to provide a mechanism for locally regulating the transcription or translation of the target gene on the other strand, providing novel mechanisms involved in the regulation of key biological processes^39^, plant development^40^ and environmentally dependent gene expression^36,37^.

As mentioned above, several differential expression analyses have been performed between purple and non-purple carrot roots allowing the identification of the main structural genes and TFs involved in anthocyanin biosynthesis in whole roots and/or phloem tissues^16,21,23–26^. However, the identification and functional prediction of lncRNA in carrot or putatively involved in carrot anthocyanin biosynthesis regulation has not yet been reported. In the present study, we combined a high throughput stranded RNA-Seq based approach with a dedicated bioinformatic pipeline, to annotate lncRNAs and analyze the expression profiles of lncNATs putatively associated to the carrot root anthocyanin biosynthesis regulation. In addition, we individually analyzed the gene expression patterns in phloem and xylem root of purple and orange *D. carota* genotypes. Our findings point to a role of antisense transcription in the anthocyanin biosynthesis regulation in the carrot root at a tissue-specific level.

## Results

### RNA-seq data mining, identification and annotation of anthocyanin-related lncRNAs

**Figure 1:**
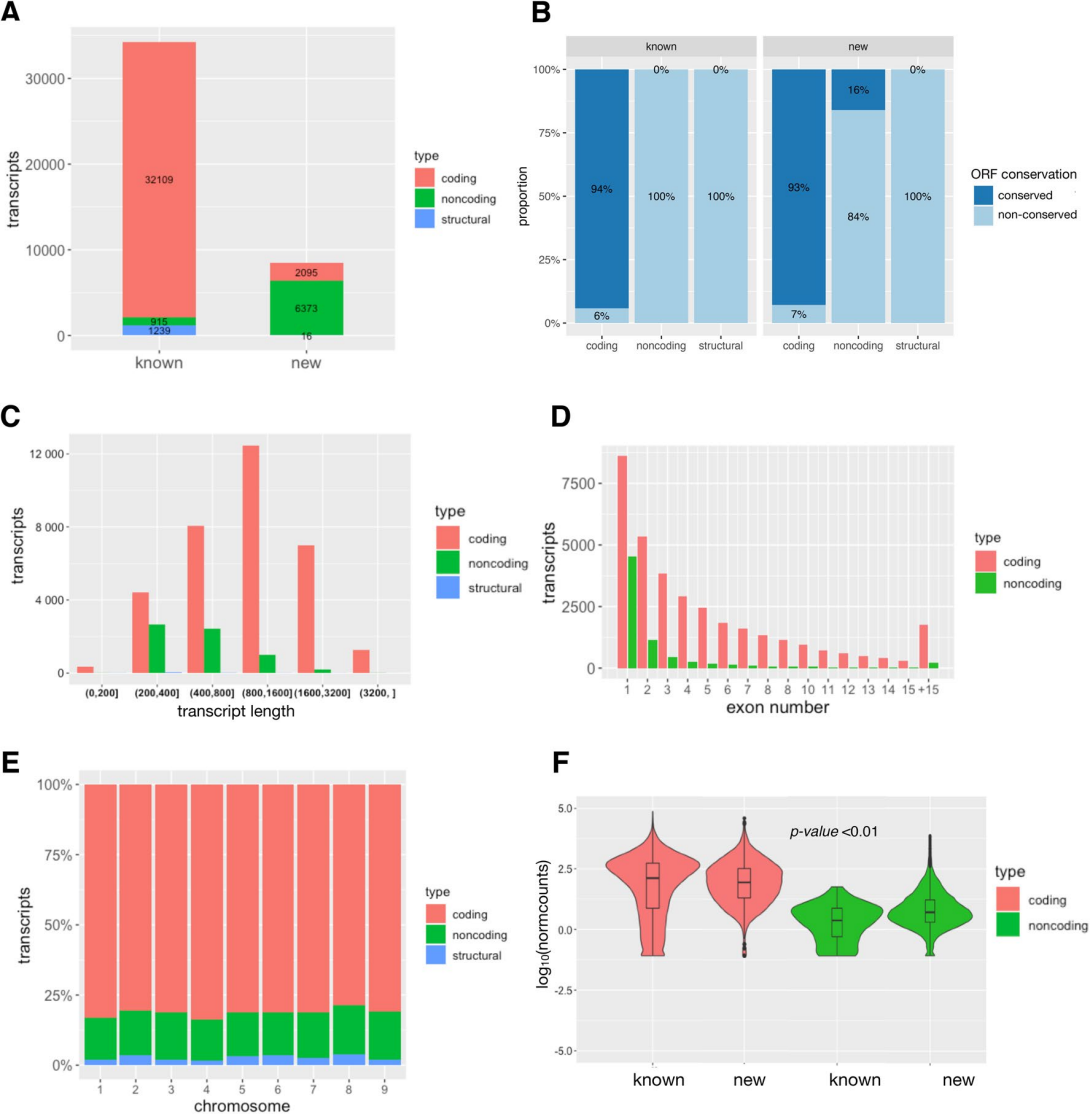
Characteristics of carrot transcripts. (**A**) Distribution of coding, noncoding and structural sequences between the known and newly annotated transcripts. (**B**) Conservation of the known and newly predicted protein-coding and non-coding transcripts. (**C**) Transcript length distributions for the total coding, noncoding and structural RNAs. (**D**) Number of exons per transcript for the total coding and noncoding RNAs. (**E**) Proportional distribution of the total coding, noncoding and structural RNAs along each chromosome. (**F**) Violin plot of the expression levels of carrot total coding and noncoding RNAs. The y-axis represents the average log2 of normalized count values. t-test *p* value < 0.01 is considered to be significantly different.

In order to thoroughly identify and annotate lncRNAs related to anthocyanin biosynthesis regulation in carrot roots, we performed a whole transcriptome RNA-seq analysis of specific tissues from the carrot genotypes ‘Nightbird’ (purple phloem and xylem) and ‘Musica’ (orange phloem and xylem) (Supplementary Figure S1). We generated an average of 51.4 million of reads per sample from the 12 carrot root samples (i.e., two phenotypes × two tissues × three biological replicates), ranging from 43.5 million to 60.3 million. The average GC content (%) was 44.8% and the average ratio of bases that have phred^41^ quality score of over 30 (Q30) was 94.1%. The average mapping rate to the carrot genome was 90.9% (Supplementary Table S1). We identified and annotated 8484 new transcripts, including 2095 new protein-coding and 6373 non-coding transcripts (1521 lncNATs, 4852 lincRNAs and 16 structural transcripts) (Supplementary Table S2 and Supplementary File S1). Those were added to the 34,263 known carrot transcripts^42^ to complete the final set of 42,747 transcripts used for this work. The set contains 34,204 coding transcripts and 7288 noncoding transcripts (1521 lncNATs, 5767 lincRNAs) and 1255 structural transcripts (Fig. 1A and Supplementary Table S3). As expected, the newly predicted protein-coding genes carry ORFs presenting strong homologies with already annotated ones. In contrary, the great majority of the newly predicted non-coding transcripts present no conservation of their predicted ORFs^43,44^ (Fig. 1B). Most non-coding transcripts presented less than 1000 bp long, being 400–800 bp the most frequent length class. Coding transcripts between 500 and 1000 bp long were the most frequent, while most structural transcripts presented less than 200 bp (Fig. 1C). Noncoding transcripts predominantly presented one exon and unexpectedly^45^, only one exon was also the most frequent class for coding transcripts (Fig. 1D). Additionally, we found no particular bias for the distribution of the noncoding transcripts along the nine carrot chromosomes (Fig. 1E). Finally, the expression level of the coding sequences (measured as normalized counts) was similar within the known, novel and total transcripts. This was also observed for the noncoding transcripts. As expected, the expression level of the coding genes was higher than that of the noncoding ones independently if they were already known or newly predicted (Fig. 1F). Normalized counts for each of the 12 sequenced libraries were included in Supplementary Table S4.

### Variation in coding and noncoding expression was mainly explained by the anthocyanin-pigmentation phenotype difference between orange and purple carrots

**Figure 2:**
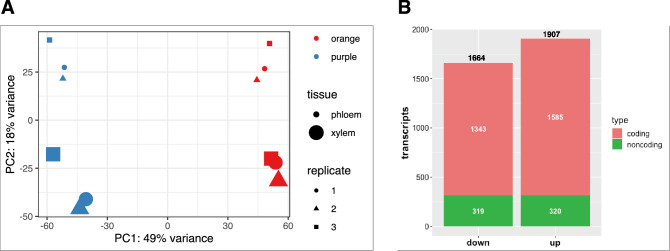
Expression of carrot coding and noncoding RNAs. (**A**) PCA analysis of the global gene expression of the 12 evaluated libraries (three replicates for each color-phenotype and tissue type combination). (**B**) Differentially expressed genes (up- and down-regulated) between purple and orange carrots (Bonferroni’s adjusted *p* value < 0.01) distributed by coding and noncoding transcripts.

We sampled phloem and xylem tissues from orange and purple carrot genotypes (Supplementary Figure S1). Considering the global gene variation of the 12 evaluated libraries (i.e., three for each phenotype/tissue combination), the color phenotype was clearly the main source of variation (PC1, 49%), while the tissue specificity factor was also important albeit less significant (PC2, 18%), (Fig. 2A).

We then assessed the variation in mRNA and ncRNA gene expression between purple and orange carrot roots in our RNA-seq analysis. A total of 3567 genes were differentially expressed (DEG) between purple and orange carrots (Bonferroni’s adjusted *p* value < 0.01), divided in 2928 mRNA and 639 lncRNAs (Fig. 2B) and representing 10% and 15% of the mRNA and lncRNA expressed genes, respectively. Within the 3567 DEGs, we found 1664 downregulated and 1907 upregulated transcripts. In turn, the downregulated transcripts were distributed into 1343 coding and 319 noncoding transcripts, while the upregulated were divided into 1585 and 320 coding and noncoding transcripts, respectively (Fig. 2B). All information concerning the differentially expressed analysis and gene annotation is detailed in Supplementary Table S5.

As expected, we identified several differentially expressed genes (DEG) between the two genotypes known to be involved in carrot root anthocyanin biosynthesis^21,23–26^. Most of the known genes of the pathway and their main regulators were differentially expressed between the two genotypes (Supplementary Table S5). Several genes were induced in purple tissues and they mainly comprised genes representing: (1) the early step in the flavonoid/anthocyanin pathway, like chalcone synthase (*DcCHS1*/DCAR_030786); chalcone isomerase (*DcCHI1*/DCAR_027694) and (*DcCHIL*/DCAR_019805); flavanone 3-hydroxylase (*DcF3H1*/DCAR_009483), and flavonoid 3′-hydroxylase (*DcF3′H1*/DCAR_014032); (2) cytochrome P450 (CYP450) proteins, putatively related to the flavonoid and isoflavonoid biosynthesis pathways^23,46^; (3) ATP-binding cassette (ABC) transporters, potentially related to anthocyanin transport^47,48^; and (4) genes from the late steps of the pathway, like dihydro-flavonol 4-reductase (*DcDFR1*/DCAR_021485), leucoanthocyanidin dioxygenase (*DcLDOX1*/DCAR_006772), and UDP-glycosyltransferase (*DcUFGT*/DCAR_009823) and the recently described *DcUCGXT1*/DCAR_021269 and *DcSAT1*/MSTRG.8365, which were confirmed to be responsible for anthocyanin glycosylation and acylation, respectively^26,49^. Finally, the most significant regulatory genes of the pathway, belonging to the MYB, bHLH and WD40 TF gene families^21,23–26^ were also differentially expressed between purple and orange genotypes (Supplementary Table S5). We further analyzed the tissue differential expression distribution of those 26 ‘MBW’ TFs and found that *DcMYB6* and *DcMYB7,* the two most studied TFs associated with anthocyanin biosynthesis regulation^23–26^, were differentially expressed between purple and orange carrots, both in phloem and xylem tissues (Supplementary Figure S2). Interestingly, three genes recently described to be regulated by *DcMYB7*^26^ (i.e. *DcbHLH3*, *DcUCGXT1* and *DcSAT1*) also displayed no tissue specificity. *DcbHLH3* was described as a co-regulator in anthocyanin biosynthesis, while *DcUCGXT1* and *DcSAT1* participate in anthocyanin glycosylation and acylation, respectively^26,49^. Additionally, seven TFs showed xylem preferential expression-specificity, while only one was preferentially expressed specifically in phloem. Finally, differential expression of 11 TFs was just detected when the 12 libraries were jointly analyzed, presumably because they have significant but low expression differences (Supplementary Figure S2).

### Putative regulation of anthocyanin-related genes by carrot antisense lncRNAs

In order to investigate the putative involvement of carrot lncRNAs in the regulation of the anthocyanin biosynthesis in different carrot root tissues, we predicted the potential targets of lncRNAs in *cis*-regulatory relationship, particularly those classified as natural antisense transcripts (lncNATs). The selection of such lncRNAs was based on three assumptions: (1) both, the lncRNA and the putative target were differentially expressed between purple and orange tissues (Supplementary Table S5); (2) the lncRNAs were antisense of the target genes; and (3) the Pearson and Spearman correlation coefficients between the expression levels of these genes were ≥ 0.70 or ≤  −0.70, and *p* < 0.01.

**Figure 3:**
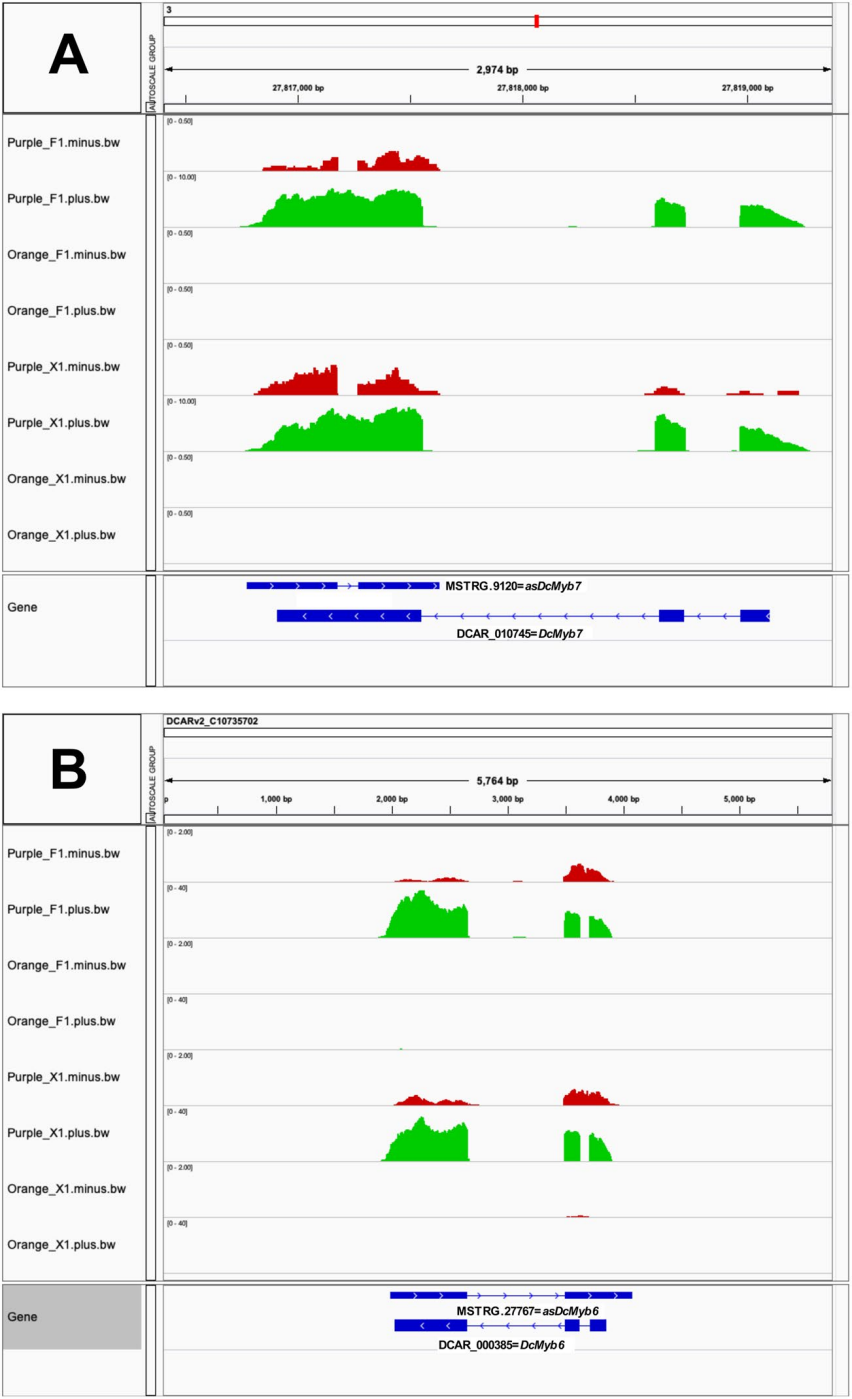
Strand specific expression of R2R3–MYB TFs and their lncNATs. Coverage data for the sense (green) and antisense (red) strands corresponding to *DcMYB7*/*asDcMYB7* (**A**) and *DcMYB6/as DcMYB6* (**B**), respectively. Tracks correspond to four carrot libraries: two phloem samples Purple_F1 and Orange F1; and two xylem samples Purple_X1 and Orange_X1. Data Range of each track was set to allow an even visualization of the mRNA and lncRNA transcripts by enlarging the last ones (20x).

According to these criteria, we found 19 differentially expressed lncNATs, since the lncRNAs were located in the antisense orientation (in the opposite strand) to a target mRNA, being most of them fully overlapping pairs (Supplementary Table S5 and S6). About 79% of those lncNATs were expressed in concordance with the sense strand transcript, while five out of the 19 presented discordant expression (i.e. when the lncNAT expression increase, the sense strand transcript was repressed) (Supplementary Table S5 and S6). Interestingly, we detected two lncNATs (MSTRG.27767/*asDcMyb6* and MSTRG.9120/*asDcMyb7*) in antisense relationship to the critical regulators *DcMYB6* and *DcMYB7*, respectively, with concordant expression correlation (Fig. 3). *DcMYB6* showed a log_2_ fold-change of 7.6 with an adjusted *p* value of 4.5 × 10^–30^, while *DcMYB7* presented a log_2_ fold-change of 11.7 with an adjusted *p* value of 3.8 × 10^–37^. Accordingly, the two detected antisense lncRNAs also presented significant differential expression, where *asDcMYB6* displayed a log_2_ fold-change of 6.5 with an adjusted *p* value of 2.1 × 10^–13^ and *asDcMYB7* presented a log_2_ fold-change of 6.1 with an adjusted *p* value of 1.3 × 10^–04^ (Supplementary Table S5). Finally, the Pearson and Spearman correlation coefficients between the expression levels of each sense/antisense pair were ≥ 0.79 and *p* value < 0.01 (Supplementary Table S6). On the other hand, as also detailed in Supplementary Table S5, two out of the four lncNATs showing discordant expression were found in the antisense relationship with disease resistance related genes (a predicted Catalase, and probable disease resistance protein At5g63020).

### The differential expression of *DcMYB6* and *DcMYB7* and their lncNATs was validated by RT-qPCR

**Figure 4:**
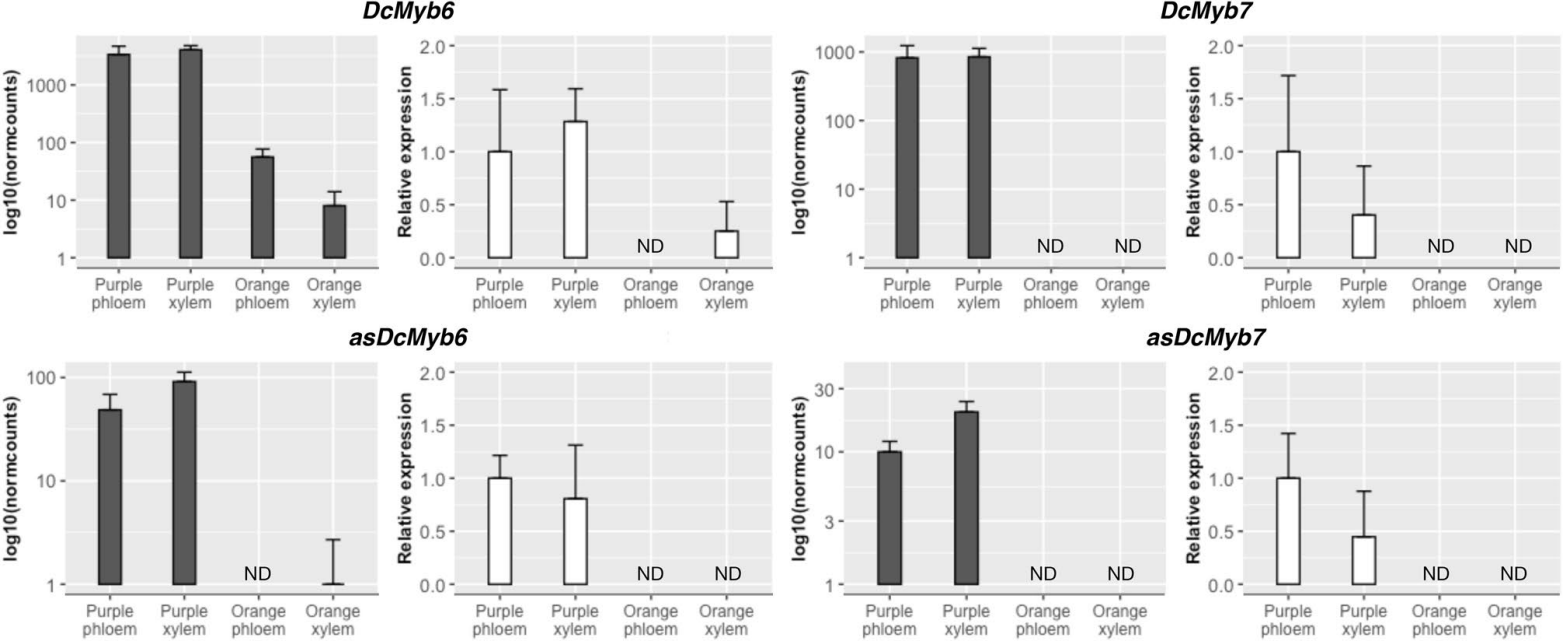
Comparison of expression results from RNA-Seq (log_10_ of normalized counts) and RT-qPCR (Relative expression) methods for *DcMyb6*, *DcMyb7* and their corresponding lncNATs. Data are means ± SD of three biological replicates. For RT-qPCR, carrot *actin-7* was used as reference gene and ‘Purple phloem’ as reference sample. *ND* not detected.

In order to validate the differential expression results obtained by RNA-seq, we performed a RT-qPCR analysis of *DcMYB6* and *DcMYB7* and their corresponding lncNATs (*asDcMYB6*and *asDcMYB7*). As shown in Fig. 4, the expression of the four genes was detected by RNA-seq and RT-qPCR in all purple samples, being mostly undetected in orange tissues. Moreover, both techniques allowed the detection of gene expression in orange tissues only for *DcMYB6*, displaying significantly lower values than in purple tissues. The comparative RT-qPCR expression of the four genes in purple phloem and xylem tissues is presented in Supplementary Figure S3.

## Discussion

The presence of color in flowers, fruits and other organs and tissues, plays several biological functions mostly driven by the adaptive behavior of plants in response to the environment^2,20,50,51^. But in turn, plant organ pigmentation has served as a natural genetic marker since the early works of Mendel^52,53^. Anthocyanins are flavonoid pigments that accumulate in plant cell vacuoles^54^ and are mainly responsible for most tissue and organ coloration^19,20,50^. Genetic analyses using model plant species like Arabidopsis, petunia and maize allowed the identification of most structural genes in the anthocyanin biosynthesis pathway as well as the main regulatory genes controlling pigment synthesis. In carrot, anthocyanin pigmentation is responsible for the purple phenotype^9,55^. Two main genes, *P*_1_* and P*_3_, have been identified in chromosome 3 and suggested to be responsible for the two independent mutations underlying the domestication of purple carrots^17^. Despite several carrot structural genes from the anthocyanin biosynthesis pathway have shown expression correlation with the purple phenotype^21,22^, none of them co-localize with *P*_1_* and P*_3_. A similar situation occurs in other plants like grapevine, where accumulation of anthocyanins correlated with the expression of several structural genes of the pathway but none of them co-localized with the ‘color locus’ in chromosome 2^56,57^. Finally, this discrepancy was solved by a study describing an insertion mutation in the promotor of a R2R3–MYB TF (i.e. *VviMybA1*)^58^ explaining the lack of color of white grapevine cultivars. In the same direction, several recent works^16,23–25,49^ focused on the role of carrot TFs putatively involved in the regulation of anthocyanin biosynthesis in purple genotypes, particularly those belonging to the ‘MBW’ complex (i.e., R2R3–MYB, basic helix–loop–helix -bHLH- and WD-repeat TFs). Two recent reports showed that three R2R3–MYB TFs are involved in the *P*_1_* and P*_3_ loci: *DcMYB113* has been suggested to correspond to *P*_1_^49^, while *DcMYB6* and *DcMYB7* were proposed as the two main candidate TFs underlying the carrot root anthocyanin pigmentation in the *P*_3_ locus^25^. However, knockdown and overexpression functional analyses demonstrated that *DcMYB7* (but not *DcMYB6*) is the *P*_3_ gene controlling purple pigmentation in carrot roots^26^. Likewise described for the grapevine *VviMybA1* gene^58^, non-purple carrot genotypes seems to arise by an insertion mutation in the promoter region of *DcMYB7*^26^, yet the authors imply the existence of an additional genetic factor suppressing the expression of *DcMYB7* in non-purple pigmented peridermal carrot root tissues.

In this work, we performed a thorough transcriptomic analysis by comparing two carrot hybrids with contrasted anthocyanin pigmentation phenotypes (i.e. purple vs. orange), both in phloem and xylem tissues. The study corroborates the involvement of the principal reported structural genes of the anthocyanin biosynthesis pathway^21,22^, but mostly, the key TF genes reported as the main regulators explain the carrot purple phenotype (i.e. *DcMYB6* and *DcMYB7*)^16,25,26^. Interestingly, the performed dissection between phloem and xylem purple samples, allowed us to show that there is no tissue-specific expression of such key genes, contrary to previously suggested for *DcMYB6* and *DcMYB7*^16,23,25^. One possible explanation for such discrepancy is that none of the reported works^16,23,25^ performed phloem and xylem transcriptomic analyses independently.

We showed here a first whole genome identification and annotation of lncRNAs in carrot by combining a high throughput stranded RNA-Seq based approach with a focused bioinformatic pipeline. Through this process, we identified 6373 novel lncRNAs, as compared to the 915 sequences annotated in the original carrot genome assembly^42^. Moreover, 10% of them (641 genes) can be defined as anthocyanin biosynthesis-related lncRNAs since we found them differentially expressed between purple and orange carrots. In order to assess the presumed function of such lncRNAs, we focused on those showing an antisense relationship with differentially expressed protein coding genes, known (or putatively) involved in carrot anthocyanin biosynthesis and depicted in the precedent paragraph. Additionally, the selected lncNATs had to present a statistically significant Pearson and Spearman correlation with their putative targets to further refine our functional predictions. This led us to identify 19 differentially expressed lncNATs between purple and orange carrots. Interestingly, we found two of these lncNATs (*asDcMYB6* and *asDcMYB7*) transcribed in opposite direction to *DcMYB6* and *DcMYB7*, respectively. Moreover, *asDcMYB6* and *asDcMYB7* exhibited concordant expression patterns with their corresponding sense transcripts opening the possibility that non-coding RNA antisense transcription is a new player in the regulation of carrot anthocyanin biosynthesis, through *DcMYB7* (and/or *DcMYB6*). This regulation maybe linked to the previously proposed unknown genetic factors^26^.

Antisense transcripts, particularly lncNATs, present in many genomes of diverse kingdoms, showed either positively or negatively correlated expression with their corresponding sense transcripts. This antisense lncRNAs regulate the expression of their sense transcripts in a negative or positive way, by means of different transcriptional or post-transcriptional mechanisms. In particular cases, upregulation of sense gene expression may be explained by the participation of a lncNAT in the inhibition of other factors at translational level, such as efficient translation initiation or elongation^59–61^.

In plants, both repression and activation roles have been assigned to some lncNATs in response to environmental conditions. While *COOLAIR* and *COLDAIR* negatively regulates *FLC* in vernalization responses^38,62^, and *SVALKA* controls *CBF1* expression to consequently regulate freezing tolerance^37^, the expression of another member of the *FLC* family (*MAF4*) is activated by the lncNAT MAS to fine-tune flowering time^36^. On the other hand, a rice lncNAT (TWISTED LEAF) have shown to maintains leaf blade flattening by regulating its associated sense R2R3-MYB gene^40^.

Anthocyanins are known to participate in abiotic stress responses and adaptation to environmental variations^3,4,63^, so the evolutionary role of the newly identified antisense transcripts *asDcMYB7* and *asDcMYB6* may be linked to the activation of anthocyanin biosynthesis through *DcMYB7* and *DcMYB6*. Hence, our work hints to new antisense regulations potentially involved in the variable expression of anthocyanin genes among carrot ecotypes.

## Methods

### Sample preparation and plant material

Total RNA was obtained independently from three biological replicates of phloem and xylem root samples of two *Daucus carota* L genotypes: ‘Nightbird’, a purple root hybrid (purple phloem and xylem) and ‘Musica’, a non-anthocyanin pigmentated root hybrid. Plants were germinated from seeds and roots were collected after 12 weeks. Frozen samples were grinded using liquid nitrogen and RNA was extracted using TRI Reagent® (Sigma-Aldrich) and purified using SV Total RNA Isolation System (Promega). RNA samples were quantified, and purity measured using a spectrophotometer (AmpliQuant AQ-07). RNA integrity and potential genomic DNA contaminations were checked through agarose gel electrophoresis.

### Library construction and RNA sequencing

Twelve samples (two genotypes × two tissues × three biological replicates) were sent to the Macrogen sequencing service (Seoul, Korea). Once in destination they were checked for total RNA integrity using a Bioanalyzer RNA Nano 6000 chip. All the samples qualified to proceed with the library construction having an RNA Integrity Number (RIN) ≥ 7. NGS transcriptomic libraries were constructed using a TruSeq Stranded mRNA LT Sample Prep Kit (Illumina). To verify the size of PCR enriched fragments, the template size distribution was checked on an Agilent Technologies 2100 Bioanalyzer using a DNA 1000 chip. The sequencing of libraries was performed as paired-end 101 bp reads on an Illumina HiSeq 2500 platform. The quality of the raw reads in the FastQ files was checked through FastQC^64^ and were then trimmed for sequencing adaptor and low quality sequences using Trimmomatic^65^ using ‘ILLUMINACLIP:TruSeq3-PE.fa:2:30:10 LEADING:21 TRAILING:21 MINLEN:30’ as parameters. For removing reads corresponding to remaining ribosomal RNA, trimmed reads were mapped to the rRNA reference using SortMeRNA^66^ using ‘-ref silva-bac-16s-id90.fasta --ref silva-bac-23s-id98.fasta --ref silva-euk-18 s-id95.fasta --ref silva-euk-28s-id98.fasta --paired_in --fastx --log -e 1e−07 -a 4 -v’ as parameters.

### New transcripts assembly and lncRNA identification

Clean filtered reads were aligned on the *D. carota* genome^42^ using the STAR aligner^67^ using ‘--alignIntronMin 20 --alignIntronMax 20,000 --outSAMtype BAM SortedByCoordinate --outReadsUnmapped Fastx’ as parameters. Subsequently, the aligned reads were assembled by means of StringTie^68^ and new transcripts were extracted and annotated using the GffCompare^69^ program (GffCompare classes “u”, “x”, to adjust). Only new transcripts whose length was greater than 200 nt were kept. The classification of the newly predicted transcript was performed as follow: (1) coding, if their predicted open reading frame (ORF) was greater than 120 aa or if they were predicted as coding by CPC2^70^ calculator; (2) structural, in case of homology with structural RNA (tRNA, rRNA, snRNA or snoRNA) after the analysis against Rfam^71^; and (3) non-coding, if they were predicted as non-coding by CPC2 calculator or in case of homology with known structured non-coding RNA in Rfam (miRNA precursors, lncRNA). For each transcript, the longest ORF on the forward strand with at least 70 amino acid was predicted using TransDecoder ("-S -m 50", v5.5.0)^72^. Each ORFs was then search against UniRef90 using DIAMOND v2.0.6^73^. Hits with an e-value lower than 1e−10 were considered as positive.

### Differential expression analysis

We performed a strand-specific read counting of coding and non-coding gene using on the carrot official annotation and the newly predicted genes of this study for each of the 12 aligned BAM files by means of the featureCount^74^ software included in the Rsubread package^75^. The resulted normalized counts (median of ratios)^76^ were used for differential expression analysis with DEseq2^77^. Differentially expressed genes were declared as having a Bonferroni’s adjusted *p* value < 0.01. Reads corresponding to the strand specific expression of mRNAs and their lncNATs were visualized with the Integrative Genomics Viewer (IGV) software^78^. Additional Venn diagrams were performed with Venny v2.1^79^.

### Real-time quantitative PCR (RT-qPCR) expression analysis

One microgram of total RNA from each of the 12 carrot samples described above was used for RT-qPCR. Protocols for cDNA synthesis and RT-qPCR were performed according to Lijavetzky et al. (2008) using a StepOne Plus Real-Time PCR System (Applied Biosystems, Life Technologies). Non-template controls were included for each primer pair, and each RT-qPCR reaction was completed in triplicate. Expression data were normalized against the carrot *actin-7* gene (LOC108202619). Relative quantification was performed by means of the ΔΔCt method using the ‘pcr’ R package^80^. Gene-specific primers were designed using the Primer Blast web tool^81^ and the sequences are described in Supplementary Table S7.

## Supplementary Information

Supplementary Information 1.

Supplementary Information 2.

Supplementary Table S2.

Supplementary Table S4.

Supplementary Table S5.

Supplementary Legends.

## Data Availability

Sequence files generated during this study have been deposited into the NCBI BioProject database accession PRJNA668894.
