# Supplementary material for: Insights into long non-coding RNA regulation of anthocyanin carrot root pigmentation
Source: Sci Rep. 2021 Feb 18;11:4093. doi: 10.1038/s41598-021-83514-4 (PMC7892999; doi:10.1038/s41598-021-83514-4)
Supplement: Supplementary file 1 — Supplementary Information 1. [file 41598_2021_83514_MOESM1_ESM.docx]

>MSTRG.3.1 gene=MSTRG.3

CAGTAATCCATTTTTCTTTCTTTACTCAGATCTACTTTCTCTCTCTCCTCGTTTCTCTTACCTCCGTCTT

TCTCTCAACCTGCCCCGATGTAAATCAATATAGTTTGCTACAACTGAATTTTGTTCCCATTGAGATAGAT

ACAAACATATAGATATAATTTAGTGCTGAAGTTGCGGTGATTTTGGTGTTTGATGATACTACGTTTTGGC

AGTGGTGATGTTGGTGATGGGGTGTCAACGGTGGTAGTGCAATGGTAGTGGCAGAGGCAGAGGCGAATTC

ACCCACCATCACGTCTCCACAAGTCCGCTGTTTACGCCTCCTGCTATATACTTTTGTGATTTTCGCTTTG

CAAATTAGTGATTTGTGCTTTATAAATTAGTGATTATCACTTTAGAAATTGGTAAAACAACGGGTACAGC

ACAGAAGCTAGTGACAGTGACACCAGGATGTGGCTGTGTTTTCAGACGACAGAGGTGAGGCTGGAGGTGA

CGGAGGTGAGGCGGG

>MSTRG.8.1 gene=MSTRG.8

GATACTACTACCTCCATTCTGCACCTTCCTGATGATTGTCTCTATTTCATTTTTCAACGCCTTGAATCTG

TTTTTGATCGCAAATCCTTTGGTCTCACTTGCCATCGCTGGCTATTCATAGAAAATACAAGCAGGCGGTC

TCTGCAATTCCCGTGTTCCTTCCGCCACCTACACCGCACTTCATTATCTCGAACCAGCTCAACTGTTGGT

TCTTTCCAGTTGTATACAATGCTTGATCGCTTCCAACACCTAGAGTTATTGTGCCTTTCAGGATGCGTAC

ATCTTCCTGACTCGGGGTTATCTCAATTGCAATATTATGGCTCGAAATTGCAGACGCTGTATCTAGATTG

TTGTTTTGGAATCACTGATGAT

>MSTRG.13.1 gene=MSTRG.13

GTAAATTTATTCTAAATAAAATGTCGATTCTGCCCTCCCCAGTCTACTCCGAGCCTGTTTCCTGACCCCG

GCCTCTGGGATGTAGCGATCATATCTTTCACATTGCATGCTCAGCTCGTTACTTCCTCCACCCACATTCA

TCTTTCACGGCTTATTTAGGCGATCATCATATCTGCGCAGTTTATGTATCAGGACTTAGGAGTGGAGCAG

GTGTCTGTGATCTAAATTCACGGTTCAGGGATGATAATTGGAGGATATGCCCTTCGGTGGTGAGCGCCTT

ATTTGCCTTCTGTGGAGGTTTGACAATGGAGTTTGTGTAATGTAGTGCGTGAAAACTCAACTGGCTTGTT

TCAGGAGATTAAGTGAGTATGATGTTAAGACTATCGGGTGTGTATCCGTGACATGTTGGACAATCTGGTA

GGCTCGAAAAGGAATTGGTATGGAGGGAAAAAAAATCAGTTGCTACAAAAGTGGTAAATAATGCAATTAT

TTTCCTTGATACATGGAATCTTGCTCGAACATATGATTCTAATCATCCGGTTGCCGCTACAAATCATGCT

GAAGTAGCAGAGCGCTGGTCTAAACTTGAAATTGGTAATATCAAGTTTAATTGTGATGCTGCTATTTTTA

GCTCTGAAAGCTGGCCCATGATCATAATTGTGTGTTGTTAGAGACTGCTGCTAATGTCCGTGTAGGGGGA

ATGGAACTGCAACTTGCTGAAGCGATTAGTTTAAAGGTGGTTGTGAATTGGATCAAAGATCTCAACATCA

ATACTTTTGCTGGTGTGCAGACTGATTGTGAGCACACATTCCTTGCTCTCTTCTTCAGGTCTGATTGTTG

CGATTGCAAGGCATTATATTACCAAATGTTTCTATGTCTTTTGTAAAATGGCTCGGAAACAGGACAATCA

ATTAGTCTTCTTGTTCACTACCTGGCCGTAGTCAATGAGGGTTCTGTCCCTATTGAGTTGGAGCCAAACT

TGATAGTTGATTTATCTTAATGAAATTGTTCCATTACCTTTCTAGAAAAATATATGTTTCCTTATATCGG

TATTAATTACACCTACGTTACAGATGTATGAACTACACTTGAC

>MSTRG.13.2 gene=MSTRG.13

CTTTCACATTGCATGCTCAGCTCGTTACTTCCTCCACCCACATTCATCTTTCACGGCTTATTTAGGCGAT

CATCATATCTGCGCAGTTTATGTATCAGGACTTAGGAGTGGAGCAGGTGTCTGTGATCTAAATTCACGGT

TCAGGGATGATAATTGGAGGATATGCCCTTCGGTGGTGAGCGCCTTATTTGCCTTCTGTGGAGGTATAGG

CTCAGCAAACCTGAAACATCAGAAATGCCATAGGGTGTTGCAAAGAACGCGTTCATTACATATACGGCCA

AAGGTTTGACAATGGAGTTTGTGTAATGTAGTGCGTGAAAACTCAACTGGCTTGTTTCAGGAGATTAAGT

GAGTATGATGTTAAGACTATCGGGTGTGTATCCGTGACATGTTGGACAATCTGGTAGGCTCGAAAAGGAA

TTGGTATGGAGGGAAAAAAAATCAGTTGCTACAAAAGTGGTAAATAATGCAATTATTTTCCTTGATACAT

GGAATCTTGCTCGAACATATGATTCTAATCATCCGGTTGCCGCTACAAATCATGCTGAAGTAGCAGAGCG

CTGGTCTAAACTTGAAATTGGTAATATCAAGTTTAATTGTGATGCTGCTATTTTTAGCTCTGAAAGCTGG

CCCATGATCATAATTGTGTGTTGTTAGAGACTGCTGCTAATGTCCGTGTAGGGGGAATGGAACTGCAACT

TGCTGAAGCGATTAGTTTAAAGGTGGTTGTGAATTGGATCAAAGATCTCAACATCAATACTTTTGCTGGT

GTGCAGACTGATTGTGAGCACACATTCCTTGCTCTCTTCTTCAGGTCTGATTGTTGCGATTGCAAGGCAT

TATATTACCAAATGTTTCTATGTCTTTTGTAAAATGGCTCGGAAACAGGACAATCAATTAGTCTTCTTGT

TCACTACCTGGCCGTAGTCAATGAGGGTTCTGTCCCTATTGAGTTGGAGCCAAACTTGATAGTTGATTTA

TCTTAATGAAATTGTTCCATTACCTTTCTAGAAAAATATATGTTTCCTTATATCGGTATTAATTA

>MSTRG.16.1 gene=MSTRG.16

GCTAAATTTTGTGTCACCTCTGGAAGCTCGTCGATAATTTTGTCCACTGGTATGCCGACGATCTGTTCCG

CCTCCTTATTGAAGAGGGTGAAAGTTGTTTGGGCTGTTGAATCTTCCACTCGGACGGTAAGTCTGAACCT

GTATTCAATTGATGAGTATCCTTAGTTTTTTTAATAGAATGCAAATGTGGTTGGCATTCATGCTAAAGGA

TGATTGCAGTTAAATTACCGTGGCTTGAAGTCTCCGGCCTCCTTAGAGCAGAATGTGCAGTAATACTTCC

CCTCAGTAGTCTGTACTTTGCGGGCGCATCGTGGGCAACAATTGTAGTACCATCCCTCGCCTTCCATCAC

CGCCACTATTGTCGCCTCGCATGTCCGTCGTAGAACCTACAATTTAAGTATTTTTTTATAGCAGTTGCTA

GGGGTTAACAGTGAGTTTGTCAGTCCTAAGAATATGCACATTAGGACCTCATGCTTACCAGGTAG

>MSTRG.22.1 gene=MSTRG.22

GGCAAAACAGGCCTCTCCCTCTTCTTCATTGTCTGCATATCCAGCCCGCTATTACTCTCAACCAAGAATT

TGAAATTAATTTCTGTAGTTTTGAAGCACAGATACAAAATTGAGAACAAGCAAGAAGACGAAGCAAAAAA

TACTCTATACTCACTTGAATTAGTTTTGGGTAAATTAACACTTGTTTATATATTTAATTATAGATAGAGA

GGCCTAGCTACAAATCGGTATCGGAAAATTCAAAATCCAGGTAATTCTAAGC

>MSTRG.26.1 gene=MSTRG.26

GTCATCGTGGATCAGTCCTCAATCATCGCATCATTTACCGGAACAGGGAATTAGATGCAATCGATGATTA

TGTTCGAATTGGTGAAAGCACCGCAATTGAAAGTTTGAGGAAATTTGTCAAAGCGATTGTGCAAATATTT

GGGGAACAATATTTAAGAAGGCCACATAGCGGTGATGTTAAAAGATTAATGGAGGTTGCTGAGCAGCGTG

GATTTCCAGGAATGCTAGGCAGTATCGATTGTATGCATTGGAGGTGGAAAAACTGTCCAACTGCGTGGCA

TGGGGCTTTTTCAGGTCGTTACCATGAGCCAACAATCATCCTTGAAGCTGTAGCTTCGTATGATCTTTGG

ATATGGCATGCTTATTTTGGGTTACCTGGATCGTTGAATGATATAAATGTATTGGACCGATCCACTTTAT

TTTCTGAATGGGCAGAGGGTCAAGCACCAGAAGTTAACTATACCATCAATGGGCACAATTACA

>MSTRG.27.1 gene=MSTRG.27

CAGGTGCACTTCAAGATGGATTGTGGTGAAAAAGTGCCATCTTTTTATAATTTGATCAGTGGAGACTGGA

GAGTATTCTGAATATGACGATTTTACACAAAATCAGGAAGAAAGGAATTTAGAAGCTACAATACAAAATT

CAAGTACTCGTTTTACAGAAAGTCAAGAAGAAAGAAATGCAGAATTCATATCAGTTGCATCTCAAGTATT

TTGTTCACAGAAAGTGAAGGAGAAAAGGCATCGAACGAAAAATTTTACAAGGCAAGAGGATGAATTATTG

ATTTCTGCTTGGCAAAATGTGTCACTGGATCCCATCACAGGGGCTGATAAAAAAATGGAACCTATTGGCA

GAGAGTGCAAAGCTACTTTATGAAGCATAAAAACTTTGAATCAGATCATACTTGGGGTTCATTGATGCAT

CGTTGGTCCGTGATTCAACTTGGGGTCAATAAATTTCAAGGATTTTATAATCAAATGGATGGAAGAAGCG

GGTACTCTGAAATTGATAAGATTCAAGGTGCAAAGCATATGTACAAAGAAGTATGCAAACAAAGCTTTTC

TCTCGAGCATTGTTGGAAGTACTTGGAACATATTGAGATATTTGCCTAAGTGGAATGTTGATTTTGCTAC

GAAAAGGACAAAAGTATCTCAAAAGAACAGTCCAACAACTTCTTCACCATCAACTCCAGAGTGTACAATC

GTGGAAAACTCGGACTTGGAAAGGCCTATCGGAAGGAAGGCTGCAAAAGAACTTCAGAAAAAAAAGAAAG

AAATTGGACAATGAGCACGATGATGATGCTGGAGCTGTAATTTTAGAAAAAAATGAGAGTTGATCAGATT

GAATCTAGAAAGCAAAGAAATGAGCACCTTAAAGAGATGTTGCAGTTGGCAAAAGAGAGAGATGAACGTG

AGAAGAGAAAAGAAGCCGCTGAACAAGTTGAAGCTGATGATAAAATTATGGCTATGGATTCAAGTTCTAT

GGGGGTAATTGAGGCTGAGTATTTTAACTTGAGAAAGCAGAAGATTATTGAAAGAAAGCGCAAACTTTTG

GTCAAGTAATTAATGTGTTATACAGCTAGAAATCTTTTGTCATTTAATTT

>MSTRG.29.1 gene=MSTRG.29

GACAGATCTTCAATCCACCAGTCTCCTTCACATTTGGCCTGCCGATTCATCTGAAGTCTTCCAGAACTAG

TATATTCCAAGCGAGAGAAGCTTGAAGCGATCTGTTCAACACACGCTTTGGATACTATAGCGTCAATGCA

TCTGCTAACAATTTTAAGCTCATCAGCAAAAGGAAGTAGGTTTTCACATTGTTGCAGTACTGCAATACAC

ATTTCAAGGTTCTTGCAGACAATAGTCTCCAGATATTCTTCAGCACGGGACCCAAGATTGTTACTTGAGA

ACTCCTCAGTCATTTCAAGGTAGTCGGAGACGCAAAGAAGTTGAGCAACATTAGCCGAT

>MSTRG.31.1 gene=MSTRG.31

CATGGCTCCCCCAGATAACTTCTCCATAAGAGTCGTGGTAGATGCTATCTCTCGTAGTGGAGATATCGTT

GAATCCGTGATCTATTTGGATTAGGAGTCTGAGGTAGTCATACTCAAGACTAATTCTAACAGGAGTAGGA

TTCTCCAAGATGGGCCTTCGGCCTATCTCCGACCTTATCGCCAAGAGGCCTAGTCCTGATCAAACTAGGA

CTCCTGGTTCCATAGAACTACGTACGGCTTGATCCCCTATAAATATAGGGGTACGTAGGCACATTGGGGG

ATCATGAGTTGAGAGCACGTGAGACCAACTCAAAACCCTAATCTCAGCCACCCCCTAAAACAAACAACCA

CCACTCTCCGGCGACCAAAACCAC

>MSTRG.38.1 gene=MSTRG.38

CTCCCTGAACTGCGCAACCAAAAACTCCCCGAAGCAGCAGCTCGGATCCCAACAGTAAACCGCTAACAAA

ATATAAACTGTGTTGCTAACCAGGAGGGGGCTTAGGAGAAGACGGAGGAGGATAGATCTAAGTTTGTGTG

GGCAATTTAGAGAATTTGATTGATGAGAATCATGCTATTGTTTCCAGTTTCTGTTGGTCCCGAGTATTGT

G

>MSTRG.42.1 gene=MSTRG.42

CAAAATTTAAGGTCAAAGAAAGTTTGTAAAAAGTATGAGCGATGATAAATGGGAGTGAAAAGTTTCTATA

ATGAGATTTTTGAAGAAATTGAGTAATGAAAATGATGAATATTTGCCTACAGAATAGTACAAATTTCATT

CCGACCTGAATTACTTTCATCCAACTAAATGGAGCATTGAGGATTCGTGTTTTAAAACTAAAAAATAAAT

CAAGATCATGAAAGTGAAATTAGGGCAAGCAGAAAAATAGGGGCAAATGACCTTTTCATCACATGTTGTA

TGGTCCTTGATTTTACTATTGCCCCTTGACCATCTCTAGAGTTAATATTTGCTATTCATAGTTCAAGCTA

AAATTTAATTCGCTATATATCATCATAAAATTCTATAATTTTTTTCCTGTGGTGTTAGGCATCAAATTAT

GATCACTAAAATCTCTCTTTCCTGTACCAGGGGCTAAACACATTGGTGGTTGTTCAGATACTGTTGAAAT

TTATCAGAAAGGGGAGCTTGAAAATTTGTTATCTGAAGCTGGCGCAAAGAAATAGAGCTAGTCTTTAACC

TCATCAAGGATCTCTATGCTGCTTCACTTTCATCAGAAAGAGTCACGAGTAATCAGTGTTGATTTGTCCA

CGAGTCCATAGGTTTCGGCATTAACACGTTTTGTTTGATATGTTTTTCTTAAGAAGTGTGCAAGTCCAGG

TAAATTTGTAGTGTTGAAAGAGTGAACTTGGTTTTATCCACATATTGCGATGGCATTGTTTTCCCTCAAG

AGGACTAAAATCTTTTTTGATCAAACTCGAGATGAGTTCCTTTTCTTATGGAACTTAGCTGATCTTGATA

AAGATTATATTAAGTACTCCCTCAGTCTCAATGAGTTGTATACATTTGGACGGAGGTGATATGTTATATG

TATACATTTGAGACGGAGGGTTCTGCACGAATTTTAAGGCTCTTATAATAGCATAAATTAGGAGTTGTAC

GGATGCTGTTGGGCCAGCCACATGTTCCCATTTCTGACTACCCAACTGGGATTCAAGTTCGAAACATTTC

CTAGATTTAAAGGCAAAAATGGATATTCTTAT

>MSTRG.43.1 gene=MSTRG.43

GTAGTATTGTGGGGTTTATGATTTTTTGTGGTGTGCAGAGCATTATGGCTCCTACAAGGAAGACAAGCTC

CTCTTTGAATACAACTAAAGTCATTATGGCTCCTACAAGGAAGACAAGATCCTCTTCGAATACAACTAAA

GTCAACCTTATGAAGGCATTAAAAAGAAACCATTCAGCAGGGGAGACAAGTGGGACGTCTAAGAAGCTTA

AGGCATCAACAAAAAACCATGCTGCAGGGGAGACAAGTGGGCTAGCTAAAAAGGCATTAAAAGGAAGCAC

TGTGCAGCCTCCCTCTCAGAGTTCTTCACCTAAATCTGAAAAGAAGAAGGCCATTTCTACAAATCTACAA

CAGCAACTGAGTAAGAAGCGGAAACAAAGGGAAGAAGGGCCTATAGATAACGAACAAGGAGTTAAGCATT

TGAAACGAGGACCTGTTACGATGGTCCGGATTCTTAGGCGTAAGATGTTGGGAGTCAAGTTAGCGGTTTC

TTTCAATGCAAAGGGTGAGCCATATGGTAAAGTTGCCACAGAGATG

>MSTRG.44.1 gene=MSTRG.44

GTACAAGACAGAAAAACTTGTAACCATAGCTGAAGAAATTGAGAAGATGAAGAAGAAAGTTGAAGAGGGG

GAAGTGACAGAGGAAGGCACTAATGATGTCTTGACCAAGGTTCTTGGTAACTCTGAGCACCGAGGACGGG

TGCGTGGTCAGGGGAGTTATGTTAAGCAATCCAACTATTTTAATCTGCCAAGACAGAAGAGAAAAAACCG

ATCAATTGAAGAAAAGATCCAAGAAGGTATTCAAAAGTTTATGGCTGATGAGACTAACAGGATCATCAAA

GAAAGAGATGAATTTTGGGCTGCAGAGATGGCAAAGCTGAAAGAAGCTTTAAGTATAAAGATTGATGGGA

GTCCAAATATTGGATCACAACAAGGAAGTTGTTCAAAAGGAGGACTTGAAAATGTGCTGGAATTGACAGC

TGTTAAAAAGAAATTGGATCTCAATGAATCTCCTAGAGGGGAAGATGCTGAGAATAATTTTCAGGAAGTT

GATGATGAAGAGAAGAACAAGGATAAAGAAAAAGATGTTGCTGAATTAAAGAGCAGTGGTGAAGAGAAAG

GAGTTGAGTATCTTGTAGTTGAGGACAAGGATGTGGAAGTGGCTGAGGAAAATGAAGGTATTCTATGGGA

GTTAGCAATTGGAACCCCAACCAACATTGTTGCTCATGCAACAGTTGACTTTGTCACTGCTGTTCTTCAT

GGGAAGCCACTTGGAGGAGATAACGTACGAGTATCAATTACTCGTGTTATCCAGGGAGCTGCTGAGATCC

CATTCCCAATTGACGATGAAATCATCACAGTAGATCAAGCTGTTGGAACTTTCATTGCATGGCCGAGAAA

CATGTTACGGGAAGTGAAGGCAAACAGTGATCGAGTGAAGTCTCGAGCAGTGAAGGGAGGCAAGAAGAAT

GGCCCAAGAAAAATGAAGAAGGTGAATGACTTGGTCTCGGAACCAGAGCCAGTGGTTAATATGGGCCCTG

ACTTCCCTCCTACTTTGAAAAAATTGTGGCTTTGGGCAAGCGATGCTTTGAAGGATGGTCGTTCACAGGC

GTTCAAGTTATCCGAAGAAGCTTTTGGATCATCGGATAAAAAGTGTTTGTTTAAATCCGACATAAGTGCT

GTGTGCTTTGGAGGTGAAATATCAGGAACTGTCATTTGCATGTTTATCAATATACTCCAGGAAAATTTAA

GAAAGCACAAAATGACAGACATGATATCTTTTGTTGATCCAGCCAAGATTGGGGCTCTTGGTTGTGGTAC

TCCAGCAGCGAGGTCACGCGCCCTTGCTCTCAGATTCAAAAGTGCTAAGCCATCCCAAGTCTTTCTTTTG

CCATATCACCACACGAACCACTGGGCACTTACTGTTGTAAATCCCGATGCACAAATGGTTTATCACTTGG

ACCCGTTAAAGCGGAGAATTGCAAATGAAGAATGGATTGAAGTTGTTAACAATGGCATAAAAATTTACAA

GGAGGATGTGAAGAGGTTTCTGAAGAAGAAAATAAATTGGGAGAATTTAGCGGGTGTTCCTGCCCAGACT

GGGACCACTGATTGTGGTCTTTTTGTGATGCTTTATATGAGAGAAATATGTATAGACAAGGAACTCAAGT

TTGCGTCCAAGTGGGCGCGGAGGAGCAATCTTGTCTTTGATAGCGATGATCTCAATGAGATCAGGAGTGC

CTGGGCAAAGTACTTCATGAGGCAGCATGCTAGTTAGGCTGCATAGAGGATTGATTATTTTCGGATGCAC

ATTTATATATATTGGGACTTATTACATGGTTTATTTTGCCTAAATTTAGACTTAAGTTATATCCATGTAT

GTCTTTTTTGGATATGTGCATCCGAAAATACAATACATTGTCGCGTCAAGATTGGTAGAATCTGTTGGTG

GGTTTTTGGATTGTTAGGTGCGTTTGCCTTGGTTAGTATTGTTGGATGATATGTGAATATTAGTTGGTGG

ATATTAGTTGGTTTTTGAATTGTTTTGGCTTTTATGTTTGGTTGGAAACATAGTTGGTAAATTGTGAAGA

TTGTTGGTATGTGGTGGC

>MSTRG.46.1 gene=MSTRG.46

GTCATACTACAAGAATCACACAATAAACACCACCAAATCAGAGTCCGTCAAAGTCGGTGCACAGCCAAGA

TAACTGCCTCTACTAGCCGTTTAAATCCGGTGAATCTCCGGCATTATATCTCACCGGACTGTCATTTTCC

GGCCAACCCACTCATCACAAGGTGTTAAAATGAATGGAATTCAGAATCACAAAACTCACAATATCGAGAT

ACCTTATCTAGGATGTTAGGGACGTGTAGTCAACGTTTTTGAACCGAGTGCAGGCATGCCAGGAAATAGA

CTGTTGATGGATAAGCCTCATCATAATGG

>MSTRG.49.1 gene=MSTRG.49

TAAGCTTTTCTGTTAAACAAAACAAGAAATGGACATTACATATAGACATGCTACAGTATTACACTATTAC

TGAAGGCAGCTGTAACCCTGATCACAGACAAATAATCAGCCAACACGAAATTAGTAAAGTCCTGGGCTCG

AATAAACCATCCTCTTCTTCCCAATTCAGGTATTTATGTCTATGCTTCTATGCTCGTGTTCATGGTACTG

GAGTTGGATGCCCTCCTTGGAATGTTTTTGTGGCACTCCTACCACTTTAAAGGACTATGATCAGTTCTAT

TTTATGGCATGGAGTGGTGGAACGACGAGTCTAGCTTGATCTGCTTATAGAGGTCAGATCTTCACGGTTG

AGCTTGAACTAATACACATACAGGGTTAGATTATCTGTCAGAAAATTCAGTTGCTTAATTAGTTTCCTCG

GTATCTTTAAGCTTTTTACTATTTCTCTACTCTTCACTGAATTTTGAGTTTTTTTCCGTCCATTTATGTC

ACTGTG

>MSTRG.59.1 gene=MSTRG.59

CGAAGGGTGTAAGGGAGAGATATATGTCTCCTCAACTCTTTACAAACTGCAAGCTGTTTGCATTAGCATA

TCGCTCCATAAATCTCATGAACCTGTCCCAATCTCGTGGAGTACGCATGACATACTTGGCTTCAATCCCT

GCAGGCTTTCCGTTTACAAACCTGGCGTTTACATCAACTGATTGAAGAGTCCCTTCCTCATCAATCATGT

AGAAGCCAGTGATCTCGCCAACCTCACCAGATGAGTCAAAAACTGATGGCTCGTCAAACCTAAATATAGC

CATACCATTTGTTCCATCCCTAGATTTAGTTAGCTTCACATCTGGAACAGTCTGCTCATCAGTCCCTTGG

ATAAATTGAATCGTTGGTTTAACCATCATTACACATAATCTTGACATCTGAATTTTTCTTCGTTGAGGGG

CATATTGTGAAGAGGACAGGTGCAATGTTTGTCCGCTGAATGAGGAGCCTGCACTACGGATG

>MSTRG.63.5 gene=MSTRG.63

TTCCCTTGACCCTAGACTTTTAAGCAGCTCCTCACGCTGTATCAATTACAATCTCAAGTCTCCGTCCATG

AATCCAATCTCGCTCAATTATATTTGATGGTTGGGACTGCACAGGCTGACAAAACGAGGCAAAGTGGTTC

CAATCCAAACATGCAATCTATGCTCAATCATAATCTGTTGATCTTCCCATTTTATACCATGCCAAACTGT

ATGGAGACGATGACAAAAGCTAAAAGCTAACGACTGTTTGCCAGTTTGCCAGCAAGTACTCCTGCTCTTT

CACAAACTAAGGTTAGTCTTGTGTTGACCGTGATGTTC

>MSTRG.65.7 gene=MSTRG.65

GTGATGATTATGATTATTGGAGTAATCTTCCCGCTGATATTCTTGAGCTTATCGTGGGGAAGTTTAGTTA

TCTCCGTGACTATTACAGATTCATTGCTGTCTGCAGATCATGGAGGGATTCACTTAGTCATTTCAAGAAG

CCCTGTCTTCCCCTCACCGAATATCCGTTCCTTCTTCTTGCGGAAGATGTTGCTCCAGGAGCTATGCTTG

AATATGACCCTAGTCAAGATTATAGTGAAGTAGAAGATAATAACGATGAGGAAGATGATAGTGGTGATGA

TGGAGGCGACAATGATGTTAGTGGTGGTGATGAAGAAGAAGAAGATAGCAGTGATGATGATGATGATGGC

AGTGGTGATGGTGATGATAATCACGAAGAAGACAATAATGGTGATGATGAAGACAATGACAAAGTAGATG

ATACCGAGGAAGATGAATATGATCATAATAAAATTTCAGTCGGTAGTCGTCGTGGTTTCTACAGTCTTTT

GACGGGAAAAACGTACTATATTGATTTGCCGGAAGCTGCTGGAAGACTAATAGTGGGGACCAATAAAGGG

TGGCTAGTAACTCTAGG

>MSTRG.73.2 gene=MSTRG.73

CTCAACTAAACCCGTACCAACACTCACTCTAATATTCCTATAAAAACACCCACACACACACTTGCTGTTT

GTTCTTTTCTTGATTCAAAAATAAAACGCAGAAATTAGAAAGCAATGGAGCTTGATCTGGGCGCAATGGC

GTCTATGATCGGCGTATCAGTCCCTGTTCTGAGGTTTCTGTTGTGTTTCATAGCTACTGTGCCGGTGAGT

TTTGTGTGGCGGTTGATTCCCGGCGGGCCGGCGGTGAAGAACTTGTATGCTGCGGTGACTGGGGTGGTTT

TGTCGTATTTGTCGTTTGGGGCGTCGTCGAATCTTCATTTTTTGGTGCCTATGCTGTGTGGGTATGGGGC

GATGGTGGTGTATCGGGAGAGATGTGGGCTTGTGACTTTTGTTTTGGCTATGGGATATCTCATTGGCTGC

CATGTATACTACATGAGTGGGGATGCATGGAAGGAAGGGGGAATTGATGCCACCGGTGCCTTGATGGTTC

TCACACTGAAAGTCATTTCTTGTGCAATTAACTACAATGATGGATTACTAAAGGAGGAAGATTTACGTGA

ATCACAAAAGAAAAATCGTTTACTTGAGTTGCCAACTATAACTGAGTATTTTGGATACTGCCTCTGTTGT

GGTAGTCACTTTGCTGGCCCAGTTTTTGAAATTAAGGATTATCTTAATTGGACAGAAAAGAAGGGGATTT

GGACAGCTTCAGATAAGGGGAAATCCCCATCCCCATATTGGCCAGCTCTCAGAGCAATCCTCCAAGCTGT

TATATGCATGGGCTTGTACCTGTATCTTTCGCCCCACTTCCCACTGACCCGTTTTACGGAACCTATATAT

CAAGAATGGGGTTTTTGGAAACGAATAAGTTACCAGTACATGGCTGGTTTTACGGCACGTTGGAAATATT

ACTTTATATGGTCAATCTCAGAATCTTCTATTATTATTTCTGGTCTGGGTTTTAGTGGCTGGACAAATTC

TTCACCACCCAAAGCACGATGGGACCGTGCAAAAAATGTAGATATCTTGGGTGTTGAGTTGGCAACGAGT

TCAGTTCAGCTGCCACTTGTATGGAACATACAAGTTAGTACCTGGCTTCGTCATTATGTTTATGACAGAC

TCGTTCAGAAGGGAAAGAAACCTGGTTTCTTCCAGTTGCTGGCAACACAGACTGTTACTTACCGCATACA

AGAGCGTATACTTCATAGCAACCATTGTTCCCGTAACATTGATCCTTCTTGGAAAGATTATTAAACCGGC

AAGGCCCGCCAGAACAAAAGCTCGCAAAGAAGAGTGAGATGGAATTGCAATTTCGGATTCTGAAATCCCT

GGATTGAACTTTACAAATCATTAGTATGCTTCTTCCAGCCATATTCCCTTGTCTTGCTATTCCGTAAAGT

GTTTTCTTTTTTATAGTTGAAATTGCTTTGCCTGTCCATGTTTGGACCACTGGCCTCACTTCAGAAATCT

AGGCCAACAGGCAATCGATTAGAAAGTTGTATTCTTACTTAGATCAGAACAAGTGATTGCAAGAATTATA

TTTCGGTTGGAATTTTTCCTCTTATCCGTGACAGTCCAATTAAAATGTGTGAAAATATAACCCTTATCAC

TTTGTGGAGGGAATTTTTTAGTTCTTCGTACCAGTAAATTACAATCCCTGTTGAAA

>MSTRG.73.1 gene=MSTRG.73

CAAAAACACAACAAGCAAACACAACTCAACTAAACCCGTACCAACACTCACTCTAATATTCCTATAAAAA

CACCCACACACACACTTGCTGTTTGTTCTTTTCTTGATTCAAAAATAAAACGCAGAAATTAGAAAGCAAT

GGAGCTTGATCTGGGCGCAATGGCGTCTATGATCGGCGTATCAGTCCCTGTTCTGAGGTTTCTGTTGTGT

TTCATAGCTACTGTGCCGGTGAGTTTTGTGTGGCGGTTGATTCCCGGCGGGCCGGCGGTGAAGAACTTGT

ATGCTGCGGTGACTGGGGTGGTTTTGTCGTATTTGTCGTTTGGGGCGTCGTCGAATCTTCATTTTTTGGT

GCCTATGCTGTGTGGGTATGGGGCGATGGTGGTGTATCGGGAGAGATGTGGGCTTGTGACTTTTGTTTTG

GCTATGGGATATCTCATTGGCTGCCATGTATACTACATGAGTGGGGATGCATGGAAGGAAGGGGGAATTG

ATGCCACCGGTGCCTTGATGGTTCTCACACTGAAAGTCATTTCTTGTGCAATTAACTACAATGATGGATT

ACTAAAGGAGGAAGATTTACGTGAATCACAAAAGAAAAATCGTTTACTTGAGTTGCCAACTATAACTGAG

TATTTTGGATACTGCCTCTGTTGTGGTAGTCACTTTGCTGGCCCAGTTTTTGAAATTAAGGATTATCTTA

ATTGGACAGAAAAGAAGGGGATTTGGACAGCTTCAGATAAGGGGAAATCCCCATCCCCATATTGGCCAGC

TCTCAGAGCAATCCTCCAAGCTGTTATATGCATGGGCTTGTACCTGTATCTTTCGCCCCACTTCCCACTG

ACCCGTTTTACGGAACCTATATATCAAGAATGGGGTTTTTGGAAACGAATAAGTTACCAGTACATGGCTG

GTTTTACGGCACGTTGGAAATATTACTTTATATGGTCAATCTCAGAATCTTCTATTATTATTTCTGGTCT

GGGTTTTAGTGGCTGGACAAATTCTTCACCACCCAAAGCACGATGGGACCGTGCAAAAAATGTAGATATC

TTGGGTGTTGAGTTGGCAACGAGTTCAGTTCAGCTGCCACTTGTATGGAACATACAAGTTAGTACCTGGC

TTCGTCATTATGTTTATGACAGACTCGTTCAGAAGGGAAAGAAACCTGGTTTCTTCCAGTTGCTGGCAAC

ACAGACTGTTAGTGCTGTATGGCATGGTCTTTATCCTGGGTACATGATATTTTTTGTTCAGACAGCTTTG

ATGATTGCTGGATCCAGAGTCCTATACAAATGGCAGCAATCTCTTCCTCCAAAAATGGCCATAGTGAAGA

ATCTATTGGTATTCATTAACTTTTTGTATACACTTATGGTTCTTAACTGCTCTGCCGCTGGTTTCATGGT

ATTAAGCTTCCATGAAACTCTTACCGCATACAAGAGCGTATACTTCATAGCAACCATTGTTCCCGTAACA

TTGATCCTTCTTGGAAAGATTATTAAACCGGCAAGGCCCGCCAGAACAAAAGCTCGCAAAGAAGAGTGAG

ATGGAATTGCAATTTCGGATTCTGAAATCCCTGGATTGAACTTTACAAATCATTAGTATGCTTCTTCCAG

CCATATTCCCTTGTCTTGCTATTCCGTAAAGTGTTTTCTTTTTTATAGTTGAAATTGCTTTGCCTGTCCA

TGTTTGGACCACTGGCCTCACTTCAGAAATCTAGGCCAACAGGCAATCGATTAGAAAGTTGTATTCTTAC

TTAGATCAGAACAAGTGATTGCAAGAATTATATTTCGGTTGGAATTTTTCCTCTTATCCGTGACAGTCCA

ATTAAAATGTGTGAAAATATAACCCTTATCACTTTGTGGAGGGAATTTTTTAGTTCTTCGTACCAGTAAA

TTACAATCCCTGTTGAAA

>MSTRG.84.1 gene=MSTRG.84

TTCTCCTGTACAAACTCACTTCTTCTTTTTCTCCGTAAAAATGAGAGAGATTCTACACATCCAAGGAGGC

CAATGCGGGAACCAGATCGGAGCCAAGTTCTGGGGAGTGGTCTGCGCTGAGCACGGAATTGACTCAACAG

GCCGTTACAGCGGAGACTCTGAGCTCCAACTCGAACGCATCAATGTCTACTACAATGAAGCTAGCTGTGG

CCGCTTTGTTCCACGTGCAGTTCTCATGGACCTGGAACCTGGTACTATGGATAGTGTCCGCTCTGGACCG

TATGGTCAGATTTTTCGACCTGATAACTTTGTTTTCTGACAGTCTGGAGCTGGGAATAACTGGGCTAAAG

GACATTACACGGAAGGAGCTGAGCTTATTGATTCGGTTCTTGATGTTGTTCGTAAAGAAGCTGAAAATTG

TGATTGCCTCCAAGGGTTTCAGGTGTGTCATTCGTTGGGAGGAGGGACGGGGTCAGGAATGGGGACCTTG

CTTATATCAAAGATCAGAGAAGAGTATCCGGATCGAATGATGATGACTTTCTCAGTTTTTCCATCGCCAA

AGGTGTCTGATACGGTGGTGGAGCCTTACAATGCTACATTGTCTGTGCATCAGCTTGTTGAGAATGCCGA

TGAGTGTATGGTCTTGGATAATGAAGCTCTGTATGATATCTGTTTCCGTACTCTCAAGCTCACCACTCCC

AGTTTTGGAGATTTGAATCACCTGATTTCTGCAACCATGAGTGGTGTCACTTGCTGCCTTCGGTTTCCTG

GTCAGCTTAACTCTGATCTGCGCAAGCTTGCTGTTAACCTGATTCCATTTCCTAGACTTCACTTCTTCAT

GGTTGGCTTTGCACCCCTTACGTCCCGTGGCTCACAGCAGTACCGTGCTCTCACTGTTCCTGAACTTACC

CAGCAAATGTGGGACTCGAAGAATATGATGTGTGCTGCAGATCCTAGACATGGTCGGTATCTGACTGCAT

CTGCCATGTTCCGTGGTAAAATGAGCACCAAGGAGGTTGATGAGCAAATGATCAATGTACAGAACAAGAA

CTCATCGTACTTCGTCGAATGGATTCCCAACAATGTGAAGTCAACTGTCTGCGACATCCCCCCTATAGGT

TTGAAGATGTCCTCGACGTTTATTGGGAATTCCACGTCAATTCAAGAGATGTTTAGGAGGGTCAGTGAGC

AATTCACTGCTATGTTCCGTAGGAAAGCTTTCCTGCATTGGTACACCGGAGAGGGGATGGACGAAATGGA

GTTCACGGAAGCTGAGAGCAACATGAATGACCTGGTTTCAGAATACCAGCAATATCAAGATGCAACTGCT

GATGAGGAAGCTGATTACGAGGAAGATGAAGAGAGTTATGAAGACGAGGCTTAAACTTAGACAGATAGAT

ATTTGGTTTTTGTTTTTGGTTCTTTCGCTTGAAGTTGAAACTTGAAACAGTGTGTTTTTCATTTCCTGCC

TCTGGGATATTTGCTTGATCCTGTGTTTATTTCACTTGCTTCAATCTTGTAAAACTAGCA

>MSTRG.88.1 gene=MSTRG.88

ATTTAATTTAAAAAGAAAAAGAAAAAAAACAAGAAGGGAGTTGCCAGTTGCCACTCTCAATTCAAAGTTC

TTGAAATTTTCTTGTTTGTATTTCATCACCATTTTGTCTTCACACACCCCTCCCCCTTCTTCCCCTCTTT

TCAGTTTCAAATCTAGGGTTTCTGTGTACTCTTTACAGCCCATTCTTCCCTTCAAAATTTTTGAGTTTTG

CTCACATTTTTAATCAAACCCTTCTGTTTTCAACCAAGATTTCTCACTCAGCAGCCACTAGAACTATAAA

GATTCAATCTTTATCATTTTTTAGGGCTTTTAGAGTGGTTTTTAATCAAACCCATCTTTTTTCACTCAGC

AGCCACTAGGAATGTAAAGTTTCAATCTTTATCATATTTTAGTGCTTTTAGAGTGGTTTTTTTTTCATAT

TCTCATGTTTTAGAACAACCCCATTAAAGCTTTTGTCAAGATTTTGGTAAATTGTATTGATATAGAATCT

GGGTTGGTGTTATTTTGTCTTTCTTGAGTGAATTGCTGAGTAAATGCATGGATAGTAGAGAAGAAATGAC

ACTTTCAGGGTCATCACCTTACTATTTTAACGGAGGGATAGGTGGGCCCGTCCTTGATTCACCTGAATTT

AAGGACTTCTCAAACCCTAATGATTCAGTTGAGACCAATGTGATGGTTAGTCATCTGGGTCCTACTCAAA

ATGTTGAAAATTTGGGTACTAATTTTGGTGAGGAGATGAACATGGTTATGAATTCTGGTGGTTATGGTGG

TGGTATGGCTTCGGGGGAGGTTGATTTAAGTAAGAAGAAGAGGGGGAGGCCTAGGAAATATGGGCCTGAT

GGGGCCAATGTGGCTTTGGCATTGTCCCCATTGTCGTCGAATCTTTCAGCTGGTTCGGGTACAGAGGTGG

AGAGGAAGAATAGAGGGAGGCCTAAAGGGAGCGGAAGGAAGCAAAGATTAGCATCTCTTGGCGAATGGAT

GAATAGTTCAGCCGGAATGGCCTTTACACCACACGTCATTCACATTGGAGCAGGAGAGGATATTGCATCA

AAGATTTTACTCTTCGCTCAACAGAGACCAAGGGCTTTATGCATTATGTCTGCAAATGGCACAGTTTCTG

CTGTGACTCTAACCCAGCCTGAATCTTCTCATGACTCTGTCACATATGAGGGCCGTTTCCAGATATTGTG

TTTGTCAGGTTCATACTTGCTCTCTGAGGGTGGCGGACCACGTGATCGAACTGGGGGTCTAAGTGTATCT

GTCTGCAGTCCTGATGGCCATGTGATAGGAGGTGCAGTTGGGGGAAGGCTTATTGCAGCGAGCTTGGTTC

AGGTCATTGTTTGCAGCTTTGTATATGGTGATTCAAAGGCAAAGATCAATCCAAATGGGGCAGCGGCCAA

AGCTGACCTTGATTCCGAAGTGCAACCCACAGAAAAACTATCTACTCCAGGTAGTGCAGGGCCCAGTGGA

GATATTACCCCCAACTTGGCACCAGCTGGTGCTGAAGATATTTCCCTTAAACCAGCAATTGCTGATTGTC

CCCCAGATTCTCAGCCGGGTATTACTGATATAAAAACGGAAATTGACCTAGCCCATGGTTGAGGCATTTA

CTGCAACAATGATGTGGAGAAGTTGATCATACCTTGTTACAAGAGCTTAGAAAATGGGTTTAACATAACT

GTTGATGTAACAGAACGCCATTTTGGGGTTCTCAAAGTAGGAATATTAGGAAGTTGGTGTAGTTAAACGA

GTTATCGTATGTTTTGATGACAATGTAGGTTGGTAGTTGTTGGAAGCTGTTTAGTGGAAACTTTTAGCTA

GGCCTAGTAAACTGTTGATGATAATGTTTGAATTTGTTCTAATTTGTGTCGAATTGTGCTTTGTATGGAG

AAACGTTTCCGCATTTTGCAAATTAGGTTGTAAAATACTCCTATTCATCCTG

>MSTRG.88.2 gene=MSTRG.88

GGGAGTTGCCAGTTGCCACTCTCAATTCAAAGTTCTTGAAATTTTCTTGTTTGTATTTCATCACCATTTT

GTCTTCACACACCCCTCCCCCTTCTTCCCCTCTTTTCAGTTTCAAATCTAGGGTTTCTGTGTACTCTTTA

CAGCCCATTCTTCCCTTCAAAATTTTTGAGTTTTGCTCACATTTTTAATCAAACCCTTCTGTTTTCAACC

AAGATTTCTCACTCAGCAGCCACTAGAACTATAAAGATTCAATCTTTATCATTTTTTAGGGCTTTTAGAG

TGTGGTTTTTTTTTCATATTCTCATGTTTTAGAACAACCCCATTAAAGCTTTTGTCAAGATTTTGGTAAA

TTGTATTGATATAGAATCTGGGTTGGTGTTATTTTGTCTTTCTTGAGTGAATTGCTGAGTAAATGCATGG

ATAGTAGAGAAGAAATGACACTTTCAGGGTCATCACCTTACTATTTTAACGGAGGGATAGGTGGGCCCGT

CCTTGATTCACCTGAATTTAAGGACTTCTCAAACCCTAATGATTCAGTTGAGACCAATGTGATGGTTAGT

CATCTGGGTCCTACTCAAAATGTTGAAAATTTGGGTACTAATTTTGGTGAGGAGATGAACATGGTTATGA

ATTCTGGTGGTTATGGTGGTGGTATGGCTTCGGGGGAGGTTGATTTAAGTAAGAAGAAGAGGGGGAGGCC

TAGGAAATATGGGCCTGATGGGGCCAATGTGGCTTTGGCATTGTCCCCATTGTCGTCGAATCTTTCAGCT

GGTTCGGGTACAGAGGTGGAGAGGAAGAATAGAGGGAGGCCTAAAGGGAGCGGAAGGAAGCAAAGATTAG

CATCTCTTGGCGAATGGATGAATAGTTCAGCCGGAATGGCCTTTACACCACACGTCATTCACATTGGAGC

AGGAGAGGATATTGCATCAAAGATTTTACTCTTCGCTCAACAGAGACCAAGGGCTTTATGCATTATGTCT

GCAAATGGCACAGTTTCTGCTGTGACTCTAACCCAGCCTGAATCTTCTCATGACTCTGTCACATATGAGG

GCCGTTTCCAGATATTGTGTTTGTCAGGTTCATACTTGCTCTCTGAGGGTGGCGGACCACGTGATCGAAC

TGGGGGTCTAAGTGTATCTGTCTGCAGTCCTGATGGCCATGTGATAGGAGGTGCAGTTGGGGGAAGGCTT

ATTGCAGCGAGCTTGGTTCAGGTCATTGTTTGCAGCTTTGTATATGGTGATTCAAAGGCAAAGATCAATC

CAAATGGGGCAGCGGCCAAAGCTGACCTTGATTCCGAAGTGCAACCCACAGAAAAACTATCTACTCCAGG

TAGTGCAGGGCCCAGTGGAGATATTACCCCCAACTTGGCACCAGCTGGTGCTGAAGATATTTCCCTTAAA

CCAGCAATTGCTGATTGTCCCCCAGATTCTCAGCCGGGTATTACTGATATAAAAACGGAAATTGACCTAG

CCCATGGTTGAGGCATTTACTGCAACAATGATGTGGAGAAGTTGATCATACCTTGTTACAAGAGCTTAGA

AAATGGGTTTAACATAACTGTTGATGTAACAGAACGCCATTTTGGGGTTCTCAAAGTAGGAATATTAGGA

AGTTGGTGTAGTTAAACGAGTTATCGTATGTTTTGATGACAATGTAGGTTGGTAGTTGTTGGAAGCTGTT

TAGTGGAAACTTTTAGCTAGGCCTAGTAAACTGTTGATGATAATGTTTGAATTTGTTCTAATTTGTGTCG

AATTGTGCTTTGTATGGAGAAACGTTTCCG

>MSTRG.90.1 gene=MSTRG.90

ATATGTGCCCCTTTCAACGATCGCTCATTTTTATACAGTGTTTTGTATAGCTTGCAGAAGCCGAGTCCTC

TGTTCCAAACGTCTACAATTCGAACGTTTACAATCCGTTGGGACAGAGGGAGTATTGCGATGTCAGGGAA

AGATTTCCATTCGAGCACAAGAATCGAACTATCAACAAAGACCGAAAAAAAATGAAAGTATGGTCACTCC

CCTCCTATGCACACATCTGACTCCAAATGACTAGCTCCTTCAGGCTGAAAATCATAGAAGCTTACTACAC

TTAACTCCATTACCATCTACTAGCATCCAAGAGGCGAGACAGAGTTTACGATCATGAAAGGCACAGGTAA

AGTCCCGATTTCTATAATGTTTGGCGTAAAAGGGATGATTCATGGGTTCAACTCTTTATTCAAACATACA

TCAATATGTATGCGATATTCTTGTTTTGCATTTCTACTCTAATATAATAAGTTAC

>MSTRG.96.1 gene=MSTRG.96

CCAAATTTCAAGTTATCCGGCCTGGCTGCTTCATGAAACTGTTATATTGGTAGTTCTAGGTAAAGGTACA

AATTTTGTAGCAAGTAGATAGTTTAGTTCTTATAGTGTCAACTAGAACTGTCGACCGCTTCTCTCTTGGA

GACTAGTGTTCCCGGACTTTCAATGTATATTTGTTCTATACATGTTACTGAAGAACACACGCTATTACAT

CGAAATATGTTCTGAATCATTGTAATCATTTACGAACTGGGCTCTGTACTTAATTTAAGCATTGGTTTGA

GGCTGTTGAACAAGGTTTTCCAGAGCTTTATACTTATCAGAACTTACAGTTGAATTGTTGGATGAAATTT

AATGTTTTTTCAA

>MSTRG.98.1 gene=MSTRG.98

CCCGAAAGCCCATGCAATGCCTTGAATCCCCACAGAAGCACATTTATCAGGTGCATTCGAGACTCCCATA

ACAGTCAAAACAGAAACATACAAGAACAAGAAAGTGGCTATAAATTCTGCAATGCCAGCCCTATAAAAAG

ACCATGAGACTAGTTCACCATGCTCATACAGAGGTGCTGGTGGTGGCTCCACGTAGTCTCGATCGTGCAT

TGCAGAAGCAGATGTTCCAAGGGGAATCCTCTCAGGGTACTTGTTGGTTCCAACTTTGTCATCCTCTTCT

TCAATCCCCATTGATGCTTTATTTTAGCAAACTTTGTTAAGGGATAAGAAGTGCTGTGAGTTGGTGCTTA

AGGTGAGTGTTTTATAGGTGAAAATTTGAGCAAAATGCAGAACACAAATTTATGCAGATTAATGTAAATT

ATTGGTTTTTTGGGAGGGAGTAAGACAATGTCACAATGGTGGTCTGGAACTGTGACTGTTATCAAATATG

TTTGTTTGTATTAATTTGTTCTTATGTGCTTG

>MSTRG.99.1 gene=MSTRG.99

ACAAACAAACATATTTGATAACAGTCACAGTTCCAGACCACCATTGTGACATTGTCTTACTCCCTCCCAA

AAAACCAATAATTTACATTAATCTGCATAAATTTGTGTTCTGCATTTTGCTCAAATTTTCACCTATAAAA

CACTCACCTTAAGCACCAACTCACAGCACTTCTTATCCCTTAACAAAGTTTGCTAAAATAAAGCATCAAT

GGGGATTGAAGAAGAGGATGACAAAGTTGGAACCAACAAGTACCCTGAGAGGATTCCCCTTGGAACATCT

GCTTCTGCAATGCACGATCGAGACTACGTGGAGCCACCACCAGCACCTCTGTATGAGCATGGTGAACTAG

TCTCATGGTCTTTTTATAGGGCTGGCATTGCAGAATTTATAGCCACTTTCTTGTTCTTGTATGTTTCTGT

TTTGACTGTTATGGGAGTCTCGAATGCACCTGATAAATGTGCTTCTGTGGGGATTCAAGGCATTGCATGG

GCTTTCGGGGGTATGATCTTTGCACTTGTCTACTGCACTGCTGGTATTTCAGGAGGACATATCAACCCTG

CTGTGACCTTTGGTTTATTTCTGGGAAGGAAGCTTTCGTTGACCAGGGCATTGTTCTACATAGTGATGCA

GTGCCTTGGTGCTATCTGTGGTGCTGGTGTTATAAAGGGCTTTCAAGGATCCTCAAAATTTGAGCTCAAT

GGTGGGGGAGCTAATGCTGTGAATCATGGCTACACAAAGGGTGATGGTCTTGGTGCTGAGATTGTTGGCA

CCTTTGTCCTTGTTTACACTGTGTTTTCCGCGACTGATGCTAAGGGAAGTGCCAGAGACTCAACTGTCCC

TATTTTGGCCCCTCTTCCCATTGGCTTCGCGGTGTTCTGTGTTCATTTAGCCACCATCCCCATTACAGGG

ACCGGAATCAACCCTGCTAGAAGTCTTGGAGCTGCCATAATTTACAACAAAAGTCACGCTTGGGACGATC

ATTGGATTTTCTGGGTCGGGCCATTCATCGGTGCCTCACTTGCTGCTGCATACCACCTGGTTGTCATCAG

GGCCTTTCCTTTCAAGAGCAAATCTTAAATTTCAGTCTGCATGTACCAGAACTATTTGTCATCCCTTTTC

GATCTCAGTCTTTCATTTTAGTCTATTGTAATTGTCACTGTCATGTGAATTCGTCGGTTTCAGCTATTAA

AGTATCCATGCACATCATTGTTTTATGCTGTATTTAAGCTTGAGTTTGCATCTGAAGAAGATATTCTTTT

TTTATTGTTTGATTCATAATAGCAACATGTTTCACATCTCCACAAAATTATATATACCTTATTCTGGAGA

CAAGATATTAATAATCA

>MSTRG.100.1 gene=MSTRG.100

ACGCCGTCTCCATCAACGTCCGCTGCTCCAACGGCTCCAAGTTTAGTGTCCGAGCGAGTGTGGGATCCAC

TGTGTCGCAGTTCAAACAAGTTGTCGCGGAGAATTGCAATGTTCCAGCTGTGCAGCAGCGGCTGATTTAC

AAGGGACGGATCTTAAAGGATGATCAAACCCTAGATAGTTATGGCTTAATTGGGTTCTGTAGCTGGGTAA

GTTCATGTGAGACCCGACCATGGCTCAGGTGAGGGGTTCCAATAACCCTAAGTGGTTTGTTACATTATTA

ATTCGATTGAATCTAGTATTGATCCCAAGTTTGTTGAATTCGAGTTATTTGATTTAATTTTATTTGTCGT

CGTTTTTTTATATTTTGATATTCATCCATTTTATATATTGTGTTGTTCTGGTATTGCTAATTTTATGGAT

GTAGGAACTTCTTGATATCTATGGTGGTGATGGATAGGTCCAGAAAGGGTGGAGAAGAAGAGCTATTTTG

GCGATAATTGTGCCTTAATTAAGGATACTAATTATTCTTAATTAAGGTTTTAGTACCGTTTATGGCGACC

ATTTATGGCGACCATTATTGGGGCATTATTGGCCCATAAGAGGCATTTAAGTGCCATAATGTTGGCTCGT

TTATGTCTTTTATTTTAATTTTAAATTTGCTCACATTCGTTCATTCTGTTTTGTCTTATTTTCAGGATGT

TTGAAAAAGCATTTAAGAATTTATCTATTAAGGCGTCTTCCGAGAAGACGCGAATTTTGGTGGACAAGAC

AGTTGAGCTACAAACAAAGATAGAAAGGAATAAAGAAGACTTCATTTTTAGTCTATTTTGTAATCGATGT

ATTTTTTTGTAGGGTACTCTTTTTCCCCCTTGAGTTTTTTTCCCACAGGGTTTTGCTCTTGAGGGGTTTT

AACGAGGCCCTTTTTTTGTGGGTTTTCTCTTCGGACTTGAAAGGTTATGTTATCTAAGCGTCCGGAGTAT

TTACATACTTTCGGTTAGATGATATACCTTCTTGAATGAAGGAAACTCTGTCCATCCGGATTGTAATTTT

TTCTTGGTTTAATACAACCGTTTATTGTGACAAAAAAAAAAAAAAAAGCTTTGTTCTTTTTACTCGCATA

ACAACTCTGATTGGGAGTGTACTACCAAACTTCTCTTGATATTGTGATGGACCCCACACGTATTGTATCC

C

>MSTRG.102.1 gene=MSTRG.102

TTGTAGTGTGGATTCTAAACTGATTGTTTCACAGATGGAAGTGAAATAAGTTAGTGCAAGACCAATGCTG

CTTGTCGGTCAGTGATCATCTGTTTACTAATCATTTCTGCAGAAGCATTCCTGCATCTGGAACGGATGAA

ATTCATGTTAGTATCATATTTATATGATTTTACCTTTTGTTTTAGACGGAAATTATGTACATTAACATTT

TTTCCGAGCTATTGGTAGGCATTTGGTATTAGAATATGTTTCTGTTCTTCTGACAGTTTCCTTGTTTAAT

TCCGTTTCATGTTTAATTCCGCTCTTTCGAGAGTTCGGAGAAAAGTACCCGGGCCTTAAAACAGACACTC

AACCGGAAACAGATGAAATTTTCGGCTGGACCTTATGGTCAGTTCAAAACTGTTCTTGTGTAGGCTCTTG

AGACTTGATGAAGTGATGTTCATG

>MSTRG.104.1 gene=MSTRG.104

CAACTACTTTCCTCCACTATCTTCATTCTATAATAATATAAACACTATTACACCCACTACTTTCTTCCAC

TATCTCAAATCTATTATTAAATATAAATGGGTCCCACCACTATACCCATTTTTCATCTAACTTTACTCAT

TTCTGACCCATTTTCTTGGTCTCCGTGTCCCAGCCATTTGTATACAAATGACTGGGACGGAGGGAGTATT

A

>MSTRG.106.1 gene=MSTRG.106

AAATATGTTGAAATACAAGTCCTGTTCATACCCTTACTTGATACTAACAACTATACTAGACTTTTATGGC

AATTGTAGTTGGGAGTTTAGCAGTTGAATAGTACCAGTAACTAGCACACGGTTTATTTATGTAGCATAGA

CTTTCTGTACATTACTTACTCTATATATGTAGCTCTTGGGCATGTAATAGCAATACACACAGAACAGTAA

TACAAGAG

>MSTRG.108.1 gene=MSTRG.108

CTTCCGCGGCCTCTCCTTCATTCTTGGCTTCCGCGGCCTCTCCTTCATCCTCGGCTTCCGCGGCCTCATC

TCTCCTTCATCCTCGGCTTCCGCGGCCTCATCTTCATCAACTCAGTCTTGGCTTCCGCGGCCTCTCCTTC

AGCAACTCAGTCTTGGCTTCCGCGGTCTCTCCTTCAGCCTCGGCTTCCGCGGCCTCATCTTCATCAACTC

AGCTTCGGCTTCCGCGGCCTCATTCTCAGCAACCTCGCCATTGTCTCTATCAAGCAAACACTGCCTTCAC

CCCCGTTAGGCAGAATCATCTTCATCTTCAAACACCTCCTTCAACATCGGCTTCAGAGGAAATTTCTTCA

ACATAAGGCGCGCCAAGTTAAACAACGTCAACATCTTCTGCATCAGCGAAGAACAACCAAGATAGGACAT

CTCAGGAGATGAAGGACATCAAGATCTCGTCGACATCTTCAATGCCACCAAGATCTGGGGGGTAGTTGTT

ATACGCAAAAATCACCATGAAGACCCGGCCCATAAAAGAAGACCATGATGGGCTCAAGAATGGACCAAGC

CCAAGCTAGCCATGTCAGTTAGAAGAGACCACTTCAAGAAGATTGCGCGCCTTATCTTCAGGGCACGTCA

AGTTGAAGCTTACAGAGAAAGATCGT

>MSTRG.109.1 gene=MSTRG.109

GAAAAATCGATCAGAATTAGGAGAAACGTATAAAAATTTTGCCACTATGATTGAAACTCAATTCAAAAAG

AAAATCAAGCTTTTTCGTGCCGATAATGCCAAAGAATATAAAGACTCAGAACTTACCAAATTTTTGGCAT

CTTATGGTACTCTTGCACAAAGCTCTTGTCCTTACACATCACAACAAAATGGTCGTGCTGAACGTAAACA

TAGGCACATTCTTGATACAGTTCGAGCTCTTCTCATTTCTTCTTCTTGTCCAGAATTATTTTGGGGAGAA

GCTGCATTAACTGCTGTTTACACAATAAACATGATTCCTTCTCCCGTTATAGATAATGTGTCACCTTATG

AAAAATTATATGAGGAACCTCCGTCATATGATTCTCTTAAGGTTTTTGGTTCTGCCTGCTTTGTTCTTCT

TCCTCCCCATGAAAGAACAAAACTAGAGCCACGTGCCCGTCTTTGTTGTTTTATTGGTTATGGTAGTGGT

CAAAAGGGATATCGCTGTTGGGATCCTATCTCTCAACGTCTTCGTGTCTCTCGTAATGTGACATTTTGGG

AACACAAAATGTTTTCATCTATGTCAGGATTTAAAATTGACACGTCGACTAGTATTGCCCACTTTACAGA

TCCTTTTGTTGATCTACACCCC

>MSTRG.110.1 gene=MSTRG.110

AGTGTTCAAACTTAACAAGGCTCTTTATGGTCTTAAACAAGCACCTCGAGCTTGGTTTGAAAAATTCAGC

AAAATAGTTCAACAATTGGGATTTTCTTCAAGCCCATATGATCATGCTCTTTTCATACGAAGGTCTGAAC

GAGGAATTGTTTTACTTCTTCTATATGTCGATGACATGATCATTACTGGAGATGACGTCAAAGGCATTTC

TGAACTTAAACAATTTCTCAGCCAGCAATTTGAAATGAAGGATCTTGGTTCACTAAGCTACTTCTTGGGT

CTTGAGGTTTCTTCTGTCCCTACTGGCTACACTCTTTCACAAGTAAAATATGC

>MSTRG.111.1 gene=MSTRG.111

GGTTTTCGTGTTAAAATCAGTTCTCTTCAGTCAGTGATCCCGCGGAAAATGAAGAAGGTCGTCGTGAAGT

TGGATATATTTGATGAAAAAGAAAAGAAGAAGGCCTTAAAAACGGTTTCCAGTCTCTCAGGTACCAATAA

TTATAAATGTAACATCTTGTACGTGGTACAGAAGACTCCCGCGCATAGATTACTGATAAGTGGCACTGTG

TTTGTAGGAATTGCGTCCATATCAGTAGATATGACGGATAGGAAGCTGACAGTGATCGGAGATGTTGATC

CTATTGGCGTGGTGAACAAATTGAGAAAACTATGGCACGCAGAATTAGTATCGGTTGGTCCTGATAAGGA

ACCGGAAAAGAATAAAGATGATGAGAAGAGTAAAGAAGAGAAGAAGAAGATTGAGGACAAGGAGAAGGAA

GAAGCGGAAAAGAAGAAAAAACAAGAAGAACAGATTCAAAATCTTCTCAGTGCCTATGGCTATCAGAATT

ACGCTCCTTATAATGTTATGCCCCAATACGTTGTTCAAAGTGCTGATGATTATCCACATTCTTGTGTTAT

CAGCTGAATAATTGTTTCGAGTTTATGAATGTAAATAAAAAACCACATGTAATTTTTTTTGGTTGTCGGA

AAATTCCATCTGAGTTTAAACAAGTGTTTGATCTCCAATTGATAGGTTTTGGTTGATTTGATCGAATCTT

CATTGGCCATGATGCTATCTGGTCTGCATTGTTGTACGATACAAGTTCCCTATCGGATGATTTTCTTGTA

CAATTCTGTTTCGTCTCTTTTTTGG

>MSTRG.114.1 gene=MSTRG.114

CTATGTCGATATAAGACTGAACTCAATTTCGAGCTCGAACCAGAGGCGATGCAAGAGGAGAATGAGGAGA

CATTATCATATGAGGAGACATCAAATGTGGAGTCACTATTCTTGGTGTCTTCATAATACTCTCATCCCAC

CTTCTGAATGACAAGCTGAGCTGCAGATTCTTATGCCACATGTGCTTCTTAGCTGCCGCTTCACGTTCAT

CAGCCTGAAGCACAAATTTCTTGATCCGTCTCAGCGCGATCCTCATCGTCTCATCATCCATATTTGCATA

GCAGACTCTGAACCACCCTGGCTCTGAGCAATGAAAGGAGGATCCAGGGGACACATTGAGCTTAACTTCA

TTGATAATTACGCGCCATAAGTCCATTTCAGCTTCGACTGTGGACTCTTTGAGTAAACGTCGTAAATCCA

TCCAGAAGAAAAGGCCTGCATTGCTCTGTAGGTTTCCTACCCGGACCTGCATTAGGCCTCTGGTGAAAAC

GCCATGTCGTGCAGCTAGTCTTTTCCTGCTTTCGGATATGTATTTATTTATGAATGAATCGTCTGACAAC

ATGTTTGCGATTAATTGTTGAGTCTGAGTTGAGACTAATCCAAAACTTGACATTTTTCTAGCAGAATTCA

CAACTTTGTCATTGTAGGAGTAAATGATCCCAATTCTGAACCCGGGGAAGCCCATGTCTTTCGACAAGCT

GTACACCAGATGAATCAAATCGCGGTTGCATTTCTTGTCCTCTTCGAGTATCTCAGCAATGCTGACATAC

ATTGGCCTGGTAAAAACCGTGGCAGCATAAATTTCATCGCAGATCAGGTGGATGTTTTTCTCATTGATAA

AATTCACTAGGCCTTGTAACGTGTCACGGTCCAAAATTGTGCCTAATGGATTCGAAGGATTTGTGATTAG

CAAGCCTTTAACGTTGATATTCGACTCCTCTGCTTTCTGATACGCTTCTTCCAGGGCTTCTCTGGTGATC

ATGAAATTATTCGAGCTCTCACAGACAACTGGAAGGAGTTTTACTCCAGTTCGCCACCTCAAATCGCGGT

CAAATCTGCAAAACATCGTAATAAAAATGTAAATTTCTGAGTTTTTTT

>MSTRG.119.1 gene=MSTRG.119

CAGGATGAAGAATAGGCATAGTTATCCTAAAGACGTTGAGGCTTGCATTAAGAAATATGGATCTTCACTA

CCCAAAAGATTCAGATACTCTGCCCTAAAAAAATAACAAATTCATTTAAAGAGGAATTAGGCAAAGGCGG

GTATGGAAACGTATACAAAGGTAAGTTACCAGATGGTTGTGTCGTGGCTGTGAAGGTTTTAATTGAAACC

AAAGGAAACGGGGAGGAATTCTTGAATGAAAATGCAAGTATTGGCAGGACTTCTCATGTCAATGTTGTCT

CTCTGCTCGGGTTTGTCTACGAGGGTAAAAGGCGAGCCCTGATATATGAATACATGCCCAATGGATCTCT

AGAGAAGTTCATCCATGGCACTACTCCGTCGTTGAAAGGACAGAATCTCAGCTGGGAGAAGCTGTACAGC

ATTGCACTTGGAATAGCTCGAGGGCTAGAGTACCTGCATTGTGGCTGCACCGCACAAATCCTACATTTTG

ACATAAAGCCGCATAATATTCTTCTTGACCAAGATTTTAGCCCCAAGATATCAGACTTTGGGCTTGCAAA

GATGTACACCAGAAAAAAAGTGTGTTATTTTCTATAATGCAAGCTAGAGGAACCATTGGCTATATTGCTC

CTGAAGTAATTAGTAGAAAAGAAATTTCGGGCCAGTGTCACATAAGTCTGATGTCTATAGCTACGGAATG

ATGACTCTGGAAATGGTTGGAGGAAGAAAGAATGTGGATGCTAGAGCAGCTCACAGTAGTGAGATATATT

TTCCTAGATGGGCTTACAAGCATATCCATGATAATGAGGATACTACTAGTACTGCAGATAATATAACAGA

AGAAGATAATATAACAGAAGACGAAAACCATGTTGCCAGGAGGATGATGATCGTGGGACTGTGGTGCATT

CAGGCCAATCCGTCACAAAGGCCATCAATAAGCAAGGTGATTGAAATGTTTGAAGTGGATTTAGCAACTT

TGGAAATACCAGCTAAGCCTTACTTGTGTTCTGCTCCCACTTCACCGCGGCATTCTCAGCAGAAGGCTCT

TTCCGGATCTTCTTCACTAAGTGAATCCACTGAAACTAGTTCGAATTAATATCAGATGAGAATTATATAA

CTCCATCGACTCATGTAATGTGTTTCCTTGAATCTTGTCTAGTGCCAAGTTAGCTAAGCTAGGTGATCAA

TTCGAC

>MSTRG.125.1 gene=MSTRG.125

CAGTCACTTAGCCCCCAAGGTAGGGAATTGGTATTGATCTTCGATTATCGGGGCAGGTACATTGTTCCCA

AAGTTTCCAGTGTATCCTCCTCTTGGACCACGGCCGCGTCCACGTCCACGACCAGGGCCATAGTATTTTT

CTCCTTCAGCAGGCTTTAGAAACTCATTAATGCTGAGAGCCTTTCTGGCTTTCTCTTCCTTTTCAGCCTC

CTTACGCTTTTCCTTATCAGACCCCAATTTGACAAAGATCTCCTCTTCATTTTTCTTGCTTGAAAGAAGC

TGCATTGATTCAAAATCTTTGTCCAAGTCAACCTTTCTCTCTTCAGATTTAAGGGCCAGCAGTGCCTTTC

TCTTCTCCTCACGCACCTTCTCATACTCCTCCAGAGTCATTTCCTCAGGCTCCTTCTCTTCAGGCTCAGT

CACAGGATTCTCCTTGCTTGCATCACCAGCATCTTCCTGCTCTGCTTGTTTCTCAACATCAACATTCTTC

TCACCATCATTGACAAGCTCTTCGTTTACC

>MSTRG.129.2 gene=MSTRG.129

ATAACTTTAATGTTGCTTAAAGCCTTCCACTCTATTCCATTCTATTCCACTCATTCTCAACGAAGACAAC

CTGATGATAATTCTCCAAACTACCCTCTTCCAGCCATTATATAAACAAATTGAAATCAGACTCGAAGCAT

TCTTACGACCCAGCTACATATATTCCTCACATCCTCCTCTCATTCCTTCATCTTATCTTCACACTCTTAG

AGTCTGAGAGAGTCATGGCCTTTTCACATGCCATGCTCATGCTAACCTTCTCACTTACGTTCGTTGTCTT

ATCCTCTGGTCAAGCTCCGGCAATGTCACCCTCCACCACCATGCCACCTATGATGATGACACCACCTCCA

ATGGTGACTCCACCTCCTATGATGATGACACCACCTCCAATGATGATGACACCTCCTCCAATGATGATGC

CAGCACCAATAGCTAGTCCACCTGAGATGATGACTCCAGCCATGAGTCCTGATTCGATGAGTTCACCACC

AGCACCAATGGGACCTGCAATGGCTCCGACCCCAACCATGACGGGTATGTCACCCGTGGAGTCTACCCCA

GGGTCAGCAGCTTTCAAGCAGGGAAGTAGCCTAGCCATGCTAGCATTATTGGGAGGAGCTGTACTTCTGG

TTTAAAACTTTTGTGTATTATATTGTGTCTCTTTTGTTTTGGTCCTCGTGGAGTTTTAATCTTTGTGTTT

TATCTTGGAGGTCAGTATGTGGTCTGTTGAAGATTTATTTAATGTTACAACTCTCTTGTAGCATTTTAAA

CTAGGTCAATAAGAATACTGTTATTCAGTTTAATCTCGTAATTGTTACTGAGTCAGGAATTACACAGTTA

TGTAAAGTCTATATATTTTTGCTTTAGAGGTATTGTTTTCAGCTATTTGTTGTGTCTCTCTTACCGTCGT

TCAGTACTGCGAATACAATATCCTTGCTACGTG

>MSTRG.129.3 gene=MSTRG.129

GACAACCTGATGATAATTCTCCAAACTACCCTCTTCCAGCCATTATATAAACAAATTGAAATCAGACTCG

AAGCATTCTTACGACCCAGCTACATATATTCCTCACATCCTCCTCTCATTCCTTCATCTTATCTTCACAC

TCTTAGAGTCTGAGAGAGTCATGGCCTTTTCACATGCCATGCTCATGCTAACCTTCTCACTTACGTTCGT

TGTCTTATCCTCTGGTCAAGCTCCGGCAATGTCACCCTCCACCACCATGCCACCTATGATGATGACACCA

CCTCCAATGGTGACTCCACCTCCTATGATGATGACACCACCTCCAATGATGATGACACCTCCTGAGATGA

TGACACCACCTCCAGTGATGATGACACCTCCTCCGATGATGATGCCAGCACCAATGGCTACTCCACCTGA

GATGATGATGACTCCAGCCATGAGTCCTGATTCGATGAGTTCACCACCAGCACCAATGGGACCTGCAATG

GCTCCGACCCCAACCATGACGGGTATGTCACCCGTGGAGTCTACCCCAGGGTCAGCAGCTTTCAAGCAGG

GAAGTAGCCTAGCCATGCTAGCATTATTGGGAGGAGCTGTACTTCTGGTTTAAAACTTTTGTGTATTATA

TTGTGTCTCTTTTGTTTTGGTCCTCGTGGAGTTTTAATCTTTGTGTTTTATCTTGGAGGTCAGTATGTGG

TCTGTTGAAGATTTATTTAATGTTACAACTCTCTTGTAGCATTTTAAACTAGGTCAATAAGAATACTGTT

ATTCAGTTTAATCTCGTAATTGTTACTGAGTCAGGAATTACACAGTTATGTAAAGTCTATATATTTTTGC

TTTAGAGGTATTGTTTTCAGCTATTTGTTGTGTCTCTCTTACCGTCGTTCAGTACTGC

>MSTRG.132.1 gene=MSTRG.132

CCGCATTTCATACAAACAAACCCCAGGAGTGCCTTCGTTTTTTTGCTTTACCGAGTGCGAGCTTGTAATT

GTTGGGGATGATTTCAGTGGTGAAGAAAGATTGGCCTGATGAGGATGCGATGAAGTTGTCGTTTGAGACT

GGCAGTGCTGTGAAGGTCAAATTTAAGCGCAGGAAAGTACCAGTTGTTAGGGATTTTCCTTTGGGATGTG

GACCAAATGCACTAAAGGTTAGTGATGTTAAGGGTTCTTCGCAGAGTGGATCAAATGCAATGAAGGTCTC

TGCTATTGGGGGATTTCCTGCGGGGAATGGGCAAAATGCAGTAAAGGTCTCTGCTGTTAGAGATTTTCCT

GCGGTGAATGTTTTGAATGCACTAAAGGTCTCTGCTGAGGATTTTTCTTTGAGGAATGGACTTGGTGAAC

AAATGGCATCTGGTGTTAAAGATTCGCCTGCAGGGAGAGGACCGGATGCACCAAATGCCAATGCTGCTGT

AGAATCTCCTACGGAGAGCGGATCAAATGCAATGAAGGGCTCTGCAACTGGCGAATTATCTGTGGGTAAT

GGACCAAATGCACTAAAGGTCCCAGGTGATAGAGATTCTTCTGCAGTGAATGTTTCGAAGGTCTCTTCTT

GGGATATTCCTGCGGGGAATGGATCAATTGAACCATTGGTCTTGCCTTCTAGGGATTCTCCTGCAGGGAG

GAGACTGAATGCACCAAAGGTCAATGTTGTTACAAATTTTCCCACGGTGAGCGGATCAAATGCAATGAAG

GTTTCTGCTATTGGGGAATTCTCTTCAAGAACTGAACCAAGTGCAATGAAGGCTCCTGCTGTTGGAGATG

TTTCTGCAATGAATGCTTCAAATGCTTTAAAGCTCTCTGCTATTGGGGATATTCCAGTGGCGAATGTACC

AAGTGCATTAAAGGCCTCTGCTGTTAAAGATTTCTCTGGAGGGAATGGACCGAAATCATCAAGGGTCTAT

GCTGTTAGACGAGGTTTTCCAGTTGTTCGGGATTTCCCTCCAGGATGTGGACCAGGTGTTAGAGGAACCA

GACTAAAAATACAGTAATGTCAAATGAAAAAACATGTTGTACAGGAGAGAAGTCACAACAGAACTTAGAG

ACCAACTAAGAACTCATGGCATCCGTGGAAAGTAACTGTCTTCTGAAACTTTTAAGATCTTTTGCTAAAT

ATTTCATTACACAGTTCCTCGACAGACAGCTGCTCCAGCGGAATCAAACTTTTATTGGTCAAGCTCAGTA

CAAAAACATATGCAGCTTAATATACCAAAAACATTTCACATCCTGACAATATCCATATCCAGTCAACACA

AGTTATGATCACTACTCTAGACTTCTTATATGATATACATATATGATAGGCTTCTTATATGATTATCCTT

TTAGTTTTGAAATTATGCATATATGATATGGCTGCCGGTC

>MSTRG.132.2 gene=MSTRG.132

CCGCATTTCATACAAACAAACCCCAGGAGTGCCTTCGTTTTTTTGCTTTACCGAGTGCGAGCTTGTAATT

GTTGGGGATGATTTCAGTGGTGAAGAAAGATTGGCCTGATGAGGATGCGATGAAGTTGTCGTTTGAGACT

GGCAGTGCTGTGAAGGTCAAATTTAAGCGCAGGAAAGTACCAGTTGTTAGGGATTTTCCTTTGGGATGTG

GACCAAATGCACTAAAGGTTAGTGATGTTAAGGGTTCTTCGCAGAGTGGATCAAATGCAATGAAGGTCTC

TGCTATTGGGGGATTTCCTGCGGGGAATGGGCAAAATGCAGTAAAGGTCTCTGCTGTTAGAGATTTTCCT

GCGGTGAATGTTTTGAATGCACTAAAGGTCTCTGCTGAGGATTTTTCTTTGAGGAATGGACTTGGTGAAC

AAATGGCATCTGGTGTTAAAGATTCGCCTGCAGGGAGAGGACCGGATGCACCAAATGCCAATGCTGCTGT

AGAATCTCCTACGGAGAGCGGATCAAATGCAATGAAGGGCTCTGCAACTGGCGAATTATCTGTGGGTAAT

GGACCAAATGCACTAAAGGTCCCAGGTGATAGAGATTCTTCTGCAGTGAATGTTTCGAAGGTCTCTTCTT

GGGATATTCCTGCGGGGAATGGATCAATTGAACCATTGGTCTTGCCTTCTAGGGATTCTCCTGCAGGGAG

GAGACTGAATGCACCAAAGGTCAATGTTGTTACAAATTTTCCCACGGTGAGCGGATCAAATGCAATGAAG

GTTTCTGCTATTGGGGAATTCTCTTCAAGAACTGAACCAAGTGCAATGAAGGCTCCTGCTGTTGGAGATG

TTTCTGCAATGAATGCTTCAAATGCTTTAAAGCTCTCTGCTATTGGGGATATTCCAGTGGCGAATGTACC

AAGTGCATTAAAGGCCTCTGCTGTTAAAGATTTCTCTGGAGGGAATGGACCGAAATCATCAAGGGTCTAT

GCTGTTAGACGAGGTTTTCCAGTTGTTCGGGATTTCCCTCCAGGATGTGGACCAGGTGTTAGAGGAACCA

GACTAAAAATACAGTAATGTCAAATGAAAAAACATGTTGTACAGGTAGATTATTGGCGTCACTTTTGTGT

CCTATGATGTTCTACCTCTGAAAATTTTGAACAAAATCCTTACATGCGCCGTTCATATTTCAGGAGAGAA

GTCACAACAGAACTTAGAGACCAACTAAGAACTCATGGCATCCGTGGAAAGTAACTGTCTTCTGAAACTT

TTAAGATCTTTTGCTAAATATTTCATTACACAGTTCCTCGACAGACAGCTGCTCCAGCGGAATCAAACTT

TTATTGGTCAAGCTCAGTACAAAAACATATGCAGCTTAATATACCAAAAACATTTCACATCCTGACAATA

TCCATATCCAGTCAACACAAGTTATGATCACTACTCTAGACTTCTTATATGATATACATATATGATAGGC

TTCTTATATGATTATCCTTTTAGTTTTGAAATTATGCATATATGATATGGCTGCCGGTC

>MSTRG.132.3 gene=MSTRG.132

CTCGAACTCTCGACTTCTTGTTATCGAGACGGAGTGCCTTCGTTTTTTTGCTTTACCGAGTGCGAGCTTG

TAATTGTTGGGGATGATTTCAGTGGTGAAGAAAGATTGGCCTGATGAGGATGCGATGAAGTTGTCGTTTG

AGACTGGCAGTGCTGTGAAGGTCAAATTTAAGCGCAGGAAAGTACCAGTTGTTAGGGATTTTCCTTTGGG

ATGTGGACCAAATGCACTAAAGGTTAGTGATGTTAAGGGTTCTTCGCAGAGTGGATCAAATGCAATGAAG

GTCTCTGCTATTGGGGGATTTCCTGCGGGGAATGGGCAAAATGCAGTAAAGGTCTCTGCTGTTAGAGATT

TTCCTGCGGTGAATGTTTTGAATGCACTAAAGGTCTCTGCTGAGGATTTTTCTTTGAGGAATGGACTTGG

TGAACAAATGGCATCTGGTGTTAAAGATTCGCCTGCAGGGAGAGGACCGGATGCACCAAATGCCAATGCT

GCTGTAGAATCTCCTACGGAGAGCGGATCAAATGCAATGAAGGGCTCTGCAACTGGCGAATTATCTGTGG

GTAATGGACCAAATGCACTAAAGGTCCCAGGTGATAGAGATTCTTCTGCAGTGAATGTTTCGAAGGTCTC

TTCTTGGGATATTCCTGCGGGGAATGGATCAATTGAACCATTGGTCTTGCCTTCTAGGGATTCTCCTGCA

GGGAGGAGACTGAATGCACCAAAGGTCAATGTTGTTACAAATTTTCCCACGGTGAGCGGATCAAATGCAA

TGAAGGTTTCTGCTATTGGGGAATTCTCTTCAAGAACTGAACCAAGTGCAATGAAGGCTCCTGCTGTTGG

AGATGTTTCTGCAATGAATGCTTCAAATGCTTTAAAGCTCTCTGCTATTGGGGATATTCCAGTGGCGAAT

GTACCAAGTGCATTAAAGGCCTCTGCTGTTAAAGATTTCTCTGGAGGGAATGGACCGAAATCATCAAGGG

TCTATGCTGTTAGACGAGGTTTTCCAGTTGTTCGGGATTTCCCTCCAGGATGTGGACCAGGTGTTAGAGG

AACCAGACTAAAAATACAGTAATGTCAAATGAAAAAACATGTTGTACAGGAGAGAAGTCACAACAGAACT

TAGAGACCAACTAAGAACTCATGGCATCCGTGGAAAGTAACTGTCTTCTGAAACTTTTAAGATCTTTTGC

TAAATATTTCATTACACAGTTCCTCGACAGACAGCTGCTCCAGCGGAATCAAACTTTTATTGGTCAAGCT

CAGTACAAAAACATATGCAGCTTAATATACCAAAAACATTTCACATCCTGACAATATCCATATCCAGTCA

ACACAAGTTATGATCACTACTCTAGACTTCTTATATGATATACATATATGATAGGCTTCTTATATGATTA

TCCTTTTAGTTTTGAAATTATGCATATATGATATGGCTGCCGGTCCACAACATACGAGTGATTCTAGCTA

AATCGAAAAACCTCAAAATGAGACAATTTTATGTTGAATGATACTGTAGCATCCTCTATATGTACATAAA

AAATTTATGGTTAATTTGTCCAGCATCTTTTTTT

>MSTRG.147.1 gene=MSTRG.147

CGATCAAGCGGTTAAAACATCAAATGTACCTCCGTCACGCGAGTGGCGTGCCCGAAACCGTGACCGGTAA

CATAATACGCGAAGATCAGCCGATTCGCACCTGCTGAACCATTAACAGCCCGATCTCCTCCTTCAATAAT

CATCCTCTCCGACTCAAACCAGATCAAATTTTCGATACCGATGAAGAAAACGTAGATTTTGTGATCAATG

TGGATGTGAATGCGCAACAATGAGAGCTTATTCGAGTGAATTTATAGAGGGTTTAGGTGAGGGAGACGCT

GACGTGGATTTGGGGCTTATCTGTTATGAAGTTAGTAACTGTTATTTGTGTGTTTAATTGCGTGAATAAA

GAAAATTCTGGTGATGCATCTGCATATTCTCCGGCCCATTCTTCTTGAAAGTGATGCCCGTTGGATTGGA

TCGAGTTTGGATAAGGGGCTGTGGGAATTAATGGGCTTCTTGGTTTTTTGCGGGTCTTGGGTTTGGAGGA

ATAA

>MSTRG.164.1 gene=MSTRG.164

CTAGATTTACAATCTTCCTGACAGATCTCGAGTCCTGAAGTTTGACAGTGGATTTTTTCTCGACACCGAG

AACGATAGTATCGGTGCCTCTGACGCCCACGGCGGCGTTACCTTTGCGGACGGCTTCAAGTGCGTATTCA

ACCTGAAATAGATGACCGTCCGGTGAAAAGACAGTGATTGCTCTATCGTATCTTGCCATCTTGGTTCCCT

TTGTG

>MSTRG.166.1 gene=MSTRG.166

GAAAAAATAAGAGCGGCACTTTAAAAAAATAAGCGGGTTAGGGTTCTCTCTCAAAGGGGGCAACAAATCT

CTCTCAGGTGGCTGTATCTTCAGGTTTTGGGTTTTTGTTAACTTCTTGGTGGATATTCTCGGTTTTCTCT

TCTCTCAGGTTTTGCGTAAGATCAAGTTTAATAGGCTTGGTGTTGAGGATCTTAGGTTTGGAGTAAATTA

ATAGTCCGAAAAGTCGGGTTCTTTTAAATATGAGAATGAACAAATGCTCAAGGAAGGATTATATCAGCGA

TCTGCCCGAGAGCATCGTCGATATCATCCTAACCAAACTGCCAATAAGGGATGCTGTAAGAACCAGCATT

TTGTCTACCAAGTGGAGGTATCAATGGGCGACCATGACACGAGTCGTATTTAAAAACCACGTGCCATATA

AGAGTGACAACAAACTTGCCGAGCAAGAAATTACAAACTTTATCATGCGATTTCTATTTCTTCATCAGGG

CCCTATTTATAAGTTCAAACTATCTACTAACTGCTCCATGAATTCTACTGACATGGATCAATGGCTACTT

TTCCTATCAAGGAAAGATATAAAAGAGTTAGTTCTGATGTTAGACGCCAACTGTCTGAAGATACCCTCAC

ATATATTTTCTTGTCAGAAACTGACCACATTGAAGCTTCAAGCATCGGACGTGAAACCTCCTTTAAGATT

TCGTGGATTGCCATGTTTGAAGTACCTTAACCTATGTAGTGGCTTGTTTACTATTGAGGATATTGAAAAC

CTTATTTCAGGTTGCCCTCTTCTAGAAAAGTTTACATTCACAAATGTTGGGGACCCTATGGGTTTTGCCA

TCCATGGCCCAAATTTGAAGCATCTTATTTTGAATGGGGACTTTTCAAACTTAAATCTTGAGCATTCTCC

GTTTCTGGATGTCTTAAGGGTTGACTTTCGTGCACAGGTTTGGGAAAGTAACGTCCTTATGAAAGTTCCA

GTTACATATGATCGGCTAAAGTTTATTGATCTTCAAGGAATAAATTACGAAGAGGGGAATGCAGTGTTGT

ATGTTCTTCACTTGATTTTGCATTCCCCTAATCTACAAGAACTGGAAATTGGAGCTACACAGTTTGAAAG

TTCTCATGATAAAGCTGTTGACTTGGATTTTTGGGAGAAAGAATGTCCTACCGATTTCACATTTAAGCAT

CTTAAATTAGTTGAGATGTGTGGTTTGCCCAACAAAAGTTGTGTGGAATTTCTCAAATTTGTGCTTGGAT

GTTCTCCGGTGCTTGAAGTAATGAGAGTGTCACTTGATGAGGCTTACGATGGAGAAATGAATATGGCGAA

TGAACTGCTGCATTTTCAATGTGCTTCTCCAAAAGTTGATATAAAATTCTTTGATTAGTCATCTGAAGCA

TACTTATGCGTTTCCTTCCCCTTTTGTTCTTAGCTGCTTTATTTCGTCATGTCTCAATTTTTTATTTTTT

TTGTGAGAGTCAAGTCTCAAATTTAGTTGCAAAAGAAATCTGTACATTAGAAGAATAAGCTTTTGCAAAT

TGGGGACTCTTCAAGACTCTAATGACATGATTTTTTTTTTTTTTATGGATGACGCCTTAACAGGGAGAGC

ATTCCTTAGTCTTTATCAAGACTTCAATCTCTGAACTCTTTCCATGCTTGGTGGATTTTCCGGTAATGAT

GAATTGAATCGACTATTAAAACAGTAAAAACAACTTTGATGATGTATCTCAGAACCACGCTTCCTGACAA

TCACTATTCTTCTGATGTTAGATTGGATATAAACA

>MSTRG.166.2 gene=MSTRG.166

ATATCTACACACACCGTGTAATCCAAGAATATGAGAGCTAAGAATAAAAGAAAAGAAAAACTCCAGGCTG

CGTGGCGAGAAGAAAACTAGAAAAGAGGTTTTGCGTAAGATCAAGTTTAATAGGCTTGGTGTTGAGGATC

TTAGGTTTGGAGTAAATTAATAGTCCGAAAAGTCGGGTTCTTTTAAATATGAGAATGAACAAATGCTCAA

GGAAGGATTATATCAGCGATCTGCCCGAGAGCATCGTCGATATCATCCTAACCAAACTGCCAATAAGGGA

TGCTGTAAGAACCAGCATTTTGTCTACCAAGTGGAGGTATCAATGGGCGACCATGACACGAGTCGTATTT

AAAAACCACGTGCCATATAAGAGTGACAACAAACTTGCCGAGCAAGAAATTACAAACTTTATCATGCGAT

TTCTATTTCTTCATCAGGGCCCTATTTATAAGTTCAAACTATCTACTAACTGCTCCATGAATTCTACTGA

CATGGATCAATGGCTACTTTTCCTATCAAGGAAAGATATAAAAGAGTTAGTTCTGATGTTAGACGCCAAC

TGTCTGAAGATACCCTCACATATATTTTCTTGTCAGAAACTGACCACATTGAAGCTTCAAGCATCGGACG

TGAAACCTCCTTTAAGATTTCGTGGATTGCCATGTTTGAAGTACCTTAACCTATGTAGTGGCTTGTTTAC

TATTGAGGATATTGAAAACCTTATTTCAGGTTGCCCTCTTCTAGAAAAGTTTACATTCACAAATGTTGGG

GACCCTATGGGTTTTGCCATCCATGGCCCAAATTTGAAGCATCTTATTTTGAATGGGGACTTTTCAAACT

TAAATCTTGAGCATTCTCCGTTTCTGGATGTCTTAAGGGTTGACTTTCGTGCACAGGTTTGGGAAAGTAA

CGTCCTTATGAAAGTTCCAGTTACATATGATCGGCTAAAGTTTATTGATCTTCAAGGAATAAATTACGAA

GAGGGGAATGCAGTGTTGTATGTTCTTCACTTGATTTTGCATTCCCCTAATCTACAAGAACTGGAAATTG

GAGCTACACAGTTTGAAAGTTCTCATGATAAAGCTGTTGACTTGGATTTTTGGGAGAAAGAATGTCCTAC

CGATTTCACATTTAAGCATCTTAAATTAGTTGAGATGTGTGGTTTGCCCAACAAAAGTTGTGTGGAATTT

CTCAAATTTGTGCTTGGATGTTCTCCGGTGCTTGAAGTAATGAGAGTGTCACTTGATGAGGCTTACGATG

GAGAAATGAATATGGCGAATGAACTGCTGCATTTTCAATGTGCTTCTCCAAAAGTTGATATAAAATTCTT

TGATTAGTCATCTGAAGCATACTTATGCGTTTCCTTCCCCTTTTGTTCTTAGCTGCTTTATTTCGTCATG

TCTCAATTTTTTATTTTTTTTGTGAGAGTCAAGTCTCAAATTTAGTTGCAAAAGAAATCTGTACATTAGA

AGAATAAGCTTTTGCAAATTGGGGACTCTTCAAGACTCTAATGACATGATTTTTTTTTTTTTTA

>MSTRG.176.1 gene=MSTRG.176

CTTTCCTCTTCATTGCATCATTTCCGCAATTAATAGTTCTTCCTGGTTTCGGAGAAACTGGCCAGTGGCC

ACAAGTCACAGCCATATCACCCAGATGTTGCAGAGCTTCAAGTGTTTGAATGATATGCGACATTTTAGGC

CTATCTTTAGGATTCGGACTCACACAACGCAGTGCCAACTTTGCCATTTCTTTTGCTCCTTTAACCGAAT

ATTGCCCACAAAGCCGGGGGTCCATGATGTAACGTAGCCTCCGGCTACTAGCCAAGTATGGTTTTGCCCA

ACTGACCAGGTTTTGCTCTGTTTTCATTCTTTTTTCAGTAGCTCTTCATCCTGTTAGTAGTTCAAGCAGA

ACAACTCCGAAGCTATAGATCACTCCTGGTTGTCAAGTGTCCTGCAAATCGATGGTCAAAATGAAATATC

TGAATACAGATTGTTACCCATTTCTGGGTCGAGAGTGGATCCCGAGAGCTCTGATACCAGGAAAATGATC

AATTCTCCC

>MSTRG.177.1 gene=MSTRG.177

GAAAACATGAAAAATTTGTCCTACTCTATCGCTTTTCAACGGAAACTAAAATTCATCTCATATAACAGAC

TCAAGAACGAAAAATTTCTGGCGAAATAGCTATTATATGTATCAGGAAATCAAAAAGTGAGTGAGAAAGT

ACAGTTATGTAAGCTTCGAGCCGCAGTACTCAGGCATGGAGTGGCATTAGTATAAGCACTGTTGACTCTA

TACACTGAGTGCTCCTTGAACCAGGTTCCAGCATCTTGTTAAAAGAACACTAAGTTCCGACAGACAGGTG

TTATTACAGCTCTGAAGATCTTCTGTTCTTTTCAAATTGATCGTAAACTGACCGAGTTCTTGCAAACAAA

AGAGCACTTGATGCTAGCCCGAAAACAGATACACATCCCCACCACACGAATGTCCAGAAATAGCATTGTC

TTCCCATGCACACCAATGAGTTGGCTGGCAGCATTTGAATGTCCTCAGTTGCATTGGCATCGTAAACGAG

AGCAGATAGAAGACCATATAAAAGCGATCCAATGGGTATATTAGTTATCAGAATGTTATGGTTGACACCC

ACACTGTTAGGCCCGAAAAGCTCAGATGTTATTGACACCGCAGCTGCAAATATGAACCCAGAGCTTAAGC

CAATCAGAGCTGTGCCTACCTGTAATGCCATCTCACTGCCTGTTCCTGCAAGCAAGAAAAAGGCAACTGG

TGTAGGTAAAAGCGCAAGGGCTAGCCATCCGGTCCTGGCAAAATAAAATTTCCTGCAGAATTAAGAATGT

TGTATCAGTAACGTACAAGCACTCGTGTAAACCAAATTGCTAAAGTCTGTACTTACATCCGCAAGATATC

TGGAGCTGCTGACAGCAAGCGGCCAAAGAAGGAGAAAGCGGAATATAGTGTGATAAGAGTCGAGGTTGTT

GAACTCAGGCCAAGTGACTGTGATATCTGTCCTAAATTGTTGCTATACACTAGTCCTATTGTACCTCCAA

AAAAGTAAGCAAAGTAGTAGAGCCAGAAATCAATCCTACGAATAAGCTTGTTTGCCTTGTGATCTTCTCC

AAGCATTACCAAGCGATCTCCTTCTATGATCCTTACAAAGAACCCTTCTCTAGATTTGGTGACTTCTTCA

ATAATACGATTAGGATATGCATCATCACTACCTGTATACGATACTACATTAATAACTGGACTATTCTCCT

GACTCATGAACTCTTTATGAAGTTGAAGATCATCGTGATCAACAAGAATGAAACTTGTGCTATCAACACG

AATGCTGGAGTAAATTGTACGGTGAAACCAATCACGAGCATACACAACACCAGGGATAGCTAGAGGAAGA

ATCAGAAGTATAATGGCACCACAGAACAGAATACGGGCGGATGATGGATCTCCTGGATGGAGCACGAGAA

GATAGAGGCCAGTGATGACAGCAAGAAAATTTAGCAGCAGAAATATTAGGCGGTCACGTTTAACAGCATC

AGTAGGAAGATGGTTCACCGGGGGTTGCCTTAGAATCGGGATTAGAGCTGCTATGGATGTTAAAAGAGGT

ATAAATGCATTGAGAAGAAGGTATAGGTCACTCGATGATGAATCAAATGATTTGGCAGCAAGGTTGTACA

AGGCTGCGCTTACACCATTGAAGCTTACAGTTAGAGATATTGCTAAAGGTCGATTAGCTGGGAAATTCTT

GATGCAGAGAACAAAACACACAGTGTTGAACCAACAGATGCTACATCCAGCTAACAAACACAGCATGAAA

ACCTGAAACAAGTTACCGGAAGAATAAGCTACCAAATGAGGTGAGTCTGTAGAGTCTCGTATACACTACA

ATGCTATAACACCTGAAAATGTCACAAGTACTTATCCGACAAATGTGGGTGAAGGAGGGATCAGTCCAAT

ATACTTCGTAAATTTGGAAAGATTCAGTTAAGGTGAGCTACCATTAAACTTAATCTTACAGCAAATGCCT

CACATTCTGAATAGTTTTAGTAATTCAGCATCATCACAACTTATACGACTCTATTTCCTGCCCAACCCCG

AAAGAAAGGCTAATTTTCC

>MSTRG.180.1 gene=MSTRG.180

AAGTACTTTCAGGAGAATCAGGTGCACTAGGCTCTTTTTTTGGAGTAGTTGTGATCTTAGTTGTTTTGCT

GTCAGTTTGGTGTAGTAGGGAAGTGGTGTTAGGAATGGCCATGGATATTAATGGGAATTCTCGAAGCGTG

GTTTCAAGTCTGGGAAATCTCAAACACCCTGCGGTTGCTCTTCTTTGTTTTTGCTCAAGTGGAGTTGCAC

TTTCTTTGTTTTAAATGTGATTCAGATTTTGTGTAGTTGCTTTTTTCTGGAGCTTGGTTTTAAAAGAGGT

TTTTGGTTTGTAATTATTTGAGGGGTGCCCACTCTGATATTTATGTATTATATGTTTTCCTTCTTTTTAA

AATTATTC

>MSTRG.187.1 gene=MSTRG.187

AGCATATTTTCTTTACTTGTACTGTTGTACATAATATTACCAATATCAACTTGTCTTCACGCAATATTTT

GAGTCCTTCGTTATACTTGGAGAAGTATACTAGCTGCACAAGATGTAGTAAGAAGAGGGTGCAGGAAGCG

AATTGGCGATGGTAAACAGACTGAAATATGGAAGGTCCCTTGGCTTCCATGTAAAGAGATTGGTCTCGTG

ACTACAGAGATGCCCATGCAAATGGAAGGAGTAAGGTTTGTAGTTTCATGCAGGCAAACCAGAAAAAATG

GGATGAGGATGTGCTTATGGACATTGTAATGAAAGAGATAGAAATTGTATCAGAAAAATTCCACCATCAA

TAAGAGAAGGAAGTGACTCATGGTTGGCTTCTACATGATAAGGGGTGCGTTACGGTTAGGAGTTGTTATA

GAATTTTGCAAGGGGAGGTGGAGGCGCCATATGCGTCGTTTTGGGCGAAACTTTGGGGTTTGAAGTTCAG

GGTAAAGTTACTCAGTATGTATGTCGCGTGTGTTCTTGGTGCTTGCCTACTACAGCAAGGCTTACTACAA

AATGAGTGCAAGTTGACACGATTTGCCAATGGTGTAGACGGTATCACGAAACAGATAATCATGTGTTAGT

CGAATGTGAAATGGCAAATGCAACGTGGATGGAAGTTGGTCCTCAGGCGTGCATTAGTACACTGCCAGGT

GATAATGTGTTTGATGTTATATGTCGATGTTTTAACAGCTGCACCAGATAACAACATACCTTGATAGTTT

TGATGTGTTGGAGTATTTGGACCAGAAGAAACAAGTGGGAGGGAAAAAATTAATATGTCAGAGTTTGGCA

TCAAAGCAGCTGCCCTGAACCTGCTTGCAGACTGGAAGAAGGCACAGGAACAACAGTTAACACCTGGTAC

AAGGTCGAGAACACTGGGTGCAGTATGTAAATGGGAGAAGCCTCAGACTTCTTGGGTGAAAATGAATTTA

GATGCTGCATTTTTTGAAGAGATTGATTGTATTGGTTTGGGCAGTGTGGTTCGAGGTGCAGACGGACAAT

TTATCATGGCTATGCATAGGAGACAACAAGGGCTGATGGCACCAAGAGAGGCTGAGGCATTATGCTTGAA

AGAAGCACTGATCTGGCTTAAGGACAAGAGTTTCAGCAAGTGTAATTTCGAAACTGATTCACAATTTCTC

GCTTGAGCTTGCAAAGGAGCTGGTGGAAGGTCATATTTTGATACAGTTATTGGAGATTGTATTGATTTAT

ATAATAACTTTGATAAAGTGTCATTATGTTTTACACATAGGTCTGCAAATGGAGTGGCTCATGTTTTAGC

AAGGATGGCTTATTCTATGTCAGGCTCTCGGGAATGGCATATTAATGCTCCCGATTTTATTCATCATGTA

ATTTGTTCCGAAGCTTTATAAGAAATGCAAGTACGAT

>MSTRG.188.1 gene=MSTRG.188

TGGAGGCGCCATATGCGTCGTTTTGGGCGAAACTTTGGGGTTTGAAGTTCAGGGTAAAGTTACTCAGTAT

GTATGTCGCGTGTGTTCTTGGTGCTTGCCTACTACAGCAAGGCTTACTACAAAATGAGTGCAAGTTGACA

CGATTTGCCAATGGTGTAGACGGTATCACGAAACAGATAATCATGTGTTAGTCGAATGTGAAATGGCAAA

TGCAACGTGGATGGAAGTTGGTCCTCAGGCGTGCATTAGTACACTGCCAGGTGATAATGTGTTTGATGTT

ATATGTCGATGTTTTAACAGCTGCACCAGATAACAACATACCTTGATAGTTTTGATGTGTTGGAGTATTT

GGACCAGAAGAAACAAGTGGGAGGGAAAAAATTAATATGTCAGAGTTTGGCATCAAAGCAGCTGCCCTGA

ACCTGCTTGCAGACTGGAAGAAGGCACAGGAACAACAGTTAACACCTGGTACAAGGTCGAGAACACTGGG

TGCAGTATGTAAATGGGAGAAGCCTCAGGCATTATGCTTGAAAGAAGCACTGATCTGGCTTAAGGACAAG

AGTTTCAGCAAGTGTAATTTCGAAACTGATTCACAATTTCTCGCTTGAGCTTGCAAAGGAGCTGGTGGAA

GGTCATATTTTGATACAGTTATTGGAGATTGTATTGATTTATATAATAACTTTGATAAAGTGTCATTATG

TTTTACACATAGGTCTGCAAATGGAGTGGCTCATGTTTTAGCAAGGATGGCTTATTCTATGTCAGGCTCT

CGGGAATGGCATATTAATGCTCCCGATTTTATTCATCATGTAATTTGTTCC

>MSTRG.189.1 gene=MSTRG.189

ATTTAACTCAAATCAAAATATTGAAGAACATAACTTGTTCAATTCAGCTAGAATATTCTCACTTAGACAT

TTAATCAAACACCTTGATATCAATTTTCATGTATAAGCAGATAATAAACACGCAGCACCAGCAGATCCAT

AAATTGCATAAATGGTGAGCCAATGCACACAAATATATGCATAAATTTGTGACGAGAGTATAGATATGAG

GAGAGTTTTGTGCAAAGAGTTACCTTGGAAAGAGCCTTCACTATGGAGCAATATGATGCAGAGATGGAGA

GTGGAGAGAGATATTGGGAGAGAGGGAGAGATGGAGAGGAGAGACTGTTAGAGAGAGAGGCGAGATGACG

TGTGAGAGAGAGAGGCGAAATAGACGCACAGGCAGAGAGAATTGAAGAAACGAGGGAGAAGATGT

>MSTRG.190.1 gene=MSTRG.190

CGGGATTGGGCTTCCCATTCCATAGCCTTACGTTTGGATTAAATTTTTAAGACAAGGGAATGGGAACTCT

TGTGCCACTAGCCTGTTAACACAAACCTGACCAATACCCCGGGTTACCAATATTGATTCCCGGTCTCGAC

ATAACATCTTCTCCCTCGTTTCTTCAATTCTCTCTGCCTGTGCGTCTATTTCGCCTCTCTCTCTCACACG

TCATCTCGCCTCTCTCTCTAACAGTCTCTCCTCTCCATCTCTCCCTCTCTCCCAATATCTCTCTCCACTC

TCCATCTCTGCATCATATTGCTCCATAGTGAAGGCTCTTTCCAAGGTAACTCTTTGCACAAAACTCTCCT

CATATCTATACTCTCGT

>MSTRG.194.1 gene=MSTRG.194

TTGATCAAACTTCTTATCTACACATTGTAGTAGATAACCATACCAGTTATATTTAAAGCACTGATTCAAA

TTTCCGGAATACGACAGAATCTTCAAACACACATAAGGTGTTCTGGACCCCTCTATTAGAATATTAGTGA

GGACAATGAGAAAGTTCAATTTAAATCTCAAGTCAACTAATTTCGATGATTAAATAACATCACATAAACT

ATTTGCAGTAATCTTATGAGGACCCTTGTACTCGTTGAACTGTGATCTCCAAACATCTGTATACTCACTG

TTGTAAGATCCTCAAACATTAACTCCCCCTTTCGGCAAGCCAAGTATAATCTGGACATCATTTTCCGAAA

AGTTTATAATATTCTCACCAATTGAAATTCTTGAATCGATTGATTTGTATGACTTCGCAATCAAAAAAGT

CAAAGGTTGAGGGTATTCATTGATGTTGAAAACTAGAACCTTTTCAAAACCAGTCTCTACAACCTATTGC

CTTTGTACATCCGAAAGATTGTCCACGACTCTCTTCAAGAACATAGGTTTTGCTCGAACCAACAAACGGC

CGGTATTCTTATCGAGATTCTTCTCAGCCATCATATCACTGAAAAAGGAATATGTGGTTGCGGAGATCAT

ACAAAAGTCATAACAGATATATTG

>MSTRG.197.5 gene=MSTRG.197

CAAACCAAGTGCCCTTTAACATATAAGCCGTCCCCGATAATTAATTTTATTCTTCCTAAATCTTCGCGTA

CTCAACAAACCAAGTGCATCGCCGCTTTCCAGGTGCCCATATCAGTCATGTAAGAATTGTTGCGCAAAAT

CTCAAAACCCGTGCCATGTACATGGAATTCTCATAGGGTACCCAAACCAGTGAGCAAGAAGAATATGTTA

GAAGTCCAGAAAAGGAGTTCAGGAGTTCAGGAGAGATGAGGCATTGGCTGCGTTTATTGACAAAATTAAC

AAAGCCCAGAATGAGAAAGATCTGAAACTCTGCATGGAGATGAAAGATCAGCTTGTTAATCCGCATACTG

AGAGTACTCAAGCAGAATCCGAAAGTGTTGCAATTCGTATGGCGCTCCAGACTGATGATGTGGTCAAACC

TCTGTTCGCTTAATTTTTTTTATTTTTTATTTTGAAAGGTACCCCAAAACTTGTTTGTTGTTTTCGGAAG

GTACCCCAAAACTTTTGATCCTCCAAATTATAGACAAATTAGTGCATACTCTGATTCCCTGGAGGAGCTG

TAGGAGCTGTAGTCTATGATGAGTTCTTGGTACCAAAAACTTGAATGACTCAGTCGGCGAATTGAATAAG

AATCAAACCATTAATATGCATCCGTCCGTTTAGAACGGTTGCATACTAATGGTTTGCTTGCTAGTCGATT

TGCCAAATGAAACATTCAAGTTTTTTGGTACCAAGAACTCATCATAGTCTACAACTCCTTAATATGCTCC

AGGAAATATAGATCATGGTTTATAAGTAAATAATCATTTATAGTTTTAGTGGAGGATCAACGAACACTTG

ATCACGTCATCTGTCAGAGATTGACCACATCATCAGTTTGGAGGCCCATAAAAATTGCATCACTTTCGGG

TTCTGCATGAGGACTCTCGGTATGCTGATTAAAAAGTTGATCTTTCATCTCCATACAGACTTTCGGATCT

TCCTCATTCTGGGCTTTGTTGATTTTGTCAATACAAGTTATCCACTCAATTAATTATATATCACATATTT

ATATTTTAATTCAGGATTTTTAATATATTTTGGGTTTTTCTTGAAAAATATACTCGCTCTGTTCTAGCTA

>MSTRG.197.6 gene=MSTRG.197

CCAAGTGCCCTTTAACATATAAGCCGTCCCCGATAATTAATTTTATTCTTCCTAAATCTTCGCGTACTCA

ACAAACCAAGTGCATCGCCGCTTTCCAGGTGCCCATATCAGTCATGTAAGAATTGTTGCGCAAAATCTCA

AAACCCGTGCCATGTACATGAACAACATCTCTGCGCTACTTGCTGAGTATATATCTAAATATATCTACGT

CCCACAAGACCCTTCAATCTCACAAGTAACGTTGCCAAGAATCATAACATCACAAACTTAGACATTCAAC

AATTGACACTGTGGAATTCTCATAGGGTACCCAAACCAGTGAGCAAGAAGAATATGTTAGAAGTCCAGAA

AAGGAGTTCAGGAGTTCAGGAGAGATGAGGCATTGGCTGCGTTTATTGACAAAATTAACAAAGCCCAGAA

TGAGAAAGATCTGAAACTCTGCATGGAGATGAAAGATCAGCTTGTTAATCCGCATACTGAGAGTACTCAA

GCAGAATCCGAAAGTGTTGCAATTCGTATGGCGCTCCAGACTGATGATGTGGTCAAACCTCTGTTCGCTT

AATTTTTTTTATTTTTTATTTTGAAAGGTACCCCAAAACTTGTTTGTTGTTTTCGGAAGGTACCCCAAAA

CTTTTGATCCTCCAAATTATAGACAAATTAGTGCATACTCTGATTCCCTGGAGGAGCTGTAGGAGCTGTA

GTCTATGATGAGTTCTTGGTACCAAAAACTTGAATGACTCAGTCGGCGAATTGAATAAGAATCAAACCAT

TAATATGCATCCGTCCGTTTAGAACGGTTGCATACTAATGGTTTGCTTGCTAGTCGATTTGCCAAATGAA

ACATTCAAGTTTTTTGGTACCAAGAACTCATCATAGTCTACAACTCCTTAATATGCTCCAGGAAATATAG

ATCATGGTTTATAAGTAAATAATCATTTATAGTTTTAGTGGAGGATCAACGAACACTTGATCACGTCATC

TGTCAGAGATTGACCACATCATCAGTTTGGAGGCCCATAAAAATTGCATCACTTTCGGGTTCTGCATGAG

GACTCTCGGTATGCTGATTAAAAAGTTGATCTTTCATCTCCATACAGACTTTCGGATCTTCCTCATTCTG

GGCTTTGTTGATTTTGTCAATACAAGTTATCCACTCAATTAATTATATATCACATATTTATATTTTAATT

CAGGATTTTTAATATATTTTGGGTTTTTCTTGAAAAA

>MSTRG.198.1 gene=MSTRG.198

TTCTGATTTCTCTGTTAAACTAAAATAGCCAGATTTGAACCATTTCATCCTCACCTACGATTTGAATCAC

AAAATCGATTAAAAACTCGAGGATTCCTAATTCTTTTTCAGAAATCCCATGGCTGCGTGTTTGTTTTGTG

TGTTTTTTTTCTCAGTCACCTACGATTTGAATCACAAAATCGATTAAAAACTCGAGGATTCCTAATCCTT

TTTCAGAAATCCCATGGCTGCGTGTTTGTTCTGTGTGTTTTTTTTCTCAGGGATATATAAAAACTAAAGA

AAGCTACCGACGAGACTCGGCTAATGACTCCACGATCTCGTACAAGAGAGACATGATAGCGGTGGTAGCC

GAAGAGGATGGTAGTGACGGCGGCAGTTCCGAGATCAGAATGAGATTCGGGTGTCGGGTTCAGAAAAGAT

GCATTAATTGTGCCTTAATTGCGGCGTTAATTACGGCATTAATGTTTTGTTAATTGTGCCTTAATTG

>MSTRG.201.1 gene=MSTRG.201

ATTGCAGTCACAGTCTTGCTACTTGTGCCCGTGGATCGATACATACACGAACACGATACATACATCTCCT

ATTCTTACCGGTACCAACGCTCACTCTCCCCGACCTCCTCTCCTTGTGTCTATACATACATCGTGTATAT

GTATGTATGTATAGATTAAAAGCTTAATTGATAAGCTCTGATCTCAATACAGAAATGAGTAGCAGGAGAG

GAGGTGCGGAGATGTTGAAAGATAGCAGGACCAAACAGATTAATGATGCTCTTGATAAGCATTTGGAGCG

ATCTTCGCCTTCTTCGTCTCGAGGCGGAGGAGGAATAGCTTTTAAGGAGAAGGATCGGGTTTCCGGGTTG

TCCGGGAACGGAAAGGCTTCGTTTCAGAAGAAGCCTATTGAGGAATCTGAAACCGACAGCGAGGAATCAG

ATGTTAGCGGTTCTGATGGGGTAGACTCATCATGGATTTCTTGGTTTTGCAATTTGCGGGGAAATGAATT

CTTTTGTGAAGTTGATGAGGATTACATTCAAGATGATTTTAATTTATGTGGATTAAGCAGCCAAGTTCCA

TACTATGATCATGCTCTTGATCTGATTCTAGATCTCGAGTCCTCTCCTGGTGATGTATTTACAGAGGAAC

AGCATGATTTAGTTGAAACAGCAGCAGAGATGCTATATGGTCTGATACATGTTCGATATGTATTGACAAC

TAAAGGGCAGGCTGCTATGTTGGAGAAGTACAAAAATGCTGAGTTCGGTCGATGCCCTAGAGTTTGCTGC

TCTGGACAACCCTGCTTAGCTGTTGGTCAATCAGATATTCCTCGTCAATCAACTGTGAAAATTTATTGTC

CCAAGTGCGAAGATATATATGCCCCTCGATCCAGGCATCAAGATAACCTTGACGGAGCTTATTTTGGAAC

GACATTTCCTCACCTGTTTTTGATGACCTACGGGCATCTTAAACCACAGAAGAAATTGACGAGTTATATT

CCAAGAGTATTTGGTTTCAAGGTTCACAAACCATGATGCTGTAGTTATGTTACTCAGTTACCTGGGATTA

AATCTTGTGCTGCTGAACGCAAACCCCAATACCATCACTCAGACTCCTCGGATAACAGATGATAGACTAG

AACAGTTATCTACTATGAAAGTCTTTGGTGAGAGCAATTGTATTCAAATGTATGCTAAATTTTACTTATA

AATTTAGGTTCGATAATGAATTTGTTGGGATGTATAATAACTTTGTTGTAGATTAGCTGCAGAGAGAATG

GTCTTCTTCTCAAGTATGTAGTGGATCTTATTGAATGGAATTATGTCCTGAATTTTTTGGTTTAAAA

>MSTRG.204.1 gene=MSTRG.204

CTTAAATTGTTGTTTAACTTGGGATTATTCTGATTGGGTTTGATTCTTTTTTCTGGAATAGCGAAAGTGA

TATTGATGATGAAGCAGATTGCGGAGAGTCCAGTTTGATGCACTTCCCAGTGCATGATCAAGCATCACTG

CACGAAACGATGCAACAATGTATCATCAAGGCAGCAAATTCCCTCCTGCATGACTCTGCCAATGCGGAAC

CTTTACCTTATATCAACCATTGGACTCATAAACACCCGCTGACACTTAGAAACAAGAACGCAACAACTTC

AACCTCTAACTTGAAACATAAATTAGCAGAAACTGAATTGCTAATCTGTGACGGGTGCACTAAACCTATA

TCTTTGGTTGATGATATATCCTACGAATGCAATTTATGCAAATTTTTTCTCCACAAATCATGCGCCCTAT

TTCCAGAAGAGATTGAGCATCATCTAGCAGGCAAGCTGTGGGGAGTACAAGTAGATAACGAACAAATAAT

TTATTGCCAGGGTTGCAGTCATCTTGGTAATGGGATTTTCATGCGCAATGAAACAGCCTGTTTTGATATC

GGGTGTGCTTCGTTACCCAGAATAATCAAACATGAATCTCATCGTCATCCTCTTAAGCAATTACCATATC

CAGATGATTATATTTGCAAAGCATGTCGTTCTGAACTTTTAACAGACAGAGAAATTATGATGTATGGATG

TGAAAGATGCGAATTCTACATACATATATGGTGTGCGTTAAGGCCACGTCGGGTGAATCATCGATGGGAT

CCTCATACTCTGGACCTGATTCTGTCGCTCAACAATGTACCTGATCATCCTCATGAATTCGAGTGTGAAC

TCTGTTCCGAGCAGATAGACCCAAACACTTGGTTCTATCACTGCAATGTTTGTGATCTATCATTTGATAC

TTTCTGTATTGATCCTGATTCCTGGCTCTCCAATATCAAGCTTGGTGCCACCAACATTCATACCGACTCA

CATCCTCACTCACACGGCCTGACATTGGCCGTGAATAAAAAGAAGAGGAAATGCGACAAGTGTGGTATAG

ATGCACCCGGCTGGATAGTCCTCGAATGTTCAGAATGCGAATTTATGGTTCATGTGCGTTGTTGATGAAA

TCAGACTTCAAATCAGATGCTGCACTTGTGTCAAATGTAATGAAGAATGAAAGTGTCTTATTGCAAAGCC

TGATGGAGCATTGCACTAGAATAAAAGGCCTTTGGCTATATTTTTTTCTTTGGAATGCAAAAATTATCAA

CTATGTTCTTGCTCCTCAAAACAATGGTCTTAATTACCATATTTGTGTTGAAAACATATTGTACT

>MSTRG.205.1 gene=MSTRG.205

CTTAAATTGTTGTTTAACTTGGGATTATTCTGATTGGGTTTGATTCTTTTTTCTGGAATAGCGAAAGTGA

TATTGATGATGAAGCAGATTGCGGAGAGTCCAGTTTGATGCACTTCCCAGTGCATGATCAAGCATCACTG

CACGAAACGATGCAACAATGTATCATCAAGGCAGCAAATTCCCTCCTGCATGACTCTGCCAATGCGGAAC

CTTTACCTTATATCAACCATTGGACTCATAAACACCCGCTGACACTTAGAAACAAGAACGCAACAACTTC

AACCTCTAACTTGAAACATAAATTAGCAGAAACTGAATTGCTAATCTGTGACGGGTGCACTAAACCTATA

TCTTTGGTTGATGATATATCCTACGAATGCAATTTATGCAAATTTTTTCTCCACAAATCATGCGCCCTAT

TTCCAGAAGAGATTGAGCATCATCTAGCAGGCAAGCTGTGGGGAGTACAAGTAGATAACGAACAAATAAT

TTATTGCCAGGGTTGCAGTCATCTTGGTAATGGGATTTTCATGCGCAATGAAACAGCCTGTTTTGATATC

GGGTGTGCTTCGTTACCCAGAATAATCAAACATGAATCTCATCGTCATCCTCTTAAGCAATTACCATATC

CAGATGATTATATTTGCAAAGCATGTCGTTCTGAACTTTTAACAGACAGAGAAATTATGATGTATGGATG

TGAAAGATGCGAATTCTACATACATATATGGTGTGCGTTAAGGCCACGTCGGGTGAATCATCGATGGGAT

CCTCATACTCTGGACCTGATTCTGTCGCTCAACAATGTACCTGATCATCCTCATGAATTCGAGTGTGAAC

TCTGTTCCGAGCAGATAGACCCAAACACTTGGTTCTATCACTGCAATGTTTGTGATCTATCATTTGATAC

TTTCTGTATTGATCCTGATTCCTGGCTCTCCAATATCAAGCTTGGTGCCACCAACATTCATACCGACTCA

CATCCTCACTCACACGGCCTGACATTGGCCGTGAATAAAAAGAAGAGGAAATGCGACAAGTGTGGTATAG

ATGCACCCGGCTGGATAGTCCTCGAATGTTCAGAATGCGAATTTATGGTTCATGTGCGTTGTTGATGAAA

TCAGACTTCAAATCAGATGCTGCACTTGTGTCAAATGTAATGAAGAATGAAAGTGTCTTATTGCAAAGCC

TGATGGAGCATTGCACTAGAATAAAAGGCCTTTGGCTATATTTTTTTCTTTGGAATGCAAAAATTATCAA

CTATGTTCTTGCTCCTCAAAACAATGGTCTTAATTACCATATTTGTGTTGAAAACATATTGTACT

>MSTRG.206.1 gene=MSTRG.206

TAATATATATATGAACAGAGTGCAGTGGTCGTGTTGAATCATTCATACTCGCCTTCAGTAAATATACATC

TCCCTTTGTCTCTCTCTCTCTCTCTCTGGTAAACATAGATGGCAGAATTGAAATTTTTTTTTCATGAGCA

TCAGCTGATACTAAATGAAGCTGAGCCTGTTGTCGGTAAGGATGTAGAATGTGTTGGGTGCAAACAGCCA

ATCAACAAACTCATAGACGCATTTTATAGGTGCAACAAGTCCCTTATTGATAGCAGTCCAGCGAGTGATT

GTGTTGGTTTCTATATGCATAAAACTTGTTCTGAGTTGCCCTCAACTTTTACACACCCTATGAACCCGAA

AAAACCTCTAAGCCTCTTTGTGCTTCCATATAAAAAAGAATACTTTTATATTTGTCATGCTTGTGACAGC

CACTCTCAGCACTTTATGTATGGTTCTGATTCTTCTGTCAGTTCCGAATTTCGTGTTTGTTTGAAATGTG

TAATGTCAGAACTTAAATCTCTAGAGGACCGTAACCTTTGTCATCCAGGTCACAATCACCCATTAACTTT

AGTCCAAAGCCCAGCTTTGTTCCTGTGTCATGCTTGTAACACTACAGCTACAGACTTGTCCTATATCTGT

ACAAGATGTTGTTTTTATATACACAAGAATTGTGCCAATGCACCCACCACCTATCAAAGTAAATTCCACA

ATGAACACGCTCTCATCTTGACCTACTCTCTTCCCCAACAATATCGTGAATATCCCTGTTACTGCAGCAT

CTGCGAGAAACATATAAATCCCATCTACTGGGTTTATATTTGTGCGAATTGCAGATTTTTTGCTCACGTG

AAATGTGCTTCATTAACTGAGTTGTTGAGTGAAAGTGATATTGATGATGAAGCAGATTGCGGAGAGTCTA

ATTTGATGCAGTTCCCAGTGCATGATGAAGCATCACTACATGAAACAATGCAAGAATGTATCATCAAGGC

AGCAAATTCTTCCACTGGGGAACCTTCACCTTATATCAATCATTGGGCCCATGGACACCAACTGGCACTC

GGAAACAAGAATGCAAAAACTTTGCCCCTCAACTTGAAGCCGAAAATAGCAGAAACTGAATTGCTAATCT

GTGATGGTTGCATGAATCCTATCTCCTTGGTTGATGTATTCTATGAATGCAGTTTATGCAATTTTTTTCT

CCACAGATCATGCTCCCAATTCCCCGAAAGGATTAAGCATCATCTAGCAGGCGATCTGGAGGGAATTCTA

GCACGAATGTCAGGTGAACTATATACTCTTGAATGCAGAGGTTGCGGCATTCGTGGTAATGGGATTTGCA

TGCACAATGATGAATACGCTTTTGACATCAAGTGCGCTTCATTACCCAGAATAATCAAACATAAAGGTCA

TCGTCACCCTCTTCAGCAATTAAAGACTCCAGATGACTTTCTTTGCAAAGCATGTTGGCGTTACCGTGTA

ACTGAAGAGGTAAGAACCATCGTGTATGGATGTGAAAAATGTGATTTTTTCACACATATAGGGTGTGTGT

TAAGTGCACAAGTGGTGAAACATAGATGGGATCCTCATCCTCTCTACTTGATCCTGTCTCTTAAGAATGT

ACCTGATCATCCCCATGATTTCCATTGTGAATTTTGCCCCGATCAGATAAACACCAACAGTTGGTTTTAT

CACTGCAATGTATGCGATCTATCATTTCATACCAGCTGTATTGATCCAGATGATTGGGTGTCCAATATCA

AGTTTGGTGCTACCGACATTTACAGCGACAAACATCCACATCCACACGGCCTCACATATATTCTCAATAA

AAAGACGAGGAATTGCAATTTATGTGGTAAAGATGCACGCGGTATGCGGGCTCTCCAATGTTCAACATGC

AAATATATAGTGCATGAGAATTGTTTCATTGAAGATTGAAATGAGGCAAAAATCAGATATTGTATTACTG

GATTATTCAGGTTCAGAGGATGTACAGAAAGAAGTAGCCACCGCATTCTTGAAGAATCTCTAGAACTCGT

TGAGCATTAGTAACAGTTCGCTCTATAGCATTAAGAGTTCTAATATTTCAAT

>MSTRG.206.2 gene=MSTRG.206

TAATATATATATGAACAGAGTGCAGTGGTCGTGTTGAATCATTCATACTCGCCTTCAGTAAATATACATC

TCCCTTTGTCTCTCTCTCTCTCTCTCTGGTAAACATAGATGGCAGAATTGAAATTTTTTTTTCATGAGCA

TCAGCTGATACTAAATGAAGCTGAGCCTGTTGTCGGTAAGGATGTAGAATGTGTTGGGTGCAAACAGCCA

ATCAACAAACTCATAGACGCATTTTATAGGTGCAACAAGTCCCTTATTGATAGCAGTCCAGCGAGTGATT

GTGTTGGTTTCTATATGCATAAAACTTGTTCTGAGTTGCCCTCAACTTTTACACACCCTATGAACCCGAA

AAAACCTCTAAGCCTCTTTGTGCTTCCATATAAAAAAGAATACTTTTATATTTGTCATGCTTGTGACAGC

CACTCTCAGCACTTTATGTATGGTTCTGATTCTTCTGTCAGTTCCGAATTTCGTGTTTGTTTGAAATGTG

TAATGTCAGAACTTAAATCTCTAGAGGACCGTAACCTTTGTCATCCAGGTCACAATCACCCATTAACTTT

AGTCCAAAGCCCAGCTTTGTTCCTGTGTCATGCTTGTAACACTACAGCTACAGACTTGTCCTATATCTGT

ACAAGATGTTGTTTTTATATACACAAGAATTGTGCCAATGCACCCACCACCTATCAAAGTAAATTCCACA

ATGAACACGCTCTCATCTTGACCTACTCTCTTCCCCAACAATATCGTCAATATCTTTGTTACTGCAGCAT

CTGCGAGAAACATATAAATCCCATCTACTGGGTTTATATTTGTGCGAATTGCAGATTTTTTGCTCACGTG

AAATGTGCTTCATTAACTGAGATGTTGAGTGAAAGTGATATTGATGATGAAGCAGATTGCGGAGAGTCTA

ATTTGATGCAGTTCCCAGTGCATGATGAAGCATCACTACATGAAACAATGCAAGAATGTATCATCAAGGC

AGCAAATTCTTCCACTGGGGAACCTTCACCTTATATCAATCATTGGGCCCATGGACACCAACTGGCACTC

GGAAACAAGAATGCAAAAACTTTGCCCCTCAACTTGAAGCCGAAAATAGCAGAAACTGAATTGCTAATCT

GTGATGGTTGCATGAATCCTATCTCCTTGGTTGATGTATTCTATGAATGCAGTTTATGCAATTTTTTTCT

CCACAGATCATGCTCCCAATTCCCCGAAAGGATTAAGCATCATCTAGCAGGCGATCTGGAGGGAATTCTA

GCACGAATGTCAGGTGAACTATATACTCTTGAATGCAGAGGTTGCGGCATTCGTGGTAATGGGATTTGCA

TGCACAATGATGAATACGCTTTTGACATCAAGTGCGCTTCATTACCCAGAATAATCAAACATAAAGGTCA

TCGTCACCCTCTTCAGCAATTAAAGACTCCAGATGACTTTCTTTGCAAAGCATGTTGGCGTTACCGTGTA

ACTGAAGAGGTAAGAACCATCGTGTATGGATGTGAAAAATGTGATTTTTTCACACATATAGGGTGTGTGT

TAAGTGCACAAGTGGTGAAACATAGATGGGATCCTCATCCTCTCTACTTGATCCTGTCTCTTAAGAATGT

ACCTGATCATCCCCATGATTTCCATTGTGAATTTTGCCCCGATCAGATAAACACCAACAGTTGGTTTTAT

CACTGCAATGTATGCGATCTATCATTTCATACCAGCTGTATTGATCCAGATGATTGGGTGTCCAATATCA

AGTTTGGTGCTACCGACATTTACAGCGACAAACATCCACATCCACACGGCCTCACATATATTCTCAATAA

AAAGACGAGGAATTGCAATTTATGTGGTAAAGATGCACGCGGTATGCGGGCTCTCCAATGTTCAACATGC

AAATATATAGTGCATGAGAATTGTTTCATTGAAGATTGAAATGAGGCAAAAATCAGATATTGTATTACTG

GATTATTCAGGTTCAGAGGATGTACAGAAAGAAGTAGCCACCGCATTCTTGAAGAATCTCTAGAACTCGT

TGAGCATTAGTAACAGTTCGCTCTATAGCATTAAGAGTTCTAATATTTCAAT

>MSTRG.206.4 gene=MSTRG.206

AGAATTATAATTTTTTTCATAGTCCGAGACAGAGAATTATAAATTTTTAATAGTCCGAAACTTCACACTC

ATACTGGTCTTTATATGTTTCTGGGATGAAATGGTATCACAAAGAATATAAATGTATGAATGATATTGAT

GATGAAGCAGATTGCGGAGAGTCTAATTTGATGCAGTTCCCAGTGCATGATGAAGCATCACTACATGAAA

CAATGCAAGAATGTATCATCAAGGCAGCAAATTCTTCCACTGGGGAACCTTCACCTTATATCAATCATTG

GGCCCATGGACACCAACTGGCACTCGGAAACAAGAATGCAAAAACTTTGCCCCTCAACTTGAAGCCGAAA

ATAGCAGAAACTGAATTGCTAATCTGTGATGGTTGCATGAATCCTATCTCCTTGGTTGATGTATTCTATG

AATGCAGTTTATGCAATTTTTTTCTCCACAGATCATGCTCCCAATTCCCCGAAAGGATTAAGCATCATCT

AGCAGGCGATCTGGAGGGAATTCTAGCACGAATGTCAGGTGAACTATATACTCTTGAATGCAGAGGTTGC

GGCATTCGTGGTAATGGGATTTGCATGCACAATGATGAATACGCTTTTGACATCAAGTGCGCTTCATTAC

CCAGAATAATCAAACATAAAGGTCATCGTCACCCTCTTCAGCAATTAAAGACTCCAGATGACTTTCTTTG

CAAAGCATGTTGGCGTTACCGTGTAACTGAAGAGGTAAGAACCATCGTGTATGGATGTGAAAAATGTGAT

TTTTTCACACATATAGGGTGTGTGTTAAGTGCACAAGTGGTGAAACATAGATGGGATCCTCATCCTCTCT

ACTTGATCCTGTCTCTTAAGAATGTACCTGATCATCCCCATGATTTCCATTGTGAATTTTGCCCCGATCA

GATAAACACCAACAGTTGGTTTTATCACTGCAATGTATGCGATCTATCATTTCATACCAGCTGTATTGAT

CCAGATGATTGGGTGTCCAATATCAAGTTTGGTGCTACCGACATTTACAGCGACAAACATCCACATCCAC

ACGGCCTCACATATATTCTCAATAAAAAGACGAGGAATTGCAATTTATGTGGTAAAGATGCACGCGGTAT

GCGGGCTCTCCAATGTTCAACATGCAAATATATAGTGCATGAGAATTGTTTCATTGAAGATTGAAATGAG

GCAAAAATCAGATATTGTATTACTGGATTATTCAGGTTCAGAGGATGTACAGAAAGAAGTAGCCACCGCA

TTCTTGAAGAATCTCTAGAACTCGTTGAGC

>MSTRG.206.3 gene=MSTRG.206

AAACATAGATGGCAGAATTGAAATTTTTTTTTCATGAGCATCAGCTGATACTAAATGAAGCTGAGCCTGT

TGTCGGTAAGGATGTAGAATGTGTTGGGTGCAAACAGCCAATCAACAAACTCATAGACGCATTTTATAGG

TGCAACAAGTCCCTTATTGATAGCAGTCCAGCGAGTGATTGTGTTGGTTTCTATATGCATAAAACTTGTT

CTGAGTTGCCCTCAACTTTTACACACCCTATGAACCCGAAAAAACCTCTAAGCCTCTTTGTGCTTCCATA

TAAAAAAGAATACTTTTATATTTGTCATGCTTGTGACAGCCACTCTCAGCACTTTATGTATGGTTCTGAT

TCTTCTGTCAGTTCCGAATTTCGTGTTTGTTTGAAATGTGTAATGTCAGAACTTAAATCTCTAGAGGACC

GTAACCTTTGTCATCCAGGTCACAATCACCCATTAACTTTAGTCCAAAGCCCAGCTTTGTTCCTGTGTCA

TGCTTGTAACACTACAGCTACAGACTTGTCCTATATCTGTACAAGATGTTGTTTTTATATACACAAGAAT

TGTGCCAATGCACCCACCACCTATCAAAGTAAATTCCACAATGAACACGCTCTCATCTTGACCTACTCTC

TTCCCCAACAATATCGTCAATATCTTTGTTACTGCAGCATCTGCGAGAAACATATAAATCCCATCTACTG

GGTTTATATTTGTGCGAATTGCAGATTTTTTGCTCACGTGAAATGTGCTTCATTAACTGAGATGTTGAGT

GAAAGTGATATTGATGATGAAGCAGATTGCGGAGAGTCCAGTTTGATGCAGTTCCCAGTGCATGATGAAG

CATCACTACATGAAACAATGCAACAATTGAAAGTGATATTGATGATGAAGCAGATTGCGGAGAGTCTAAT

TTGATGCAGTTCCCAGTGCATGATGAAGCATCACTACATGAAACAATGCAAGAATGTATCATCAAGGCAG

CAAATTCTTCCACTGGGGAACCTTCACCTTATATCAATCATTGGGCCCATGGACACCAACTGGCACTCGG

AAACAAGAATGCAAAAACTTTGCCCCTCAACTTGAAGCCGAAAATAGCAGAAACTGAATTGCTAATCTGT

GATGGTTGCATGAATCCTATCTCCTTGGTTGATGTATTCTATGAATGCAGTTTATGCAATTTTTTTCTCC

ACAGATCATGCTCCCAATTCCCCGAAAGGATTAAGCATCATCTAGCAGGCGATCTGGAGGGAATTCTAGC

ACGAATGTCAGGTGAACTATATACTCTTGAATGCAGAGGTTGCGGCATTCGTGGTAATGGGATTTGCATG

CACAATGATGAATACGCTTTTGACATCAAGTGCGCTTCATTACCCAGAATAATCAAACATAAAGGTCATC

GTCACCCTCTTCAGCAATTAAAGACTCCAGATGACTTTCTTTGCAAAGCATGTTGGCGTTACCGTGTAAC

TGAAGAGGTAAGAACCATCGTGTATGGATGTGAAAAATGTGATTTTTTCACACATATAGGGTGTGTGTTA

AGTGCACAAGTGGTGAAACATAGATGGGATCCTCATCCTCTCTACTTGATCCTGTCTCTTAAGAATGTAC

CTGATCATCCCCATGATTTCCATTGTGAATTTTGCCCCGATCAGATAAACACCAACAGTTGGTTTTATCA

CTGCAATGTATGCGATCTATCATTTCATACCAGCTGTATTGATCCAGATGATTGGGTGTCCAATATCAAG

TTTGGTGCTACCGACATTTACAGCGACAAACATCCACATCCACACGGCCTCACATATATTCTCAATAAAA

AGACGAGGAATTGCAATTTATGTGGTAAAGATGCACGCGGTATGCGGGCTCTCCAATGTTCAACATGCAA

ATATATAGTGCATGAGAATTGTTTCATTGAAGATTGAAATGAGGCAAAAATCAGATATTGTATTACTGGA

TTATTCAGGTTCAGAGGATGTACAGAAAGAAGTAGCCACCGCATTCTTGAAGAATCTCTAGAACTCGTTG

AGC

>MSTRG.206.7 gene=MSTRG.206

CTCTCTCTCTCTCTCTGGTAAACATAGATGGCAGAATTGAAATTTTTTTTTCATGAGCATCAGCTGATAC

TAAATGAAGCTGAGCCTGTTGTCGGTAAGGATGTAGAATGTGTTGGGTGCAAACAGCCAATCAACAAACT

CATAGACGCATTTTATAGGTGCAACAAGTCCCTTATTGATAGCAGTCCAGCGAGTGATTGTGTTGGTTTC

TATATGCATAAAACTTGTTCTGAGTTGCCCTCAACTTTTACACACCCTATGAACCCGAAAAAACCTCTAA

GCCTCTTTGTGCTTCCATATAAAAAAGAATACTTTTATATTTGTCATGCTTGTGACAGCCACTCTCAGCA

CTTTATGTATGGTTCTGATTCTTCTGTCAGTTCCGAATTTCGTGTTTGTTTGAAATGTGTAATGTCAGAA

CTTAAATCTCTAGAGGACCGTAACCTTTGTCATCCAGGTCACAATCACCCATTAACTTTAGTCCAAAGCC

CAGCTTTGTTCCTGTGTCATGCTTGTAACACTACAGCTACAGACTTGTCCTATATCTGTACAAGATGTTG

TTTTTATATACACAAGAATTGTGCCAATGCACCCACCACCTATCAAAGTAAATTCCACAATGAACACGCT

CTCATCTTGACCTACTCTCTTCCCCAACAATATCGTCAATATCTTTGTTACTGCAGCATCTGCGAGAAAC

ATATAAATCCCATCTACTGGGTTTATATTTGTGCGAATTGCAGATTTTTTGCTCACGTGAAATGTGCTTC

ATTAACTGAGATGTTGAGGTTGATGTACTATCTCCTATTATTATTTCTTATATATTTGATATTGAATACT

TATTTAATAAATATTTAGTTTTCTGACATTGTTGTTTTACTTGAAATTATTCTGATTGGATATGAATCTT

TCTTTTGGAATAGTGAAAGTGATATTGATGATGAAGCAGATTGCGGAGAGTCTAATTTGATGCAGTTCCC

AGTGCATGATGAAGCATCACTACATGAAACAATGCAAGAATGTATCATCAAGGCAGCAAATTCTTCCACT

GGGGAACCTTCACCTTATATCAATCATTGGGCCCATGGACACCAACTGGCACTCGGAAACAAGAATGCAA

AAACTTTGCCCCTCAACTTGAAGCCGAAAATAGCAGAAACTGAATTGCTAATCTGTGATGGTTGCATGAA

TCCTATCTCCTTGGTTGATGTATTCTATGAATGCAGTTTATGCAATTTTTTTCTCCACAGATCATGCTCC

CAATTCCCCGAAAGGATTAAGCATCATCTAGCAGGCGATCTGGAGGGAATTCTAGCACGAATGTCAGGTG

AACTATATACTCTTGAATGCAGAGGTTGCGGCATTCGTGGTAATGGGATTTGCATGCACAATGATGAATA

CGCTTTTGACATCAAGTGCGCTTCATTACCCAGAATAATCAAACATAAAGGTCATCGTCACCCTCTTCAG

CAATTAAAGACTCCAGATGACTTTCTTTGCAAAGCATGTTGGCGTTACCGTGTAACTGAAGAGGTAAGAA

CCATCGTGTATGGATGTGAAAAATGTGATTTTTTCACACATATAGGGTGTGTGTTAAGTGCACAAGTGGT

GAAACATAGATGGGATCCTCATCCTCTCTACTTGATCCTGTCTCTTAAGAATGTACCTGATCATCCCCAT

GATTTCCATTGTGAATTTTGCCCCGATCAGATAAACACCAACAGTTGGTTTTATCACTGCAATGTATGCG

ATCTATCATTTCATACCAGCTGTATTGATCCAGATGATTGGGTGTCCAATATCAAGTTTGGTGCTACCGA

CATTTACAGCGACAAACATCCACATCCACACGGCCTCACATATATTCTCAATAAAAAGACGAGGAATTGC

AATTTATGTGGTAAAGATGCACGCGGTATGCGGGCTCTCCAATGTTCAACATGCAAATATATAGTGCATG

AGAATTGTTTCATTGAAGATTGAAATGAGGCAAAAATCAGATATTGTATTACTGGATTATTCAGGTTCAG

AGGATGTACAGAAAGAAGTAGCCACCG

>MSTRG.206.5 gene=MSTRG.206

TAATATATATATGAACAGAGTGCAGTGGTCGTGTTGAATCATTCATACTCGCCTTCAGTAAATATACATC

TCCCTTTGTCTCTCTCTCTCTCTCTCTGGTAAACATAGATGGCAGAATTGAAATTTTTTTTTCATGAGCA

TCAGCTGATACTAAATGAAGCTGAGCCTGTTGTCGGTAAGGATGTAGAATGTGTTGGGTGCAAACAGCCA

ATCAACAAACTCATAGACGCATTTTATAGGTGCAACAAGTCCCTTATTGATAGCAGTCCAGCGAGTGATT

GTGTTGGTTTCTATATGCATAAAACTTGTTCTGAGTTGCCCTCAACTTTTACACACCCTATGAACCCGAA

AAAACCTCTAAGCCTCTTTGTGCTTCCATATAAAAAAGAATACTTTTATATTTGTCATGCTTGTGACAGC

CACTCTCAGCACTTTATGTATGGTTCTGATTCTTCTGTCAGTTCCGAATTTCGTGTTTGTTTGAAATGTG

TAATGTCAGAACTTAAATCTCTAGAGGACCGTAACCTTTGTCATCCAGGTCACAATCACCCATTAACTTT

AGTCCAAAGCCCAGCTTTGTTCCTGTGTCATGCTTGTAACACTACAGCTACAGACTTGTCCTATATCTGT

ACAAGATGTTGTTTTTATATACACAAGAATTGTGCCAATGCACCCACCACCTATCAAAGTAAATTCCACA

ATGAACACGCTCTCATCTTGACCTACTCTCTTCCCCAACAATATCGTGAATATCCCTGTTACTGCAGCAT

CTGCGAGAAACATATAAATCCCATCTACTGGGTTTATATTTGTGCGAATTGCAGATTTTTTGCTCACGTG

AAATGTGCTTCATTAACTGAGTTGTTGAGGTTGATGTACTATCTCCTATTATTATTTCTTATATATTTGA

TATTGAATACTTATTTAATAAATATTTAGTTTTCTGACATTGTTGTTTTACTTGAAATTATTCTGATTGG

ATATGAATCTTTCTTTTGGAATAGTGAAAGTGATATTGATGATGAAGCAGATTGCGGAGAGTCTAATTTG

ATGCAGTTCCCAGTGCATGATGAAGCATCACTACATGAAACAATGCAAGAATGTATCATCAAGGCAGCAA

ATTCTTCCACTGGGGAACCTTCACCTTATATCAATCATTGGGCCCATGGACACCAACTGGCACTCGGAAA

CAAGAATGCAAAAACTTTGCCCCTCAACTTGAAGCCGAAAATAGCAGAAACTGAATTGCTAATCTGTGAT

GGTTGCATGAATCCTATCTCCTTGGTTGATGTATTCTATGAATGCAGTTTATGCAATTTTTTTCTCCACA

GATCATGCTCCCAATTCCCCGAAAGGATTAAGCATCATCTAGCAGGCGATCTGGAGGGAATTCTAGCACG

AATGTCAGGTGAACTATATACTCTTGAATGCAGAGGTTGCGGCATTCGTGGTAATGGGATTTGCATGCAC

AATGATGAATACGCTTTTGACATCAAGTGCGCTTCATTACCCAGAATAATCAAACATAAAGGTCATCGTC

ACCCTCTTCAGCAATTAAAGACTCCAGATGACTTTCTTTGCAAAGCATGTTGGCGTTACCGTGTAACTGA

AGAGGTAAGAACCATCGTGTATGGATGTGAAAAATGTGATTTTTTCACACATATAGGGTGTGTGTTAAGT

GCACAAGTGGTGAAACATAGATGGGATCCTCATCCTCTCTACTTGATCCTGTCTCTTAAGAATGTACCTG

ATCATCCCCATGATTTCCATTGTGAATTTTGCCCCGATCAGATAAACACCAACAGTTGGTTTTATCACTG

CAATGTATGCGATCTATCATTTCATACCAGCTGTATTGATCCAGATGATTGGGTGTCCAATATCAAGTTT

GGTGCTACCGACATTTACAGCGACAAACATCCACATCCACACGGCCTCACATATATTCTCAATAAAAAGA

CGAGGAATTGCAATTTATGTGGTAAAGATGCACGCGGTATGCGGGCTCTCCAATGTTCAACATGCAAATA

TATAGTGCATGAGAATTGTTTCATTGAAGATTGAAATGAGGCAAAAATCAGATATTGTATTACTGGATTA

TTCAGGTTCAGAGGATGTACAGAAAGAAGTAGCCACCG

>MSTRG.206.6 gene=MSTRG.206

TAATATATATATGAACAGAGTGCAGTGGTCGTGTTGAATCATTCATACTCGCCTTCAGTAAATATACATC

TCCCTTTGTCTCTCTCTCTCTCTCTCTGGTAAACATAGATGGCAGAATTGAAATTTTTTTTTCATGAGCA

TCAGCTGATACTAAATGAAGCTGAGCCTGTTGTCGGTAAGGATGTAGAATGTGTTGGGTGCAAACAGCCA

ATCAACAAACTCATAGACGCATTTTATAGGTGCAACAAGTCCCTTATTGATAGCAGTCCAGCGAGTGATT

GTGTTGGTTTCTATATGCATAAAACTTGTTCTGAGTTGCCCTCAACTTTTACACACCCTATGAACCCGAA

AAAACCTCTAAGCCTCTTTGTGCTTCCATATAAAAAAGAATACTTTTATATTTGTCATGCTTGTGACAGC

CACTCTCAGCACTTTATGTATGGTTCTGATTCTTCTGTCAGTTCCGAATTTCGTGTTTGTTTGAAATGTG

TAATGTCAGAACTTAAATCTCTAGAGGACCGTAACCTTTGTCATCCAGGTCACAATCACCCATTAACTTT

AGTCCAAAGCCCAGCTTTGTTCCTGTGTCATGCTTGTAACACTACAGCTACAGACTTGTCCTATATCTGT

ACAAGATGTTGTTTTTATATACACAAGAATTGTGCCAATGCACCCACCACCTATCAAAGTAAATTCCACA

ATGAACACGCTCTCATCTTGACCTACTCTCTTCCCCAACAATATCGTCAATATCTTTGTTACTGCAGCAT

CTGCGAGAAACATATAAATCCCATCTACTGGGTTTATATTTGTGCGAATTGCAGATTTTTTGCTCACGTG

AAATGTGCTTCATTAACTGAGATGTTGAGTGAAAGTGATATTGATGATGAAGCAGATTGCGGAGAGTCCA

GTTTGATGCAGTTCCCAGTGCATGATGAAGCATCACTACATGAAACAATGCAACAATGTATCATCAAGGC

CGTAAATCCTTCCACTGGGGAACCTTCACCTTATATCAATCATTGGGCCCATGGACACCAACTGGCACTC

GGAAACAAGAATGCAAAAACTTTGCCCCTCAACTTGAAGCCGAAAATAGCAGAAACTGAATTGCTAATCT

GTGATGGTTGCATGAATCCTATCTCCTTGGTTGATGTATTCTATGAATGCAGTTTATGCAATTTTTTTCT

CCACAGATCATGCTCCCAATTCCCCAAAAGGATTAAGCATCATCTAGCAGGCGATCTGGAGGCAGTGCTA

GCACGAGAGTCAGGTGAACTATATACTTTTCAATGCAGTGGTTGCGGCATTTATGGTAATGGGATTTGCA

TGCTCAATGATGAATACACTTTTGACATCAAGTGCGCTTCATTACCCAGAATAATCAAACATAAAGGTCA

TCGTCACCCTCTTCAGCAATTAAAGACTCCAGATGACTTTCTTTGCAAAGGTTGTTGGCGTGAGCCTGTA

ACTGAAGAGGTAAGAACCATCGTGTACGGATGTGAAAAATGTGAATTTTACACACATATAGGGTGTGTGT

TAAGTGCACAAGTGGTGAAACATAGGTGGGATCCTCATCCTCTCTACTTGATCCTGTCTCTCAAGAATGT

ACCCGATCATCCCCACGAATTCCCTTGTGAATTTTGCTCCAAAGAGATAAACACCAACAGTTGGTTTTAT

CACTGCAATGTATGCGATCTATCATTTCATATTAGCTGTATTGATCCAGATGATTGGCTGTCCAATATCA

AGTTTGGTGCCACCAACATTTACAGCGACAAACATCCTCATCCACACGGCCTCACATATATTCTCAATAA

AAAGACAAGGAATTGCAATTTATGTGGGTTTGCTTCAGTTGGGATTGCTTTTAGTGGGGATGAATAATTG

TGATTTGGGATTCTTTGACTTGGATAATATGATCTAGGGTTCTTTGGCTTGGAGTCTTGGACAATCTGAG

TTTGAGTGAAAGTGATATTGATGATGAAGCAGATTGCGGAGAGTCTAATTTGATGCAGTTCCCAGTGCAT

GATGAAGCATCACTACATGAAACAATGCAAGAATGTATCATCAAGGCAGCAAATTCTTCCACTGGGGAAC

CTTCACCTTATATCAATCATTGGGCCCATGGACACCAACTGGCACTCGGAAACAAGAATGCAAAAACTTT

GCCCCTCAACTTGAAGCCGAAAATAGCAGAAACTGAATTGCTAATCTGTGATGGTTGCATGAATCCTATC

TCCTTGGTTGATGTATTCTATGAATGCAGTTTATGCAATTTTTTTCTCCACAGATCATGCTCCCAATTCC

CCGAAAGGATTAAGCATCATCTAGCAGGCGATCTGGAGGGAATTCTAGCACGAATGTCAGGTGAACTATA

TACTCTTGAATGCAGAGGTTGCGGCATTCGTGGTAATGGGATTTGCATGCACAATGATGAATACGCTTTT

GACATCAAGTGCGCTTCATTACCCAGAATAATCAAACATAAAGGTCATCGTCACCCTCTTCAGCAATTAA

AGACTCCAGATGACTTTCTTTGCAAAGCATGTTGGCGTTACCGTGTAACTGAAGAGGTAAGAACCATCGT

GTATGGATGTGAAAAATGTGATTTTTTCACACATATAGGGTGTGTGTTAAGTGCACAAGTGGTGAAACAT

AGATGGGATCCTCATCCTCTCTACTTGATCCTGTCTCTTAAGAATGTACCTGATCATCCCCATGATTTCC

ATTGTGAATTTTGCCCCGATCAGATAAACACCAACAGTTGGTTTTATCACTGCAATGTATGCGATCTATC

ATTTCATACCAGCTGTATTGATCCAGATGATTGGGTGTCCAATATCAAGTTTGGTGCTACCGACATTTAC

AGCGACAAACATCCACATCCACACGGCCTCACATATATTCTCAATAAAAAGACGAGGAATTGCAATTTAT

GTGGTAAAGATGCACGCGGTATGCGGGCTCTCCAATGTTCAACATGCAAATATATAGTGCATGAGAATTG

TTTCATTGAAGATTGAAATGAGGCAAAAATCAGATATTGTATTACTGGATTATTCAGGTTCAGAGGATGT

ACAGAAAGAAGTAGCCACCG

>MSTRG.206.8 gene=MSTRG.206

GATTTGTTCAATGCAAACTCAAAACTGGTTTAAAGTCAAAAGCACGAGATTTTCATATCAAGGAGGTGGG

CATTAAGTCTGGGTTTTTCACTATGCGGCCTTCTACTTGAAAGTGATATTGATGATGAAGCAGATTGCGG

AGAGTCCAGTTTGATGCAGTTCCCAGTGCATGATGAAGCATCACTACATGAAACAATGCAACAATGTATC

ATCAAGGCCGTAAATCCTTCCACTGGGGAACCTTCACCTTATATCAATCATTGGGCCCATGGACACCAAC

TGGCACTCGGAAACAAGAATGCAAAAACTTTGCCCCTCAACTTGAAGCCGAAAATAGCAGAAACTGAATT

GCTAATCTGTGATGGTTGCATGAATCCTATCTCCTTGGTTGATGTATTCTATGAATGCAGTTTATGCAAT

TTTTTTCTCCACAGATCATGCTCCCAATTCCCCAAAAGGATTAAGCATCATCTAGCAGGCGATCTGGAGG

CAGTGCTAGCACGAGAGTCAGGTGAACTATATACTTTTCAATGCAGTGGTTGCGGCATTTATGGTAATGG

GATTTGCATGCTCAATGATGAATACACTTTTGACATCAAGTGCGCTTCATTACCCAGAATAATCAAACAT

AAAGGTCATCGTCACCCTCTTCAGCAATTAAAGACTCCAGATGACTTTCTTTGCAAAGGTTGTTGGCGTG

AGCCTGTAACTGAAGAGGTAAGAACCATCGTGTACGGATGTGAAAAATGTGAATTTTACACACATATAGG

GTGTGTGTTAAGTGCACAAGTGGTGAAACATAGGTGGGATCCTCATCCTCTCTACTTGATCCTGTCTCTC

AAGAATGTACCCGATCATCCCCACGAATTCCCTTGTGAATTTTGCTCCAAAGAGATAAACACCAACAGTT

GGTTTTATCACTGCAATGTATGCGATCTATCATTTCATATTAGCTGTATTGATCCAGATGATTGGCTGTC

CAATATCAAGTTTGGTGCCACCAACATTTACAGCGACAAACATCCTCATCCACACGGCCTCACATATATT

CTCAATAAAAAGACAAGGAATTGCAATTTATGTGGGGAAGATGCACGCGATACGCCGGCTCTCCAATGTT

CACCATGCAAATATATAGTGCATCGGAATTGTTTCATTAAAGAATGAAATCAGGGAAAATTATATTGTGT

ATTGCTGGATTGTATAGGTTCAGAAAATTTACAAATCCTTTGTGCTTGTATATAT

>MSTRG.206.9 gene=MSTRG.206

TCTCTCTTAAAAATAGATGGCAGAACTGAAACATTTTTTTCATGAGCATAAGCTGATACTGAATGAAGCT

GAGCCTGTTATCGATAAGGAAGTAGAATGTGTTGGGTGCAGACGGCCAATAAACAAACTCATAGACGCGT

TTTATAAGTGCAACAATTCCCTTATTGATAGCAGTCCATCGAGTGATTGTGTTGGTTTCTATATGCATAA

AACTTGTTCTGAGTTGCCCTCAACTTTTACACGCCCTTTGTTCCCAAAACAACCTCTAAGCCTCTTTGCG

CTTCCATATAAAAAACAAAACTTTTTTTCTTGTGATGCTTGTGACAGCGAATCTCAGTGCTTTATGTATT

GTTCTGAGTCTTCTGCCAATTGGGCCAATTCCGAATTTCTTGTTTGTTTGAAATGTGTAATATCAGAACT

TAAATCTCTAGAGGAACGAAACCGTTATCATCCGGGTCATGATCACCCATTAACCTTAGTCCAAAGCCCA

GCTTTGTTCCTGTGTCATGCTTGTAACACTACAGCAACAGATTTGTCCTATATTTGTACAACTTGTCCTT

TCTGGATACACGAGAGTTGTGCCAATGCACCCATCACCTACCAAAGTAAATTTCACAATGAACACGCTCT

CATCTTGAACTACTCTCTTCCCCAACAATTTCGTCAATTTACCTGTTTCTGCAGCATCTGCGATGAAAAT

ATAAATCCCATCGACTGGGTTTATATTTGTGCAAATTGCAGATTTTTTGCTCACGTGAAATGTGCTTTAT

CAACTTTGATTGAAAGTGATATTGATGATGAAGCAGATTGCGGAGAGTCCAGTTTGATGCAGTTCCCAGT

GCATGATGAAGCATCACTACATGAAACAATGCAACAATGTATCATCAAGGCCGTAAATCCTTCCACTGGG

GAACCTTCACCTTATATCAATCATTGGGCCCATGGACACCAACTGGCACTCGGAAACAAGAATGCAAAAA

CTTTGCCCCTCAACTTGAAGCCGAAAATAGCAGAAACTGAATTGCTAATCTGTGATGGTTGCATGAATCC

TATCTCCTTGGTTGATGTATTCTATGAATGCAGTTTATGCAATTTTTTTCTCCACAGATCATGCTCCCAA

TTCCCCAAAAGGATTAAGCATCATCTAGCAGGCGATCTGGAGGCAGTGCTAGCACGAGAGTCAGGTGAAC

TATATACTTTTCAATGCAGTGGTTGCGGCATTTATGGTAATGGGATTTGCATGCTCAATGATGAATACAC

TTTTGACATCAAGTGCGCTTCATTACCCAGAATAATCAAACATAAAGGTCATCGTCACCCTCTTCAGCAA

TTAAAGACTCCAGATGACTTTCTTTGCAAAGGTTGTTGGCGTGAGCCTGTAACTGAAGAGGTAAGAACCA

TCGTGTACGGATGTGAAAAATGTGAATTTTACACACATATAGGGTGTGTGTTAAGTGCACAAGTGGTGAA

ACATAGGTGGGATCCTCATCCTCTCTACTTGATCCTGTCTCTCAAGAATGTACCCGATCATCCCCACGAA

TTCCCTTGTGAATTTTGCTCCAAAGAGATAAACACCAACAGTTGGTTTTATCACTGCAATGTATGCGATC

TATCATTTCATATTAGCTGTATTGATCCAGATGATTGGCTGTCCAATATCAAGTTTGGTGCCACCAACAT

TTACAGCGACAAACATCCTCATCCACACGGCCTCACATATATTCTCAATAAAAAGACAAGGAATTGCAAT

TTATGTGGGGAAGATGCACGCGATACGCCGGCTCTCCAATGTTCACCATGCAAATATATAGTGCATCGGA

ATTGTTTCATTAAAGAATGAAATCAGGGAAAATTATATTGTGTATTGCTGGATTGTATAGGTTCAGAAAA

TTTACAAATCCTTTGTGCTTGTATATAT

>MSTRG.219.1 gene=MSTRG.219

GAGTGTAAAGTGCTGAATCGTTTCGTGATGCTATCTGTTTCGAAGTCTAAAAACAGTAAACAATGAAGAA

GATGCTCCAGAAAAACAAAACAATGATCTGCCTGATTGGGTTGGCAAGGGAAAACAGTAGTTCTACCAAA

CCCGCCTCAAAGTGGACAACGAGAAATGCCAACATTCTCATTCCGGAATTCCCACTTTGCTTGATATTTG

TCAGACAACAAAAATTATTTGTGGTCATGCAGCTGATATGAGGCAAAAGAACCCAAAGGCTGGCAACCCA

TTTAATGAAATTGCACCTATTAAATGGGAGATAATTTGTGACCACTGCGAGGGATTCCTTGTGACTCGGC

CAACTGTGTGCGAGAGAACATCAGAAGAGCACACAATTGCTGAAGGCGAAAGCATTGGAAGCAGCTATGA

ATGACATTAATAACTCATTTGGAAATGGAGCTGTAACAATATTAGGCAATGCTGGTGGATCTCTTGTTGA

CTCTTTTCCAAGTGGTTGTCTGACATTGGACATTGCATTGGGTGTCGGCCTTCCTAGAGGAAATATTGTT

GAGTAAACAATGAAGATGATGCTCCAAAAAGATGGAAAACAGTAACTAAGGCTGGCTAATCTGCTACTTA

TCTGCTATTAAGAAGCTGCTGCTTGTCTACTTGGTTATTTCGTTGAAGATAAAGCAACGCGACATATCGG

GAACTTTTGAAATTGCAAGAACTAAGTTAAAAGCTGGCAATGTAGATGATTTTTTTCCAAGTAGTTGAAC

CTACCAATGTATTCCTTACCATATTGTCTTATTCTTATTTCTATGTAACATCAATGCTCAAAAGACTTAA

AATCTAAGTTACTCAAGTAGTTTTTATATTTGTCTGTTTATTTATTTACCTATATAATGTATATAATACG

TTTGTGTGATGACAGAGGATCGATTCAGCACTTTGTAAAAAAAATTCATAGTTTAC

>MSTRG.219.2 gene=MSTRG.219

AGGCCTTTTAGTTAACCCAATTTATTTTTAGGTATAAAATCACTCGTCGTGTAGGTTAAGGCGGCAGCTT

CTCAATTACATTAGGGCATCGCCTAGATTAGGGCATCGCCGCAACGATCTATGATAATCGTGTGTGTATG

TATCTAGAGTGTAAAGTGCTGAATCGTTTCGTGATGCTATCTGTTTCGAAGTCTAAAAACAGTAAACAAT

GAAGAAGATGCTCCAGAAAAACAAAACAATGATCTGCCTGATTGGGTTGGCAAGGGAAAACAGTAGTTCT

ACCAAACCCGCCTCAAAGTGGACAACGAGAAATGCCAACATTCTCATTCCGGAATTCCCACTTTGCTTGA

TATTTGTCAGACAACAAAAATTATTTGTTACATTGGTCATGCAGCTGATATGAGGCAAAAGAACCCAAAG

GCTGGCAACCCATTTAATGAAATTGCACCTATTAAATGGGAGATAATTTGTGACCACTGCGAGGGATTCC

TTGTGACTCGGCCAACTGTGTGCGAGAGAACATCAGAAGAGCACACAATTGCTGAAGTGACTCTTTTCCA

AGTGGTTGTCTGACATTGGACATTGCATTGGGTGTCGGCCTTCCTAGAGGAAATATTGTTGAGGTATGTT

TTCGTACACACTTTGAGGTATTGTAAAACAAACTCAGTACAATCTTTTTCACGTGAAGATTATCTTGTTT

AATATCCGGGATACGTATGGCTCAGAGATGGTGAAGTAAACAATGAAGATGATGCTCCAAAAAGATGGAA

AACAGTAACTAAGGCTGGCTAATCTGCTACTTATCTGCTATTAAGAAGCTGCTGCTTGTCTACTTGGTTA

TTTCGTTGAAGATAAAGCAACGCGACATATCGGGAACTTTTGAAATTGCAAGAACTAAGTTAAAAGCTGG

CAATGTAGATGATTTTTTTCCAAGTAGTTGAACCTACCAATGTATTCCTTACCATATTGTCTTATTCTTA

TTTCTATGTAACATCAATGCTCAAAAGACTTAAAATCTAAGTTACTCAAGTAGTTTTTATATTTGTCTGT

TTATTTATTTACCTATATAATGTATATAATACGTTTGTGTGATGACAGAGGATCGATTCAG

>MSTRG.219.3 gene=MSTRG.219

AGGCCTTTTAGTTAACCCAATTTATTTTTAGGTATAAAATCACTCGTCGTGTAGGTTAAGGCGGCAGCTT

CTCAATTACATTAGGGCATCGCCTAGATTAGGGCATCGCCGCAACGATCTATGATAATCGTGTGTGTATG

TATCTAGAGTGTAAAGTGCTGAATCGTTTCGTGATGCTATCTGTTTCGAAGTCTAAAAACAGTAAACAAT

GAAGAAGATGCTCCAGAAAAACAAAACAATGATCTGCCTGATTGGGTTGGCAAGGGAAAACAGTAGTTCT

ACCAAACCCGCCTCAAAGTGGACAACGAGAAATGCCAACATTCTCATTCCGGAATTCCCACTTTGCTTGA

TATTTGTCAGACAACAAAAATTATTTGTGGTCATGCAGCTGATATGAGGCAAAAGAACCCAAAGGCTGGC

AACCCATTTAATGAAATTGCACCTATTAAATGGGAGATAATTTGTGACCACTGCGAGGGATTCCTTGTGA

CTCGGCCAACTGTGTGCGAGAGAACATCAGAAGAGCACACAATTGCTGAAGTGACTCTTTTCCAAGTGGT

TGTCTGACATTGGACATTGCATTGGGTGTCGGCCTTCCTAGAGGAAATATTGTTGAGGTATGTTTTCGTA

CACACTTTGAGGTATTGTAAAACAAACTCAGTACAATCTTTTTCACGTGAAGATTATCTTGTTTAATATC

CGGGATACGTATGGCTCAGAGATGGTGAAGTAAACAATGAAGATGATGCTCCAAAAAGATGGAAAACAGT

AACTAAGGCTGGCTAATCTGCTACTTATCTGCTATTAAGAAGCTGCTGCTTGTCTACTTGGTTATTTCGT

TGAAGATAAAGCAACGCGACATATCGGGAACTTTTGAAATTGCAAGAACTAAGTTAAAAGCTGGCAATGT

AGATGATTTTTTTCCAAGTAGTTGAACCTACCAATGTATTCCTTACCATATTGTCTTATTCTTATTTCTA

TGTAACATCAATGCTCAAAAGACTTAAAATCTAAGTTACTCAAGTAGTTTTTATATTTGTCTGTTTATTT

ATTTACCTATATAATGTATATAATACGTTTGTGTGATGACAGAG

>MSTRG.219.4 gene=MSTRG.219

TCTAAGAGCACATATATTAATATTTTAGTACTCTCATATCTGTCAATTTTTTAATTAATGCATAGAATAT

GTAATTCGTGAAATATTGAAATTCATTATGTCAGGTTGGCAAGGGAAAACAGTAGTTCTACCAAACCCGC

CTCAAAGTGGACAACGAGAAATGCCAACATTCTCATTCCGGAATTCCCACTTTGCTTGATATTTGTCAGA

CAACAAAAATTATTTGTGGTCATGCAGCTGATATGAGGCAAAAGAACCCAAAGGCTGGCAACCCATTTAA

TGAAATTGCACCTATTAAATGGGAGATAATTTGTGACCACTGCGAGGGATTCCTTGTGACTCGGCCAACT

GTGTGCGAGAGAACATCAGAAGAGCACACAATTGCTGAAGGCGAAAGCATTGGAAGCAGCTATGAATGAC

ATTAATAACTCATTTGGAAATGGAGCTGTAACAATATTAGGCAATGCTGGTGGATCTCTTGTGTATGTTG

ACTCTTTTCCAAGTGGTTGTCTGACATTGGACATTGCATTGGGTGTCGGCCTTCCTAGAGGAAATATTGT

TGAGGTATGTTTTCGTACACACTTTGAGGTATTGTAAAACAAACTCAGTACAATCTTTTTCACGTGAAGA

TTATCTTGTTTAATATCCGGGATACGTATGGCTCAGAGATGGTGAAGTAAACAATGAAGATGATGCTCCA

AAAAGATGGAAAACAGTAACTAAGGCTGGCTAATCTGCTACTTATCTGCTATTAAGAAGCTGCTGCTTGT

CTACTTGGTTATTTCGTTGAAGATAAAGCAACGCGACATATCGGGAACTTTTGAAATTGCAAGAACTAAG

TTAAAAGCTGGCAATGTAGATGATTTTTTTCCAAGTAGTTGAACCTACCAATGTATTCCTTACCATATTG

TCTTATTCTTATTTCTATGTAACATC

>MSTRG.219.5 gene=MSTRG.219

TGGGAAATTGGATTTCATTTGAAATTTTGGATTTGATCAAATAATCTGTTTGGGAATGTGGATTTGGATT

TCATTTGAAATCCAGACATTCAAATATTTGTATAAATTTGAGTGTTTGAAATGACAACTTAAATCTTGTC

ATTTGAAATGAAATGCATGTCTCCAAACGGCCTCTTATTTAAATTAAAACATCGAGGGAGGATCCATTTG

AATCAGCGCTATCGTGCAATTGGGATCAATTATATATGTTGGCAAGGGAAAACAGTAGTTCTACCAAACC

CGCCTCAAAGTGGACAACGAGAAATGCCAACATTCTCATTCCGGAATTCCCACTTTGCTTGATATTTTGG

TCATGCAGCTGATATGAGGCAAAAGAACCCAAAGGCTGGCAACCCATTTAATGAAATTGCACCTATTAAA

TGGGAGATAATTTGTGACCACTGCGAGGGATTCCTTGTGACTCGGCCAACTGTGTGCGAGAGAACATCAG

AAGAGCACACAATTGCTGAAGGCGAAAGCATTGGAAGCAGCTATGAATGACATTAATAACTCATTTGGAA

ATGGAGCTGTAACAATATTAGGCAATGCTGGTGGATCTCTTGTGTATGTTGACTCTTTTCCAAGTGGTTG

TCTGACATTGGACATTGCATTGGGTGTCGGCCTTCCTAGAGGAAATATTGTTGAGGTATGTTTTCGTACA

CACTTTGAGGTATTGTAAAACAAACTCAGTACAATCTTTTTCACGTGAAGATTATCTTGTTTAATATCCG

GGATACGTATGGCTCAGAGATGGTGAAGTAAACAATGAAGATGATGCTCCAAAAAGATGGAAAACAGTAA

CTAAGGCTGGCTAATCTGCTACTTATCTGCTATTAAGAAGCTGCTGCTTGTCTACTTGGTTATTTCGTTG

AAGATAAAGCAACGCGACATATCGGGAACTTTTGAAATTGCAAGAACTAAGTTAAAAGCTGGCAATGTAG

ATGATTTTTTTCCAAGTAGTTGAACCTACCAATGTATTCCTTACCATATTGTCTTATTCTTATTTCTATG

TAACATC

>MSTRG.219.6 gene=MSTRG.219

TTTAGGTATAAAATCACTCGTCGTGTAGGTTAAGGCGGCAGCTTCTCAATTACATTAGGGCATCGCCTAG

ATTAGGGCATCGCCGCAACGATCTATGATAATCGTGTGTGTATGTATCTAGAGTGTAAAGTGCTGAATCG

TTTCGTGATGCTATCTGTTTCGAAGTCTAAAAACAGTAAACAATGAAGAAGATGCTCCAGAAAAACAAAA

CAATGATCTGCCTGATTGGGTTGGCAAGGGAAAACAGTAGTTCTACCAAACCCGCCTCAAAGTGGACAAC

GAGAAATGCCAACATTCTCATTCCGGAATTCCCACTTTGCTTGATATTTTGGTCATGCAGCTGATATGAG

GCAAAAGAACCCAAAGGCTGGCAACCCATTTAATGAAATTGCACCTATTAAATGGGAGATAATTTGTGAC

CACTGCGAGGGATTCCTTGTGACTCGGCCAACTGTGTGCGAGAGAACATCAGAAGAGCACACAATTGCTG

AAGTGACTCTTTTCCAAGTGGTTGTCTGACATTGGACATTGCATTGGGTGTCGGCCTTCCTAGAGGAAAT

ATTGTTGAGGTATGTTTTCGTACACACTTTGAGGTATTGTAAAACAAACTCAGTACAATCTTTTTCACGT

GAAGATTATCTTGTTTAATATCCGGGATACGTATGGCTCAGAGATGGTGAAGTAAACAATGAAGATGATG

CTCCAAAAAGATGGAAAACAGTAACTAAGGCTGGCTAATCTGCTACTTATCTGCTATTAAGAAGCTGCTG

CTTGTCTACTTGGTTATTTCGTTGAAGATAAAGCAACGCGACATATCGGGAACTTTTGAAATTGCAAGAA

CTAAGTTAAAAGCTGGCAATGTAGATGATTTTTTTCCAAGTAGTTGAACCTACCAATGTATTCCTTACCA

TATTGTCTTATTCTTATTTCTAT

>MSTRG.219.7 gene=MSTRG.219

AAGTGGACAACGAGAAATGCCAACATTCTCATTGCGGAATTCCCACTTTGCTTGATATTTGTCAGACAAC

AAAAATTATTTGTTACATGTACAAACTTCATACAGTATATGCAAAATCGTGACAGTCAATCTTTATCTCT

CTAACAAGCTTTATATGTCCAATGTACTCTTTGTGTCCAGTGGTCATGCAGCTGATATGAGGCAAAAGAA

CCCAAAGGCTGGCAACCCATTTAATGAAATGCGAAAGCATTGGAAGCAGCTATGAATGACATTAATAACT

CATTTGGAAATGGAGCTGTAACAATATTAGGCAATGCTGGTGGATCTCTTGTTGACTCTTTTCCAAGTGG

TTGTCTGACATTGGACATTGCATTGGGTGTCGGCCTTCCTAGAGGAAATATTGTTGAGGTATGTTTTCGT

ACACACTTTGAGGTATTGTAAAACAAACTCAGTACAATCTTTTTCACGTGAAGATTATCTTGTTTAATAT

CCGGGATACGTATGGCTCAGAGATGGTGAAGTAAACAATGAAGATGATGCTCCAAAAAGATGGAAAACAG

TAACTAAGGCTGGCTAATCTGCTACTTATCTGCTATTAAGAAGCTGCTGCTTGTCTACTTGGTTATTTCG

TTGAAGATAAAGCAACGCGACATATCGGGAACTTTTGAAATTGCAAGAACTAAGTTAAAAGCTGGCAATG

TAGATGATTTTTTTCCAAGTAGTTGAACCTACCAATGTATTCCTTACCATATTGTCTTATTCTTATTTCT

AT

>MSTRG.220.1 gene=MSTRG.220

GCAAGGTCTGCAATTTTTTCTGTCTTCGAGTCTTTGGAATGTTGCTGTTATGGAATGCCCGAGGAAAATA

TAACTTCGGAATCAAGAAGTTTCGAAAGGTAGCTTGGCAAATCAGCCCAACCAAAGCTGCTCAGAAGTAC

AACTTAGATACAAGTTGTTTTTTCAAATATGTGTGAGTCCTTGTATTGATATCTATCAGGCTAACGAGTG

ATGCCACTATGGATATGATCTTCTAGTGAAATTTGTATATACATTCATTTATGCACGTATGTATATTAAT

GTGTTAACTGTACCAAATATTTATCATTTAATATTTTTATTCACGGCAATGTTGGTTTTAA

>MSTRG.220.2 gene=MSTRG.220

GCAAGGTCTGCAATTTTTTCTGTCTTCGAGTCTTTGGAATGTTGCTGTTATGGAATGCCCGAGGAAAATA

TAACTTCGGAATCAAGAAGTTTCGAAAGGTAGCTTGGCAAATCAGCCCAACCAAAGCTGCTCAGAAGTAC

AACTTAGATACAAGTTGTTTTTTCAAATATGTGTGAGTCCTTGTATTGATATCTATCAGGCTAACGAGTG

ATGCCACTATGGATATGATCTTCTAGCACGTATGTATATTAATGTGTTAACTGTACCAAATATTTATCAT

TTAATATTTTTATTCACGGCAATGTTGGTTTTAA

>MSTRG.221.1 gene=MSTRG.221

CTGCCATAAAACAAACTCAATACAATCTTATTCACGTAAAGATTGTTTTGTTTAATAATATCCGGGAGAC

GGATGGCTCATAAATGGTGAAGTAAACAAGGAAGATGATTTGCAGTAAACAATGAAGATGATGCTCCAAA

ATGATGAAAAACAATTATCTAAGGCCCGCTAATTTGCTAGTAACCATATTGTCTTATTCTTATTCCTATG

TAAGATCAGGGCTCAAAAG

>MSTRG.222.1 gene=MSTRG.222

TTTTTCACGTGAAGATTATCTCGTTTAATATCTGGGATACGTATGGCTCAGAGATGGTGAAGGTATGCTT

GTTATATAGTTATAATGTACTTGAAAATCTATTAAGTTTTCTAGTATTCAATTCACTGGTTTTCAAATTT

CTTCTTGCAGTAAACAATGAAGATGATGCTCCAAAAAGATGGAAAACAGTAACTAAGGCTGGCTAATCTG

CTACTTATCTGCTATTAAGAAGCTGCTGCTTGTCTACTTGGTTATTTCGTTGAAGATAAAGCAACGCGAC

ATATCGGGAACTTTTGAAATTGCAAGAACTAAGTTAAAAGCTGGCAATGTAGATGATTTTTTTCCAAGTA

GTTGAACCTACCAATGTATTCCTTACCATATTGTCTTATTCTTATTTCTATGTAACATCAATGCTCAAAA

GACTTAAAATCTAAGTTACTCAAGTAGTTTTTATATTTGTCTGTTTATTTATT

>MSTRG.222.2 gene=MSTRG.222

TTTTTCACGTGAAGATTATCTCGTTTAATATCTGGGATACGTATGGCTCAGAGATGGTGAAGTAAACAAT

GAAGATGATGCTCCAAAAAGATGGAAAACAGTAACTAAGGCTGGCTAATCTGCTACTTATCTGCTATTAA

GAAGCTGCTGCTTGTCTACTTGGTTATTTCGTTGAAGATAAAGCAACGCGACATATCGGGAACTTTTGAA

ATTGCAAGAACTAAGTTAAAAGCTGGCAATGTAGATGATTTTTTTCCAAGTAGTTGAACCTACCAATGTA

TTCCTTACCATATTGTCTTATTCTTATTTCTATGTAACATCAATGCTCAAAAGACTTAAAATCTAAGTTA

CTCAAGTAGTTTTTATATTTGTCTGTTTATTTATT

>MSTRG.223.1 gene=MSTRG.223

CTATCATTGATTAAATATTAAAAAATTTAGAATTTAACTTTAAAGGCGTTTTCTCTTGGCTTTTAATCTG

AAACACTTGCAATATCACTTGTACAAAAGGTAACCGAGAAGGTAATAGGAAAATTAATCCAGCTTCTGCA

AAGAGAGAAGGTATTCTACTTCTTTTGATCAGAGATTATTATATGTTCCTTGGACCCTGCAATAAAACCA

ACTCAATACAATCTTATTCACGTGAAGATTGTCTTGTTTAATAATATCTTTGAGACGGATGGCTCAGAAA

TGGTGAAGTAAACAAGGAAGATGATTTGCAGTAAACAATGAAGATGATGCTCCAAAATGATGAAAAACAA

TTTTCTAAGGC

>MSTRG.224.1 gene=MSTRG.224

CATGGCGTTACTCAGCAACGCTCTCCGGCAAGCCTTCATGCCGAAGCACGAGTACGACAATCTGAGCGAA

GAAGACAAAGCGCTGATCCAATTACAACGCCCCGTCTTGATTTCCCTCTTGTTGTGTATCGTGATTGTGA

TCGTTGTGTCCACCTCGATTAGCGTGAAGATTGTGTTCCCGGCGAAGGACGGGAAACGCGTGTTTTGCCG

GGATTTGAGGATTCAGCCGTTGTCGATAAATGTGAGCTCTGGTGGCGGTGGCGGCGGGGAGGATGTGTTT

CCTGGGGCGTTTTATTTGACGGATCAGCAGACGGTGGATTATTATTGGATGGTGGTGTTTATGCC

>MSTRG.225.1 gene=MSTRG.225

ATTAGATTAGGGCACGCCGCATCGATGGATAATAATCGTGTGTGTATGTATCTATTCGAGTGTATTCATA

ATTCAGTGTTGCTAGATCGATGTTCAAATTCTTTATGTTGTCCGTTTCGAAGCCGAAAACCAGTAAACAA

TGAAGAAGATGCTCCAGCAAAACAAAACTATGTTCTATTGCCACCTCAGTGGGCCGGCAAGGGATAAAAG

TGATTCTACCAAACCTTGCTCTAAGTGGACAAAAAGAAATGCCAACATTTCTCGATATGTCTTGCCGCTG

CTTCTTTTGGTGGAAAAGAACCCATAGGCTGGCACCACTGCTAGGGATTCCTTGTGACTCGGCTCACCGA

AATAAAAGAAGAACTCTTCATTTGAGCAAACAATTGCTGAAGGTTGATCTACTTTCTATTTAATTCAGTA

ACATATAAATGATGTCTCTTATATACTACTATGCATTGACAAAAATATATTAGTTTGGCCTTTAGTTCTT

GCTTCTCTTTCCC

>MSTRG.226.1 gene=MSTRG.226

TCCCTACTCTCTGCACTTTTGGCTATTTCTTTTATATTTTCTCTCAGCTTGCCTAGCGGTTATGTTCAGC

CTGGGACTTGGACAGGCACTTCAGTTACCCGCTCCGTAACTGAGGTCTCTGTGCCTCCTCAGAGATTTAC

TTATAAATGGCCTTCTCTGGACAATCTTTTCACACCGGGGAAAGCCGATCGGCATGAATATGACGGATTC

AGCTAGAGGGTCAAAGCGGGATAAAGTCTTAGATTTGACAGAGGCAAGGAAAGGTGAGGAAAATCCAGGC

ATCCAGCTGTATGCTCAAAAACAAACCTCCAGCGCAAGTGGAGACTGTTCTAAGTGACTGCTCCTCAGCA

GCAGTTCACCAACCTAAGCGCAAGAGCCATCGTTACATTCCACAGGCGGTGATTATTTTTTTCAACTTAC

TTTAAGGGGACTTGTGTGTTCATGCCTTGCTAATAATAGTTGTCATTTGTTTCTTGTATTCACATATGAA

ATGTTTTTGCAGCTCAAGGTAATACGTAGTTGCAGCTTAGTCATTCCACATGCAATTTTATTTTACAAGC

ACTTGTGATTTATGATAGAAATCTTCTCATTTATTGTGTCATTTCTCGGACTTCAATCTTCTTTTCAGGG

AGATCTTGTATGGGCAATCAGTTCAGACACGATTCATTCTCTCGTGGGACTCTCTGAACTTTTCTTTGTA

CTTCCGCTTATGAATTCAGGCCTGTGAGCTTTCAAGGATGTTCTTGCATTGGCGTCACTCGACAGGGCCA

ACAATTGAGCTGTTGAGAAACAAAAAAATAAAAGAGGCCTTCGAAGATGGTTTTAGGAGTTGCGTAAAGA

TTTTTATGGCCATTGATCCGAAATATATTGGGCAGACAAATTTGGCGAAGGGGTGGCCAAATGGATGAAA

GAATTTTCTGTTATGGAGGCCGTTGTATTACTGAGAGAAATGCTCTGTGAAAGCGAGAGATAGAGCACAC

CTTTAAAATCCAAATACAAAAGATGATGTTTGCTCAGAGAAAACAGAGGATGTACTTCAACTACTTTAAC

TTAAAAATTTATGAAATGTTTTGGGACTTCTTTTAGTGTGAGCAGCCTTTGAGGCTTTGCAAATTGTAGG

ATAATGGTGGACAAAAATTGTCTTGTCTTGATTAAGATATTGTATAAGCTGCTATTTATTTGGTTCTCAA

CTTTTAGACAAATGTGTTATCTTTAATGTAAAAGTTTAAATTTGCAA

>MSTRG.227.1 gene=MSTRG.227

TTTATTTTTAGGTATAAAATCACTCGTCGTGTAGGTTAAGGCGGCAGCTTCTCAATTACATTAGGGCATC

GCCTAGATTAGGGCATCGCCGCAACGATCTATGATAATCGTGTGTGTATGTATCTATAGTGTAAAGTGCT

GAATCGTTTCGTGATGCTATCTGTTTCGAAGTCTAAAAACAGTAAACAATGAAGAAGATGCTCCAGAAAA

ACAAAACAATGATCTGCCTGATTGGGTTGGCAAGGGAAAACAGTAGTTCTACCAAACCCGCCTCAAAGTG

GACAACGAGAAATGCCAACATTATCATTCCGGAATTCCCACTTTGCTTGATATTTGTCAGACAACAAAAA

TTATTTGTTACATGTACAAACTTCATACAGTATATGCAAAATCGTGACAGTCAATCTTTATCTCTCTAAC

AAGCTTTATATGTCCAATGTACTCTTTGTGTCCAGTGGTCATGCAGCTGATATGAGGCAAAAGAACCCAA

AGGCTGGCAACCCATTTAATGGAATGTATATAATACGTTTGTGTGATGACAGAGGATCGATTCAGCACTT

TGTAAAAAAAATTCATAGTTTACATGCTACTCACTTGACGTATATGTAATTAATATTATTTTTAAAATTT

TATACTAAAGAATGTCAAATCTTATATCTATATAAAGCAAAATCTCGGGCGTCTTACGTGGCGCTCTTTA

CGCTCTTGAATCAAAATCCCTGCTCTCTGCACTTTTGATTGTTGTTAGGAATTAATCTAATGTCCATATT

TGATGTAAATATTTTGTTACAGGCGAAAGCATTGGAAGCAGCTATGAATGACATTAATAACTCATTTGGA

AATGGAGCTGTAACAAGATTAGGCAATGCTGGTGGATCTCTTGTGTATGTTGACTCTTTTCCAAGTGGTT

GTCTGACATTGGACATTGCATTGGGTGTCGGCCTTCCTAGAGGAAATATTGTTGAGGTATGTTTTCGTAC

ACACTTTGAGGTATTGTAAAACAAACTCAGTACAATCTTTTTCACGTGAAGATTATCTCGTTTAATATCT

GGGATACGTATGGCTCAGAGATGGTGAAGTAAACAATGAAGATGATGCTCCAAAAAGATGGAAAACAGTA

ACTAAGGCTGGCTAATCTGCTACTTATCTGCTATTAAGAAGCTGCTGCTTGTCTACTTGGTTATTTCGTT

GAAGATAAAGCAACGCGACATATCGGGAACTTTTGAAATTGCAAGAACTAAGTTAAAAGCTGGCAATGTA

GATGATTTTTTTCCAAGTAGTTGAACCTACCAATGTATTCCTTACCATATTGTCTTATTCTTATTTCTAT

GTAACATCAATGCTCAAAAGACTTAAAATCTAAGTTACTCAAGTAGTTTTTATATTTGTCTGTTTATTTA

TTTACCTATATAATGTATATAATACGTTTGTGTGATGACAGAGGATCGATTCAGCACTTTGTAAAAAAAT

TCATAGTTTACATGCTACTCACTTGACGTATATGTAATTAATATTATTTTTAAAATTTTATACTAAAGAA

TGTCAAATCTTATATCTATATAAAGC

>MSTRG.227.2 gene=MSTRG.227

GTGGTCATGCAGCTGATATGAGGCAAAAGAACCCAAAGGCTGGCAACCCATTTAATGAAATTGCACCTAT

TAAATGGGAGATAATTTGTGACCACTGCGAGGGATTCCTTGTGACTCGGCGAACTGTGTGCGAGAGAACA

TCAGAAGAGCACACAATTGCTGAAGGCGAAAGCATTGGAAGCAGCTATGAATGACATTAATAACTCATTT

GGAAAAGGAGCTGTAACAAGATTAGGCAATGCTGGTGGATCTCTTGTTGACTCTTTTCCAAGTGGTTGTC

TGACATTGGACATTGCATTGGGTGTCGGCCTTCCTAGAGGAAATATTGTTGAGTAAACAATGAAGATGAT

GCTCCAAAAAGATGGAAAACAGTAACTAAGGCTGGCTAATCTGCTACTTATCTGCTATTAAGAAGCTGCT

GCTTGTCTACTTGGTTATTTCGTTGAAGATAAAGCAACGCGACATATCGGGAACTTTTGAAATTGCAAGA

ACTAAGTTAAAAGCTGGCAATGTAGATGATTTTTTTCCAAGTAGTTGAACCTACCAATGTATTCCTTACC

ATATTGTCTTATTCTTATTTCTATGTAACATCAATGCTCAAAAGACTTAAAATCTAAGTTACTCAAGTAG

TTTTTATATTTGTCTGTTTATTTATTTACCTATATAATGTATATAATACGTTTGTGTGATGACAGAGGAT

CGATTCAGC

>MSTRG.227.4 gene=MSTRG.227

TTTTAGTTAAACCAATTTATTTTTAGGTATAAAATCACTCGTCGTGTAGGTTAAGGCGGCAGCTTCTCAA

TTACATTAGGGCATCGCCTAGATTAGGGCATCGCCGCAACGATCTATGATAATCGTGTGTGTATGTATCT

AGAGTGTAAAGTGCTGAATCGTTTCGTGATGCTATCTGTTTCGAAGTCTAAAAACAGTAAACAATGAAGA

AGATGCTCCAGAAAAACAAAACAATGATCTGCCTGATTGGGTTGGCAAGGGAAAACAGTAGTTCTACCAA

ACCCGCCTCAAAGTGGACAACGAGAAATGCCAACATTATCATTCCGGAATTCCCACTTTGCTTGATATTT

GTCAGACAACAAAAATTATTTGTTACATTGGTCATGCAGCTGATATGAGGCAAAAGAACCCAAAGGCTGG

CAACCCATTTAATGGAATGCGAAAGCATTGGAAGCAGCTATGAATGACATTAATAACTCATTTGGAAATG

GAGCTGTAACAAGATTAGGCAATGCTGGTGGATCTCTTGTTGACTCTTTTCCAAGTGGTTGTCTGACATT

GGACATTGCATTGGGTGTCGGCCTTCCTAGAGGAAATATTGTTGAGGTATGTTTTCGTACACACTTTGAG

GTATTGTAAAACAAACTCAGTACAATCTTTTTCACGTGAAGATTATCTCGTTTAATATCTGGGATACGTA

TGGCTCAGAGATGGTGAAGTAAACAATGAAGATGATGCTCCAAAAAGATGGAAAACAGTAACTAAGGCTG

GCTAATCTGCTACTTATCTGCTATTAAGAAGCTGCTGCTTGTCTACTTGGTTATTTCGTTGAAGATAAAG

CAACGCGACATATCGGGAACTTTTGAAATTGCAAGAACTAAGTTAAAAGCTGGCAATGTAGATGATTTTT

TTCCAAGTAGTTGAACCTACCAATGTATTCCTTACCATATTGTCTTATTCTTATTTCTATGTAACATCAA

TGCTCAAAAGACTTAAAATCTAAGTTACTCAAGTAGTTTTTATATTTGTCTGTTTATTTATTTACCTATA

TAATGTATATAATACGTTTGTGTGATGACAGAGGATCGATTCA

>MSTRG.227.5 gene=MSTRG.227

TTTTAGTTAAACCAATTTATTTTTAGGTATAAAATCACTCGTCGTGTAGGTTAAGGCGGCAGCTTCTCAA

TTACATTAGGGCATCGCCTAGATTAGGGCATCGCCGCAACGATCTATGATAATCGTGTGTGTATGTATCT

AGAGTGTAAAGTGCTGAATCGTTTCGTGATGCTATCTGTTTCGAAGTCTAAAAACAGTAAACAATGAAGA

AGATGCTCCAGAAAAACAAAACAATGATCTGCCTGATTGGGTAAAATTCTGATCTTTGTAATGAAAGGCT

TGTTTAATGGACTAGCTAAATGCAATTTAATTGTGGTCCGTTTTGAACATATATGTGACAACTGGTAGTG

GTCGGCTATATGCTGTTGATAGATCGTGCCCTTGATCGGAATTTTTTGTTGTCTGTTTTGAACATATACG

TGGTCAGATTTATTGATGGGTTTTGAATGCTGGTAACTAACCCTATGATATATTGTGCCCTTGGTTGTGT

TGGTTAGCTGTATCGGAATATTTTGTCATAACATAAGTGGGTAATTTGTGTTAGTTTTGAGCGCCCTTGT

TCTTGTACTATAATATGGAAGGTAATTGAAAAACTAAACCACATTTCGATCTCTATCTATGAAAATACTT

TCTCTTATTGGAGAGATGATAAAGGGAAGCCATAATCTCGAAATTGAACGATTAATGGTCATTAAGAATC

CAGATATTGTTGATAAGATTTTATTGTATCTTGTTGGATTACATTTATGGCCTGTTTGGGAAATTGGATT

TCATTTGAAATTTTGGATTTGATCAAATAATCTGTTTGGGAATGTGGATTTGGATTTCATTTGAAATCCA

GACATTCAAATATTTGTATAAATTTGAGTGTTGGAGATGACAACTTAAATCTTGTCATTTGAAATGAAAT

GCATGTCTCCAAATGGCCTCTTATTTAAATTAAAACATCGAGGGAGGATCCATTTGAATCAGCGCTATCG

TGCAATTGGGATCAATTATATATGTTGGCAAGGGAAAACAGTAGTTCTACCAAACCCGCCTCAAAGTGGA

CAACGAGAAATGCCAACATTATCATTCCGGAATTCCCACTTTGCTTGATATTTGTCAGACAACAAAAATT

ATTTGTTACATGTACAAACTTCATACAGTATATGCAAAATCGTGACAGTCAATCTTTATCTCTCTAACAA

GCTTTATATGTCCAATGTACTCTTTGTGTCCAGTGGTCATGCAGCTGATATGAGGCAAAAGAACCCAAAG

GCTGGCAACCCATTTAATGGAATGTATATAATACGTTTGTGTGATGACAGAGGATCGATTCAGCACTTTG

TAAAAAAAATTCATAGTTTACATGCTACTCACTTGACGTATATGTAATTAATATTATTTTTAAAATTTTA

TACTAAAGAATGTCAAATCTTATATCTATATAAAGCAAAATCTCGGGCGTCTTACGTGGCGCTCTTTACG

CTCTTGAATCAAAATCCCTGCTCTCTGCACTTTTGATTGTTGTTAGGAATTAATCTAATGTCCATATTTG

ATGTAAATATTTTGTTACAGGCGAAAGCATTGGAAGCAGCTATGAATGACATTAATAACTCATTTGGAAA

TGGAGCTGTAACAAGATTAGGCAATGCTGGTGGATCTCTTGTGTATGTTGACTCTTTTCCAAGTGGTTGT

CTGACATTGGACATTGCATTGGGTGTCGGCCTTCCTAGAGGAAATATTGTTGAGTAAACAATGAAGATGA

TGCTCCAAAAAGATGGAAAACAGTAACTAAGGCTGGCTAATCTGCTACTTATCTGCTATTAAGAAGCTGC

TGCTTGTCTACTTGGTTATTTCGTTGAAGATAAAGCAACGCGACATATCGGGAACTTTTGAAATTGCAAG

AACTAAGTTAAAAGCTGGCAATGTAGATGATTTTTTTCCAAGTAGTTGAACCTACCAATGTATTCCTTAC

CATATTGTCTTATTCTTATTTCTATGTAACATCAATGCTCAAAAGACTTAAAATCTAAGTTACTCAAGTA

GTTTTTATATTTGTCTGTTTATTTATTTACCTATATAATGTATATAATACGTTTGTGTGATGACAGAGGA

TCGATTCA

>MSTRG.227.3 gene=MSTRG.227

AGTGTAAAGTGCTGAATCGTTTCGTGATGCTATCTGTTTCGAAGTCTAAAAACAGTAAACAATGAAGAAG

ATGCTCCAGAAAAACAAAACAATGATCTGCCTGATTGGGTTGGCAAGGGAAAACAGTAGTTCTACCAAAC

CCGCCTCAAAGTGGACAACGAGAAATGCCAACATTCTCATTGCGGAATTCCCACTTTGCTTGATATTTGT

CAGACAACAAAAATTATTTGTGGTCATGCAGCTGATATGAGGCAAAAGAACCCAAAGGCTGGCAACCCAT

TTAATGAAATTGCACCTATTAAATGGGAGATAATTTGTGACCACTGCGAGGGATTCCTTGTGACTCGGCG

AACTGTGTGCGAGAGAACATCAGAAGAGCACACAATTGCTGAAGGCGAAAGCATTGGAAGCAGCTATGAA

TGACATTAATAACTCATTTGGAAAAGGAGCTGTAACAAGATTAGGCAATGCTGGTGGATCTCTTGTTGAC

TCTTTTCCAAGTGGTTGTCTGACATTGGACATTGCATTGGGTGTCGGCCTTCCTAGAGGAAATATTGTTG

AGGTATGTTTTCGTACACACTTTGAGGTATTGTAAAACAAACTCAGTACAATCTTTTTCACGTGAAGATT

ATCTTGTTTAATATCCGGGATACGTATGGCTCAGAGATGGTGAAGTAAACAATGAAGATGATGCTCCAAA

AAGATGGAAAACAGTAACTAAGGCTGGCTAATCTGCTACTTATCTGCTATTAAGAAGCTGCTGCTTGTCT

ACTTGGTTATTTCGTTGAAGATAAAGCAACGCGACATATCGGGAACTTTTGAAATTGCAAGAACTAAGTT

AAAAGCTGGCAATGTAGATGATTTTTTTCCAAGTAGTTGAACCTACCAATGTATTCCTTACCATATTGTC

TTATTCTTATTTCTATGTAACATCAATGCTCAAAAGACTTAAAATCTAAGTTACTCAAGTAGTTTTTATA

TTTGTCTGTTTATTTATTTACCTATATAATGTATATAATACGTTTGTGTGATGACAGAGGATCGATTCA

>MSTRG.227.7 gene=MSTRG.227

CACTCGTCGTGTAGGTTAAGGCGGCAGCTTCTCAATTACATTAGGGCATCGCCTAGATTAGGGCATCGCC

GCAACGATCTATGATAATCGTGTGTGTATGTATCTATAGTGTAAAGTGCTGAATCGTTTCGTGATGCTAT

CTGTTTCGAAGTCTAAAAACAGTAAACAATGAAGAAGATGCTCCAGAAAAACAAAACAATGATCTGCCTG

ATTGGGTTGGCAAGGGAAAACAGTAGTTCTACCAAACCCGCCTCAAAGTGGACAACGAGAAATGCCAACA

TTATCATTCCGGAATTCCCACTTTGCTTGATATTTGTCAGACAACAAAAATTATTTGTTACATGTACAAA

CTTCATACAGTATATGCAAAATCGTGACAGTCAATCTTTATCTCTCTAACAAGCTTTATATGTCCAATGT

ACTCTTTGTGTCCAGTGGTCATGCAGCTGATATGAGGCAAAAGAACCCAAAGGCTGGCAACCCATTTAAT

GGAATGTATATAATACGTTTGTGTGATGACAGAGGATCGATTCAGCACTTTGTAAAAAAAATTCATAGTT

TACATGCTACTCACTTGACGTATATGTAATTAATATTATTTTTAAAATTTTATACTAAAGAATGTCAAAT

CTTATATCTATATAAAGCAAAATCTCGGGCGTCTTACGTGGCGCTCTTTACGCTCTTGAATCAAAATCCC

TGCTCTCTGCACTTTTGATTGTTGTTAGGAATTAATCTAATGTCCATATTTGATGTAAATATTTTGTTAC

AGGCGAAAGCATTGGAAGCAGCTATGAATGACATTAATAACTCATTTGGAAATGGAGCTGTAACAAGATT

AGGCAATGCTGGTGGATCTCTTGTGTATGTTGACTCTTTTCCAAGTGGTTGTCTGACATTGGACATTGCA

TTGGGTGTCGGCCTTCCTAGAGGAAATATTGTTGAGTAAACAATGAAGATGATGCTCCAAAAAGATGGAA

AACAGTAACTAAGGCTGGCTAATCTGCTACTTATCTGCTATTAAGAAGCTGCTGCTTGTCTACTTGGTTA

TTTCGTTGAAGATAAAGCAACGCGACATATCGGGAACTTTTGAAATTGCAAGAACTAAGTTAAAAGCTGG

CAATGTAGATGATTTTTTTCCAAGTAGTTGAACCTACCAATGTATTCCTTACCATATTGTCTTATTCTTA

TTTCTATGTAACATC

>MSTRG.227.6 gene=MSTRG.227

TTTATTTTTAGGTATAAAATCACTCGTCGTGTAGGTTAAGGCGGCAGCTTCTCAATTACATTAGGGCATC

GCCTAGATTAGGGCATCGCCGCAACGATCTATGATAATCGTGTGTGTATGTATCTATAGTGTAAAGTGCT

GAATCGTTTCGTGATGCTATCTGTTTCGAAGTCTAAAAACAGTAAACAATGAAGAAGATGCTCCAGAAAA

ACAAAACAATGATCTGCCTGATTGGGTTGGCAAGGGAAAACAGTAGTTCTACCAAACCCGCCTCAAAGTG

GACAACGAGAAATGCCAACATTATCATTCCGGAATTCCCACTTTGCTTGATATTTGTCAGACAACAAAAA

TTATTTGTTACATGTACAAACTTCATACAGTATATGCAAAATCGTGACAGTCAATCTTTATCTCTCTAAC

AAGCTTTATATGTCCAATGTACTCTTTGTGTCCAGTGGTCATGCAGCTGATATGAGGCAAAAGAACCCAA

AGGCTGGCAACCCATTTAATGGAATGTATATAATACGTTTGTGTGATGACAGAGGATCGATTCAGCACTT

TGTAAAAAAAATTCATAGTTTACATGCTACTCACTTGACGTATATGTAATTAATATTATTTTTAAAATTT

TATACTAAAGAATGTCAAATCTTATATCTATATAAAGCAAAATCTCGGGCGTCTTACGTGGCGCTCTTTA

CGCTCTTGAATCAAAATCCCTGCTCTCTGCACTTTTGATTGTTGTTAGGAATTAATCTAATGTCCATATT

TGATGTAAATATTTTGTTACAGGCGAAAGCATTGGAAGCAGCTATGAATGACATTAATAACTCATTTGGA

AATGGAGCTGTAACAAGATTAGGCAATGCTGGTGGATCTCTTGTGTATGTTGACTCTTTTCCAAGTGGTT

GTCTGACATTGGACATTGCATTGGGTGTCGGCCTTCCTAGAGGAAATATTGTTGAGTAAACAATGAAGAT

GATGCTCCAAAAAGATGGAAAACAGTAACTAAGGCTGGCTAATCTGCTACTTATCTGCTATTAAGAAGCT

GCTGCTTGTCTACTTGGTTATTTCGTTGAAGATAAAGCAACGCGACATATCGGGAACTTTTGAAATTGCA

AGAACTAAGTTAAAAGCTGGCAATGTAGATGATTTTTTTCCAAGTAGTTGAACCTACCAATGTATTCCTT

ACCATATTGTCTTATTCTTATTTCTATGTAACATC

>MSTRG.227.8 gene=MSTRG.227

TTTTAGTTAAACCAATTTATTTTTAGGTATAAAATCACTCGTCGTGTAGGTTAAGGCGGCAGCTTCTCAA

TTACATTAGGGCATCGCCTAGATTAGGGCATCGCCGCAACGATCTATGATAATCGTGTGTGTATGTATCT

AGAGTGTAAAGTGCTGAATCGTTTCGTGATGCTATCTGTTTCGAAGTCTAAAAACAGTAAACAATGAAGA

AGATGCTCCAGAAAAACAAAACAATGATCTGCCTGATTGGGTTGGCAAGGGAAAACAGTAGTTCTACCAA

ACCCGCCTCAAAGTGGACAACGAGAAATGCCAACATTATCATTCCGGAATTCCCACTTTGCTTGATATTT

GTCAGACAACAAAAATTATTTGTTACATGTACAAACTTCATACAGTATATGCAAAATCGTGACAGTCAAT

CTTTATCTCTCTAACAAGCTTTATATGTCCAATGTACTCTTTGTGTCCAGTGGTCATGCAGCTGATATGA

GGCAAAAGAACCCAAAGGCTGGCAACCCATTTAATGGAATGCGAAAGCATTGGAAGCAGCTATGAATGAC

ATTAATAACTCATTTGGAAATGGAGCTGTAACAAGATTAGGCAATGCTGGTGGATCTCTTGTGTATGTTG

ACTCTTTTCCAAGTGGTTGTCTGACATTGGACATTGCATTGGGTGTCGGCCTTCCTAGAGGAAATATTGT

TGAGGTATGTTTTCGTACACACTTTGAGGTATTGTAAAACAAACTCAGTACAATCTTTTTCACGTGAAGA

TTATCTCGTTTAATATCTGGGATACGTATGGCTCAGAGATGGTGAAGTAAACAATGAAGATGATGCTCCA

AAAAGATGGAAAACAGTAACTAAGGCTGGCTAATCTGCTACTTATCTGCTATTAAGAAGCTGCTGCTTGT

CTACTTGGTTATTTCGTTGAAGATAAAGCAACGCGACATATCGGGAACTTTTGAAATTGCAAGAACTAAG

TTAAAAGCTGGCAATGTAGATGATTTTTTTCCAAGTAGTTGAACCTACCAATGTATTCCTTACCATATTG

>MSTRG.227.9 gene=MSTRG.227

TTTATTTTTAGGTATAAAATCACTCGTCGTGTAGGTTAAGGCGGCAGCTTCTCAATTACATTAGGGCATC

GCCTAGATTAGGGCATCGCCGCAACGATCTATGATAATCGTGTGTGTATGTATCTATAGTGTAAAGTGCT

GAATCGTTTCGTGATGCTATCTGTTTCGAAGTCTAAAAACAGTAAACAATGAAGAAGATGCTCCAGAAAA

ACAAAACAATGATCTGCCTGATTGGGTTGGCAAGGGAAAACAGTAGTTCTACCAAACCCGCCTCAAAGTG

GACAACGAGAAATGCCAACATTCTCATTGCGGAATTCCCACTTTGCTTGATATTTGTCAGACAACAAAAA

TTATTTGTTACATTGGTCATGCAGCTGATATGAGGCAAAAGAACCCAAAGGCTGGCAACCCATTTAATGA

AATTGCACCTATTAAATGGGAGATAATTTGTGACCACTGCGAGGGATTCCTTGTGACTCGGCGAACTGTG

TGCGAGAGAACATCAGAAGAGCACACAATTGCTGAAGGCGAAAGCATTGGAAGCAGCTATGAATGACATT

AATAACTCATTTGGAAAAGGAGCTGTAACAAGATTAGGCAATGCTGGTGGATCTCTTGTGTATGTTGACT

CTTTTCCAAGTGGTTGTCTGACATTGGACATTGCATTGGGTGTCGGCCTTCCTAGAGGAAATATTGTTGA

GGTATGTTTTCGTACACACTTTGAGGTATTGTAAAACAAACTCAGTACAATCTTTTTCACGTGAAGATTA

TCTTGTTTAATATCCGGGATACGTATGGCTCAGAGATGGTGAAGTAAACAATGAAGATGATGCTCCAAAA

TGATGAAAAACAATTATCTAAGGCCCGCTAATTTGCTAGTAACCATATTGTCTTATTCTTATTCCTATGT

AAGATCAGGGCTCAAAAGACTTAAAATAGATGATGTTCAAGTAACCATTAAGTTACTTGAAGTATTTCTA

GTATTTGTCTGTTTATTTATTGACCTGTATAATGTATATAATAAGCTTGTGTGTGTGTTAAAACAGGATT

CTGCATTTTGG

>MSTRG.227.10 gene=MSTRG.227

AGTGTAAAGTGCTGAATCGTTTCGTGATGCTATCTGTTTCGAAGTCTAAAAACAGGTATATATGTTGTGT

TTTTAAGCCCTAGATTTCTCAATTCTGATTGTTTAATAGTTAATTTATCTAATTTGAAATACACTAATTA

TCAGATTTCTTCTTGCAGTAAACAATGAAGAAGATGCTCCAGAAAAACAAAACAATGATCTGCCTGATTG

GGTTGGCAAGGGAAAACAGTAGTTCTACCAAACCCGCCTCAAAGTGGACAACGAGAAATGCCAACATTCT

CATTGCGGAATTCCCACTTTGCTTGATATTTGTCAGACAACAAAAATTATTTGTGGTCATGCAGCTGATA

TGAGGCAAAAGAACCCAAAGGCTGGCAACCCATTTAATGAAATTGCACCTATTAAATGGGAGATAATTTG

TGACCACTGCGAGGGATTCCTTGTGACTCGGCGAACTGTGTGCGAGAGAACATCAGAAGAGCACACAATT

GCTGAAGGCGAAAGCATTGGAAGCAGCTATGAATGACATTAATAACTCATTTGGAAAAGGAGCTGTAACA

AGATTAGGCAATGCTGGTGGATCTCTTGTTGACTCTTTTCCAAGTGGTTGTCTGACATTGGACATTGCAT

TGGGTGTCGGCCTTCCTAGAGGAAATATTGTTGAGGTATGTTTTCGTACACACTTTGAGGTATTGTAAAA

CAAACTCAGTACAATCTTTTTCACGTGAAGATTATCTTGTTTAATATCCGGGATACGTATGGCTCAGAGA

TGGTGAAGTAAACAATGAAGATGATGC

>MSTRG.228.1 gene=MSTRG.228

GGTCTCTGTGCCTCCTCAGAGATTTACTTATAAATGGCCTTCTCTGGACAATCTTTTCACACCGGGGAAA

GCCGATCGGCATGAATATGACGGATTCAGCTAGAGGGTCAAAGCGGGATAAAGTCTTAGATTTGACAGAG

GCAAGGAAAGGTGAGGAAAATCCAGGCATCCAGCTGTATGCTCAAAAACAAACCTCCAGCGCAAGTGGAG

ACTGTTCTAAGTGACTGCTCCTCAGCAGCAGTTCACCAACCTAAGCGCAAGAGCCATCGTTACATTCCAC

AGGGAGATCTTGTATGGGCAATCAGTTCAGACACGATTCATTCTCTCGTGGGACTCTCTGAACTTTTCTT

TGTACTTCCGCTTATGAATTCAGTTCTAGGCCTGTGAGCTTTCAAGGATGTTCTTGCATTGGCGTCACTC

GACAGGGCCAACAATTGAGCTGTTGAGAAACAAAAAAATAAAAGAGGCCTTCGAAGATGGTTTTAGGAGT

TGCGTAAAGATTTTTATGGCCATTGATCCGAAATATATTGGGCAGACAAATTTGGCGAAGGGGTGGCCAA

ATGGATGAAAGAATTTTCTGTTATGGAGGCCGTTGTATTACTGAGAGAAATGCTCTGTGAAAGCGAGAGA

TAGAGCACACCTTTAAAATCCAATTACAAAAGAGGATGTTTGCTCAGAGAAAACAGAGGATGTACTTCAA

CTACTTTAACTTAAAAATTTATGAAATGTTTTGGGACTTCTTTTAGTGTGAGCAGCCTTTGAGGCTTTGC

AAATTGTAGGATAATGGTGGACAAAAATTGTCTTGTCTTGATTAAGATATTGTATAAGCTGCTATTCATT

TGGTTCTCAACTTTTAGACAAATGTGTTATCTTTAATGTAAAAGTTTAAATTTGCAATTTTAAAGTTTTT

TATTTTTTTTTCATTCCTTCTTTCACCAGCTTCACATACCTCGATCTTTGATAACCTTTTTTCTGGAGCC

ATTCAACATCAGCTTGGTAATCTTTTCAAATTACAGCAGCTTAATCTCGGCCACACTGGTATTCTACAAG

GTGGCATATTTGGTTGGTTATTGAATTTATCTACTCTGGT

>MSTRG.228.3 gene=MSTRG.228

GGTCTCTGTGCCTCCTCAGAGATTTACTTATAAATGGCCTTCTCTGGACAATCTTTTCACACCGGGGAAA

GCCGATCGGCATGAATATGACGGATTCAGCTAGAGGGTCAAAGCGGGATAAAGTCTTAGATTTGACAGAG

GCAAGGAAAGGTGAGGAAAATCCAGGCATCCAGCTGTATGCTCAAAAACAAACCTCCAGCGCAAGTGGAG

ACTGTTCTAAGTGACTGCTCCTCAGCAGCAGTTCACCAACCTAAGCGCAAGAGCCATCGTTACATTCCAC

AGGGAGATCTTGTATGGGCAATCAGTTCAGACACGATTCATTCTCTCGTGGGACTCTCTGAACTTTTCTT

TGTACTTCCGCTTATGAATTCAGGCCTGTGAGCTTTCAAGGATGTTCTTGCATTGGCGTCACTCGACAGG

GCCAACAATTGAGCTGTTGAGAAACAAAAAAATAAAAGAGGCCTTCGAAGATGGTTTTAGGAGTTGCGTA

AAGATTTTTATGGCCATTGATCCGAAATATATTGGGCAGACAAATTTGGCGAAGGGGTGGCCAAATGGAT

GAAAGAATTTTCTGTTATGGAGGCCGTTGTATTACTGAGAGAAATGCTCTGTGAAAGCGAGAGATAGAGC

ACACCTTTAAAATCCAATTACAAAAGAGGATGTTTGCTCAGAGAAAACAGAGGATGTACTTCAACTACTT

TAACTTAAAAATTTATGAAATGTTTTGGGACTTCTTTTAGTGTGAGCAGCCTTTGAGGCTTTGCAAATTG

TAGGATAATGGTGGACAAAAATTGTCTTGTCTTGATTAAGATATTGTATAAGCTGCTATTCATTTGGTTC

TCAACTTTTAGACAAATGTG

>MSTRG.228.2 gene=MSTRG.228

ACAATGAAGATGATGCTCCAAAAAGATGGAAAACAGTAACTAAGGCTGGCTAATCTGCTACTTATCTGCT

ATTAAGAAGCTGCTGCTTGTCTACTTGGTTATTTCGTTGAAGATAAAGCAACGCGACATATCGGGAACTT

TTGAAATTGCAAGAACTAAGTTAAAAGCTGGCAATGTAGATGATTTTTTTCCAAGTAGTTGAACCTACCA

ATGTATTCCTTACCATATTGTCTTATTCTTATTTCTATGTAACATCAATGCTCAAAAGACTTAAAATCTA

AGTTACTCAAGTAGTTTTTATATTTGTCTGTTTATTTATTTACCTATATAATGTATATAATACGTTTGTG

TGATGACAGAGGATCGATTCAGCACTTTGTAAAAAAAATTCATAGTTTACATGCTACTCACTTGACGTAT

ATGTAATTAATATTATTTTTAAAATTTTATACTAAAGAATGTCAAATCTTATATCTATATAAAGCAAAAT

CTCGGGCGTCTTACGTGGCGCTCTTTACGCTCTTGAATCAAAATCCCTGCTCTCTGCACTTTTGTCTATT

TCTTTTATATTTTCTCTCAGCTTGCCTAGCGGTTATGTTCAGCCTGGGACTTGGACAGGCACTTCAGTTA

CCCGCTCCGTAACCGAGGTCTCTGTGCCTCCTCAGAGATTTACTTATAAATGGCCTTCTCTGGACAATCT

TTTCACACCGGGGAAAGCCGATCGGCATGAATATGACGGATTCAGCTAGAGGGTCAAAGCGGGATAAAGT

CTTAGATTTGACAGAGGCAAGGAAAGGTGAGGAAAATCCAGGCATCCAGCTGTATGCTCAAAAACAAACC

TCCAGCGCAAGTGGAGACTGTTCTAAGTGACTGCTCCTCAGCAGCAGTTCACCAACCTAAGCGCAAGAGC

CATCGTTACATTCCACAGGGAGATCTTGTATGGGCAATCAGTTCAGACACGATTCATTCTCTCGTGGGAC

TCTCTGAACTTTTCTTTGTACTTCCGCTTATGAATTCAGGTAAGCTGAATTATTATTGCTTGATAGAATG

AATTGATAAGCTTTATGGTGCCAGCAAGTAGAAAGCCAGGTTATATTCGCACAACGTCCTTTATCTGAGG

CCACCAAGTCTCTACCTCTTTATACTGCTTTTCCCAACTGATGTTTTTAAATAAGTTGTATAACTTTGTT

GTTCATTCTATGACGATGTGCCTAAGTTTGTCTGCTTATATATTGTTAGTTCGTAATCGTAATAAATTGT

GATTACACATAGATATAGACTATTCTTGTAGAGATAAAGTTTAATGTTTTAAAGAATATCTTTTTATTTA

TAAGATTGTTTTCAGTTCTAGGCCTGTGAGCTTTCAAGGATGTTCTTGCATTGGCGTCACTCGACAGGGC

CAACAATTGAGCTGTTGAGAAACAAAAAAATAAAAGAGGCCTTCGAAGATGGTTTTAGGAGTTGCGTAAA

GATTTTTATGGCCATTGATCCGAAATATATTGGGCAGACAAATTTGGCGAAGGGGTGGCCAAATGGATGA

AAGAATTTTCTGTTATGGAGGCCGTTGTATTACTGAGAGAAATGCTCTGTGAAAGCGAGAGATAGAGCAC

ACCTTTAAAATCCAATTACAAAAGAGGATGTTTGCTCAGAGAAAACAGAGGATGTACTTCAACTACTTTA

ACTTAAAAATTTATGAAATGTTTTGGGACTTCTTTTAGTGTGAGCAGCCTTTGAGGCTTTGCAAATTGTA

GGATAATGGTGGACAAAAATTGTCTTGTCTTGATTAAGATATTGTATAAGCTGCTATTCATTTGGTTCTC

AACTTTTAGACAAATGTG

>MSTRG.229.1 gene=MSTRG.229

GATACCATCATATCTCAGTAAATCAGTGTTTGTCGAGCACAAATTGTTCTTGCCGATGATTAGTTGCCCA

AAAATACTGTAAGACATACTGTAAAAAGAATTCAAGAGGCCGATAACAGCAGCGGAGATAATAAAGGCAG

TGATTCCCAGTTGCAAGGTCTGCAATTTTTTCTGTCTTCGAGTCTTTGGAATGTTGCTGTTATGGAATGC

CCAAGGAAAATATAACTTCGGAATCAAGAAGTTTCGAAAGG

>MSTRG.230.1 gene=MSTRG.230

ATGGCGTTACTCAGCAACGCTCTCCGGCAAGCCTTCATGCCGAAGCACGAGTACGACAATCTGCGCGAAG

AAGACAAAGCGATGATCCAATTACAACGCCCCGTCTTGATTTCCCTCTTGTTGTGTATCGTGATTGTGAT

CGTTGTGTCCACCTCGATTAGCGTGAAGATTGTGTTCCCGGCGGAGGACGGGAAACGCGTGTTTTGCCGG

GATTTGAGGATTCAGCCGTTGTCGATAAATGTGAGCTCTGGTGGCGGTGGCGGCGGGGAGGATGTGTTTC

CTGGGGCGTTTTATTTGACGGATCAGCAGACGGTGGATTATTATTGGATGGTGGTGTTTATGCCGTCGGT

TTTGG

>MSTRG.231.1 gene=MSTRG.231

CAATACTCGTAAGAAAGCCCAGGCCTTTTAGTTAACCCAATTTATTTTTAGGTATAAAATCACTCGTCGT

GTAGGTTAAGGCGGCAGCTTCTCAATTACATTAGGGCATCGCCTAGATTAGGGCATCGCCGCAACGATCT

ATGATAATCGTGTGTGTATGTATCTATTTGAGTGTAAAGTGCTGAATCGTTTCGTGATGCTATCTGTTTC

GAAGTTTAAAAACAGTAAACAATGAAGAAGATGCTCCAG

>MSTRG.231.2 gene=MSTRG.231

CAATACTCGTAAGAAAGCCCAGGCCTTTTAGTTAACCCAATTTATTTTTAGGTATAAAATCACTCGTCGT

GTAGGTTAAGGCGGCAGCTTCTCAATTACATTAGGGCATCGCCTAGATTAGGGCATCGCCGCAACGATCT

ATGATAATCGTGTGTGTATGTATCTATTTGAGTGTAAAGTGCTGAATCGTTTCGTGATGCTATCTGTTTC

GAAGTTTAAAAACAGTAAACAATGAAGAAGATGCTCCAGAAAAACAAAACTTTGGGTAAAATTCTGACAA

TACAGGTATGGAAGCGTCCCGTGGACGTGATGTTAATCTTATGGCCCCTGTTATCAGCTCAGATGTCAAC

CTCGGTAATACTAACACCGAGCTTTCCCTGGCCATCCTCCCTCCACAAGCACCAGGCGGAACTATTCCAA

GGAAACGACGCAGGCCTTGTTACGGGTGGATTGATGATGAGGACGACATTGATGACATTGTGCACTTAAG

AACTTCTCCTGG

>MSTRG.231.3 gene=MSTRG.231

CCTTTTAGTTAACCCAATTTATTTTTAGGTATAAAATCACTCGTCGTGTAGGTTAAGGCGGCAGCTTCTC

AATTACATTAGGGCATCGCCTAGATTAGGGCATCGCCGCAACGATCTATGATAATCGTGTGTGTATGTAT

CTATTTGAGTGTAAAGTGCTGAATCGTTTCGTGATGCTATCTGTTTCGAAGTTTAAAAACAGTAAACAAT

GAAGAAGATGCTCCAGAAAAACAAAACAATGATCTGCCTGATTGGGTTGGCAAGGGAAAACAGTAGTTCT

ACCAAACCCGCCTCAAAGTGGACAACGAGAAATGCCAACATATTCGTTGCGGAATTCCCACTTTGCTTGA

TATTTTGGTCATGCAGCTGATATGAGGCAAAAGAACCCAAAGGCTGGCAACCCATTTAATGAAATTGCAC

CTATTAAATGGGAGATAATTTGTGACCACTGCGAGGGATTCCTTGTGACTCGGCCAACTGTGTGCGAGAG

AACATCAGAAGAGCACACAATTGCTGAAGGCGAAAGCATTGGAAGCAGCTATGAATGACATTAATAACTC

ATTTGGAAAAGGAGCTGTAACAAGATTAGGCAATGCTGGTGGATCTCTTGTTGACTCTTTTCCAAGTGGT

TGTCTGACATTGGACATTGCATTGGGTGTCGGCCTTCCTAGAGGAAATATTGTTGAGGATACGTATGGCT

CTGAGATGGTGAAGTAAACAATGAAGATGATGCTCCAAAAAGATGGAAAACAGTAACTAAGGCTGGCTAA

TCTGCTACTTATCTGCTATTAAGAAGCTGCTGCTTGTCTACTTGGTTATTTCGTTGAAGATAAAGCAACG

CGATGTATTCCTTACCATATTGTCTTATTCTTATTTCTATGTAACATCAATGCTCAAAAGACTTAAAATC

TAAGTTACTCAAGTAGTTTTTATATTTGTCTGTTTATTTATTTACCTATATAATGTATATAATACGTTTG

TGTGATGACAGAGGATCGATTCAGCACTTTGTAAAAAAAATTCATAGTTTACATGCTACTCACTTGACGT

ATATGTAATTAATATTATTTTTAAAGTTTTATACTAAAGAATGTCAAATCTTATATCTATATAAAGCAAA

ATCTCGGGCGTCTTACGTGGCGCTCTTTACGCTCTTGAATCAAAATCCCTGCTCTCTGCACTTTTGGCTA

TTTCTTTTATATTTTCTCTCAGCTTGCCTAGCGGTTATGTTCAGCCTGGGACTTGGACAGGCACTTCAGT

TACCCGCTCCGTAACTGAGGTCTCTGTGCCTCCTCAGAGATTTACTTATAAATGGCCTTCTCTGGACAAT

CTTTTCACACCGGGGAAAGCCGATCGGCATGAATATGACGGATTCAGCTAGAGGGTCAAAGCGGGATAAA

GTCTTAGATTTGACAGAGGCAAGGAAAGGTGAGGAAAATCCAGGCATCCAGCTGTATGCTCAAAAACAAA

CCTCCAGCGCAAGTGGAGACTGTTCTAAGTGACTGCTCCTCAGCAGCAGTTCACCAACCTAAGCGCAAGA

GCCATCGTTACATTCCACAGGGAGATCTTGTATGGGCAATCAGTTCAGACACGATTCATTCTCTCGTGGG

ACTCTCTGAACTTTTCTTTGTACTTCCGCTTATGAATTCAGGCCTGTGAGCTTTCAAGGATGTTCTTGCA

TTGGCGTCACTCGACAGGGCCAACAATTGAGCTGTTGAGAAACAAAAAAATAAAAGAGGCCTTCGAAGAT

GGTTTTAGGAGTTGCGTAAAGATTTTTATGGCCATTGATCCGAAATATATTGGGCAGACAAATTTGGCGA

AGGGGTGGCCAAATGGATGAAAGAATTTTCTGTTATGGAGGCCGTTGTATTACTGAGAGAAATGCTCTGT

GAAAGCGAGAGATAGAGCACACCTTTAAAATCCAAATACAAAAGATGATGTTTGCTCAGAGAAAACAGAG

GATGTACTTCAACTACTTTAACTTAAAAATTTATGAAATGTTTTGGGACTTCTTTTAGTGTGAGCAGCCT

TTGAGGCTTTGCAAATTGTAGGATAATGGTGGACAAAAATTGTCTTGTCTTGATTAAGATATTGTATAAG

CTGCTATTCATTTGGTTCTCAACTTTTAGACAAATGTGTTATCTTTAATGTAAAAGTTTAAATTTGCAAT

TTTAAAGTTTTTTATTTTTTTTTCATTCCTTCTTTCACCAGCTTCACATACCTCGATCTTTGATAACCTT

TTTTCTGGAGCCATTCAACATCAGCTTGGTAATCTTTTCAAATTACAGCAGCTTAATCTCGGCCACACTG

GTATTCTACAAGGTGGCATATTTGGTTGGTTATTGAATTTATCTACTCTGGT

>MSTRG.231.4 gene=MSTRG.231

CAGGCCTTTTAGTTAACCCAATTTATTTTTAGGTATAAAATCACTCGTCGTGTAGGTTAAGGCGGCAGCT

TCTCAATTACATTAGGGCATCGCCTAGATTAGGGCATCGCCGCAACGATCTATGATAATCGTGTGTGTAT

GTATCTATTTGAGTGTAAAGTGCTGAATCGTTTCGTGATGCTATCTGTTTCGAAGTTTAAAAACAGTAAA

CAATGAAGAAGATGCTCCAGAAAAACAAAACAATGATCTGCCTGATTGGGTTGGCAAGGGAAAACAGTAG

TTCTACCAAACCCGCCTCAAAGTGGACAACGAGAAATGCCAACATATTCGTTGCGGAATTCCCACTTTGC

TTGATATTTTGGTCATGCAGCTGATATGAGGCAAAAGAACCCAAAGGCTGGCAACCCATTTAATGAAATT

GCACCTATTAAATGGGAGATAATTTGTGACCACTGCGAGGGATTCCTTGTGACTCGGCCAACTGTGTGCG

AGAGAACATCAGAAGAGCACACAATTGCTGAAGGCGAAAGCATTGGAAGCAGCTATGAATGACATTAATA

ACTCATTTGGAAAAGGAGCTGTAACAAGATTAGGCAATGCTGGTGGATCTCTTGTGTATGTTGACTCTTT

TCCAAGTGGTTGTCTGACATTGGACATTGCATTGGGTGTCGGCCTTCCTAGAGGAAATATTGTTGAGGAT

ACGTATGGCTCTGAGATGGTGAAGTAAACAATGAAGATGATGCTCCAAAAAGATGGAAAACAGTAACTAA

GGCTGGCTAATCTGCTACTTATCTGCTATTAAGAAGCTGCTGCTTGTCTACTTGGTTATTTCGTTGAAGA

TAAAGCAACGCGACATATCGGGAACTTTTGAAATTGCAAGAACTAAGTTAAAAGCTGGCAATGTAGATGA

TTTTTTTCCAAGTAGTTGAACCTACCAATGTATTCCTTACCATATTGTCTTATTCTTATTTCTATGTAAC

ATCAATGCTCAAAAGACTTAAAATCTAAGTTACTCAAGTAGTTTTTATATTTGTCTGTTTATTTATTTAC

CTATATAATGTATATAATACGTTTGTGTGATGACAGAGGATCGATTCAGCACTTTGTAAAAAAAATTCAT

AGTTTACATGCTACTCACTTGACGTATATGTAATTAATATTATTTTTAAAGTTTTATACTAAAGAATGTC

AAATCTTATATCTATATAAAGCAAAATCTCGGGCGTCTTACGTGGCGCTCTTTACGCTCTTGAATCAAAA

TCCCTGCTCTCTGCACTTTTGGCTATTTCTTTTATATTTTCTCTCAGCTTGCCTAGCGGTTATGTTCAGC

CTGGGACTTGGACAGGCACTTCAGTTACCCGCTCCGTAACTGAGGTCTCTGTGCCTCCTCAGAGATTTAC

TTATAAATGGCCTTCTCTGGACAATCTTTTCACACCGGGGAAAGCCGATCGGCATGAATATGACGGATTC

AGCTAGAGGGTCAAAGCGGGATAAAGTCTTAGATTTGACAGAGGCAAGGAAAGGTGAGGAAAATCCAGGC

ATCCAGCTGTATGCTCAAAAACAAACCTCCAGCGCAAGTGGAGACTGTTCTAAGTGACTGCTCCTCAGCA

GCAGTTCACCAACCTAAGCGCAAGAGCCATCGTTACATTCCACAGGGAGATCTTGTATGGGCAATCAGTT

CAGACACGATTCATTCTCTCGTGGGACTCTCTGAACTTTTCTTTGTACTTCCGCTTATGAATTCAGTTCT

AGGCCTGTGAGCTTTCAAGGATGTTCTTGCATTGGCGTCACTCGACAGGGCCAACAATTGAGCTGTTGAG

AAACAAAAAAATAAAAGAGGCCTTCGAAGATGGTTTTAGGAGTTGCGTAAAGATTTTTATGGCCATTGAT

CCGAAATATATTGGGCAGACAAATTTGGCGAAGGGGTGGCCAAATGGATGAAAGAATTTTCTGTTATGGA

GGCCGTTGTATTACTGAGAGAAATGCTCTGTGAAAGCGAGAGATAGAGCACACCTTTAAAATCCAAATAC

AAAAGATGATGTTTGCTCAGAGAAAACAGAGGATGTACTTCAACTACTTTAACTTAAAAATTTATGAAAT

GTTTTGGGACTTCTTTTAGTGTGAGCAGCCTTTGAGGCTTTGCAAATTGTAGGATAATGGTGGACAAAAA

TTGTCTTGTCTTGATTAAGATATTGTATAAGCTGCTATTCATTTGGTTCTCAACTTTTAGACAAATG

>MSTRG.231.7 gene=MSTRG.231

TAGATTAGGGCATCGCCGCAACGATCTATGATAATCGTGTGTGTATGTATCTATTTGAGTGTAAAGTGCT

GAATCGTTTCGTGATGCTATCTGTTTCGAAGTTTAAAAACAGTAAACAATGAAGAAGATGCTCCAGAAAA

ACAAAACAATGATCTGCCTGATTGGGTTGGCAAGGGAAAACAGTAGTTCTACCAAACCCGCCTCAAAGTG

GACAACGAGAAATGCCAACATATTCGTTGCGGAATTCCCACTTTGCTTGATATTTTGGTCATGCAGCTGA

TATGAGGCAAAAGAACCCAAAGGCTGGCAACCCATTTAATGAAATTGCACCTATTAAATGGGAGATAATT

TGTGACCACTGCGAGGGATTCCTTGTGACTCGGCCAACTGTGTGCGAGAGAACATCAGAAGAGCACACAA

TTGCTGAAGGCGAAAGCATTGGAAGCAGCTATGAATGACATTAATAACTCATTTGGAAAAGGAGCTGTAA

CAAGATTAGGCAATGCTGGTGGATCTCTTGTTGACTCTTTTCCAAGTGGTTGTCTGACATTGGACATTGC

ATTGGGTGTCGGCCTTCCTAGAGGAAATATTGTTGAGTAAACAATGAAGATGATGCTCCAAAAAGATGGA

AAACAGTAACTAAGGCTGGCTAATCTGCTACTTATCTGCTATTAAGAAGCTGCTGCTTGTCTACTTGGTT

ATTTCGTTGAAGATAAAGCAACGCGACATATCGGGAACTTTTGAAATTGCAAGAACTAAGTTAAAAGCTG

GCAATGTAGATGATTTTTTTCCAAGTAGTTGAACCTACCAATGTATTCCTTACCATATTGTCTTATTCTT

ATTTCTATGTAACATCAATGCTCAAAAGACTTAAAATCTAAGTTACTCAAGTAGTTTTTATATTTGTCTG

TTTATTTATTTACCTATATAATGTATATAATACGTTTGTGTGATGACAGAGGATCGATTCAGCACTTTGT

AAAAAAAATTCATAGTTTACATGCTACTCACTTGACGTATATGTAATTAATATTATTTTTAAAGTTTTAT

ACTAAAGAATGTCAAATCTTATATCTATATAAAGCAAAATCTCGGGCGTCTTACGTGGCGCTCTTTACGC

TCTTGAATCAAAATCCCTGCTCTCTGCACTTTTGGCTATTTCTTTTATATTTTCTCTCAGCTTGCCTAGC

GGTTATGTTCAGCCTGGGACTTGGACAGGCACTTCAGTTACCCGCTCCGTAACTGAGGTCTCTGTGCCTC

CTCAGAGATTTACTTATAAATGGCCTTCTCTGGACAATCTTTTCACACCGGGGAAAGCCGATCGGCATGA

ATATGACGGATTCAGCTAGAGGGTCAAAGCGGGTATGTATGTTATTCTTGCTGATTTTCAGGGTTGATTT

ATGTTATATGATCATAGTCGTGGGCAGTTATCTCCTTCTGATTTATCCGGAGAGCATAGCAGCAGATTTT

TATGATAAAGTCTTAGATTTGACAGAGGCAAGGAAAGGTGAGGAAAATCCAGGCATCCAGCTGTATGCTC

AAAAACAAACCTCCAGCGCAAGTGGAGACTGTTCTAAGTGACTGCTCCTCAGCAGCAGTTCACCAACCTA

AGCGCAAGAGCCATCGTTACATTCCACAGGGAGATCTTGTATGGGCAATCAGTTCAGACACGATTCATTC

TCTCGTGGGACTCTCTGAACTTTTCTTTGTACTTCCGCTTATGAATTCAGGTAAGCTGAATTATTATTGC

TTGATAGAATGAATTGATAAGCTTTATGGTGCCAGCAAGTAGAAAGCCAGGTTATATTCGCACAACGTCC

TTTATCTGAGGCCACCAAGTCTCTACCTCTTTATACTGCTTTTCCCAACTGATGTTTTTAAATAAGTTGT

ATAACTTTGTTGTTCATTCTATGACGATGTGCCTAAGTTTGTCTGCTTATATATTGTTAGTTCGTAATCG

TAATAAATTGTGATTACACATAGATATAGACTATTCTTGTAGAGATAAAGTTTAATGTTTTAAAGAATAT

CTTTTTATTTATAAGATTGTTTTCAGTTCTAGGCCTGTGAGCTTTCAAGGATGTTCTTGCATTGGCGTCA

CTCGACAGGGCCAACAATTGAGCTGTTGAGAAACAAAAAAATAAAAGAGGCCTTCGAAGATGGTTTTAGG

AGTTGCGTAAAGATTTTTATGGCCATTGATCCGAAATATATTGGGCAGACAAATTTGGCGAAGGGGTGGC

CAAATGGATGAAAGAATTTTCTGTTATGGAGGCCGTTGTATTACTGAGAGAAATGCTCTGTGAAAGCGAG

AGATAGAGCACACCTTTAAAATCCAAATACAAAAGATGATGTTTGCTCAGAGAAAACAGAGGATGTACTT

CAACTACTTTAACTTAAAAATTTATGAAATGTTTTGGGACTTCTTTTAGTGTGAGCAGCCTTTGAGGCTT

TGCAAATTGTAGGATAATGGTGGACAAAAATTGTCTTGTCTTGATTAAGATATTGTATAAGCTGC

>MSTRG.231.8 gene=MSTRG.231

TAGATTAGGGCATCGCCGCAACGATCTATGATAATCGTGTGTGTATGTATCTATTTGAGTGTAAAGTGCT

GAATCGTTTCGTGATGCTATCTGTTTCGAAGTTTAAAAACAGTAAACAATGAAGAAGATGCTCCAGAAAA

ACAAAACAATGATCTGCCTGATTGGGTTGGCAAGGGAAAACAGTAGTTCTACCAAACCCGCCTCAAAGTG

GACAACGAGAAATGCCAACATATTCGTTGCGGAATTCCCACTTTGCTTGATATTTTGGTCATGCAGCTGA

TATGAGGCAAAAGAACCCAAAGGCTGGCAACCCATTTAATGAAATTGCACCTATTAAATGGGAGATAATT

TGTGACCACTGCGAGGGATTCCTTGTGACTCGGCCAACTGTGTGCGAGAGAACATCAGAAGAGCACACAA

TTGCTGAAGGCGAAAGCATTGGAAGCAGCTATGAATGACATTAATAACTCATTTGGAAAAGGAGCTGTAA

CAAGATTAGGCAATGCTGGTGGATCTCTTGTTGACTCTTTTCCAAGTGGTTGTCTGACATTGGACATTGC

ATTGGGTGTCGGCCTTCCTAGAGGAAATATTGTTGAGTAAACAATGAAGATGATGCTCCAAAAAGATGGA

AAACAGTAACTAAGGCTGGCTAATCTGCTACTTATCTGCTATTAAGAAGCTGCTGCTTGTCTACTTGGTT

ATTTCGTTGAAGATAAAGCAACGCGATGTATTCCTTACCATATTGTCTTATTCTTATTTCTATGTAACAT

CAATGCTCAAAAGACTTAAAATCTAAGTTACTCAAGTAGTTTTTATATTTGTCTGTTTATTTATTTACCT

ATATAATGTATATAATACGTTTGTGTGATGACAGAGGATCGATTCAGCACTTTGTAAAAAAAATTCATAG

TTTACATGCTACTCACTTGACGTATATGTAATTAATATTATTTTTAAAGTTTTATACTAAAGAATGTCAA

ATCTTATATCTATATAAAGCAAAATCTCGGGCGTCTTACGTGGCGCTCTTTACGCTCTTGAATCAAAATC

CCTGCTCTCTGCACTTTTGGCTATTTCTTTTATATTTTCTCTCAGCTTGCCTAGCGGTTATGTTCAGCCT

GGGACTTGGACAGGCACTTCAGTTACCCGCTCCGTAACTGAGGTCTCTGTGCCTCCTCAGAGATTTACTT

ATAAATGGCCTTCTCTGGACAATCTTTTCACACCGGGGAAAGCCGATCGGCATGAATATGACGGATTCAG

CTAGAGGGTCAAAGCGGGATAAAGTCTTAGATTTGACAGAGGCAAGGAAAGGTGAGGAAAATCCAGGCAT

CCAGCTGTATGCTCAAAAACAAACCTCCAGCGCAAGTGGAGACTGTTCTAAGTGACTGCTCCTCAGCAGC

AGTTCACCAACCTAAGCGCAAGAGCCATCGTTACATTCCACAGGGAGATCTTGTATGGGCAATCAGTTCA

GACACGATTCATTCTCTCGTGGGACTCTCTGAACTTTTCTTTGTACTTCCGCTTATGAATTCAGGCCTGT

GAGCTTTCAAGGATGTTCTTGCATTGGCGTCACTCGACAGGGCCAACAATTGAGCTGTTGAGAAACAAAA

AAATAAAAGAGGCCTTCGAAGATGGTTTTAGGAGTTGCGTAAAGATTTTTATGGCCATTGATCCGAAATA

TATTGGGCAGACAAATTTGGCGAAGGGGTGGCCAAATGGATGAAAGAATTTTCTGTTATGGAGGCCGTTG

TATTACTGAGAGAAATGCTCTGTGAAAGCGAGAGATAGAGCACACCTTTAAAATCCAAATACAAAAGATG

ATGTTTGCTCAGAGAAAACAGAGGATGTACTTCAACTACTTTAACTTAAAAATTTATGAAATGTTTTGGG

ACTTCTTTTAGTGTGAGCAGCCTTTGAGGCTTTGCAAATTGTAGGATAATGGTGGACAAAAATTGTCTTG

TCTTGATTAAGATATTGTATAAGCTGC

>MSTRG.231.6 gene=MSTRG.231

CAGGCCTTTTAGTTAACCCAATTTATTTTTAGGTATAAAATCACTCGTCGTGTAGGTTAAGGCGGCAGCT

TCTCAATTACATTAGGGCATCGCCTAGATTAGGGCATCGCCGCAACGATCTATGATAATCGTGTGTGTAT

GTATCTATTTGAGTGTAAAGTGCTGAATCGTTTCGTGATGCTATCTGTTTCGAAGTTTAAAAACAGTAAA

CAATGAAGAAGATGCTCCAGAAAAACAAAACAATGATCTGCCTGATTGGGTTGGCAAGGGAAAACAGTAG

TTCTACCAAACCCGCCTCAAAGTGGACAACGAGAAATGCCAACATATTCGTTGCGGAATTCCCACTTTGC

TTGATATTTTGGTCATGCAGCTGATATGAGGCAAAAGAACCCAAAGGCTGGCAACCCATTTAATGAAATT

GCACCTATTAAATGGGAGATAATTTGTGACCACTGCGAGGGATTCCTTGTGACTCGGCCAACTGTGTGCG

AGAGAACATCAGAAGAGCACACAATTGCTGAAGGCGAAAGCATTGGAAGCAGCTATGAATGACATTAATA

ACTCATTTGGAAAAGGAGCTGTAACAAGATTAGGCAATGCTGGTGGATCTCTTGTTGACTCTTTTCCAAG

TGGTTGTCTGACATTGGACATTGCATTGGGTGTCGGCCTTCCTAGAGGAAATATTGTTGAGGTATGTTTT

CGTACACACTTTGAGGTATTGTAAAACAAACTCAGTACAATCTTTTTCACGTGAAGATTATCTTGTTTAA

TATCCAGGATACGTATGGCTCTGAGATGGTGAAGTAAACAATGAAGATGATGCTCCAAAAAGATGGAAAA

CAGTAACTAAGGCTGGCTAATCTGCTACTTATCTGCTATTAAGAAGCTGCTGCTTGTCTACTTGGTTATT

TCGTTGAAGATAAAGCAACGCGACATATCGGGAACTTTTGAAATTGCAAGAACTAAGTTAAAAGCTGGCA

ATGTAGATGATTTTTTTCCAAGTAGTTGAACCTACCAATGTATTCCTTACCATATTGTCTTATTCTTATT

TCTATGTAACATCAATGCTCAAAAGACTTAAAATCTAAGTTACTCAAGTAGTTTTTATATTTGTCTGTTT

ATTTATTTACCTATATAATGTATATAATACGTTTGTGTGATGACAGAGGATCGATTCAGCACTTTGTAAA

AAAAATTCATAGTTTACATGCTACTCACTTGACGTATATGTAATTAATATTATTTTTAAAGTTTTATACT

AAAGAATGTCAAATCTTATATCTATATAAAGCAAAATCTCGGGCGTCTTACGTGGCGCTCTTTACGCTCT

TGAATCAAAATCCCTGCTCTCTGCACTTTTGGCTATTTCTTTTATATTTTCTCTCAGCTTGCCTAGCGGT

TATGTTCAGCCTGGGACTTGGACAGGCACTTCAGTTACCCGCTCCGTAACTGAGGTCTCTGTGCCTCCTC

AGAGATTTACTTATAAATGGCCTTCTCTGGACAATCTTTTCACACCGGGGAAAGCCGATCGGCATGAATA

TGACGGATTCAGCTAGAGGGTCAAAGCGGGATAAAGTCTTAGATTTGACAGAGGCAAGGAAAGGTGAGGA

AAATCCAGGCATCCAGCTGTATGCTCAAAAACAAACCTCCAGCGCAAGTGGAGACTGTTCTAAGTGACTG

CTCCTCAGCAGCAGTTCACCAACCTAAGCGCAAGAGCCATCGTTACATTCCACAGGGAGATCTTGTATGG

GCAATCAGTTCAGACACGATTCATTCTCTCGTGGGACTCTCTGAACTTTTCTTTGTACTTCCGCTTATGA

ATTCAGGTAAGCTGAATTATTATTGCTTGATAGAATGAATTGATAAGCTTTATGGTGCCAGCAAGTAGAA

AGCCAGGTTATATTCGCACAACGTCCTTTATCTGAGGCCACCAAGTCTCTACCTCTTTATACTGCTTTTC

CCAACTGATGTTTTTAAATAAGTTGTATAACTTTGTTGTTCATTCTATGACGATGTGCCTAAGTTTGTCT

GCTTATATATTGTTAGTTCGTAATCGTAATAAATTGTGATTACACATAGATATAGACTATTCTTGTAGAG

ATAAAGTTTAATGTTTTAAAGAATATCTTTTTATTTATAAGATTGTTTTCAGTTCTAGGCCTGTGAGCTT

TCAAGGATGTTCTTGCATTGGCGTCACTCGACAGGGCCAACAATTGAGCTGTTGAGAAACAAAAAAATAA

AAGAGGCCTTCGAAGATGGTTTTAGGAGTTGCGTAAAGATTTTTATGGCCATTGATCCGAAATATATTGG

GCAGACAAATTTGGCGAAGGGGTGGCCAAATGGATGAAAGAATTTTCTGTTATGGAGGCCGTTGTATTAC

TGAGAGAAATGCTCTGTGAAAGCGAGAGATAGAGCACACCTTTAAAATCCAAATACAAAAGATGATGTTT

GCTCAGAGAAAACAGAGGATGTACTTCAACTACTTTAACTTAAAAATTTATGAAATGTTTTGGGACTTCT

TTTAGTGTGAGCAGCCTTTGAGGCTTTGCAAATTGTAGGATAATGGTGGACAAAAATTGTCTTGTCTTGA

TTAAGATATTGTATAAGCTGC

>MSTRG.231.5 gene=MSTRG.231

CAATACTCGTAAGAAAGCCCAGGCCTTTTAGTTAACCCAATTTATTTTTAGGTATAAAATCACTCGTCGT

GTAGGTTAAGGCGGCAGCTTCTCAATTACATTAGGGCATCGCCTAGATTAGGGCATCGCCGCAACGATCT

ATGATAATCGTGTGTGTATGTATCTATTTGAGTGTAAAGTGCTGAATCGTTTCGTGATGCTATCTGTTTC

GAAGTTTAAAAACAGTAAACAATGAAGAAGATGCTCCAGAAAAACAAAACAATGATCTGCCTGATTGGGT

TGGCAAGGGAAAACAGTAGTTCTACCAAACCCGCCTCAAAGTGGACAACGAGAAATGCCAACATATTCGT

TGCGGAATTCCCACTTTGCTTGATATTTTGGTCATGCAGCTGATATGAGGCAAAAGAACCCAAAGGCTGG

CAACCCATTTAATGAAATTGCACCTATTAAATGGGAGATAATTTGTGACCACTGCGAGGGATTCCTTGTG

ACTCGGCCAACTGTGTGCGAGAGAACATCAGAAGAGCACACAATTGCTGAAGGCGAAAGCATTGGAAGCA

GCTATGAATGACATTAATAACTCATTTGGAAAAGGAGCTGTAACAAGATTAGGCAATGCTGGTGGATCTC

TTGTTGACTCTTTTCCAAGTGGTTGTCTGACATTGGACATTGCATTGGGTGTCGGCCTTCCTAGAGGAAA

TATTGTTGAGGATACGTATGGCTCTGAGATGGTGAAGTAAACAATGAAGATGATGCTCCAAAAAGATGGA

AAACAGTAACTAAGGCTGGCTAATCTGCTACTTATCTGCTATTAAGAAGCTGCTGCTTGTCTACTTGGTT

ATTTCGTTGAAGATAAAGCAACGCGACATATCGGGAACTTTTGAAATTGCAAGAACTAAGTTAAAAGCTG

GCAATGTAGATGATTTTTTTCCAAGTAGTTGAACCTACCAATGTATTCCTTACCATATTGTCTTATTCTT

ATTTCTATGTAACATCAATGCTCAAAAGACTTAAAATCTAAGTTACTCAAGTAGTTTTTATATTTGTCTG

TTTATTTATTTACCTATATAATGTATATAATACGTTTGTGTGATGACAGAGGATCGATTCAGCACTTTGT

AAAAAAAATTCATAGTTTACATGCTACTCACTTGACGTATATGTAATTAATATTATTTTTAAAGTTTTAT

ACTAAAGAATGTCAAATCTTATATCTATATAAAGCAAAATCTCGGGCGTCTTACGTGGCGCTCTTTACGC

TCTTGAATCAAAATCCCTGCTCTCTGCACTTTTGGCTATTTCTTTTATATTTTCTCTCAGCTTGCCTAGC

GGTTATGTTCAGCCTGGGACTTGGACAGGCACTTCAGTTACCCGCTCCGTAACTGAGGTCTCTGTGCCTC

CTCAGAGATTTACTTATAAATGGCCTTCTCTGGACAATCTTTTCACACCGGGGAAAGCCGATCGGCATGA

ATATGACGGATTCAGCTAGAGGGTCAAAGCGGGTATGTATGTTATTCTTGCTGATTTTCAGGGTTGATTT

ATGTTATATGATCATAGTCGTGGGCAGTTATCTCCTTCTGATTTATCCGGAGAGCATAGCAGCAGATTTT

TATGTATGCGCAGTTCGTTGTCTTTATCAATTTCTATGTGTATTTGAATTGGTGTCTGTGTTTCATTAGG

GTTTTGATGCTTAATTTATCGTGGATTTTGATTTTTTAATCGGTGATGATTAGGATAAAGTCTTAGATTT

GACAGAGGCAAGGAAAGGTGAGGAAAATCCAGGCATCCAGCTGTATGCTCAAAAACAAACCTCCAGCGCA

AGTGGAGACTGTTCTAAGTGACTGCTCCTCAGCAGCAGTTCACCAACCTAAGCGCAAGAGCCATCGTTAC

ATTCCACAGGGAGATCTTGTATGGGCAATCAGTTCAGACACGATTCATTCTCTCGTGGGACTCTCTGAAC

TTTTCTTTGTACTTCCGCTTATGAATTCAGGTAAGCTGAATTATTATTGCTTGATAGAATGAATTGATAA

GCTTTATGGTGCCAGCAAGTAGAAAGCCAGGTTATATTCGCACAACGTCCTTTATCTGAGGCCACCAAGT

CTCTACCTCTTTATACTGCTTTTCCCAACTGATGTTTTTAAATAAGTTGTATAACTTTGTTGTTCATTCT

ATGACGATGTGCCTAAGTTTGTCTGCTTATATATTGTTAGTTCGTAATCGTAATAAATTGTGATTACACA

TAGATATAGACTATTCTTGTAGAGATAAAGTTTAATGTTTTAAAGAATATCTTTTTATTTATAAGATTGT

TTTCAGTTCTAGGCCTGTGAGCTTTCAAGGATGTTCTTGCATTGGCGTCACTCGACAGGGCCAACAATTG

AGCTGTTGAGAAACAAAAAAATAAAAGAGGCCTTCGAAGATGGTTTTAGGAGTTGCGTAAAGATTTTTAT

GGCCATTGATCCGAAATATATTGGGCAGACAAATTTGGCGAAGGGGTGGCCAAATGGATGAAAGAATTTT

CTGTTATGGAGGCCGTTGTATTACTGAGAGAAATGCTCTGTGAAAGCGAGAGATAGAGCACACCTTTAAA

ATCCAAATACAAAAGATGATGTTTGCTCAGAGAAAACAGAGGATGTACTTCAACTACTTTAACTTAAAAA

TTTATGAAATGTTTTGGGACTTCTTTTAGTGTGAGCAGCCTTTGAGGCTTTGCAAATTGTAGGATAATGG

TGGACAAAAATTGTCTTGTCTTGATTAAGATATTGTATAAGCTGC

>MSTRG.231.9 gene=MSTRG.231

CAGGCCTTTTAGTTAACCCAATTTATTTTTAGGTATAAAATCACTCGTCGTGTAGGTTAAGGCGGCAGCT

TCTCAATTACATTAGGGCATCGCCTAGATTAGGGCATCGCCGCAACGATCTATGATAATCGTGTGTGTAT

GTATCTATTTGAGTGTAAAGTGCTGAATCGTTTCGTGATGCTATCTGTTTCGAAGTTTAAAAACAGTAAA

CAATGAAGAAGATGCTCCAGAAAAACAAAACAATGATCTGCCTGATTGGGTTGGCAAGGGAAAACAGTAG

TTCTACCAAACCCGCCTCAAAGTGGACAACGAGAAATGCCAACATATTCGTTGCGGAATTCCCACTTTGC

TTGATATTTTGGTCATGCAGCTGATATGAGGCAAAAGAACCCAAAGGCTGGCAACCCATTTAATGAAATT

GCACCTATTAAATGGGAGATAATTTGTGACCACTGCGAGGGATTCCTTGTGACTCGGCCAACTGTGTGCG

AGAGAACATCAGAAGAGCACACAATTGCTGAAGGCGAAAGCATTGGAAGCAGCTATGAATGACATTAATA

ACTCATTTGGAAAAGGAGCTGTAACAAGATTAGGCAATGCTGGTGGATCTCTTGTGTATGTTGACTCTTT

TCCAAGTGGTTGTCTGACATTGGACATTGCATTGGGTGTCGGCCTTCCTAGAGGAAATATTGTTGAGGTA

TGTTTTCGTACACACTTTGAGGTATTGTAAAACAAACTCAGTACAATCTTTTTCACGTGAAGATTATCTT

GTTTAATATCCAGGATACGTATGGCTCTGAGATGGTGAAGTAAACAATGAAGATGATGCTCCAAAAAGAT

GGAAAACAGTAACTAAGGCTGGCTAATCTGCTACTTATCTGCTATTAAGAAGCTGCTGCTTGTCTACTTG

GTTATTTCGTTGAAGATAAAGCAACGCGACATATCGGGAACTTTTGAAATTGCAAGAACTAAGTTAAAAG

CTGGCAATGTAGATGATTTTTTTCCAAGTAGTTGAACCTACCAATGTATTCCTTACCATATTGTCTTATT

CTTATTTCTATGTAACATCAATGCTCAAAAGACTTAAAATCTAAGTTACTCAAGTAGTTTTTATATTTGT

CTGTTTATTTATTTACCTATATAATGTATATAATACGTTTGTGTGATGACAGAGGATCGATTCAGCACTT

TGTAAAAAAAATTCATAGTTTACATGCTACTCACTTGACGTATATGTAATTAATATTATTTTTAAAGTTT

TATACTAAAGAATGTCAAATCTTATATCTATATAAAGCAAAATCTCGGGCGTCTTACGTGGCGCTCTTTA

CGCTCTTGAATCAAAATCCCTGCTCTCTGCACTTTTGGCTATTTCTTTTATATTTTCTCTCAGCTTGCCT

AGCGGTTATGTTCAGCCTGGGACTTGGACAGGCACTTCAGTTACCCGCTCCGTAACTGAGGTCTCTGTGC

CTCCTCAGAGATTTACTTATAAATGGCCTTCTCTGGACAATCTTTTCACACCGGGGAAAGCCGATCGGCA

TGAATATGACGGATTCAGCTAGAGGGTCAAAGCGGGTATGTATGTTATTCTTGCTGATTTTCAGGGTTGA

TTTATGTTATATGATCATAGTCGTGGGCAGTTATCTCCTTCTGATTTATCCGGAGAGCATAGCAGCAGAT

TTTTATGTATGCGCAGTTCGTTGTCTTTATCAATTTCTATGTGTATTTGAATTGGTGTCTGTGTTTCATT

AGGGTTTTGATGCTTAATTTATCGTGGATTTTGATTTTTTAATCGGTGATGATTAGGATAAAGTCTTAGA

TTTGACAGAGGCAAGGAAAGGTGAGGAAAATCCAGGCATCCAGCTGTATGCTCAAAAACAAACCTCCAGC

GCAAGTGGAGACTGTTCTAAGTGACTGCTCCTCAGCAGCAGTTCACCAACCTAAGCGCAAGAGCCATCGT

TACATTCCACAGGGAGATCTTGTATGGGCAATCAGTTCAGACACGATTCATTCTCTCGTGGGACTCTCTG

AACTTTTCTTTGTACTTCCGCTTATGAATTCAGGTAAGCTGAATTATTATTGCTTGATAGAATGAATTGA

TAAGCTTCTTTATCCTTGCTATATGCTATGGTAACTCTCTCCACATATGCACACAGCCACACACTTAACT

AACTTCGTATCAGACTCTTTGAGGCATTTAATTTTTGCATCAACTAGCTACTATTGCTCTACCTGAT

>MSTRG.231.10 gene=MSTRG.231

AAAGCCCAGGCCTTTTAGTTAACCCAATTTATTTTTAGGTATAAAATCACTCGTCGTGTAGGTTAAGGCG

GCAGCTTCTCAATTACATTAGGGCATCGCCTAGATTAGGGCATCGCCGCAACGATCTATGATAATCGTGT

GTGTATGTATCTAGAGTGTAAAGTGCTGAATCGTTTCGTGATGCTATCTGTTTCGAAGTCTAAAAACAGT

AAACAATGAAGAAGATGCTCCAGAAAAACAAAACAATGATCTGCCTGATTGGGTTGGCAAGGGAAAACAG

TAGTTCTACCAAACCCGCCTCAAAGTGGACAACGAGAAATGCCAACATATTCGTTGCGGAATTCCCACTT

TGCTTGATATTTTGGTCATGCAGCTGATATGAGGCAAAAGAACCCAAAGGCTGGCAACCCATTTAATGAA

ATTGCACCTATTAAATGGGAGATAATTTGTGACCACTGCGAGGGATTCCTTGTGACTCGGCCAACTGTGT

GCGAGAGAACATCAGAAGAGCACACAATTGCTGAAGGCGAAAGCATTGGAAGCAGCTATGAATGACATTA

ATAACTCATTTGGAAAAGGAGCTGTAACAAGATTAGGCAATGCTGGTGGATCTCTTGTTGACTCTTTTCC

AAGTGGTTGTCTGACATTGGACATTGCATTGGGTGTCGGCCTTCCTAGAGGAAATATTGTTGAGGTATGT

TTTCGTACACACTTTGAGGTATTGTAAAACAAACTCAGTACAATCTTTTTCACGTGAAGATTATCTTGTT

TAATATCCAGGATACGTATGGCTCTGAGATGGTGAAGTAAACAATGAAGATGATGCTCCAAAAAGATGGA

AAACAGTAACTAAGGCTGGCTAATCTGCTACTTATCTGCTATTAAGAAGCTGCTGCTTGTCTACTTGGTT

ATTTCGTTGAAGATAAAGCAACGCGACATATCGGGAACTTTTGAAATTGCAAGAACTAAGTTAAAAGCTG

GCAATGTAGATGATTTTTTTCCAAGTAGTTGAACCTACCAATGTATTCCTTACCATATTGTCTTATTCTT

ATTTCTATGTAACATCAATGCTCAAAAGACTTAAAATCTAAGTTACTCAAGTAGTTTTTATATTTGTCTG

TTTATTTATTTACCTATATAATGTATATAATACGTTTGTGTGATGACAGAGGATCGATTCAGCACTTTGT

AAAAAAAATTCATAGTTTAC

>MSTRG.231.12 gene=MSTRG.231

GCCTTTTAGTTAACCCAATTTATTTTTAGGTATAAAATCACTCGTCGTGTAGGTTAAGGCGGCAGCTTCT

CAATTACATTAGGGCATCGCCTAGATTAGGGCATCGCCGCAACGATCTATGATAATCGTGTGTGTATGTA

TCTAGAGTGTAAAGTGCTGAATCGTTTCGTGATGCTATCTGTTTCGAAGTCTAAAAACAGTAAACAATGA

AGAAGATGCTCCAGAAAAACAAAACAATGATCTGCCTGATTGGGTTGGCAAGGGAAAACAGTAGTTCTAC

CAAACCCGCCTCAAAGTGGACAACGAGAAATGCCAACATTTTCGTTGCGGAATTCCCACTTTGCTTGATA

TTTTGGTCATGCAGCTGATATGAGGCAAAAGAACCCAAAGGCTGGCAACCCATTTAATGAAATTGCACCT

ATTAAATGGGAGATAATTTGTGACCACTGCGAGGGATTCCTTGTGACTCGGCCAACTGTGTGCGAGAGAA

CATCAGAAGAGCACACAATTGCTGAAGGCGAAAGCATTGGAAGCAGCTATGAATGACATTAATAACTCAT

TTGGAAAAGGAGCTGTAACAAGATTAGGCAATGCTGGTGGATCTCTTGTGTATGTTGACTCTTTTCCAAG

TGGTTGTCTGACATTGGACATTGCATTGGGTGTCGGCCTTCCTAGAGGAAATATTGTTGAGGTATGTTTT

CGTACACACTTTGAGGTATTGTAAAACAAACTCAGTACAATCTTTTTCACGTGAAGATTATCTTGTTTAA

TATCCAGGATACGTATGGCTCTGAGATGGTGAAGTAAACAATGAAGATGATGCTCCAAAAAGATGGAAAA

CAGTAACTAAGGCTGGCTAATCTGCTACTTATCTGCTATTAAGAAGCTGCTGCTTGTCTACTTGGTTATT

TCGTTGAAGATAAAGCAACGCGACATATCGGGAACTTTTGAAATTGCAAGAACTAAGTTAAAAGCTGGCA

ATGTAGATGATTTTTTTCCAAGTAGTTGAACCTACCAATGTATTCCTTACCATATTGTCTTATTCTTATT

TCTATGTAACATCAATGCTCAAAAGACTTAAAATCTAAGTTACTCAAGTAGTTTTTATATTTGTCTGTTT

ATTTATTTACCTATATAATGTATATAATACGTTTGTGTGATGACAGAGGATCGATTCAGCAC

>MSTRG.231.11 gene=MSTRG.231

AGGCCTTTTAGTTAACCCAATTTATTTTTAGGTATAAAATCACTCGTCGTGTAGGTTAAGGCGGCAGCTT

CTCAATTACATTAGGGCATCGCCTAGATTAGGGCATCGCCGCAACGATCTATGATAATCGTGTGTGTATG

TATCTAGAGTGTAAAGTGCTGAATCGTTTCGTGATGCTATCTGTTTCGAAGTCTAAAAACAGTAAACAAT

GAAGAAGATGCTCCAGAAAAACAAAACAATGATCTGCCTGATTGGGTTGGCAAGGGAAAACAGTAGTTCT

ACCAAACCCGCCTCAAAGTGGACAACGAGAAATGCCAACATATTCGTTGCGGAATTCCCACTTTGCTTGA

TATTTTGGTCATGCAGCTGATATGAGGCAAAAGAACCCAAAGGCTGGCAACCCATTTAATGAAATTGCAC

CTATTAAATGGGAGATAATTTGTGACCACTGCGAGGGATTCCTTGTGACTCGGCCAACTGTGTGCGAGAG

AACATCAGAAGAGCACACAATTGCTGAAGGCGAAAGCATTGGAAGCAGCTATGAATGACATTAATAACTC

ATTTGGAAAAGGAGCTGTAACAAGATTAGGCAATGCTGGTGGATCTCTTGTGTATGTTGACTCTTTTCCA

AGTGGTTGTCTGACATTGGACATTGCATTGGGTGTCGGCCTTCCTAGAGGAAATATTGTTGAGGATACGT

ATGGCTCTGAGATGGTGAAGTAAACAATGAAGATGATGCTCCAAAAAGATGGAAAACAGTAACTAAGGCT

GGCTAATCTGCTACTTATCTGCTATTAAGAAGCTGCTGCTTGTCTACTTGGTTATTTCGTTGAAGATAAA

GCAACGCGACATATCGGGAACTTTTGAAATTGCAAGAACTAAGTTAAAAGCTGGCAATGTAGATGATTTT

TTTCCAAGTAGTTGAACCTACCAATGTATTCCTTACCATATTGTCTTATTCTTATTTCTATGTAACATCA

ATGCTCAAAAGACTTAAAATCTAAGTTACTCAAGTAGTTTTTATATTTGTCTGTTTATTTATTTACCTAT

ATAATGTATATAATACGTTTGTGTGATGACAGAGGATCGATTCAGCAC

>MSTRG.231.13 gene=MSTRG.231

CAGGCCTTTTAGTTAACCCAATTTATTTTTAGGTATAAAATCACTCGTCGTGTAGGTTAAGGCGGCAGCT

TCTCAATTACATTAGGGCATCGCCTAGATTAGGGCATCGCCGCAACGATCTATGATAATCGTGTGTGTAT

GTATCTAGAGTGTAAAGTGCTGAATCGTTTCGTGATGCTATCTGTTTCGAAGTCTAAAAACAGTAAACAA

TGAAGAAGATGCTCCAGAAAAACAAAACAATGATCTGCCTGATTGGGTTGGCAAGGGAAAACAGTAGTTC

TACCAAACCCGCCTCAAAGTGGACAACGAGAAATGCCAACATATTCGTTGCGGAATTCCCACTTTGCTTG

ATATTTTGGTCATGCAGCTGATATGAGGCAAAAGAACCCAAAGGCTGGCAACCCATTTAATGAAATTGCA

CCTATTAAATGGGAGATAATTTGTGACCACTGCGAGGGATTCCTTGTGACTCGGCCAACTGTGTGCGAGA

GAACATCAGAAGAGCACACAATTGCTGAAGGCGAAAGCATTGGAAGCAGCTATGAATGACATTAATAACT

CATTTGGAAAAGGAGCTGTAACAAGATTAGGCAATGCTGGTGGATCTCTTGTTGACTCTTTTCCAAGTGG

TTGTCTGACATTGGACATTGCATTGGGTGTCGGCCTTCCTAGAGGAAATATTGTTGAGGATACGTATGGC

TCTGAGATGGTGAAGTAAACAATGAAGATGATGCTCCAAAAAGATGGAAAACAGTAACTAAGGCTGGCTA

ATCTGCTACTTATCTGCTATTAAGAAGCTGCTGCTTGTCTACTTGGTTATTTCGTTGAAGATAAAGCAAC

GCGACATATCGGGAACTTTTGAAATTGCAAGAACTAAGTTAAAAGCTGGCAATGTAGATGATTTTTTTCC

AAGTAGTTGAACCTACCAATGTATTCCTTACCATATTGTCTTATTCTTATTTCTATGTAACATCAATGCT

CAAAAGACTTAAAATCTAAGTTACTCAAGTAGTTTTTATATTTGTCTGTTTATTTATTTACCTATATAAT

GTATATAATACGTTTGTGTGATGACAGAG

>MSTRG.231.14 gene=MSTRG.231

CCCATACCTGTATATCATTTTATTTCTAAAAAGACATGATAAGATTGTTGTTAGGAATTAATCTAATGTC

CATATTTGATGTAAATATTTTGTTACAGGCGAAAGCATTGGAAGCAGCTATGAATGACATTAATAACTCA

TTTGGAAAAGGAGCTGTAACAAGATTAGGCAATGCTGGTGGATCTCTTGTGTATGTTGACTCTTTTCCAA

GTGGTTGTCTGACATTGGACATTGCATTGGGTGTCGGCCTTCCTAGAGGAAATATTGTTGAGGATACGTA

TGGCTCTGAGATGGTGAAGTAAACAATGAAGATGATGCTCCAAAAAGATGGAAAACAGTAACTAAGGCTG

GCTAATCTGCTACTTATCTGCTATTAAGAAGCTGCTGCTTGTCTACTTGGTTATTTCGTTGAAGATAAAG

CAACGCGACATATCGGGAACTTTTGAAATTGCAAGAACTAAGTTAAAAGCTGGCAATGTAGATGATTTTT

TTCCAAGTAGTTGAACCTACCAATGTATTCCTTACCATATTGTCTTATTCTTATTTCTATGTAACATCAA

TGCTCAAAAGACTTA

>MSTRG.232.1 gene=MSTRG.232

AAGCTAAATGAGTGTCAGAGATACCATCATATCTCAGTAAATCAGTGTTTGTCGAGCACAAATTGTTCTT

GCCGATGATTAGTTGCCCAAAAATACTGTAAGACATACTGTAAAAAGAATTCAAGAGGCCGATAACAGCA

GCGGAGATAATAAAGGCCGTGATTCCCAGTTGCAAGGTCTGCAATTTTTTCTGTCTTCGAGTCTTTGGAA

TGTTGCTGTTATGGAATGCCCAAGGAAAATATAACTTCGGAATCAANCGAGTCTTTGGAATGTTGCTGTC

ATGGAATGCCCGAGGGAAATATAACTTCGGAATCAAGAAGTTTCGAAAGGTAGCTTGGCAAATCAGCCCA

ACCAAAGCTGCTCAGAAGTACAACTTAGATACAAGTTGTTTTTTCAAATATGTGTGAGTCCTTGTATTGA

TATCTATCAGGCTAACGAGTGATGCCACTATGGATATGATCTTCTAGCACGTATGTATATTAATGTGTTA

ACTGTACCAATTGTTAGTATTGGTGTCCTAGAGACAATACTATTGTGTTCTATCGTAGACATTTATGGAT

TATGTTATATTGATTAT

>MSTRG.233.1 gene=MSTRG.233

GTTCCGCATTCTATAGTAAGGTATTCATTATCGAACTGATACCCGACTTCCCAAATATTGAACTCTCAGG

TGTCAGAGATACCATCATATCTCAGTAAATCAGTGTTTGTCGAGCACAAATTGTTCTTGCCGATGATTAG

TTGCCCAAAAATACTGTAAGACATACTGTAAAAAGAATTCAAGAGGCCGATAACAGCAGCGGAGATAATA

AAGGCCGTGATTCCCAGTTGCAAGGTCTGCATTTTTTCTGTCTTCGAGTCTTTGGAATGTTGCTGTCATG

GAATGCCCGAGGGAAATATAACTTCGGAATCAAGAAGTTTCGAAAGGTAGCTTGGCAAATC

>MSTRG.232.2 gene=MSTRG.232

GTGTTGAAAAGGTGAAACCTCCAACCGGTAGCCCAATATCTATGTTAATGGCTACTCCAGATGGCTCATA

TGCATTACCAAGCGGTTGATGTTCGTAAGCTAAATGAGTGTCAGAGATACCATCATATCTCAGTAAATCA

GTGTTTGTCGAGCACAAATTGTTCTTGCCGATGATTAGTTGCCCAAAAATACTGTAAGACATACTGTAAA

AAGAATTCAAGAGGCCGATAACAGCAGCGGAGATAATAAAGGCCGTGATTCCCAGTTGCAAGGTCTGCAA

TTTTTTCTGTCTTCGAGTCTTTGGAATGTTGCTGTTATGGAATGCCCAAGGAAAATATAACTTCGGAATC

AANCGAGTCTTTGGAATGTTGCTGTCATGGAATGCCCGAGGGAAATATAACTTCGGAATCAAGAAGTTTC

GAAAGGTAGCTTGGCAAATCAGCCCAACCAAAGCTGCTCAGAAGTACAACTTAGATACAAGTTGTTTTTT

CAAATATGTGTGAGTCCTTGTATTGATATCTATCAGGCTAACGAGTGATGCCACTATGGATATGATCTTC

TAGCACGTATGTATATTAATGTGTTAACTGTACCAAATATTTATCATTTAATATTTTTATTCACGGCAAT

GTTGGTTTTAACCGATATTAAGAAAATGAAA

>MSTRG.234.1 gene=MSTRG.234

CATCCATGGCGTTACTCAGCAACGCTCTCCGGCAAGCCTTCATGCCGAAGCACGAGTACGACAATCTGCG

CGAAGAAGACAAAGCGATGATCCAATTACAACGCCCCGTCTTGATTTCCCTCTTGTTGTGTATCGTGATT

GTGATCGTTGTGTCCACCTCGATTAGCGTGAAGATTGTGTTCCCGGCGGAGGACGGGAAACGCGTGTTTT

GCCGGGATTTGAGGATTCAGCCGTTGTCGATAAATGTGAGCTCTGGTGGCGGTGGCGGCGGGGAGGATGT

GTTTCCTGGGGCGTTTTATTTGACGGATCAGCAGACGGTGGATTATTATTGGATGGTGGTGTTTATGCC

>MSTRG.235.1 gene=MSTRG.235

TTTTTTTATTCAGTGTAGATATTATTTTTTTGCATCTCAATATACGTGTTAAAAAGTCAAAGTCGGTTAA

AATGTAACCGGGACAGAGGGAATATTAAACTTTCTGTCGTAGTATATTGTATAGTTTGGTTGAAAGTTAT

TAGTTTGTGATATATCTTTACTTCTTTTTAAAGGTATGTTTTCGTACACACTTTGAGGTATTGTAAAACA

AACTCAGTACAATCTTTTTCACGTGAAGATTATCTTGTTTAATATCCAGGATACGTATGGCTCTGAGATG

GTGAAGTAAACAATGAAGATGATGCTCCAAAAAGATGGAAAACAGTAACTAAGGCTGGCTAATCTGCTAC

TTATCTGCTATTAAGAAGCTGCTGCTTGTCTACTTGGTTATTTCGTTGAAGATAAAGCAACGCGATGTAT

TCCTTACCATATTGTCTTATTCTTATTTCTATGTAACATCAATGCTCAAAAGACTTAAAATCTAAGTTAC

TCAAGTAGTTTTTATATTTGTCTGTTTATTTATTTACCTATATAATGTATATAATACGTTTGTGTGATGA

CAGAGGATCGATTCAGCACTTTGTAAAAAAAATTCATAGTTTACATGCTACTCACTTGACGTATATGTAA

TTAATATTATTTTTAAAATTTTATACTAAAGAATGTCAAATCTTATATCTATATAAAGCAAAATCTCGGG

CGTCTTACGTGGCGCTCTTTACGCTCTTGAATCAAAATCCCTGCTCTCTGCACTTTTGGCTATTTCTTTT

ATATTTTCTCTCAGCTTGCCTAGCGGTTATGTTCAGCCTGGGACTTGGACAGGCACTTCAGTTACCCGCT

CCGTAACTGAGGTCTCTGTGCCTCCTCAGAGATTTACTTATAAATGGCCTTCTCTGGACAATCTTTTCAC

ACCGGGGAAAGCCGATCGGCATGAATATGACGGATTCAGCTAGAGGGTCAAAGCGGGATAAAGTCTTAGA

TTTGACAGAGGCAAGGAAAGGTGAGGAAAATCCAGGCATCCAGCTGTATGCTCAAAAACAAACCTCCAGC

GCAAGTGGAGACTGTTCTAAGTGACTGCTCCTCAGCAGCAGTTCACCAACCTAAGCGCAAGAGCCATCGT

TACATTCCACAGGGAGATCTTGTATGGGCAATCAGTTCAGACACGATTCATTCTCTCGTGGGACTCTCTG

AACTTTTCTTTGTACT

>MSTRG.235.2 gene=MSTRG.235

CTATTATTTGGTATAAGAATTGAATTTTTTTATTCAGTGTAGATATTATTTTTTTGCATCTCAATATACG

TGTTAAAAAGTCAAAGTCGGTTAAAATGTAACCGGGACAGAGGGAATATTAAACTTTCTGTCGTAGTATA

TTGTATAGTTTGGTTGAAAGTTATTAGTTTGTGATATATCTTTACTTCTTTTTAAAGGTATGTTTTCGTA

CACACTTTGAGGTATTGTAAAACAAACTCAGTACAATCTTTTTCACGTGAAGATTATCTTGTTTAATATC

CAGGATACGTATGGCTCTGAGATGGTGAAGGTATGCTTGTTATATAGTTATAATGTACTTGAAAATCTAT

TAAGTTTTCTAGTATTCAATTCACTGGTTTTCAAATTTCTTCTTGCAGTAAACAATGAAGATGATGCTCC

AAAAAGATGGAAAACAGTAACTAAGGCTGGCTAATCTGCTACTTATCTGCTATTAAGAAGCTGCTGCTTG

TCTACTTGGTTATTTCGTTGAAGATAAAGCAACGCGACATATCGGGAACTTTTGAAATTGCAAGAACTAA

GTTAAAAGCTGGCAATGTAGATGATTTTTTTCCAAGTAGTTGAACCTACCAATGTATTCCTTACCATATT

GTCTTATTCTTATTTCTATGTAACATCAATGCTCAAAAGACTTAAAATCTAAGTTACTCAAGTAGTTTTT

ATATTTGTCTGTTTATTTATTTACCTATATAATGTATATAATACGTTTGTGTGATGACAGAGGATCGATT

CAGCACTTTGTAAAAAAAATTCATAGTTTACATGCTACTCACTTGACGTATATGTAATTAATATTATTTT

TAAAATTTTATACTAAAGAATGTCAAATCTTATATCTATATAAAGCAAAATCTCGGGCGTCTTACGTGGC

GCTCTTTACGCTCTTGAATCAAAATCCCTGCTCTCTGCACTTTTGGCTATTTCTTTTATATTTTCTCTCA

GCTTGCCTAGCGGTTATGTTCAGCCTGGGACTTGGACAGGCACTTCAGTTACCCGCTCCGTAACTGAGGT

CTCTGTGCCTCCTCAGAGATTTACTTATAAATGGCCTTCTCTGGACAATCTTTTCACACCGGGGAAAGCC

GATCGGCATGAATATGACGGATTCAGCTAGAGGGTCAAAGCGGGTATGTATGTTATTCTTGCTGATTTTC

AGGGTTGATTTATGTTATATGATCATAGTCGTGGGCAGTTATCTCCTTCTGATTTATCCGGAGAGCATAG

CAGCAGATTTTTATGTATGCGCAGTTCGTTGTCTTTATCAATTTCTATGTGTATTTGAATTGGTGTCTGT

GTTTCATTAGGGTTTTGATGCTTAATTTATCGTGGATTTTGATTTTTTAATCGGTGATGATTAGGATAAA

GTCTTAGATTTGACAGAGGCAAGGAAAGGTGAGGAAAATCCAGGCATCCAGCTGTATGCTCAAAAACAAA

CCTCCAGCGCAAGTGGAGACTGTTCTAAGTGACTGCTCCTCAGCAGCAGTTCACCAACCTAAGCGCAAGA

GCCATCGTTACATTCCACAGGGAGATCTTGTATGGGCAATCAGTTCAGACACGATTCATTCTCTCGTGGG

ACTCTCT

>MSTRG.235.3 gene=MSTRG.235

CATTACTCGTAAGAAAGCCCAGGCCTTTTAGTTAACCCAATTTATTTTTAGGTATAAAATCACTCGTCGT

GTAGGTTAAGGCGGCAGCTTCTCAATTACATTAGGGCATCGCCTAGATTAGGGCATCGCCGCAACGATCT

ATGATAATCGTGTGTGTATGTATCTAGAGTGTAAAGTGCTGAATCGTTTCGTGATGCTATCTGTTTCGAA

GTCTAAAAACAGTAAACAATGAAGAAGATGCTCCAGAAAAACAAAACAATGATCTGCCTGATTGGGTTGG

CAAGGGAAAACAGTAGTTCTACCAAACCCGCCTCAAAGTGGACAACGAGAAATGCCAACATTTTCGTTGC

GGAATTCCCACTTTGCTTGATATTTTGGTCATGCAGCTGATATGAGGCAAAAGAACCCAAAGGCTGGCAA

CCCATTTAATGAAATTGCACCTATTAAATGGGAGATAATTTGTGACCACTGCGAGGGATTCCTTGTGACT

CGGCCAACTGTGTGCGAGAGAACATCAGAAGAGCACACAATTGCTGAAGGTATGTTTTCGTACACACTTT

GAGGTATTGTAAAACAAACTCAGTACAATCTTTTTCACGTGAAGATTATCTTGTTTAATATCCAGGATAC

GTATGGCTCTGAGATGGTGAAGTAAACAATGAAGATGATGCTCCAAAAAGATGGAAAACAGTAACTAAGG

CTGGCTAATCTGCTACTTATCTGCTATTAAGAAGCTGCTGCTTGTCTACTTGGTTATTTCGTTGAAGATA

AAGCAACGCGACATATCGGGAACTTTTGAAATTGCAAGAACTAAGTTAAAAGCTGGCAATGTAGATGATT

TTTTTCCAAGTAGTTGAACCTACCAATGTATTCCTTACCATATTGTCTTATTCTTATTTCTATGTAACAT

CAATGCTCAAAAGACTTAAAATCTAAGTTACTCAAGTAGTTTTTATATTTGTCTGTTTATTTATTTACCT

ATATAATGTATATAATACGTTTGTGTGATGACAGAGGATCGATTCAG

>MSTRG.236.1 gene=MSTRG.236

CCGCACAATCCATCTTCATTCAGGTTCAGGCCCAAGCCCAGTTAGTCATTGTTTTCTCCGCTGCTTGTGT

GTATATGTACGTATACGTGTGAGATGAGGATGAATCAATGGCGATTCCAAAGATTTAAGATCAATTCTCA

CACTCTACATTGTTATGATACTTGTTTCTATCGCTTCCACTCTCAAATTTTAAACAATGAAGAAAGTGCT

TCAGGAAGCCGATAAGAATGGTCGAAGAAAGCGCTTAATTAATAAATAAGGTAGTGGCATCAATGGACTG

GCCTGAGGTCACCACATACAGAGAACTCTACACACATGGTCAGTGAGAGGAAATATTAAGGAATCTCTAC

TGCACTTGTGAAACTGAAAGGGGTGTTGTTGCTAGTGGCATGACCAGGGACTATTTGATGGCATTCTACC

AGCAAACAGAACTCCGACCTAAACGGATCATATTCTACAGGGATGGAGTCAGTGAAGGTCAGTTTAATCA

GGTCTTGCTGAAAGAGATGGATGCCATTAGGACGGTTGATGTGTTTTATTTTTTGTCTGCCAGGCGCTGC

AATCAAATTATTTACAATGGCAGGAAATCAACTGATCGGAGTGGCAACATATCACCTGGTACTGTTGTAG

ATATGACCATCTGCCATCCCACTGAGTTTGACTTTTATTTGTGTAGCCATGCTGGTATACAGGACGGACT

TTGACTTTGTTGTCGGCAATGCACAGACTAATGAGGGTGTTGAAAAGGTGAAACCTCCAACCGGTAGCCC

AATATCTATGTTAATGGCTACTCCAGATGGCTCATATGCATTACCAAGCAGTTGATGTTTTTAAGCTAAA

TGAGTGTCAGAGATACCATCATATCTCAGTAAATCAGTGTTTGTCGAGCACAAAATGTTCTTGCCGATGA

TTAGTTGCCCAAAAATATTGTAAGACATACTGTAAAAAGAATTCAAGAGGCCCATAACAGCAGCGGAGAT

AATAAAGGCCGTGATTCCCAGTTGCAAGGTCTGCATTTTTTCTGTCTTTGGAATGTTGCTGTCATGGAAT

GCCCGAGGAAAATATAACTTCGGAATCAAGAAGTTTCGAAAGGTAGCTTCGCAAATCAGCCCAACCAAAG

CTGCTCAGAAGTACAACTTAGATGCAAGTTTTTTTTTCAAATATGTGTGAGTCCTTGTATTGATATCTAT

CAGGCTAACGAGTGATGCCACTATGGATATGATCTTCTAGTGAAATTTGTATATACATTCATTTATGCAC

GTATGTATATTGTGTTAACTGTGCCAAATATTTATCATTTAATATTTTTATTCACGGCAA

>MSTRG.236.7 gene=MSTRG.236

CCGCACAATCCATCTTCATTCAGGTTCAGGCCCAAGCCCAGTTAGTCATTGTTTTCTCCGCTGCTTGTGT

GTATATGTACGTATACGTGTGAGATGAGGATGAATCAATGGCGATTCCAAAGATTTAAGATCAATTCTCA

CACTCTACATTGTTATGATACTTGTTTCTATCGCTTCCACTCTCAAATTTTAAACAATGAAGAAAGTGCT

TCAGGAAGCCGATAAGAATGGTCGAAGAAAGCGCTTAATTAATAAATAAGGTAGTGGCATCAATGGACTG

GCCTGAGGTCACCACATACAGAGAACTCTACACACATGGTCAGTGAGAGGAAATATTAAGGAATCTCTAC

TGCACTTGTGAAACTGAAAGGGGTGTTGTTGCTAGTGGCATGACCAGGGACTATTTGATGGCATTCTACC

AGCAAACAGAACTCCGACCTAAACGGATCATATTCTACAGGGATGGAGTCAGTGAAGGTCAGTTTAATCA

GGTCTTGCTGAAAGAGATGGATGCCATTAGGACGGTTGATGTGTTTTATTTTTTGTCTGCCAGGCGCTGC

AATCAAATTATTTACAATGGCAGGAAATCAACTGATCGGAGTGGCAACATATCACCTGGTTACATATTGT

TCTATTGTATCTTTAAAAATCTTTATTATTCTGATTAGCAAATAACTGGCTTTTGTATACCAGGTACTGT

TGTAGATATGACCATCTGCCATCCCACTGAGTTTGACTTTTATTTGTGTAGCCATGCTGGTATACAGGGT

GTTGAAAAGGTGAAACCTCCAACCGGTAGCCCAATATCTATGTTAATGGCTACTCCAGATGGCTCATATG

CATTACCAAGCAGTTGATGTTTTTAAGCTAAATGAGTGTCAGAGATACCATCATATCTCAGTAAATCAGT

GTTTGTCGAGCACAAAATGTTCTTGCCGATGATTAGTTGCCCAAAAATATTGTAAGACATACTGTAAAAA

GAATTCAAGAGGCCCATAACAGCAGCGGAGATAATAAAGGCCGTGATTCCCAGTTGCAAGGTCTGCATTT

TTTCTGTCTTTGGAATGTTGCTGTCATGGAATGCCCGAGGAAAATATAACTTCGGAATCAAGAAGTTTCG

AAAGGTAGCTTCGCAAATCAGCCCAACCAAAGCTGCTCAGAAGTACAACTTAGATGCAAGTTTTTTTTTC

AAATATGTGTGAGTCCTTGTATTGATATCTATCAGGCTAACGAGTGATGCCACTATGGATATGATCTTCT

AGTGAAATTTGTATATACATTCATTTATGCACGTATGTATATTGTGTTAACTGTGCCAAATATTTATCAT

TTAATATTTTTATTCACGGCAA

>MSTRG.236.2 gene=MSTRG.236

AAAATGCCATCTGTACCCGCACAGTCCATCTTCATTCAGCTTCAGGCCCAAGCCCAGTTAGTCATTGTTT

TCTCCGCTGCCTGTGTGTATATGTACGTATACGTGTGAGATGAGGAAGAATCAATGGCGATTCCAAAGAA

TTAAGATCAATTCTCACACTCTGCATTGTTATGATAGTTGTTTCTATTGCTTTTACTCTCAAATTTTAAA

CAATGAAGAAAATGCTTCAGAAAGCCGATAAGAATGGTCGAAGAAAGCGCTTAATTAATAAATAAGGTAG

TGGCATCAATGGACTGGCCCGAGGTCACCACATACAAAGAACTCTACACACATGGTCAGTGAGAGGAAAT

ATTAAGGAATCTCTACTGCACTTGTGAAACTGAAAGGGGTGTTGTTGCTAGTGGCATGACCAGGGACTAT

TTGATGGCATTCTACCAGCAAACAGAACTCCGACCTAAACGGATCATATTCTACAGGGATGGAGTCAGTG

AAGGTCAGTTTAATCAGGTCTTGCTGAAAGAGATGGATGCCATTAGGACGGTTGATGTGTTTTATTTTTT

GTCTGCCAGGCGCTGCAATCAAATTATTTACAATGGCAGGAAATCAACTGATCGGAGTGGCAACATATCA

CCTGGTTACATATTGTTCTATTGTATCTTTAAAAATCTTTATTATTCTGATTAGCAAATAACTGGCTTTT

GTATACCAGGTACTGTTGTAAATATGACCATCTGCCATCCCACTGAGTTTGACTTTTATTTGTGTAGCCA

TGCTGGTATACAGGGTGTTGAAAAGGTGAAACCTCCAACCGGTAGCCCAATATCTATGTTAATGGCTACT

CCAGATGGCTCATATGCATTACCAAGCAGTTGATGTTTTTAAGCTAAATGAGTGTCAGAGATACCATCAT

ATCTCAGTAAATCAGTGTTTGTCGAGCACAAAATGTTCTTGCCGATGATTAGTTGCCCAAAAATATTGTA

AGACATACTGTAAAAAGAATTCAAGAGGCCCATAACAGCAGCGGAGATAATAAAGGCCGTGATTCCCAGT

TGCAAGGTCTGCATTTTTTCTGTCTTTGGAATGTTGCTGTCATGGAATGCCCGAGGAAAATATAACTTCG

GAATCAAGAAGTTTCGAAAGGTAGCTTCGCAAATCAGCCCAACCAAAGCTGCTCAGAAGTACAACTTAGA

TGCAAGTTTTTTTTTCAAATATGTGTGAGTCCTTGTATTGATATCTATCAGGCTAACGAGTGATGCCACT

ATGGATATGATCTTCTAGTGAAATTTGTATATACATTCATTTATGCACGTATGTATATTGTGTTAACTGT

GCCAAATATTTATCATTTAATATTTTTATTCACGGCAA

>MSTRG.236.3 gene=MSTRG.236

AAAATGCCATCTGTACCCGCACAGTCCATCTTCATTCAGCTTCAGGCCCAAGCCCAGTTAGTCATTGTTT

TCTCCGCTGCCTGTGTGTATATGTACGTATACGTGTGAGATGAGGAAGAATCAATGGCGATTCCAAAGAA

TTAAGATCAATTCTCACACTCTGCATTGTTATGATAGTTGTTTCTATTGCTTTTACTCTCAAATTTTAAA

CAATGAAGAAAATGCTTCAGAAAGCCGATAAGAATGGTCGAAGAAAGCGCTTAATTAATAAATAAGGTAG

TGGCATCAATGGACTGGCCCGAGGTCACCACATACAAAGAACTCTACACACATGGTCAGTGAGAGGAAAT

ATTAAGGAATCTCTACTGCACTTGTGAAACTGAAAGGGGTGTTGTTGCTAGTGGCATGACCAGGGACTAT

TTGATGGCATTCTACCAGCAAACAGAACTCCGACCTAAACGGATCATATTCTACAGGGATGGAGTCAGTG

AAGGTCAGTTTAATCAGGTCTTGCTGAAAGAGATGGATGCCATTAGGACGGTTGATGTGTTTTATTTTTT

GTCTGCCAGGCGCTGCAATCAAATTATTTACAATGGCAGGAAATCAACTGATCGGAGTGGCAACATATCA

CCTGGTTACATATTGTTCTATTGTATCTTTAAAAATCTTTATTATTCTGATTAGCAAATAACTGGCTTTT

GTATACCAGGTACTGTTGTAGATATGACCATCTGCCATCCCACTGAGTTTGACTTTTATTTGTGTAGCCA

TGCTGGTATACAGGGTGTTGAAAAGGTGAAACCTCCAACCGGTAGCCCAATATCTATGTTAATGGCTACT

CCAGATGGCTCATATGCATTACCAAGCAGTTGATGTTTTTAAGCTAAATGAGTGTCAGAGATACCATCAT

ATCTCAGTAAATCAGTGTTTGTCGAGCACAAAATGTTCTTGCCGATGATTAGTTGCCCAAAAATATTGTA

AGACATACTGTAAAAAGAATTCAAGAGGCCCATAACAGCAGCGGAGATAATAAAGGCCGTGATTCCCAGT

TGCAAGGTCTGCATTTTTTCTGTCTTTGGAATGTTGCTGTCATGGAATGCCCGAGGAAAATATAACTTCG

GAATCAAGAAGTTTCGAAAGGTAGCTTCGCAAATCAGCCCAACCAAAGCTGCTCAGAAGTACAACTTAGA

TGCAAGTTTTTTTTTCAAATATGTGTGAGTCCTTGTATTGATATCTATCAGGCTAACGAGTGATGCCACT

ATGGATATGATCTTCTAGTGAAATTTGTATATACATTCATTTATGCACGTATGTATATTGTGTTAACTGT

GCCAAATATTTATCATTTAATATTTTTATTCACGGCAA

>MSTRG.236.4 gene=MSTRG.236

AAAATGCCATCTGTACCCGCACAGTCCATCTTCATTCAGCTTCAGGCCCAAGCCCAGTTAGTCATTGTTT

TCTCCGCTGCCTGTGTGTATATGTACGTATACGTGTGAGATGAGGAAGAATCAATGGCGATTCCAAAGAA

TTAAGATCAATTCTCACACTCTGCATTGTTATGATAGTTGTTTCTATTGCTTTTACTCTCAAATTTTAAA

CAATGAAGAAAATGCTTCAGAAAGCCGATAAGAATGGTCGAAGAAAGCGCTTAATTAATAAATAAGGTAG

TGGCATCAATGGACTGGCCCGAGGTCACCACATACAAAGAACTCTACACACATGGTCAGTGAGAGGAAAT

ATTAAGGAATCTCTACTGCACTTGTGAAACTGAAAGGGGTGTTGTTGCTAGTGGCATGACCAGGGACTAT

TTGATGGCATTCTACCAGCAAACAGAACTCCGACCTAAACGGATCATATTCTACAGGGATGGAGTCAGTG

AAGGTCAGTTTAATCAGGTCTTGCTGAAAGAGATGGATGCCATTAGGACGGTTGATGTGTTTTATTTTTT

GTCTGCCAGGCGCTGCAATCAAATTATTTACAATGGCAGGAAATCAACTGATCGGAGTGGCAACATATCA

CCTGGTTACATATTGTTCTATTGTATCTTTAAAAATCTTTATTATTCTGATTAGCAAATAACTGGCTTTT

GTATACCAGGTACTGTTGTAAATATGACCATCTGCCATCCCACTGAGTTTGACTTTTATTTGTGTAGCCA

TGCTGGTATACAGGGTGTTGAAAAGGTGAAACCTCCAACCGGTAGCCCAATATCTATGTTAATGGCTACT

CCAGATGGCTCATATGCATTACCAAGCAGTTGATGTTTTTAAGCTAAATGAGTGTCAGAGATACCATCAT

ATCTCAGTAAATCAGTGTTTGTCGAGCACAAAATGTTCTTGCCGATGATTAGTTGCCCAAAAATATTGTA

AGACATACTGTAAAAAGAATTCAAGAGGCCCATAACAGCAGCGGAGATAATAAAGGCCGTGATTCCCAGT

TGCAAGGTCTGCATTTTTTCTGTCTTTGGAATGTTGCTGTCATGGAATGCCCGAGGAAAATATAACTTCG

GAATCAAGAAGTTTCGAAAGGTAGCTTCGCAAATCAGCCCAACCAAAGCTGCTCAGAAGTACAACTTAGA

TGCAAGTTTTTTTTTCAAATATGTGTGAGTCCTTGTATTGATATCTATCAGGCTAACGAGTGATGCCACT

ATGGATATGATCTTCTAGTGAAATTTGTATATACATTCATTTATGCACGTATGTATATTGTGTTAACTGT

GCCAAATATTTATCATTTAATATTTTTATTCACGGCAA

>MSTRG.236.5 gene=MSTRG.236

AAAATGCCATCTGTACCCGCACAGTCCATCTTCATTCAGCTTCAGGCCCAAGCCCAGTTAGTCATTGTTT

TCTCCGCTGCCTGTGTGTATATGTACGTATACGTGTGAGATGAGGAAGAATCAATGGCGATTCCAAAGAA

TTAAGATCAATTCTCACACTCTGCATTGTTATGATAGTTGTTTCTATTGCTTTTACTCTCAAATTTTAAA

CAATGAAGAAAATGCTTCAGAAAGCCGATAAGAATGGTCGAAGAAAGCGCTTAATTAATAAATAAGGTAG

TGGCATCAATGGACTGGCCCGAGGTCACCACATACAAAGAACTCTACACACATGGTCAGTGAGAGGAAAT

ATTAAGGAATCTCTACTGCACTTGTGAAACTGAAAGGGGTGTTGTTGCTAGTGGCATGACCAGGGACTAT

TTGATGGCATTCTACCAGCAAACAGAACTCCGACCTAAACGGATCATATTCTACAGGGATGGAGTCAGTG

AAGGTCAGTTTAATCAGGTCTTGCTGAAAGAGATGGATGCCATTAGGACGGTTGATGTGTTTTATTTTTT

GTCTGCCAGGCGCTGCAATCAAATTATTTACAATGGCAGGAAATCAACTGATCGGAGTGGCAACATATCA

CCTGGTACTGTTGTAAATATGACCATCTGCCATCCCACTGAGTTTGACTTTTATTTGTGTAGCCATGCTG

GTATACAGGACGGACTTTGACTTTGTTGTCGGCAATGCACAGACTAATGAGGGTGTTGAAAAGGTGAAAC

CTCCAACCGGTAGCCCAATATCTATGTTAATGGCTACTCCAGATGGCTCATATGCATTACCAAGCAGTTG

ATGTTTTTAAGCTAAATGAGTGTCAGAGATACCATCATATCTCAGTAAATCAGTGTTTGTCGAGCACAAA

ATGTTCTTGCCGATGATTAGTTGCCCAAAAATATTGTAAGACATACTGTAAAAAGAATTCAAGAGGCCCA

TAACAGCAGCGGAGATAATAAAGGCCGTGATTCCCAGTTGCAAGGTCTGCATTTTTTCTGTCTTTGGAAT

GTTGCTGTCATGGAATGCCCGAGGAAAATATAACTTCGGAATCAAGAAGTTTCGAAAGGTAGCTTCGCAA

ATCAGCCCAACCAAAGCTGCTCAGAAGTACAACTTAGATGCAAGTTTTTTTTTCAAATATGTGTGAGTCC

TTGTATTGATATCTATCAGGCTAACGAGTGATGCCACTATGGATATGATCTTCTAGTGAAATTTGTATAT

ACATTCATTTATGCACGTATGTATATTGTGTTAACTGTGCCAAATATTTATCATTTAATATTTTTATTCA

CGGCAA

>MSTRG.236.6 gene=MSTRG.236

AAAATGCCATCTGTACCCGCACAGTCCATCTTCATTCAGCTTCAGGCCCAAGCCCAGTTAGTCATTGTTT

TCTCCGCTGCCTGTGTGTATATGTACGTATACGTGTGAGATGAGGAAGAATCAATGGCGATTCCAAAGAA

TTAAGATCAATTCTCACACTCTGCATTGTTATGATAGTTGTTTCTATTGCTTTTACTCTCAAATTTTAAA

CAATGAAGAAAATGCTTCAGAAAGCCGATAAGAATGGTCGAAGAAAGCGCTTAATTAATAAATAAGGTAG

TGGCATCAATGGACTGGCCCGAGGTCACCACATACAAAGAACTCTACACACATGGTCAGTGAGAGGAAAT

ATTAAGGAATCTCTACTGCACTTGTGAAACTGAAAGGGGTGTTGTTGCTAGTGGCATGACCAGGGACTAT

TTGATGGCATTCTACCAGCAAACAGAACTCCGACCTAAACGGATCATATTCTACAGGGATGGAGTCAGTG

AAGGTCAGTTTAATCAGGTCTTGCTGAAAGAGATGGATGCCATTAGGACGGTTGATGTGTTTTATTTTTT

GTCTGCCAGGCGCTGCAATCAAATTATTTACAATGGCAGGAAATCAACTGATCGGAGTGGCAACATATCA

CCTGGTTACATATTGTTCTATTGTATCTTTAAAAATCTTTATTATTCTGATTAGCAAATAACTGGCTTTT

GTATACCAGGTACTGTTGTAAATATGACCATCTGCCATCCCACTGAGTTTGACTTTTATTTGTGTAGCCA

TGCTGGTATACAGGACGGACTTTGACTTTGTTGTCGGCAATGCACAGACTAATGAGGGTGTTGAAAAGGT

GAAACCTCCAACCGGTAGCCCAATATCTATGTTAATGGCTACTCCAGATGGCTCATATGCATTACCAAGC

AGTTGATGTTTTTAAGCTAAATGAGTGTCAGAGATACCATCATATCTCAGTAAATCAGTGTTTGTCGAGC

ACAAAATGTTCTTGCCGATGATTAGTTGCCCAAAAATATTGTAAGACATACTGTAAAAAGAATTCAAGAG

GCCCATAACAGCAGCGGAGATAATAAAGGCCGTGATTCCCAGTTGCAAGGTCTGCATTTTTTCTGTCTTT

GGAATGTTGCTGTCATGGAATGCCCGAGGAAAATATAACTTCGGAATCAAGAAGTTTCGAAAGGTAGCTT

CGCAAATCAGCCCAACCAAAGCTGCTCAGAAGTACAACTTAGATGCAAGTTTTTTTTTCAAATATGTGTG

AGTCCTTGTATTGATATCTATCAGGCTAACGAGTGATGCCACTATGGATATGATCTTCTAGTGAAATTTG

TATATACATTCATTTATGCACGTATGTATATTGTGTTAACTGTGCCAAATATTTATCATTTAATATTTTT

ATTCACGGCAA

>MSTRG.236.9 gene=MSTRG.236

TCCATCTTCATTCAGGTTCAGGCCCAAGCCCAGTTAGTCATTGTTTTCTCCGCTGCTTGTGTGTATATGT

ACGTATACGTGTGAGATGAGGATGAATCAATGGCGATTCCAAAGATTTAAGATCAATTCTCACACTCTAC

ATTGTTATGATACTTGTTTCTATCGCTTCCACTCTCAAATTTTAAACAATGAAGAAAGTGCTTCAGGAAG

CCGATAAGAATGGTCGAAGAAAGCGCTTAATTAATAAATAAGGTAGTGGCATCAATGGACTGGCCTGAGG

TCACCACATACAGAGAACTCTACACACATGGTCAGTGAGAGGAAATATTAAGGAATCTCTACTGCACTTG

TGAAACTGAAAGGGGTGTTGTTGCTAGTGGCATGACCAGGGACTATTTGATGGCATTCTACCAGCAAACA

GAACTCCGACCTAAACGGATCATATTCTACAGGGATGGAGTCAGTGAAGGTCAGTTTAATCAGGTCTTGC

TGAAAGAGATGGATGCCATTAGGACGGTTGATGTGTTTTATTTTTTGTAAGTAATTACTACCTTCACTTG

TTAGTTGTTACCCCTATAGTTCAAGTTTGATGAATGGGAAGATATATCTGTTTTTAAGGTCTGCCAGGCG

CTGCAATCAAATTATTTACAATGGCAGGAAATCAACTGATCGGAGTGGCAACATATCACCTGGTTACATA

TTGTTCTATTGTATCTTTAAAAATCTTTATTATTCTGATTAGCAAATAACTGGCTTTTGTATACCAGGTA

CTGTTGTAGATATGACCATCTGCCATCCCACTGAGTTTGACTTTTATTTGTGTAGCCATGCTGGTATACA

GGGTGTTGAAAAGGTGAAACCTCCAACCGGTAGCCCAATATCTATGTTAATGGCTACTCCAGATGGCTCA

TATGCATTACCAAGCAGTTGATGTTTTTAAGCTAAATGAGTGTCAGAGATACCATCATATCTCAGTAAAT

CAGTGTTTGTCGAGCACAAAATGTTCTTGCCGATGATTAGTTGCCCAAAAATATTGTAAGACATACTGTA

AAAAGAATTCAAGAGGCCCATAACAGCAGCGGAGATAATAAAGGCCGTGATTCCCAGTTGCAAGGTCTGC

ATTTTTTCTGTCTTTGGAATGTTGCTGTCATGGAATGCCCGAGGAAAATATAACTTCGGAATCAAGAAGT

TTCGAAAGGTAGCTTCGCAAATCAGCCCAACCAAAGCTGCTCAGAAGTACAACTTAGATGCAAGTTTTTT

TTTCAAATATGTGTGAGTCCTTGTATTGATATCTATCAGGCTAACGAGTGATGCCACTATGGATATGATC

TTCTAGTGAAATTTGTATATACATTCATTTATGCACGTATGTATATTGTGTTAACTGTGCC

>MSTRG.236.8 gene=MSTRG.236

CTCCAAAATGCCATCTGTACCCGCACAGTCCATCTTCATTCAGCTTCAGGCCCAAGCCCAGTTAGTCATT

GTTTTCTCCGCTGCCTGTGTGTATATGTACGTATACGTGTGAGATGAGGAAGAATCAATGGCGATTCCAA

AGAATTAAGATCAATTCTCACACTCTGCATTGTTATGATAGTTGTTTCTATTGCTTTTACTCTCAAATTT

TAAACAATGAAGAAAATGCTTCAGAAAGCCGATAAGAATGGTCGAAGAAAGCGCTTAATTAATAAATAAG

GTAGTGGCATCAATGGACTGGCCCGAGGTCACCACATACAAAGAACTCTACACACATGGTCAGTGAGAGG

AAATATTAAGGAATCTCTACTGCACTTGTGAAACTGAAAGGGGTGTTGTTGCTAGTGGCATGACCAGGGA

CTATTTGATGGCATTCTACCAGCAAACAGAACTCCGACCTAAACGGATCATATTCTACAGGGATGGAGTC

AGTGAAGGTCAGTTTAATCAGGTCTTGCTGAAAGAGATGGATGCCATTAGGACGGTTGATGTGTTTTATT

TTTTGTCTGCCAGGCGCTGCAATCAAATTATTTACAATGGCAGGAAATCAACTGATCGGAGTGGCAACAT

ATCACCTGGTACTGTTGTAAATATGACCATCTGCCATCCCACTGAGTTTGACTTTTATTTGTGTAGCCAT

GCTGGTATACAGGTAATCATCTTAATACAAGACTACATGGATTTTGTACAGGCAGCAGTAATAAATGCAT

GTGTTGTGTGGCAACACAATAGAGATACAAATGTACATATTTATTAATATACAAATAGAGAAACAAATGT

AAAGATCTTAATCAAAATAAACACAAATACACTCATATTAAAAAACTAACATGAACCAATGAGATATAGA

AACCATTAAAAATCAACTAGTTTATTCAATCTACCAAAACATCTAAGTAAAGGAAATCCAGATCTGTTCA

TATTTTACCGGTAACAATTAGCTAGTTAGGATATTAAATAAGAACAGAAAATTTATGCTCACTAGTTCTC

CACGAATTTAGGAAGCTTGTTGATTGAATATCATATACTAAAACACACAATTTCTACTATACATACTGTG

ACTATTTTAATGTCATTAGCCTAATTCATAACTTACGAAAAATGCTTATGGAGTATGTAGATTCAGGATA

GGTAAATAAAAGGATCAGGGAACATGTAAGCTAACAAACACTGCATTGTATCTTGTTGAATAATGAATTT

GTTTAAAATAAAACTTGCTCTGACGCACGTGATCTACGATGGAGTTATATACGCATGCCATTGCATTTAC

ATGTATAGGTTTCCCTATGAACTTTATTTAATGTGATATCTTAATGGACAAAATTTAATATTTTAATATT

CTCTTATCTGTTAATGCATTCATAGATTATGACACTCAAAATATTTTAATGGGCAATAGGACGGACTTTG

ACTTTGTTGTCGGCAATGCACAGACTAATGAGGGTGTTGAAAAGGTGAAACCTCCAACCGGTAGCCCAAT

ATCTATGTTAATGGCTACTCCAGATGGCTCATATGCATTACCAAGCAGTTGATGTTTTTAAGCTAAATGA

GTGTCAGAGATACCATCATATCTCAGTAAATCAGTGTTTGTCGAGCACAAAATGTTCTTGCCGATGATTA

GTTGCCCAAAAATATTGTAAGACATACTGTAAAAAGAATTCAAGAGGCCCATAACAGCAGCGGAGATAAT

AAAGGCCGTGATTCCCAGTTGCAAGGTCTGCATTTTTTCTGTCTTTGGAATGTTGCTGTCATGGAATGCC

CGAGGAAAATATAACTTCGGAATCAAGAAGTTTCGAAAGGTAGCTTCGCAAATCAGCCCAACCAAAGCTG

CTCAGAAGTACAACTTAGATGCAAGTTTTTTTTTCAAATATGTGTGAGTCCTTGTATTGATATCTATCAG

GCTAACGAGTGATGCCACTATGGATATGATCTTCTAGTGAAATTTGTATATACATTCATTTATGCACGTA

TGTATATTGTGTTAACTGTGCC

>MSTRG.236.10 gene=MSTRG.236

CTCCAAAATGCCATCTGTACCCGCACAGTCCATCTTCATTCAGCTTCAGGCCCAAGCCCAGTTAGTCATT

GTTTTCTCCGCTGCCTGTGTGTATATGTACGTATACGTGTGAGATGAGGAAGAATCAATGGCGATTCCAA

AGAATTAAGATCAATTCTCACACTCTGCATTGTTATGATAGTTGTTTCTATTGCTTTTACTCTCAAATTT

TAAACAATGAAGAAAATGCTTCAGAAAGCCGATAAGAATGGTCGAAGAAAGCGCTTAATTAATAAATAAG

GTAGTGGCATCAATGGACTGGCCCGAGGTCACCACATACAAAGAACTCTACACACATGGTCAGTGAGAGG

AAATATTAAGGAATCTCTACTGCACTTGTGAAACTGAAAGGGGTGTTGTTGCTAGTGGCATGACCAGGGA

CTATTTGATGGCATTCTACCAGCAAACAGAACTCCGACCTAAACGGATCATATTCTACAGGGATGGAGTC

AGTGAAGGTCAGTTTAATCAGGTCTTGCTGAAAGAGATGGATGCCATTAGGACGGTTGATGTGTTTTATT

TTTTGTCTGCCAGGCGCTGCAATCAAATTATTTACAATGGCAGGAAATCAACTGATCGGAGTGGCAACAT

ATCACCTGGTACTGTTGTAAATATGACCATCTGCCATCCCACTGAGTTTGACTTTTATTTGTGTAGCCAT

GCTGGTATACAGGGTGTTGAAAAGGTGAAACCTCCAACCGGTAGCCCAATATCTATGTTAATGGCTACTC

CAGATGGCTCATATGCATTACCAAGCAGTTGATGTTTTTAAGCTAAATGAGTGTCAGAGATACCATCATA

TCTCAGTAAATCAGTGTTTGTCGAGCACAAAATGTTCTTGCCGATGATTAGTTGCCCAAAAATATTGTAA

GACATACTGTAAAAAGAATTCAAGAGGCCCATAACAGCAGCGGAGATAATAAAGGCCGTGATTCCCAGTT

GCAAGGTCTGCATTTTTTCTGTCTTTGGAATGTTGCTGTCATGGAATGCCCGAGGAAAATATAACTTCGG

AATCAAGAAGTTTCGAAAGGTAGCTTCGCAAATCAGCCCAACCAAAGCTGCTCAGAAGTACAACTTAGAT

GCAAGTTTTTTTTTCAAATATGTGTGAGTCCTTGTATTGATATCTATCAGGCTAACGAGTGATGCCACTA

TGGATATGATCTTCTAGTGAAATTTGTATATACATTCATTTATGCACGTATGTATATT

>MSTRG.236.11 gene=MSTRG.236

CTCCAAAATGCCATCTGTACCCGCACAGTCCATCTTCATTCAGCTTCAGGCCCAAGCCCAGTTAGTCATT

GTTTTCTCCGCTGCCTGTGTGTATATGTACGTATACGTGTGAGATGAGGAAGAATCAATGGCGATTCCAA

AGAATTAAGATCAATTCTCACACTCTGCATTGTTATGATAGTTGTTTCTATTGCTTTTACTCTCAAATTT

TAAACAATGAAGAAAATGCTTCAGAAAGCCGATAAGAATGGTCGAAGAAAGCGCTTAATTAATAAATAAG

GTAGTGGCATCAATGGACTGGCCCGAGGTCACCACATACAAAGAACTCTACACACATGGTCAGTGAGAGG

AAATATTAAGGAATCTCTACTGCACTTGTGAAACTGAAAGGGGTGTTGTTGCTAGTGGCATGACCAGGGA

CTATTTGATGGCATTCTACCAGCAAACAGAACTCCGACCTAAACGGATCATATTCTACAGGGATGGAGTC

AGTGAAGGTCAGTTTAATCAGGTCTTGCTGAAAGAGATGGATGCCATTAGGACGGTTGATGTGTTTTATT

TTTTGTCTGCCAGGCGCTGCAATCAAATTATTTACAATGGCAGGAAATCAACTGATCGGAGTGGCAACAT

ATCACCTGGTACTGTTGTAAATATGACCATCTGCCATCCCACTGAGTTTGACTTTTATTTGTGTAGCCAT

GCTGGTATACAGATTATGACACTCAAAATATTTTAATGGGCAATAGGACGGACTTTGACTTTGTTGTCGG

CAATGCACAGACTAATGAGGTTGGTTGGATTAAATCTCTTAGGTGTTTACCATATTGTTGCTTAATAATG

CATTTGAAGGATGGGAGGTGATCTTTACTTGATGTAGCAATGAAGATGAAAAACAGATCACCTATTTGTA

ATATTGATATGCGGATTATGCTTGTCTGTGCTCTATTATGCTTATTTTTGCTCTAATGTTGGATGTTAAT

ATGCTTCCATTATCCTACAAATGGAGTTAGGGTGTTGAAAAGGTGAAACCTCCAACCGGTAGCCCAATAT

CTATGTTAATGGCTACTCCAGATGGCTCATATGCATTACCAAGCAGTTGATGTTTTTAAGCTAAATGAGT

GTCAGAGATACCATCATATCTCAGTAAATCAGTGTTTGTCGAGCACAAAATGTTCTTGCCGATGATTAGT

TGCCCAAAAATATTGTAAGACATACTGTAAAAAGAATTCAAGAGGCCCATAACAGCAGCGGAGATAATAA

AGGCCGTGATTCCCAGTTGCAAGGTCTGCATTTTTTCTGTCTTTGGAATGTTGCTGTCATGGAATGCCCG

AGGAAAATATAACTTCGGAATCAAGAAGTTTCGAAAGGTAGCTTCGCAAATCAGCCCAACCAAAGCTGCT

CAGAAGTACAACTTAGATGCAAGTTTTTTTTTCAAATATGTGTGAGTCCTTGTATTGATATCTATCAGGC

TAACGAGTGATGCCACTATGGATATGATCTTCTAGTGAAATTTGTATATACATTCATTTATGCACGTATG

TATATT

>MSTRG.236.12 gene=MSTRG.236

GCCATCTGTACCCGCACAGTCCATCTTCATTCAGCTTCAGGCCCAAGCCCAGTTAGTCATTGTTTTCTCC

GCTGCCTGTGTGTATATGTACGTATACGTGTGAGATGAGGAAGAATCAATGGCGATTCCAAAGAATTAAG

ATCAATTCTCACACTCTGCATTGTTATGATAGTTGTTTCTATTGCTTTTACTCTCAAATTTTAAACAATG

AAGAAAGTGCTTCAGGAAGCCGATAAGAATGGTCGAAGAAAGCGCTTAATTAATAAATAAGGTAGTGGCA

TCAATGGACTGGCCTGAGGTCACCACATACAGAGAACTCTACACACATGGTCAGTGAGAGGAAATATTAA

GGAATCTCTACTGCACTTGTGAAACTGAAAGGGGTGTTGTTGCTAGTGGCATGACCAGGGACTATTTGAT

GGCATTCTACCAGCAAACAGAACTCCGACCTAAACGGATCATATTCTACAGGGATGGAGTCAGTGAAGGT

CAGTTTAATCAGGTCTTGCTGAAAGAGATGGATGCCATTAGGACGGTTGATGTGTTTTATTTTTTGTCTG

CCAGGCGCTGCAATCAAATTATTTACAATGGCAGGAAATCAACTGATCGGAGTGGCAACATATCACCTGG

TTACATATTGTTCTATTGTATCTTTAAAAATCTTTATTATTCTGATTAGCAAATAACTGGCTTTTGTATA

CCAGGTACTGTTGTAGATATGACCATCTGCCATCCCACTGAGTTTGACTTTTATTTGTGTAGCCATGCTG

GTATACAGGTAATCATCTTAATACAAGACCACATGGATTTTGTACAGGCAGCAGTAATAAATGCATGTGT

TGTGTGGCAACACAATAGAGATACAAATGTACATATTTATTAATATACAAATAGAGAAACAAATGTAAAG

ATCATAATCAAAATAAACACAAATACACTCATATTAAAAAACTAACATGAACCAATGAGATATAGAAACC

ATTAAACATCAACTAGTTTATTCAATCTACCAAAACATCTGAGTAAAGGAAATCCAGATCTGTTCATATT

TTACCGGTAACAATTAGCTAGTTAGGATATTAAATAAGAACAGAAAATTTATGCTCACTAGTTCTCCACG

AATTTAGGAAGCTTGTTGATTGAATATCATATACTAAAACACACAAATTCTACTATACATACTGTGACTA

TTTTAATGTCATTAGCCTAATTCATAACTTACGAAAAATGCTTATGGAGTATGTAGATTCAGGATAGGCA

AATAAAAGGATCAGGGAACATGTAAGCTAACAAACACTGCATTGTATCTTGTTGAATAATGAATTTGTTT

AAAATAAAACTTGCTCTGACGCACGTGATCTACGATGGAGTTATATATGCATGCCATTGCATTTACATGT

ATAGGTTTCCCTATGAAATTTATTCAATGTGATATCTTAATGGACAAAATTTAATATTTTAATATTCTCT

TATCTGTTAATGCATTCATAGATTATGACACTCAAAATATTTTAATGGGCAATAGGACGGACTTTGACTT

TGTTGTCGGCAATGCACAGACTAATGAGGGTGTTGAAAAGGTGAAACCTCCAACCGGTAGCCCAATATCT

ATGTTAATGGCTACTCCAGATGGCTCATATGCATTACCAAGCAGTTGATGTTTTTAAGCTAAATGAGTGT

CAGAGATACCATCATATCTCAGTAAATCAGTGTTTGTCGAGCACAAAATGTTCTTGCCGATGATTAGTTG

CCCAAAAATATTGTAAGACATACTGTAAAAAGAATTCAAGAGGCCCATAACAGCAGCGGAGATAATAAAG

GCCGTGATTCCCAGTTGCAAGGTCTGCATTTTTTCTGTCTTTGGAATGTTGCTGTCATGGAATGCCCGAG

GAAAATATAACTTCGGAATCAAGAAGTTTCGAAAGGTAGCTTCGCAAATCAGCCCAACCAAAGCTGCTCA

GAAGTACAACTTAGATGCAAGTTTTTTTTTCAAATATGTGTGAGTCCTTGTATTGATATCTATCAGGCTA

ACGAGTGATGCCACTATGGATATGATCTTCTAGTGAAATTTGTATATACATTCATTTATGCACG

>MSTRG.236.13 gene=MSTRG.236

GCCATCTGTACCCGCACAGTCCATCTTCATTCAGCTTCAGGCCCAAGCCCAGTTAGTCATTGTTTTCTCC

GCTGCCTGTGTGTATATGTACGTATACGTGTGAGATGAGGAAGAATCAATGGCGATTCCAAAGAATTAAG

ATCAATTCTCACACTCTGCATTGTTATGATAGTTGTTTCTATTGCTTTTACTCTCAAATTTGTAAGTAAT

TTCCTTCCAGATCTTCAATTAATTATTTCTCTGAATCAAAAATAAACAATGAAGAAAATGCTTCAGAAAG

CCGATAAGAATGGTCGAAGAAAGCGCTTAATTAATAAATAAGGTAGTGGCATCAATGGACTGGCCCGAGG

TCACCACATACAAAGAACTCTACACACATGGTCAGTGAGAGGAAATATTAAGGAATCTCTACTGCACTTG

TGAAACTGAAAGGGGTGTTGTTGCTAGTGGCATGACCAGGGACTATTTGATGGCATTCTACCAGCAAACA

GAACTCCGACCTAAACGGATCATATTCTACAGGGATGGAGTCAGTGAAGGTCAGTTTAATCAGGTCTTGC

TGAAAGAGATGGATGCCATTAGGACGGTTGATGTGTTTTATTTTTTGTAAGTAATTACTACCTTCACTTG

TTAGTTGTTACCCCTATAGTTCAAGTTTGATGAATGGGAAGATATATCTGTTTTTAAGGTCTGCCAGGCG

CTGCAATCAAATTATTTACAATGGCAGGAAATCAACTGATCGGAGTGGCAACATATCACCTGGTTACATA

TTGTTCTATTGTATCTTTAAAAATCTTTATTATTCTGATTAGCAAATAACTGGCTTTTGTATACCAGGTA

CTGTTGTAGATATGACCATCTGCCATCCCACTGAGTTTGACTTTTATTTGTGTAGCCATGCTGGTATACA

GGGTGTTGAAAAGGTGAAACCTCCAACCGGTAGCCCAATATCTATGTTAATGGCTACTCCAGATGGCTCA

TATGCATTACCAAGCAGTTGATGTTTTTAAGCTAAATGAGTGTCAGAGATACCATCATATCTCAGTAAAT

CAGTGTTTGTCGAGCACAAAATGTTCTTGCCGATGATTAGTTGCCCAAAAATATTGTAAGACATACTGTA

AAAAGAATTCAAGAGGCCCATAACAGCAGCGGAGATAATAAAGGCCGTGATTCCCAGTTGCAAGGTCTGC

ATTTTTTCTGTCTTTGGAATGTTGCTGTCATGGAATGCCCGAGGAAAATATAACTTCGGAATCAAGAAGT

TTCGAAAGGTAGCTTCGCAAATCAGCCCAACCAAAGCTGCTCAGAAGTACAACTTAGATGCAAGTTTTTT

TTTCAAATATGTGTGAGTCCTTGTATTGATATCTATCAGGCTAACGAGTGATGCCACTATGGATATGATC

TTCTAGTGAAATTTGTATATACATTCATTTATGCACG

>MSTRG.236.14 gene=MSTRG.236

GCACAGTCCATCTTCATTCAGCTTCAGGCCCAAGCCCAGTTAGTCATTGTTTTCTCCGCTGCCTGTGTGT

ATATGTACGTATACGTGTGAGATGAGGAAGAATCAATGGCGATTCCAAAGAATTAAGATCAATTCTCACA

CTCTGCATTGTTATGATAGTTGTTTCTATTGCTTTTACTCTCAAATTTGTAAGTAATTTCCTTCCAGATC

TTCAATTAATTATTTCTCTGAATCAAAAATAAACAATGAAGAAAATGCTTCAGAAAGCCGATAAGAATGG

TCGAAGAAAGCGCTTAATTAATAAATAAGGTAGTGGCATCAATGGACTGGCCCGAGGTCACCACATACAA

AGAACTCTACACACATGGTCAGTGAGAGGAAATATTAAGGAATCTCTACTGCACTTGTGAAACTGAAAGG

GGTGTTGTTGCTAGTGGCATGACCAGGGACTATTTGATGGCATTCTACCAGCAAACAGAACTCCGACCTA

AACGGATCATATTCTACAGGGATGGAGTCAGTGAAGGTCAGTTTAATCAGGTCTTGCTGAAAGAGATGGA

TGCCATTAGGACGGTTGATGTGTTTTATTTTTTGTCTGCCAGGCGCTGCAATCAAATTATTTACAATGGC

AGGAAATCAACTGATCGGAGTGGCAACATATCACCTGGTTACATATTGTTCTATTGTATCTTTAAAAATC

TTTATTATTCTGATTAGCAAATAACTGGCTTTTGTATACCAGGTACTGTTGTAAATATGACCATCTGCCA

TCCCACTGAGTTTGACTTTTATTTGTGTAGCCATGCTGGTATACAGGTAATCATCTTAATACAAGACTAC

ATGGATTTTGTACAGGCAGCAGTAATAAATGCATGTGTTGTGTGGCAACACAATAGAGATACAAATGTAC

ATATTTATTAATATACAAATAGAGAAACAAATGTAAAGATCTTAATCAAAATAAACACAAATACACTCAT

ATTAAAAAACTAACATGAACCAATGAGATATAGAAACCATTAAAAATCAACTAGTTTATTCAATCTACCA

AAACATCTAAGTAAAGGAAATCCAGATCTGTTCATATTTTACCGGTAACAATTAGCTAGTTAGGATATTA

AATAAGAACAGAAAATTTATGCTCACTAGTTCTCCACGAATTTAGGAAGCTTGTTGATTGAATATCATAT

ACTAAAACACACAATTTCTACTATACATACTGTGACTATTTTAATGTCATTAGCCTAATTCATAACTTAC

GAAAAATGCTTATGGAGTATGTAGATTCAGGATAGGTAAATAAAAGGATCAGGGAACATGTAAGCTAACA

AACACTGCATTGTATCTTGTTGAATAATGAATTTGTTTAAAATAAAACTTGCTCTGACGCACGTGATCTA

CGATGGAGTTATATACGCATGCCATTGCATTTACATGTATAGGTTTCCCTATGAACTTTATTTAATGTGA

TATCTTAATGGACAAAATTTAATATTTTAATATTCTCTTATCTGTTAATGCATTCATAGATTATGACACT

CAAAATATTTTAATGGGCAATAGGACGGACTTTGACTTTGTTGTCGGCAATGCACAGACTAATGAGGTTG

GTTGGATTAAATCTCTTAGGTGTTTACCATATTGTTGCTTAATAATGCATTTGAAGGATGGGAGGTGATC

TTTACTTGATGTAGCAATGAAGATGAAAAACAGATCACCTATTTGTAATATTGATATGCGGATTATGCTT

GTCTGTGCTCTATTATGCTTATTTTTGCTCTAATGTTGGATGTTAATATGCTTCCATTATCCTACAAATG

GAGTTAGGGTGTTGAAAAGGTGAAACCTCCAACCGGTAGCCCAATATCTATGTTAATGGCTACTCCAGAT

GGCTCATATGCATTACCAAGCAGTTGATGTTTTTAAGCTAAATGAGTGTCAGAGATACCATCATATCTCA

GTAAATCAGTGTTTGTCGAGCACAAAATGTTCTTGCCGATGATTAGTTGCCCAAAAATATTGTAAGACAT

ACTGTAAAAAGAATTCAAGAGGCCCATAACAGCAGCGGAGATAATAAAGGCCGTGATTCCCAGTTGCAAG

GTCTGCATTTTTTCTGTCTTTGGAATGTTGCTGTCATGGAATGCCCGAGGAAAATATAACTTCGGAATCA

AGAAGTTTCGAAAGGTAGCTTCGCAAATCAGCCCAACCAAAGCTGCTCAGAAGTACAACTTAGATGCAAG

TTTTTTTTTCAAATATGTGTGAGTCCTTGTATTGATATCTATCAGGCTAACGAGTGATGCCACTATGGAT

ATGATCTTCTAGTGAAATTTGTATATACATTCATTTATGCAC

>MSTRG.236.15 gene=MSTRG.236

GCACAGTCCATCTTCATTCAGCTTCAGGCCCAAGCCCAGTTAGTCATTGTTTTCTCCGCTGCCTGTGTGT

ATATGTACGTATACGTGTGAGATGAGGAAGAATCAATGGCGATTCCAAAGAATTAAGATCAATTCTCACA

CTCTGCATTGTTATGATAGTTGTTTCTATTGCTTTTACTCTCAAATTTTAAACAATGAAGAAAATGCTTC

AGAAAGCCGATAAGAATGGTCGAAGAAAGCGCTTAATTAATAAATAAGGTAGTGGCATCAATGGACTGGC

CCGAGGTCACCACATACAAAGAACTCTACACACATGGTCAGTGAGAGGAAATATTAAGGAATCTCTACTG

CACTTGTGAAACTGAAAGGGGTGTTGTTGCTAGTGGCATGACCAGGGACTATTTGATGGCATTCTACCAG

CAAACAGAACTCCGACCTAAACGGATCATATTCTACAGGGATGGAGTCAGTGAAGGTCAGTTTAATCAGG

TCTTGCTGAAAGAGATGGATGCCATTAGGACGGTTGATGTGTTTTATTTTTTGTCTGCCAGGCGCTGCAA

TCAAATTATTTACAATGGCAGGAAATCAACTGATCGGAGTGGCAACATATCACCTGGTACTGTTGTAAAT

ATGACCATCTGCCATCCCACTGAGTTTGACTTTTATTTGTGTAGCCATGCTGGTATACAGGGTGTTGAAA

AGGTGAAACCTCCAACCGGTAGCCCAATATCTATGTTAATGGCTACTCCAGATGGCTCATATGCATTACC

AAGCAGTTGATGTTTTTAAGCTAAATGAGTGTCAGAGATACCATCATATCTCAGTAAATCAGTGTTTGTC

GAGCACAAAATGTTCTTGCCGATGATTAGTTGCCCAAAAATATTGTAAGACATACTGTAAAAAGAATTCA

AGAGGCCCATAACAGCAGCGGAGATAATAAAGGCCGTGATTCCCAGTTGCAAGGTCTGCATTTTTTCTGT

CTTTGGAATGTTGCTGTCATGGAATGCCCGAGGAAAATATAACTTCGGAATCAAGAAGTTTCGAAAGGTA

GCTTCGCAAATCAGCCCAACCAAAGCTGCTCAGAAGTACAACTTAGATGCAAGTTTTTTTTTCAAATATG

TGTGAGTCCTTGTATTGATATCTATCAGGCTAACGAGTGATGCCACTATGGATATGATCTTCTAGTGAAA

TTTGTATATACATTCATTTATGCAC

>MSTRG.236.16 gene=MSTRG.236

GCACAGTCCATCTTCATTCAGCTTCAGGCCCAAGCCCAGTTAGTCATTGTTTTCTCCGCTGCCTGTGTGT

ATATGTACGTATACGTGTGAGATGAGGAAGAATCAATGGCGATTCCAAAGAATTAAGATCAATTCTCACA

CTCTGCATTGTTATGATAGTTGTTTCTATTGCTTTTACTCTCAAATTTTAAACAATGAAGAAAATGCTTC

AGAAAGCCGATAAGAATGGTCGAAGAAAGCGCTTAATTAATAAATAAGGTAGTGGCATCAATGGACTGGC

CCGAGGTCACCACATACAAAGAACTCTACACACATGGTCAGTGAGAGGAAATATTAAGGAATCTCTACTG

CACTTGTGAAACTGAAAGGGGTGTTGTTGCTAGTGGCATGACCAGGGACTATTTGATGGCATTCTACCAG

CAAACAGAACTCCGACCTAAACGGATCATATTCTACAGGGATGGAGTCAGTGAAGGTCAGTTTAATCAGG

TCTTGCTGAAAGAGATGGATGCCATTAGGACGGTTGATGTGTTTTATTTTTTGTCTGCCAGGCGCTGCAA

TCAAATTATTTACAATGGCAGGAAATCAACTGATCGGAGTGGCAACATATCACCTGGTTACATATTGTTC

TATTGTATCTTTAAAAATCTTTATTATTCTGATTAGCAAATAACTGGCTTTTGTATACCAGGTACTGTTG

TAAATATGACCATCTGCCATCCCACTGAGTTTGACTTTTATTTGTGTAGCCATGCTGGTATACAGATTAT

GACACTCAAAATATTTTAATGGGCAATAGGACGGACTTTGACTTTGTTGTCGGCAATGCACAGACTAATG

AGGTTGGTTGGATTAAATCTCTTAGGTGTTTACCATATTGTTGCTTAATAATGCATTTGAAGGATGGGAG

GTGATCTTTACTTGATGTAGCAATGAAGATGAAAAACAGATCACCTATTTGTAATATTGATATGCGGATT

ATGCTTGTCTGTGCTCTATTATGCTTATTTTTGCTCTAATGTTGGATGTTAATATGCTTCCATTATCCTA

CAAATGGAGTTAGGGTGTTGAAAAGGTGAAACCTCCAACCGGTAGCCCAATATCTATGTTAATGGCTACT

CCAGATGGCTCATATGCATTACCAAGCAGTTGATGTTTTTAAGCTAAATGAGTGTCAGAGATACCATCAT

ATCTCAGTAAATCAGTGTTTGTCGAGCACAAAATGTTCTTGCCGATGATTAGTTGCCCAAAAATATTGTA

AGACATACTGTAAAAAGAATTCAAGAGGCCCATAACAGCAGCGGAGATAATAAAGGCCGTGATTCCCAGT

TGCAAGGTCTGCATTTTTTCTGTCTTTGGAATGTTGCTGTCATGGAATGCCCGAGGAAAATATAACTTCG

GAATCAAGAAGTTTCGAAAGGTAGCTTCGCAAATCAGCCCAACCAAAGCTGC

>MSTRG.236.17 gene=MSTRG.236

GCACAGTCCATCTTCATTCAGCTTCAGGCCCAAGCCCAGTTAGTCATTGTTTTCTCCGCTGCCTGTGTGT

ATATGTACGTATACGTGTGAGATGAGGAAGAATCAATGGCGATTCCAAAGAATTAAGATCAATTCTCACA

CTCTGCATTGTTATGATAGTTGTTTCTATTGCTTTTACTCTCAAATTTTAAACAATGAAGAAAGTGCTTC

AGGAAGCCGATAAGAATGGTCGAAGAAAGCGCTTAATTAATAAATAAGGTAGTGGCATCAATGGACTGGC

CTGAGGTCACCACATACAGAGAACTCTACACACATGGTCAGTGAGAGGAAATATTAAGGAATCTCTACTG

CACTTGTGAAACTGAAAGGGGTGTTGTTGCTAGTGGCATGACCAGGGACTATTTGATGGCATTCTACCAG

CAAACAGAACTCCGACCTAAACGGATCATATTCTACAGGGATGGAGTCAGTGAAGGTCAGTTTAATCAGG

TCTTGCTGAAAGAGATGGATGCCATTAGGACGGTTGATGTGTTTTATTTTTTGTCTGCCAGGCGCTGCAA

TCAAATTATTTACAATGGCAGGAAATCAACTGATCGGAGTGGCAACATATCACCTGGTACTGTTGTAGAT

ATGACCATCTGCCATCCCACTGAGTTTGACTTTTATTTGTGTAGCCATGCTGGTATACAGGTAATCATCT

TAATACAAGACCACATGGATTTTGTACAGGCAGCAGTAATAAATGCATGTGTTGTGTGGCAACACAATAG

AGATACAAATGTACATATTTATTAATATACAAATAGAGAAACAAATGTAAAGATCATAATCAAAATAAAC

ACAAATACACTCATATTAAAAAACTAACATGAACCAATGAGATATAGAAACCATTAAACATCAACTAGTT

TATTCAATCTACCAAAACATCTGAGTAAAGGAAATCCAGATCTGTTCATATTTTACCGGTAACAATTAGC

TAGTTAGGATATTAAATAAGAACAGAAAATTTATGCTCACTAGTTCTCCACGAATTTAGGAAGCTTGTTG

ATTGAATATCATATACTAAAACACACAAATTCTACTATACATACTGTGACTATTTTAATGTCATTAGCCT

AATTCATAACTTACGAAAAATGCTTATGGAGTATGTAGATTCAGGATAGGCAAATAAAAGGATCAGGGAA

CATGTAAGCTAACAAACACTGCATTGTATCTTGTTGAATAATGAATTTGTTTAAAATAAAACTTGCTCTG

ACGCACGTGATCTACGATGGAGTTATATATGCATGCCATTGCATTTACATGTATAGGTTTCCCTATGAAA

TTTATTCAATGTGATATCTTAATGGACAAAATTTAATATTTTAATATTCTCTTATCTGTTAATGCATTCA

TAGATTATGACACTCAAAATATTTTAATGGGCAATAGGACGGACTTTGACTTTGTTGTCGGCAATGCACA

GACTAATGAGGGTGTTGAAAAGGTGAAACCTCCAACCGGTAGCCCAATATCTATGTTAATGGCTACTCCA

GATGGCTCATATGCATTACCAAGCAGTTGATGTTTTTAAGCTAAATGAGTGTCAGAGATACCATCATATC

TCAGTAAATCAGTGTTTGTCGAGCACAAAATGTTCTTGCCGATGATTAGTTGCCCAAAAATATTGTAAGA

CATACTGTAAAAAGAATTCAAGAGGCCCATAACAGCAGCGGAGATAATAAAGGCCGTGATTCCCAGTTGC

AAGGTCTGCATTTTTTCTGTCTTTGGAATGTTGCTGTCATGGAATGCCCGAGGAAAATATAACTTCGGAA

TCAAGAAGTTTCGAAAGGTAGCTTCGCAAATCAGCCCAACCAAAGCTGC

>MSTRG.236.18 gene=MSTRG.236

CACAGTCCATCTTCATTCAGCTTCAGGCCCAAGCCCAGTTAGTCATTGTTTTCTCCGCTGCCTGTGTGTA

TATGTACGTATACGTGTGAGATGAGGAAGAATCAATGGCGATTCCAAAGAATTAAGATCAATTCTCACAC

TCTGCATTGTTATGATAGTTGTTTCTATTGCTTTTACTCTCAAATTTTAAACAATGAAGAAAATGCTTCA

GAAAGCCGATAAGAATGGTCGAAGAAAGCGCTTAATTAATAAATAAGGTAGTGGCATCAATGGACTGGCC

CGAGGTCACCACATACAAAGAACTCTACACACATGGTCAGTGAGAGGAAATATTAAGGAATCTCTACTGC

ACTTGTGAAACTGAAAGGGGTGTTGTTGCTAGTGGCATGACCAGGGACTATTTGATGGCATTCTACCAGC

AAACAGAACTCCGACCTAAACGGATCATATTCTACAGGGATGGAGTCAGTGAAGGTCAGTTTAATCAGGT

CTTGCTGAAAGAGATGGATGCCATTAGGACGGTTGATGTGTTTTATTTTTTGTCTGCCAGGCGCTGCAAT

CAAATTATTTACAATGGCAGGAAATCAACTGATCGGAGTGGCAACATATCACCTGGTACTGTTGTAAATA

TGACCATCTGCCATCCCACTGAGTTTGACTTTTATTTGTGTAGCCATGCTGGTATACAGGGTGTTGAAAA

GGTGAAACCTCCAACCGGTAGCCCAATATCTATGTTAATGGCTACTCCAGATGGCTCATATGCATTACCA

AGCAGTTGATGTTTTTAAGCTAAATGAGTGTCAGAGATACCATCATATCTCAGTAAATCAGTGTTTGTCG

AGCACAAAATGTTCTTGCCGATGATTAGTTGCCCAAAAAT

>MSTRG.236.19 gene=MSTRG.236

CACAGTCCATCTTCATTCAGCTTCAGGCCCAAGCCCAGTTAGTCATTGTTTTCTCCGCTGCCTGTGTGTA

TATGTACGTATACGTGTGAGATGAGGAAGAATCAATGGCGATTCCAAAGAATTAAGATCAATTCTCACAC

TCTGCATTGTTATGATAGTTGTTTCTATTGCTTTTACTCTCAAATTTTAAACAATGAAGAAAATGCTTCA

GAAAGCCGATAAGAATGGTCGAAGAAAGCGCTTAATTAATAAATAAGGTAGTGGCATCAATGGACTGGCC

CGAGGTCACCACATACAAAGAACTCTACACACATGGTCAGTGAGAGGAAATATTAAGGAATCTCTACTGC

ACTTGTGAAACTGAAAGGGGTGTTGTTGCTAGTGGCATGACCAGGGACTATTTGATGGCATTCTACCAGC

AAACAGAACTCCGACCTAAACGGATCATATTCTACAGGGATGGAGTCAGTGAAGGTCAGTTTAATCAGGT

CTTGCTGAAAGAGATGGATGCCATTAGGACGGTTGATGTGTTTTATTTTTTGTCTGCCAGGCGCTGCAAT

CAAATTATTTACAATGGCAGGAAATCAACTGATCGGAGTGGCAACATATCACCTGGTTACATATTGTTCT

ATTGTATCTTTAAAAATCTTTATTATTCTGATTAGCAAATAACTGGCTTTTGTATACCAGGTACTGTTGT

AAATATGACCATCTGCCATCCCACTGAGTTTGACTTTTATTTGTGTAGCCATGCTGGTATACAGATTATG

ACACTCAAAATATTTTAATGGGCAATAGGACGGACTTTGACTTTGTTGTCGGCAATGCACAGACTAATGA

GGTTGGTTGGATTAAATCTCTTAGGTGTTTACCATATTGTTGCTTAATAATGCATTTGAAGGATGGGAGG

TGATCTTTACTTGATGTAGCAATGAAGATGAAAAACAGATCACCTATTTGTAATATTGATATGCGGATTA

TGCTTGTCTGTGCTCTATTATGCTTATTTTTGCTCTAATGTTGGATGTTAATATGCTTCCATTATCCTAC

AAATGGAGTTAGGGTGTTGAAAAGGTGAAACCTCCAACCGGTAGCCCAATATCTATGTTAATGGCTACTC

CAGATGGCTCATATGCATTACCAAGCAGTTGATGTTTTTAAGCTAAATGAGTGTCAGAGATACCATCATA

TCTCAGTAAATCAGTGTTTGTCGAGCACAAAATGTTCTTGCCGATGATTAGTTGCCCAAAAAT

>MSTRG.236.20 gene=MSTRG.236

CACAGTCCATCTTCATTCAGCTTCAGGCCCAAGCCCAGTTAGTCATTGTTTTCTCCGCTGCCTGTGTGTA

TATGTACGTATACGTGTGAGATGAGGAAGAATCAATGGCGATTCCAAAGAATTAAGATCAATTCTCACAC

TCTGCATTGTTATGATAGTTGTTTCTATTGCTTTTACTCTCAAATTTTAAACAATGAAGAAAATGCTTCA

GAAAGCCGATAAGAATGGTCGAAGAAAGCGCTTAATTAATAAATAAGGTAGTGGCATCAATGGACTGGCC

CGAGGTCACCACATACAAAGAACTCTACACACATGGTCAGTGAGAGGAAATATTAAGGAATCTCTACTGC

ACTTGTGAAACTGAAAGGGGTGTTGTTGCTAGTGGCATGACCAGGGACTATTTGATGGCATTCTACCAGC

AAACAGAACTCCGACCTAAACGGATCATATTCTACAGGGATGGAGTCAGTGAAGGTCAGTTTAATCAGGT

CTTGCTGAAAGAGATGGATGCCATTAGGACGGTTGATGTGTTTTATTTTTTGTCTGCCAGGCGCTGCAAT

CAAATTATTTACAATGGCAGGAAATCAACTGATCGGAGTGGCAACATATCACCTGGTTACATATTGTTCT

ATTGTATCTTTAAAAATCTTTATTATTCTGATTAGCAAATAACTGGCTTTTGTATACCAGGTACTGTTGT

AGATATGACCATCTGCCATCCCACTGAGTTTGACTTTTATTTGTGTAGCCATGCTGGTATACAGGGTGTT

GAAAAGGTGAAACCTCCAACCGGTAGCCCAATATCTATGTTAATGGCTACTCCAGATGGCTCATATGCAT

TACCAAGCAGTTGATGTTTTTAAGCTAAATGAGTGTCAGAGATACCATCATATCTCAGTAAATCAGTGTT

TGTCGAGCACAAAATGTTCTTGCCGATGATTAGTTGCCCAAAAAT

>MSTRG.236.21 gene=MSTRG.236

CATCTTCATTCAGCTTCAGGCCCAAGCCCAGTTAGTCATTGTTTTCTCCGCTGCCTGTGTGTATATGTAC

GTATACGTGTGAGATGAGGAAGAATCAATGGCGATTCCAAAGAATTAAGATCAATTCTCACACTCTGCAT

TGTTATGATAGTTGTTTCTATTGCTTTTACTCTCAAATTTTAAACAATGAAGAAAATGCTTCAGAAAGCC

GATAAGAATGGTCGAAGAAAGCGCTTAATTAATAAATAAGGTAGTGGCATCAATGGACTGGCCCGAGGTC

ACCACATACAAAGAACTCTACACACATGGTCAGTGAGAGGAAATATTAAGGAATCTCTACTGCACTTGTG

AAACTGAAAGGGGTGTTGTTGCTAGTGGCATGACCAGGGACTATTTGATGGCATTCTACCAGCAAACAGA

ACTCCGACCTAAACGGATCATATTCTACAGGGATGGAGTCAGTGAAGGTCAGTTTAATCAGGTCTTGCTG

AAAGAGATGGATGCCATTAGGACGGTTGATGTGTTTTACACACATGGTCAGTGAGAGGAAATATTAAGGA

ATCTCTACTGCACTTGTGAAACTGAAAGGGGTGTTGTTGCTAGTGGCATGACCAGGGACTATTTGATGGC

ATTCTACCAGCAAACAGAACTCCGACCTAAACGGATCATATTCTACAGGGATGGAGTCAGTGAAGGTCAG

TTTAATCAGGTCTTGCTGAAAGAGATGGATGCCATTAGGACGGTTGATGTGTTTTATTTTTTGTCTGCCA

GGCGCTGCAATCAAATTATTTACAATGGCAGGAAATCAACTGATCGGAGTGGCAACATATCACCTGGTTA

CATATTGTTCTATTGTATCTTTAAAAATCTTTATTATTCTGATTAGCAAATAACTGGCTTTTGTATACCA

GGTACTGTTGTAGATATGACCATCTGCCATCCCACTGAGTTTGACTTTTATTTGTGTAGCCATGCTGGTA

TACAGGGTGTTGAAAAGGTGAAACCTCCAACCGGTAGCCCAATATCTATGTTAATGGCTACTCCAGATGG

CTCATATGCATTACCAAGCAGTTGATGTTTTTAAGCTAAATGAGTGTCAGAGATACCATCATATCTCAGT

AAATCAGTGTTTGTCGAGCACAAAATGTTCTTGCCGATGATTAGTTGCCC

>MSTRG.236.22 gene=MSTRG.236

CACAGTCCATCTTCATTCAGCTTCAGGCCCAAGCCCAGTTAGTCATTGTTTTCTCCGCTGCCTGTGTGTA

TATGTACGTATACGTGTGAGATGAGGAAGAATCAATGGCGATTCCAAAGAATTAAGATCAATTCTCACAC

TCTGCATTGTTATGATAGTTGTTTCTATTGCTTTTACTCTCAAATTTTAAACAATGAAGAAAATGCTTCA

GAAAGCCGATAAGAATGGTCGAAGAAAGCGCTTAATTAATAAATAAGGTAGTGGCATCAATGGACTGGCC

CGAGGTCACCACATACAAAGAACTCTACACACATGGTCAGTGAGAGGAAATATTAAGGAATCTCTACTGC

ACTTGTGAAACTGAAAGGGGTGTTGTTGCTAGTGGCATGACCAGGGACTATTTGATGGCATTCTACCAGC

AAACAGAACTCCGACCTAAACGGATCATATTCTACAGGGATGGAGTCAGTGAAGGTCAGTTTAATCAGGT

CTTGCTGAAAGAGATGGATGCCATTAGGACGGTTGATGTGTTTTATTTTTTGTCTGCCAGGCGCTGCAAT

CAAATTATTTACAATGGCAGGAAATCAACTGATCGGAGTGGCAACATATCACCTGGTACTGTTGTAAATA

TGACCATCTGCCATCCCACTGAGTTTGACTTTTATTTGTGTAGCCATGCTGGTATACAGGGTGTTGAAAA

GGTGAAACCTCCAACCGGTAGCCCAATATCTATGTTAATGGCTACTCCAGATGGCTCATATGCATTACCA

AGCAGTTGATGTTTTTAAGCTAAATGAGTGTCAGAGATACCATCATATCTCAGTAAATCAGTGTTTGTCG

AGCACAAAATGTTCTTGCCGATGATTAGTTGCCC

>MSTRG.236.23 gene=MSTRG.236

CACAGTCCATCTTCATTCAGCTTCAGGCCCAAGCCCAGTTAGTCATTGTTTTCTCCGCTGCCTGTGTGTA

TATGTACGTATACGTGTGAGATGAGGAAGAATCAATGGCGATTCCAAAGAATTAAGATCAATTCTCACAC

TCTGCATTGTTATGATAGTTGTTTCTATTGCTTTTACTCTCAAATTTTAAACAATGAAGAAAATGCTTCA

GAAAGCCGATAAGAATGGTCGAAGAAAGCGCTTAATTAATAAATAAGGTAGTGGCATCAATGGACTGGCC

CGAGGTCACCACATACAAAGAACTCTACACACATGGTCAGTGAGAGGAAATATTAAGGAATCTCTACTGC

ACTTGTGAAACTGAAAGGGGTGTTGTTGCTAGTGGCATGACCAGGGACTATTTGATGGCATTCTACCAGC

AAACAGAACTCCGACCTAAACGGATCATATTCTACAGGGATGGAGTCAGTGAAGGTCAGTTTAATCAGGT

CTTGCTGAAAGAGATGGATGCCATTAGGACGGTTGATGTGTTTTATTTTTTGTCTGCCAGGCGCTGCAAT

CAAATTATTTACAATGGCAGGAAATCAACTGATCGGAGTGGCAACATATCACCTGGTACTGTTGTAAATA

TGACCATCTGCCATCCCACTGAGTTTGACTTTTATTTGTGTAGCCATGCTGGTATACAGGGTGTTGAAAA

GGTGAAACCTCCAACCGGTAGCCCAATATCTATGTTAATGGCTACTCCAGATGGCTCATATGCATTACCA

AGCAGTTGATGTTTTTAAGCTAAATGAGTGTCAGAGATACCATCATATCTCAGTAAATCAGTGTTTGTCG

AGCACAAAATGTTCTTGCCGATGATTAGTTGCCC

>MSTRG.237.1 gene=MSTRG.237

ATTGAGTTGTGTTTTGGAGCCATTTGATATCACGTGATGCTGTCTGATTTCTTCCTACTTCACATCACTT

TCCACCGCAGTGGTTTAGACAGTTTCGGCAAAGAGACAACAAGCTCATGGCTCCAAAATCCCCACGAGCT

GAATCTTTTCCTCTTCAATCCATTTCTTACAGGTCAGTTTGAATTTTTGTTCAATTTGAATTCAAAACC

>MSTRG.236.24 gene=MSTRG.236

GCACAGTCCATCTTCATTCAGCTTCAGGCCCAAGCCCAGTTAGTCATTGTTTTCTCCGCTGCCTGTGTGT

ATATGTACGTATACGTGTGAGATGAGGAAGAATCAATGGCGATTCCAAAGAATTAAGATCAATTCTCACA

CTCTGCATTGTTATGATAGTTGTTTCTATTGCTTTTACTCTCAAATTTTAAACAATGAAGAAAATGCTTC

AGAAAGCCGATAAGAATGGTCGAAGAAAGCGCTTAATTAATAAATAAGGTAGTGGCATCAATGGACTGGC

CCGAGGTCACCACATACAAAGAACTCTACACACATGGTCAGTGAGAGGAAATATTAAGGAATCTCTACTG

CACTTGTGAAACTGAAAGGGGTGTTGTTGCTAGTGGCATGACCAGGGACTATTTGATGGCATTCTACCAG

CAAACAGAACTCCGACCTAAACGGATCATATTCTACAGGGATGGAGTCAGTGAAGGTCAGTTTAATCAGG

TCTTGCTGAAAGAGATGGATGCCATTAGGACGGTTGATGTGTTTTATTTTTTGTCTGCCAGGCGCTGCAA

TCAAATTATTTACAATGGCAGGAAATCAACTGATCGGAGTGGCAACATATCACCTGGTACTGTTGTAAAT

ATGACCATCTGCCATCCCACTGAGTTTGACTTTTATTTGTGTAGCCATGCTGGTATACAGGGTGTTGAAA

AGGTGAAACCTCCAACCGGTAGCCCAATATCTATGTTAATGGCTACTCCAGATGGCTCATATGCATTACC

AAGCAGTTGATGTTTTTAAGCTAAATGAGTGTCAGGGATACCATCATATCTCAGTAAATCAGTGTTTGTC

GAGCACAAATTGTTCTTGCCGATGATTAGTTGCCCAAAAATACTGTAAGACATACTGTAAAAAGAATTCA

AGAGGCCGATAACAGCAGCGGAGATAATAAAGGCCGTGATTCCCAGTTGCAAGGTCTGCAATTTTTTCTG

TCTTCGAGTCTTTGGAATGTTGCTGTTATGGAATACCCCAGGAAAATATAACTTCGGAATCAAGAAGTTT

CGAAAGGTAGCTTGGCAAATCAGCCCAACCAAAGCTGCTCAGAAGTACAACTTAGATACAAGTTGTTTTT

TCAAATATGTGTGAGTCCTTGTATTGATATCTATCAGGCTAACGAGTGATGCCACTATGGATATGATCTT

GTAGTGAAATTTATATATACATTCATTTATGCACGTATGTATATTAATGTGTTAACTGTACCAAATATTT

ATCATTTAATATTTTTATTCACGGCAATGTTGGTTTTAACCGATATTAAGAAAAT

>MSTRG.236.25 gene=MSTRG.236

GCACAGTCCATCTTCATTCAGCTTCAGGCCCAAGCCCAGTTAGTCATTGTTTTCTCCGCTGCCTGTGTGT

ATATGTACGTATACGTGTGAGATGAGGAAGAATCAATGGCGATTCCAAAGAATTAAGATCAATTCTCACA

CTCTGCATTGTTATGATAGTTGTTTCTATTGCTTTTACTCTCAAATTTTAAACAATGAAGAAAATGCTTC

AGAAAGCCGATAAGAATGGTCGAAGAAAGCGCTTAATTAATAAATAAGGTAGTGGCATCAATGGACTGGC

CCGAGGTCACCACATACAAAGAACTCTACACACATGGTCAGTGAGAGGAAATATTAAGGAATCTCTACTG

CACTTGTGAAACTGAAAGGGGTGTTGTTGCTAGTGGCATGACCAGGGACTATTTGATGGCATTCTACCAG

CAAACAGAACTCCGACCTAAACGGATCATATTCTACAGGGATGGAGTCAGTGAAGGTCAGTTTAATCAGG

TCTTGCTGAAAGAGATGGATGCCATTAGGACGGTTGATGTGTTTTATTTTTTGTCTGCCAGGCGCTGCAA

TCAAATTATTTACAATGGCAGGAAATCAACTGATCGGAGTGGCAACATATCACCTGGTTACATATTGTTC

TATTGTATCTTTAAAAATCTTTATTATTCTGATTAGCAAATAACTGGCTTTTGTATACCAGGTACTGTTG

TAGATATGACCATCTGCCATCCCACTGAGTTTGACTTTTATTTGTGTAGCCATGCTGGTATACAGGGTGT

TGAAAAGGTGAAACCTCCAACCGGTAGCCCAATATCTATGTTAATGGCTACTCCAGATGGCTCATATGCA

TTACCAAGCAGTTGATGTTTTTAAGCTAAATGAGTGTCAGGGATACCATCATATCTCAGTAAATCAGTGT

TTGTCGAGCACAAATTGTTCTTGCCGATGATTAGTTGCCCAAAAATACTGTAAGACATACTGTAAAAAGA

ATTCAAGAGGCCGATAACAGCAGCGGAGATAATAAAGGCCGTGATTCCCAGTTGCAAGGTCTGCAATTTT

TTCTGTCTTCGAGTCTTTGGAATGTTGCTGTTATGGAATACCCCAGGAAAATATAACTTCGGAATCAAGA

AGTTTCGAAAGGTAGCTTGGCAAATCAGCCCAACCAAAGCTGCTCAGAAGTACAACTTAGATACAAGTTG

TTTTTTCAAATATGTGTGAGTCCTTGTATTGATATCTATCAGGCTAACGAGTGATGCCACTATGGATATG

ATCTTGTAGTGAAATTTATATATACATTCATTTATGCACGTATGTATATTAATGTGTTAACTGTACCAAA

TATTTATCATTTAATATTTTTATTCACGGC

>MSTRG.236.26 gene=MSTRG.236

AAAATGCCATCTGTACCCGCACAGTCCATCTTCATTCAGCTTCAGGCCCAAGCCCAGTTAGTCATTGTTT

TCTCCGCTGCCTGTGTGTATATGTACGTATACGTGTGAGATGAGGAAGAATCAATGGCGATTCCAAAGAA

TTAAGATCAATTCTCACACTCTGCATTGTTATGATAGTTGTTTCTATTGCTTTTACTCTCAAATTTTAAA

CAATGAAGAAAATGCTTCAGAAAGCCGATAAGAATGGTCGAAGAAAGCGCTTAATTAATAAATAAGGTAG

TGGCATCAATGGACTGGCCCGAGGTCACCACATACAAAGAACTCTACACACATGGTCAGTGAGAGGAAAT

ATTAAGGAATCTCTACTGCACTTGTGAAACTGAAAGGGGTGTTGTTGCTAGTGGCATGACCAGGGACTAT

TTGATGGCATTCTACCAGCAAACAGAACTCCGACCTAAACGGATCATATTCTACAGGGATGGAGTCAGTG

AAGGTCAGTTTAATCAGGTCTTGCTGAAAGAGATGGATGCCATTAGGACGGTTGATGTGTTTTATTTTTT

GTCTGCCAGGCGCTGCAATCAAATTATTTACAATGGCAGGAAATCAACTGATCGGAGTGGCAACATATCA

CCTGGTTACATATTGTTCTATTGTATCTTTAAAAATCTTTATTATTCTGATTAGCAAATAACTGGCTTTT

GTATACCAGGTACTGTTGTAGATATGACCATCTGCCATCCCACTGAGTTTGACTTTTATTTGTGTAGCCA

TGCTGGTATACAGATTATGACACTCAAAATATTTTAATGGGCAATAGGACGGACTTTGACTTTGTTGTCG

GCAATGCACAGACTAATGAGGGTGTTGAAAAGGTGAAACCTCCAACCGGTAGCCCAATATCTATGTTAAT

GGCTACTCCAGATGGCTCATATGCATTACCAAGCAGTTGATGTTTTTAAGCTAAATGAGTGTCAGGGATA

CCATCATATCTCAGTAAATCAGTGTTTGTCGAGCACAAATTGTTCTTGCCGATGATTAGTTGCCCAAAAA

TACTGTAAGACATACTGTAAAAAGAATTCAAGAGGCCGATAACAGCAGCGGAGATAATAAAGGCCGTGAT

TCCCAGTTGCAAGGTCTGCAATTTTTTCTGTCTTCGAGTCTTTGGAATGTTGCTGTTATGGAATACCCCA

GGAAAATATAACTTCGGAATCAAGAAGTTTCGAAAGGTAGCTTGGCAAATCAGCCCAACCAAAGCTGCTC

AGAAGTACAACTTAGATACAAGTTGTTTTTTCAAATATGTGTGAGTCCTTGTATTGATATCTATCAGGCT

AACGAGTGATGCCACTATGGATATGATCTTGTAGTGAAATTTATATATACATTCATTTATGCACGTATGT

ATATTAATGTGTTAACTGTACCAAATATTTATCATTTAATATTTTTATTCACG

>MSTRG.236.27 gene=MSTRG.236

CATCTTCATTCAGCTTCAGGCCCAAGCCCAGTTAGTCATTGTTTTCTCCGCTGCCTGTGTGTATATGTAC

GTATACGTGTGAGATGAGGAAGAATCAATGGCGATTCCAAAGAATTAAGATCAATTCTCACACTCTGCAT

TGTTATGATAGTTGTTTCTATTGCTTTTACTCTCAAATTTTAAACAATGAAGAAAATGCTTCAGAAAGCC

GATAAGAATGGTCGAAGAAAGCGCTTAATTAATAAATAAGTGGCATCAATGGACTGGCCCGAGGTCACCA

CATACAAAGAACTCTACACACATGGTCAGTGAGAGGAAATATTAAGGAATCTCTACTGCACTTGTGAAAC

TGAAAGGGGTGTTGTTGCTAGTGGCATGACCAGGGACTATTTGATGGCATTCTACCAGCAAACAGAACTC

CGACCTAAACGGATCATATTCTACAGGGATGGAGTCAGTGAAGGTCAGTTTAATCAGGTCTTGCTGAAAG

AGATGGATGCCATTAGGACGGTTGATGTGTTTTATTTTTTGTCTGCCAGGCGCTGCAATCAAATTATTTA

CAATGGCAGGAAATCAACTGATCGGAGTGGCAACATATCACCTGGTACTGTTGTAAATATGACCATCTGC

CATCCCACTGAGTTTGACTTTTATTTGTGTAGCCATGCTGGTATACAGATTATGACACTCAAAATATTTT

AATGGGCAATAGGACGGACTTTGACTTTGTTGTCGGCAATGCACAGACTAATGAGGTTGGTTGGATTAAA

TCTCTTAGGTGTTTACCATATTGTTGCTTAATAATGCATTTGAAGGATGGGAGGTGATCTTTACTTGATG

TAGCAATGAAGATGAAAAACAGATCACCTATTTGTAATATTGATATGCGGATTATGCTTGTCTGTGCTCT

ATTATGCTTATTTTTGCTCTAATGTTGGATGTTAATATGCTTCCATTATCCTACAAATGGAGTTAGGGTG

TTGAAAAGGTGAAACCTCCAACCGGTAGCCCAATATCTATGTTAATGGCTACTCCAGATGGCTCATATGC

ATTACCAAGCAGTTGATGTTTTTAAGCTAAATGAGTGTCAGGGATACCATCATATCTCAGTAAATCAGTG

TTTGTCGAGCACAAATTGTTCTTGCCGATGATTAGTTGCCCAAAAATACTGTAAGACATACTGTAAAAAG

AATTCAAGAGGCCGATAACAGCAGCGGAGATAATAAAGGCCGTGATTCCCAGTTGCAAGGTCTGCAATTT

TTTCTGTCTTCGAGTCTTTGGAATGTTGCTGTTATGGAATACCCCAGGAAAATATAACTTCGGAATCAAG

AAGTTTCGAAAGGTAGCTTGGCAAATCAGCCCAACCAAAGCTGCTCAGAAGTACAACTTAGATACAAGTT

GTTTTTTCAAATATGTGTGAGTCCTTGTATTGATATCTATCAGGCTAACGAGTGATGCCACTATGGATAT

GATCTT

>MSTRG.236.28 gene=MSTRG.236

CATCTTCATTCAGCTTCAGGCCCAAGCCCAGTTAGTCATTGTTTTCTCCGCTGCCTGTGTGTATATGTAC

GTATACGTGTGAGATGAGGAAGAATCAATGGCGATTCCAAAGAATTAAGATCAATTCTCACACTCTGCAT

TGTTATGATAGTTGTTTCTATTGCTTTTACTCTCAAATTTTAAACAATGAAGAAAATGCTTCAGAAAGCC

GATAAGAATGGTCGAAGAAAGCGCTTAATTAATAAATAAGTGGCATCAATGGACTGGCCCGAGGTCACCA

CATACAAAGAACTCTACACACATGGTCAGTGAGAGGAAATATTAAGGAATCTCTACTGCACTTGTGAAAC

TGAAAGGGGTGTTGTTGCTAGTGGCATGACCAGGGACTATTTGATGGCATTCTACCAGCAAACAGAACTC

CGACCTAAACGGATCATATTCTACAGGGATGGAGTCAGTGAAGGTCAGTTTAATCAGGTCTTGCTGAAAG

AGATGGATGCCATTAGGACGGTTGATGTGTTTTATTTTTTGTCTGCCAGGCGCTGCAATCAAATTATTTA

CAATGGCAGGAAATCAACTGATCGGAGTGGCAACATATCACCTGGTACTGTTGTAAATATGACCATCTGC

CATCCCACTGAGTTTGACTTTTATTTGTGTAGCCATGCTGGTATACAGGGTGTTGAAAAGGTGAAACCTC

CAACCGGTAGCCCAATATCTATGTTAATGGCTACTCCAGATGGCTCATATGCATTACCAAGCAGTTGATG

TTTTTAAGCTAAATGAGTGTCAGGGATACCATCATATCTCAGTAAATCAGTGTTTGTCGAGCACAAATTG

TTCTTGCCGATGATTAGTTGCCCAAAAATACTGTAAGACATACTGTAAAAAGAATTCAAGAGGCCGATAA

CAGCAGCGGAGATAATAAAGGCCGTGATTCCCAGTTGCAAGGTCTGCAATTTTTTCTGTCTTCGAGTCTT

TGGAATGTTGCTGTTATGGAATACCCCAGGAAAATATAACTTCGGAATCAAGAAGTTTCGAAAGGTAGCT

TGGCAAATCAGCCCAACCAAAGCTGCTCAGAAGTACAACTTAGATACAAGTTGTTTTTTCAAATATGTGT

GAGTCCTTGTATTGATATCTATCAGGCTAACGAGTGATGCCACTATGGATATGATCTT

>MSTRG.236.29 gene=MSTRG.236

CACAGTCCATCTTCATTCAGCTTCAGGCCCAAGCCCAGTTAGTCATTGTTTTCTCCGCTGCCTGTGTGTA

TATGTACGTATACGTGTGAGATGAGGAAGAATCAATGGCGATTCCAAAGAATTAAGATCAATTCTCACAC

TCTGCATTGTTATGATAGTTGTTTCTATTGCTTTTACTCTCAAATTTTAAACAATGAAGAAAATGCTTCA

GAAAGCCGATAAGAATGGTCGAAGAAAGCGCTTAATTAATAAATAAGGTAGTGGCATCAATGGACTGGCC

CGAGGTCACCACATACAAAGAACTCTACACACATGGTCAGTGAGAGGAAATATTAAGGAATCTCTACTGC

ACTTGTGAAACTGAAAGGGGTGTTGTTGCTAGTGGCATGACCAGGGACTATTTGATGGCATTCTACCAGC

AAACAGAACTCCGACCTAAACGGATCATATTCTACAGGGATGGAGTCAGTGAAGGTCAGTTTAATCAGGT

CTTGCTGAAAGAGATGGATGCCATTAGGACGGTTGATGTGTTTTATTTTTTGTCTGCCAGGCGCTGCAAT

CAAATTATTTACAATGGCAGGAAATCAACTGATCGGAGTGGCAACATATCACCTGGTTACATATTGTTCT

ATTGTATCTTTAAAAATCTTTATTATTCTGATTAGCAAATAACTGGCTTTTGTATACCAGGTACTGTTGT

AAATATGACCATCTGCCATCCCACTGAGTTTGACTTTTATTTGTGTAGCCATGCTGGTATACAGGGTGTT

GAAAAGGTGAAACCTCCAACCGGTAGCCCAATATCTATGTTAATGGCTACTCCAGATGGCTCATATGCAT

TACCAAGCAGTTGATGTTTTTAAGCTAAATGAGTGTCAGGGATACCATCATATCTCAGTAAATCAGTGTT

TGTCGAGCACAAATTGTTCTTGCCGATGATTAGTTGCCCAAAAATACTGTAAGACATACTGTAAAAAGAA

TTCAAGAGGCCGATAACAGCAGCGGAGATAATAAAGGCCGTGATTCCCAGTTGCAAGGTCTGCAATTTTT

TCTGTCTTCGAGTCTTTGGAATGTTGCTGTTATGGAATACCCCAGGAAAATATAACTTCGGAATCAAGAA

GTTTCGAAAGGTAGCTTGGCAAATCAGCCCAACCAAAGCTGCTCAGAAGTACAACTTAGATACAAGTTGT

TTTTTCAAATATGTGTGAGTCCTTGTATTGATATCTATCAGGCTAACGAGTGATGCCACTATGGA

>MSTRG.236.30 gene=MSTRG.236

GCACAGTCCATCTTCATTCAGCTTCAGGCCCAAGCCCAGTTAGTCATTGTTTTCTCCGCTGCCTGTGTGT

ATATGTACGTATACGTGTGAGATGAGGAAGAATCAATGGCGATTCCAAAGAATTAAGATCAATTCTCACA

CTCTGCATTGTTATGATAGTTGTTTCTATTGCTTTTACTCTCAAATTTTAAACAATGAAGAAAATGCTTC

AGAAAGCCGATAAGAATGGTCGAAGAAAGCGCTTAATTAATAAATAAGGTAGTGGCATCAATGGACTGGC

CCGAGGTCACCACATACAAAGAACTCTACACACATGGTCAGTGAGAGGAAATATTAAGGAATCTCTACTG

CACTTGTGAAACTGAAAGGGGTGTTGTTGCTAGTGGCATGACCAGGGACTATTTGATGGCATTCTACCAG

CAAACAGAACTCCGACCTAAACGGATCATATTCTACAGGGATGGAGTCAGTGAAGGTCAGTTTAATCAGG

TCTTGCTGAAAGAGATGGATGCCATTAGGACGGTTGATGTGTTTTATTTTTTGTCTGCCAGGCGCTGCAA

TCAAATTATTTACAATGGCAGGAAATCAACTGATCGGAGTGGCAACATATCACCTGGTACTGTTGTAGAT

ATGACCATCTGCCATCCCACTGAGTTTGACTTTTATTTGTGTAGCCATGCTGGTATACAGGTAATCATCT

TAATACAAGACTACATGGATTTTGTACAGGCAGCAGTAATAAATGCATGTGTTGTGTGGCAACACAATAG

AGATACAAATGTACATATTTATTAATATACAAATAGAGAAACAAATGTAAAGATCT

>MSTRG.240.1 gene=MSTRG.240

ATGTAGGAGGGCCAAAGTCCCGGCCTTACAAAATCAATCATTGTCAGTAATTTCAGTTGCAAGGAGATTG

CTGATGTTCTGGCATTTTCCAGGATTCCTTCTGCAATCAATCAATTTTATCAAGTTAGAGAGGGGTCTCC

TGTTTCTTTTGTTGCTGGTTCTTCAAACAAACATGGAAAGGGACAATCGACTTCGCCTGATTTGCCAGCG

GTGT

>MSTRG.241.1 gene=MSTRG.241

AGAAAATCCAAGACATACTAAGTTGTACAGAAATTTTAAGTAAAAGATGCCTATCCCAGAAATTTGTTTG

AGCTCCGGAGATGCGAAAGCTATGCCAATTATGGGACTGGGATTAGGAGTTCCTGATCTAACACCATAAG

TTATGAATAATACGAAGAAAGTTGTGCTTGACGCGATTGAGCTTGGTTACAGGATATCAAATCTGTTTGG

ACAGCAATGTAGGAGTGCCAAAGTCCCGGCCTTATAAAATCAATCATTCTCAGTAATTTCAGTT

>MSTRG.244.1 gene=MSTRG.244

TATGGACTTGAACTCTTCTCTGCATGCCAGCATTGTTGAAATTAAGAAAGGTCTCTTCTTGAAATTCTAC

TTAACTCTAGCATTTTGTATGCATATTCTTGACACTAGTGTAATAAATAGAACTGTTATCAGTATATTCT

TTTTATCGAAAATGATGTGATCAATTTGTTATTTATTTCATCTTTTGCTAGTTTTGTTGTTCTTCAAACC

ATAGTTTTCATGTGATTGTTTGCAAGTGATAACTAATAATCATGGTCCATGTGGCAGTAATTTTTTTAAA

TTGCCGTAGATTAATTTAGCAGTTGAAGGAGCAAGACACACAGTGTGGCATTGAATCGAGCTTGGGGGAG

GCCCTTTATCCAGGTTTAGGATGCAAGCCAAGGGAGAGAGATGCTTTATGGAGAAAGTATGGAATTGGTA

GTCTGAGTTACTGTTGGCCTTTTCATCTTGTTGGGGATTTGTGTTCAGAATGAACAAGCGGGAGTCGTGA

GATTCTAGCATGACAATAGACTACACTGATTTTCGGTATATTAAAAACATTAAGAATGTTGATAACAGAC

TAGTACAATATAAGGCATTTGCATCTGGCAAATTTACGTGAGAGTTTTTGAAGAGGTTGATGAAGTTCAC

ATTGTGAAATTGTATATGGTACTTACTAATTCATAATCTTATTTTGATGCTGATCTCATGTTAAATAATG

TGAATGCTTTTTGTTACAGATTCGGCATCTACTATATTTCATGTTGATATATGTGCCGTTTCAAAACTTC

AGGCCCCTGTGATATATATTCAGTTTAGAGTTTTAGCAGAGTTGCATTTGTAGCCAGTGGCAGTTCTCAG

AGAGGCCGTGCAGAAATGTGGTGGAAGGATGACAAATC

>MSTRG.244.2 gene=MSTRG.244

TATGGACTTGAACTCTTCTCTGCATGCCAGCATTGTTGAAATTAAGAAAGGAATTGGTAGTCTGAGTTAC

TGTTGGCCTTTTCATCTTGTTGGGGATTTGTGTTCAGAATGAACAAGCGGGAGTCGTGAGATTCTAGCAT

GACAATAGACTACACTGATTTTCGGTATATTAAAAACATTAAGAATGTTGATAACAGACTAGTACAATAT

AAGGCATTTGCATCTGGCAAATTTACGTGAGAGTTTTTGAAGAGGTTGATGAAGTTCACATTGTGAAATT

GTATATGGTACTTACTAATTCATAATCTTATTTTGATGCTGATCTCATGTTAAATAATGTGAATGCTTTT

TGTTACAGATTCGGCATCTACTATATTTCATGTTGATATATGTGCCGTTTCAAAAC

>MSTRG.256.1 gene=MSTRG.256

CTAAACATGAGAGCTGCTGTTTCAAATTGGCGCCAAAAAACATTAAACTTGTCTTGTGCTCAAACTCATC

ATCTAGCTGCTTCTGCTGATCAAACAACTCCCAAGACATTCTTCAACTGCGATTCCCATGTTAAACACTT

GTGTAATGACAGGAAATTCTCGGAAGCCATTCACATTTTGTGTAAAAACAAGCGATTATCCGAAGCAATT

GAGCTGTTAAGCCGTATTGATCGGCCTTCTCCGGCGATATATTCGACTCTCTTGCAGCTCTGTATACAGC

AGAGAGCGGTTGATTTGGGTAAAAAAGTGCATGATCATGCGGAGAATAATGTGGGTTTTAGAGCTGGGAT

TGTGATATGTAATCGTTTGTTGGATTTGTATTGTAAATGTGGGTGTGTTGGGGATGCGCGGAAGGTGTTT

GATGAAATGTCTGCGAGAGATGTGTGTTCTTGGAATATAATGGTATCTGGGTATGTGAAAGCGGGGGATT

TGGGGGGTGCTAGGGGGTTGTTTGATGAAATGCCGGAGAGGGATTATTTTTCATGGAATGCGATGGTTTC

GGGGTATGTGAGGTATGATCAGGCGGGTGAGGGGTTGAAGTTGTTTAGGGTGATGGAGAAGAATGGGGAT

TTGGGGAGTAGTAAGTTTGTGGTTTCGAGTGCTCTTTCGGCTTGTTCTGCTGCGAGATGTTTGCGTTCTG

GGAAGGAGATTCATGGGCATATAGTGAGGACGGGGTTGGATTCTGATGAAGTGGTGTGGAGTTCGTTGTC

CGATATGTATGGGAAATGTGGTAGTATAGATGATGCAAGGCATATATTTGACAAATCTTTGAATAGAGAT

GTTGTTTCGTGGACAGCAATGATTGATAGGTACTTTGAGGATGGAAGGAAGGAAGAGGGGTTTAAATTAT

TTATGGAGTTGTTAAGTTCGGGGATTAGACCAAATGAGTTTACTTTTGCTGGGGTTTTAAATGCTTGTAC

AAGTCAATCTGCAGAATATCTGGGGAAACAAGTTCATGGCCATATGATTCGGATTGGGTGTGATCCGTCA

TCATTTGCAACTAGCACACTGGTCCACATGTATTCCAAGTTGGGAAACATGGAGAATGCTTATAGGGTTT

TCAAAGACATACTGGAGCCTGACCTGGTTTCATGGACTTCTTTAATCAATGGATATGCTCAGAATGGTCA

ACCTCATGAAGCTCTTCGGTTATTCGAGTTGCTTCTTAAGTCTGGTAATAAACCCGATCACGTTACTTTC

ATTGGAGTTCTCTCTGCTTGCACCCATGCTGGTCTAGTTGACAGAGGGCTAGAGTATTTCCACTCAATAA

AAGAGAAACATGGTTTAAAATACACTGCAGATCACTATGCTTGTGTTGTTGATCTCCTGGGTCGTTCTGG

CAGATTTAAAGAGGCTGAAGATATCATTGTAAGCATGCCCATGAAACCAGATAAGTTTCTTTGGGCTTCC

TTGCTTGGTGGTTGCAGAATTCATGGAAATATAGAATTGGCAAAACGAGCAGCTGAAGCGTTATTTCAGA

TTGAACCTGATAATCCAGCTACTTATGTTACTCTTGCAAATATTTATGCCACTGCTGGTAAATGGGATGA

AGTGGCAAAACTCAGAAAGGCTATGAATGACAAAGGAGTTGTCAAGAAACCAGGTATTAGTTGGACAGAG

ATCAAAGGAAAAGTCCACACTTTCATGGTGGGAGATAAATCCCACCCATATTCTACTAAAATATACGACT

ATCTGTCAGAACTGTTCAGAAGAATGAAGGAAGAAGGGTATAATCCCGAGACAGACTATGTGCTGCATGA

TGTAGAAGACGAACAAAAGGAGCAAAATATATTCTACCATAGTGAGAAGCTCGCAGTTGCATTTGGAATT

ATTGCTACTCCACAGGGAACCACAATTAAAGTTTTTAAGAACTTAAGAACTTGTGTTGATTGCCATACCT

CATTCAAATATATCTCCAAAATTGTTAAGAGAAAAATAATAGTAAGGGATTCAACCCGATTCCATTGTTT

TGAGGGAGGAAGCTGTTCTTGCAAAGACTATTGGTGATTTAGGATATATTAACTTTGAAAGTTATTGGAT

GATTGTTGGTTGAAGTGAGCTATGAACTTATGGGACCTATGGTTTGAACTGTTTGTTGTTTTGTGGTTAC

ATTCGTTCTCTTTTACTTCCTCAAACTTTCAGTTCCTCATTCGTATTAAAAAGTTAATATCATTTATCTC

TTCATCGTCTTTGGAGAGTTCTTCTATAGAGTTTGGCCACGGGCAGCACCATCAAGTAGTTCATGCATGA

GTTAATCTCAGAGTTTTTCACTGTGAGATCAACCTATCCCCACAGCTGGTTCCTCGAGCAGAATTCTATA

CAGTTACTGATAGTATACAGGAGTAGTACAAGCAGATTAATTATTGTAGAAACATTTCTTTATAGAATTG

ATGGTGGCGAAACTATAGACTGCTTCAAGAAACTCTGTAGGTGTATGTCATCAAAAGTTTGAATTGTGTT

CTGAGGCCTGTACATTAGTAATTGTTTAT

>MSTRG.257.1 gene=MSTRG.257

TTACATATATGCATATGCTTATTCATCAACATCTGAAAAACACAGAGCTATCTTTCTTATGATATGGAGC

AACTAGATCAGAATTCAGAGCTTGTTGTTATACCAATAAATCCCTTTCCTTTCGTCTTCCTCGACATAGA

CACAGTCTTTTATAGCACTCCACAGCGATTTGTAAAATATTGTGCCATCATACCGGTAGTACTCTCCGAG

TATCGGTTTGATTGCTCTGTTGGCCTCCATTGCATGGTAATGTGGGATTGATGTGAAGAGATGATGTGCT

ACATGAGTATTGGTTGCTTGATAATAGATTGTGTCAAGGATTCCGTAGTTTCGATCAATGGTAGACAATG

ATCAATGGTAGACAAGAAGTTAGAACAATGGAGAAAATTGTGGTGCTCTTCATTCTCATCGTTACATCAA

GTGTTGTTCAGGGTTGCATGACTTGGAATGAGACGGAAATGTCGCAAACAGTGGCAAAAATAAATCAGAT

CACGGAGAGTTTTATGGTTTTTCCAAAAAGGATTATGTATGTGCATAAGGAGAAAAACACTTGTTCTGGG

TATGAAAAGACAAACTGTGATTGACGCTCATGTATTTTAATTGATAAATAATCATGAAATGCGTATTGAT

AATTAAATCACTTTAGTTTATATTAGTTTGTTTGTGT

>MSTRG.257.2 gene=MSTRG.257

TTACATATATGCATATGCTTATTCATCAACATCTGAAAAACACAGAGCTATCTTTCTTATGATATGGAGC

AACTAGATCAGAATTCAGAGCTTGTTGTTATACCAATAAATCCCTTTCCTTTCGTCTTCCTCGACATAGA

CACAGTCTTTTATAGCACTCCACAGCGATTTGTAAAATATTGTGCCATCATACCGGTAGTACTCTCCGAG

TATCGGTTTGATTGCTCTGTTGGCCTCCATTGCATGGTAATGTGGGATTGATGTGAAGAGATGATGTGCT

ACATGAGTATTGGTTGCTTGATAATAGATTGTGTCAAGGATTCCGTAGTTTCGATCAATGGTAGACAATG

ATCAATGGTAGACAAGAAGTTAGAACAATGGAGAAAATTGTGGTGCTCTTCATTCTCATCGTTACATCAA

GTGTTGTTCAGGGTTGCATGACTTGGAATGAGACGGAAATGTCGCAAACAGTGGCAAAAATAAATCAGAT

CACGGAGAGTTTTATGATTTTTCCAAAAAGGATTATGTATGTGCATAAGGAGAAAAACACTTGTTCTGGG

TATGAAAAGACAAACTGTGATTGACGCTCATGTATTTTAATTGATAAATAATCATGAAATGCGTATTGAT

AATTAAATCACTTTAGTTTATATTA

>MSTRG.258.1 gene=MSTRG.258

AATCATGTCCCACGAAGAATCTTCTCAACAAGCAGCAACACCCCAAGTGGAAAAGGGGCTCTGGCTAGCG

GATGTGTTTAACGTGCTATACAGAGGCTGTGACATGATAATGCCGGAAAAGCAGATCGGCACCATCAAAG

TCAGTGATGATCAGAATGATCATTACTTGTACAAGGTGGTAGATGCAGATATGACTAAGTCATCACTTCA

ACAGATAGATGAACTTATGTTGAATGGTCCTGAGATATCATCAATCTCAGCAGAGGATTCCCTGAAAATT

GATGTTGATCTTTTTTGTGGAGCTTTCAAGGACACTTTATATATCGATGATTGCCCCAGCGATGATTCGA

TTGAGACGGATTCTCCCCTCGAAATGAAAATCGTATCAGAAGATGGCAAAGGAGAGATTTATCTTCTGTA

TGCAATATTTGATAATGCTGTAGAAGCCCATTTGGAGGTTAAGCTGTTCGCTACTTTTGAGGTTTATGGA

TGTATTGCTGCGAGTACAAGCAAAATTGAAGTCCCCGGCTATGCACATATGCTCTTTTTAGAGAGACCTT

ACAGTAAAACAAAAGCAGGACCCCTTAATCCCCCTAATCTATTACTGCATTCCAAATCCGTAGTAGCTGT

GCCGTTGGAGTCTGAGTTGCTTGTGGACATTCACTTGATGAGGGGTGATGAGATTGATATTCATGAAGGC

ACCGTTAGTTTTGTGGCTAAACGGACAGGTATCAGTACAGAAGTCGTGAGGGGTGCAAAATGGAAAATTG

AATTGGAAATCTGCTGGAAGTGTGGGACATGAGCCGTGGCAGCCTAGAAATTATATTTAGACTAGGTGCT

TAAATTCTCCACTGTAACGAGATAGTAAGTAGGGATGCATATATTTATGCCAAGAGTTTCTAATGTTTTT

GGACTGAAGTACTGTATGGTTTTTGTTGTACCTTGTACGGATCCATCTTAAAAACTCATTAGAAATTGGA

CATGGCATAAACCAGCTTCAAATGTTAAAAGTAATATGTTAC

>MSTRG.259.1 gene=MSTRG.259

TCTTTCAGCTTCCTCAAAACCAGAGAGAGAGAGAGAGAGCGAGAGAGGTAGAAATCATAGAATCCGTGAG

GCTATGGAAGACAGGGAAACCAGAGATTTTAGGCCAAGAAAGAGAGACCGTGGAGGAGAATCGAGTCTGC

CTCCAATGGATATCTACTGCTCACGCTACGTCTCCAAACTTCTCAGCTTCACCGTCAAAAGAAAGCTAAA

AAGACTTGGTGAAGTTACTTCTGTAATTTGCTCCCCTGATCATGAATCTGTTGAGAAGCTGAGGGCTCTC

AAACAATTGGCTTCTTTGATTGTAGAAGACACAAGACTTGGTGATTATCTGGACTCGAAGGTGCTCCCGG

AACTTATTAGCTTCCTCGAGTTTAAAGAACAGAAAACCCCCCTCCAGGCCGATGGTCGGGTTAGGAAAAA

ACCCGTCTTGGAAGATTTAGTTAACAGGAGATTCCAGTACTATTCTGCAATCGTTCTCACGAATATTGTA

TCGTCCAAGAAAGGCTTTAAGTTCAAAGATAAAGCAATCCCGGCTCTTGTGAAACTTATAACTACTTGGC

ATTGGTTCGTGCAGATTCAGGTTCTGTGTGCATTGTCCAATATAGCCAATGTATTTCCTGAAACCTGTGA

TGCCATCATTAAACATGGGGCATTTGAAATCTTGGAGTCTATTATTACAAATGGGAGGAACCACTATGCT

CTGCGAAAAAGTGCCTGCGAGCTTTTGTCAGCCCTTTGTAAACTGTATGTACCCTTTGAGAAGAGAAAGA

TAGTGTTACATACCCTCAAGACAGCAATATTCTCCGAAAGTGAGTCTGTACTAGTACCAACATGCCTTGC

GCTTTCTAATTTCTCCAACAAAAGATTTGTGGATGTTGGTGCTGAAGTATACAAACGTATTCTTCATCTG

ATTGAGTATACAATACCGGAGGTAGCCCTTTCTGCCCTCAGAGTAATTGGAAATCATGTGAGATGGTGTG

ATAATGACCGGCTTCAGTCTATAATTATTGGAGGTTTACCACTTCACCTTATGGGCTTGTTACACCATAA

ATATACGGAAGTAAAGAAGGAGACTTGCTGGATAATTTCAAACATTATTGCAGCTGCAAACAATTCGCAG

TTACAAGTGGTGCTTAACTGTCAGTTGATAATACTTCTCGTCGGATTTGTTCAAAAAAACAAGGTGGAAT

TGATGGAAGTTGCATCCCGTGCAATTTCAAATGCTGTACTTATTAGTGACAAAAATCAGTTCGAATATCT

TAGAGAGAAATGCTTTGAGCCTTCGAATACCACACGATATTTTTCCAATGATGCAAGGACGATTGCTGCC

TGTTTACAAATACGTGACAATATCTTATATCGTGCCAAGGTTTTGGGATGTGCTTCTCTATCTCAAGAAA

TGGAAAGATTGAGCATCGGAGCGCATGACTATTCTAGAGCTGAATATGCTCAACATTCTAAAAGGATTGG

TAAGAAAAAGAAGACCAAAAAAAGGTAAGCAAATGTCCACTAGTCTTGCACCAAATTAACTAATGATGGA

AGATTTCGTACTAATTTATGAGAGAGATAAAATATACTCGCAATTAAAAATAG

>MSTRG.265.1 gene=MSTRG.265

ACAAATATTGTAAGTGGCTGATCGGAAAGATATCCCGAGGAAATGTTTGCATGAAAAGTTTGGCCATTGT

CATAATTGCCCGTGGAGTGGGTACTGTTATGTCCCCCACCATCGAGTCTTTGGATTGGAAGCAACTTTCC

GTCTTCTTCAACCAGCAATCCTTCTTAACCAGGTAAAGAAGCAAATTTGCAGCCAGGAGGTATCCAGGAC

CTGCAACATTGATCCATAATGATATTGACATCAAACAGAACAAGTTCCGCTTCAATAAAAAGTATGAGAT

GTCTGAGATGCTCTTGGGTGAATTATGAACCCTTCGAATATATAAATAGTTGAGAGAAGATAGACTTGGG

TTTTAATATCTGATGAAAATATAATAATGGTGGTTTATGGCATGAACTTTTAGTACTTTTCGGATACATA

GATG

>MSTRG.266.1 gene=MSTRG.266

GTCCTGGAACGTACAGGTCATGGACTAAATATTGAGAATCGTTTTCGTGATGGGACCTATACAATAATGC

GTGATATATCAAATAGAGACCTCAATCCAAACCTGGGTTCTGACAATCTCCAACTTCGCTTAAACGTTGA

TGCACCCCCTGGTAATTGTACTTTCAGCCACACTATAAAACTAGACATCTTAAATTCATACAAATTGGAC

CCATTGTGTTGTCTGGTATTATTGATCCGTTGAGCTACTTTCTATGTAAACCCAAACGTAATGGCCGGGG

TCCTAACATTGAGAAGCAATTGAGGTTTTCCATGATAGCCAGTCCACTATGTCAAAATGAAATTAGCTGT

TCTGTCGTGAGTGAATCAGTAGTAAAAAAGCGCAAAGGCCGTGGGTTTAGCACTGAGAAACGGATACAGA

TGCGGGAGAAAGAAACACCTCATGGTCAGACACACGAGAACCACTCCTCAGCCATCAACACAGATTAAAG

GCGGCAAAGCATTCACACTCCGTCTTTAAGACCTTCATCTTCGTATTCATCTGTATTGACTGATATATCT

GGCAAAGAGGGACGAACCAATCCCACTACTTGGAATTCTCCTACCGGTATCAAGAATCTGCTTGATGTGT

TTGAGGACGAAGCTGATCACCTAGGTCAAAATCAGCAGCCTCTTGATCATGCTACTAGACCTTCTCCGTT

GTGTAATGTTGATCAGAATGTATCTGTTGGCTGTGACAATCTGTTGGCTATGAGTTGGATTCTACCATCA

AAGGTACATAGATTTTGTACTAGGTTGGATTCTATTTAGATTGTTTTTAAGGTTTGATTATACTGTGATC

TCACCTTCTATTTCTCTACCTGCCAACCCATTACCACTTTTGTATAATGCTGCGCCATGTATGTTTTTGT

CTCTTTTTACTGGATTATAACTATGTACAATTTTGTACATACTTTTGGATCAAAGTAGAGGTAGATATTA

ATTTAATACAGTTTTTCTTTCAC

>MSTRG.266.2 gene=MSTRG.266

GGCAGGTAATCTGAAATTGCATTTCTCTCGCACAATCGGCGCCGATTCAGACCATGAGGTTGTTTTTTCA

AATTTGAATGACAGATAAGTGGCAATTAATAATATGTATCAAGCTTAATCTGTATCATGCTCTTCTGAAG

CGTATCATTCATAATATTCACAGGATTGCAGGCATGGAGGATCATTCGTGAATCGAGAGTCTTCGCTTCT

AATCTCGATCGCGATCTAACAAGACAATCGCTTGACACGCTTTACTGACGGGTTGTTCTCACTCATCCTA

TCAAAGTAGTGGATTCTCACCTTCCACTTACGCCTCGAAGGGCCACGACAGTGGCTCGAAGACGTGGGTT

AGAGTGTTCCGATAATACAGATAATGGATAACGTCGTGTTCATCGCAATGCTCTCCTTCACCAGCGGGTT

GCAAATATGCAGAAACATGCCAGACCCGCTGAAGACAAAAATCAGGTAGGAGAATGGTATAATGCAGGAG

CCCAAAAGGATGCAGATCAATCAGCCTCTTCAATTGATGTTAGTGGGAGCGGCATGTTTGTATCCGGGAT

CACACAGAATGATCCCGGAACAATCACTGGACAAGTTTCAACTGACATGATAGGTTATCGACACCTTTAC

AACTATTAAATTATATTGTTTATAATTAATCAATTTGGATGTACGCTGCCCTCAAATCTTACAATTCATA

CTCATAAACCAGGAAACTCTAAAAGAAGACGTGGTCCAAATGTTGAAACTCTTCTATCGGATAATGTGTT

GAAGAAGGCGCATTCTCAAGACAAGGAAAACCATGTTCCTGTTCATGTCCTGGAACGTACAGGTCATGGA

CTAAATATTGAGAATCGTTTTCGTGATGGGACCTATACAATAATGCGTGATATATCAAATAGAGACCTCA

ATCCAAACCTGGGTTCTGACAATCTCCAACTTCGCTTAAACGTTGATGCACCCCCTGTAGTAAAAAAGCG

CAAAGGCCGTGGGTTTAGCACTGAGAAACGGATACAGATGCGGGAGAAAGAAACACCTCATGGTCAGACA

CACGAGAACCACTCCTCAGCCATCAACACAGATTAAAGGCGGCAAAGCATTCACACTCCGTCTTTAAGAC

CTTCATCTTCGTATTCATCTGTATTGACTGATATATCTGGCAAAGAGGGACGAACCAATCCCACTACTTG

GAATTCTCCTACCGGTATCAAGAATCTGCTTGATGTGTTTGAGGACGAAGCTGATCACCTAGGTCAAAAT

CAGCAGCCTCTTGATCATGCTACTAGACCTTCTCCGTTGTGTAATGTTGATCAGAATGTATCTGTTGGCT

GTGACAATCTGTTGGCTATGAGTTGGATTCTACCATCAAAGGTACATAGATTTTGTACTAGGTTGGATTC

TATTTAGATTGTTTTTAAGGTTTGATTATACTGTGATCTCACCTTCTATTTCTCTACCTGCCAACCCATT

ACCACTTTTGTATAATGC

>MSTRG.266.3 gene=MSTRG.266

GTCCTGGAACGTACAGGTCATGGACTAAATATTGAGAATCGTTTTCGTGATGGGACCTATACAATAATGC

GTGATATATCAAATAGAGACCTCAATCCAAACCTGGGTTCTGACAATCTCCAACTTCGCTTAAACGTTGA

TGCACCCCCTGTAAAAAAGCGCAAAGGCCGTGGGTTTAGCACTGAGAAACGGATACAGATGCGGGAGAAA

GAAACACCTCATGGTCAGACACACGAGAACCACTCCTCAGCCATCAACACAG

>MSTRG.272.1 gene=MSTRG.272

ATTTACATATATGGACAATAGCACCAAATCTGTTATACAGCTATGATATCTAAATCTGACTAGATATGAC

TATTATACAGCTAATACTTTTAACACCCTCCCTCAAATTGATGTTGTTGGTAAATCTACAAGCATCAATT

TGCCAACAAGAAAGTTGTGGCGCTGACGTGGCATCGATTTGGTAAAAATATCAGCGATCTGTAATGTAGT

GGAGGTATGTGGGAGAGTAATGACCAAGTGGTCATAGGCCTCGCGAATAGAGTGGCAGTCAACCTCGATG

TGTTTCGTGCGTTCATGGTAAACTGGGTTAGCTGCGATTTGAATGGCGTTGGTGTTATCACCATGTAAGG

GAGTTGGTTCAGCTTGGGAAAACCCAAGTTCTGCAAGGAGCCCACGCAACCAAATAATCTCAGAACAAGC

AGCAGACATGGATCGATATTCTGCTTCCGTGGATGACTTAGAGACGCGATTCTGCTTTTTACATTTCCAT

GAAATAAGGGAGTCTCCAAGAAACATACACCAACCAGTAATAGATCTTCTAGTGTCTGGGCAACCAGCCC

AATCGGCATCACTATATGCCTGAAGCTTGAGTGAGGAACCAGAAGGGAAAAACAGGCCGCGATTAGGAGT

TCCAAGAATATATCGAATGATCCGTTTGACTGCTGATAGGTGGAAATGCCGAGGACACTGCATGAACTTA

CTTACAATATGGACAGCATAAGAGATGTCTGGTCGAGTGATTGTAAGATATATGAGGCTGCCAACTAGCT

TCCGATATAATGTAGGATCTGAAAGTAGCTCCCCATCATTTCTGCGATATTTGACATTAATTTCCATTGG

GGTTTCAACAGAATTAGTGCCATTGAGTCTTGCCAATTCAACCAGATCCTGAATATACTTGTGTTGGTTC

AAAAAGATACCTTCTGGTCGATGATGTACCTCTAATCCTAAGAAGTAGTGTAACTTTCCAAGATCTTTCA

TTTCAAAAGTAGAACACAACAGATTTTTAGGATGATTGATGGCTTGTATATCAGAACCTGTAACAAGAAT

ATCATCCACGTAGACAAGAAGCACAACTATACCCTTACTTGTCTTTAGCAGAAAAAGGGAAGAGTCATAT

TTACTTTGTATATATGAGAAACCAAGTAGAGTGGATTTGAACTTTTCAAACCAAATTCTAGGTGCCTGCT

TCAGACCATACAAAGATCGCTTGAGTTTGCACACATGATTAGGTGAAATAGGCATGCCATTGGGAAGTGT

TATGTAAACTTCTTCTTTGAGGTCACCATGTAAGAAAGCGTTTTTTACATCCATTTGAGACAAGGTCCAT

GATTGAGACGCCGCAATGGCTAGTATGGTTCGCACAATAGTCATCTTAGCTACAGGGGCAAACGTCTCTT

CGTAGTCAATGCCGTACTCTTGTTTGTTTCCAAGAACTACTAACCGAGCCTTATATCGCTCCACAGAACC

ATCAGGATGAAGTTTTACAGAGAATACAAACTTGCTGTCCAACGGTTTTGCAGATGAGGGACGCACTACA

ACATCCCATGTTTGGTTTTTTTCAAGTGCAAGTAGCTCTGTTTCAATAGCTTCCCGCCAACATTCATGCT

TCATTGCCTGTTTATATGATGATGGAATAGAAATGGGAGACACAGTAGTTGTCAATGATACCGGATTAGA

AAAACCATACCTGTCGGGAGGCTGACTAACTCGAGTACTGTGTCTTCGAGTGGCAGGTGCATTTGCTTGA

ACAAGATCAGCAGCAGGGGAATCAGTTTGAGGGGGTTGAGCAGCTTGAACATGATGAGTAGCTTGATTTG

GATCAGGTTCTGTGGTTTCTGACATGTTTAAGGTTTTCACAGCTTTCGGGCGTCGTTGATATACCAAGAG

TGGTTTAGAGGGAGGCTCTGAATTTGACGATACTGAAGTAGATAAATGTTCTGCAGAAGAGTTAGTAAAT

AATGGTACAATTGATAATGAAGGAGCATGATGATCATTGTAGGTAGAGAAAAAAGGCTGATTTTCTAAGA

AAATTACATTTCTTGAAACCCTTATACGTCGTAAATTAGGATCATAACAGAGAAATCCTTTTTGGTGCTG

AGCATATCCTAAGAAAGCACATCTTATCGATTGAGCTGTGAGTTTTGTGCGCTCATGTGGAGGTAGGTGA

ACAAAACATACACACCCAAAAGTACGAAGACCAGAATAATCAGGAGTATAACCAAAGAACCGAGTGAAAG

GTGAAACATGATTTAAGGATGAAGAGGGTAACCGATTTATGAGGTATACTGCAGCAGAAAGAGCTTCACA

CCAGAACCTAGCGGGAACAAATGATTCTAACAAAAGGGTACGTACAACATCAAGAAGATGACGGTTTTTT

CTTTCAGCTACCCCATTTTGTTGAGGAGTTGAAGGACAAGACCGTTGAAATATTATGCCAATCTTTTGTA

AAAATTCTTGAAACAAATGAGACGTATATTCACCCCCATTATCCGAGCGAAGAATTTTAATATTTGCACG

GAATTGTGTTTGAACATATGCATGAAACACCTTGAAGGTTGAAAGGACCTCAGCTTTGGAAAGAAGGAAA

TATATCCATGTAAAACGACTGTAATCGTCAATAAATGTGACAAAGTACTTATAGTTAGCATGGGAAACAA

CCGGTGACATACCCCAAACATCACTATGTATTAAATCAAAAGCTCGAGTTGCACTAGATGTATGAATGGG

AAATGGTAAAGTCTTGCTTTTTCCAAGTTTACAAGAATTGCAATCAAACTGGACAGAAGATAGCTTTTCA

TTCCCAAGTACACCAGATTTCAACAAAACACGAAGAACATTGTTATTAGGGTGTCCTAGACGCTTATGCC

ATGCATGGTAATCAACACGGGCAGAATTACAAGAAACAATGGGAAAGGAGCAGCTTGGAGATAAAGGAAA

TTAGATAGGAAACAAACGTTCAACTTTAGGCCCCCTCGTGATCACCCTCCCCGACTGTTGATCCTGCACA

ACACAACCAGGTTTTGAAAATGTCACTTTGCAATTATTTTCAACCAATTGACCAACAGAGATAAGATTAG

TAGTAAGGGCAGGAGAGACAAAGACATCGGTTAGGGAGGAAGAAATATCACCGGTTTCTGTAATAG

>MSTRG.273.1 gene=MSTRG.273

AATATATATAATGAGGACTCTCCAGCAATGGGAGATAAACTTTTCCCCACATCTTTTACTTCTAATATTC

TTGTCATGGTATCAGAGCCACAATTCTGATTCGCCTCTTGCTCAAGATCTGTTCTTGTTCTTCGTTCTGT

TCTTGTTCATAGTTCTTGTCTATTGACAAAGCTTCTTTCCTGGTTACTCTTGATCTTTTGAGTCATGCTT

TT

>MSTRG.276.1 gene=MSTRG.276

TTCAGACAAGTCAATGTCCTTGAATTTGGTCCCTGCAATAGATTCAGCCTCCCAACCAGTGCCGAACACG

AACTCCACAGGCTCATAGCCCCTACAATCAAACTGCATCATAGGAGCATATTTACCTGATTCACTTAGTT

CTTGAGTCAGTGCACTGCCTTTGCCACTGATCATAGTCACATTTCCATCCCTCCCACAAAATTTACACTT

CTGAACAAGATGAGCGGTACCCTTCCCCATAGGCACAACTTCAT

>MSTRG.280.1 gene=MSTRG.280

CGCCAGCATACATGTATGGATTGGTTGGCATCCCGAGTACACCAGGGCCGCCATGGTCGGAGTAGTATAT

GAAAATGTGATCATTTGGACCACTATCCACAACCTTCCCACTCCCTCCAGTAGTAGCAGATTTGTCGCCA

AGAATAGCAGCAAAAACATTGTTGACGGTAACATCTTCACCAGTGTAATCCTTTGGAACACCTTTATATA

CATCAGAACCATGAGGGCTGTTAATTATTACTCCAGGCCTAGGATTCTCTTCATCATATGCAATGTCATC

ATACATGAAAACAACAATATTTTCTTCTTTGAC

>MSTRG.290.1 gene=MSTRG.290

TGCTCTGTAGAGAGCACATCATAGGTAGTATGACTGCCTCCCTGCAACTCATAGCTATAAATGCTAGTCA

CACTTGAAGGAGAAGAACTCCCAGAGCTGCACACACTATGTGAATTCTCCAATATCTCCCGAAAAACCAG

CTGTAACATCTTTACTGAATCAATTCCTGGTATCCCATGATCTGGAATCTCAAGTGAATCATTCAATAAC

ATTATGTCATCTGTTGCTACTGAACTCCAGTAGTGACCAAATTTTTCGTGCTCTTGATCATATTCCACTG

CAGTTGCAGATATATATTCTATAATCACTTCTGCAACTGACAACAGATATGACAACACAGTATCCTCCCA

ATTGGACTTCTTCATCCGCTGAACTTCCTTAAACTGCTTAACCTGATCATCCACCCGTAAAGCCAACTGA

TTCGACGTGAGACTAACAGGTGATGAGATGTCAAGCGGAAGAACTCCAACCTCACATTTGACTACCAAGC

AATCATCTTTCAAATAGCCCAATTCCTCCAAATAGCTCTTGTTCATGAAAAGAGGGAATCCCCACTGAAT

ACCGCGGCTTGGACTCACAGAAACTTGCTTCTCCCTGCCCCTGCCGAGCAACCTGCAGGCCTTATGCTTC

CCCTCCCCACTCTGATCAACAAGTGTCAACTGAAACATTGCATACATATCAGTTTCTTCACTCATTAAGC

ACACATACAGCGATAAGTATCCGCTACACTGATCATTTATGCCTCTGGGGTATAAACTGATTGCCCACTT

GTGCCTACCAGCATTAAAAGTATTTGATCTCAAGTATGTTCCAGCTCTGAGACCTCGTTGTGAGTAGTGC

TGAAATGAATACGTCTGTGATCCCATCTCTATTGTAACTCCACTTAGTACTGAAGCAGATGGTAGCTGGC

AGAGAGTGGGAAATTCTAATGCTGTTGAGCTGCTTTCTCTGGATGCTGCAGACCCTTCAGGTGTCGAAGA

TGATGATGAGGCCATTGTTTCTGTGATTTCCGCGTCTTGTGAAGGCTAAGAAAATGCAATTTTCTGCATT

GGATTATGATACAATCTTGTAAAAGATTTAGCAGGTTTAGTGAAGATCTTAGAATAAGGCTTGATATTAA

AAATTATTGAGGTTATAGAAGGGCTAGGCTCATTTTAAAGATTAGTGGTGCTATGTTATCAGACGCGGTA

GCGAGGATGATTGCCATTGACTTGCTTTTCTGAGTCTTTTTATTTAACTTCCAGGGTCTTCTTGAATCTA

GCAGTGTTGTTTGTACAGTCT

>MSTRG.294.1 gene=MSTRG.294

GCCGATTGCTACTGAAGGCACAATCTTAATGTAATTAATGCTGAGGCCAGCAAATAACTGTCTCCAACCT

TGATCACGTGCAATAGTTGTAAGCCCTTCCCATGAGCTTCTGTATCTGGCTCCAGAATGTGTTAAAGATT

GCAAATGTTCTACCTGCATCTGTCTCCTTACAACATCTAATGGGTATGTAAAGGTCTGCCCAAATAAACC

AGCAAGA

>MSTRG.295.1 gene=MSTRG.295

AAAACAACACTTTTCACACTACTTTCTATCTCTTTGCAAAATTCTCAACTCCTGCGGCGTAGATCTAAGG

AGTTTGAGAAAACCCTAATCGTACTTTTCAAGAACCCACTTCACTCTTCAACTCATCTTCAACAATCATG

GCTGGAGCTGCACCAGAAGGTTCTCAATTTGATGCACGTCAATTTGACGCAAAAATGACCGAGTTACTTG

GTGCTGAGGGAGAAGATTTCTTTACATCCTATGATGAGGTTTATGACAGTTTTGATGCTATGGGATTGCA

GGAGAATCTTTTGAGAGGCATCTATGCTTACGGTTTTGAGAAACCATCTGCAATTCAGCAGAGGGGAATT

GTTCCATTCTGCAAGGGGCTAGATGTTATTCAACAGGCACAGTCTGGTACTGGGAAAACAGCAACTTTCT

GCTCTGGAGTTCTGCAGCAGCTTGATTATAGTGTTGTTGAATGTCAAGGTCTGGTTCTTGCTCCTACTCG

TGAACTAGCACAACAGATTGAGAAGGTTATGCGAGCTCTTGGTGATTATCTTGGTGTAAAGGTTCATGCT

TGTGTTGGGGGAACCAGTGTCCGTGAAGATCAGCGCATTCTGTCAAGTGGAGTTCATGTCGTGGTTGGTA

CTCCTGGCCGTGTGTTCGACATGTTGCGAAGACAATCTCTGCGCTCAGATTACATCAAGATGTTTGTTTT

GGACGAAGCTGATGAAATGCTCTCCAGAGGATTCAAGGATCAGATTTATGATATCTTCCAGCTGTTACCT

CCCAAAGTCCAGGTTGGGGTCTTCTCTGCCACCATGCCTCCTGAGGCCCTTGAGATCACAAGGAAGTTCA

TGAATAAGCCTGTGAGGATTCTGGTAAAGAGAGATGAGCTCACTCTTGAGGGTATCAAACAATTTTATGT

TAATGTTGACAAGGAGGAATGGAAACTGGAAACACTTTGTGATCTTTATGAGACCTTGGCTATTACTCAG

AGTGTCATCTTTGTTAATACCAGGCGCAAGGTTGATTGGCTGACTGACAAAATGCGCAGCCGTGATCACA

CAGTCTCTGCCACTCACGGAGACATGGATCAAAACACCAGAGATATAATTATGCGTGAATTCCGTTCTGG

TTCTTCTCGTGTGCTCATTACCACCGATCTTCTGGCTCGTGGTATAGATGTCCAACAAGTATCCCTTGTA

ATTAACTATGATTTGCCGACTCAGCCAGAGAACTACCTTCATCGTATTGGTCGTAGTGGACGTTTTGGAA

GGAAAGGTTTTGCAATCAACTTTGTGACCAAGGATGATGACAGGATGTTGGTTGACATACAGAAGTTCTA

CAATGTAGTAGTGGAGGAGCTGCCAGCCAATGTTGCCGATCTTCTTTAGGGTGGAATTCATCTGTTTATT

TAAGTGTTACTTAAAAGTTACCTTGCTGTCCAATCTTGGTAGGTTTACTATCCGAGACACATATTTTGGG

TATCGAACTCCCTTTACAACTTCTTCCTAGTTTGTTGTAATTTTGTTTTTGTTTTTTTTGCGCAAAATTT

GGTGAATTTCTGTATCCTATATTCTGATACGATTTGCAGAATGGTATGCTTGAAGTCGAACACTTGAGCT

CAAGTGCTGAATCTTAAGTTTTGATTTTTTTCTCATCTGCAAGGATTTTGTCATTTTGCTGTCCGTCTTG

TTTCAGTTGGGTTATAAAGGTTATGTGAGATGTTTGAATATTTATTTACCTTGTGATTC

>MSTRG.295.2 gene=MSTRG.295

AAAACAACACTTTTCACACTACTTTCTATCTCTTTGCAAAATTCTCAACTCCTGCGGCGTAGATCTAAGG

AGTTTGAGAAAACCCTAATCGTACTTTTCAAGAACCCACTTCACTCTTCAACTCATCTTCAACAACTTGA

TTATAGTGTTGTTGAATGTCAAGGTCTGGTTCTTGCTCCTACTCGTGAACTAGCACAACAGATTGAGAAG

GTTATGCGAGCTCTTGGTGATTATCTTGGTGTAAAGGTTCATGCTTGTGTTGGGGGAACCAGTGTCCGTG

AAGATCAGCGCATTCTGTCAAGTGGAGTTCATGTCGTGGTTGGTACTCCTGGCCGTGTGTTCGACATGTT

GCGAAGACAATCTCTGCGCTCAGATTACATCAAGATGTTTGTTTTGGACGAAGCTGATGAAATGCTCTCC

AGAGGATTCAAGGATCAGATTTATGATATCTTCCAGCTGTTACCTCCCAAAGTCCAGGTTGGGGTCTTCT

CTGCCACCATGCCTCCTGAGGCCCTTGAGATCACAAGGAAGTTCATGAATAAGCCTGTGAGGATTCTGGT

AAAGAGAGATGAGCTCACTCTTGAGGGTATCAAACAATTTTATGTTAATGTTGACAAGGAGGAATGGAAA

CTGGAAACACTTTGTGATCTTTATGAGACCTTGGCTATTACTCAGAGTGTCATCTTTGTTAATACCAGGC

GCAAGGTTGATTGGCTGACTGACAAAATGCGCAGCCGTGATCACACAGTCTCTGCCACTCACGGAGACAT

GGATCAAAACACCAGAGATATAATTATGCGTGAATTCCGTTCTGGTTCTTCTCGTGTGCTCATTACCACC

GATCTTCTGGCTCGTGGTATAGATGTCCAACAAGTATCCCTTGTAATTAACTATGATTTGCCGACTCAGC

CAGAGAACTACCTTCATCGTATTGGTCGTAGTGGACGTTTTGGAAGGAAAGGTTTTGCAATCAACTTTGT

GACCAAGGATGATGACAGGATGTTGGTTGACATACAGAAGTTCTACAATGTAGTAGTGGAGGAGCTGCCA

GCCAATGTTGCCGATCTTCTTTAGGGTGGAATTCATCTGTTTATTTAAGTGTTACTTAAAAGTTACCTTG

CTGTCCAATCTTGGTAGGTTTACTATCCGAGACACATATTTTGGGTATCGAACTCCCTTTACAACTTCTT

CCTAGTTTGTTGTAATTTTGTTTTTGTTTTTTTTGCGCAAAATTTGGTGAATTTCTGTATCCTATATTCT

GATACGATTTGCAGAATGGTATGCTTGAAGTCGAACACTTGAGCTCAAGTGCTGAATCTTAAGTTTTGAT

TTTTTTCTCATCTGCAAGGATTTTGTCATTTTGCTGTCCGTCTTGTTTCAGTTGGGTTATAAAGGTTATG

TGAGATGTTTGAATATTTATTTACCTTGTGATTC

>MSTRG.295.3 gene=MSTRG.295

CAAGTGTCTCTAGCTTGAATAAAAGCACTCTCCCTCAGCACTGCTATTACTTCTTCGGGAATATTGATGG

TCTTTTTGTCTTCGACTCATGCTGCATCTTCGCTCATTCATTGTTACCGGTAAATCCTAGAGTTGAAGAT

ACTTCTATGAACTATAGTTTGTGCTCTAGAGGTGCAATGGATTCATTTGATCTAATGACTCTAAACCAGT

CCAACTACAAAATCTGGAGAAGTTGTATGGAGTCCTATCTTGCTAGTGAAGATTTGTGGGATATTGTGTG

TGGAGCACATGGTATTTCTTTGCAAAATACCCGAGTGAATGATGATGCTCTCAAAGATTGGAAGAAAAAG

AACGGCAGAGCTGAATTTGTCTTAAAGAGATCCATCTCTGATGAACTCTTTCATCATATTCTCTATTGCA

AGTCAGCAAGTAGTATCTGGGAAACTCTGACTAGTGTATTCAATAAAACTGACAAGGAAAATTCACTAAG

GGTGTTAGAGAATGAGCTTGAAAATGCCAGACAAGGTACTTTGTCTATTTCACAGTTTTTCTTGAAAATC

AAGAACCTGTGCTCAGAGATTTCTAAACTTACCCCAGATGAGCCGGTTTCTGACGCCCGGCTGAAGTGCA

TAGTTGATCATGGCTTGAAGCCCGAATTCGTTCCCTTTGTGAAATCAATCCAAGGATGGCCTGTACAACC

ATCTTTTGAAGAATATGAGAATATATTGGTTTCACTGGAGGGCTTAGCAAAGAAAAGTGCAGAAATTACG

TCTAAGAATGAAAGGGAAAGCGATTTGAATGCAAGAAAGCCTAAAAATAATAAAGACAAAGTCATGGCTG

GTTGTGTACCAGAAGCTTCTTGCTTCAATACAAGTATGACTGAGCTGAGCAGACTTGCTGCTGACGGAGA

AAAATTCTTTACAACATGTGATGAGGTTTATGACAGCTTTGATGCAATGGGATTGGACGAAAATCTTTTG

AGAGGCATATATGCATTTGGTTTCGAGAAGCCATCTGCGATTCAGCAGCTCGGGATTGTTCCATTCTGCA

AGGGACTAGATGTTATTCAACAGGCAGAGTCTGGTATCGGAAAATCTGTAATTTTTTGCTCTGGAGTTTT

GCAGCAACTTGATTATGATGTTGTAGAGTGTCAAGCACTGGTTCTTGCTCCTACACGTGAACTCGCACAA

CAAATTGAGATGGTTATGCAAGCTCTTGGTGGTCATCTTGGCGTAAGGGTTCTTTCCTGTGGGTGGAATA

GTGCCCGTAATGATCAACGTATTCTGTCAAGAGGTTTTCATGTTGTGGTTGGTTCTCCGAGCCGTGTATT

ACACCTGTTGCGGAAACAGTCTCTTTGTTCAGATCACATTAAAATGCTTGTTTTGGACGAAGTGGATAAA

ATTCCGTCGTTAGACTTCAAAGAGATTAATGATATCCGGCAGTTGCTACCACCTAAAATTCAAGTTGGAA

TTTTCTCGGCCACCACCATACCTCCTGAGGCTCTTGAAATCACAGGAAAATTCATGAATAAGCCTGTGAG

AATTCTGGTAAAGAGAGATGGGCTCAATCTTGAGGGTATCAAACAATTTTATGTTAATGTTGACAAGGAG

GAACGGAAACTGGAAACACTTTGTGATCATTATGAGACCTTGGTTATTTTTCAGAGTGTCATCTTTGTTA

ATACTGAGTGCAAGGTTGATTGGCTGACTGACAAAATGCGCAGCCGTGATTACACAGTCTCTGCTACTCA

TGGAGACATGGATCAAAACACTAGAGATTTTATTATGCATGAATTTCATTCTGGTTCTTCTCGTGTGCTC

ATTACCACCGATCTTCTGGCTCGTGGTATAGATGTCCAACAAGTATCCCTTGTAATTAACTATGATTTGC

CGACTCAGCCAGAGAACTACGTTCATCGAATTGGCTGTATTGGACGTTTTGGAAGGAAAGGTTTTGCAAT

CAACTTTGTGACCAAGGATGATGTCAGGATGTTGGTTGACATACAGAAGTTCTACAATGTAGTAGTGGAC

GAGCTGCCAGCCACTTTTGCCGATCTTTAGTGTGGAATTCATCTGTTTATTAAATTGTTACTTGAAAGTT

ACTCAGCTGTCGAATCTTGGCAGGTTTACTATCCGAGACATATATTGGGTATTGAACTCCCTTTACAACT

TCTTCCTAGTATGTTGTAATTTTGTTTTTGTTTTATGCGCAAAATTTGGTGAATTTCTGTATCCTATATT

CCGATACAATTTGTCGAATGGTATGCTTGAAGTTGAACACTTGAGCTCAAGTGCTGAATCGTAAGTTTTG

ATTTTTTTCTTCTCTTCTCCAAGGATTTCGTCATTTTGCTGTCCTTGTTTCACCTGGGTTATAAAGGTGA

TATGAGATGTTGAATATTTATT

>MSTRG.295.4 gene=MSTRG.295

GTTCAACTCCGAGGTTTTGTATACAAAACAACACTTTTCACACTACTTTCTATCTCTTTGCAAAATTCTC

AACTCCTGCGGCGTAGATCTAAGGAGTTTGAGAAAACCCTAATCGTACTTTTCAAGAACCCACTTCACTC

TTCAACTCATCTTCAACAATCATGGCTGGAGCTGCACCAGAAGGTTCTCAATTTGTCTGGTACTGGGAAA

ACAGCAACTTTCTGCTCTGGAGTTCTGCAGCAGCTTGATTATAGTGTTGTTGAATGTCAAGGTCTGGTTC

TTGCTCCTACTCGTGAACTAGCACAACAGATTGAGAAGGTTATGCGAGCTCTTGGTGATTATCTTGGTGT

AAAGGTTCATGCTTGTGTTGGGGGAACCAGTGTCCGTGAAGATCAGCGCATTCTGTCAAGTGGAGTTCAT

GTCGTGGTTGGTACTCCTGGCCGTGTGTTCGACATGTTGCGAAGACAATCTCTGCGCTCAGATTACATCA

AGATGTTTGTTTTGGACGAAGCTGATGAAATGCTCTCCAGAGGATTCAAGGATCAGATTTATGATATCTT

CCAGCTGTTACCTCCCAAAGTCCAGGTTGGGGTCTTCTCTGCCACCATGCCTCCTGAGGCCCTTGAGATC

ACAAGGAAGTTCATGAATAAGCCTGTGAGGATTCTGGTAAAGAGAGATGAGCTCACTCTTGAGGGTATCA

AACAATTTTATGTTAATGTTGACAAGGAGGAATGGAAACTGGAAACACTTTGTGATCTTTATGAGACCTT

GGCTATTACTCAGAGTGTCATCTTTGTTAATACCAGGCGCAAGGTTGATTGGCTGACTGACAAAATGCGC

AGCCGTGATCACACAGTCTCTGCCACTCACGGAGACATGGATCAAAACACCAGAGATATAATTATGCGTG

AATTCCGTTCTGGTTCTTCTCGTGTGCTCATTACCACCGATCTTCTGGCTCGTGGTATAGATGTCCAACA

AGTATCCCTTGTAATTAACTATGATTTGCCGACTCAGCCAGAGAACTACCTTCATCGTATTGGTCGTAGT

GGACGTTTTGGAAGGAAAGGTTTTGCAATCAACTTTGTGACCAAGGATGATGACAGGATGTTGGTTGACA

TACAGAAGTTCTACAATGTAGTAGTGGAGGAGCTGCCAGCCAATGTTGCCGATCTTCTTTAGGGTGGAAT

TCATCTGTTTATTTAAGTGTTACTTAAAAGTTACTCAGCTGTCGAATCTTGGCAGGTTTACTATCCGAGA

CATATATTGGGTATTGAACTCCCTTTACAACTTCTTCCTAGTATGTTGTAATTTTGTTTTTGTTTTATGC

GCAAAATTTGGTGAATTTCTGTATCCTATATTCCGAT

>MSTRG.295.5 gene=MSTRG.295

CTACTTTCTATCTCTTTGCAAAATTCTCAACTCCTGCGGCGTAGATCTAAGGAGTTTGAGAAAACCCTAA

TCGTACTTTTCAAGAACCCACTTCACTCTTCAACTCATCTTCAACAATCATGGCTGGAGCTGCACCAGAA

GGTTCTCAATTTGATGCACGTCAATTTGACGCAAAAATGACCGAGTTACTTGGTGCTGAGGGAGAAGATT

TCTTTACATCCTATGATGAGGTTTATGACAGTTTTGATGCTATGGGATTGCAGGAGAATCTTTTGAGAAG

ATTTCTTTACATCCTATGATGAGGTTTATGACAGTTTTGATGCTATGGGATTGCAGGAGAATCTATACAG

AAGTTCTACAATGTAGTAGTGGAGGAGCTGCCAGCCAATGTTGCCGATCTTCTTTAGGGTGGAATTCATC

TGTTTATTTAAGTGTTACTTAAAAGTTACTCGGCTGTCCAATCTTGGTAGGTTTACTATCCGAGACAGAT

ATTTGGGTATTGAACTCCC

>MSTRG.295.6 gene=MSTRG.295

AAACCGTAGCAATATTGAGCAATAAAATCAAACGGCGCCGTTCAACTCCGAGGTTTTGTATACAAAACAA

CACTTTTCACACTACTTTCTATCTCTTTGCAAAATTCTCAACTCCTGCGGCGTAGATCTAAGGAGTTTGA

GAAAACCCTAATCGTACTTTTCAAGAACCCACTTCACTCTTCAACTCATCTTCAACAATCATGGCTGGAG

CTGCACCAGAAGGTTCTCAATTTGATGCACGTCAATTTGACGCAAAAATGACCGAGTTACTTGGTGCTGA

GGGAGAAGATTTCTTTACATCCTATGATGAGGTTTATGACAGTTTTGATGCTATGGGATTGCAGGAGAAT

CTTTTGAGAGGCATCTATGCTTACGGTTTTGAGAAACCATCTGCAATTCAGCAGAGGGGAATTGTTCCAT

TCTGCAAGGGGCTAGATGTTATTCAACAGGCACAGTCTGGTACTGGGAAAACAGCAACTTTCTGCTCTGG

AGTTCTGCAGCAGCTTGATTATAGTGTTGTTGAATGTCAAGGTCTGGTTCTTGCTCCTACTCGTGAACTA

GCACAACAGATTGAGAAGGTTATGCGAGCTCTTGGTGATTATCTTGGTGTAAAGGTTCATGCTTGTGTTG

GGGGAACCAGTGTCCGTGAAGATCAGCGCATTCTGTCAAGTGGAGTTCATGTCGTGGTTGGTACTCCTGG

CCGTGTGTTCGACATGTTGCGAAGACAATCTCTGCGCTCAGATTACATCAAGATGTTTGTTTTGGACGAA

GCTGATGAAATGCTCTCCAGAGGATTCAAGGATCAGATTTATGATATCTTCCAGCTGTTACCTCCCAAAG

TCCAGGTTGGGGTCTTCTCTGCCACCATGCCTCCTGAGGCCCTTGAGATCACAAGGAAGTTCATGAATAA

GCCTGTGAGGATTCTGGTAAAGAGAGATGAGCTCACTCTTGAGGGTATCAAACAATTTTATGTTAATGTT

GACAAGGAGGAATGGAAACTGGAAACACTTTGTGATCTTTATGAGACCTTGGCTATTACTCAGAGTGTCA

TCTTTGTTAATACCAGGCGCAAGGTTGATTGGCTGACTGACAAAATGCGCAGCCGTGATCACACAGTCTC

TGCCACTCACGGAGACATGGATCAAAACACCAGAGATATAATTATGCGTGAATTCCGTTCTGGTTCTTCT

CGTGTGCTCATTACCACCGATCTTCTGGCTCGTGGTATAGATGTCCAACAAGTATCCCTTGTAATTAACT

ATGATTTGCCGACTCAGCCAGAGAACTACCTTCATCGTATTGGTCGTAGTGGACGTTTTGGAAGGAAAGG

TTTTGCAATCAACTTTGTGACCAAGGATGATGACAGGATGTTGGTTGACATACAGAAGTTCTACAATGTA

GTAGTGGAGGAGCTGCCAGCCAATGTTGCCGATCTTCTTTAGGGTGGAATTCATCTGTTTATTTAAGTGT

TACTTAAAAGTTACCTTGCTGTCCAATCTTGGTAGGTTTACTATCCGAGACACATAACTATGATTTGCCG

ACTCAGCCAGAGAACTACCTTCATCGAATTGGCTGTATTGGACGTTTTGGAAGGAAAGGTTTTGCAATCA

ACTTTGTGACCAAGGATGATGACAGGATGTTGGTTGACATACAGAAGTTCTACAATGTAGTAGTGGAGGA

GCTGCCAGCCAATGTTGCCGATCTTCTTTAGGGTGGAATTCATCTGTTTATTTAAGTGTTACTTAAAAGT

TACTCGGCTGTCCAATCTTGGTAGGTTTACTATCCGAG

>MSTRG.295.7 gene=MSTRG.295

GTTCAACTCCGAGGTTTTGTATACAAAACAACACTTTTCACACTACTTTCTATCTCTTTGCAAAATTCTC

AACTCCTGCGGCGTAGATCTAAGGAGTTTGAGAAAACCCTAATCGTACTTTTCAAGAACCCACTTCACTC

TTCAACTCATCTTCAACAATCATGGCTGGAGCTGCACCAGAAGGTTCTCAATTTGATGCACGTCAATTTG

ACGCAAAAATGACCGAGTTACTTGGTGCTGAGGGAGAAGATTTCTTTACATCCTATGATGAGGTTTATGA

CAGTTTTGATGCTATGGGATTGCAGGAGAATCTTTTGAGAGGCATCTATGCTTACGGTTTTGAGAAACCA

TCTGCAATTCAGCAGAGGGGAATTGTTCCATTCTGCAAGGGGCTAGATGTTATTCAACAGGCACAGTCTG

GTACTGGGAAAACAGCA

>MSTRG.295.8 gene=MSTRG.295

CTACTTTCTATCTCTTTGCAAAATTCTCAACTCCTGCGGCGTAGATCTAAGGAGTTTGAGAAAACCCTAA

TCGTACTTTTCAAGAACCCACTTCACTCTTCAACTCATCTTCAACAATCATGGCTGGAGCTGCACCAGAA

GGTTCTCAATTTGATGCACGTCAGCAACTTTCTGCTCTGGAGTTCTGCAGCAGCTTGATTATAGTGTTGT

TGAATGTCAAGGTCTGGTTCTTGCTCCTACTCGTGAACTAGCACAACAGATTGAGAAGGTTATGCGAGCT

CTTGGTGATTATCTTGGTGTAAAGGTTCATGCTTGTGTTGGGGGAACCAGTGTCCGTGAAGATCAGCGCA

TTCTGTCAAGTGGAGTTCATGTCGTGGTTGGTACTCCTGGCCGTGTGTTCGACATGTTGCGAAGACAATC

TCTGCGCTCAGATTACATCAAGATGTTTGTTTTGGACGAAGCTGATGAAATGCTCTCCAGAGGATTCAAG

GATCAGATTTATGATATCTTCCAGCTGTTACCTCCCAAAGTCCAGGTTGGGGTCTTCTCTGCCACCATGC

CTCCTGAGGCCCTTGAGATCACAAGGAAGTTCATGAATAAGCCTGTGAGGATTCTGGTAAAGAGAGATGA

GCTCACTCTTGAGGGTATCAAACAATTTTATGTTAATGTTGACAAGGAGGAATGGAAACTGGAAACACTT

TGTGATCTTTATGAGACCTTGGCTATTACTCAGAGTGTCATCTTTGTTAATACCAGGCGCAAGGTTGATT

GGCTGACTGACAAAATGCGCAGCCGTGATCACACAGTCTCTGCCACTCACGGAGACATGGATCAAAACAC

CAGAGATATAATTATGCGTGAATTCCGTTCTGGTTCTTCTCGTGTGCTCATTACCACCGATCTTCTGGCT

CGTGGTATAGATGTCCAACAAGTATCCCTTGTAATTAACTATGATTTGCCGACTCAGCCAGAGAACTACC

TTCATCGTATTGGTCGTAGTGGACGTTTTGGAAGGAAAGGTTTTGCAATCAACTTTGTGACCAAGGATGA

TGACAGGATGTTGGTTGACATACAGAAGTTCTACAATGTAGTAGTGGAGGAGCTGCCAGCCAATGTTGCC

GATCTTCTTTAGGGTGGAATTCATCTGTTTATTTAAGTGTTACTTAAAAGTTACCTTGCTGTCCAATCTT

GGTAGGTTTACTATCCGAGACACATATTTTGGGTATCGAACTCCCTTTACAACTTCTTCCTAGTTTGTTG

TAATTTTGTTTTTGTTTTTTTTGCGCAAAATTTGGTGAATTTCTGTATCCTATATTCTGATACGATTTGC

AGAATGGTATGCTTGAAGTCGAACACTTGAGCTCAAGTGCTGAATCTTAAGTTTTGATTTTTTTCTCATC

TGCAAGGATTTTGTCATTTTGCTGTCCGTCTTGTTTCAGTTGGGTTATAAAGGTTATGTGAGATGTTTGA

ATACTCCCTCCGCCCCCTGAGTAGTATACGGGGAACGGGGACGCGGCACGGACTTTAATACTCCTGTAAA

GTGTAGTTGTG

>MSTRG.296.1 gene=MSTRG.296

TCTATGTTACCCGGACTCTCCATTTTTGTGCCTATACCCGTGTCCACCGGACATGACATGGGTGTGGGTA

TGGGATCTGTGTCGGATCCTTCTTATTACAACCGGACACCTCACCTTGTAACATCCCATGTCAAAATGAA

AACTTAGGTCTTGTTCCAACTTGTTTATAGAGATCAAGATCCATGAATGACTTGTTTCTATGTTGTTCTA

TTGATTTTATGCTTCAATTTTCAGCATATGTGATTTATGTGCAATAGACACTATTTTTATGAATGTACCA

TAAATCAGGCTATATTAAGTTTGACTTGCATGGAGCTTAAGTGCCAAGTCCCCGTACCCGAGTCTAATTC

CGGATCCACATCCACGAATCCTAATTATCCTAAAACTACGAATCTGACTCTTGGATCTGTACCCGTGTCC

GATACCCATACCCGTGTCCGGGTAACTTAG

>MSTRG.304.1 gene=MSTRG.304

TGTTTACAGACATGGAATCGAATACTGATACTATCATGGACGTGGAAAGAGGTGCAGGGAGAAACAAAAG

GAAGTGTATTTTTTGGAGCATCGGATGCAGAGAAGAAACAACTTGTGGATGCTATACTTGCCGGTGAAAT

ATAAGCATTACTGCACTTATTTATAAACTTATGAATGTTTCGAGACTTGTCGATTTTTATTTTGTACTTA

TTTGATGATGTTCAATTTAGAATTTTGTATCATTTTGCCTTTAGTTAATTGCATTTATTTTGTCGATT

>MSTRG.304.2 gene=MSTRG.304

TGTTTACAGACATGGAATCGAATACTGATACTATCATGGACGTGGAAAGAGGTGCAGGGAGAAACAAAAG

GAAGTGGTCTGAAGAAGAAGATGAGAAATTGGTCGAGTCTCTTATTGAACTTGTGAACAATGGAGCATAC

AAAGCTGACAATGGTTTCAAGCCGGGATATCTTGGATTTCTGGAAAACTCATTGAGCGTCAAACTACCAA

CTTCAGGCCTCAAGGGGAAGCCACATATCGAGTCTCGAATAAAAACTCTCAAGAAGGATTTTAATATGGT

TTATGACTTAAGATATGGAAGCACATCTGGTTTTGGTTGGAATTCTGAAAATCAACTAGTCACGGCATCA

AGAGATGTATGGGCTGAATATGCCAAGTCTCATGTTGGGGTTTTGAAATGGAGAAGCACACCATTTCCAT

TTTTCGACGATCTTGCAATCATCTTTGGCAAAGATAGAGCAACTGGTCATAATGCTGAAAGTGCAATGGA

GGCGGAAGAAAACATTAATCTAGAAGAAGCTGCTCAAGGAAAGAATGATGATTCGAGTATGCAAGCATCT

GATGAGTCTACTTCTAGAAGGAGTTCAAAGAGGAAAAAGTTTGATGCTGAACAAATGGCTGAGGTGATGT

ATAATGCTTCTAAAATGATAGCGGCTGAGTTTGCAAATTCAACTAAAGTGATGGCAGCTGAATTTGCGAG

TTCCACCAAGCTGTTGATTGCTGCAGAAACTGATAGGTTGGAGAAAAAAGAAAAGCTAATGGACGAGTTG

TCAAAGATATCTGACATTGATGTTGTGCAAAGGTTCAAGGCTGCAAAGAAGATAGCTGATAGTGAAAATT

TGATGGTGTTATTTTTTGGAGCATCGGATGCAGAGAAGAAACAACTTGTGGATGCTATACTTGCCGGTGA

AATATAAGCATTACTGCACTTATTTATAAACTTATGAATGTTTCGAGACTTGTCGATTTTTATTTTGTAC

TTATTTGATGATGTTCAATTTAGAATTTTGTATCATTTTGCCTTTAGTTAATTGCATTTATTTTGTCGA

>MSTRG.309.1 gene=MSTRG.309

GCCAAGACTGCAGGTAAAAGGTCACACCAGAGAGGCATAACTCATTACAATGAAAGCTCCAACGAGTATA

GGCCAGAGACGAGGATGGCGAAAGAAACTATAGCTTCCACTGCAATCATCATTGGCACCACTGTAACATC

CGGGTCTGGTTGACTTAGCCACACCTCCTTCAAAAGCGAAGCTGCTGTTGCCTTTGCCATTGCTGAACAG

AACACACCAAAAATACGGACCTCCTCCATCTGCCCCGCTAACACCTGCACCCACCTCAGAGTGGTTACTG

TTGTGGACAATTTCCAAGGCCTTGTCATTTGTCACTAGGATGTCTGAGAATGCTTGCTTAGGCTGGATAT

ACTTTGACTGGCATCCGAGTAGACGTCCAGTGAAAGGGGCTAGTGATGAGACCTCAACACCGCAGTTGGG

GGCAAAGGTTTCAGCAATTTCAGCATCAGGAGGCTTCTTGGCGTTTTTCCCTCCCACTGAATCACAATCT

CCTCCGTATGCCTTTATGTACTGCAGGGCTATGCATGCCAGGCCCGGATTGTTAAAGAGTGCTGATGATC

CTTTTCGTGCAGTTCTATTACTGTTAAGCTCCGCGACTAGCTTATCAGCAGGGTTATCTGTCACTTTCTC

TGAGACTTTATATCAAAGAAAATGTTCAGATTAAACGAGCCTAT

>MSTRG.310.1 gene=MSTRG.310

GTTTGAACTTAGGTTAATTTTTTTGCTAGAGAGGAAGCAAGGAAAAGAAAAGAGGAAGACGAGAAAAAGA

GGAGGAGAATGACATCGAATTTTTATTGGATTTGAAGTTGAGGTTGCAAGGCGGGCTATGGGGAGAACGC

AACTTGTGGACTTAGAAGAGTTCGCTTTATGGATTGTATCTTTTGTAGACTTCTAGAATTTTATTCGAAT

TTAGTGATTGTGGATTAAATTTGAATTTGAATATATTATTTTTAGTTTCATGGAATGTAAGTTGGATTTG

TTCGAATTGAGTTTTTTATTGATATACTATGGAC

>MSTRG.311.1 gene=MSTRG.311

GTGAAAGCATTGCCTGATGAACTGGGGAATATTGAATCTCTAAGAGAGGTCAGAATATCGGGAATAGGAG

TTTCGAAATTGCCAGACAGCATTTGCAACCTGAGATCATTGGAAATTCTTGATATATCTTCTTCTGATAC

ACTAGTCAGGTTACCTGATCAACTGTGGAAGCTTACAAGTTTATTAGAGCTAAATGCATGTTCTGCTAGT

CGTTTAGAGAAAGTTCCTGACATAGAATCAAGCCAGACTTCATTACCACTGACAAAGTTGAATTTATCTA

ATAGTAATATCACTGCCCTGCCATCCGGTATTAGTCAGCTCTCAAACCTAGAACGTCTTCATCTTTCGAA

ATGTGATTATCTGTTGTCCATAACAGAACTTCCTCCTAATCTGAAGTACATTTCTGCCATACACTGTACA

TCTTTGGAAAGAGTAAATCTATCCAATTTGAAACTGTTGCGGAGGTTGCAACTCACAAATTGCAGTGCTT

TGACAGAGATTCTAGGCCTGGAGGAACTCACTTCTTTAGAATATCTGCATTTGCTAGATTGCCGCAGCTC

TCTACTGACACATACTTTAACAAAGCCACTGTTCCAGATGTTCTCTGGTTTTGAGAAAAAAATCAATATT

TCGGTTGGCGTGGAAGACTTTCCAGATTGGATTATTCCAGATGAAGATGGAGATGCGTCATGTTCTGTGA

ACTCTTTCGAAGAAACTTCAGATGGAGACGCGTCATTATCTGTGAATTTGCGACCAAACTTGTCACACAA

TTACTTGGGGATGATTCTCTGCTTTGACCAGCGATATTACGATGCCTGTTATTCTGTTATGACTTCCACA

AGTAATATATTAGAGAGCCGTATATATGACTCAGGTATAGTAATAGTTCCGAGATCAATCTTTAGAGTCA

CAGATAGCGATCATACAATCAAACTTACATCAAAAGGAGTAAGTGGATATTGGATTCATCTACTGTACAA

AAACGAGGACAACACATTTGCACCCAGACTTTGGATGCATCTACTGTACAAAAATGAGGACACCAGCATC

ACTCACATTGTTGCAGATGCAGGA

>MSTRG.312.1 gene=MSTRG.312

AAAATGCTGGCAAGACTTTGCACTTTTTGACTAGCAACTCGTATAACAACATTCAACATTGACAGCTGTA

TTGCAAATTTGAAGCTCTTATCTTTTGTATCTAATTCTCCTCCACACAGCATCTATCTCTTCCCCTTATC

TTATCCAATGGCTTCAACAAGTTCTCAAACCCGCACTACCTGGGACGTTTTCTTGAGCTTCCGTGGCACA

GACACTCGAATTGGTTTCACGAGTCATTTATACTCTGCCCTGGATCGCAATGGTATTCGAACTTTTATGG

ATGATCCTGAGCTACGTCTTGGAGATGGGATCACGGCTGAATTGCTCAAAGCTATTCAGGAATCGGAAAT

TTACATTGTTGTCCTCTCGGTAAATTATGCTTCTTCGAGTTGGTGCCTTGATGAGCTGGTAGAGATTCTT

GATTGTAAGGAAACAATGAAAAGATTGGTTATTCCTGTGTTTTTCAACATTGATCCTTCAGTGTAGAGCT

GTCAAACGGATAATTCGGATACGGATATGCCTATATCCATATCCGTATCCGTAATGGCCGGATTCAGATA

CGGATAATATCCGCATTATTTCGGATACGGATAATATCCGCTTTTTCTCGGATACGGATGCGAATATTTC

GGACGGATGTCGGATATTTTTGATGACTCTACCGTATTAACGATGATTTTCAAACAATTTTTTGATGACT

AAGCTCA

>MSTRG.313.1 gene=MSTRG.313

GTCCAGTGAAAGGGGCTAGTGATGAGACCTCAACACCACGGTTGGGGGCAAAGGTTTCAGCAATTTCAGC

ATCAGGAGGCTTCTTGGCGTTTTTCCCTCCCACGGAATCACAATCTCCTCCGTATGCTTTTATGTACTGC

AGGGCTATGCATGCCAGGCCCGAATTGTTAAAGAGTGCTGATGATCCTTTTCGTGCAGTTCTATCACTGT

TAAGCTCAGCTAATTTAACAATGAGAATTTTAGATCTGTCAAAAGGATGTACGTTGAGGAGCTTCTCACC

TGCAACAAGTATTGAAGCAGCAACCAGTTCAGTGCCTATTTCATGTGGGCGATTGTACCTTTGCCGACTC

ATTTCATAATCTTCAACTTCTTCTATTTTCTGTTCTCTCTGTCTTTCAGTAGAACCTCATGGTATATATT

TGTCAATGAAGAAGGATACTTAATATTTGAAGCCTCTCCGAGTTACTTCCATATTCAGCAATAAAATTAA

TAATTTCAGATACTACTGTGATATCACCTCCACAAGGAAACTACACATCGTTGGCACAGGCATTCGTCAT

CAGAGAAACTACTACTTATAAGCTCGACATTTCTTGCAACTCTAGAATGCGCATACAATACTCACCATTA

TCACTTGACAAACAGACAAGAAACTACACAACTCTTGCAACTCCGAGG

>MSTRG.314.1 gene=MSTRG.314

CAAACCCACACCCACTTGCATATATAGTGTGCTATACTAGAGAAGCACATTGATACACACGCACAGATTC

AAAATTTCAAATTCAAATCACATCCTCTTCTTTTTCTCCTTTACATTTGATTATCTTCTCCTCTCTCCCT

CTGATTTGTGGCTGGAAATCAAGCATAAGAGAATCTACTCAGAAAAGGCGTGAATGAATATAAGATTCAG

GTAGCACGAATCCAACTCGAGGACTACAAGAGGAGGATTTCTTTTTTGACAATTATTTCATTGTTCTCAG

GTGATTTTCTGTTGAGATTGCAAAGTCGTTGCTTGATAACAAATGTTGTGTGATCTTATCTGATTTACAC

AATTTGGAAGCTGTAGACTCATTAACTCCCAAGTTGAGTTCAGTCAATTGGAGCCGCCGCATGGCAATTC

CTTACAAGAAGAAAACAACGATGTTCTTTTTGATGAGGATGAAGAAATTTCTGAAGTTGATTCTAAAGAT

TCAGAGGATGAATCTGACCCAGAGACTGATGATTTGGCTATAGGTTTGACTTAAAATGCGAAGCCTATAC

AACGAAAAGGGAAGCATGCAGCAGCCAGACCAAGGTGCAGATAACGCAATGAATAAGGTTGAGACAATTG

ACTCAGGCAAATATTCAAAATCTTCCAGCT

>MSTRG.314.2 gene=MSTRG.314

CAAACCCACACCCACTTGCATATATAGTGTGCTATACTAGAGAAGCACATTGATACACACGCACAGATTC

AAAATTTCAAATTCAAATCACATCCTCTTCTTTTTCTCCTTTACATTTGATTATCTTCTCCTCTCTCCCT

CTATTTGTGGCTGGAAATCAAGCATAAGAGAATCTACTCAGAAAAGGCGTGAATGAATATAAGATTCAGG

TAGCACGAATCCAACTCGAGGACTACAAGAGGAGGATTTCTTTTTTGACAATTATTTCATTGTTCTCAGG

TGATTTTCTGTTGAGATTGCAAAGTCGTTGCTTGATAACAAATGTTGTGTGATCTTATCTGATTTACACA

ATTTGGAAGCTGTAGACTCATTAACTCCCAAGTTGAGTTCAGTCAATTGGAGCCGCCGCATGGCAATTCC

TTACAAGAAGAAAACAACGATGTTCTTTTTGATGAGGATGAAGAAATTTCTGAAGTTGATTCTAAAGATT

CAGAGGATGAATCTGACCCAGAGACTGATGATTTGGCTATAGGTTTGACTTAAAATGCGAAGCCTATACA

ACGAAAAGGGAAGCATGCAGCAGCCAGACCAAGGTGCAGATAACGCAATGAATAAGGTTGAGACAATTGA

CTCAGGCAAATATTCAAAATCTTCCAGCT

>MSTRG.315.1 gene=MSTRG.315

GTGTTAGTGGGGCAGATGGAGGAGGTCCGTATTTTTGGTGTGTTCTGTTCAGCAATGGCAAAGGCCACAG

CAGCTTCGCTTTTGAAGGAGGTGTGGTGAAGTCAACCAGACCCGAATGTTACAGTGGTGCCAATGATGAT

TGCAGTGGAAGCTATAGTTTCTTTCGACATCCTCGTCTCTGGCCTATACTCGTTGGAGCTTTCATTGTAA

TGAGTTATGCCTCTCTGGTGTGACCTTTT

>MSTRG.316.3 gene=MSTRG.316

AAAATTGCAGTACTTTGACAGAGATTTTAGGCCTGGAGGAACTCACTTCTTTAGATGAACTATACTTGCG

AGGTTGCCGCTACTCTCTACTTACACATACTTTAACAAAGCCATTGTTCCAGATGTACTCTGCTCTTGAG

CAAAAAATCAATATTCAGCTTGACGTGGAAGGCTTTCCAGATTGGATTATTCCAGATGCGTCATCTTCTG

TGAACTCTTCCAAAGAAACT

>MSTRG.317.1 gene=MSTRG.317

CTAATATCTTTGCTATCCACCAGTTACAATATCTTCAAAACTTACATACAATGGCTTCAACAAGTGCTGA

AACTCCCACTAGCTGGGACGTTTTCTTGAGCTTCCGTGGCATAGACACTCGACTTGGTTTCGTCAGTCAT

TTATATTCTGCCCTGGATCGCAATGGTATTCGAACTTTTATGGATGATCCTGAGCTACGTCTTGGAGATG

GGATCTCGGCTGAATTGCTCAAAGCTATTCGGGAATCCAAAATTTACATTGTTGTCCTCTCTGTAAATTA

TGCTTCTTCGAGTTGGTGCCTTGATGAGCTGGTAGAGATTCTTGATTGTAAGGAAACAATGAAAAGATTG

GTTATTCCTGTGTTTTTCAACATTGATCCTTCAGTGGTGCGATATCAAAAAGGGAGCTTTGAAGAACATT

TTAGAGTGCATGGAGTTAGATATGCGGATAAAATGGAAAGAATGGAGAATTGGCGCCGTGCACTTAGTCA

ACTTGCTGAGAATTCAGG

>MSTRG.318.1 gene=MSTRG.318

AATCTTTTCATTGTTTCCTTACAATCAAGAATCTCTACCAGCTCATCAAGGCACCAACTCGAAGAAGCAT

AATTTACAGAGAGGACAACAATGTAAATTTTGGATTCCCGAATAGCTTTGAGCAATTCAGCCGAGATCCC

ATCTCCAAGACGTAGCTCAGGATCATCCATAAAAGTTCGAATACCATTGCGATCCAGGGCAGAATATAAA

TGACTGACGAAACCAAGTCGAGTGTCTATGCCACGGAAGCTCAAGAAAACGTCCCAGCTAGTGGGAGTTT

CAGCACTTGTTGAAGCCATT

>MSTRG.316.4 gene=MSTRG.316

TTTGAACATTCTACTAGCCACTTGTATTGCAAAACTGAAGTTCCTATCTTTTGTTTCTAATTCTCCTTCT

CCCCAGAACATCTTTTGTAAAAAAGGCTCTGCTCCCATATACAATATTTTCAAAACTTACATAAATCTCT

TCCCAATGGCTTCAACAAGTGCTGAAACTCCCACTAGCTGGGACGTTTTCTTGAGCTTCCGTGGCACAGA

CACTCGAATTGGTTTCGCCAGTCATTTATACTCTGCCCTGCATCGCAATGGTATTCGAACTTTTATGGAC

GATCCTGAGCTACGTCTTGGAGATGGGATCTCGGCTGAATTGCTCAAAGCTATTCGGGAATCCGAAATTT

ACATTATTCTCCTCTCGGTAAATTATGCTTCTTCGAATTGGTGTCTTGATGAGCTGGTAGAGATCATTAA

TTGTAAGGAAACAATGAATAGATTGGTTATTCCTGTGTTTTTCAACATTGATCCTTCAGCCGTGCGATAT

CAAAAAGGGAGCTTTGAACAACATTTCATAAGGCATGAAATTAGATATGCTGATAAAATAGAAAGAGTCA

ATAATTGGCGCCGTGCACTAGGACAAGTTGCTGAGAATTCGGGAACACATGTAGACGGAAAAAAGTCTGA

AGCTGATATTGTCAACGAAATTGTCAAGGAGATTCTGCTTCAAATAAAACCCACTTCTTTAGATGTTGCC

AGATATCCAGTTGGATTGGATCCCCGAGTTAAAGACATAACAGCAGCATTGTTGAGCAGTGGCACAAAAG

GTGTCATTAAGATCGGTATATATGGTATGGGTGGCGTAGGCAAAACAACTCTTGCCAAGGCACTTTTTAA

TAAACTCTTGCTAGGAAGCTTTGAAGGAAGCTGTTTCCTCGAAAATGTGAGGGAAATTTTGGGGACTATA

AAAGGCCTCGAGTCATTGCAACAACAACTTATAAGTGATGTTCTTAAAATTAGTAAGGACGAAGTTAAAA

TTAGCAGTGTTGGTCAAGGAACTGAGCAGATAGAACGAAGAATTTGTTCTAGAAAAATTTTGGTTGTTAT

TGATGACTTAGAGCACAAGGAAAAATTTGAGTCACTGGTGAGACCATTTGCTCCTGGGAGTGTAGTTATC

ATAACTACAAGGAATGAAGAGATACTTCGGGGAATTGAAGTAGAAACTCAGTATCGATACAAGGTTAATG

AGATGGGTGACGCTGAGGCAGAGATGCTATTTTTCAGACATGCATTTCGAGATACTAAACCAAACGATAA

TCTAATGATATTGTCTAAAGACATTCTACGTCTTGCTGGAGGACTCCCCTTGGCTCTCGAGGTTTTTGGC

TCGTACTTGCATAACACACCCGAGGTTAAATGGAAACCTTACATTGAGAAACTGCAGCGAGATCCCGACA

ACAGTATCGAGCAAAGACTTATAATTAGCTTGGATGCCTCTGGATCAGATGATCCCCTGCTAAAGAAGAT

GTTCCTTGACATTGCTTGTCTTTTCATTGGAAGGACGAAAGAATTTTTGATTAAAATATTGGATACTTAC

TATCCCCACGCGGATGATAAAATTGATATTCTCGAGAAAAGGAGTCTGTTAACATTTAATGACAGAGACG

AAGTACGAATGCATGATCTGCTTCATGATATGGGAAAGAAAATTGCGCGTAACAACTGTCCTGATGAGCC

TGGAAAGCATAGCAGATTATGGGTATCAAAAGAAATATGCGATGTGTTGGAGAAAGACAAGGGAACAGAA

GCGATTGAAGGTTTCTCGTTCGAACGCTTTATAATTAATGGAGGATCGTTTTCTATGGATAGATTGAGAA

GAATGACTAAATTGAGATTTCTTTACTTGGATGGCATTATTCTCACTGGAAAGTTTAAACTGACACTACA

AGTTTTGAGGTGGTTTTGTTGGAATGAGTGTCCTTTAAAGTGTTTACCTTTTGATTTTTGCCCTGAAAAA

CTTGTTATTCTTGAGTTGCCGAAGAGCAAACTGACAACAATGTGGGAGGTAAAGATGGTTTCAGATGTTT

TGGAGAAGCTAAAGACTATAAACATGTTTTTTTCCCAAGATTTAACTTACAGCCCAGATTTCCGAAGACC

ACCGCGTCTCTTTAGTTTAAAAACTCTAAACATGTCATTTTCTGAAGATTTGACTACCACCCCAGACTTC

AAAAGACTGCCGAATCTTGAAAATCTGAATCTTGAGGGCTGTAGAAGTTTGAAGGAGGTCCATGAATCAG

TTGGAAGTTTGGAGAGGCTTGTTTCCCTAAATTTGAAGGATTGTGCAAACCTTAGAAGTCTTCCAGATAC

TATCTGCAAGTTGGAAGCTTTGGAAGTTCTGTGTATTGATCACTGTATAAGGCTAAAAGCACTGCCAATA

GAATTAGGGAACATTAAATCCCTAAAAGAGCTCATTGCTTCGGGAACAAGTTTTCCGAAATTACCAGATT

CGATCGGAGATCTTAGTAAGCTTGTTAAGCTGAAATGGACTCGATGTTCCTTATACGACATGGAACTTGA

ATCTCTTCCAAACACCATTTGCAACTTAAGACAATTGGAAGTTTTAATTGTTACTGTGAAAGCATTGCCT

GATGAACTGGGGAACATTGAATCTCTAAGAGAGCTCAATACATCGGGAATAGCCGTTTCAAAATTGCCCG

ACACCATTTGCAACCTGAGATTATTGGAAATTCTTCATATTACAGTTTCTGAGACACTAGAGAGATTACC

TGATCAACTGTGGAAGCTTACAAGGTTGTTGGAGCTAAAAGTAAGTTGTACTAGTCATTTAAAGAAACTT

CCTGACATAGAATCAAGCCAGACTTCATTACCATTGACAGCGTTGAATTTATCTTATAGTAAAATCACTG

CCCTACCATCCGGTATTAGTCATCTCTCAAACCTAGAAAATCTACAACTTACGAGTTGTCATCATCTGTT

GTCCATAGCAGAACTTCCTCCCAATCTGAAGTACATACACGCCGATCACTGTACATCTCTGAAAAGACTG

AATCTATCCAATTTGAAACTGTTGCGGCAGTTGCACCTTAAAAATTGCAGTACTTTGACAGAGATTTTAG

GCCTGGAGGAACTCACTTCTTTAGATGAACTATACTTGCGAGGTTGCCGCTACTCTCTACTTACACATAC

TTTAACAAAGCCATTGTTCCAGATATACTCTGGTTTTGAGAAAAAAAGTCGATATTTGGCTTGAGACAGA

AAAGTTTCCAGATTGGATTATTCAATCAAGTATAGAATGCTCAGCTGAAGATGACATTTCAGATGGAGAT

GATTCTTCATCATCTTCGGTGAATTTGAAACCAAACTTGTCACACAATTACTTGGGGATGATTCTCTGCC

TTGACCTGAGTCAGAATAAGTTTGATCAACCTTTTACTGCCTCTTATTCTGTTAAGACTTCCGCCAGTAA

TATATTAGAGAGCCGTATATATGAATCAGGTATAGTAATAGTTCCGAGATCAATCTTTACAGGCACGGAT

ACCGATCATACAATCAAATTTGCATCAAGAAGAGCACGCAGACATATTGGACTACTGTACAAAAACGAGG

ACAACATCATCATTCCCAATGTTGCAGGAAGAAACTCCTCTAGAGACAAGAAAAAGAGGAAAAGGATAAA

GAGAAGGAGGAGATGGATTTAGACATGAGAGGTGAAAGGTCACACCAGAGAGGCATAACTCATTACAATG

AAAGCTCCAACGAGTATAGGCCAGAGCCGACGGTGGCAAAAGAAACTATAGCTTCCGCTGCAATCATAAT

CATTGGCACCACTGTAACATCCGGGTCTGGTTGACTTGGCCACACCTCCTTCAAAAGCGAAGCTGCTGTT

GCCTTTGCCATTGCTGAACAGAAAACACCAAAAATACGGACCTCCTCCATCTGCCCCGCTAACACCTGCA

CCCACCTCCGAGTGGTTACTGTTGTGGACAATTTCCAAGGCCTTGTCATTTGTGACTAGGATGTCTGAGA

ATGCTTGCTTAGGCTGGATATACTTTGACTGGCATCCGAGTAGACGTCGAGCGAAAGGGGCTAGTGATGA

GACCTCAACACCACAGTTGGGGGCAAAGGTTTCGTCACAGTATAATACTCTTTCCGCCCCATTCTTATAC

GTTACTTTTTGGTTCATTTTTTAAGACTTTTTAAACTATAGCTCGTAATATATTTTTAAATTATTTTTTC

GGAATAAAA

>MSTRG.320.1 gene=MSTRG.320

AGCAGCTGTGTGCCATATATTTGATTCAAGAACAAACTGGAACAGGACCTGCTAATAAGAGTTAATGAGA

CCAAGGCTTGAGGAAGTAAATTGATGGATATTAGAGAAGTCATAATGCGAAGTAGATTGTTTGTGGAAAA

CCAGACATAGCAAACAACAGAGGCTGGACAGAATTTCAGATGATGGCATCGGAGTATAAGGC

>MSTRG.325.1 gene=MSTRG.325

TTTTTCTCTCTTCTCTCTAGCCGCCTCCTCTTTTCATCTCCGACGGGGCTTCCATCGGTTTCCGGTGGAA

AGCCCCAAATCTTTTCGTTTGATTGCTAGTTTTTGATTGTTCTTGCTTTTTCATTGAGTTTCTTAATAAA

TCTCCATCTTCGGGGAAAGTTTTCTTAGATCTATATCACCGAAACTTTCGATTTTCGTTCTTATTCGCTT

CCAGAGGCCTTTTTGGATTTGTGAGTGGATCGATTATCTCCTAAGCGTCGTGGTGCAGATCTAATTGTTT

TAGTTTGTTTTGATTATAATTTGATTTCTCTCCCTGGTGAGAGTGTGTGATTTTTCTCTCCTTCTTTTGT

GAGAGTGGTTTGGATTCATAGATAAAGCCTTTCGGGAATTGCATTTGTGGTCGATAGCTCAAACGGAGTT

TTTGGAAAAGATGGTGAACACAGAGCAAGGATCTATACATGTACCATCGTCAATTTCAACATCGCTTATT

TGTCTCATCTTGGGTATGAAGACAGGCATGTTAATTTTTTCTATGTTTGTTCGACTCGATCATTCTTGTA

GATGCCGTATGGCCATTATTTATTGAATTA

>MSTRG.326.1 gene=MSTRG.326

CTGCAACAGAATAAAGGGATAGAGATGCACACTACACACACAGACGTCCTGCCCCTCACTTTCTAAATAT

AAAGCGCAAACTGATAAGCATTGCGAACGCACTCGCGCTCCTAGAAATTTCTCAAGCCTCGGTTACATTA

CAAACTGCCAAGAGCCTCGAGCGGCTCTGAGATGTATATGATCTCATATCTCTTCCTTATTTGTACTGAG

TTTGTCGAATCTGAAGGCTCCGACTGGGAGAAAGAGAGATAAATTGCGTAGAGTTTGTTCATTGTTTCTC

ATTCAATTCTCGGTTTCTGAATGACGGAGAAGGAGGAGAGTGCGGTTAGGAAGACCGGAGGTGAATCGCC

GGAGGTGAAGACGCCGGCGGCGAGCAAATCGGCGGTGGCGGCGGCGACTCCGACGGAGGTGGAGGTGGTG

AAGTGCGAGTGCTGCGAGTTTACGGAGGAGTGTACGATCGCGTATATCGCGCGTGTGCGGGAGAGGTACG

AAGGGCGGTGGATCTGCGGGCTGTGTGCGGAGGCGGTGAAGGATGAGATCGGGAGATCTGGCCGGAGGAT

CAGCGAGGAGGAGGCGCTGGAGCGGCATGCGAGTTTCTGTGAGGAGTTCAGGTCGCCGCCGGAGAAGCCG

GCGGAGGAGCTCATCTCCGCCGTGAAGCAGCTGCTGATCAGGAGCTTGGAGTCGCCGACCAGTGTCAGGT

CTAATCCGAGTAGTCCGAGGGGCAAGGACGGGTCGGATCTTGCCCGAGCTACGTTCGGGAGGTCCCAGAG

TTGTTTCCCCAGCATGGATGCCTGAAACATCACCGAACTAAACCGAACGTGCTCAGTATATCTCGTTCCC

GATCCCGAAGAAAGATGCTTCATCGAAGGCTGATGCGCCAATTGCAATGAATAAACGATTATTGTAAAAT

AAATAAATTAGTTTAGGAAAATAAAACGAAATTGAATTAGAGGAAGTGAAGGTTCTTGGCCAATGCAATA

GTTAGGTTCTGGATCTTACTAATTCAGATTGTTGAGTTGTATTTCGAATAAATTAGCCATGGAAATGAAT

AAATTTGATAAATGTAGTGTTTATGTACTTATCAAAAACATGTGGTGAAGAAGATGATGGGTCCGGAGCT

AGTTCATGTTTGTACAATTCTGTTGTGTAATTGCCTTCAGAAATCAATCTTTCACCTCACCAAGTTCAAG

AACCA

>MSTRG.328.1 gene=MSTRG.328

TTGTGATGATAGCAGAAAAGCGTTAGAAATCTTTGCCAAAATTTACAGAAGTGGGAAGAGGTTAGATGAG

ATCACATTCATTACTGTGGCTAATGCCTGCAGCCGCTTGACTGGGTTAGAACAAGGCAGACAAATTCATG

CATGTGTGGTAAAGTTCGGGATCGATTTAGATCTGACGGTCATATCAGGAATCCTTGACATGTATGTTAA

ATGTGGAGCCATGGCAGATGCACACAATGTTTTCCAAGAGATTCCTATACCTGATCAAGTTGCATGGACA

TCAATGATCTCGGGATGCCTGGAAAATGGTGACGAGGATCGTGCCCTTCTAATTTACTATCAGATGAGGA

AATCTGGTATAGCACCAGATGAATACACCTTTGCTACTCTTATCAAAGCCTGCTCTTGCTTAACTGCATT

AGAACAAGGGCGACAAATCCATGCCAACGTGATCAAGTCGTTTTATGTTTCAGATACTTTTGTCTGGACT

TCACTGATTGACATGTATGCTAAATGTGGTAATATAGAAGACTCCTATAATTTATTTAAAAGAGTAACTG

TGAGGGACATTGGCCCGTGGAATGCGATGATAGTTGGTTTAGCTCAGCATGGACATGGAAAAGAGGCTCT

TAAACTCTTTAACAGTTTGAAATTTGTTGGCATAAGACCTGACAGTGTCACCCTTCTTGGAGTTCTTTCC

GCCTGTGGTCATTCTGGCCTGACTTCTGAAGCTAATTCCTATTTTCATTCAATGTATAATGATTATGGTA

TTCAACCTGAGATTGAGCACTATTCCTGCCTCGTGGATGCCCTTGGCCGTGCAGGGCGTGTTAAAGAGGC

AGAAGAACTGATTGAATCAATGCCATACAAAGCTTCTTCTTCAATGTATACAGCTCTGCTTGCCGCTTGT

AAGAGTCAAGGCAATATAGAAACCGGAAAACGGGTAGCTGACCAGCTTTTAGCTTTGGAGCCACCTGATT

CGTCATCTACTTATGTTCTTCTATCAAACATATATGCGACTGCCAACCAATGGGATAAAGTAACTGATGC

TCGAAGAATGATGAAGGGCAAGAATGTTAAGAAGGATCCTGGGTTTAGTTGGATAGATATTAAAAATAAA

GTACATCTGTTTGTGGTGGATGATAAATCACATCCTCATGCTGATGTGATATATGACAAAGTAGAGGAAA

TGATTAGGATGATAAAAAAAGATGGATATGTGCCTGATAAGGATTATGTTTTGCTTGATGTGGAGGATGA

AGAAAAGGAACGTGCTCTCTATTACCATAGTGAGAAGCTAGCTATAGCTTTCGGGCTGCTAAGCACTCCT

CCATGTACAACAATCCGAGTAATCAAGAACCTTAGAGTTTGCGGGGATTGTCATAATGCCATTAAATATA

TATCCAAGGTGTCAAAACGTGAAATCATTCTGAGAGATGCAAATCGATTTCATCGCTTTAGCAACGGGAA

TTGTTCTTGTGGAGATTTCTGGTAACATCAAATCAATTACGAGTTGCCTAAAATTTTGTCAGAATATTGA

ATGTATAATACAGTTGCGAACAAGGTGGTATATAAGCTATTTATTGAACTCGGTCGGATTATAGCCCAGG

TTCTGTAATATATGTTATGATGTGCAAACAAAGCTCATGGTGAAGAACCGAAGACATCGCAGAAAGTAAC

CCCTTTATCTCTCTTGTACTTGTAGAATTTTGTACACACTCTTGTGTATGCCAATGTGACAATGTCTATG

CATATACAATTGTTTCATTAAATAAATCTGTGAGTCAATAGTTTTCCTATTGATGCACTAATTAGGCACA

ATCTGGGGTACTTCAAATTCAAAAGCAGCAGCAAAACACATTCTTTCTGACAAGCTGGCATTGGGAAGAT

GGTGATTATCGTCGTGGCGAAGGGCATTGTTGCATCTGTCAAACAGTATTGTGATGAAGAGATTCATTTA

TCACGCATCACCTACAGGGTGGTTTGCAGCAACGTGCTTCCTGCGTATGGTTTCTACTATTAGCACAGCT

GCTGTAAAGAAGCTTGCTATGTATCAGACCAGTTCGACTTCTGAGTGTATATAGGTTTTGAAAGCTTCAA

GCTGAAGAATATAATGGAGCCTGAAGGATATATAGGTAAACCATCAAATCCAAGGATGTAGGGAACTTTA

GATTACGACTATGAGTTGAATCTCATCGACTAGTTAGATTTATGTCTACCTTGACATGCAGCATACTTGG

AGGAACATAGCCTTCATCTCTAAAGATTAGGAGATACCATTATTTATTTTGGTATAGTTGTACTTGATCG

TTTGTTTAA

>MSTRG.336.1 gene=MSTRG.336

CTTCAATTCGCGTTGGATCCGACCGAAAGCGGGCGCTCCACATTATTTTTTGAAATCCTCGGTCGGTTAT

TTTTTCCCGTCATTTCAGGAGCTGTTAACTCCACCACTTTCCATCCATGATTTCAGCAGCCAGATACTCC

TGCAATTTGAAAACTCAACGTGCCTGTGACGACTCTTTCTACCGACGCAGAGTTTCAGATTCCGGTACAC

ACTCATCTCTCAGACAATGGAATCAGGTAAGTGTCGATCTTCGCTAGGTGTACTCGAGATTAAG

>MSTRG.338.1 gene=MSTRG.338

TTTTAGTCTGTGATGCTCGGACTCTCCAGAAAACCCAGCCGTACCCGTGTCCACAGGACACGACACTGAC

ACTGACACTGGGATCCGGATCCGAACATCATCCAGAAATCAGACTCTCCATCTTAGACAACCTATCTTTA

ATTTCAGTGACCTATCTCTAAGAGGAAGATAGGAATAAGAAAATATATGAAGAAACAGAGGTGAAATGGA

TGTAAAAGGAGAAACGAAGCAACTTTTGCCTTAATTTGTGCAATTGTTTGTTATGCAGTAT

>MSTRG.347.1 gene=MSTRG.347

GGGGAATGTGATGATGAGTACAAAAGTTTGTGGAAGAGAATCTGGGCGTTGAAATTACCTCGAAAGGTGA

CACACGTCATATGGAGGTTGTGTAAAGATTGCCTTCCTACTAATACAGCACTGTACAATAGATATGTTAA

TGTAAATCCACTATGTCTGTGGTGTCATGCTGCAGCTGAAACGAATTTGCATGCTATTTTCCTCTGTGAT

TTTGCTCTGACGATGTGGCGAACGGTGGGTTTACAATCGCTGGTGCATTGTACAGACCAGGAAGCGCCTA

AGGAGATTTTTGATCGTGTG

>MSTRG.348.1 gene=MSTRG.348

AATCATACTTGTGTGCATGCATTAGCTAAACACTGATACTAAAACGGCACTTTAACTGATCAAGAAGGCC

AGTAACAGTCGTAGCAAATCCATGCAACTCTCTACCACCATTTTCATACGGACACGCCGGAATCCACTGC

AACCTCTTCATCCCCATGTCGAACAACGCCGCCTTCCCGGACCCTCTAATCACCATCGCAACATACTGAC

CATTTCCAACGCAGTTAAACCCTCTGCCACTCTCCTCAGCCTCGAACTGTGCATACAGTTGTAGTGGAAT

TCTGTAAAGCCCAGAGCCTCAGGCTTTTCGGCACGTTGAGCTTGCTTTTCTCCAC

>MSTRG.350.6 gene=MSTRG.350

CAGAAGCTTGATTCTAGGGTATTTTGAGGAGTATAGATTTATGATTTAGCTGTTTGACTGACGATTCTCT

CTGTTGTGTGCCCCTTTTTTCTAGGATTTCAAGCGCTGCTTCGCCGCTGGGAAATCAATTATCCTATTAA

ATCTATCTTTCTCGCCGGAGCTGGTTGTGTGATTGTGTATGTTTATACTAGCAAATATTGAAGCAAGGCT

GGGTGACCTGGGTCCATGTGATCCTAGAACACCATAATTGTGCAAACCTGCCTCTTCGTACTAGCACACA

CTCTCCAACATGAACTGCTGAAGATGTTCCAAAATACACAGAACGAACTGGAAAAAGACATAAGAGCATG

GAAGAGTCATAGCCAGTGTTATGAAAAGCGCAAATCGGCTCTAGGCGGTGAAAGGACCTTCTAGCGCTTA

AGCGGTAAAGCGGACGCTTAAGC

>MSTRG.351.1 gene=MSTRG.351

GATGAGTACAAGTAATGCTACATAATCTCCTCAAGCTGATCCACTTACCTTTTTTCACTGTGGTTTCTAA

AACACACTATCCTTTCCTCTAATAGCTGCTCATCACCAGGGAGAGATGAAGATACAGTGTAACGTGTGTG

AGGCGGCGGAGGCGACGGTGCTGTGCTGCGCCGACGAGGCGGCGTTGTGTTATGGTTGCGATGAGACCGT

CCATGCAGCTAACAAGCTGGCTAGTAAACACCAGAGAGTTTCTCTCACTAACTCCTCATCTCCTATGCCC

AAATGCGATATTTGTCAGGAAACAGTTGGATATTTCTTTTGTCTGGAAGATCGTGCTTTGCTATGCAGGA

AATGTGACGTCTCCATACATTCGATCAATGCCCATGTTTCAGCTCATCAACGCTTTCTGCTAACTGGAGT

GAAGGTGGGACTTGAACCTACTGATGAACGTAGTGCGCCATCTTCTTCAGGAAAGTCTCAGTCCCCTGAC

AAAGTTACTGAAACAGAGTCAAGGCCATTGTCTAAAAAGAATAACAACGTGGCTTTGGAAGGTCAAAACA

ACAGTGGACATTTCCAAGTTGGCGAAGTTGGTGAGGTGGCACCGACCAATTCGCCTTTTGGTGGAGGGTC

TGATTGTGAAAGCTTTCAACAATGGCAATTAGATGAAATATTTGGAGTAAGCGATCTCAATCAGAATTAC

AACTATATGGACAATGGGTCATCCAAGGTTGTGTACCTGAACTATCACTTTCTGGCACTTTGTGTACCTG

AACTATCACTTTCTGGCACATTGTGTACCTAGAGTTTGAAGATTCTATCGAAAAGCATACTCCCGTTAGT

TTTATGTTAACACCGTTAGTTGAAGTTGTAATAAGACTACAAAGTGAAGGGTAGTTGAGTACTTTCCAGC

GGTGTACTACCATTACTTCTAGCAATTAAACAGAGGAAGCCAGGTCTGTGCACTGCCTCAGCAACAAATT

GTGAGGTCCTGTGTAAAAATTTAATAATAGGATCCGGAATATTTATATAAACATATTACTATTGCTCAAT

TGTTACATTAATATAA

>MSTRG.351.2 gene=MSTRG.351

GTACAAGTAATGCTACATAATCTCCTCAAGCTGATCCACTTACCTTTTTTCACTGTGGTTTCTAAAACAC

ACTATCCTTTCCTCTAATAGCTGCTCATCACCAGGGAGAGATGAAGATACAGTGTAACGTGTGTGAGGCG

GCGGAGGCGACGGTGCTGTGCTGCGCCGACGAGGCGGCGTTGTGTTATGGTTGCGATGAGACCGTCCATG

CAGCTAACAAGCTGGCTAGTAAACACCAGAGAGTTTCTCTCACTAACTCCTCATCTCCTATGCCCAAATG

CGATATTTGTCAGGAAACAGTTGGATATTTCTTTTGTCTGGAAGATCGTGCTTTGCTATGCAGGAAATGT

GACGTCTCCATACATTCGATCAATGCCCATGTTTCAGCTCATCAACGCTTTCTGCTAACTGGAGTGAAGA

GTCAAGGCCATTGTCTAAAAAGAATAACAACGTGGCTTTGGAAGGTCAAAACAACAGTGGACATTTCCAA

GTTGGCGAAGTTGGTGAGGTGGCACCGACCAATTCGCCTTTTGGTGGAGGGTCTGATTGTGAAAGCTTTC

AACAATGGCAATTAGATGAAATATTTGGAGTAAGCGATCTCAATCAGAATTACAACTATATGGACAATGG

GTCATCCAAGGTTGTGTACCTGAACTATCACTTTCTGGCACTTTGTGTACCTGAACTATCACTTTCTGGC

ACATTGTGTACCTAGAGTTTGAAGATTCTATCGAAAAGCATACTCCCGTTAGTTTTATGTTAACACCGTT

AGTTGAAGTTGTAATAAGACTACAAAGTGAAGGGTAGTTGAGTACTTTCCAGCGGTGTACTACCATTACT

TCTAGCAATTAAACAGAGGAAGCCAGGTCTGTGCACTGCCTCAGCAACAAATTGTGAGGTCCTGTGTAAA

AATTTAATAATAGGATCCGGAATATTTATATAAACATATTACTATTGCTCAA

>MSTRG.356.1 gene=MSTRG.356

AAAAGAACAACTGGAGAGCTACTAAATTTAACAGAAGGAGGTAAGAAGCTGAGCATAGAAAGAAAGCTCG

GGTGCATCTTTCTTCATTACCGAGTGTGGCTATTTATAGCCAATGTGAATTTGCAAGAAGCCAAGAACAT

TAAATGCATACACAAATTCTACTCCTATAAATCATAGCCCTACTATTTGATTCATTGACCACATGCATGG

CAATCACTATATTATAACACTCCCCTTTGATTGTCATGATAGTGTGGATCATCAATTGCCTCGTTAAAAC

CTTGTCAAAGAAAAACCCAGTGGGATAAAAACTTTAACGAAGGAAAAAGAGTACAATCTCCCCTTGGAAG

AAAACATCAATCACTGGAGTTTTGTTGCAGCATATTCTTGAGTCGACG

>MSTRG.371.1 gene=MSTRG.371

GTATTAAGGGGCACCCTCTAATTATAAGGTGCTTTTATAAATACAGCTTTTGCAAGGAGCATCTGCAAAG

ACACTCTACAGGAGAACAAGGACGGGTACAGGTAACACTAAGAAGTTGTTTTGCACTCGGAATCAAGATG

GCTCAACTGAACGGAGATGGCATACAGTTATGGGCTAAAGGCATAATGCACCAAAATGAGTTTCGTTCAG

CGGTTCTGTATGCAAGAAATCTGTCAATAGTTTGGGGTTCAGATGGTCGTTACTGGAAGTGGGAACGGAT

CAGCAACCCGATAAGCAGCGAGGATCTTGAGGTTGCTAAACTAATTGAAGTATGCTGGCTACAGATCGAT

GGAAAATGTAGGGACCAGAACCTGGCTAAGGGAGTGACATATGGAGTGTATTTCATTGTCAAGCTTGAAG

AGAATCATTCCATCACCCGTCCTGTGACTCTTAAGCTTACTTGTCCCAACGGTGACACCCAAGAGAACAA

GGTGGATTTGAAGCAGCAGCCGAAGAAGGAACTGATAGGGCTTAAGGTCGGTGAATTTACTGGCTCAGGA

ACCAACAAATTTATCCGATTTTCTTTTAATGGACGGGAGGGTACCAGTTGGAAGACCGGCCTTACTTGCT

TCGGTGCTGTAATTGTGCCAATATGATTTCTATATAACTTGAACTGCCAACACCCTCCTGTTATGCCTGT

GATATAAGAAATGTATCAGATGTACTAAATGTATTAAATAATAAATGACCTGATATGTTAACGTTCGAG

>MSTRG.372.1 gene=MSTRG.372

GCGAGGATCTTGAGATTGCTGAACTAATTGAAGTATGCTGGCTGCAGATCGATGGAAAATACAGGGACCA

GAACCTGGCTAAGGGAGTGACATATGGAGTTTATTTCATTGTTAAGCTCGAAGAGAATCATTCCATCACC

CGTCCTGTGACTCTTAAGCTTACTTGTCCCAGTGGAAACACCCAAGAGAACAAGGTGGATTTGAAGCAGC

AGCCGAAGAAAGAACTGATAGGGC

>MSTRG.376.1 gene=MSTRG.376

CAACCTTCATTTTCAAAGGCCATTCTTTCATAATATATAAGGCTTTGGATATTTATCTTGTTCATTCTTT

CTATTAAAAAGAATAAATAATCAGACCAAAATCTCAATTCTGATCATTCACTAGCAATAGTACTGGTTCA

AGAAACTATAAGCCCAGATCAAGAATGGCTTATCTTCTCCAATTTCCAAACAATTTCAGGACCTTCTCTG

TTTCAGCTTCCTCCAATGGAGCTTCTCCAAGTGTGCCTCAGACAGGAGGGCCAGTGATTCTTGAACTTCC

CCTGGATAAAATAAGAAGACCTCTTATGCGTACCCGATCGAATGATCAAGAAAAGGTTAAGGATCTTATG

GATAGCATCGCGCAAATCGGTCTTCAAGTGCCTATTGATGTGCTCGAGGTAGATGGTGTCTACTATGGTT

TCTCAGGCTGTCACAGGTATGAAGCCCATCAGCGCTTAGGACTTCCAACAATCCGCTGTAAAATTCGGCG

TGGAACGAAGGAGACTTTGAGGCATCACCTCCGCTGAGTCCAAGAGCTGGGCTCACTTACAGGGCTGAAT

GATACAATTGCTCGACATCTATCTCACGGGGGTTTCACTACAAGTATTATTATAGCTTGTATACTTGAAT

ACATGCCCGATCCTGATCCTGATCACTCAATCAGAATATCAGATTGTAACTTATGTATGTATATAGTACT

CTGCTAATTGAAGATGGAGATTTGGAACGTTGCTTAGAACTGAATCCTTAGTATTATAGTATGCATCTGC

ATTATATAAAGCATACTACTACTATACTTTTTCTGACCATATGTAAAGAAGGCACACTTCATCCTTGGAA

TGTTTTAATTTTTTTTGCATGATTGCACATTTAAAGTTGACAATTGAGCACATTTACGGAACAAGTTGTT

GAGAAGGGCTTACTGAGTAATTCATGAAATACAATGTG

>MSTRG.376.2 gene=MSTRG.376

CTTTCATAATATATAAGGCTTTGGATATTTATCTTGTTCATTCTTTCTATTAAAAAGAATAAATAATCAG

ACCAAAATCTCAATTCTGATCATTCACTAGCAATAGTACTGGTTCAAGAAACTATAAGCCCAGATCAAGA

ATGGCTTATCTTCTCCAATTTCCAAACAATTTCAGGACCTTCTCTGTTTCAGCTTCCTCCAATGGAGCTT

CTCCAAGTGTGCCTCAGACAGGAGGGCCAGTGATTCTTGAACTTCCCCTGGATAAAATAAGAAGACCTCT

TATGCGTACCCGATCGAATGATCAAGAAAAGGTTAAGGATCTTATGGATAGCATCGCGCAAATCGGTCTT

CAAGTGCCTATTGATGTGCTCGAGGTAGATGGTGTCTACTATGTTTCTTTGTTTCTCCACCTACAGGTTT

CTCAGGCTGTCACAGGTATGAAGCCCATCAGCGCTTAGGACTTCCAACAATCCGCTGTAAAATTCGGCGT

GGAACGAAGGAGACTTTGAGGCATCACCTCCGCTGAGTCCAAGAGCTGGGCTCACTTACAGGGCTGAATG

ATACAATTGCTCGACATCTATCTCACGGGGGTTTCACTACAAGTATTATTATAGCTTGTATACTTGAATA

CATGCCCGATCCTGATCCTGATCACTCAATCAGAATATCAGATTGTAACTTATGTATGTATATAGTACTC

TGCTAATTGAAGATGGAGATTTGGAACGTTGCTTAGAACTGAATCCTTAGTATTATAGTATGCATCTGCA

TTATATAAAGCATACTACTACTATACTTTTTCTGACCATATGTAAAGAAGGCACACTTCATCCTTGGAAT

GTTTTAATTTTTTTTGCATGATTGCACATTTAAAGTTGAC

>MSTRG.377.1 gene=MSTRG.377

CCTTGAGCAGCGCCTAAAATAAAAAGTAAAACGACATCGATAATAGCCCTTCAGATTTCTTCTACTATCA

ATCTATCGCACTCTAATGGGCGACCGCGACGCCTATCGTGACCGCGAGAGAGATCGAGATCGAGATCGAG

AGAGAGACACCAGAAAACGCGACCGTGACCGTGAGCGCGACCGAGACCGTAGCAAACGCTCGCACACGCC

GGACCGGGCCAGATCGCGCCACGCCCGGTCGAGAACTCGCTCGCCGGACCGGTGGCGAAGCCGCTCCCGC

TCTCCCGACCGGCACAGGTCTCGCCGCCATCGCTCTCCCTCCCCGGACCACTCACGGAAGCGACAGAAGC

GCGGAGAGAGCAGTGAGAGAGACCGCCGGAGAGACTCATCCTCCGCCGCCGCCGCCGTGTCGGAGTTTGT

GGACGGGATAGTAAAGGAGAAGAAGGAGAAGAAGGGAGAGAGTGTGGAACCGGAGATGGATAAGGATGAA

GCTGAGATGATGAAGATGATGGGGATACCGGTAGGTTTCGATTCGACGAAAGGGAAGCATGTGGATGGCA

ATGATGTTAGCGGTATTAGAGCTGTTACGAAGCGGATGCCGAGACAGTATATGAATCGTCGTGGCGGGTT

TAATCGTCCCTTGCCTGCTGAAAGGAATCGTTGAGTTTTACTTAGGCCTAAGATGGAACATGGAATCAGG

CTAATGAATCAAATCAATTAGGGAATGAGCAGCGGCTTGGTATTGGCGGAAGTGAATTTTGTAAAACACC

TTTTGTATTCTTTGAATAGATGTATTGTACCTGAATCGGTTATTACTGCTAGTCAAGATAGTAGTCAGCT

TCGCCAGTCTGTTGTAACATACAAAATCGGTCTTCACATTGTATTTCATGAATTACTCAGTAAGCCCTTC

TCAACAACTTGTTCCGTAAATGTGCTCAATTGTCAACTTTAAATGTGCAATCATGCAAAAAAAATTAAAA

CATTCCAAGGA

>MSTRG.378.1 gene=MSTRG.378

TGCTCATCTTTCTCCTACGCTGAATATCATTAAAAAAATATACATCTTTCTTTGTTTCCACATGACGAGT

CTTAACTTTCCTCAAATGACCTAACATTACACATGTTCTTGGTCTTCCCAGCACCTTCCGCTTCCACTTT

GAGATCATACTCTTCAAACCCATTTTGCTCTCTCCTTTGTTTTTTTCCCGGAAAAAGACTAACTGTGCAC

AGCTTACCAAATAATCATGGTTATAAACAAATTTCTCACTGGCAATCAGGATTCGCCTGCAATTCTTTCT

GCTTCAGTGCATATTTGATTGTCCAAATGAATTAAATTCAAATTCAAATGAATTCAAATTCGCGCTGAAG

TACTCCAGCTAATTACAAATGGTTGACGGCTTCTTTAA

>MSTRG.384.1 gene=MSTRG.384

ATTTCAACAGCCCACTTAGACATCATCAAGATTTTGTCATCTGTCCCACAATTCAACATATGATGACCCC

TTTCTATAATGAAAAATAAAAATATAGCCGGATAGCCCTCCTAATTTTTCTATTCACATCGAACACTTCC

CCTCATCCAATCCAACAGTCACCGTCAAAAGTTGCTGGTGCCGTAGTTTTCGGACTTAACTCAGCTATAG

GCTACATGCTGATGCTTGCGATCATGTCGTCTAATGGCCGCGTTTTCATCGCGATTGTCGTCGGAATCGC

GGCCGGATATTTTATGCTTCGGAGTGGTGATGAGGATGATCAGGGGGGTGTTGATAATCCTTGTGCCTGT

GTCTGATTAATCTCCGTATGGTGCACAAATAACGAATCAAGAACAGATTGTCGAGTTTCATGAAACTTTT

AGTCTTTTCGACAAGATGCGATCTTTCCAGTACATAAAATTTTATGATTATGGTTATGTGATTATTTATA

GATTCACAGTATATATTGTTATAGCGTGGCTCATAATTTCATGATTAAGTATAATGGGGTGATGTGCTTG

AATCTATTAGTGTATGTGTTTTGTATCCTATTTTGTCATTCCTTAATCTATTTTGCCTGACTTGGAGGAG

AGTGGGTATGTAATCATTTTTATATTATATGTTTAGTTGC

>MSTRG.388.1 gene=MSTRG.388

GAAAGGAAAACAGTACTAAGTTAATACTTGTTCTTTCAATCGAGATAATGGCTCAAGCAGTGAGGTGTCT

TGATGGTCAGTCACTTTTGTGTCAGGCCAAGGCCGGCCAGAACATGCAGTGTCCCAGGAACCAGAACAAT

TCGAGTGTCATTGGACATCAGGATGCAAGGGAACTGGAAATCATTTGGGGTTCTGACACTCGCTACTGGA

AGTGGACAAGCATGCAAATCGGTGGACATTGCATTGAAGTTGCTGAATTGATTGATGTCTGCTGGCTGGA

AATCAATGGGAAATACGCGATGAAAAAACTTACTCCAGGAATGGATTACCAAGTGAAGTTCGTGTTGAAG

CTTAACAACAACTTTAACATTGACAAACCAGTGACCTTTAGCCTGACTACTCCAGAGGGATGCAAGGAAG

AGCATAAGGAGAACATGATGCAAATGCCAAGAAACCAAATCATTACCATCAATGCCGGGGAGTTTCAGAC

CCCCCGGAACAACTGTGGATGTGGAGAAGTCAAGTACCAGATGATGAACACTGATGGCTTCTGGAAGCGT

GGCCTGGTTGTCATTGGAGTCGTCATCGTGCCCAAATACAATTAAATAAAACCAGTATGCTAGTGAACTA

CTCCTAAATACCATCTCTATCAATAAATGCAACTCAGTTACATGGGTGGCTGTTACTAATCAGAATGATA

TGTAATAGATAACAACTACCTACTAATGGTGGCTTG

>MSTRG.391.1 gene=MSTRG.391

TTTTATGGATGTAGGAACTTATTGCCTAACAAGGTCATATCCATGGTGGTGATGGATAGGTTGAGAAGGG

GTGGAGAAGAAGAGCTAATTTGGCGATAATTGTGTCTTAATTAAGGATATTAATTATTCTTAATTAAGGT

TTTTAGTACCATTTATGGCGACCATTTATGGCGACCATTATTGGGGCATTATTGGCCCATAGAGGCATTT

AAGTGCCATAATGTTGGCTCGTTTATGTTTTTATTTTAATTTTAATTATTTTCAAATTCTCTCTCATTCG

TTTCATTCTGTTTTGTCTTATTTTCAGGATGTTTGAAAAAGATTTTAAGAATTTATTTATTAAGGCTGCT

TCCGAGAAGACGCGAACCTTGGTGTACAAGACAGTTGAGCTACAAACATAGATGGAAAGGAATAAAGAAG

ACATCATTTTTAGTCTGTTTTGTAATCGAT

>MSTRG.392.1 gene=MSTRG.392

AATACAGCTCCTTAAGTGGGACTTTTATATGGTATTGACTCTCTTATGTACTTGGTTTGAAACATAGTAC

TGACAATCTTCATTCATTTGTCCGAAACAGGCACAGGATTTCAGGACTGCAGGCACACAGATCCGTAGAA

AAATGTGGTTTCAGAATATGAAAATCAAGCTGATAGTGTTGAGTATTCTGATTGCCTTGATCCTAATCAT

AATACTGTCTGTCTGTCATGGTTTTAACTGTTGAAATTGAGCTGCACCTCTCTGGAGAACTCATTGGAAA

AAGCAGCCAAGTACTGATTTTGTCAAAGCTTTGTATTAAATGCTTGAATATACTTGTGTTAGTGTACTTT

GTATGTCGTAAGAAACCATTCACCTAAATTTGCTTCAGACATTTGGGCTGCAAGAACTTGCTAAAACGTG

CTTAGTATAGAAAAGCTGCTCAAGTTTGAGGTACTCTGATAAAAGTTGCTTTCTTCTCTATGTTAAATCC

CTAACTGTTGGCAACATTTCGTATTGGTACATACATCCGGGGATTTATTTGTACTGTGATTGTAAAATAT

TGTTGTTTATCTGTTCATAAAAGTTATGTATGCCTGAAAATTTTATACTCTAAACTACG

>MSTRG.392.2 gene=MSTRG.392

AAACAGGCACAGGATTTCAGGACTGCAGGCACACAGATCCGTAGAAAAATGTGGTTTCAGAATATGAAAA

TCAAGCTGATAGTGTTGAGTATTCTGATTGCCTTGATCCTAATCATAATACTGTCTGTCTGTCATGGTTT

TAACTGTTGAAATTGAGCTGCACCTCTCTGGAGAACTCATTGGAAAAAGCAGCCAAGTACTGATTTTGTC

AAAGCTTTGTAGTTAAATGCTTGAATATACTTGTGTTAGTATACTTTGTATGTCGTAAGAAACCATTCAC

CTAAATTTGCTTCAGACATTTGGGCTGCAAGAACTTGCTAAAACGTGCTTAGTATAGAAAAGCTGCTCAA

GTTTGAGGTACTCTGATAAAAGTTGCTTTCTTCTCTATGTTAAATCCCTAACTGTTGGCAACATTTCGTA

TTGGTACATACATCCGGGGATTTATTTGTACTGTGATTGTAAAATATTGTTGTTTATCTGTTCATAAAAG

TTATGTATGCCTGAAAATTTTATACTCTAAACTACGGGATCTTTCAGTAA

>MSTRG.392.3 gene=MSTRG.392

CAGGCACAGGATTTCAGGACTGCAGGCACACAGATCCGTAGAAAAATGTGGTTTCAGAATATGAAAATCA

AGCTGATAGTGTTGAGTATTCTGATTGCCTTGATCCTAATCATAATACTGTCTGTCTGTCATGGTTTTAA

CTGTTGAAATTGAGCTGCACCTCTCTGGAGAACTCATTGGAAAAAGCAGCCAAGTACTGATTTTGTCAAA

GCTTTGTAGTTAAATGCTTGAATATACTTGTGTTAGTATACTTTGTATGTCGTAAGAAACCATTCACCTA

AATTTGCTTCAGACATGTAGTTAAATGCTTGAATATACTTGTGTTAGTGTACTTTGTATGTCGTAAGAAA

CCATTCACCTAAATTTGCTTCAGACATTTGGGCTGCAAGAACTTGCTAAAACGTGCTTAGTATAGAAAAG

CTGCTCAAGTTTGAGGTACTCTGATAAAAGTTGCTTTCTTCTCTATGTTAAATCCCTAACTGTTGGCAAC

ATTTCGTATTGGTACATACATCCGGGGATTTATTTGTACTGTGATTGTAAAATATTGTTGTTTATCTGTT

CATAAAAGTTATGTATGCCTGAAAATTTTATACTCTAAACTACGGGATCTTTCAGTAA

>MSTRG.393.1 gene=MSTRG.393

CCTCCATCTGCTCCCCTAACAAGTCTTTAAACATCATATTCACCAGTCTTTGATAAGTAGCACCAGCGTT

CAAAAGTCCAAAAGGCATTCCAACGTAGCAGTAGAGACCACGGTCCGTGATGAAAGAGGTGTGTTCCTGG

TCTGGCTAATGCATTGGGATCTGATTGTAGCCGGAGTAGGCATCCATAAAGCTTAACAGGGCGTGGCCAG

CAGTAGCGTCAACTAGTTGATCAATCCTCGGAAGGGGAAAGCTGTCTTTTGGACATGCCTTATTCAGATC

TGTGAAATCGATACATACCCTCCATTTTCCATTGGCCTTCTTCACCAGCACTGGGTTCGCCAGCCATTCC

GGGTAATAAGCTTCTTTAACCAAACCAACCTTCTGAAGTCTATCGACCTCCGCTTTGGGAAAGCTGTCTT

TTGGACATGCCTTATTCAGATCTGTGAAATCGATACATACCCTCCATTTTCCATTGGCCTTCTTCACCAG

TACTGGGTTCGCGAGCCATTCTGGATAATAAGCTTCTTTAACCAAACCAACCTTCTGAAGTCTATCGACC

TCCGCTTTTAGTGCTTCTGCTCGTTCACCGCTGATGGGTCTGCGTTTTTGTCTGATACCTTTCTTCCTCG

GATCAATGTTCAAGCGGTGACACATAATGTTCGGATCAATTCCTGTCATATCAGCATGGCTCCAGGCGAA

TACATCAAGATTGCTTTTCAAAAAATCCACCATCTTCTTTCTCATCAGATTGTCAAGACGAGTACCAATG

AATAAGACTTTCGTTGCATCATTCTCATCAACCAGGATCCCAACTGTGTCCTCGGCTGGTCCTACCTTCT

GCAGTGTTTCTGGTAGTCTGGGGTCGAGATCGACATCTCTCAGATCGTCATCATCATCCTTCGGATGAAC

A

>MSTRG.394.1 gene=MSTRG.394

ATATGGTATTGACTCTCTTATGTACTTGGTTTGAAACATAGTACTGACAATCTTCATTCATTTGTCCGAA

ACAGGCACAGGATTTCAGGACTGCAGGCACACAGATCCGTAGAAAAATGTGGTTTCAGAATATGAAAATC

AAGCTGATAGTGTTGAGTATTCTGATTGCCTTGATCCTAATCATAATACTGTCTGTCTGTCATGGTTTTA

ACTGTTGAAATTGAGCTGCACCTCTCTGGAGAACTCATTGGAAAAAGCAGCCAAGTACTGATTTTGTCAA

AGCTTTGTAGTTAAATGCTTGAATATACTTGTGTTAGTGTACTTTGTATGTTGTAAGAAACCATTCACCT

AAATTTGCTTCAGACATTTGGGCTGCAAGAACTTGCTAAAACGTTCT

>MSTRG.396.1 gene=MSTRG.396

GTAGAAAAATGTGGTTTCAGAATATGAAAATCAAGCTGATAGTGTTGAGTATTCTGATTGCCTTGATCCT

AATCATAATACTGTCTGTCTGTCATGGTTTTAACTGTTGAAATTGAGCTGCACCTCTCTGGAGAACTCAT

TGGAAAAAGCAGCCAAGTACTGATTTTGTCAAAGCTTTGTAGTTAAATGCTTGAATATACTTGTGTTAGT

GTACAATTTGTATGTCGTAAGAAACCATTC

>MSTRG.398.1 gene=MSTRG.398

AAAGCATGTATCTTTGTGACCTAAAGGCTAAACCTGAATATGAGACCAGTTCATTAACTATCAAACATAT

CACACCCATCGCCGCTACCTGCTACAGCGAGTCGGCAACCTGCTGCAACCACATTCATCCAGCCTCAAGC

AATCAAACCCAATCTCATCCAGCTTCCAGGTCATCCGTTTTGTTTTTTGAATCGATCTCTGGCTTTTGTT

TCTTTGCGTGCCTGGACAAGTGGGGGATTTTATGCTTCTGTTATGTGTATAAGTGTATTTCAGAACTGGT

GAATGGTGATTCTTTATTTTTAATGTGCTGCAAGAAACCCATGTACTTTATTTTTAACTAGTTTGGCAGC

AATTTTCAGATTTTCATACACTATTTAGGAATTTCGGTTTATAAATTCGGTTTTCGGTTTTAACCGAAAT

TTAAAATTCGGTTCCGTTACAAAACCGAAATAATTTCGGTTCGATTTTCGGTAGACATTTTTTGTGAATT

TCGGTTTCGGTTAACCGAAAAAAAATTCGATTTCGGTTTCGGTTAACCGAATGCACAACCCTACTCCTAA

GTCCCAACTATCTCATGTACAGTTTTACTACCGAC

>MSTRG.400.1 gene=MSTRG.400

TTAAAACACAAAACCACAAGCCCTAAAACCCCAAATCTCTCTGCTTTATTTCACTAAACTTAGAGCGCCT

AGCCCTAGATTTCATCTCCTCGTCGACAATGAAGCTCGTCAGGTTTTTGATGAAATTGAACAACGAGACT

GTCTCCATTGAGCTTAAGAATGGGACAATTGTTCACGGTACTATCACAGGTGTGGATATCAGTATGAACA

CACATTTAAAGACTGTAAAACTTACACTGAGGGGGAAGAACCCAGTGACTCTAGATCACCTGAGTGTTAG

GGGGAACAACATCTGTTACTATATCTTGCCTGATAGCTTGAATCTTGAGACCTTGTTGGTGGAAGAGACA

CCGAGGGTTAAGCCCAAGAAGCCAACTGCAGGGAGGCCTGTGGGACGTGGTAGGGGACGTGGACGTGGGC

GTGGTCGTGGGCGAGGCCGTTAATTCTGATGTACTGCTACAATTTTTCCAATATTATGTTTCATTGCATT

GTTGGAGTTTATATGGTAATGTGCCAACAGTACCTAAAATGGACGATCGGAGGTTATTAGTTTAAGTTAT

GCACCGAGTG

>MSTRG.401.1 gene=MSTRG.401

TATATAAACTCTAAACATCTTTAACTTGTGAAACTCAACAACATAGGAGACTAACTCAATTTCAAGATTC

ATAAGATTATTATGGGGAAGGGCAATTCGGTGAAGACAGCAGCAGTGGTGCTTGGAGCCCTGGCTCTAGG

ATGGGCCACAATTGAGCTGGCTTTCAAGCCATGGCTTGACCAGGCTCGAAAAGCTATGGACAAGTCTGAT

CCTGCTAAGGACCCTGATGATGCCTCTACTTCTGCTCCTGCTCCTGACCCTGATGATGCCTCTACTTCTG

CTCCTGCTCCTGTTGGTGATGAAGAGCAGCCTGCTTCTTAGTTTTATGTATATGTGTGTTATTATGTATT

GGCTTCGGCCTGGATTAGCCTCCTAGATTTCGCTAATTGTGATGATTTATCGTTGGGATGTGGTTTGGAT

TATATGTGATGGATCTTTTGTTATGGAAATTTGTTTTCTCTGTATATTTGG

>MSTRG.402.1 gene=MSTRG.402

ATTGAGTTGAGAAGATCTTATCAAGCATATAATGTTATGCCTAGTTTAAGCAAAGCTTTAAAAAGATATG

GTGTATAACATCTGGAGACAGTCTATCAATTTTGTATCAAGAAACCCTCTATAAACGGACTGATACGGAT

CATCATCACAGAGATATAACCGAAGGTGAAGAGTCCAAGTCCTGCATGTTAAGAAACTATGGGGTGTACT

TTCGCCAATAGATCCCGTTGAGTAAGGTTACACCTAGTTCCCTACCCGACACCAGGAGGCTACGAAAGGA

TGAGACCCAGTCTTGATTGTCTGTTATGCCATAATGATCTAGTCTTGCTTCAGTTAGTAACAAAGATGAG

CTCTGCATATTCTGTATAGGTAGAAATAGTATTTGTTACCCTAAATGTATTACTGTCTGTTGAATGTTAA

TTGC

>MSTRG.404.1 gene=MSTRG.404

GTCATATTTCACAAGTCACCTGACATGAGTCATTTAGCGTGACCCTTCATCTCTCTCCCATGTGAAACCG

AAATGTAAAAAACAAACGGCTTAACAATCGATTGATGATCCGAACGAAAAGAAGTGAGCGTGACGCCTTT

GTGAACGCAACCAAAAAACCCAGCTCATCACTCATGACAACATAGCCCCCGTCACCTCCACGACTTCATC

TTCTTCCAACCTATACATACATTATACTTACAACTTTTTCATCATCCTAACAACTCTCTCTCCCCATTTT

CCTCATCTCCCTTCACTTTTTATACATTTTAATCATCTGGGCTGCTCTTAAATCTTTCTAAATTATCATA

ATTCTCACTATATATTCATTCTTGATTTTTGAAAATTTTCTTGGGTTTTCTTGAATTTTTGTGATCAGCT

CTGTTTATTAGTCTAAGATTCTGCTAGTTTGATCAAATTCTGTAGATTTTGCTGTTCAATTTTATTTATT

TAGTGCAATTTGTTTCGTTTTGTGCAATGTTCTTGATCAAATGTGATGCTTGGATTGTTAGTTAATGAGG

TGTTGAAGTGAATTGACGATGTTTTCAAGAAAATTGGTTAGGCTATTCATAAAGGGTTAGCGGCTTTGCG

GCGCAGCTTGTTGCTGGTTGAATGAATTGATTGTTACTTAAAAGGAAAAAAATAGGAATTGGAAATAGAA

ATAAAGGTTAAATTGTGAATTTTGAGCAATGGAGAATTTGACTGAACCATCATCATCGATAAGTTTTAGT

TCTTCTAATGGCTCAACTGGCTACACTTTAACCGAATCATCTGTCTCGGAGAAAGTATCTAGTCTTCAAG

TTGTGAGTTTATATAAGCTTAGTGCTAGCTTAGAGCAACTTTTGATTGATAGTGTTGGTGAGTATAGTGA

TGCGGATATTGTAGTTGAAGATATTGCTGTAGGAGTCCACCGTTGTATATTAGCTGCTAGGAGTAAATTT

TTCGATGCACTTTTTAAGAAAGAGAAGAAGGGTGGTTCTGCGGGGAAGGGAAGTAGACCGAGATATTGTA

TGAGTGATATGTTGCCATTTGGTAATGTTGGATATGAGGCCTTCTTAATTGTCTTGAGTTATTTGTATAC

CGGAAAGCTGAAGCCTTCTCCAGCGGAGGTGTCAACTTGTGTTGATGATGGATGTGCTCATGATGCCTGT

CGACCTGCTATTAACTTCTCCGTGGAATTGATGTATGCATCTGCCATATTTGAGGTCCCGGAGCTGATTT

CACTTTTCCAGCGGCGTCTTTTCAACTTTGTGGAAAAGGCTCATGTTGAAGATGTCATCCCAATCATCCT

TGCCGCTTTCCACTGTCAATTAACTCAGCTTCTCGATCAATGTATTCATAGAGTCGCACGATCAGATCTC

GACAGCATTTCTCTTGAGAAGGAATTCCCGTTTGAAGTTGCAGAGAGAATTAAGTTGCTCCGTGATAAAT

TACAGGGTGTTAAGTTACAGGCAGATGATAGTGCCATGATACCCGAGGATCCGTTGCGTGAAAAGAGTAC

AAGGAGAATACACAAGGCTTTGGACTCTGATGATGTTGAACTTGTAAAACTTCTTCTGACCGAGTCTAAT

ATAAGTTTAGATGAAGCCAATGCCCTGCATTATGCTGCTGCATACTGTGATCCAAAAGTTGTATCTCAGT

TGCTTAGTCTGGATCTGGCTGATGTCAACCTTCGAAATGCACGGGGCTACACAGTTCTCCATGTTGCTGC

ATTACGTAAGGAACCATCAATAATTGTACCACTTCTAAGCAGAGGAGCTTGTGCCTTAGAAACAACACTA

GATGGACGGAGTGCTGTCAGTATCTGCAAGAGGCTGACAAGGCCAAAGGATTTTCATGCTAAAACAGAAA

AAGGCCAAAAATCAAACAAAGACAGATTATGCATTAATGTTTTAGAGCAAGAGGTGCACAGGAATCCAAT

GGCTGGAAATTCATCAAGGTCCTCTTCAAACATAACTGATGATCTGCACTTGAAGCTTTTATGCCTTGAA

AACAGAGTGGCATTTGCACGGTTACTTTTTCCGTACGAGGCCCAGCTAGCCATGGAAAATGCCAGCGCAG

ACACAACGTCTGAGTTCTCTAGCATTTTGGAATCCAAAGTTTCAAGTGGCAATTTAAGAGAGGTAGATCT

GAATGAGACACCCATAGCACAGAACAAAAGACTTCTCTCAAGGATGGAAGCCCTCTCGAAAACAGTTGAG

ACAGGTCGGCGGTATTTCCCACATTGCTCGGAAATCTTGGATAACTTTATGGTGGATGATCTACCTGATT

CGTTCTACCTTGAAGAGGGCAGTGCAGAAGAACAGGAATTCAGAAGAAAGCGTTTTATCGAGCTTAAAGA

GGATGTTCAAAAAGCATTTACCAAGGACAAAGCTGAGTTAGAGAGGGTTGGCTTGGGCTCTTCACTATCC

ACATCCTCTAATAAGAAGAGACGTAAATTTCAAAGTTGAAAATTTTATATATGTATCCAAGTTAAAAATT

TTATATATGTATCCGAATTTTGTCATTCCTGACATACTGTGTATAAACTGAGTCATCTACATGAAAATTC

AGATGTAAGATTGGCAACTTTA

>MSTRG.410.2 gene=MSTRG.410

TGAGTTTTCTCCGATTTATCTCTTCTCAATTTCTCCATTAATCTCATCTCTTTCATCAATTTCTCAAGCA

CCTTGCTTAATTAGTTCTTTAACTTGCATAAGATATATAGATGATTTATACATTCATGATCCATGTGTGT

AAATCTAATATTCTAATGTAGATGAACTTGTTTTGCAGACATGTGTGTGAATCATGACTTGGAACAGCAA

CACGATGTTTGTACACATGCAGATGCTGATCAAACACTATACGTCGATTATAAAATCTAGATGATTAACA

CTGTTCTTGTGTGTCTGTATATATCTAGACATGACTGATTACTACTGTGTGTTTCAGTTTCAGTCGGCTT

CGTAATTACATGTACATGCATCTAGGTTTTTCCCTATGATTTTTTTACAACATAGATAGATCTGTGTCTG

CATGGTGACACAGACTACTCCGAAAATAAACTTCTTGCTTTGTATATAACATTTCAGTTTTTATTCAATG

TATTATAGGTGACAGGATCTAGGATTCTTGTTCAGACAGCACTAATTCTGATTAAGATAAAAGGAAGACA

TGGGTCAGGTGAGGTGCAGCTTTAAAATATTCTGTTGCTCTTCCAATCCAAAAAAGAAAGTAAAAAGGAA

TATTACAATGCTGTATAGTGTTTGATGGAACAAATTCCCATCCCATTATCAAAAAGATGCCTGGCCTTTT

ACATTTTGTTTCTGATTCGCTTAGCCATCTTTTTTACTTTTTTGTTCTGGTATCCACTTTAGTTCGACAA

AATATGAC

>MSTRG.413.1 gene=MSTRG.413

AGGTGTTCAGTTTGTTCAAACGAGGGACTCATTTATGATTTCTTTGGCGGCAGTCCTGTAGGCATACACC

AAAAGCGGAAATTGGGTCCTCAATATATAGTTCTTGTGATTGTAGTTTTATAGCACATCTAGGCCTAGTA

TAATTTGTTTTGGAAAGAACAATTCTGGTGATCTTTTTAAACTTTGCCTATTGTTCTTTTTTATTCTCAG

AAGTAGCTCAAACCTTATCATTCTTTAAAAGTGCCTTAAATTCATCAATGAGGTGTTGTGTTTATTTTCA

A

>MSTRG.415.1 gene=MSTRG.415

TTTAAATTAGGGAAGAAATGAGGGGTGCATGGAGAGTGCTAAAGTGACTAGATTTACAGTACTTGAACTT

CTTCCGTACGCCAGTGATAGACCCGTTTACTAACCCGAATTTGCCACCATGTCCACCGGAGTTCTCCCCG

TCGATTTACTCGAGGAGATCTTCAGCAAGCTTCCAGTGAAGTCCCTAATCTCCCTCACCATCATCTGCAA

ATCCTGGCTCGCACTAATCTCATCACAATCTTTTGCAAAAACCCATCTTTTCAAGTACCCTCTTGACCCC

TCTTCTCATTTGCTTCTTCTCCACCGCCCCTGTCGCGAAACAATCACCATTTCCGAGCTCAATTTCTCGG

GATTGCCAAGAATTGTGGAAACCCCAATTCCAGAATCTGAACCCCCGCCGATTCAAGCCCCGTCGACTCC

GGTGGTCTCGATCTGTTGTGCGAGAAATGTTCAAGTTCGTCAAGAAGTTAGGAGCTGTTTGTTGAGATTG

GTGGGCTCGATTAATGGGTTGGTGTGTTTTTCTACGCCTACTCTTAGGCCTACTAATGTTGTGATTTGGA

ACCCTGCTACTCGGAGATTTAGGGATGTGGCGGTGTCGCATTTGAATTATAGTAACCCTTTGAGGATTTA

TGTGGCTTTTGGGTATGATGGTGTTGGTGATGATTATAAGGTGGTCTGTGTTTATCGGTTTCGGGTTAGG

AATAATCCGGAGATGTCGTTTAGGTTTAGGATGTTTTCGTGTAGGGATGGTTGTTGGAAGGATGTAGAGC

CGGGGTTCGGGTTTAGTTTGGGGTTTATAGTGGGGTGTGTGATCGTAAAGGGGAATCCTTTTTGGATGGG

GTTTTACAAGGATAAAGAGATTTGGCTGACGCTTGATGTTCAAACTGAGGTGATCCGGATGTTTTCTGGG

CCGAAGTATGTAAGGGGTGCATCTACTTCGATCTCAATTATGGCTTTAGGTGATAATGCTGCTCAGATTG

TGTATTCACCCGGAATTGAATCAAGTCATATGATTCATGTGTATTGTTTGGAAGAAAGTAGTGGTACGTG

GAATTTGATGTACGCCATAGAGTCGATTGGGTTACAGAGGCCTGTACACATGGAATGCTATAAGAATGGC

AAGGTTGTTACAAAGGATAGGAACGGCAAGTTGTTCACGTATGACTTGATAAGTAAAGAAATTAAGGATC

TTGGGGTTGGAGAAGGCATGGAGGATCATTACATCGCCATTAATTACATCGAAAGTCTTGTTTCCATCGA

AAGTATGGAGAAAGTAAGAGAAGAAGCTGCTGAAGAAGCAGGGCAGGAGACTGGACTTGCTTCAAATGAT

AGGGATGCTTCGGATGCTATGATGTCAGTAAGGATAGATGAATCTCTTAAAGATGTATGAGAATCAAGGT

TACTGTTGCGGGTTGTTGGCTTTCAATTGAACTGAAGAAGTATACAAGCATGGAGGGACTGAAATGCTGT

TTCATGTACAATAGACTATTGTTGTATAATCTTGAAAAAATTGTATTATGTATTACTAGCTGTTTTCTGT

ATCAGAGACCTCAGCAAGTCTGTTGTGTTTCTATTTTTTTTGACTTTAAAATACAAATTGTGTTGTCTGA

TACTATCTGAGCTTGATCTTGATTTATGTTTACTTGGTTCTGGTTGGGTTCGAAACAGATATATGGTCAT

TTTTTATCTTGCCTTTGTATGCATAATACTGGAATGTGGTG

>MSTRG.415.2 gene=MSTRG.415

TTTAAATTAGGGAAGAAATGAGGGGTGCATGGAGAGTGCTAAAGTGACTAGATTTACAGTACTTGAACTT

CTTCCGTACGCCAGTGATAGACCCGTTTACTAACCCGAATTTGCCACCATGTCCACCGGAGTTCTCCCCG

TCGATTTACTCGAGGAGATCTTCAGCAAGCTTCCAGTGAAGTCCCTAATCTCCCTCACCATCATCTGCAA

ATCCTGGCTCGCACTAATCTCATCACAATCTTTTGCAAAAACCCATCTTTTCAAGTACCCTCTTGACCCC

TCTTCTCATTTGCTTCTTCTCCACCGCCCCTGTCGCGAAACAATCACCATTTCCGAGCTCAATTTCTCGG

GATTGCCAAGAATTGTGGAAACCCCAATTCCAGAATCTGAACCCCCGCCGATTCAAGCCCCGTCGACTCC

GGTGGTCTCGATCTGTTGTGCGAGAAATGTTCAAGTTCGTCAAGAAGTTAGGAGCTGTTTGTTGAGATTG

GTGGGCTCGATTAATGGGTTGGTGTGTTTTTCTACGCCTACTCTTAGGCCTACTAATGTTGTGATTTGGA

ACCCTGCTACTCGGAGATTTAGGGATGTGGCGGTGTCGCATTTGAATTATAGTAACCCTTTGAGGATTTA

TGTGGCTTTTGGGTATGATGGTGTTGGTGATGATTATAAGGTGGTCTGTGTTTATCGGTTTCGGGTTAGG

AATAATCCGGAGATGTCGTTTAGGTTTAGGATGTTTTCGTGTAGGGATGGTTGTTGGAAGGATGTAGAGC

CGGGGTTCGGGTTTAGTTTGGGGTTTATAGTGGGGTGTGTGATCGTAAAGGGGAATCCTTTTTGGATGGG

GTTTTACAAGGATAAAGAGATTTGGCTGACGCTTGATGTTCAAACTGAGGTGATCCGGATGTTTTCTGGG

CCGAAGTATGTAAGGGGTGCATCTACTTCGATCTCAATTATGGCTTTAGGTGATAATGCTGCTCAGATTG

TGTATTCACCCGGAATTGAATCAAGTCATATGATTCATGTGTATTGTTTGGAAGAAAGTAGTGGTACGTG

GAATTTGATGTACGCCATAGAGTCGATTGGGTTACAGAGGCCTGTACACATGGAATGCTATAAGAATGGC

AAGGTTGTTACAAAGGATAGGAACGGCAAGTTGTTCACGTATGACTTGATAAGTAAAGAAATTAAGGATC

TTGGGGTTGGAGAAGGCATGGAGGATCATTACATCGCCATTAATTACATCGAAAGTCTTGTTTCCATCGA

AAGTATGGAGAAAGTAAGAGAAGAAGCTGCTGAAGAAGCAGGGCAGGAGACTGGACTTGCTTCAAATGAT

AGGTATCCCGACTTCTCTTAGGATGCTTCGGATGCTATGATGTCAGTAAGGATAGATGAATCTCTTAAAG

ATGTATGAGAATCAAGGTTACTGTTGCGGGTTGTTGGCTTTCAATTGAACTGAAGAAGTATACAAGCATG

GAGGGACTGAAATGCTGTTTCATGTACAATAGACTATTGTTGTATAATCTTGAAAAAATTGTATTATGTA

TTACTAGCTGTTTTCTGTATCAGAGACCTCAGCAAGTCTGTTGTGTTTCTATTTTTTTTGACTTTAAAAT

ACAAATTGTGTTGTCTGATACTATCTGAGCTTGATCTTGATTTATGTTTACTTGGTTCTGGTTGGGTTCG

AAACAGATATATGGTCATTTTTTATCTTGCCTTTGTATGCATAATACTGGAATGTGGTG

>MSTRG.416.1 gene=MSTRG.416

TTTCTGTATTATATGTAAAATGCTAGGATAAGTATTTTATTTAGAGAAGGGAGGGAGAAGAACAACAGTG

AAAGAGATAGGAAAATTGTGGCAAAGATTATTACTATTACATGTTCTTCTTGGGAAACTTCTTTACACTT

CTCCCAATGCAATAAAACAAGAGTGGTTTCCCATTTTCTTGGAGAAGAACAACATAATCTTGTATCCCCA

GCTTTATCAAATCCTTGGTTTTCTCACAAATTTCTTCTAGCTATGCAGAGTCAACTAGTGTGCAGGGGAT

GTAGGACGATGCTTCTGTACCCACAAGGAGCAGCAAATGTTTGTTGTGCAGTGTGCAGCGTAGTTACTAC

CGTACCTCCTCCAGGAATGGATATGTCCCAACTAATATGTGGAGGTTGCCGTACATTGCTGATGTATACT

CGTGGAGCTTCAAGTGTGAGATGCTCGTGCTGTCACACCGTGAGCCTTGTGCCAGCACCAAACCACCAAG

TTGCTCACATCAACTGTGGCAATTGCACCACAACACTTATGTATCCCTATGGATCTCAATCTGTCAGATG

TGCCATTTGTCAATACGTAACCAACATAAATATGGGTAATACAAATATCCCTGTTCCTGCAAGCAGACCC

GATGGAACAGCTTCATCTGGAACAACAACTGCTACTTCAACAACTCAAACAGTTATTGTCCAGAATCCTA

TGTCCGTTGATGAAAGTGGAAAAACAGTGAACAACGTTGTTGTTGGTGTTACAACTTGAGAAAACTGATT

TGATTGTAACAACCGAGCTTCAAGACTCTTCAAATCTGTATCTGTTGGTCAGTGACTACTTGTGTTTTCA

AATGTGACATGCATAATAGGACGGTAACATATCCTGCCATTTTTCAGGACGGGAAAATTTTCTTTTTGGA

TGTAAAATGATGTAAAAACGACTTAGAACTATAGAGGATGGTGATGGCTAGTAATTAGTTGTGTGAAAAT

CTTTTGTTTTTATCGGCAATCAAGCCAGTTGCCTGGAATATATATGCAAGTGCAGAGAAAGAGGGGCTAA

TGTGCACCTGTCTTTGTTAAATAAAATTTAGTTTATTCATTGTTGTTGTAGATTGTTGTCTGATGTATGT

TCTGTCAGCTACTGACTTCCTTGATTTTATGGGAAGAATAACTTCTCTTGATATATATAAATGAATTTTC

ATTCTA

>MSTRG.417.1 gene=MSTRG.417

TTTAAACGTATATAATAAAATATATAATTAACCCGACCCGGAAACCTTCGTAAGCCTGTGACAGGTCGAT

TTTGAGCAAAAGAAAGCAGAGCCTCCGTTTAAAGCAGGGTAAGAAAGTTTGAAGCTTGACTGTCTTTTAG

AAGTTGAGATGGCAGTTAAAGATTTTGTAGAGGTCTGGCATTTTGTGTGGTCAATGGAATTTTGGAGGAT

GGGAGTTTGTTGGACATTGTCTCTTCTTTTGGCGTATGCTCGATTGCTTGGTCAGAGGTTTTTGACTCGA

GGGAACAAATCGTATGGTCGTTGTTGGAAACACAGTGGTGCTAGAAAGCCTGTCTGCGTGATCACAGGCG

CAACGTCTGGTCTCGGTGCTGCAGCAGCAGCTGATCTGGCAAGGAATGGATTCTATGTTGTTCTTGTTGG

AAGATCATCTCATCTGCTATCGAAGACTATTTCTGAGATCAGATTGCAAAATAAAGCTGCGGATCTCAAA

GCATTTCATCTAGACTTATCATCATTTGGGTCAATTTTAAAGTTTGAAGCCAACCTTCAGCAGTGGCTTC

TGGATTCAGATTTACATTGTTCTATACAGATCTTAATTAATAATGCTGGAATACTTGCAACGTCATATAG

ATGCACTCCTGAGGGCTATGATCAGATGATGGGAACTAATTATATTGGCGCATTTGCTCTAACAAAGGTT

CTACAGCCGCTATTGGAAAAAAGTCCTACCCCTGCTCGGATTGTTAATGTTTCATCATTTACACATTGGA

ATGCACTATCTGGCATTCGCGTTGACAAGGAAACTGTATCTGGGATGTCCTTTTCAAAATTGAAATGCTA

TCCTTATGCTCATATATACGAGTATTCAAAATTATGCTTACTACTATTCACTTACGAGCTTCACCGACAG

TTTGATATCTTGCAAAAGCCACATCAGATATCTGTAGTTGCCGTAGATCCTGGAGTAGTGAAAACCAACA

TTATGCGAGAAATTCCTTCATGTCTCTCAGAGTCTGCATTTTTGGTCCTGAGAGTTCTGGGCCTGTTGCA

GCCACCTGAAGTTGGGGTAACCTCCATTGTTGATGCAGCCCTTTCCCCTCCTGAAGCATCTGGATCTTAC

TTCTTTGGTGGGAGTGGCAGAACTCTTAAATCCTCTGAACTTTCTTATGACAAAGACTTTGCAAAAGAAC

TCTGGAAAACCTCATGCGAGTTGTTCCTAGATGCACAGCAGCTATCTCAGGAGACTTCCAGATGATCTCA

AGGTGTATAGATGCGATTGAAATGTTCTTGATAGTATGTACTCTATGCTTTTGTGCTGAACGAGTATCAG

ACCGCAAATGTACATAAGCATACACCTTGTCATCATATCACATTGAACACAGTTTTCTAACACATTTATT

CATACAAAATTGTACAATTAGATTCATCTTCTGTGATTTACCTTGTAGATATTATTAACCAAC

>MSTRG.424.1 gene=MSTRG.424

TATGATTCATACTCTCTATTTCTCCAAAAAATTAGATCAAAAGATGAATCCAAACAACACTCCTCCTACA

AATTCTCAAAATTCAAACCCTCAATTTCCATATCCATATCCAAATCCTTTTTTCCCAAACACACAAAATT

TTAATTTCAATTCTCAAACCCCGAATTATAATGCATAATATGATTGTTGAAGACGAGAGAGACACTTATG

CCACGCAATTTGGTCCTTTACCAATTTATGATGATGCAACAAATGGTTTATCTCAACCAAATTTAGGTGA

AGAACCCTTTATCCCGTATGAAAGGTATATCCAAAATACGTTACAAATGCGTGATAAACGGACACATCGT

CAGCTACAAAATGACTTGGTTGAGCACATCTCGCAGTTCCATAATAGTCGTTAGTTTCTTATTGTACTAT

TTTTAATTGTTTGTTTCCTTGTGTAATGTTTTTAATTTATTGTTTTTAATTCAGTTGAATGTATTTTATT

TTAATA

>MSTRG.426.1 gene=MSTRG.426

AAGTTTTAAAATCTTAATCGTGATCAGCATGATTAAACTGAAAATGACCTGCAAAAAAAAAATTGAGACC

AGCTTATGATATTATAACCACACGTAACTATTAATCTAGCAGTATCACTTAATCTGTGCTATTAAATATT

GCTTTTTCCTCGATGAACTTATTGTTATGTCACATTCGGAATCAGAAAATACAGTATATGTTGTAATCAA

TCTGATCTGGTAACTATTCTGCAGTTTGACAAATTTTATTGAAGTGACAGATGGACATTAATGTTGCAGT

GAGGGGTTATGGCTGGCTTATGCTCTTAGATTTTGATGTTTTCGATTTTGTTACATATAAAATAAACTGG

TAACATGTTCATAAAATGATTGAGGTACAGATTAGTAAACTGATGATCATCTAGAAATAATTTGACATCA

CGGTTGTCTTGATAATTTCCAGTTGTTCTGTTCTGAATTCTGAGTGAATTGGAATACATCAAATATACAC

AACAGTTTCAGACATTCATACGTTTATTTATATGTGAATATGTACCTCATGGTAAATATGTAGATGCTCT

TCAAGATACTTGAGCACAAAGGTAATACTCTAACAGAGCTGGTAATCTGCAGTTCCATTGGCTCAGAGGG

CAGGATATCTGTTCAAGATCCAGTATATTTCCTCCGTCTCATTCAATTGTATACGTTACTTTTTGGCACG

CCTTTCAAGACTCTTGTAAGGTATAATATTATTTTTTACTGAAAAAAATTTGATGCTTAAACTTTTATTC

AAAAAAGAAAATTTTAAAAAAACATTATGGAACTATACTTTATAGGAGCATCAAACTGTGTGCAAACAGT

GAACGTATACCATTCACCGGACGGAGGGAGTATTTTGTATTGGACTCATCATGGAAAAACTAATGCAGAT

CAAAATATGCAGAAGGCCAGAGAATTTTTTGAATTCATGGCTCCATATGTGACCAGCTCTCCAAGGGAAG

CATTTTTGAACTACAGAGATCTTGACATTGGTCAGAATCCAATAGGCAATGCAAGCTATGCAACTGCC

>MSTRG.426.2 gene=MSTRG.426

AAGTTTTAAAATCTTAATCGTGATCAGCATGATTAAACTGAAAATGACCTGCAAAAAAAAAATTGAGACC

AGCTTATGATATTATAACCACACGTAACTATTAATCTAGCAGTATCACTTAATCTGTGCTATTAAATATT

GCTTTTTCCTCGATGAACTTATTGTTATGTCACATTCGGAATCAGAAAATACAGTATATGTTGTAATCAA

TCTGATCTGGTAACTATTCTGCAGTTTGACAAATTTTATTGAAGTGACAGATGGACATTAATGTTGCAGT

GAGGGGTTATGGCTGGCTTATGCTCTTAGATTTTGATGTTTTCGATTTTGTTACATATAAAATAAACTGG

TAACATGTTCATAAAATGATTGAGGTACAGATTAGTAAACTGATGATCATCTAGAAATAATTTGACATCA

CGGTTGTCTTGATAATTTCCAGTTGTTCTGTTCTGAATTCTGAGTGAATTGGAATACATCAAATATACAC

AACAGTTTCAGACATTCATACGTTTATTTATATGTGAATATGTACCTCATGGTAAATATGTAGATGCTCT

TCAAGATACTTGAGCACAAAGGTAATACTCTAACAGAGCTGGTAATCTGCAGTTCCATTGGCTCAGAGGG

CAGGATATCTGTTCAAGATCCAGTATATTTCCTCCGTCTCATTCAATTGTATACGTTACTTTTTGGCACG

CCTTTCAAGACTCTTGTAAGGAGCATCAAACTGTGTGCAAACAGTGAACGTATACCATTCACCGGACGGA

GGGAGTATTTTGTATTGGACTCATCATGGAAAAACTAATGCAGATCAAAATATGCAGAAGGCCAGAGAAT

TTTTTGAATTCATGGCTCCATATGTGACCAGCTCTCCAAGGGAAGCATTTTTGAACTACAGAGATCTTGA

CATTGGTCAGAATCCAATAGGCAATGCAAGCTATGCAACTGCC

>MSTRG.431.1 gene=MSTRG.431

CNGCAACCAAATTGGCGCCAAGTTCTGGGAAGTCATCTGCGATGAGCATGCAATCAACCAGTCAGGATTC

TACGATGGAACCACCGATCATCTTCAGCTCGAGAGGATCAACGTGTATTATAACGAAGCTAGTGGAGGGA

GGTATGTGCCTAGAGCTGTGCTGGTGGACTTGGAGCCAGGGACTATGGATGCTGTGAGGACAGGGCCTTA

TGGCCAGATTTTTAGGCCGGATAACTTCGTTTTCGGGCAGTCGGGTGCGGGGAATAACTGGGCTAAAGGG

CATTATACGGAGGGGGCAGAGCTGGTTGATTCTGTTCTTGATGTTGTTAGAAAAGAGGCTGAGAATTGTG

ATTGTCTTCAAGGATTCCAAGTATGTCATTCTCTGGGTGGTGGCACTGGATCTGGGATGGGCACTCTTCT

CATTTCTAAAATCCGAGAGGAGTATCCAGATCGGATGATGCTAACATTTTCAGTCTTCCCTTCTCCGAAA

GTATCTGACACCGTTGTTGAGCCATACAATGCCACACTTTCTGTTCATCAGCTTGTTGAGAATGCAGATC

AGTGCATGGTCTTGGATAATGAGGCACTCTATGACATCTGTTTCCGAACCCTCAAGCTTACTACCCCCAC

ATTCGGTGATCTCAACCACCTGATCTCTGCTACCATGAGTGGTGTCACATGTTGCCTTAGGTTTCCTGGT

CAGCTGAACTCTGACCTAAGGAAACTAGCAGTTAACCTCATTCCATTTCCGCGACTCCACTTCTTCATGG

TTGGTTTTGCGCCCTTGACATCAAGAGGGTCCCAGCAATATCGTGCTCTCACTGTCCCAGAACTGACCCA

ACAGATGTGGGATGCCAAGAACATGATGTGCGCTGCTGACCCACGAAATGGTCGCTACTTAACAGCGTCA

GCTATGTTTCGTGGTAAGATGAGCACAAAAGAGGTTGATGAACAGTTGATTAATGTCCAGAACAAGAACT

CATCATACTTTGTTGAGTGGATACCGAACAATGTCAAGTCTAGTGTCTGTGACATCCCGCCAAAGGGCTT

AAAAATGTCTTCAACATTTATTGGCAACTCAACCTCTATTCAGGAGATGTTCCGACGGGTTAGTGAACAA

TTCACAGCCATGTTCAGGCGAAAGGCTTTCCTGCACTGGTACACTGGTGAAGGAATGGACGAGATGGAAT

TCACAGAGGCCGAGAGTAACATGAATGACCTTGTGGCTGAATACCAACAATACCAGGATGCAACAGCTGA

GGAGTACTACGAGGATGAAGAAGAAGCAGAAGCTGAAGCTTGAGTCTAAGATTGGTGCATCTTTGGATCT

CTCTCAGCAGGTTCATATGTGCTTATCATTGTGAATGAATGAAATAGTGTGACATAGTTACTCTGTATTG

AATGATATGTCAGATTGAAGATTATGTGTTTTACATAAATGACCGGAAATGTTTGTATTTAACAGAATGC

TGCTTGGGGTTTGAAGTATACTGCAATTTCTTGCCTTA

>MSTRG.432.1 gene=MSTRG.432

GGAAAAGTGAATCTAGTTTGGACTGACAGTCCCACTAGCTAGACCCCCTTTAGGCTTTAAGCCTGTGAAT

CCTCTGGGAGGATTTTTGAACCTCACTTTGTAGTCCTTTTTCCAGAACATCAATAGCAGAAGATCAGTTG

GTTCCAAGGATGCTGAAGTTTGTAAACTGAGAACTGAGGATTTGGATCTTATGTACTAAAATAAGATCTT

TCTTGTCCATAACACAAGTGGTAGAGCATATTTTGCGACATTCTTCTAAAAAAATCACTGGATTTGAGTG

CAAAGCACCAAGAGTCAATGGATATGCTTGCTTGGACCCGAAACTTGTTAAAGCAGACCACTTCTCAGTA

GGAGGGCTGCATCTGGATGGCAGCACGTCGAATTCCCTTGGCACTTTTGTTAACAGAATCACCGTAGCTG

AATTGCCTGGACTGAATACACTAGGTCTGTCTAATGTACGTGTCAACTATGCACGACATGGTGTAGTCCC

TCCCCACATACACCCTCGTGCCACTGAGATATTAACAGTTCTAAAGGGCCGCCTTCGTGTTGGATTTGTC

ACTGGAAACCCTGAGAATAGGCTTATAACAAAGGTTCTTGGAGAAGGTGATGTGTTTGTCTTCCCTATGG

GGCTTGTTCACTTCCAACAAAATGTTGCAGAGGGAAATACTACTGTACTAGCTTTCTTAAGCGCTGCAAA

CCCTGGGGTGATCACAGTCGCCAATGCTGTTTTTGGATCAAATTCACCGATAGGTGATGATGTACTAGGA

AAGGCATTCCAGGCAGATGCTAGGACCATTAAAGCTTTCAAATCTCAGTTTTAAGTATTCATTCACAGAT

ATGTAGTTTGCTAGTATGAGTCTATATTGATTTTTTTTATTTGTTTAAGTTATAATAAAATTTGTTTCTT

TACAATGGA

>MSTRG.437.1 gene=MSTRG.437

AAGTGGGTCACCTTTTTTCCAAGCCACCACTTGAGATTTTTCAGGCTCATCGAGAGACAAAGAATGGATG

GCTGGAAAAGAAGCTAAAAAAGGCTCGTATTTGGTCAAAATGGCTTTGACCTCGTTAATTTCCTGGACAG

TGAGTGGGTCAAGTGGGTGGAGTGGGATGGCATCGGTGATCTCCTTCCATTTCTGGTTCGGGTTTCGGTT

CGGGTTCGAGTTTGATTGACGTGAGCCTGCGGGCTCGCGGCTCGAGAAGGTGCACCTGAGTGTCTTGGAT

CCGGTGCAGTCAAAGTAGCCAGGTTTACTCAAAGTATACCATGTTACTGCAGAGATGAAAAGCATGAAGA

TGAGGAAAGAGAGGCGATTTAAGGAGCAAATCTCCATAACTTAACAAGTCTTCTCTCCACAATTTGGCTC

TTCTTTTTCTGCATATACATATAATATAGAATTCTTGTACGTCAGGCTCAGCGCTCCCTCT

>MSTRG.442.1 gene=MSTRG.442

GTTTAATGTGCCCTGGAGACTCTAGTGTTACATTTTGGGTTCAGGGAGTTGAATGTTGAGAGAGAACTTT

CAGAGTTCAGAAACGACATCTCCGGCCCAGGAAAAGACTTGGGGCAACATACATTTCAGTACTTCCATTC

AGCAACGAAACCAGGGGCAAGGTATCATCTGCCTAGATAAAGACTTGGAGCAAAATACATTTTAGTACTT

CCATTCAGCGATGAAACCAGGGAC

>MSTRG.450.1 gene=MSTRG.450

CACGTTGTCAATTTTAAAAAATCAACACATACCACACGACCCCAATCGAGCTTTGATTTCTGCTCAACCA

CCCACCACAACCAAAGAAACCAATTCTGATTCAGTTCGTTTCAATAATTTGATCTTAAACTAAACCCGAA

ACTCAGAACCTCTGGATCTTATCAGCCTTAACAACCGGATTAGCAACCTTAATAGCTTCCTTTCCGACAG

TCTTGAAATCAAACTCACACTCATGCTTCTCCGGATACCTATGGACCCCACAGTACACGTCCCCACACTT

GCACTTAAACCCCAGAACTCCCACCTTCTTCCGACAGCTCTTACACTTGTTCGCCGACTTCACCTCCTCA

TTCTCCGAAACTCTCGCCGAAGATTCCGCCACATGAACCAACTGAACCGGCTCAACCACTTTCGGCTTCA

CCGAAAACCGGTCGACTATTTTATCCACCGCTTTTTTCGCCGAAGCCGCTTGCTCTTCATGAATAACAAG

GTCTCTGTGACACTTTGAACACAAATTATTCGTTGAAGCCGAGCCGAAAAAGCCGCACCCGTTGGCGCAG

AGTATCGGCTCCGACGTCTTGTAACTCGTGTCATCGTCGTTTCCGTTAGAACTCATCTCCGATCTAAAAA

AATTTAAAATTTAAGATAATAATTCAATCGACGAATAAATTGTGTTTGTGTGGAATATATAGGTGTTGAA

TAAAGTGGATGAGTATAAACAGAAGTTGATTGATTTGTACGCGGTGTGGATTAGGACTCAATATGAGAGG

CGGTAGTAAAAAACGACGTGTATATCTCATGTACATGTAAACAAAATAATATTTTATATCGATAAACG

>MSTRG.453.1 gene=MSTRG.453

CTCTTCCACTCCTCTCTTTCGTTTCTTGATTTTATTTCAAATTTGAATCTCGCTGTGTGTATCTGTCTCT

ATCTCTCCTTCTTACTCTCATATGTTTCTCTCTCATATTTTAACATATAGTCATTGATGATTGATTGTTC

TTGCAGAAGCTTGATTCTAGGGTATTTTGAGGAGTATAGATTTATGATTTAGCTGTTTGACTGACGATTC

TCTCTGTTGTGTGCCCCTTTTTTCTAGGATTTCAAGCGCTGCTTCGCCGCTGGGAAATCAATTATCCTAT

TAAATCTATCTTTCTCGCCGGAGCTGGT

>MSTRG.454.1 gene=MSTRG.454

CATGGGTCACTCACGTGCACATTCACCCTCAAGTCTCCACTCTCTCACTCTCTCAGCGTGAGTCATTCTC

TCTCAAAAACACCATAGCCCTAATTTCCTTTCCATCTCATATCGCCGCTGATTAGTTCAAGTCGCTCATT

GTCCGCCGCTCCATCTCCGACTACAATGTTCGCTTCCGCCTCTGCTCGTAAATCGTGTAGACATTCATGT

TAATTGCTAGATCTAGTATAAGATATTCAAATCATTCAGTTTATCAAACCCTTGTACTATAGTGAAATGT

ACCCCTGTTTCTTTTACTTCACTTCTGATGCCGAATAAGTGGTCCAGATCTTAGATTTTTTTTGCAAGTC

TGTAACAATAAACACAACAAAGAGTCCTGATTTAAGGTGAGTTGATTTAGGCATTCGTTTCTTGACATCT

TCATATTACAAAAACATGAGTTTGTTTAGATTATAC

>MSTRG.456.1 gene=MSTRG.456

CTTCTGGATGGATATGCATGGGGGTTTCTTCTCTTGTAATCATGACTCCCCTTCATCATTACACTTCTTG

AATCTAACAATCTCCTCTCCCTCTCTCTCAGAGCCTACAGGCCACCATGTAATATACCACATTCACCCAT

AACTAACCATAACATTCAGCTATTTATACCAATCCCTCTACAAGTTCTAATTCTACTCAATCCTTGTAAT

ATATACTTCCTATTCGATAAAATTGAACGATGGGAAGCGCAAAACAGCTTTAAAAACTGGGAAGATGTGT

ATCGGAAGTACCTCCTCCGGATGGACTTCGTCGTCCTTCTCTTTGTACAAAAGAAAAGCAAGATCCAAAA

GATACAGGATTCAAGTTCATAGGCTTAACAGGAGAAAGAGAAGTAGAGATGATATGAAAGAGAAGAACTT

GAAGCTGTACATGGAGAACATTACTATCTTTCATGAGAATGAGAAGCTGAGAATGAAAGCTAGTCTTCTA

CACCAAGAAAACCTAAGCTTGATGTCTGAGTTTCAGAAGAAAAAACCCTCTCATCAGCACCAATGCGTAT

CCTCCACCCTAAATTCCCCAACTTACCACCAAGACTGAGTCCCTAGCTAGAATCGTTTCATTGCTTTGCA

TTATTATTATTGATCTACTCATCAAGCTGCTATTCAATAATTAATGTTAATCTTAATAAGAAGATGTACT

TTAATGGGTGGGAAGAGATCAAGGTGCAGAGCCGTAGTTCATTGTGGAAGTCGCAGTGGAGGAGTACTAC

CACATATTCTGTCGTGTACATCCTTTTAAGAGTATCAAATAATATTGAGAGCTCTAGAGCTCTACAAGAC

TCACTAGTCTTTGACAAAGGCATAACTCTAGTTATTTTTTCAGAATGTGTTGTTTAAAGTAACCACCTTC

CTTGTTTAAATAATTTCTCTTCTGTAATCCGTAGACACATCATACACTAATAAACTCACATCTCTTGATC

CCAATAGTCTTTCTAAGTTGTTTTGATGATTTAAGAGATTTAATACAAGTCCTTGCTCAAATATAAAAT

>MSTRG.456.2 gene=MSTRG.456

TAATTCTACTCAATCCTTGTAATATATACTTCCTATTCGATAAAATTGAACGATGGGAAGCGCAAAACAG

CTTTAAAAACTGGGAAGATGTGTATCGGAAGTACCTCCTCCGGATGGACTTCGTCGTCCTTCTCTTTGTA

CAAAAGAAAAGCAAGATCCAAAAGATACAGGATTCAAGTTCATAGGCTTAACAGGAGAAAGAGAAGTAGA

GATGATATGAAAGAGAAGAACTTGAAGCTGTACATGGAGAACATTACTATCTTTCATGAGAATGAGAAGC

TGAGAATGAAAGCTAGTCTTCTACACCAAGAAAACCTAAGCTTGATGTCTGAGTTTCAGAAGAAAAAACC

CTCTCATCAGCACCAATGCGTATCCTCCACCCTAAATTCCCCAACTTACCACCAAGACTGAGTCCCTAGC

TAGAATCGTTTCATTGCTTTGCATTATTATTATTGATCTACTCATCAAGCTGCTATTCAATAATTAATGT

TAATCTTAATAAGAAGATGTACTTTAATGGGTGGGAAGAGATCAAGGTGCAGAGCCGTAGTTCATTGTGG

AAGTCGCAGTGGAGGAGTACTACCACATATTCTGTCGTGTACATCCTTTTAAGAGTATCAAATAATATTG

AGAGCTCTAGAGCTCTACAAGACTCACTAGTCTTTGACAAAGGCATAACTCTAGTAACCACCTTCCTTGT

TTAAATAATTTCTCTTCTGTAATCCGTAGACACATCATACACTAATAAACTCACATCTCTTGATCCCAAT

AGTCTTTCTAAGTTGTTTTGATGATTTAAGAGATTTAATACAAGTCCTTGCTCAAATATAAAA

>MSTRG.479.1 gene=MSTRG.479

GTGAAGAGGGCTAAAGTTGAGTCCAGTGACAAGGAGAAATCTGACAATGGTATAGCTACTCGGAAAATCA

TTTTGGGCAGACCTATCTCGGGTAAGGCATTCTTCAATTGCGGTGTAATTAAGCTGTTCTCTGACTTAGG

GTTTGAATCTTTGATTGTTGATTTACCGAAAATCTGCTACTCCGTGTGTTGATTTGGGTTTGTCTGCTAA

GGAGATTATGGAATTCATGGATGAACTCCGTGATAACCATAAGCAGTTGGTTGAGGGTCAGAAGTTGTTG

TCTGAGCAAATGGATGATTTGGCCAACCAATTTCAGTTCTGGAAAGATATAGTTTTTGGAGGAAAGACAG

GGAATACACCAGAGAAGTGTAGCTCTGGTAGTTTTGTTCATGAGCTTCAGAAGCGGATGTATGGGTCTGG

TGGATCTTCTGATTTCAAGTTTACTTTTACTTCTACTGATGATGCCACTGACAGTCCTAGGCCACGTACT

GCAATGGATGCTCTTAAGGAGGCTGCCGGTACTGATTCTGCTTATGCTGATGCTGGAAATTTGATGATGG

AACAGAATCGTGTTGCTGCGGAGCTTACCAAGAAGTTTGCACAGGAGAAGCCTGACAAGGATGATGAGGA

GACCTAAATTTCTAACCTTTTCGATGATTAAGGGGGA

>MSTRG.487.1 gene=MSTRG.487

CTCGTAGATTCCCATCTTCACATCTTTCTCTTCCTTTTAAATACTCCAAACTAGTAGCTCCTCAATACAT

AAACACCTACAAGATTTTTATAATCACAATAGATACACGCATATATAATCAATTTCTCCCACGCAATCAA

ATACCCAAAAACCCTTCTTTCTTGTTATTTTTTAATCAAAAAATTCCATCTATTTACCAAACAACACAAG

AATTACTAGATTATCATCTTATAGTGATTTTTTGATCTGGGTTGTTGAAAAAGGTGCTAAATTTGGTCTT

TCTTCTGCTGGGTTTTAGTCTGTCT

>MSTRG.489.1 gene=MSTRG.489

TGCTTGATTCACATGACGGATGATGACTTAGAAAAGCTTCAGCATGACTCCCATTCTTGGACAGCAAGAA

GTCTTGCCAGTCTCCTCAATCTGCCCGACAAGAATCCTGCAGTACTGCTTCATTGATGATTTTGCTTCTG

ATTTCTTTTCAACACTTCAAAAACCTTGTTACTACTTTGATGCCAACTCGAGCAACAGATAATAATCAGC

CTGGGACGATGTAACAAATATTACAGTACGAGTGATCAGTTTGTGTAACAGTGGGAATCAAGTGACACGA

ACCGAG

>MSTRG.490.1 gene=MSTRG.490

TGCTTGATTCACATGACGGATGATGACTTAGAAAAGCTTCAGCATGACTCCCATTCTTGGACAGCAAGAA

GTCTTGCCAGTCTCCTCAATCTGCCCGACAAGAATCCTGCAGTACTGCTTCATTGATGATTTTGCTTCTG

ATTTCTTTTCAACACTTCAAAAACCTTGTTACTACTTTGATGCCAACTCGAGCAACAGATAATAATCAGC

CTGGGACGATGTAACAAATATTACAGTACGAGTGATCAGTTTGTGTAACAGTGGGAATCAAGTGACACGA

ACCGAGACCAAACAATCACCTACAATTATTAATAAAAGGATCAACATATAGTAAGCCATGGACAAGCCTG

CGGATGACATTCAACACAAATTTGAAGACTCAAGTCTAGTTGAAAGAGCACCGGATGCAATGACAAGCGA

AATGGAAAGCTTTAGTCTCGAAAAGCTCCACCATACCATATCTACAGGAATGGCCGAAATCGAGTTGTAA

AATTATGCATCAAGCTCCTAATTAAATGTGTGCCATCTCAGACAGCCTTTGTTGAATCAACTTATGAAGA

AAACTGAAGCCCAAACCGGATACTTCATCTAATGGTTCAAGCCAACAAGAAAGGAAGTGCTGATTTTTGA

GCCCCCGTTTCCAAAACAACACAGATAAAGAAATTAAAGTTCTGGGAACATCTACCAACTATTTAGAAAA

CAAGCCTCATTCAGACCCTCCGAGGTCGAGAAAAAAGGAAAAAGAAGAAGAAGGGAGAAGAATGACAATA

CTGCAGCAAACTGATGGATAACTTTTGGTAGAGAAACAACACCCGACAAAACTATCTCTCCAGGATCATC

AACGACTATTCAGGCCAGATACAATATTTGTCGAGCTGCTGATAGAGCTTCGAGCATAGCATTCTTGCTG

TTATAAGATAAGGAAAAAAGATTTCAACAAGTCGGAAGCTAGAGACGGAATTCAACAGCATCAACACAAC

TTCCCAGAAAATAAGATGTGGAGCAGAACTATTGGTTGTTAACTACAATCTATAGTGAAAGAATTGTGTT

TGATAAATACCAGAATGACACATGAGTTCCAAATTAATGTCACGTCGGGTGATCTCGTAAGACATGGCCC

CTGCAATTTTCTATGGGTTATCTAGAGTTATAATAAAAGCACCATCAGAGCACAAGTCTGAAGACAGGAT

CGTGCAAATCGGAGGGCTAAAATCTCGATAAAAGTATATCAGGCGACAAAGTCATCAAAAACAACAATGT

CATGAAAGTCACAAGGTAACAAAGTCAAAAAGTCACACAAGTCATAAGGCAAAAAAAAAGCGAACTACAT

CCCTGATAACAAAGTATACACATGACGAACTACACCCGTTCCTATCAAATACCATGGGACTTCAACGCCC

ATACACAAACACAACACATGACGGACTACACCCCGTTGCTATCAAATACCATGGGACTTCAACGCCCATA

CACAAACACAACACATGACGGACTACACCCCGTTGCTATCACATACCATGGGACTTCAACGCCCATACAC

AAACACAACACATGACGGACTACACCCCGTTGCTATCACATAACATGGGACTTCAACGCCCATACACAAA

CACAACACATGACGGACTACACCCCGTTGCTATCACATAACATGGGACTTCAACGCCCATACACAAACAC

AACACATGACGGACTACACCCCGTTGTTATCACATAACATGGGACTTCAACGCCCATACACAAACACAAC

ACATGACGGACTACACCCCGTTGCTATCACATAACATGGGACTTCAACGCCCATTCACAAACACAACACA

TGACGGACTACACCCCGTTGCTATCAAATA

>MSTRG.502.1 gene=MSTRG.502

GCAAGTGTCATGATTGTGCCTAGACCAAATATGACCTCTTGCAGATATTCTATCCATATAAAATTATGCA

AGTCAACAGATTTGCAATAACAAAAAAGATCAGAGCTTCTAGTTCATCCTCTGTGCAGCTTCCTTGGCAA

GGATCCTCTCTTTGGCCCTTGTTTTGGCTTGTGACTTTGAACCCATAATACCTCCTCCCCACTTCTTACG

GTACTCCTCATATTTGTCATTAAAATTGGCCTTAATAGCTTCCAATATTTTACTGAACTCCATCTTGTCC

TCATTTTTCACAGTGGTCAGGCACAAAACAGATGCAG

>MSTRG.509.1 gene=MSTRG.509

CGTACATAGATAGAGATGATTTTAAGTATCGGGAAAGACCCGAATAAAATAAATTGAGAATGGGTTGTGC

ATTCATCAATCTCAAATCACTGCTACTTGTCATATGCCTGCTGTTGCTTTCTTCCCATGATGTTCTTGCA

AAGTCTCGTCGCCCAATTTCTGAAACTGAGATCCGAGAGAAGAAGAATCGGTGCTTTGAAGATATTGACA

ATGGTTTATGGGGTCAACAGTGCACGTCTTCGATGATAGCCAAGGAAAATTGTATGTTGCAATGTGTGTC

CCCGCCTTGTTATGAACTTGTCTATGAAGGGGATCCGTTAGAAGAAGGGGAGAAAGATTATAGCAGGAGC

CAGGAGTACAAGTACTGCATGCACAGGTTATCTCTAGGGGAGAGCATAGATGGCATTCGAGGTTCCTTTG

ACATGTAAACATGGAAACACGAAACGGTAGCTATCATGGGACACTCAGATGCATCAGCAGTGAGAGTTGC

ATATTAAACTGAAAATGACTAGGCACTGTTCGCCATTATGATTAGTAGTTAATGTGCTTTGTATGAATAG

GCATCACGTCTTATTGGTAAAACTGCAAAAGAAGAAACCGAGTAGGGCATAATGCAAGAAACATTTTCAA

CTCTTTACAACTGACCAGAAGAGATTTGCATTGTATCTGTTTATTACCAACGGATTGACAAATATGTTGT

GCTGTAGTATCATGTGGTTGCAAGATTATTGTTGTATCTTTGTCATACAACTATACAAGTGTGTTAAATG

TTTAGTTTGGAGCCTATTTTATTTTTA

>MSTRG.511.1 gene=MSTRG.511

ATGAAATGATATAAATAGTAATGAAATAAAAGAAAAAGAATTTGATTCCAGAAACCCATGTTTCATATCC

ACCTCCCCCCTTGGGTATGAAAATCTCATACCTTGTGGGTTTGAGGAATGGGGTTGATAAAAGAAAATTT

TCAACCAAACATCAGGTATGAGTTTGGAATGAACAAAACCCATACCTGATACCAGAGAAGCCCCAACCAA

ACGACCCC

>MSTRG.516.1 gene=MSTRG.516

AATTACTCCCTCCGTCCCTTCCATTTCTTTACACTTTCCTTTTTGGGGTGTCCCATCCAATTCTTTACAT

TTCAAAACTTACCCAAAATAGTCAATGGGTCCCACCACTTCTTCACTTTACTTTCCTTTTCACACTACTA

TTACTCCACTATCTTCTTTTTATACATTAAAAATCAATGGGTCCCACCACTTCACCCACTTTTCTTTCTC

TTTTCCACTACTTTATACATATTTCTTAACATCCGTGCCCAACCCAAACGATAAGAATTGGGAGGGACGG

AGGGAGTA

>MSTRG.522.1 gene=MSTRG.522

GGTGCGTCTAGTGGTAGCTCAATTGTTGCATTGTTTTGATTGGGAACTTCCAAACGGAATGCAGCCATCA

GAACTTGACATGACTGAGGAATTCGGGCTCGTTGTTGCAAGGGCTACGCACCTTATGGCCATTCCGACCT

GTCGCCTTCACCAAAGCTAGGCGCCAATCAAGTAATACCGCTTGCTATAAACATTCCATCATGTACTTGC

TTTTATGTAGTAGTGCATTTAAGTGTCTTGTACACAGGTATCGTAACCCAGAAAAACCTCTATGTACTGA

GATGAATATGCATTGAG

>MSTRG.525.1 gene=MSTRG.525

AGGAAAAAAATATGCCAACATGATCATTGTTTTAGGAAGAGGCGATTCTCAACAGATTGTAGTAGAAATA

ATATATAAAATTTTGCTAATTATATCCACAAAGGTAAGACAACTCTACTTTTTCTTTTAATTACTTTTCT

TTTATAATCAACAATATGCATTTAATTTTGATAATTATGCACACAACCAAGAGGTATTGTATGAAGCTGG

ATATGTCCCTTCAAATTCTGAAAAGCGTCCTTTGGGGGTTATCATCTCTATTATTCAAATTGCTTTCCAT

GCAACTCCAGAGTTTACTTGCTCACAAGGTGCTGTAGAGGAACTTCAACTGTGCTTTTATGAGTATTTTA

AGATATGAATAGGGTGGCGGAAATGGGGAATATTGCTCTAGGCTTGGATTCTTAATAAGGAGAAAAAAAT

TGATTCTAAGGCTCCCCAGGGTTCTCGGCAACTGCCAAGACACTCACTGCTTTTGACCTCATTGTTATTG

GTACATTAATTTGTTTAGCTCCCTTATAACAGTATATTTTCTCGTTCAGACATATGGTTTTTTGTTTTAC

ACCTTGTCAAATATATTGTGTTTACTTGTTGCAGAATGCAGATACATCAGTAAAATGTATTTTATGGTTA

GTACTTTCTTGATGACTGAAAGGGGAGCCATCTTTTGTGCACCCCTTTGGTTTTGCCCAACGGTGTTATA

ATTATATTAGATTAATATAAAGTAATCACTATTGTATACGATATGGGAATAGCCATTGGTGCAAGTATCT

ACATTTCGGTTGGAACAATTACTAGAGGGCATGCAAAACCTATTTTTGATATCTGTTGTCTTATCGGTAG

AATGTCAGCTGATTTCTTAACGTGAGCTGGCTTATCACTGCCCTATGGTCGTTTATGGTCGTAAGTGCTT

ACAGGAACGAAGGCACAGGGGACATTGATGACGACTGTTCCCCAATTTTTTTGATAATCATGTATATTTT

CTCTTAAATTATGTATATCACATGTAAAAAAGAATTAATTTATGATCCTCA

>MSTRG.526.1 gene=MSTRG.526

CTTCAATCGTCTTCAAGGGAGTCAGCGGTTACATGAACAGGCAGGCGGCAGTTCCTCCATTTTAATTGGA

TGATGGAGTCGCCGGTGACAGGAAAGCGAAGCGAGTGTCTCCGGGGGGAGTGTTATGGATTCCGATGTGA

TTGAGAGGCCTCTGCCGACGCTGATTAATCGGAGTTCGAAATCAAAAACTCTTGTAGTTAAGGAGTGTAT

TTTTTGGAATCCGTAGAACCAATGGATGATTTTCATGCATGTTTCCAGTTTTGCTTTAAG

>MSTRG.528.1 gene=MSTRG.528

TTTTTTTTTATTTTATTTTGTTTTTATTTCTCACGTTTTTAGTTCACGTTTTTTTTTATTTTATTTTGTT

TTTATTTCTCACGTTTTTTTTCTCATATTTTTTTTTTATTTTATTTTTACATGTTTAGTAGTAATTGTTG

TAGCTAGTATCACGAGCATTTGCATGAATTATGAAGTTAAGCCAAGTTAATTGCCTATGATGAGTTATGT

GCGTAGTTCTTGATTAGTAATTTCAATTGCAATGCTAGGTTAGTACTTAGAAATTGCTTGTGTTGGAAAT

TGTAATTCACATATGTTCTTGAGATAGGATTTAGACGAAATTCCATGAATTCTAGGATTGAATTGAACAC

CCTTCAGCTTAGGTCTGTAAATCTTAAGTTTGGGGGAACTGAGGAGTAGATATACCTTTCTAAAAAAAAA

AATAGATAGAAAAAAAAAAAAAAGTAGTAAATAAATTCACTAAAAAAATAAGTGGTAGAATTAACTAGGT

TGAGCTCATTAGTACTCGAGTAATTAAGTCTGAGGGGACTTTGTGCCTAACAACCTAAAGCCCTTCGTGG

TTTGGGATTTGTTGACCCAACGCTCGCTACATGGGTACTAGTGCATAAATCTTTAGGGATCTCAACCATT

GCACGGTTAAATAAACCACTAGAATAGAGTGAATAATTGGTG

>MSTRG.532.1 gene=MSTRG.532

CCAGTTTCTCTGCACGAATACCGAGAGAAACCGAGCGGTGTTCTTCGTGTTCTTCATAATCAAACGTGGA

TTTGAAGGCGTTATTGGAGTCAGAATCAAGCGTGCGAATAGTCAAAACGAAGATATCGAGGAGTAGAATC

CATATCAATGATCAAAAACAACATGAGATTCCCAGAAAGGCTAAAAGATGAGATGATTGATTGAGATTGT

GGATAAGGCTAATGCTAGGCGAGGAGACAGAATTGAGGATCCAGTGGTCGAGGAGTGTTCAGGAAAGTTG

ATGAGGGTGGTGCAAGCTAGAGCTGTGCTATAGGGATTCAAGTGAAAGTCATAGCTGGAATAGTTAACAG

TGATTCTTCTTATCGAAGATGATCTGACTTGGTAAGAAAAGTTGTGTAATATTAGTGGTGGATTCTAATT

TCAATACTGTAACCTGGAAGCGATCCTGTTGTTTTTGGGGGTTAAATGTTCTCTTAAAATTTTCTTATT

>MSTRG.533.1 gene=MSTRG.533

TGCAGTTTAATGGACTGGAGTTTGATTGATTCCTTGACGTGGCCAATTTTTACAGCCGGATATATGATGA

TGATGGGGTATACTGAGGGGCCTAAATGGAAAGGGTTTTACGTGGATGATTCTCCTGATACATCTACGTG

GCTTTACTAAGTCGACTAAAGCAGGAGTATGAGCATGATCTAATCCAAAATGACCTACCAGATGTCTTGC

AAAGGCATATGGATGGCCAATATAGCTTTGGTTGCAGTTTGTCGAGGCTGGCCTGCATTTCCTTTCATCC

AAGTCGACATAAAAGCATGGATCTGGCTT

>MSTRG.534.1 gene=MSTRG.534

TGGATGATTCTCCTGATACATCTACGTGGCTTTACTAAGTCGACTAAAGCAGGAGTATGAGCATGATCTA

ATCCAAAATGACCTACCACATGTCTTGCAAAGGCATATGGATGGCCAATATAGTTTGTCGAGGCTGACCT

GCATTTCCTTTGATCCAAGTCGACATAAAAGCATGGATCTGGCTTGGAGTTTGCCTCTTGAGCAATGAAT

ATTGATAAATTCAATCCGGAGTGAGGAAAAGCTTTATTCACAGTCAAATGTAATCTGGAGCCAAATGTAT

TTTTGGCTTTCTCCTTCCTCCATGCAATATCAGAGTTGTTAATGCCAAAAAGGTTAGAGCTTACAAGCTC

CATTAAACAGATATGTTTTATGTCATGGAACCACATTGCTTATGCTATTATGCTCTCTCAGGTTTTACAT

TTTTACATTTTTACATATATAAAAAACAATACCATGCAATGTAATCAGATTCCCTACAGGATGTGAATAA

TGGGAGATTATTTGCATAATCATATTGAATTGTATTATTTTGTTAGCATGGTGACATTTAAAACTAAATG

ATGTATAGTTTATATATAAATACTGAGTTAATCGACTTACTAGAATTTTCGTGTAGGCACTAGAATGTGA

GTCTATATAGTTATTTTAAAACAGAATATACCAGTGTGTTGCCACATGTTTCACATTAAATCAAATGATG

CAGTTTCATATCTAATATGTGGAAAAAGAAGTTTGTGTAATTTAACAGTGTAGATGTAACTACATGACTC

TTTATCTGGTGTATGTGTTTCTATTTGGTGTGTATATAGTGGAATTCTGGCATATCTATGAATGCAATGA

AGCTGATGGGTTTCACATGCTGGTGGTTTTTGGTAGTTTAATTTAGTAGGTCCTTGAACAGAGTTTAGTG

GTTTAAATTAGCAATTGAAGGTAACACAATATCTATACATAAAAAAATTATTAATTTTTTTGGTCAGGTT

AAGTGATAGTTTTACTTAGAAAACCGACTCTACTTCTTGCGCCATTGGAAATGAGTTGGAAATTGCTAGC

ATTACCTCGGATTAGATATACTGATAAAGCAGACTCATCCCAGATTAATGGTTGTATCTCACTTTCTTCT

CAAATTCTTTGAC

>MSTRG.537.1 gene=MSTRG.537

GTGTGCGAATTAATTTGTAGGCGCTGTCGGTGTTACTCTCACAGCACAGCTTCTAGTAGTTAGCATCTCT

CTCTCTCTCTCTCTCTCGTGTAGACATACATTATATAGATATACACACTCTACCATCTCTCTCTCTCTCT

CTCTCTCTCTCTCTCTCTCTCTCTCTCTCTCTCTCTCTCTCTCTCTCTCTCTCTCTCTCTCTCTCTCTCT

CTCTCTCCGTATATACGCACAACAATATCTCTATATCATCCATATTGTTGTGAGTCTTGAGCTTCAACTA

TCTTTTCTTCGCTTCTTTCCTACGAAATGCATTGACAAGATCTCCCTCCCTCCCCTTTAGCTCTCTCTCT

CTCTCTGCGTTTATACTGTCCCAATTGTCAGCTACCTACTTCATTCAATCTGTTCTCCAGATCTTCCTAG

CTTATCTCAATCCTCACTTAACTCATTCATGTGAAGCTAGCTTCGCTTTGTAACTGTATTTTGGAGGTAG

ATCTTGAGATCTAATTAGCAAAGCTGGCGGCTACAGTAGCTTGGAGAGGGGTTGCTGCCAGCAATGGCAC

AGCCCTGTCATCTAATGATCTGGAAAGAAATGGGGATGCTAAATCTCATGACCAAGAGCCTCCAACACCA

CATTCACTCTTAAAGATGAGTTCGAGGGAGCGCAGTAGCATGGAGGATCCAGATGGAACTTTAGCTAGTG

TTGCTCAATGCATTGAGCAGTTGCGCCAGAGTTCCTCATCTGCACAAGAAAAAGAGTTCTCCTTAAAGCA

ACTATTGGAGCTTATTGATACACGTGAAAATGCTTTCAGTGCTGTTGGATCACATTCCCAGGCAGTTCCA

GTGTTGGTATCCCTTCTTAGATCAGGATCACTTGGGGTAAAAATACAGGCTGCTACAGTTTTAGGGTCAC

TGTGCAAGGAGAATGAACTAAGGGTGAAGGTATTGCTGGGGGGTTGCATTCCACCACTTCTTGGTCTACT

CAGGTCTAGCTCAGCGGAAGCTCAGATCGCTGCAGCGAAGACGATATATGCTGTTTCTCAAGGTGGTGCT

AAGGATCATGTTGGCTCAAAGATATTTTCAACTGAAGGAGTTGTGCCAGTTCTGTGGGGGCAGCTTGAGA

AGGGATTGAAGGCTGGAAACGTGGTTGATAACTTACTGACTGGAGCTTTGAAAAACCTTTCAAGCAGCAC

TGAGGGTTTCTGGTTTGCAACAATACAAGCTGGGGGATTAGATACACTTGTAAAGCTGCTTGCAACTGGA

CAATCTAACACCCAAGCAAATGTTTGCTTTCTCCTTGCATGCATGATGATGGAGGATGCATCTGTTTGTC

CTAAAATTCTGGCCGCTGAGACTACCAAGCAACTCCTAAAGCTACTAGGGCCCGGAAATGAAGCCTCGGT

CAGAGCAGAAGCTGCTGGTGCTCTAAAATCTCTATCTGCTCAGTGCAAAGAAGCAAGGCGGGAAATCGCA

AGTTCTAATGGTATACCCGCTTTGATAAATGCTACAATTGCTCCTTCAAAAGAGTTTATGCAAGGTGAGT

ATGCACAGGCTTTGCAGGAGAATGCAATGTGTGCTCTTGCAAATATATCTGGTGGTTTGTCATTTGTCAT

CTCAAGTCTTGGCCAAAGTCTTGAATCCTGCACGTCGCCTGCACAGGTAGCTGACACGTTAGGGGCATTA

GCTTCTGCACTAATGATTTATGACAGCAAAGCAGAAAGTACAAGAGCTTCAGATCCTGACGATGTTGAGA

AGACTCTTGTGAAGCAGTTCAAACCTCGCTTGCCATTTCTTGTGCAGGAGCGTACAATAGAAGCTCTTGC

CAGTTTGTATGGAAATTCCACACTTTCTGGTAAACTTGCAAATTCTGATGCAAAGCGTTTGCTAGTTGGT

TTGATCACAATGGCAAGCGATGAAGTTCAGGATGAGTTGATACGATCACTTCTGGTATTGTGTAACAATG

AAGGTACACTATGGCATGCCCTTCAGGGACGTGAGGGAATTCAATTATTGATCTCTCTTCTTGGGCTGTC

ATCTGAGCAACAACAGGAGTGTGCCGTCGCATTACTTTCCCTTTTATCCGATGAGAATGATGAAAGCAAA

TGGGCCATCACAGCTGCTGGAGGTATACCTCCACTTGTTCAGATATTAGAGACAGGATCTCCAAAAGCCA

AGGAAGATTCTGCAACAATCCTCGGAAACCTCTGTAATCACAGTGAAGATATTAGAGCATGTGTTGAAAG

TGCTGATGCTGTTCCTGCTTTGCTGTGGCTATTAAAGAATGGAAGCTCCAATGGCAAAGAAATTGCTGCA

AAGACATTGAATCATCTGATCCACAAATCAGATACAGCAACCATTAGTCAGCTCACCGCATTACTTACAA

GCGATCTACCCGAATCTAAAGTGTATGTTTTAGATGCACTAAAAAGTTTGCTATCTGTGGCCCCTCTTAG

TGATATGTTGCGTGATGGAAGTGCTTCAAACGATGCTATCGAGACAATGATTAAAATATTAAGTTCTACC

AGAGATGAGACACAGTCTAAGTCTGCATCAGCTCTTGCTGGAATATTTAATCTTAGAAAGGACTTGCGTG

AAAGTCCCATAGCTGTTAAGACTGTTAGGTCGGTCATGAAGCTTTTACATGTAGAATCCGAAAGCATATT

GGCGGAGTCTACTCGTTGCCTTGCTGCAATATTTCTGTCAATTAAGGAGAACCGTGACATGGCTATGATT

GGGAGAGATTCATTGCCTACACTAGAGGTGCTTGCAACTTCGTCTTCACTGCAAGTTGCAGAGCAGGCCA

TTTGTGCTTTGGCTAACCTTCTTTTGGATAGTGAAGTTTCAGAAAAAGCCAAGCCTGAAGAAATTATTTT

GCCTTCTACTAGGGTATTGCGTGAAAGCACAGTTACTGGAAGGAGCCATGCAGCAGCAGCAATTGCCCGG

CTTCTGAGTTCTCGTAAAATTGATGCTGAGATTGCTGACTGTGTGAACCGTACAGGAACAGTGCTTGCAT

TGGTTTCGTTCCTAGAATCAGAAAATGCCGGGACTGCTGCCATATCAGAGGGGTTAGAGGCACTTGCCAT

TATATCGAGATCTGGAGGAGATAAAGGACAGAACAAACCTGCATGGGCAGTTCTGGCTGAATTCCCAGAT

AGCATAATCCCGATCGTTTCATGCATTTCTGATGCAAACCCCTTGTTGCTTGATAAGGCTATAGAAATAT

TGTCACGGATTTCTTTGGCTCAACCTGTTGTTTTGGGGAACAATATTGTGAGTGCTTCAGGGTGTATCTC

GTCAATTGCTAGACGGGTAGTTAGCTCTTCAAAGGAAGCAGTAAAGATTGGAGGCACCACCCTTCTTGTT

TGTGCTGCCAAAGTAAATCTTCAGAGAGTTGTAGATGATTTGAATGAATCATATTCTTGCGCATATCTCA

TTCAGTCCCTTGTAGGAATGCTGACCTACACAAAAAATCCTCTGGTTGGTGACCAGGGAAGTAATGAAGG

CGTAAATATTCTTGGGCATGCTGAAGAACTAAAAGCTGGTGAGAATGAGACGAGTAGATATGTCATCTAT

GGTTCAAATACCGCAATATGGCTTCTCTCTGCTCTTGCTAGTAATAACGAAAAGAGCAAAACTCAGATCA

TGGAAGCAGGTGCCATTGAAGTTCTAACTGAGAGAATCAATCAGTGTTTATCAGAATATTCTCAGGTCGA

TTCTAAAGAGGAAAGCAGCATTTGGATATGTGCTTTACTTCTAGCAATTCTATTTCAAGATAGAGATATT

ATACGTGCGCATGCAACAATGAAATCTGTACCTGTACTGGCTAATTTGTTGAAGTCAGAGGAGGCAGCAA

ACAGATATTTTGGTGCACAAGCCATTGCCAGTCTAGTATGCAATGGTAGCAGGGGGACTCTTCTATCAGT

CGCTAATTCAGGGGCTGCAGGTGGACTCATTTCACTGCTTGGGTGTGCTGATGCTGATATATGTGATATG

CTTGACTTGGCAGACGAATTTTCCTTGGTGCGGTATCCTGAACAAGTTGCTCTTGAGAGGTTGTTTAGGG

TTGAGGATATCAGAGTTGGTGCTACTTCAAGAAAAGCAATACCTGCACTAGTTGATCTACTTAAACCGAT

TCCAGATCGTCCAGGGGCACCATTTTTAACCTTAGGGCTTCTGCTTCAGCTTGGTAAAGATTGCCCTTCG

AATAAAATTCTTATGGTAGAAGCTGGTGCTCTGGAAGCATTGACCAAATACCTTTCACTTGGCTTACAAG

ATGCAACTGAAGAAGCAGCTACGGATCTCTTAGGTATGTTATTTAGTACTGCTGAGATACGTAGACATGA

AGCAGCATTTGGTGCCGTAAGCCAACTTGTAGCAGTATTGCGATTGGGTGGAAGAGGTGCAAGGTATAGT

GCTGCTTTAGCATTGGAAAGCCTTTTTTCTGCAGACCATATTAGGAATGCAGAATCTTCCAGGCAGGCTG

TACAGCCTTTAGTGGAAATCCTTAATACAGGTTTGGAGAAAGAGCAGCATGCTGCTATTGCTGCATTAGC

TAGGTTACTGAGTGAAAATCCATCAAGAGCCTTGGCAGTTGCTGATGTTGAAATGAATGCGGTAGATGTT

CTTTGCAGGATTCTTTCATCAAACTGTTCACTAGAGCTGAAGGGGGATTCGGCTGAGTTGTGCTGTGTAC

TTTTTGGAAATACAAAGATCAGATCCACCCTGGCTGCAGCGCGCTGTGTGGAACCTCTGGTTTCTTTGCT

CGTGGCTGAGTATAGTCCTACTCAGCACTCTGTTGTCCGTGCATTAGATAAACTCGTTGATGACGATCAA

TTAGCTGAGCTCATTGCTGCACATGGAGCAGTTATACCTCTTGTAGGCCTTCTGTATGGCCACAACTATG

TGCTCCATGAGGCTATTTCCAGAGCTCTTGTGAAGTTGGGAAAAGACAGGCCTTCTTGTAAGATGGAGAT

GGTGAAGGCTGGGGTGATTGAGAGTGTACTTGATATTCTCCATGATGCACCAGATTTTCTGTGTGCTGCT

TTTGCAGAATTGCTACGAATATTAACCAATAATGCTACCATTGCCAAGGGTCCATCTGCAGCGAAAGTGG

TTGAGCCTTTCTTTGTGTTGTTAACAAGAGCAGAGTTCGGACCTGATGGACAGCATAGTGCTTTACAAGT

TCTTGTGAATATCTTAGAACATCCTCAGTGCCGTGCTGATTATACCTTAACATCACATCAAGTGATCGAA

CCCATCATTCCATTACTTGATTCTCCTGCACCGCCAGTGCAGCAGCTAGCAGCTGAACTTCTTTCTCATT

TACTCCTAGAGGAACATCTTCAGAAGGATTCAGTTACACAGCAAGTAATTGGCCCACTTATGCGAGTTCT

TGGTTCTGGTATACACATTCTGCAGCAGAGAGCTGTTAAAGCTCTTGTTAATATTGCACTGATTTGGCCC

AATGAAATTGCCAAGGAGGGTGGCGTCAGTGAGCTATCCAAAGTGATATTGCTAGCTGATCCCTCTTTGC

CTCATGTCTTGTGGGAATCAGCTGCTTCTGTTTTGTCCAGTATTCTTCAGTTCAGTTCTGAGTACTATTT

GGAAGTGCCTATTGCGGTTTTGGTGAGATTGCTTCGATCTGGTTCAGAATCTACAGTAGTTGGTGCATTA

AATGCTCTTCTAGTTCTGGAAAGTGATGATGCTACAAGTGCTGTAGCAATGGCTGAAAGTGGAGCCATTG

AAGCTCTTTTAGAACTTCTTAGATGTCATCAGTGTGAGGAAACTGCTGCAAGACTTCTGGAGGTATTGCT

GAACAATATTAAGATCAGAGAAACAAAAGCCACAAAGTCTGCAATAGTACCACTATCACAGTATCTCTTG

GATCCACAAAGCCAAGCTCAACAAGCAAGATTACTAGCAACTCTTGCTCTAGGGGATTTATTTCAGAATG

AGGTTCTTGCTCGATCTGCTGATGCTGTTTTAGCTTGCCGTGCTTTAGTAAATCTGCTTGAGGATCAACC

TTCAGAAGAAATGAAAGTGGTTGCAATATGTGCATTGCAAAACCTTGTAATGTACAGTAGATCAAATAAG

AGAGCAGTTGCTGAAGCTGGAGGTGTTCAGGTCGTGCTGGATCTGATTGGTTCAAGTGAACCAGATACAT

CAGTTCAGGCTGCAATGTTTGTTAAGCTTCTCTTCTCTAACAACACAATTCAAGAGTATGCTTCCAGCGA

AACCGTTAGAGCTATAACAGCTGCAATTGAAAAAGATTTATGGGCTAATGGTGTAGTGAATGAAGAGTAT

CTTAAAGCTCTTAATGCACTCCTTGGCAACTTCCCACGTTTAAGAGCCACAGAACCTGCAACCCTGAGCA

TTCCCCATCTAGTGACAGCCCTCAAGACTGGCTCAGAGACAACTCAAGAAGCAGCATTGGATTCACTGTC

TCTTCTCAAGCAAGCTTGGGCAGCATGCCCTGCCGAGGTCTCCAGAGCGCAATCAACAGCTGCTTCTGAG

GGGATTCCCCTGTTACAGTACTTAATTATGTCTGCCCCACCTCGAGTTCAAGATAAGGCAGACCATCTTT

TGCAGTGTCTGCCGGGAACATTGACAGTGACTATAAAACGTGGAAAGAATATCAAACAGTCAGTTGGAAA

TCCAAGTGTGTTTTGCAAGCTAACACTTGGCAACACCCCCTCCAGGGAAACCAAGATTGTGTCGACCGGA

CCAAATCCCGAGTGGGATGAGCCCTTCCAGTGGCAATTTGAAAGTCCTCCAAAAGGCCAAAAGCTTCACA

TTTCTTGCAAGAACAAGAGCAAGATGGGAAAGAAATCGTTTGGAAAAGTCACTGTCCAGATTGATCGAGT

CGTTACCCAAGGAGCAGCTGCAGGAGAGTACTTCCTGCTACCTGAAAGCAAGAGTGGTAGTAAAAGGAGC

CTGGAAATAGAATTTCAATGGACTAATAGCAATAACATGCCTCAGTCTGAAGCTTAAAGAGATTTGCCTG

GTGTTAATTTTTTCTTTTTATCGAATATAATTATTGTGCTGGTGTACATAATATCTGGAAGGTTTATTTT

TGTAGTTAGTTGCTGCAATCCGGAAAGTCGGAAACTGCAATTCATTGTATTCTTTTGCAAAGTGTGGTAG

TTTTAGTCATCTTCTGTTCTTTTTGTTATAGTTGTATTGGAGTTGTATTCTGTGTTTGATATTCAGACTC

GAGAGGTTAATAAAATCTTGTGTATTGAATTCATTCTTACCTGTTTT

>MSTRG.537.2 gene=MSTRG.537

GTGTGCGAATTAATTTGTAGGCGCTGTCGGTGTTACTCTCACAGCACAGCTTCTAGTAGTTAGCATCTCT

CTCTCTCTCTCTCTCTCGTGTAGACATACATTATATAGATATACACACTCTACCATCTCTCTCTCTCTCT

CTCTCTCTCTCTGCACAACAATATCTCTATATCATCCATATTGTTGTGAGTCTTGAGCTTCAACTATCTT

TTCTTCGCTTCTTTCCTACGAAATGCATTGACAAGATCTCCCTCCCTCCCCTTTAGCTCTCTCTCTCTCT

CTGCGTTTATACTGTCCCAATTGTCAGCTACCTACTTCATTCAATCTGTTCTCCAGATCTTCCTAGCTTA

TCTCAATCCTCACTTAACTCATTCATGTGAAGCTAGCTTCGCTTTGTAACTGTATTTTGGAGGTAGATCT

TGAGATCTAATTAGCAAAGCTGGCGGCTACAGTAGCTTGGAGAGGGGTTGCTGCCAGCAATGGCACAGCC

CTGTCATCTAATGATCTGGAAAGAAATGGGGATGCTAAATCTCATGACCAAGAGCCTCCAACACCACATT

CACTCTTAAAGATGAGTTCGAGGGAGCGCAGTAGCATGGAGGATCCAGATGGAACTTTAGCTAGTGTTGC

TCAATGCATTGAGCAGTTGCGCCAGAGTTCCTCATCTGCACAAGAAAAAGAGTTCTCCTTAAAGCAACTA

TTGGAGCTTATTGATACACGTGAAAATGCTTTCAGTGCTGTTGGATCACATTCCCAGGCAGTTCCAGTGT

TGGTATCCCTTCTTAGATCAGGATCACTTGGGGTAAAAATACAGGCTGCTACAGTTTTAGGGTCACTGTG

CAAGGAGAATGAACTAAGGGTGAAGGTATTGCTGGGGGGTTGCATTCCACCACTTCTTGGTCTACTCAGG

TCTAGCTCAGCGGAAGCTCAGATCGCTGCAGCGAAGACGATATATGCTGTTTCTCAAGGTGGTGCTAAGG

ATCATGTTGGCTCAAAGATATTTTCAACTGAAGGAGTTGTGCCAGTTCTGTGGGGGCAGCTTGAGAAGGG

ATTGAAGGCTGGAAACGTGGTTGATAACTTACTGACTGGAGCTTTGAAAAACCTTTCAAGCAGCACTGAG

GGTTTCTGGTTTGCAACAATACAAGCTGGGGGATTAGATACACTTGTAAAGCTGCTTGCAACTGGACAAT

CTAACACCCAAGCAAATGTTTGCTTTCTCCTTGCATGCATGATGATGGAGGATGCATCTGTTTGTCCTAA

AATTCTGGCCGCTGAGACTACCAAGCAACTCCTAAAGCTACTAGGGCCCGGAAATGAAGCCTCGGTCAGA

GCAGAAGCTGCTGGTGCTCTAAAATCTCTATCTGCTCAGTGCAAAGAAGCAAGGCGGGAAATCGCAAGTT

CTAATGGTATACCCGCTTTGATAAATGCTACAATTGCTCCTTCAAAAGAGTTTATGCAAGGTGAGTATGC

ACAGGCTTTGCAGGAGAATGCAATGTGTGCTCTTGCAAATATATCTGGTGGTTTGTCATTTGTCATCTCA

AGTCTTGGCCAAAGTCTTGAATCCTGCACGTCGCCTGCACAGGTAGCTGACACGTTAGGGGCATTAGCTT

CTGCACTAATGATTTATGACAGCAAAGCAGAAAGTACAAGAGCTTCAGATCCTGACGATGTTGAGAAGAC

TCTTGTGAAGCAGTTCAAACCTCGCTTGCCATTTCTTGTGCAGGAGCGTACAATAGAAGCTCTTGCCAGT

TTGTATGGAAATTCCACACTTTCTGGTAAACTTGCAAATTCTGATGCAAAGCGTTTGCTAGTTGGTTTGA

TCACAATGGCAAGCGATGAAGTTCAGGATGAGTTGATACGATCACTTCTGGTATTGTGTAACAATGAAGG

TACACTATGGCATGCCCTTCAGGGACGTGAGGGAATTCAATTATTGATCTCTCTTCTTGGGCTGTCATCT

GAGCAACAACAGGAGTGTGCCGTCGCATTACTTTCCCTTTTATCCGATGAGAATGATGAAAGCAAATGGG

CCATCACAGCTGCTGGAGGTATACCTCCACTTGTTCAGATATTAGAGACAGGATCTCCAAAAGCCAAGGA

AGATTCTGCAACAATCCTCGGAAACCTCTGTAATCACAGTGAAGATATTAGAGCATGTGTTGAAAGTGCT

GATGCTGTTCCTGCTTTGCTGTGGCTATTAAAGAATGGAAGCTCCAATGGCAAAGAAATTGCTGCAAAGA

CATTGAATCATCTGATCCACAAATCAGATACAGCAACCATTAGTCAGCTCACCGCATTACTTACAAGCGA

TCTACCCGAATCTAAAGTGTATGTTTTAGATGCACTAAAAAGTTTGCTATCTGTGGCCCCTCTTAGTGAT

ATGTTGCGTGATGGAAGTGCTTCAAACGATGCTATCGAGACAATGATTAAAATATTAAGTTCTACCAGAG

ATGAGACACAGTCTAAGTCTGCATCAGCTCTTGCTGGAATATTTAATCTTAGAAAGGACTTGCGTGAAAG

TCCCATAGCTGTTAAGACTGTTAGGTCGGTCATGAAGCTTTTACATGTAGAATCCGAAAGCATATTGGCG

GAGTCTACTCGTTGCCTTGCTGCAATATTTCTGTCAATTAAGGAGAACCGTGACATGGCTATGATTGGGA

GAGATTCATTGCCTACACTAGAGGTGCTTGCAACTTCGTCTTCACTGCAAGTTGCAGAGCAGGCCATTTG

TGCTTTGGCTAACCTTCTTTTGGATAGTGAAGTTTCAGAAAAAGCCAAGCCTGAAGAAATTATTTTGCCT

TCTACTAGGGTATTGCGTGAAAGCACAGTTACTGGAAGGAGCCATGCAGCAGCAGCAATTGCCCGGCTTC

TGAGTTCTCGTAAAATTGATGCTGAGATTGCTGACTGTGTGAACCGTACAGGAACAGTGCTTGCATTGGT

TTCGTTCCTAGAATCAGAAAATGCCGGGACTGCTGCCATATCAGAGGGGTTAGAGGCACTTGCCATTATA

TCGAGATCTGGAGGAGATAAAGGACAGAACAAACCTGCATGGGCAGTTCTGGCTGAATTCCCAGATAGCA

TAATCCCGATCGTTTCATGCATTTCTGATGCAAACCCCTTGTTGCTTGATAAGGCTATAGAAATATTGTC

ACGGATTTCTTTGGCTCAACCTGTTGTTTTGGGGAACAATATTGTGAGTGCTTCAGGGTGTATCTCGTCA

ATTGCTAGACGGGTAGTTAGCTCTTCAAAGGAAGCAGTAAAGATTGGAGGCACCACCCTTCTTGTTTGTG

CTGCCAAAGTAAATCTTCAGAGAGTTGTAGATGATTTGAATGAATCATATTCTTGCGCATATCTCATTCA

GTCCCTTGTAGGAATGCTGACCTACACAAAAAATCCTCTGGTTGGTGACCAGGGAAGTAATGAAGGCGTA

AATATTCTTGGGCATGCTGAAGAACTAAAAGCTGGTGAGAATGAGACGAGTAGATATGTCATCTATGGTT

CAAATACCGCAATATGGCTTCTCTCTGCTCTTGCTAGTAATAACGAAAAGAGCAAAACTCAGATCATGGA

AGCAGGTGCCATTGAAGTTCTAACTGAGAGAATCAATCAGTGTTTATCAGAATATTCTCAGGTCGATTCT

AAAGAGGAAAGCAGCATTTGGATATGTGCTTTACTTCTAGCAATTCTATTTCAAGATAGAGATATTATAC

GTGCGCATGCAACAATGAAATCTGTACCTGTACTGGCTAATTTGTTGAAGTCAGAGGAGGCAGCAAACAG

ATATTTTGGTGCACAAGCCATTGCCAGTCTAGTATGCAATGGTAGCAGGGGGACTCTTCTATCAGTCGCT

AATTCAGGGGCTGCAGGTGGACTCATTTCACTGCTTGGGTGTGCTGATGCTGATATATGTGATATGCTTG

ACTTGGCAGACGAATTTTCCTTGGTGCGGTATCCTGAACAAGTTGCTCTTGAGAGGTTGTTTAGGGTTGA

GGATATCAGAGTTGGTGCTACTTCAAGAAAAGCAATACCTGCACTAGTTGATCTACTTAAACCGATTCCA

GATCGTCCAGGGGCACCATTTTTAACCTTAGGGCTTCTGCTTCAGCTTGGTAAAGATTGCCCTTCGAATA

AAATTCTTATGGTAGAAGCTGGTGCTCTGGAAGCATTGACCAAATACCTTTCACTTGGCTTACAAGATGC

AACTGAAGAAGCAGCTACGGATCTCTTAGGTATGTTATTTAGTACTGCTGAGATACGTAGACATGAAGCA

GCATTTGGTGCCGTAAGCCAACTTGTAGCAGTATTGCGATTGGGTGGAAGAGGTGCAAGGTATAGTGCTG

CTTTAGCATTGGAAAGCCTTTTTTCTGCAGACCATATTAGGAATGCAGAATCTTCCAGGCAGGCTGTACA

GCCTTTAGTGGAAATCCTTAATACAGGTTTGGAGAAAGAGCAGCATGCTGCTATTGCTGCATTAGCTAGG

TTACTGAGTGAAAATCCATCAAGAGCCTTGGCAGTTGCTGATGTTGAAATGAATGCGGTAGATGTTCTTT

GCAGGATTCTTTCATCAAACTGTTCACTAGAGCTGAAGGGGGATTCGGCTGAGTTGTGCTGTGTACTTTT

TGGAAATACAAAGATCAGATCCACCCTGGCTGCAGCGCGCTGTGTGGAACCTCTGGTTTCTTTGCTCGTG

GCTGAGTATAGTCCTACTCAGCACTCTGTTGTCCGTGCATTAGATAAACTCGTTGATGACGATCAATTAG

CTGAGCTCATTGCTGCACATGGAGCAGTTATACCTCTTGTAGGCCTTCTGTATGGCCACAACTATGTGCT

CCATGAGGCTATTTCCAGAGCTCTTGTGAAGTTGGGAAAAGACAGGCCTTCTTGTAAGATGGAGATGGTG

AAGGCTGGGGTGATTGAGAGTGTACTTGATATTCTCCATGATGCACCAGATTTTCTGTGTGCTGCTTTTG

CAGAATTGCTACGAATATTAACCAATAATGCTACCATTGCCAAGGGTCCATCTGCAGCGAAAGTGGTTGA

GCCTTTCTTTGTGTTGTTAACAAGAGCAGAGTTCGGACCTGATGGACAGCATAGTGCTTTACAAGTTCTT

GTGAATATCTTAGAACATCCTCAGTGCCGTGCTGATTATACCTTAACATCACATCAAGTGATCGAACCCA

TCATTCCATTACTTGATTCTCCTGCACCGCCAGTGCAGCAGCTAGCAGCTGAACTTCTTTCTCATTTACT

CCTAGAGGAACATCTTCAGAAGGATTCAGTTACACAGCAAGTAATTGGCCCACTTATGCGAGTTCTTGGT

TCTGGTATACACATTCTGCAGCAGAGAGCTGTTAAAGCTCTTGTTAATATTGCACTGATTTGGCCCAATG

AAATTGCCAAGGAGGGTGGCGTCAGTGAGCTATCCAAAGTGATATTGCTAGCTGATCCCTCTTTGCCTCA

TGTCTTGTGGGAATCAGCTGCTTCTGTTTTGTCCAGTATTCTTCAGTTCAGTTCTGAGTACTATTTGGAA

GTGCCTATTGCGGTTTTGGTGAGATTGCTTCGATCTGGTTCAGAATCTACAGTAGTTGGTGCATTAAATG

CTCTTCTAGTTCTGGAAAGTGATGATGCTACAAGTGCTGTAGCAATGGCTGAAAGTGGAGCCATTGAAGC

TCTTTTAGAACTTCTTAGATGTCATCAGTGTGAGGAAACTGCTGCAAGACTTCTGGAGGTATTGCTGAAC

AATATTAAGATCAGAGAAACAAAAGCCACAAAGTCTGCAATAGTACCACTATCACAGTATCTCTTGGATC

CACAAAGCCAAGCTCAACAAGCAAGATTACTAGCAACTCTTGCTCTAGGGGATTTATTTCAGAATGAGGT

TCTTGCTCGATCTGCTGATGCTGTTTTAGCTTGCCGTGCTTTAGTAAATCTGCTTGAGGATCAACCTTCA

GAAGAAATGAAAGTGGTTGCAATATGTGCATTGCAAAACCTTGTAATGTACAGTAGATCAAATAAGAGAG

CAGTTGCTGAAGCTGGAGGTGTTCAGGTCGTGCTGGATCTGATTGGTTCAAGTGAACCAGATACATCAGT

TCAGGCTGCAATGTTTGTTAAGCTTCTCTTCTCTAACAACACAATTCAAGAGTATGCTTCCAGCGAAACC

GTTAGAGCTATAACAGCTGCAATTGAAAAAGATTTATGGGCTAATGGTGTAGTGAATGAAGAGTATCTTA

AAGCTCTTAATGCACTCCTTGGCAACTTCCCACGTTTAAGAGCCACAGAACCTGCAACCCTGAGCATTCC

CCATCTAGTGACAGCCCTCAAGACTGGCTCAGAGACAACTCAAGAAGCAGCATTGGATTCACTGTCTCTT

CTCAAGCAAGCTTGGGCAGCATGCCCTGCCGAGGTCTCCAGAGCGCAATCAACAGCTGCTTCTGAGGGGA

TTCCCCTGTTACAGTACTTAATTATGTCTGCCCCACCTCGAGTTCAAGATAAGGCAGACCATCTTTTGCA

GTGTCTGCCGGGAACATTGACAGTGACTATAAAACGTGGAAAGAATATCAAACAGTCAGTTGGAAATCCA

AGTGTGTTTTGCAAGCTAACACTTGGCAACACCCCCTCCAGGGAAACCAAGATTGTGTCGACCGGACCAA

ATCCCGAGTGGGATGAGCCCTTCCAGTGGCAATTTGAAAGTCCTCCAAAAGGCCAAAAGCTTCACATTTC

TTGCAAGAACAAGAGCAAGATGGGAAAGAAATCGTTTGGAAAAGTCACTGTCCAGATTGATCGAGTCGTT

ACCCAAGGAGCAGCTGCAGGAGAGTACTTCCTGCTACCTGAAAGCAAGAGTGGTAGTAAAAGGAGCCTGG

AAATAGAATTTCAATGGACTAATAGCAATAACATGCCTCAGTCTGAAGCTTAAAGAGATTTGCCTGGTGT

TAATTTTTTCTTTTTATCGAATATAATTATTGTGCTGGTGTACATAATATCTGGAAGGTTTATTTTTGTA

GTTAGTTGCTGCAATCCGGAAAGTCGGAAACTGCAATTCATTGTATTCTTTTGCAAAGTGTGGTAGTTTT

AGTCATCTTCTGTTCTTTTTGTTATAGTTGTATTGGAGTTGTATTCTGTGTTTGATATTCAGACTCGAGA

GGTTAATAAAATCTTGTGTATTGAATTCATTCTTACCTGTTTT

>MSTRG.537.3 gene=MSTRG.537

CGGTGTTACTCTCACAGCACAGCTTCTAGTAGTTAGCATCTCTCTCTCTCTCTCTCTCTCTCTCTCTCTC

TCTCTCTCTCTCTCTCGTGTAGACATACATTATATAGATATACACACTCTACCATCTCTCTCTCTCTCTC

TCTCTCTCTCTGCACAACAATATCTCTATATCATCCATATTGTTGTGAGTCTTGAGCTTCAACTATCTTT

TCTTCGCTTCTTTCCTACGAAATGCATTGACAAGATCTCCCTCCCTCCCCTTTAGCTCTCTCTCTCTCTC

TGCGTTTATACTGTCCCAATTGTCAGCTACCTACTTCATTCAATCTGTTCTCCAGATCTTCCTAGCTTAT

CTCAATCCTCACTTAACTCATTCATGTGAAGCTAGCTTCGCTTTGTAACTGTATTTTGGAGGTAGATCTT

GAGATCTAATTAGCAAAGCTGGCGGCTACAGTAGCTTGGAGAGGGGTTGCTGCCAGCAATGGCACAGCCC

TGTCATCTAATGATCTGGAAAGAAATGGGGATGCTAAATCTCATGACCAAGAGCCTCCAACACCACATTC

ACTCTTAAAGATGAGTTCGAGGGAGCGCAGTAGCATGGAGGATCCAGATGGAACTTTAGCTAGTGTTGCT

CAATGCATTGAGCAGTTGCGCCAGAGTTCCTCATCTGCACAAGAAAAAGAGTTCTCCTTAAAGCAACTAT

TGGAGCTTATTGATACACGTGAAAATGCTTTCAGTGCTGTTGGATCACATTCCCAGGCAGTTCCAGTGTT

GGTATCCCTTCTTAGATCAGGATCACTTGGGGTAAAAATACAGGCTGCTACAGTTTTAGGGTCACTGTGC

AAGGAGAATGAACTAAGGGTGAAGGTATTGCTGGGGGGTTGCATTCCACCACTTCTTGGTCTACTCAGGT

CTAGCTCAGCGGAAGCTCAGATCGCTGCAGCGAAGACGATATATGCTGTTTCTCAAGGTGGTGCTAAGGA

TCATGTTGGCTCAAAGATATTTTCAACTGAAGGAGTTGTGCCAGTTCTGTGGGGGCAGCTTGAGAAGGGA

TTGAAGGCTGGAAACGTGGTTGATAACTTACTGACTGGAGCTTTGAAAAACCTTTCAAGCAGCACTGAGG

GTTTCTGGTTTGCAACAATACAAGCTGGGGGATTAGATACACTTGTAAAGCTGCTTGCAACTGGACAATC

TAACACCCAAGCAAATGTTTGCTTTCTCCTTGCATGCATGATGATGGAGGATGCATCTGTTTGTCCTAAA

ATTCTGGCCGCTGAGACTACCAAGCAACTCCTAAAGCTACTAGGGCCCGGAAATGAAGCCTCGGTCAGAG

CAGAAGCTGCTGGTGCTCTAAAATCTCTATCTGCTCAGTGCAAAGAAGCAAGGCGGGAAATCGCAAGTTC

TAATGGTATACCCGCTTTGATAAATGCTACAATTGCTCCTTCAAAAGAGTTTATGCAAGGTGAGTATGCA

CAGGCTTTGCAGGAGAATGCAATGTGTGCTCTTGCAAATATATCTGGTGGTTTGTCATTTGTCATCTCAA

GTCTTGGCCAAAGTCTTGAATCCTGCACGTCGCCTGCACAGGTAGCTGACACGTTAGGGGCATTAGCTTC

TGCACTAATGATTTATGACAGCAAAGCAGAAAGTACAAGAGCTTCAGATCCTGACGATGTTGAGAAGACT

CTTGTGAAGCAGTTCAAACCTCGCTTGCCATTTCTTGTGCAGGAGCGTACAATAGAAGCTCTTGCCAGTT

TGTATGGAAATTCCACACTTTCTGGTAAACTTGCAAATTCTGATGCAAAGCGTTTGCTAGTTGGTTTGAT

CACAATGGCAAGCGATGAAGTTCAGGATGAGTTGATACGATCACTTCTGGTATTGTGTAACAATGAAGGT

ACACTATGGCATGCCCTTCAGGGACGTGAGGGAATTCAATTATTGATCTCTCTTCTTGGGCTGTCATCTG

AGCAACAACAGGAGTGTGCCGTCGCATTACTTTCCCTTTTATCCGATGAGAATGATGAAAGCAAATGGGC

CATCACAGCTGCTGGAGGTATACCTCCACTTGTTCAGATATTAGAGACAGGATCTCCAAAAGCCAAGGAA

GATTCTGCAACAATCCTCGGAAACCTCTGTAATCACAGTGAAGATATTAGAGCATGTGTTGAAAGTGCTG

ATGCTGTTCCTGCTTTGCTGTGGCTATTAAAGAATGGAAGCTCCAATGGCAAAGAAATTGCTGCAAAGAC

ATTGAATCATCTGATCCACAAATCAGATACAGCAACCATTAGTCAGCTCACCGCATTACTTACAAGCGAT

CTACCCGAATCTAAAGTGTATGTTTTAGATGCACTAAAAAGTTTGCTATCTGTGGCCCCTCTTAGTGATA

TGTTGCGTGATGGAAGTGCTTCAAACGATGCTATCGAGACAATGATTAAAATATTAAGTTCTACCAGAGA

TGAGACACAGTCTAAGTCTGCATCAGCTCTTGCTGGAATATTTAATCTTAGAAAGGACTTGCGTGAAAGT

CCCATAGCTGTTAAGACTGTTAGGTCGGTCATGAAGCTTTTACATGTAGAATCCGAAAGCATATTGGCGG

AGTCTACTCGTTGCCTTGCTGCAATATTTCTGTCAATTAAGGAGAACCGTGACATGGCTATGATTGGGAG

AGATTCATTGCCTACACTAGAGGTGCTTGCAACTTCGTCTTCACTGCAAGTTGCAGAGCAGGCCATTTGT

GCTTTGGCTAACCTTCTTTTGGATAGTGAAGTTTCAGAAAAAGCCAAGCCTGAAGAAATTATTTTGCCTT

CTACTAGGGTATTGCGTGAAAGCACAGTTACTGGAAGGAGCCATGCAGCAGCAGCAATTGCCCGGCTTCT

GAGTTCTCGTAAAATTGATGCTGAGATTGCTGACTGTGTGAACCGTACAGGAACAGTGCTTGCATTGGTT

TCGTTCCTAGAATCAGAAAATGCCGGGACTGCTGCCATATCAGAGGGGTTAGAGGCACTTGCCATTATAT

CGAGATCTGGAGGAGATAAAGGACAGAACAAACCTGCATGGGCAGTTCTGGCTGAATTCCCAGATAGCAT

AATCCCGATCGTTTCATGCATTTCTGATGCAAACCCCTTGTTGCTTGATAAGGCTATAGAAATATTGTCA

CGGATTTCTTTGGCTCAACCTGTTGTTTTGGGGAACAATATTGTGAGTGCTTCAGGGTGTATCTCGTCAA

TTGCTAGACGGGTAGTTAGCTCTTCAAAGGAAGCAGTAAAGATTGGAGGCACCACCCTTCTTGTTTGTGC

TGCCAAAGTAAATCTTCAGAGAGTTGTAGATGATTTGAATGAATCATATTCTTGCGCATATCTCATTCAG

TCCCTTGTAGGAATGCTGACCTACACAAAAAATCCTCTGGTTGGTGACCAGGGAAGTAATGAAGGCGTAA

ATATTCTTGGGCATGCTGAAGAACTAAAAGCTGGTGAGAATGAGACGAGTAGATATGTCATCTATGGTTC

AAATACCGCAATATGGCTTCTCTCTGCTCTTGCTAGTAATAACGAAAAGAGCAAAACTCAGATCATGGAA

GCAGGTGCCATTGAAGTTCTAACTGAGAGAATCAATCAGTGTTTATCAGAATATTCTCAGGTCGATTCTA

AAGAGGAAAGCAGCATTTGGATATGTGCTTTACTTCTAGCAATTCTATTTCAAGATAGAGATATTATACG

TGCGCATGCAACAATGAAATCTGTACCTGTACTGGCTAATTTGTTGAAGTCAGAGGAGGCAGCAAACAGA

TATTTTGGTGCACAAGCCATTGCCAGTCTAGTATGCAATGGTAGCAGGGGGACTCTTCTATCAGTCGCTA

ATTCAGGGGCTGCAGGTGGACTCATTTCACTGCTTGGGTGTGCTGATGCTGATATATGTGATATGCTTGA

CTTGGCAGACGAATTTTCCTTGGTGCGGTATCCTGAACAAGTTGCTCTTGAGAGGTTGTTTAGGGTTGAG

GATATCAGAGTTGGTGCTACTTCAAGAAAAGCAATACCTGCACTAGTTGATCTACTTAAACCGATTCCAG

ATCGTCCAGGGGCACCATTTTTAACCTTAGGGCTTCTGCTTCAGCTTGGTAAAGATTGCCCTTCGAATAA

AATTCTTATGGTAGAAGCTGGTGCTCTGGAAGCATTGACCAAATACCTTTCACTTGGCTTACAAGATGCA

ACTGAAGAAGCAGCTACGGATCTCTTAGGTATGTTATTTAGTACTGCTGAGATACGTAGACATGAAGCAG

CATTTGGTGCCGTAAGCCAACTTGTAGCAGTATTGCGATTGGGTGGAAGAGGTGCAAGGTATAGTGCTGC

TTTAGCATTGGAAAGCCTTTTTTCTGCAGACCATATTAGGAATGCAGAATCTTCCAGGCAGGCTGTACAG

CCTTTAGTGGAAATCCTTAATACAGGTTTGGAGAAAGAGCAGCATGCTGCTATTGCTGCATTAGCTAGGT

TACTGAGTGAAAATCCATCAAGAGCCTTGGCAGTTGCTGATGTTGAAATGAATGCGGTAGATGTTCTTTG

CAGGATTCTTTCATCAAACTGTTCACTAGAGCTGAAGGGGGATTCGGCTGAGTTGTGCTGTGTACTTTTT

GGAAATACAAAGATCAGATCCACCCTGGCTGCAGCGCGCTGTGTGGAACCTCTGGTTTCTTTGCTCGTGG

CTGAGTATAGTCCTACTCAGCACTCTGTTGTCCGTGCATTAGATAAACTCGTTGATGACGATCAATTAGC

TGAGCTCATTGCTGCACATGGAGCAGTTATACCTCTTGTAGGCCTTCTGTATGGCCACAACTATGTGCTC

CATGAGGCTATTTCCAGAGCTCTTGTGAAGTTGGGAAAAGACAGGCCTTCTTGTAAGATGGAGATGGTGA

AGGCTGGGGTGATTGAGAGTGTACTTGATATTCTCCATGATGCACCAGATTTTCTGTGTGCTGCTTTTGC

AGAATTGCTACGAATATTAACCAATAATGCTACCATTGCCAAGGGTCCATCTGCAGCGAAAGTGGTTGAG

CCTTTCTTTGTGTTGTTAACAAGAGCAGAGTTCGGACCTGATGGACAGCATAGTGCTTTACAAGTTCTTG

TGAATATCTTAGAACATCCTCAGTGCCGTGCTGATTATACCTTAACATCACATCAAGTGATCGAACCCAT

CATTCCATTACTTGATTCTCCTGCACCGCCAGTGCAGCAGCTAGCAGCTGAACTTCTTTCTCATTTACTC

CTAGAGGAACATCTTCAGAAGGATTCAGTTACACAGCAAGTAATTGGCCCACTTATGCGAGTTCTTGGTT

CTGGTATACACATTCTGCAGCAGAGAGCTGTTAAAGCTCTTGTTAATATTGCACTGATTTGGCCCAATGA

AATTGCCAAGGAGGGTGGCGTCAGTGAGCTATCCAAAGTGATATTGCTAGCTGATCCCTCTTTGCCTCAT

GTCTTGTGGGAATCAGCTGCTTCTGTTTTGTCCAGTATTCTTCAGTTCAGTTCTGAGTACTATTTGGAAG

TGCCTATTGCGGTTTTGGTGAGATTGCTTCGATCTGGTTCAGAATCTACAGTAGTTGGTGCATTAAATGC

TCTTCTAGTTCTGGAAAGTGATGATGCTACAAGTGCTGTAGCAATGGCTGAAAGTGGAGCCATTGAAGCT

CTTTTAGAACTTCTTAGATGTCATCAGTGTGAGGAAACTGCTGCAAGACTTCTGGAGGTATTGCTGAACA

ATATTAAGATCAGAGAAACAAAAGCCACAAAGTCTGCAATAGTACCACTATCACAGTATCTCTTGGATCC

ACAAAGCCAAGCTCAACAAGCAAGATTACTAGCAACTCTTGCTCTAGGGGATTTATTTCAGAATGAGGTT

CTTGCTCGATCTGCTGATGCTGTTTTAGCTTGCCGTGCTTTAGTAAATCTGCTTGAGGATCAACCTTCAG

AAGAAATGAAAGTGGTTGCAATATGTGCATTGCAAAACCTTGTAATGTACAGTAGATCAAATAAGAGAGC

AGTTGCTGAAGCTGGAGGTGTTCAGGTCGTGCTGGATCTGATTGGTTCAAGTGAACCAGATACATCAGTT

CAGGCTGCAATGTTTGTTAAGCTTCTCTTCTCTAACAACACAATTCAAGAGTATGCTTCCAGCGAAACCG

TTAGAGCTATAACAGCTGCAATTGAAAAAGATTTATGGGCTAATGGTGTAGTGAATGAAGAGTATCTTAA

AGCTCTTAATGCACTCCTTGGCAACTTCCCACGTTTAAGAGCCACAGAACCTGCAACCCTGAGCATTCCC

CATCTAGTGACAGCCCTCAAGACTGGCTCAGAGACAACTCAAGAAGCAGCATTGGATTCACTGTCTCTTC

TCAAGCAAGCTTGGGCAGCATGCCCTGCCGAGGTCTCCAGAGCGCAATCAACAGCTGCTTCTGAGGGGAT

TCCCCTGTTACAGTACTTAATTATGTCTGCCCCACCTCGAGTTCAAGATAAGGCAGACCATCTTTTGCAG

TGTCTGCCGGGAACATTGACAGTGACTATAAAACGTGGAAAGAATATCAAACAGTCAGTTGGAAATCCAA

GTGTGTTTTGCAAGCTAACACTTGGCAACACCCCCTCCAGGGAAACCAAGATTGTGTCGACCGGACCAAA

TCCCGAGTGGGATGAGCCCTTCCAGTGGCAATTTGAAAGTCCTCCAAAAGGCCAAAAGCTTCACATTTCT

TGCAAGAACAAGAGCAAGATGGGAAAGAAATCGTTTGGAAAAGTCACTGTCCAGATTGATCGAGTCGTTA

CCCAAGGAGCAGCTGCAGGAGAGTACTTCCTGCTACCTGAAAGCAAGAGTGGTAGTAAAAGGAGCCTGGA

AATAGAATTTCAATGGACTAATAGCAATAACATGCCTCAGTCTGAAGCTTAAAGAGATTTGCCTGGTGTT

AATTTTTTCTTTTTATCGAATATAATTATTGTGCTGGTGTACATAATATCTGGAAGGTTTATTTTTGTAG

TTAGTTGCTGCAATCCGGAAAGTCGGAAACTGCAATTCATTGTATTCTTTTGCAAAGTGTGGTAGTTTTA

GTCATCTTCTGTTCTTTTTGTTATAGTTGTATTGGAGTTGTATTCTGTGTTTGATATTCAGACTCGAGAG

AAAAATTGCTGCTCCTTCCATCTTGGTGGTTTCTTTACGTACCAGCTGGACACGGAGACAAGACAAACAT

ATAATAATTTTTCTATAAATTTTTA

>MSTRG.537.4 gene=MSTRG.537

TCTCTCTCTCTCTCTCTCGTGTAGACATACATTATATAGATATACACACTCTACCATCTCTCTCTCTCTC

TCTCTCTCTCTCTCTGCACAACAATATCTCTATATCATCCATATTGTTGTGAGTCTTGAGCTTCAACTAT

CTTTTCTTCGCTTCTTTCCTACGAAATGCATTGACAAGATCTCCCTCCCTCCCCTTTAGCTCTCTCTCTC

TCTCTGCGTTTATACTGTCCCAATTGTCAGCTACCTACTTCATTCAATCTGTTCTCCAGATCTTCCTAGC

TTATCTCAATCCTCACTTAACTCATTCATGTGAAGCTAGCTTCGCTTTGTAACTGTATTTTGGAGGTAGA

TCTTGAGATCTAATTAGCAAAGCTGGCGGCTACAGTAGCTTGGAGAGGGGTTGCTGCCAGCAATGGCACA

GCCCTGTCATCTAATGATCTGGAAAGAAATGGGGATGCTAAATCTCATGACCAAGAGCCTCCAACACCAC

ATTCACTCTTAAAGATGAGTTCGAGGGAGCGCAGTAGCATGGAGGATCCAGATGGAACTTTAGCTAGTGT

TGCTCAATGCATTGAGCAGTTGCGCCAGAGTTCCTCATCTGCACAAGAAAAAGAGTTCTCCTTAAAGCAA

CTATTGGAGCTTATTGATACACGTGAAAATGCTTTCAGTGCTGTTGGATCACATTCCCAGGCAGTTCCAG

TGTTGGTATCCCTTCTTAGATCAGGATCACTTGGGGTAAAAATACAGGCTGCTACAGTTTTAGGGTCACT

GTGCAAGGAGAATGAACTAAGGGTGAAGGTATTGCTGGGGGGTTGCATTCCACCACTTCTTGGTCTACTC

AGGTCTAGCTCAGCGGAAGCTCAGATCGCTGCAGCGAAGACGATATATGCTGTTTCTCAAGGTGGTGCTA

AGGATCATGTTGGCTCAAAGATATTTTCAACTGAAGGAGTTGTGCCAGTTCTGTGGGGGCAGCTTGAGAA

GGGATTGAAGGCTGGAAACGTGGTTGATAACTTACTGACTGGAGCTTTGAAAAACCTTTCAAGCAGCACT

GAGGGTTTCTGGTTTGCAACAATACAAGCTGGGGGATTAGATACACTTGTAAAGCTGCTTGCAACTGGAC

AATCTAACACCCAAGCAAATGTTTGCTTTCTCCTTGCATGCATGATGATGGAGGATGCATCTGTTTGTCC

TAAAATTCTGGCCGCTGAGACTACCAAGCAACTCCTAAAGCTACTAGGGCCCGGAAATGAAGCCTCGGTC

AGAGCAGAAGCTGCTGGTGCTCTAAAATCTCTATCTGCTCAGTGCAAAGAAGCAAGGCGGGAAATCGCAA

GTTCTAATGGTATACCCGCTTTGATAAATGCTACAATTGCTCCTTCAAAAGAGTTTATGCAAGGTGAGTA

TGCACAGGCTTTGCAGGAGAATGCAATGTGTGCTCTTGCAAATATATCTGGTGGTTTGTCATTTGTCATC

TCAAGTCTTGGCCAAAGTCTTGAATCCTGCACGTCGCCTGCACAGGTAGCTGACACGTTAGGGGCATTAG

CTTCTGCACTAATGATTTATGACAGCAAAGCAGAAAGTACAAGAGCTTCAGATCCTGACGATGTTGAGAA

GACTCTTGTGAAGCAGTTCAAACCTCGCTTGCCATTTCTTGTGCAGGAGCGTACAATAGAAGCTCTTGCC

AGTTTGTATGGAAATTCCACACTTTCTGGTAAACTTGCAAATTCTGATGCAAAGCGTTTGCTAGTTGGTT

TGATCACAATGGCAAGCGATGAAGTTCAGGATGAGTTGATACGATCACTTCTGGTATTGTGTAACAATGA

AGGTACACTATGGCATGCCCTTCAGGGACGTGAGGGAATTCAATTATTGATCTCTCTTCTTGGGCTGTCA

TCTGAGCAACAACAGGAGTGTGCCGTCGCATTACTTTCCCTTTTATCCGATGAGAATGATGAAAGCAAAT

GGGCCATCACAGCTGCTGGAGGTATACCTCCACTTGTTCAGATATTAGAGACAGGATCTCCAAAAGCCAA

GGAAGATTCTGCAACAATCCTCGGAAACCTCTGTAATCACAGTGAAGATATTAGAGCATGTGTTGAAAGT

GCTGATGCTGTTCCTGCTTTGCTGTGGCTATTAAAGAATGGAAGCTCCAATGGCAAAGAAATTGCTGCAA

AGACATTGAATCATCTGATCCACAAATCAGATACAGCAACCATTAGTCAGCTCACCGCATTACTTACAAG

CGATCTACCCGAATCTAAAGTGTATGTTTTAGATGCACTAAAAAGTTTGCTATCTGTGGCCCCTCTTAGT

GATATGTTGCGTGATGGAAGTGCTTCAAACGATGCTATCGAGACAATGATTAAAATATTAAGTTCTACCA

GAGATGAGACACAGTCTAAGTCTGCATCAGCTCTTGCTGGAATATTTAATCTTAGAAAGGACTTGCGTGA

AAGTCCCATAGCTGTTAAGACTGTTAGGTCGGTCATGAAGCTTTTACATGTAGAATCCGAAAGCATATTG

GCGGAGTCTACTCGTTGCCTTGCTGCAATATTTCTGTCAATTAAGGAGAACCGTGACATGGCTATGATTG

GGAGAGATTCATTGCCTACACTAGAGGTGCTTGCAACTTCGTCTTCACTGCAAGTTGCAGAGCAGGCCAT

TTGTGCTTTGGCTAACCTTCTTTTGGATAGTGAAGTTTCAGAAAAAGCCAAGCCTGAAGAAATTATTTTG

CCTTCTACTAGGGTATTGCGTGAAAGCACAGTTACTGGAAGGAGCCATGCAGCAGCAGCAATTGCCCGGC

TTCTGAGTTCTCGTAAAATTGATGCTGAGATTGCTGACTGTGTGAACCGTACAGGAACAGTGCTTGCATT

GGTTTCGTTCCTAGAATCAGAAAATGCCGGGACTGCTGCCATATCAGAGGGGTTAGAGGCACTTGCCATT

ATATCGAGATCTGGAGGAGATAAAGGACAGAACAAACCTGCATGGGCAGTTCTGGCTGAATTCCCAGATA

GCATAATCCCGATCGTTTCATGCATTTCTGATGCAAACCCCTTGTTGCTTGATAAGGCTATAGAAATATT

GTCACGGATTTCTTTGGCTCAACCTGTTGTTTTGGGGAACAATATTGTGAGTGCTTCAGGGTGTATCTCG

TCAATTGCTAGACGGGTAGTTAGCTCTTCAAAGGAAGCAGTAAAGATTGGAGGCACCACCCTTCTTGTTT

GTGCTGCCAAAGTAAATCTTCAGAGAGTTGTAGATGATTTGAATGAATCATATTCTTGCGCATATCTCAT

TCAGTCCCTTGTAGGAATGCTGACCTACACAAAAAATCCTCTGGTTGGTGACCAGGGAAGTAATGAAGGC

GTAAATATTCTTGGGCATGCTGAAGAACTAAAAGCTGGTGAGAATGAGACGAGTAGATATGTCATCTATG

GTTCAAATACCGCAATATGGCTTCTCTCTGCTCTTGCTAGTAATAACGAAAAGAGCAAAACTCAGATCAT

GGAAGCAGGTGCCATTGAAGTTCTAACTGAGAGAATCAATCAGTGTTTATCAGAATATTCTCAGGTCGAT

TCTAAAGAGGAAAGCAGCATTTGGATATGTGCTTTACTTCTAGCAATTCTATTTCAAGATAGAGATATTA

TACGTGCGCATGCAACAATGAAATCTGTACCTGTACTGGCTAATTTGTTGAAGTCAGAGGAGGCAGCAAA

CAGATATTTTGGTGCACAAGCCATTGCCAGTCTAGTATGCAATGGTAGCAGGGGGACTCTTCTATCAGTC

GCTAATTCAGGGGCTGCAGGTGGACTCATTTCACTGCTTGGGTGTGCTGATGCTGATATATGTGATATGC

TTGACTTGGCAGACGAATTTTCCTTGGTGCGGTATCCTGAACAAGTTGCTCTTGAGAGGTTGTTTAGGGT

TGAGGATATCAGAGTTGGTGCTACTTCAAGAAAAGCAATACCTGCACTAGTTGATCTACTTAAACCGATT

CCAGATCGTCCAGGGGCACCATTTTTAACCTTAGGGCTTCTGCTTCAGCTTGGTAAAGATTGCCCTTCGA

ATAAAATTCTTATGGTAGAAGCTGGTGCTCTGGAAGCATTGACCAAATACCTTTCACTTGGCTTACAAGA

TGCAACTGAAGAAGCAGCTACGGATCTCTTAGGTATGTTATTTAGTACTGCTGAGATACGTAGACATGAA

GCAGCATTTGGTGCCGTAAGCCAACTTGTAGCAGTATTGCGATTGGGTGGAAGAGGTGCAAGGTATAGTG

CTGCTTTAGCATTGGAAAGCCTTTTTTCTGCAGACCATATTAGGAATGCAGAATCTTCCAGGCAGGCTGT

ACAGCCTTTAGTGGAAATCCTTAATACAGGTTTGGAGAAAGAGCAGCATGCTGCTATTGCTGCATTAGCT

AGGTTACTGAGTGAAAATCCATCAAGAGCCTTGGCAGTTGCTGATGTTGAAATGAATGCGGTAGATGTTC

TTTGCAGGATTCTTTCATCAAACTGTTCACTAGAGCTGAAGGGGGATTCGGCTGAGTTGTGCTGTGTACT

TTTTGGAAATACAAAGATCAGATCCACCCTGGCTGCAGCGCGCTGTGTGGAACCTCTGGTTTCTTTGCTC

GTGGCTGAGTATAGTCCTACTCAGCACTCTGTTGTCCGTGCATTAGATAAACTCGTTGATGACGATCAAT

TAGCTGAGCTCATTGCTGCACATGGAGCAGTTATACCTCTTGTAGGCCTTCTGTATGGCCACAACTATGT

GCTCCATGAGGCTATTTCCAGAGCTCTTGTGAAGTTGGGAAAAGACAGGCCTTCTTGTAAGATGGAGATG

GTGAAGGCTGGGGTGATTGAGAGTGTACTTGATATTCTCCATGATGCACCAGATTTTCTGTGTGCTGCTT

TTGCAGAATTGCTACGAATATTAACCAATAATGCTACCATTGCCAAGGGTCCATCTGCAGCGAAAGTGGT

TGAGCCTTTCTTTGTGTTGTTAACAAGAGCAGAGTTCGGACCTGATGGACAGCATAGTGCTTTACAAGTT

CTTGTGAATATCTTAGAACATCCTCAGTGCCGTGCTGATTATACCTTAACATCACATCAAGTGATCGAAC

CCATCATTCCATTACTTGATTCTCCTGCACCGCCAGTGCAGCAGCTAGCAGCTGAACTTCTTTCTCATTT

ACTCCTAGAGGAACATCTTCAGAAGGATTCAGTTACACAGCAAGTAATTGGCCCACTTATGCGAGTTCTT

GGTTCTGGTATACACATTCTGCAGCAGAGAGCTGTTAAAGCTCTTGTTAATATTGCACTGATTTGGCCCA

ATGAAATTGCCAAGGAGGGTGGCGTCAGTGAGCTATCCAAAGTGATATTGCTAGCTGATCCCTCTTTGCC

TCATGTCTTGTGGGAATCAGCTGCTTCTGTTTTGTCCAGTATTCTTCAGTTCAGTTCTGAGTACTATTTG

GAAGTGCCTATTGCGGTTTTGGTGAGATTGCTTCGATCTGGTTCAGAATCTACAGTAGTTGGTGCATTAA

ATGCTCTTCTAGTTCTGGAAAGTGATGATGCTACAAGTGCTGTAGCAATGGCTGAAAGTGGAGCCATTGA

AGCTCTTTTAGAACTTCTTAGATGTCATCAGTGTGAGGAAACTGCTGCAAGACTTCTGGAGGTATTGCTG

AACAATATTAAGATCAGAGAAACAAAAGCCACAAAGTCTGCAATAGTACCACTATCACAGTATCTCTTGG

ATCCACAAAGCCAAGCTCAACAAGCAAGATTACTAGCAACTCTTGCTCTAGGGGATTTATTTCAGAATGA

GGTTCTTGCTCGATCTGCTGATGCTGTTTTAGCTTGCCGTGCTTTAGTAAATCTGCTTGAGGATCAACCT

TCAGAAGAAATGAAAGTGGTTGCAATATGTGCATTGCAAAACCTTGTAATGTACAGTAGATCAAATAAGA

GAGCAGTTGCTGAAGCTGGAGGTGTTCAGGTCGTGCTGGATCTGATTGGTTCAAGTGAACCAGATACATC

AGTTCAGGCTGCAATGTTTGTTAAGCTTCTCTTCTCTAACAACACAATTCAAGAGTATGCTTCCAGCGAA

ACCGTTAGAGCTATAACAGCTGCAATTGAAAAAGATTTATGGGCTAATGGTGTAGTGAATGAAGAGTATC

TTAAAGCTCTTAATGCACTCCTTGGCAACTTCCCACGTTTAAGAGCCACAGAACCTGCAACCCTGAGCAT

TCCCCATCTAGTGACAGCCCTCAAGACTGGCTCAGAGACAACTCAAGAAGCAGCATTGGATTCACTGTCT

CTTCTCAAGCAAGCTTGGGCAGCATGCCCTGCCGAGGTCTCCAGAGCGCAATCAACAGCTGCTTCTGAGG

GGATTCCCCTGTTACAGTACTTAATTATGTCTGCCCCACCTCGAGTTCAAGATAAGGCAGACCATCTTTT

GCAGTGTCTGCCGGGAACATTGACAGTGACTATAAAACGTGGAAAGAATATCAAACAGTCAGTTGGAAAT

CCAAGTGTGTTTTGCAAGCTAACACTTGGCAACACCCCCTCCAGGGAAACCAAGATTGTGTCGACCGGAC

CAAATCCCGAGTGGGATGAGCCCTTCCAGTGGCAATTTGAAAGTCCTCCAAAAGGCCAAAAGCTTCACAT

TTCTTGCAAGAACAAGAGCAAGATGGGAAAGAAATCGTTTGGAAAAGTCACTGTCCAGATTGATCGAGTC

GTTACCCAAGGAGCAGCTGCAGGAGAGTACTTCCTGCTACCTGAAAGCAAGAGTGGTAGTAAAAGGAGCC

TGGAAATAGAATTTCAATGGACTAATAGCAATAACATGCCTCAGTCTGAAGCTTAAAGAGATTTGCCTGG

TGTTAATTTTTTCTTTTTATCGAATATAATTATTGTGCTGGTGTACATAATATCTGGAAGGTTTATTTTT

GTAGTTAGTTGCTGCAATCCGGAAAGTCGGAAACTGCAATTCATTGTATTCTTTTGCAAAGTGTGGTAGT

TTTAGTCATCTTCTGTTCTTTTTGTTATAGTTGTATTGGAGTTGTATTCTGTGTTTGATATTCAGACTCG

AGAGGTTAATAAAATCTTGTGTATTGAATTCATTCTTACCTGTTTTCTCTTCAGGGGTTAAGTAA

>MSTRG.537.5 gene=MSTRG.537

TATAGATATACACACTCTACCATCTCTCTCTCTCTCTCTCTCTCTCTGCACAACAATATCTCTATATCAT

CCATATTGTTGTGAGTCTTGAGCTTCAACTATCTTTTCTTCGCTTCTTTCCTACGAAATGCATTGACAAG

ATCTCCCTCCCTCCCCTTTAGCTCTCTCTCTCTCTCTGCGTTTATACTGTCCCAATTGTCAGCTACCTAC

TTCATTCAATCTGTTCTCCAGATCTTCCTAGCTTATCTCAATCCTCACTTAACTCATTCATGTGAAGCTA

GCTTCGCTTTGTAACTGTATTTTGGAGGTAGATCTTGAGATCTAATTAGCAAAGCTGGCGGCTACAGTAG

CTTGGAGAGGGGTTGCTGCCAGCAATGGCACAGCCCTGTCATCTAATGATCTGGAAAGAAATGGGGATGC

TAAATCTCATGACCAAGAGCCTCCAACACCACATTCACTCTTAAAGATGAGTTCGAGGGAGCGCAGTAGC

ATGGAGGATCCAGATGGAACTTTAGCTAGTGTTGCTCAATGCATTGAGCAGTTGCGCCAGAGTTCCTCAT

CTGCACAAGAAAAAGAGTTCTCCTTAAAGCAACTATTGGAGCTTATTGATACACGTGAAAATGCTTTCAG

TGCTGTTGGATCACATTCCCAGGCAGTTCCAGTGTTGGTATCCCTTCTTAGATCAGGATCACTTGGGGTA

AAAATACAGGCTGCTACAGTTTTAGGGTCACTGTGCAAGGAGAATGAACTAAGGGTGAAGGTATTGCTGG

GGGGTTGCATTCCACCACTTCTTGGTCTACTCAGGTCTAGCTCAGCGGAAGCTCAGATCGCTGCAGCGAA

GACGATATATGCTGTTTCTCAAGGTGGTGCTAAGGATCATGTTGGCTCAAAGATATTTTCAACTGAAGGA

GTTGTGCCAGTTCTGTGGGGGCAGCTTGAGAAGGGATTGAAGGCTGGAAACGTGGTTGATAACTTACTGA

CTGGAGCTTTGAAAAACCTTTCAAGCAGCACTGAGGGTTTCTGGTTTGCAACAATACAAGCTGGGGGATT

AGATACACTTGTAAAGCTGCTTGCAACTGGACAATCTAACACCCAAGCAAATGTTTGCTTTCTCCTTGCA

TGCATGATGATGGAGGATGCATCTGTTTGTCCTAAAATTCTGGCCGCTGAGACTACCAAGCAACTCCTAA

AGCTACTAGGGCCCGGAAATGAAGCCTCGGTCAGAGCAGAAGCTGCTGGTGCTCTAAAATCTCTATCTGC

TCAGTGCAAAGAAGCAAGGCGGGAAATCGCAAGTTCTAATGGTATACCCGCTTTGATAAATGCTACAATT

GCTCCTTCAAAAGAGTTTATGCAAGGTGAGTATGCACAGGCTTTGCAGGAGAATGCAATGTGTGCTCTTG

CAAATATATCTGGTGGTTTGTCATTTGTCATCTCAAGTCTTGGCCAAAGTCTTGAATCCTGCACGTCGCC

TGCACAGGTAGCTGACACGTTAGGGGCATTAGCTTCTGCACTAATGATTTATGACAGCAAAGCAGAAAGT

ACAAGAGCTTCAGATCCTGACGATGTTGAGAAGACTCTTGTGAAGCAGTTCAAACCTCGCTTGCCATTTC

TTGTGCAGGAGCGTACAATAGAAGCTCTTGCCAGTTTGTATGGAAATTCCACACTTTCTGGTAAACTTGC

AAATTCTGATGCAAAGCGTTTGCTAGTTGGTTTGATCACAATGGCAAGCGATGAAGTTCAGGATGAGTTG

ATACGATCACTTCTGGTATTGTGTAACAATGAAGGTACACTATGGCATGCCCTTCAGGGACGTGAGGGAA

TTCAATTATTGATCTCTCTTCTTGGGCTGTCATCTGAGCAACAACAGGAGTGTGCCGTCGCATTACTTTC

CCTTTTATCCGATGAGAATGATGAAAGCAAATGGGCCATCACAGCTGCTGGAGGTATACCTCCACTTGTT

CAGATATTAGAGACAGGATCTCCAAAAGCCAAGGAAGATTCTGCAACAATCCTCGGAAACCTCTGTAATC

ACAGTGAAGATATTAGAGCATGTGTTGAAAGTGCTGATGCTGTTCCTGCTTTGCTGTGGCTATTAAAGAA

TGGAAGCTCCAATGGCAAAGAAATTGCTGCAAAGACATTGAATCATCTGATCCACAAATCAGATACAGCA

ACCATTAGTCAGCTCACCGCATTACTTACAAGCGATCTACCCGAATCTAAAGTGTATGTTTTAGATGCAC

TAAAAAGTTTGCTATCTGTGGCCCCTCTTAGTGATATGTTGCGTGATGGAAGTGCTTCAAACGATGCTAT

CGAGACAATGATTAAAATATTAAGTTCTACCAGAGATGAGACACAGTCTAAGTCTGCATCAGCTCTTGCT

GGAATATTTAATCTTAGAAAGGACTTGCGTGAAAGTCCCATAGCTGTTAAGACTGTTAGGTCGGTCATGA

AGCTTTTACATGTAGAATCCGAAAGCATATTGGCGGAGTCTACTCGTTGCCTTGCTGCAATATTTCTGTC

AATTAAGGAGAACCGTGACATGGCTATGATTGGGAGAGATTCATTGCCTACACTAGAGGTGCTTGCAACT

TCGTCTTCACTGCAAGTTGCAGAGCAGGCCATTTGTGCTTTGGCTAACCTTCTTTTGGATAGTGAAGTTT

CAGAAAAAGCCAAGCCTGAAGAAATTATTTTGCCTTCTACTAGGGTATTGCGTGAAAGCACAGTTACTGG

AAGGAGCCATGCAGCAGCAGCAATTGCCCGGCTTCTGAGTTCTCGTAAAATTGATGCTGAGATTGCTGAC

TGTGTGAACCGTACAGGAACAGTGCTTGCATTGGTTTCGTTCCTAGAATCAGAAAATGCCGGGACTGCTG

CCATATCAGAGGGGTTAGAGGCACTTGCCATTATATCGAGATCTGGAGGAGATAAAGGACAGAACAAACC

TGCATGGGCAGTTCTGGCTGAATTCCCAGATAGCATAATCCCGATCGTTTCATGCATTTCTGATGCAAAC

CCCTTGTTGCTTGATAAGGCTATAGAAATATTGTCACGGATTTCTTTGGCTCAACCTGTTGTTTTGGGGA

ACAATATTGTGAGTGCTTCAGGGTGTATCTCGTCAATTGCTAGACGGGTAGTTAGCTCTTCAAAGGAAGC

AGTAAAGATTGGAGGCACCACCCTTCTTGTTTGTGCTGCCAAAGTAAATCTTCAGAGAGTTGTAGATGAT

TTGAATGAATCATATTCTTGCGCATATCTCATTCAGTCCCTTGTAGGAATGCTGACCTACACAAAAAATC

CTCTGGTTGGTGACCAGGGAAGTAATGAAGGCGTAAATATTCTTGGGCATGCTGAAGAACTAAAAGCTGG

TGAGAATGAGACGAGTAGATATGTCATCTATGGTTCAAATACCGCAATATGGCTTCTCTCTGCTCTTGCT

AGTAATAACGAAAAGAGCAAAACTCAGATCATGGAAGCAGGTGCCATTGAAGTTCTAACTGAGAGAATCA

ATCAGTGTTTATCAGAATATTCTCAGGTCGATTCTAAAGAGGAAAGCAGCATTTGGATATGTGCTTTACT

TCTAGCAATTCTATTTCAAGATAGAGATATTATACGTGCGCATGCAACAATGAAATCTGTACCTGTACTG

GCTAATTTGTTGAAGTCAGAGGAGGCAGCAAACAGATATTTTGGTGCACAAGCCATTGCCAGTCTAGTAT

GCAATGGTAGCAGGGGGACTCTTCTATCAGTCGCTAATTCAGGGGCTGCAGGTGGACTCATTTCACTGCT

TGGGTGTGCTGATGCTGATATATGTGATATGCTTGACTTGGCAGACGAATTTTCCTTGGTGCGGTATCCT

GAACAAGTTGCTCTTGAGAGGTTGTTTAGGGTTGAGGATATCAGAGTTGGTGCTACTTCAAGAAAAGCAA

TACCTGCACTAGTTGATCTACTTAAACCGATTCCAGATCGTCCAGGGGCACCATTTTTAACCTTAGGGCT

TCTGCTTCAGCTTGGTAAAGATTGCCCTTCGAATAAAATTCTTATGGTAGAAGCTGGTGCTCTGGAAGCA

TTGACCAAATACCTTTCACTTGGCTTACAAGATGCAACTGAAGAAGCAGCTACGGATCTCTTAGGTATGT

TATTTAGTACTGCTGAGATACGTAGACATGAAGCAGCATTTGGTGCCGTAAGCCAACTTGTAGCAGTATT

GCGATTGGGTGGAAGAGGTGCAAGGTATAGTGCTGCTTTAGCATTGGAAAGCCTTTTTTCTGCAGACCAT

ATTAGGAATGCAGAATCTTCCAGGCAGGCTGTACAGCCTTTAGTGGAAATCCTTAATACAGGTTTGGAGA

AAGAGCAGCATGCTGCTATTGCTGCATTAGCTAGGTTACTGAGTGAAAATCCATCAAGAGCCTTGGCAGT

TGCTGATGTTGAAATGAATGCGGTAGATGTTCTTTGCAGGATTCTTTCATCAAACTGTTCACTAGAGCTG

AAGGGGGATTCGGCTGAGTTGTGCTGTGTACTTTTTGGAAATACAAAGATCAGATCCACCCTGGCTGCAG

CGCGCTGTGTGGAACCTCTGGTTTCTTTGCTCGTGGCTGAGTATAGTCCTACTCAGCACTCTGTTGTCCG

TGCATTAGATAAACTCGTTGATGACGATCAATTAGCTGAGCTCATTGCTGCACATGGAGCAGTTATACCT

CTTGTAGGCCTTCTGTATGGCCACAACTATGTGCTCCATGAGGCTATTTCCAGAGCTCTTGTGAAGTTGG

GAAAAGACAGGCCTTCTTGTAAGATGGAGATGGTGAAGGCTGGGGTGATTGAGAGTGTACTTGATATTCT

CCATGATGCACCAGATTTTCTGTGTGCTGCTTTTGCAGAATTGCTACGAATATTAACCAATAATGCTACC

ATTGCCAAGGGTCCATCTGCAGCGAAAGTGGTTGAGCCTTTCTTTGTGTTGTTAACAAGAGCAGAGTTCG

GACCTGATGGACAGCATAGTGCTTTACAAGTTCTTGTGAATATCTTAGAACATCCTCAGTGCCGTGCTGA

TTATACCTTAACATCACATCAAGTGATCGAACCCATCATTCCATTACTTGATTCTCCTGCACCGCCAGTG

CAGCAGCTAGCAGCTGAACTTCTTTCTCATTTACTCCTAGAGGAACATCTTCAGAAGGATTCAGTTACAC

AGCAAGTAATTGGCCCACTTATGCGAGTTCTTGGTTCTGGTATACACATTCTGCAGCAGAGAGCTGTTAA

AGCTCTTGTTAATATTGCACTGATTTGGCCCAATGAAATTGCCAAGGAGGGTGGCGTCAGTGAGCTATCC

AAAGTGATATTGCTAGCTGATCCCTCTTTGCCTCATGTCTTGTGGGAATCAGCTGCTTCTGTTTTGTCCA

GTATTCTTCAGTTCAGTTCTGAGTACTATTTGGAAGTGCCTATTGCGGTTTTGGTGAGATTGCTTCGATC

TGGTTCAGAATCTACAGTAGTTGGTGCATTAAATGCTCTTCTAGTTCTGGAAAGTGATGATGCTACAAGT

GCTGTAGCAATGGCTGAAAGTGGAGCCATTGAAGCTCTTTTAGAACTTCTTAGATGTCATCAGTGTGAGG

AAACTGCTGCAAGACTTCTGGAGGTATTGCTGAACAATATTAAGATCAGAGAAACAAAAGCCACAAAGTC

TGCAATAGTACCACTATCACAGTATCTCTTGGATCCACAAAGCCAAGCTCAACAAGCAAGATTACTAGCA

ACTCTTGCTCTAGGGGATTTATTTCAGAATGAGGTTCTTGCTCGATCTGCTGATGCTGTTTTAGCTTGCC

GTGCTTTAGTAAATCTGCTTGAGGATCAACCTTCAGAAGAAATGAAAGTGGTTGCAATATGTGCATTGCA

AAACCTTGTAATGTACAGTAGATCAAATAAGAGAGCAGTTGCTGAAGCTGGAGGTGTTCAGGTCGTGCTG

GATCTGATTGGTTCAAGTGAACCAGATACATCAGTTCAGGCTGCAATGTTTGTTAAGCTTCTCTTCTCTA

ACAACACAATTCAAGAGTATGCTTCCAGCGAAACCGTTAGAGCTATAACAGCTGCAATTGAAAAAGATTT

ATGGGCTAATGGTGTAGTGAATGAAGAGTATCTTAAAGCTCTTAATGCACTCCTTGGCAACTTCCCACGT

TTAAGAGCCACAGAACCTGCAACCCTGAGCATTCCCCATCTAGTGACAGCCCTCAAGACTGGCTCAGAGA

CAACTCAAGAAGCAGCATTGGATTCACTGTCTCTTCTCAAGCAAGCTTGGGCAGCATGCCCTGCCGAGGT

CTCCAGAGCGCAATCAACAGCTGCTTCTGAGGGGATTCCCCTGTTACAGTACTTAATTATGTCTGCCCCA

CCTCGAGTTCAAGATAAGGCAGACCATCTTTTGCAGTGTCTGCCGGGAACATTGACAGTGACTATAAAAC

GTGGAAAGAATATCAAACAGTCAGTTGGAAATCCAAGTGTGTTTTGCAAGCTAACACTTGGCAACACCCC

CTCCAGGGAAACCAAGATTGTGTCGACCGGACCAAATCCCGAGTGGGATGAGCCCTTCCAGTGGCAATTT

GAAAGTCCTCCAAAAGGCCAAAAGCTTCACATTTCTTGCAAGAACAAGAGCAAGATGGGAAAGAAATCGT

TTGGAAAAGTCACTGTCCAGATTGATCGAGTCGTTACCCAAGGAGCAGCTGCAGGAGAGTACTTCCTGCT

ACCTGAAAGCAAGAGTGGTAGTAAAAGGAGCCTGGAAATAGAATTTCAATGGACTAATAGCAATAACATG

CCTCAGTCTGAAGCTTAAAGAGATTTGCCTGGTGTTAATTTTTTCTTTTTATCGAATATAATTATTGTGC

TGGTGTACATAATATCTGGAAGGTTTATTTTTGTAGTTAGTTGCTGCAATCCGGAAAGTCGGAAACTGCA

ATTCATTGTATTCTTTTGCAAAGTGTGGTAGTTTTAGTCATCTTCTGTTCTTTTTGTTATAGTTGTATTG

GAGTTGTATTCTGTGTTTGATATTCAGACTCGAGAGGTTAATAAAATCTTGTGTATTGAATTCA

>MSTRG.537.6 gene=MSTRG.537

TATAGATATACACACTCTACCATCTCTCTCTCTCTCTCTCTCTCTCTGCACAACAATATCTCTATATCAT

CCATATTGTTGTGAGTCTTGAGCTTCAACTATCTTTTCTTCGCTTCTTTCCTACGAAATGCATTGACAAG

ATCTCCCTCCCTCCCCTTTAGCTCTCTCTCTCTCTCTGCGTTTATACTGTCCCAATTGTCAGCTACCTAC

TTCATTCAATCTGTTCTCCAGATCTTCCTAGCTTATCTCAATCCTCACTTAACTCATTCATGTGAAGCTA

GCTTCGCTTTGTAACTGTATTTTGGAGGTAGATCTTGAGATCTAATTAGCAAAGCTGGCGGCTACAGTAG

CTTGGAGAGGGGTTGCTGCCAGCAATGGCACAGCCCTGTCATCTAATGATCTGGAAAGAAATGGGGATGC

TAAATCTCATGACCAAGAGCCTCCAACACCACATTCACTCTTAAAGATGAGTTCGAGGGAGCGCAGTAGC

ATGGAGGATCCAGATGGAACTTTAGCTAGTGTTGCTCAATGCATTGAGCAGTTGCGCCAGAGTTCCTCAT

CTGCACAAGAAAAAGAGTTCTCCTTAAAGCAACTATTGGAGCTTATTGATACACGTGAAAATGCTTTCAG

TGCTGTTGGATCACATTCCCAGGCAGTTCCAGTGTTGGTATCCCTTCTTAGATCAGGATCACTTGGGGTA

AAAATACAGGCTGCTACAGTTTTAGGGTCACTGTGCAAGGAGAATGAACTAAGGGTGAAGGTATTGCTGG

GGGGTTGCATTCCACCACTTCTTGGTCTACTCAGGTCTAGCTCAGCGGAAGCTCAGATCGCTGCAGCGAA

GACGATATATGCTGTTTCTCAAGGTGGTGCTAAGGATCATGTTGGCTCAAAGATATTTTCAACTGAAGGA

GTTGTGCCAGTTCTGTGGGGGCAGCTTGAGAAGGGATTGAAGGCTGGAAACGTGGTTGATAACTTACTGA

CTGGAGCTTTGAAAAACCTTTCAAGCAGCACTGAGGGTTTCTGGTTTGCAACAATACAAGCTGGGGGATT

AGATACACTTGTAAAGCTGCTTGCAACTGGACAATCTAACACCCAAGCAAATGTTTGCTTTCTCCTTGCA

TGCATGATGATGGAGGATGCATCTGTTTGTCCTAAAATTCTGGCCGCTGAGACTACCAAGCAACTCCTAA

AGCTACTAGGGCCCGGAAATGAAGCCTCGGTCAGAGCAGAAGCTGCTGGTGCTCTAAAATCTCTATCTGC

TCAGTGCAAAGAAGCAAGGCGGGAAATCGCAAGTTCTAATGGTATACCCGCTTTGATAAATGCTACAATT

GCTCCTTCAAAAGAGTTTATGCAAGGTGAGTATGCACAGGCTTTGCAGGAGAATGCAATGTGTGCTCTTG

CAAATATATCTGGTGGTTTGTCATTTGTCATCTCAAGTCTTGGCCAAAGTCTTGAATCCTGCACGTCGCC

TGCACAGGTAGCTGACACGTTAGGGGCATTAGCTTCTGCACTAATGATTTATGACAGCAAAGCAGAAAGT

ACAAGAGCTTCAGATCCTGACGATGTTGAGAAGACTCTTGTGAAGCAGTTCAAACCTCGCTTGCCAGTTT

GTATGGAAATTCCACACTTTCTGGTAAACTTGCAAATTCTGATGCAAAGCGTTTGCTAGTTGGTTTGATC

ACAATGGCAAGCGATGAAGTTCAGGATGAGTTGATACGATCACTTCTGGTATTGTGTAACAATGAAGGTA

CACTATGGCATGCCCTTCAGGGACGTGAGGGAATTCAATTATTGATCTCTCTTCTTGGGCTGTCATCTGA

GCAACAACAGGAGTGTGCCGTCGCATTACTTTCCCTTTTATCCGATGAGAATGATGAAAGCAAATGGGCC

ATCACAGCTGCTGGAGGTATACCTCCACTTGTTCAGATATTAGAGACAGGATCTCCAAAAGCCAAGGAAG

ATTCTGCAACAATCCTCGGAAACCTCTGTAATCACAGTGAAGATATTAGAGCATGTGTTGAAAGTGCTGA

TGCTGTTCCTGCTTTGCTGTGGCTATTAAAGAATGGAAGCTCCAATGGCAAAGAAATTGCTGCAAAGACA

TTGAATCATCTGATCCACAAATCAGATACAGCAACCATTAGTCAGCTCACCGCATTACTTACAAGCGATC

TACCCGAATCTAAAGTGTATGTTTTAGATGCACTAAAAAGTTTGCTATCTGTGGCCCCTCTTAGTGATAT

GTTGCGTGATGGAAGTGCTTCAAACGATGCTATCGAGACAATGATTAAAATATTAAGTTCTACCAGAGAT

GAGACACAGTCTAAGTCTGCATCAGCTCTTGCTGGAATATTTAATCTTAGAAAGGACTTGCGTGAAAGTC

CCATAGCTGTTAAGACTGTTAGGTCGGTCATGAAGCTTTTACATGTAGAATCCGAAAGCATATTGGCGGA

GTCTACTCGTTGCCTTGCTGCAATATTTCTGTCAATTAAGGAGAACCGTGACATGGCTATGATTGGGAGA

GATTCATTGCCTACACTAGAGGTGCTTGCAACTTCGTCTTCACTGCAAGTTGCAGAGCAGGCCATTTGTG

CTTTGGCTAACCTTCTTTTGGATAGTGAAGTTTCAGAAAAAGCCAAGCCTGAAGAAATTATTTTGCCTTC

TACTAGGGTATTGCGTGAAAGCACAGTTACTGGAAGGAGCCATGCAGCAGCAGCAATTGCCCGGCTTCTG

AGTTCTCGTAAAATTGATGCTGAGATTGCTGACTGTGTGAACCGTACAGGAACAGTGCTTGCATTGGTTT

CGTTCCTAGAATCAGAAAATGCCGGGACTGCTGCCATATCAGAGGGGTTAGAGGCACTTGCCATTATATC

GAGATCTGGAGGAGATAAAGGACAGAACAAACCTGCATGGGCAGTTCTGGCTGAATTCCCAGATAGCATA

ATCCCGATCGTTTCATGCATTTCTGATGCAAACCCCTTGTTGCTTGATAAGGCTATAGAAATATTGTCAC

GGATTTCTTTGGCTCAACCTGTTGTTTTGGGGAACAATATTGTGAGTGCTTCAGGGTGTATCTCGTCAAT

TGCTAGACGGGTAGTTAGCTCTTCAAAGGAAGCAGTAAAGATTGGAGGCACCACCCTTCTTGTTTGTGCT

GCCAAAGTAAATCTTCAGAGAGTTGTAGATGATTTGAATGAATCATATTCTTGCGCATATCTCATTCAGT

CCCTTGTAGGAATGCTGACCTACACAAAAAATCCTCTGGTTGGTGACCAGGGAAGTAATGAAGGCGTAAA

TATTCTTGGGCATGCTGAAGAACTAAAAGCTGGTGAGAATGAGACGAGTAGATATGTCATCTATGGTTCA

AATACCGCAATATGGCTTCTCTCTGCTCTTGCTAGTAATAACGAAAAGAGCAAAACTCAGATCATGGAAG

CAGGTGCCATTGAAGTTCTAACTGAGAGAATCAATCAGTGTTTATCAGAATATTCTCAGGTCGATTCTAA

AGAGGAAAGCAGCATTTGGATATGTGCTTTACTTCTAGCAATTCTATTTCAAGATAGAGATATTATACGT

GCGCATGCAACAATGAAATCTGTACCTGTACTGGCTAATTTGTTGAAGTCAGAGGAGGCAGCAAACAGAT

ATTTTGGTGCACAAGCCATTGCCAGTCTAGTATGCAATGGTAGCAGGGGGACTCTTCTATCAGTCGCTAA

TTCAGGGGCTGCAGGTGGACTCATTTCACTGCTTGGGTGTGCTGATGCTGATATATGTGATATGCTTGAC

TTGGCAGACGAATTTTCCTTGGTGCGGTATCCTGAACAAGTTGCTCTTGAGAGGTTGTTTAGGGTTGAGG

ATATCAGAGTTGGTGCTACTTCAAGAAAAGCAATACCTGCACTAGTTGATCTACTTAAACCGATTCCAGA

TCGTCCAGGGGCACCATTTTTAACCTTAGGGCTTCTGCTTCAGCTTGGTAAAGATTGCCCTTCGAATAAA

ATTCTTATGGTAGAAGCTGGTGCTCTGGAAGCATTGACCAAATACCTTTCACTTGGCTTACAAGATGCAA

CTGAAGAAGCAGCTACGGATCTCTTAGGTATGTTATTTAGTACTGCTGAGATACGTAGACATGAAGCAGC

ATTTGGTGCCGTAAGCCAACTTGTAGCAGTATTGCGATTGGGTGGAAGAGGTGCAAGGTATAGTGCTGCT

TTAGCATTGGAAAGCCTTTTTTCTGCAGACCATATTAGGAATGCAGAATCTTCCAGGCAGGCTGTACAGC

CTTTAGTGGAAATCCTTAATACAGGTTTGGAGAAAGAGCAGCATGCTGCTATTGCTGCATTAGCTAGGTT

ACTGAGTGAAAATCCATCAAGAGCCTTGGCAGTTGCTGATGTTGAAATGAATGCGGTAGATGTTCTTTGC

AGGATTCTTTCATCAAACTGTTCACTAGAGCTGAAGGGGGATTCGGCTGAGTTGTGCTGTGTACTTTTTG

GAAATACAAAGATCAGATCCACCCTGGCTGCAGCGCGCTGTGTGGAACCTCTGGTTTCTTTGCTCGTGGC

TGAGTATAGTCCTACTCAGCACTCTGTTGTCCGTGCATTAGATAAACTCGTTGATGACGATCAATTAGCT

GAGCTCATTGCTGCACATGGAGCAGTTATACCTCTTGTAGGCCTTCTGTATGGCCACAACTATGTGCTCC

ATGAGGCTATTTCCAGAGCTCTTGTGAAGTTGGGAAAAGACAGGCCTTCTTGTAAGATGGAGATGGTGAA

GGCTGGGGTGATTGAGAGTGTACTTGATATTCTCCATGATGCACCAGATTTTCTGTGTGCTGCTTTTGCA

GAATTGCTACGAATATTAACCAATAATGCTACCATTGCCAAGGGTCCATCTGCAGCGAAAGTGGTTGAGC

CTTTCTTTGTGTTGTTAACAAGAGCAGAGTTCGGACCTGATGGACAGCATAGTGCTTTACAAGTTCTTGT

GAATATCTTAGAACATCCTCAGTGCCGTGCTGATTATACCTTAACATCACATCAAGTGATCGAACCCATC

ATTCCATTACTTGATTCTCCTGCACCGCCAGTGCAGCAGCTAGCAGCTGAACTTCTTTCTCATTTACTCC

TAGAGGAACATCTTCAGAAGGATTCAGTTACACAGCAAGTAATTGGCCCACTTATGCGAGTTCTTGGTTC

TGGTATACACATTCTGCAGCAGAGAGCTGTTAAAGCTCTTGTTAATATTGCACTGATTTGGCCCAATGAA

ATTGCCAAGGAGGGTGGCGTCAGTGAGCTATCCAAAGTGATATTGCTAGCTGATCCCTCTTTGCCTCATG

TCTTGTGGGAATCAGCTGCTTCTGTTTTGTCCAGTATTCTTCAGTTCAGTTCTGAGTACTATTTGGAAGT

GCCTATTGCGGTTTTGGTGAGATTGCTTCGATCTGGTTCAGAATCTACAGTAGTTGGTGCATTAAATGCT

CTTCTAGTTCTGGAAAGTGATGATGCTACAAGTGCTGTAGCAATGGCTGAAAGTGGAGCCATTGAAGCTC

TTTTAGAACTTCTTAGATGTCATCAGTGTGAGGAAACTGCTGCAAGACTTCTGGAGGTATTGCTGAACAA

TATTAAGATCAGAGAAACAAAAGCCACAAAGTCTGCAATAGTACCACTATCACAGTATCTCTTGGATCCA

CAAAGCCAAGCTCAACAAGCAAGATTACTAGCAACTCTTGCTCTAGGGGATTTATTTCAGAATGAGGTTC

TTGCTCGATCTGCTGATGCTGTTTTAGCTTGCCGTGCTTTAGTAAATCTGCTTGAGGATCAACCTTCAGA

AGAAATGAAAGTGGTTGCAATATGTGCATTGCAAAACCTTGTAATGTACAGTAGATCAAATAAGAGAGCA

GTTGCTGAAGCTGGAGGTGTTCAGGTCGTGCTGGATCTGATTGGTTCAAGTGAACCAGATACATCAGTTC

AGGCTGCAATGTTTGTTAAGCTTCTCTTCTCTAACAACACAATTCAAGAGTATGCTTCCAGCGAAACCGT

TAGAGCTATAACAGCTGCAATTGAAAAAGATTTATGGGCTAATGGTGTAGTGAATGAAGAGTATCTTAAA

GCTCTTAATGCACTCCTTGGCAACTTCCCACGTTTAAGAGCCACAGAACCTGCAACCCTGAGCATTCCCC

ATCTAGTGACAGCCCTCAAGACTGGCTCAGAGACAACTCAAGAAGCAGCATTGGATTCACTGTCTCTTCT

CAAGCAAGCTTGGGCAGCATGCCCTGCCGAGGTCTCCAGAGCGCAATCAACAGCTGCTTCTGAGGGGATT

CCCCTGTTACAGTACTTAATTATGTCTGCCCCACCTCGAGTTCAAGATAAGGCAGACCATCTTTTGCAGT

GTCTGCCGGGAACATTGACAGTGACTATAAAACGTGGAAAGAATATCAAACAGTCAGTTGGAAATCCAAG

TGTGTTTTGCAAGCTAACACTTGGCAACACCCCCTCCAGGGAAACCAAGATTGTGTCGACCGGACCAAAT

CCCGAGTGGGATGAGCCCTTCCAGTGGCAATTTGAAAGTCCTCCAAAAGGCCAAAAGCTTCACATTTCTT

GCAAGAACAAGAGCAAGATGGGAAAGAAATCGTTTGGAAAAGTCACTGTCCAGATTGATCGAGTCGTTAC

CCAAGGAGCAGCTGCAGGAGAGTACTTCCTGCTACCTGAAAGCAAGAGTGGTAGTAAAAGGAGCCTGGAA

ATAGAATTTCAATGGACTAATAGCAATAACATGCCTCAGTCTGAAGCTTAAAGAGATTTGCCTGGTGTTA

ATTTTTTCTTTTTATCGAATATAATTATTGTGCTGGTGTACATAATATCTGGAAGGTTTATTTTTGTAGT

TAGTTGCTGCAATCCGGAAAGTCGGAAACTGCAATTCATTGTATTCTTTTGCAAAGTGTGGTAGTTTTAG

TCATCTTCTGTTCTTTTTGTTATAGTTGTATTGGAGTTGTATTCTGTGTTTGATATTCAGACTCGAGAGG

TTAATAAAATCTTGTGTATTGAATTCA

>MSTRG.541.1 gene=MSTRG.541

CAAGAAAAAGAAGAAAAGGGAAAAGAAAAGAAGAAAAAAAGAGAGTTGGCAACTAAGGGACACATGGAGG

GCCACAATTCTCCCAACACTCAAGGCACATGGATGGTCAAGATGGAGTCACCCTACCCCTAAACCCTAAT

TCTTTAGTTATAAATACCCCTCTTCTTTTACTTGTAATCACCAACCTAGCAACCTAGTAACCCTAGCCAC

CTACTCACCTTTTCTACCTAGTCATTTCTATAGTTAGTATCTTAGATAGATTTACATTTTGCTAGCCCCA

ATTTCCTTTCCAAGCTTGTATACACTTTTTAATTTCTA

>MSTRG.545.1 gene=MSTRG.545

TTCCCCCCTTTCTCATCTCTCACTCTTTTGTTGTGCCTTCAGACTTTTCATAAATCCATACCTACCCACA

GTGTATACACACTCCAGCTCTCATTCAAAATAATAGTTTTTCATGGGCAACTCTCATCCGGCCTCTGCCA

TTCACGACTTGGGGGTTTTGTTCCAGATCTGGTGCACTTCTTCCATTAGGGTTAAGATTGGAACCAATTT

AAATGGGGATTTATACATCGAGTAGATTAGACTTGTAATAAGGTATTATTCTCAATTTTAACTATTTTTC

ATCCTCTCTTTGTCTCTCTTCTTTTGTCA

>MSTRG.546.1 gene=MSTRG.546

CTTGAGAGGAGAAATTTGGAAGATTTCCAGGAGAATTCAAGGACGAGATTTAGAAGAAAACAACACTAGC

TAATAATGAGTTCAAATAATTAAGTAAAGACAGAAAATGGCACAAATGAGAAGGGTAATTCGGAAGCATA

TAATAGCAAAATCAATGGCAAAGCGTGAGGCTGTGGCACGAAGGGTGCGAAGACTTGTTCCAGTAACGAG

AATGTTTAATGATTATAATAATGGTAGATTGTACTCTTGAACTTATCATTGTAATGTTAGAATAGACTGC

GTGGAAGTGTAAGATCGACCCTGGACTTGTGGGGCGAGCTATGTAACGTAAGACAGGTATGCTATTACAG

TGTGACGGCCTAGATCCGGGATATACGGATCTGGGGGCGTTACAG

>MSTRG.546.2 gene=MSTRG.546

GAGAAATTTGGAAGATTTCCAGGAGAATTCAAGGACGAGATTTAGAAGAAAACAACACTAGCTAATAATG

AGTTCAAATAATTAAGAATATTTACTATCTGGAAAGGCGAGCTCAATTTGAGACAAGAATATTTTGTGTT

ATTATCAAAAGACGCCAGTAAAGACAGAAAATGGCACAAATGAGAAGGGTAATTCGGAAGCATATAATAG

CAAAATCAATGGCAAAGCGTGAGGCTGTGGCACGAAGGGTGCGAAGACTTGTTCCAGTAACGAGAATGTT

TAATGATTATAATAATGGTAGATTGTACTCTTGAACTTATCATTGTAATGTTAGAATAGACTGCGTGGAA

GTGTAAGATCGACCCTGGACTTGTGGGGCGAGCTATGTAACGTAAGACAGGTATGCTATTACAGTGTGAC

GGCCTAGATCCGGGATATACGGATCTGGGGGCGTTACAG

>MSTRG.547.1 gene=MSTRG.547

AAACAAATGGTAGACTCCTGATAGCACGGAGTTGGCTGCTTCTTCCTACAATTCTTTCATTCACGAAAAG

AGGGTCTTCTTTGGAGAAGATTTGCAGTTTATTGAGGCCTGAACCTTTGATTCCTTTCGCGGCTTTCTCG

ATTTTTGATTCTCAGATTTCTGATTCGAAGTATTCTATCCCTGTTGAATAACCTTTTAACAAAAAGGTTC

TTTATATTTAAGTCCCATATCTTCTTTCCTTGGCTGTGATGCCCTCCCTTTCTTGCCATTCTTTTCACGG

TTGTATAGCTCAC

>MSTRG.550.1 gene=MSTRG.550

TAGGGCTGTAAATGAGTTGATACGCTCGATAACCGCTCGATGTTCGGTTCGAAGAAAGCTCGGTTCGACC

TCGGTTCGATTCATAAACGAGTCGATCTTGAGCACAATTTTAAGGTTCGTTTTATAAACGAGCTGAACTT

GAGCACAGTAGAGTTCGACTCGATAGTTCGCGAACAAGTTCGAATTATGAGTTCGTGAACAGAGTTCGTG

AACATGGTTCGTGAACCTGGCTCGTG

>MSTRG.551.1 gene=MSTRG.551

GTGGTAGAATTAACTAGGTTGAGCTCATTAGTACTCGAGTAATTAAGTCTGAGGGGACTTTGTGCCTAAC

AACCTAAAGCCCTTCGTGGTTTGGGATTGTTGACCCAACGCTCGCTACATGGGTACTAGTGCATAAATCT

TTAGGGATCTCAACCATTGCACGGTTAAATAAACCACTAGAATAGAGTGAATAATTGGTGTGTGAAGTCT

TGTGGTGTTCGTAACGCATTTATACTGCGAAGCGCTCTGACCTTAGACCGAGCAATTAATCACCAATAAA

AAGAAAAAAAGAAGAAAAAAGATATAGTGAATAAAATAGAAGGGTGTGGACATTCTTTGGGACCTTGGCT

TTTAGTTGGACTTGAGTGACGGG

>MSTRG.552.1 gene=MSTRG.552

GTGCCTATTTTTTGTTTATTTTCGTAGTATTAATCGCTTATTTATTTTATTTCAGGAAATTGTAGATAAA

TAAAGAAAGAAGAGAAAATGCAAGAAAAAGAAGAAAAGGAAAAGAAAAGAAGAAAAAAAAAGAGTTGGCA

AATAAGGGACACATGGAGGGCCACAATCTTCCCAACACACAAGGCACATGGATGGTCAAGATTGAGCCAC

CCTACCCCTAAACCCTAATTCTTTAGTTATAAATACC

>MSTRG.555.1 gene=MSTRG.555

GTTGGTTTAGGGAGAACATTAGTGATACTTGTCCTATAGGGATGAACGATTGTCACACGCTAGAGAAAGT

CTTTGTGCATAAAAATTTTCGAAAATATTTTGACTCTTTCTCGAAGCTTCCTCCCATCTTTCCTTCCGGA

ATTGGTTGATTTGCTTCCGTCTTCAAACCTTCCTTCATGAAGTTCAGGTATGGGGAAGGAAATGTGTGTG

TGCGTGGGTAGTGTTAGTGTTTCGTTCGTTAATAAAAATCCCCTTCATATCCATTTCAGGTATTTTCTCT

TTACTCTCTCATTTCAATTCTCTCTTTTGCATACATTGAGGACAATGCATGATTTAGGTATGGGGAGGGC

TTTAGTGTAATTCTTAAAAAATGAAAATCCCTAAAAAATGAAAAAAT

>MSTRG.556.1 gene=MSTRG.556

AAAAAAAAGAGAAGAATAAGAGTTTGTGAAGTTTGGCTAGATATTTGTTTCGATAGAGATTGGGTCAGTT

CAAAATTGGTTGAAAAAGAGAAAATGTTATTAGACGATTAGGATCCATCAAAGGCCTTAAATTACTTACA

CTCCTTGGATTTCTAGTAAATTTTTGTGTCGATTCAAGTAGATTAGAGACAACTGTATCTTGATTATTAG

TAGATGAGTCTTTAAGTCGTATTTTCCTTAAAAACGCTTTTTGTTAACACCAACGAAATTAGAGTAGAGT

CGAGTATACACTTGCTAGAATGTCTCGAAGATTTTATGGGTATATCTTCTGTAAACCCTTAGGAGACAAC

>MSTRG.557.1 gene=MSTRG.557

TTACTCCCTCCGTCCCCCTCAATTGTTTACATTGGAGGGGGACACGGAGACCAAGACAATGTATGAAAAA

TGAGTAAAATTAGGTGAAAAGTGAGTAAAGTGGTGGGACCCATCAATATTTAATAATAGATTTGAGATAG

TGGAGGAAGGTAGTGGGTGTAATAGTAGTTTTTATTGTTAAATATGAGATAGTGGAAGAAGATAGTGGGT

GTAATGATGAAAAAACTTACTATTTATGGTAACGTAAAGAAATGAGAGGGACATCCCAAAATAGTAACGG

TAAAGAAATG

>MSTRG.559.1 gene=MSTRG.559

GAAAAATTCCTGCAAAAAGCGTTACAGTGAACACGAGATTCCTATAAGCAATTGGATCAGCCTCAGGGTC

TTGAACTTTTTGAACCATTGAAGATATCAAGAGTGAAACTACAGCCACTGGTCCGATAGCTATTTCCCGT

GAACTTCCCATGAACGCATAGATGATCGGAGGGACAACGTTCGTGTCTGGAATCAAAAAC

>MSTRG.561.1 gene=MSTRG.561

TGATAAGTGGTATTTATACACACTTATAGGCCTTCATTTCCACTTAAATTGGTTGATTGTACTTAAGTGT

TTAGTGTCTTTTGATGTGTTTTTATTGTTTTTCTGTGCAGGTCACGAGTTGAGGTGATGAAGTGATTTTC

TATCATTTTAGGTTGGTTTTGGTGCATTATTTGCAAGAATAGAGAATTGTTGATTCACCGCGACTTGGGA

TTTTGTGGGTATATCTTCTACAAACCCTCACGAGAGTACACTCGTCCACTAGAGCTATCTAGGGGTTTAA

AGGGCTTGTTGCATATGCTAAATGCAACCGTGATCACCTACGGAAGTGGTACTAGAAGCGAGGAAGGACA

TGCACG

>MSTRG.562.1 gene=MSTRG.562

TGGGTACTAGTGCATAAATCTTTAGGGATCTCAACCATTGCACGGTTAAATAAACCACTAGAATAGAGTG

AATAATTGGTGTGTGAAGTCTTGTGGTGTTCGTAACGCATTTACACTGCGAAGCGCTCTGACCTTAGACC

GAGCAATTAATCACCAATAAAAAAAAAAAAAAAATAGTGAATAAAATAGAAGGGTGTGGACATTCTTTGG

GACCTTGGCTTTTAGTTGGACTTGAGTGACGGGGTGTTAGTTTTGAACTTTTACGATTCTTGATTAGGAT

TCTGTGGGAGTAGTAGTTTTTTATTAGTTTTAAACTTTTACGATTCTTGATTAGGATTCTGTGGGAGTAG

TAGTTTTTGTCTTGTCTCAATAAAAGAGCATGCTTGCACACACTGGCACTCCACACTTGTAAATAGAAAA

GTAATTGACTTGGTAGCGAGAGAGATGAAATTCAAGAACATTAGCACAATCTGAGGTATTATAATTGACA

AGGCAATTACTTCATAGTTCACTCATTCATCATGTAGTTGCATTCATGCATTGCATTGTATTATTGTATA

GTGTCGTTGACGCTTGAGGACAAGCATCAGTTTAAGTTTGGGGGTGTGATAAGTGGTATTTATACACACT

TATAGGCCTTCATTTCCACTTAAATTGGTTGGTTGTACTTAAGTGTTTAGTGTCTTTTGATGTGTTTTTA

GTGTTTTTCTGTGCAGGTCACGAGTTG

>MSTRG.563.1 gene=MSTRG.563

TTTTAAATTGTTTGAGGGGAATAGTAACTTGTGAATATCTATCCCGGATGAGATTTTAATTCTGAAAATG

TTTAGTGATTATCATTGCTAGACCTGTGATATAATTTTTGAGACATGGTATTGATTCATGAATATCATGT

AATGGTTTTGAAAATGGGATGGATACAAATCAAAGTGAAAACTGGAAATGGATCAGATATCTCTATTTGG

AGTTGCGTAAGAGGTTCCGTTATTTGATAACGACCCAGCATGGTGGCGTAAGAGGCTTGCGTGGTTTACA

CACCCAAAACCACCTGGTCCAGCGTCTAAAGACCTAGCTAGTCTTTAGGTTCCGG

>MSTRG.564.1 gene=MSTRG.564

CATTTATTCAGTCATTTTATGATGTTTTTGTTTCGTTTTATTAAAGCAAACCCAGTACCCAATTTCCCCT

GCTTTCGTTAAACACAAAACCCCTGTTTTGTGGTTATTTAATCAAACCCATTTCCTTTTCTTTTTAAAAC

ATTCAAACCCTCTTCGTTTTATCAAAATCTCACCTCTGTTTTCTTTGCATCAGTTAGCTTGTAGCGAGTC

GAGTAGCTGGATGCCTTGATTTCTTTTAAATTTCTTGCTGTGATTTATTTTGCCATCGACGAGTTCAAGA

TGCCGAGTTGCCTGCGGAGTCTGGTCTTAGTTTGACTCCTGTAGTTTTGTTAGAGTTCGATAGCTGAGTT

GTAGCTTTCTGTTCTGAGTTTTGTTTGCGTGATTTCTCTGTTGGTTGTGTTTTCTTTTACTGAGTTTAGC

AACGAGTTGTCACTGGACTTCACTGAATAGAATCGTCCGAGTTCACCGAGTTGTTTTTCTGCTGAGTCCG

AGTTTTGTTTTGTTGAGTCACTGAGTCGATGTTGTTAGAGTTTGTTTTCGTTGAATCCGAGTATGCTGTG

ATTTGAGCTTGCTGCGTGTATGCTTTCATTTGTTCAGTTTAGTTTTTTCTTTTTGTTATTTCTGCTGTTT

GTCGAGTTGCTTTATTGCCTTGCTGCGATTTGTTTGCTTCCTGTTGAGTTCTTGAGTTTGGGTAGGTTCT

GTATCATGTTGGTGTATTGAGTTGCTACCCCTTTTTCTCTTTTGTTTAGAAACCACTCGCACCTGCTTTG

TTTTTCTTTTGTTGCGTCGCAAAAACGAGTACTTGGAATTTCCGAGTTTAGGTGCTGT

>MSTRG.565.1 gene=MSTRG.565

ATTATACTCCCTCCATCCCATTTTAAGTGTCCACTTTGCAAATTTCACACATCTTAAGAAACAATTAATG

TAATGCTTTTATTATTATCTTCACACACCTATTCACCTTGGTTTTTATAAATATTAATTACGTATAGCAC

CACTCCTAGTGTGTATTTGACCTTTCTTCATATTGGAATTAGCTGGTTGTATTTAATACTATATTGAAAC

TAGTGAAAAATTGCATGGGTTAAAGAGTATGACACATATTTTGGGAATTTTTTTTTTGGCAAAGTGGACA

CTTAAAATGGGATGGAGGGAGTA

>MSTRG.566.1 gene=MSTRG.566

CTTGTCAGATCTTCTAGATAGATCAGAAGCTTCACAATTTGCTAAAGGATCGCGCGAAGCAAGCCAGGAC

ATTTCAGGTCATATCTTTGGGATGATGTCCACTATATTGTGCGAGGTGGATGAAATGGTGCGCTCTCTGC

CTACTCGTGAGGTCACGCAGCCCGTAGTGGATCAGGAACTGAGCAAGTTGGCTGCTGCCACTTTTCCAGA

CCCAGTCCAGGAGTTATTCCGGACTGATTATGTGCGTGCAGCGAAGGGATTACTTAGTCAAATCCTCAAG

AACAATGACAAAGTCATTGTAGAGGTCAGTCAGCAGCAGCAGTTAGAGGGGAATGGGAAGGAGCAGCAGC

AGCAGCCAGAGGGGCAGCCAGAGGGGAAGGGGAAGGAGGCTGCTAGCACAGAGAAAAATCCGTATGAAGA

TGACTATGATGTAAATGCGTTTTTATACCCTCTGTTAGATTAGCAGCAGTACTGGTGGTAATTTGGAATT

TCATACTTTTGGTTGATGGTTTAGTAAGCCAGTGAATATTCTAACTTTTGGTGGATGGATGAATTTCTAA

CTTTTGGTGGAT

>MSTRG.567.1 gene=MSTRG.567

AAGGATCGCGCGAAGCAAGCCAGGACATTTCAGGTCATATCTTTGGGATGATGTCCACTATATTGTGCGA

GGTGGATGAAATGGTGCGCTCTCTGCCTACTCGTGAGGTCACGCAGCCCGTAGTGGATCAGGAACTGAGC

AAGTTGGCTGCTGCCACTTTTCCAGACCCAGTCCAGGAGTTATTCCGGACTGATTATGTGCGTGCAGCGA

AGGGATTACTTAGTCAAATCCTCAAGAACAATGACAAAGTCATTGTAGAGGTCAGTCAGCAGCAGCAGTT

AGAGGGGAATGGGAAGGAG

>MSTRG.572.1 gene=MSTRG.572

TGTACGCTCGAAGACGTCAGGAAGTCGAAATGGTATTGCAAGGCGAATAAGGGATGAATCGAGTAAGCGA

AGCGATGTGGCGAATAGAGTACGGCTAGAGATGGTCTAGAAATTATGATATGGATAGATTGTGAAAGTGG

AAAGATGAGATAGAGATGTGAGACTGGAGTCAGATTTAAGAAAGGATTAAAGATGAATGGCTAGTCTATG

ATTGCGAGTAAAGCTAAGGCTAGGCGAGGAGTGTTCAGGAAAGTTGATGAGGTTGGTGCAAGCTAGAGCT

GTGCTACAGAGATTCAAGTGCAAGTCATAGCTGGAATAGTTGACAGTGATTCTTCTTATCGAAGATGATC

TGATTTGGTAAGAGATGTTGTGTAATAATAGTGGTAGATTCTAATTTCAATACTGTAACCTGGATGCGAT

CCTGTTGTTTTAGGGGG

>MSTRG.572.2 gene=MSTRG.572

ATTGGACCCCGCTTTATTAATATAAAGCTATTAAATACGTATTTTCATGTCATAAACGATCCAAAGGGGT

ACCAAGTATTTGTAAATATAAATAGACTTTTCTATAGCTGATTTTCACCGTAGAAATCATTTTCACCAGT

TTCTTTTCACAAATACCGAGAGAAACCGAGCGGTGTTCTTCGTGTTCTTCATAATCAAACGAGGATTTGG

AGGTGTTATTGGAATCCGATGGAGGTGTACGAATAGTCAAAACGAAGCTATCGACGCGTAGAATCATGTT

TAATCACCCGTTTGTATGCAGATTTATCAGGATTTGGACGAGACGTGTATGCCCGAAGACGTTCAGAAAG

TCACGTGGTGTTACAAAGCGAATAAGGGATGAATCGAGTAAGCGAAGCGAAGATGTACGGTAGATAGAGT

ATAGCCAGAGATGGTCTAGAGACTATGAGATGCATAGATTGTGAAAGTGGAAAGATGAGATTGAGATGTG

AGACTGGAGTCAGATTTAAGAAAGGATTAAAGATGAATGGCTAGTCTATGATTGCGAGTAAAGCTAAGGC

TAGGCGAGGAGTGTTCAGGAAAGTTGATGAGGTTGGTGCAAGCTAGAGCTGTGCTACAGAGATTCAAGTG

CAAGTCATAGCTGGAATAGTTGACAGTGATTCTTCTTATCGAAGATGATCTGATTTGGTAAGAGATGTTG

TGTAATAATAGTGGTAGATTCTAATTTCAATACTGTAACCTGGATGCGATCCTGTTGTTTTAG

>MSTRG.573.1 gene=MSTRG.573

CTTGCCGGATATTTAAACTCCGGCGGCGTTCTTAGCTTTGATCTAATTTTCTGGCTTGATTAATGATTTG

TTTTGAAATCCGAGACCATATACGAGTTCCTGGGATGCTATTTAATAAATCTGGAGTTGTTTGCTGAGTT

CTGGGCGCGGTGGCAACACCGGCGACTCGCCGGCGGCGAGTCTGGGGGCGGCGACGGCGAAAATGCCGTT

TAGCCCCCAAACTTTTGGAGGTGGTGAAGATTTTGTACTCTAGTTTCTTTTCTGGCAAATTGACCCCTGA

CACTTTTATAAGTTACACTTTACACCCTTGC

>MSTRG.574.1 gene=MSTRG.574

AAAAAAAAAGAAAAAAAAATAGAAAAAGCAAAATTGAATGGCGGTCGTGACTCACTTACACTGAGAAGCG

TCCCAACCTTAGACTTAGCAAGAAAAAAAAAGAAAAAAAAATAGAAAAAAATAGGAGAGGCATAGGAATT

CTTTAGTGACCTTAAATTGTAGTTGACTCTTTAGTAAAGAAGCGTTTAAGCCTTATTCTGATTAACGGCT

CATAGTGAGGACTTTGTGAAGTTTGATGGTCTTTGTTTTGTTTCACTAGAGAGCATGCTAGCACACACGA

TGCACTTCACACTAGTAGTGGAAGAGAACATGGATAAGATGTGAAAGAGATGAATGCCGAGA

>MSTRG.577.1 gene=MSTRG.577

TATCCGACAAACTCCGTCTCACCTAATTTAACAAATCAGTAGGCTGACAGCAAAATTTGCTTATATAGTT

TGATCTAATATTGCTTTTTCTCCGTCCTCTCTGATTTGAGTCACGATTAGAGGATCTGTTAACTACCATC

TGCTATACACTACTCTTCACCTGCCCTTTGACATGAAGTTGGTATCATACTAAAGTGGGGAGGGGTTCTG

TAAGTTAATATGGGAAGTATGAAGAATTATAAAGACCGCTTATTCCGTCCTTGCACGCTTTGACTGAGCT

TGTCTCATCCAGTCGATGCACTCCTCGGCAGACCCAGAAGGGTGAGCAAGCTGCCATTCAAGCAACTTTT

CTACCTGCAATATGATGGAATAATTTTAGCATACACAGTTGCTTAGATAGAGTCATTAGCATATATAAGG

TTGGAAAGTCCGCACCCATATATAGTTAATTTAGCAGTAAAAGAGGATGGCTTACCCATTCCCTCACAAG

AGGTCCTCCTGTCTTTAGCTGCAAAACGCTCATAATTTCCTTTCCGTTAACCAATGGCTTCACTTCCCAT

ATTTTGTCAAGACCTTCATTCAGTATCACAAAGAAGAG

>MSTRG.579.1 gene=MSTRG.579

ACAAGAATGAGAAGGTTGAGGAATGATGCTGAAAATATATTAGTCAATCTGGACAGAGGTTACCTGTTCA

ATCTGGATAATAGTTATGTGCTCAATTTGGACATCAAATACAAGCTTTATCCAGGCAGCATCTGCAATCT

GGGCAACTTGTTTCCACTCAATCTGGACAACGCGTTCGAGCTCCATATGCATAACAATCAAGACAAGGTC

CTTATTTTTGATATTTTTGGTATTGTTGGTTGTTATGGTTTATAAGTAGTAATGCTATCCGATCCGGTTT

GTTCTTTTGGATATCCACATTTTGAATATGTATTATATAATGAAACGTTGGG

>MSTRG.582.1 gene=MSTRG.582

ATCTACTTTTCCATGATACTAGTATCACTCAAGTTAAAGTGCGTTTAGATGATGCACAATGTAATGAATA

TTGCAAATTTGAGTATCCGCTTGACTTGCCCACTTGTTTGTGGAATTTGCAATCAAATGGTTTAATCTGT

TTGTCTAATATGTTTAATGAAAATGTGGGTTATAATCCCGACATTTATCTCTGGAATCCTCTTGTTCAGA

AATTTAGGAATGTGCCGGACTCTCCCTTTTCGATGTTTACATTTAGGGAGACTAAGTGGAATGCTTTAGC

TTTTGGGTTTTTGCCGGAAGTTAATGATTATGTTGTGGTACATGTTATCAAACCTATTTCGACGGCTGCA

CCCCCTTGCTACTTAATTTCTAATCCTGACATAGGTTATGAAGAATACCCACACTCAGTCAAGATTGGTG

TTTATAGTCTCAACAGTAATTCTTGGAAGGAAGTATGTCAGGATAAGGTATTTGTTGATTACATGAGTAC

GGATGTATCGGTATTTGTTGATGGTACTGCATTTTGGGTAGGCTATAATAATGAGGATTTGTGCCAGTTA

GTTATGTACTTTGATACGAAGACAAATGTACTGGGACAAGTCATTTTACCTGATGACATTATATTTCGTG

CATGTCAGCTTGAGAATCCACTTATTCTTCCATTTGGTCAATCGATTGCTTACTTTGTTGAGGTCGGTGA

GGATGATGCAAGCGAGGATGATGAGGACTATGGATCTCCTCATATGGACATATGGGTATTGAACAAAGAT

ACGATGGGTGAGTTTTCTTGGGAGGAAAAGATGAGTGTTACTTTAAGTGAAAATGTTTGGCCTGAAGTCT

TGGGTATAAGGAACAATGGTGAGCCAATACAAGCAAAATTGGACAAACTGATTTCATATGATCTTGATAC

TCATGAACCATATGTTTTTGTTGAATCATGTGAACATTTGACTCCAACTCCCTATTACAAGGAGGGCTCT

AAATCACCTTTTGTCATTCGTCCTTTCTTGGAAACTCTACTTTTTCTTGATATGGATTGAGAAATTTGAA

CGAAATGTTTGTCGGACCCTTGTTATCGCAATACGAACTTGCTAGAGTTCTTTAACTCTTCATTTTTCTA

GATAGAGAATTAGAGATGATGATGTAGACGTCATTATTTGTGCATCAAAAGTGGACCTCGAACTAAGATT

TCAACAAATGATTGCTAGTGACATGGCTCGTCTGAATTTAAGGAATCCAGTGTTGAATCAGGCAAGTGCT

TCCCATTTGACAACTTCATGGCAATTCCGGTTCCTATGGTATTAGCAGAACTCGAGTATAGCAGATAGAT

GTTATGGGGAAGGTTAAAAACAGATAGTAGCTATACTACCTCTGTTGAAGTTGTAAGCAACACAAAGGGA

AGCTAATTGTCTTAATTTCTTATGTTTTTGATAAAGTTCTAATGGAAGTGTGTTTATGATCAATAGCTAA

TGTAAGTGTACCCTATATCTTTTGTCATAGTAGTTCATCCTGTTAAAGAAAGAGAGTCATAGTTGTTGTT

ATAGTTACATAGGACTGGGTTCGATTCGAAACGGGGAACGAAATCGGGTACGTGGAACATTAGTTTTAGT

GAATCTGGTACGATGGAACGTATAGGTACGATGGAACGTATGGGTACGATTG

>MSTRG.585.1 gene=MSTRG.585

CTTGTAAACATTCTCTCGACTTCACCTCACAAACACTCTGAGCGACGAAAACAAGAGAGAGATAGATGGC

GAGATAAAGAGAAATGGCGATAAGAAGAAGATGAGAGAGAGTGAAGAAAGCACCCCGTAGAGGATACAAA

AGAGATTTGCATAAATTATGGAACAGATACAGGTAAGACAATTGTGACTTTGATGGAACATAATCGAATT

ATGGAACAGATACAGGTTAGACAATTCTGACTTTGATGGGTGATCCAAATGGAGAACAGAAACAGGTGAT

ATTCGTATGAATTATACTGAATATTATTGCTTTAGGCTGTTGCTTCTAGTTCCTTTGCTTTGATGTCAGT

ATATTTTTTAATCACTCGTATTTTTTCGTTGTAATGATGGGAATGGGTTAGCATTGTACAACTTGCAATA

AACCAAGGACTTTAAGTGATGTACAATATCAACATATATTCTGCTTTTAACATTCTTCTGCTTTTGAGGA

AGCTCTTGCCCTTGTAACTACAGACAAACTATTTGAAAGACTGGTTTAATTATCATCCACTTGTCGCATA

CCATGTCTTATATGGCGGAGCATGTGGTTGGTACTGGTTTATTTGGTGTTGTATATCAGGTGCGCAAAAT

GAGGGACTTTTTCCCAGGTTTCTAATAAATGGATAAGTTGAGTTTCAGGGTTTTTTTAGTAATGTCCGCT

TGG

>MSTRG.585.5 gene=MSTRG.585

CTTCGGTCGATATTTGATTTGTTAAGGTTTTAATGGCTGTTGAAGAAAGTTGTTGATATTCTGTCCAAGC

ATCTTGATCTTTCAGACCAATGCTAAATATTGAGAAAAGATTTGAGGAGGTGAAGAAAGAAAAATCCAGT

CCACAAGCAAAATTTTAAGAGGAAACACAAAAGTCTATATTCAAAAGAATTCAGCAGGCTCTGATGGATG

GAAAACGAGCTCGATCAGTTTGAAAAGGATGTTATAAAGCACATGTGTTTACTTGCTATATCCCTATGAT

GTGTGCTTGATTAGTAATACCTTTTAGCAAATTGGATGTTGAAAAATTGGTGTATTGATATTTTGTCTGT

TTTCTTGATTGTTCTAGACGTGTAGTTGGTAATAATCGTAGTATTGTTTTAGGATGTTCTTGTAATGAAC

CTTTGAAGGTGGATTAATGTATTTTCGAAATGGTCATGTCAAATTTTTAACAAG

>MSTRG.586.1 gene=MSTRG.586

ATTTTATATTCTAAACTTATACTTCATCTCTACCCATTCCCCACCAACCACCCCCACCCCAGCCCCACTC

ACCACCATTGTCGCACATCAGCACACCACCATCACGCACACCACTGCCACCGAACATCATATCGACAGAT

CTCTCTCTCCAGCAACACGGCAGGCCGCACTCTCTCTCGCACTGCAGCAGCAAGACAGGCTCACCGGCAT

CGCAGTGGTCGCCGACATCGCTACATCACCACGCGGAAACTATTATTTCTGATGTTTCCAACAATTCCGG

CGATTTTCCACTGCCTCATTCACACCAGGCCTAGCCTTTCCTCCGCGCGCATCACCATGGCGATTATATA

CATACACATCTGTTCAATCTCTTCCATCTCTCTCTTGTTCATTTTCAGCGATTTTACTTTGTAATTAGTG

ATTTTTCAGATTTGGTGGTTTGTAGATGGTGGTGGTTGTGATTATTGTAGTGATTTATACTTAAATATGG

TGATTTTTATTATGATGTTGGTGGTCGGAGTTGATGAATGGAGCTAGTCGCCGGATCTGGTGAGCATGAA

TAG

>MSTRG.588.1 gene=MSTRG.588

ATTTTTTTGAAATCGTCGGTGGGTTATTTTTTCCCGTCATTTCAGGAACTGTTAACTCCACGACTTTCCA

ATCCTGTGAGGCTGCTGGATTAATCATACTGCAGTTCTTCTCTATACGAATATAGATCATATTGGTGGCC

ATGGAAAATGGACATCATATCACCGTCACTGTTTGTATCGTTCCATCTATGGACCACGGAGTGCATTCTA

AGTGTTAGGAATTCTACTACTGCGCCCATTATTTCGTTGGATTCGCTTTCTGCTGATTCACAGGATTCAG

CATTTAATAGTTTTCATGGGAGAAAAAATTTCAACACCCAGGTTTAAGTCTGGTGTCACACAAACCATCA

GGTGCGATTGATAATGCTTAATTTCTCTGCAACACGTTCAAGTCAATTTTGTTGTAATATTTTACGCCAT

TGCAAAGGAGGCTCCACGGAGGTGATTGTCTGTTATATTTTAAAAACATCTAATGCATACATGATCCTTA

TATATACAAGCTCTACATGTGTTGCTTTTGTCTTCACAGCTACAAGGTCACCTTTTTCAGTTGTAAGGGA

CACAGTGAGGAAGACTACATCCCTTGCAAAGTTGCCTCCATTTCAATTACAATCCTCTACAATCTCTTCC

GGTTTTAAGTCTCATTCATCCGGCGAGTGTGATATTTTAAAGGACCCTATTTTAGGACATTTATATTTTT

ACTGTTTTGTTGATCATTTTTTTGCACATATTGTTTCAATTCATATTATTTAATATTAGGTATCATTGGC

CTATTGTGAGCCACGTATAAATTAGTTGTTAATGAAGAAAAGATTTTCATAAAGAAACTCTATTTTGTTG

CTGATGTGTGTAAAAGCAAATAAGTTAATGCGGGGTTCTAGGATTTCTATTGGTCTATTTTTTTAAACGC

AGATTCTTTGCTTTCTACAGTGAAACATATAAGTCCTGAC

>MSTRG.593.1 gene=MSTRG.593

CATCATCCGCCTCTTGTGCATCTCTTTCTCTGTATAAATATCTCTTTCTTTCCCATCATTTAATTCAAGA

TCTGAATCACAGACACCCCTTTTCACATTCTCTCTGGTTTCCACTCAGATATGTGCTTTTGCAGCTAGTT

CTTGCACTTAGATCCACTAAACCCACTTGAAAGGTACAAGCTTTTTCAATACCCAGATCATAATTTGTT

>MSTRG.595.1 gene=MSTRG.595

CTCCAATCTTTTTGGCCATTGTTCTGGCCACTGGCTACCATGTTCTGACACTTTTTCTGGTACTTTGTGT

ATGCATGCTTGAAGCTGCACATTCCAGACTGCATCTGGCTCGTCAGCTTTGTTGCAGAGTGGGGGTTCAT

TTTGTGATCTTTTCTCATAGCACTCATTCGATGATGGTTTTCGGTATATTGCTGCTCCTACTTCATTCAA

GTCATCACGGCTAATTTTTACTAGTTCCCAGCACATGGACTTCGTTAGTTCAGACATAGCTTTCCATATG

T

>MSTRG.601.1 gene=MSTRG.601

TCATGAAATAGTCCAAACTCCTTCCTTGCACATCCAGCCATTCAGTACAGCAATCATTGGGCAATACTAC

TATAGCAATCATTTGTATGATTACATTGATGATCGGTTGTTGCAGTTAATCAGGCATGTCAAACTTGGAT

TAATCTATTGTCCCATTCTGGCAGCATACTCCTTAATGCATGTAGTCAGCTCCGAGGCTCTCATTGCCTC

GGAGCTTCAAGTAACCACTATCAAGTTTCCAGTATTACTTGACCACATACATCTACTACTAGTCAGGGTT

GGAACTGTCATGTTTTTGTGAGTTTCAAAGGCAAGGATACTCGCAACCCCTTTACAGGCCATCTCATTGC

AGACAGACGAGGATGAATTACCTAGAGGTGAAGAAATATCACCACGGCTATGGAAAGCAATGCACGGATC

AAGTATTTGTATCTGTATACTTGTTTTCTCAAACAACTACGCTTCATCAAGCTTGTGGAAATTCTTGATT

GCCAGAAACATTGTGTGTCCTATATCCTATCATGTGAATCCTTTGCATTTACGTTATCAGAGGGGAAGTT

TCGCAGATATTTTTGCAAGGTGATATTCATATCGACAATGGAGAAGCAAAAGATAAAAACTTGGCAATTT

GCACTATCTGAAGCAGCTGATCTATCAGGATTCAAGTTTTTGACATCTATTTATCAATCTTAATTACTTC

AAATGCTTCACCAGAATTATTTTCT

>MSTRG.604.1 gene=MSTRG.604

CTTCATTTCCACTTAAATTGGTTGGTTGTACTTAAGTGATTAGTGTCTTTTGATGTGTTTTTAGTGTTTT

TCTGTGCAGGTCACGAGTTGAGGTGATGAAGTGATTTTCTATCATTTTAGGTTGTTTTTGGTGCATTATT

TGCAAGAATAGAGAATTGTGGATTCATCGCGACTTGGGATTTTGTGGGTATATCTTCTACAAACCCTCAC

GAGAGCACACTCGTCCACTAGAGCTATCTAGGGGTTTAAAGGGCTTGTTGCATGTGCTAAATGCAACCGT

GATCACCTACGGAAGTGGTACTAGAGCGAGGAAGGACATGCACGCGAGAACGAGCTGAAAACGAAGAATC

AGGGCTGCCAGGGCAGACAGTAGCGCGCCCGCGCTAACCTCTAGCGCGCCCGCGCTACCTACTGCTGCCA

CTAGCGCGCCTGCGCTAGTTTAGCGCGGTCGCGCCCAGCAGAAGTGCCCCGGGCCGATTTTTACAGCTTT

TCTGACGGATTTTAGCAGCGGTCGAGGCCCGGTTGACTTGGTCAATTTATTTTAATTTCTCGTGTGTAAA

ACCCTAGCCTCCAAG

>MSTRG.605.1 gene=MSTRG.605

AAAATAAAATAGAAAATGCTGCATTTTATTTAAATTTAAGCTTGGTTATGGTATAAACTTGCTGGATGTT

TCTGACTATATATATGTGTTTTTGGCAGTATGCATGCATCAGCAACCATGAGCTTAAGGACAGCCTTGTT

GTTACTAGCCTTGATGGTTTCAGTAACTTCATCTGTTCCAAAAGATAGAGGTGCATACATAGTTCACATG

TACAAGTCGAAGATCAAAAGTCTGCGGACAATAAAAAAATGGCATGAATATGTCATGGACTCTATCCATA

GATTGTCAATTCAAGAAAATAATGAAGAAGAAGCATATCCACCCAATCTTCTATACGTTTACGGAACAAC

CATATTTGCTTTTTCTGCAGCACTCACTAAATCAAACCTAAGATCATTAGACAAAATCAACGGTTTTCTT

TATGCCATTCCTGATGAAATGCTAAGCATCCACACCACTCATTCACCAGGTTTTCTCAGCCTAAATCCTG

GCAGGGGACTCTGACATCCCAAGAACTTGACTTCTGATGTCATAATCGGTATTCTAGACACTGAAATATG

GCCAGGACACATCAGTTTCTTCGACACTGGCATGCCTCCAGTGCCCTACAATTGGAAAGCCAAATGCGAA

GAGGGCACAAACTTTACAATTTCAGATTGTAACAGAAAGCTAATCGGAGCAAGAGCTTTCTTCAAGGGTT

ATGAATCTGTTAGAGGAACAGATTAATCAAACAGAGGATTGCAGATCTCCCCTTGATGCAGAAGGCCATG

GTACACATGCTGCATCAACTGCAGCTGGTAATCTTGTACCAGGTGCAAGCTTTCTTGGTAATGCTAAAGG

CGCAGCTACTGGAATGATGTTCACAGCAAGAATTGCAGCATATAAAGTTTGTTATGCTTTCGGTGGTTCA

AGCTTAGATAATTTAGCTGCCATGATACAGCTATTAGTGATGGGGTCAATATATTGTCACTCTCTTTGGG

TGGCATTGCAAAGCCTTATTATGAACATAGCATAGCTATTGCATCACTTGGTGCATTTCAACAAGGGGTC

TTTGTTTTATGCTCAGCTGGACAGGAGCTTTCCAACCACATTTAAACTCAGGAATGGTCAACTATTTAAA

GGGCCTTCATTGTGTTCCGGGAAAAATATTAAAAGCTTGCAACCTGTGTATGGTGCAGCTGCAGGTATAC

AAGGGGCTCAGTACTGTTCTAATGGCTCTCTTACACCGGAGCTTGTGAAGGGAAAGATTGTAGTTTGTCA

AAGAGGAGGGAGTAGCCGAGCCAAAAAAGCAGAACAGGTACTGTTTGCAGGGGGTGCTGCAATGCTGCTT

GTAAACACTGATAATGAAGGTGAGGACATCTTTGCTGATCCACATATAATCCCTGCAACACCTTTGGGAG

CTTTAGCTGCAAGTGCTTTAGATGCTCTATGTCGGTCAAGGGGCTTATTATCTTTTTTTGATTTGTTTGA

ATATGAGGATTTTATGAAAGCGGATTTATGGACTCTACCCCACTTAACAACGATCAATATTGAATATCGT

TCTTATAAAAGACTCAAGGTTCACAAATCATGTTGGAGGCTGCCTGTCTTGACAACTCTATGGTCTTTCC

TCAGAATATGATTGACTACTTTCTCGCACTTACCGGTCTAAGGAATCTTGTCATTGATTTTTGCCAACAC

TATATCAGACGTCTTGTCATTTGGTGAACCTAAAAATTAAGGTCAGCTTATCTTCTGTACATATACTTAG

GCTCAAAAATTGTGGTATGGCCACAAAAACTGTGTAATTTTCAAGCTATTGGTTTCTATCGGGTGTCTTA

TGAAGGTTCTAAGTTGGAGATTGCAGCTATTAAATTTTGGGATCTTCTATTAAGCTCGACGGTCTGGAGA

CACTATCAAGATGTGATTGAAACTATGTTCTCCCAGCTTGGCAGCGCCAAGATTCTTAGTATTGACTTGG

TGACCATTCAGGATAAAATATCTGGAAAATTTGAATATAAAATCCCATCAAAATTAGTCAAGAATGCAAC

AAAATGTTGAGATGAATGCTGAGGTGCTTTCTGAGTATCGGGCTCTTTTTACTGACTTGCAGGGATGGGG

ATTCAAGTTAAATTGGCTGATAAACCATCTCACCTTTATTGAACCGCTCTTGTTTTCAAAGACTAAACTT

AACGAACTTCATGCAATTGACTTTCACATTAATGATGCCAAACGTAAATTACACGATTTGCAAACTCTTC

ATGTCGAGAAAATGACACATTCAGAAAGCTCTCCAAGTAAGAGTACTAGCCTTGCGGTTATTTCTGGCTA

CCTTGGAGATGTTATACTGTAAGAACAAAAAAAAAACTTGACAGAATGTTACTATATATCTGTGTTTGAT

CCTCGGTAATTTCTATGTAATTTATGATATTTATTCAAGAATTTCAAGAACACTTTTCCTTTGGAGAGGG

TAATAGTTGATTTAAGTTATGCCTCCAGTCC

>MSTRG.605.2 gene=MSTRG.605

ATAAACTTGCTGGATGTTTCTGACTATATATATGTGTTTTTGGCAGTATGCATGCATCAGCAACCATGAG

CTTAAGGACAGCCTTGTTGTTACTAGCCTTGATGGTTTCAGTAACTTCATCTGTTCCAAAAGATAGAGGT

GCATACATAGTTCACATGTACAAGTCGAAGATCAAAAGTCTGCGGACAATAAAAAAATGGCATGAATATG

TCATGGACTCTATCCATAGATTGTCAATTCAAGAAAATAATGAAGAAGAAGCATATCCACCCAATCTTCT

ATACGTTTACGGAACAACCATATTTGCTTTTTCTGCAGCACTCACTAAATCAAACCTAAGATCATTAGAC

AAAATCAACGGTTTTCTTTATGCCATTCCTGATGAAATGCTAAGCATCCACACCACTCATTCACCAGGTT

TTCTCAGCCTAAATCCTGGCAGGGGACTCTGACATCCCAAGAACTTGACTTCTGATGTCATAATCGGTAT

TCTAGACACTGAAATATGGCCAGGACACATCAGTTTCTTCGACACTGGCATGCCTCCAGTGCCCTACAAT

TGGAAAGCCAAATGCGAAGAGGGCACAAACTTTACAATTTCAGATTGTAACAGAAAGCTAATCGGAGCAA

GAGCTTTCTTCAAGGGTTATGAATCTGTTAGAGGAACAGATTAATCAAACAGAGGATTGCAGATCTCCCC

TTGATGCAGAAGGCCATGGTACACATGCTGCATCAACTGCAGCTGGTAATCTTGTACCAGGTGCAAGCTT

TCTTGGTAATGCTAAAGGCGCAGCTACTGGAATGATGTTCACAGCAAGAATTGCAGCATATAAAGTTTGT

TATGCTTTCGGTGGTTCAAGCTTAGATAATTTAGCTGCCATGATACAGCTATTAGTGATGGGGTCAATAT

ATTGTCACTCTCTTTGGGTGGCATTGCAAAGCCTTATTATGAACATAGCATAGCTATTGCATCACTTGGT

GCATTTCAACAAGGGGTCTTTGTTTTATGCTCAGCTGGACAGGAGCTTTCCAACCACATTTAAACTCAGG

AATGGTCAACTATTTAAAGGGCCTTCATTGTGTTCCGGGAAAAATATTAAAAGCTTGCAACCTGTGTATG

GTGCAGCTGCAGGTATACAAGGGGCTCAGTACTGTTCTAATGGCTCTCTTACACCGGAGCTTGTGAAGGG

AAAGATTGTAGTTTGTCAAAGAGGAGGGAGTAGCCGAGCCAAAAAAGCAGAACAGGTACTGTTTGCAGGG

GGTGCTGCAATGCTGCTTGTAAACACTGATAATGAAGGTGAGGACATCTTTGCTGATCCACATATAATCC

CTGCAACACCTTTGGGAGCTTTAGCTGCAAGTGCTTTAGATGCTCTATGTCGGTCAAGGGGCTTATTATC

TTTTTTTGATTTGGTATCGACACTATTTGTTTGAATATGAGGATTTTATGAAAGCGGATTTATGGACTCT

ACCCCACTTAACAACGATCAATATTGAATATCGTTCTTATAAAAGACTCAAGGTTCACAAATCATGTTGG

AGGCTGCCTGTCTTGACAACTCTATGGTCTTTCCTCAGAATATGATTGACTACTTTCTCGCACTTACCGG

TCTAAGGAATCTTGTCATTGATTTTTGCCAACACTATATCAGACGTCTTGTCATTTGGTGAACCTAAAAA

TTAAGGTCAGCTTATCTTCTGTACATATACTTAGGCTCAAAAATTGTGGTATGGCCACAAAAACTGTGTA

ATTTTCAAGCTATTGGTTTCTATCGGGTGTCTTATGAAGGTTCTAAGTTGGAGATTGCAGCTATTAAATT

TTGGGATCTTCTATTAAGCTCGACGGTCTGGAGACACTATCAAGATGTGATTGAAACTATGTTCTCCCAG

CTTGGCAGCGCCAAGATTCTTAGTATTGACTTGGTGACCATTCAGGATAAAATATCTGGAAAATTTGAAT

ATAAAATCCCATCAAAATTAGTCAAGAATGCAACAAAATGTTGAGATGAATGCTGAGGTGCTTTCTGAGT

ATCGGGCTCTTTTTACTGACTTGCAGGGATGGGGATTCAAGTTAAATTGGCTGATAAACCATCTCACCTT

TATTGAACCGCTCTTGTTTTCAAAGACTAAACTTAACGAACTTCATGCAATTGACTTTCACATTAATGAT

GCCAAACGTAAATTACACGATTTGCAAACTCTTCATGTCGAGAAAATGACACATTCAGAAAGCTCTCCAA

GTAAGAGTACTAGCCTTGCGGTTATTTCTGGCTACCTTGGAGATGTTATACTGTAAGAACAAAAAAAAAA

CTTGACAGAATGTTACTATATATCTGTGTTTGATCCTCGGTAATTTCTATGTAATTTATGATATTTATTC

AAGAATTTCAAGAACACTTTTCCTTTGGAGAGGGTAATAGTTGATTTAAGTTATGCCTCCAGTCCAACAA

GTTATCAGTGGC

>MSTRG.606.1 gene=MSTRG.606

TTGAATGGTGTTGTTCATTGCTTGAGGACTTCAAGAAAGCTTGTTGTTTTCAAGGGAGTCGAGTAATTAA

AACTGAAGATCGATGGTCTCCTCCAAATAATGGTAGCATAATGTTGAATGTTGATGCTAGTTTCTTAAGT

TGTAACAATTCTGGAGGAGTTGGCGTGGTGGCTCGTGATCATGATGGTAAGGTTCTCGATGCATCCACTA

AGTTTATGTCATTTGTACCTAATGTGTTGTTGGCTGAACTAAATGCAATCAAGGAAGGGGTCATCTTGGC

TACTAGTAAAGGCTGGAACAAATTTTGTATAGTGTCTGATAGCCAAAATGCAATAGCTGCACTACACAAC

TTTCCGAGGTATTGCAGTGACTTAGATCCCCTCTTGTCAAGTATTATCAATCTTCTGCCTAGTATAGGTT

TTGAGGGTTTTATATTTAAGCCGAGAAACACTAATGGTGTGGCTCATAGTTTAGCCAATTATGCTTTGCA

TGCCAAAGTTAGTGCTCTTTGGGGTGGGGTTCTTCCCTTAGTTGCCGGTCAAGCTTTAGCACAAGACTGG

CCTACCTCTTTGTAACTAATATTTGTTTAATGAAGTTAATTTCCTTTCAAAAAAAAAAAAA

>MSTRG.607.1 gene=MSTRG.607

AGTCCTGTAAGTGAGTGAGGTTTCTCTCTAAGTTTCTAGACTTTTCAACTTCTATATTTCTTTCTCTCTC

TAAAACCCTTTCTCTCTCTAATAGTTTTCTGGGTTGCATGGCTGATTTAATAGAGGAGATAGTCTTTAAG

ACTAATAATCTTGTAGTTGATAATGAAGAAGAGTGGGTGATCAATGATGATATTGAAGATCCAAGCGAAA

AAGCTCTCGTGGGAAGAATTTTAGCGAAGAAACAATGTAATAGGAAGTTCATTCACCAAGTATTTGAAGG

GATGTGGAGAACTAAGGGAAAATGGGAGGTTAAAATACTAAAGCATGACAATAGAAGTACTTATGTTGGA

TTTACTTTTCAAGAAATTGAAGATATGAAATGGGTAAAAGAAAAAATGCCATGGAACTTTGGTGGGGGAC

TGTTGATACTAGAAGAGTGGCCAAAAAGCGGTAAGTGGCAAGATGCTAAACTAGATGGTGTTCCATGTTG

GATCAGAATGCGAGGCTTTCCTTGGAAAGTATTAAATCTCACTAATGCAAGGAAAATGGGCGGTTTAGCT

GGGGAGATTCTAGATCTGAGGTGGAAAGATGGTGCTAGAAATCTTCTACACAATTATGCAAGGGTAAGAA

TCAAATTTCCAATAAACAAATCTATTTTTGTGGGAAAATATATCTTGGTGGAAGGCAAAAAAGAATGGGT

GCAATTTAAATTTGAGAGGTTGCCCCTTCTTTGTTTTAAGTGCGGAATATGGGGCCATGATAGATCTGAA

TGTAATGAGCCATCAGTTAAGGTGGAAAACATTGATGGAAAATTATATGATTTATATGGTGGATGGCTAA

AGGAAGCTGATAGTAGAGAAAATTGTTTTTCAATCAATAGTAGTGCAGAGGAAGATGGGAGCAAGGAACA

AGTTCTTGAAACAAGGGGAATGTTGAATGCTAATAAGGGAGATGTAGTGAGAAAAGAGAACCAGATAAAA

GAAAAGTCCAAGGGAAATTTAGTGTTTACCGGAGAATCGCAGCTAACTGAGGAAAATACAAAAAATCAGA

TTGTCAAAACAAAGGATAACAACATGCGTGTGATGGATGCAGAGATGATAGCTGGTTCTGTTCAATCAGG

GGGATTGGATAAAATATTTAATTGTCAAGAGAGAGAGTCATTTGGGGAAGAAGAGGTCCTAAATAATGTA

GCAGAACAAAATAGAGAAAACATTATGCCGGAGAAAGTGGAAACTTCATCCACTTCTAAAGGTAGTGAAG

GAAAAAAAAGAAAAGGTGTAGTCAATACGGAAGAAAGTAAAGACCAGCCAAACAAAGAAGATCATGAATT

AAAGAAAAAGATTAAGAGCAAGTTTGAAGATGCCGGGCAAAGTAACAAGGAGGACCAGAATAAAAGTGAA

GAGCATGCATTTGTCATTGGTAAGCATGATCCAATTTTGCAGCAGAAATGTAATATTTCGATTAAGAAAA

TGGCTAGAGGAAGAATGCAGAGTCTTGCAAGAAAAGAAAGAGATGGCTCGAAACATCAACATGCATCTGA

AGGTAATAAGCAAATGGAATTAGTAATATACCCAGTTGATGATAATTCTTATATAGGAGTGGACTTTTCT

AAAGAATTAGATGGTTCTTCTCCTTTGTCAGAAATTGTATCGGCGTCCCTTGCTAGTCAAGGGCGCCGGG

AGCCATGAATTGCTTATGTTGGAATGTCCAGGGGGTTGGGAGAACCTGGACATTCAATATGTTAAAATCC

CATATTAAGAAATTTAATCCTGATTTGGTTTTTTTATCGGAGACGAAGGCATTGTATCATCAGGCTGAAG

TTGTAGCTCGTAATTTAAATTTTAATAATTTCTGGGTTGTTGAAAGAAAGGGACTTGGAGGAGGTTTGCT

TTTGATGTGGTCTGATAAATTGGCTGTAAAGGTCCTTAACTGGAATTTAGGGAACATAACAGCAGTGGTA

GCTGGTAAAGGATTCAGACCATGGGTCTTTACTGGTTTTTATGGGAATCCAGATCCCAAACAAAGAAAAC

ACTCATGGGAGCTACTTTGCAGAATTAGAAGAAGCATTAATGGTGCTTGGTTGGTTGTAGGGGATTTCAA

TGAGATTGCAAGTGCAAATGAGAAATATGGAGGGTGTGATAGAAATCCTGCAGCAATGCGTAGATTTAGG

GAAGCTATGGATAATTGCAAGCTCATTGATTTTTCGAATATCAAGAGTGAATTTACTTGGTACAAAGGGC

ATGGTCGCCATTTGGTAATGGAAAGACTTGATAGGTGCCTCTGCGATGATGAATGGTTAAATTTATTCCC

AGAGGTATCTACTTCCTTGTTGGAGTGGGGAGGATCAGATCATAGACCTATGCTAATTAATCTCTTCTCT

CAAAAGCAGAATGTCAAAATTGGGGCTAAAAAAAGAAACACCAGGTTTCATTTTGAGGAGGCATGGTGTG

ATGAGCCTGAATGCAAAAATATTGTTGAAAATCTTTGGGAGAATAGTGGCCATTGTAGAAATAGTTGGGA

GTTAAAGAAAAAGATCAGGAAGTGCGGAGATTCTCTATCTGATTGGAACAAAAGACAAAGAGCAGAATTT

GGAAGAGAGCTGAAAGAAGCTAGGCAAAGGCTTGCTGATTTATCAAAGGAAAATGATCCAATGTTATGGG

AATGTATAAAGAAAGAAGAAGACAGAGTGAAGGGCCTTATGCAGAAGGATGAGATATATTGGAGGCAAAG

AAGCCGCTCACTATGGCTTAAGTGGGGTGATAAAAATACAAAATATTTTCACCGTAAAGCAACAACGAGA

AGAAAGAAGAATGAGGTGAGAGGGATTACGGATGAAGCTGGAATTTGGCAAGAGGATGAGGAAGTGGTTC

ATCAGTGCTTTTATAAGTATTTCTCTAAATTATTCACATCTTCGCACCCAGATATCAACTCTATTGATGA

GGCTATTGATGAGTTAGAGGAAAAAATTTCAAGAGAAGATAATATGAAGCTGCTAAGTCCTTTCACTAGT

GATGATGTGCTAAATGCGGTAAAAGATATGAACCCAACCAAAGCTCCAGGTGAAGATGGGTTGCCAGCGT

TGTTTTATCAGAAATTTTGGACCTCCCTGAAGAATGAGATTTGGAATGTCAGAAATAGCTGGGTTCATGG

AAAGGTACTGCCTATGGGATCTTCATTGGTTGAATGGTGTTGTTCATTGCTTGAGGACTTCAAGAAAGCT

TGTTGTTTTCAAGGGAGTCGAGTAATTAAAACTGAAGAACGATGGTCTCCTCCAAATAATGGTAGCATAA

TGTTGAATGTTGATGCTAGTTTCTTAAGTTGTAACAATTCTGGAGGAGTTGGCGTGGTGGCTCGTGATCA

TGATGGTAAGGTTCTCGATGCATCCACTAAGTTTATGTCATTTGTACCTAATGTGTTGTTGGCTGAACTA

AATGCAATCAAGGAAGGGGTCATCTTGGCTACTAGTAAAGGCTGGAACAAATTTTGTATAGTGTCTGATA

GCCAAAATGCAATAGCTGCACTACACAACTTTCCGAGGTATTGCAGTGACTTAGATCCCCTCTTGTCAAG

TATTATCAATCTTCTGCCTAGTATAGGTTTTGAGGGTTTTATATTTAAGCCGAGAAACACTAATGGTGTG

GCTCATAGTTTAGCCAATTATGCTTTGCATGCCAAAGTTAGTGCTCTTTGGGGTGGGGTTCTTCCCTTAG

TTGCCGGTCAAGCTTTAGCACAAGACTGGCCTACCTCTTTGTAACTAATATTTGTTTAATGAAGTTAATT

TCCTTTCAAAAAAAAAAAAA

>MSTRG.609.1 gene=MSTRG.609

GAGAAACCGAGCTGCGTTCTTCGTGTTCTTCGTAATCAAACGAGGATTTGAAGGTGTTATTGGAATCCGA

TGGAGGTGTATGAAGAGTCAAAACGAAGCTATCGACGCGTAGAATCAAGTTTAATCATCCGTTTATATGC

AGATTCAACAGGATTTGGACGAGACGTGTACGCTTGAAGACGTCAGAAAGTCGGAATGGTATTGCAAAGC

GAATAAGGGATGAATCGAGTAAGCGAAGCGATGTGGCGAATAGAGTATAGCTAGAGATGGTCTAGAGATT

ACGAGATGCATAGATTGGGAAAGTGAAGAGATGAGATAGATGTGAGA

>MSTRG.612.3 gene=MSTRG.612

TGAATATCCGCTAAACATAATAATAATATTCTTTTTAACACGTTAGCTTTTGAAACTGCTGAATATGATC

CTGCTATTTATCGGCGACGGTGCCGTGGAAAACTCGTTCTTGGGAAGCCTCGGACCTAGGCGCTCTCTTC

TGGCTTCTGAGCCTCCTTTTGCTGATATCGACTGAGGATATGCTGCTGCATAGGTAAATACTAGTCACCT

AGGGCTTTGAAAGGAAGGAAAACGGGTCCTGGGAAATTTACTCATATCCAAGTTGAATTTGACAGAAGTG

GTCTCACTGCATGGTAGGAGAGACATTGTATGAGTTGACAGTTAACCGCGGAACTGAGGCCTGATGTATC

ATTCAATTTTATGGTCACATGATTAGATTTTTGCTTCTTCTTTTCCCTTAGCCAAGCTGATTACTTAATT

TTACGACTATGTATGTGAAACATCTCTAAGAGTTTTCACAGATGCATCCTTTTAACATGTCTT

>MSTRG.615.1 gene=MSTRG.615

CTCCGTTTACTCTCTCTTTTCCCTTCCTCTCTATGTATCCCTTTTTCTCTCTCTCTACTCTATTTCCATC

TCATTGCTCTCGTTTCTCTCGCTCATTGCTCTCTTCTTCGTTCTGTCTCGTGTTCTCTCCTGCCCCCTTT

TCTTTTCTCTGATTTTCAATCTCTCCTCCTCTTACTCTCTTTATCTTTTTAAAAACAAATATAAATTTAC

ACAATCAAAACAAGAAAAACTGAGATAACATCATAAGAGACAAATGTTGCTGACTTGAATAAAACGAAGG

AGTTCGTCGATAATGGGGATCGGAGCTTCATCACCGGCGAAGAGCGGCCGCGATTGTATCGCTAGCCGGC

GGACAGGATGAAGCATGGCGACAACAAGAATGACGAGAAGAGAATGTTGATGTTGAGCCATTTCATGTTG

TAGTAGAGATTCTTTAAATTTGATTTTTAGATTTGCTTTTGGTTGGTTGCTTTTGAGTTGATTTCTAGTT

TGTTATTTTATTTACTTTTTAGGTTTCAATGTTGATATTAGTATAGACCCGGAGGGGTTTTCATTTTTTT

CATTTGCAACCAGTTGTAACTTATTATACAACTAAATGTAATTTTCAAAAAAAA

>MSTRG.616.2 gene=MSTRG.616

ATATAGATCAATCATTGCATATACATACACACCGTCGATACATGCCTACATATATAATCTTCACGACACA

CACACAATACGGTTGCGCCAAAAGCGGTTAAATCGAAATGAACGTCGGAAATATCGCCTCCAGCCCGGCC

GACCAATCGCCGGAAAAAGGCAAACCCATCGGAGTATCCTCCACTCCGGCGAACAAATCGCCGGGAAGTG

ACGCGATCAAACTACCGCCTTCGCTTGCCGCGAAGAATGACGTTCCGATAAAATCGCCGTTGCCGCGGTT

GCAGACGCAGTCGGAGGCGACGGCGTCGGCGGGAGAAGATGCTCCGGCGCTTCCGCCGCATATAAGGAAT

GCTATGTTCCCGACTGCTGATCCTGGATCCGTTGTTAGCGGGGGAGGCATAAGTTTTCTCACTGGAAACC

GAAACGCAAAGTTCAGCTATGGTTATTCTAGTTTCAAGGGTAAAAGATCTTCAATGGAGGATTTCTATGA

AGCAAGTATATCTGAAGTTGATGGTCAGATGGTTGCCTGCTTTGGCGTCTTTGATGGTCATGGTGGCTCC

AGAACTGCAGAATACCTGAAGAACAATCTTTTCAAAAATCTAAGTAGCCATCCAGATTTTATTAAAGACA

CCAAGTCAGCTATTGGTTTAACGATCTGGATTGCAGTTGATTCATTTCGCCGGACAGATGCGGATTACCT

CAATGAAGAAAAAACCCAACAAAAAGATGCAGGATCAACTGCGGCAGCTGCTGTATTGGTGGGGGATCGG

TTGCTTGTAGCTAATGTGGGAGATTCTAGAGTTGTTGCATCTAGAGCCGGCTCAGCTATACCTCTATCCG

TTGATCACAAGCCCGATAGATCTGATGAACGTGAGAGGATTGAACAGGCTGGGGGTTTCATCATTTGGGC

AGGAACTTGGAGGGTTGGCGGTGTTCTTGCTGTTTCCCGTGCATTTGGAGATAAACTACTAAAGCCATAT

GTGGTGGCTGATCCAGAAATTCAGGAAGAAGAAATTGAGGGTTTTGATTTTTTAATCATTGCAAGTGATG

GACTTTGGAATGTCTTTTCGAATAAGGATGCTGTGGCTATGGTGCAGGATATTTCTGATGCAGAAGCAGC

ATCTAGAAAACTCATAGAAGAAGCTTATGCAAAAGGGAGCTCGGACAATATAACGTGTATGGTTGTCCGA

TTTGATGGCCAGTGAGAGATGCATAAAGGTCTTGTATTGCCCACCTTGTATCTTCTTGCATTTGAGATAA

ATTACTCTTCAAACTACAAATACCAAGGATCTTGCAGGCTTGAAGAACTGGAGTCTTAAAATGGAATCTT

GTGATTATTGTAATGAAGAAGCTTCAGTACATTCCGTGTTTTTTAGGCTAACTAGTAATCAACTTCTTGG

TGTTTGTATTATCACATGTCACATTTTTTATTGTTTCTTAATAGATGTATCCACACAAATGCTAAATTAT

GTTTCTGTAGTCTATATGTATCGGACTTCTCAAAAAGGTACAAGTATGAGTTGAGGATGAGAGCAAGTTT

GGAACTTGTGGGCCAGTGTCACTGCTGGCATGTTGTGTTTGTTGTTTCTAAACACATCTGACTTGTAATA

ATGTGTGCATATTTATAAAGGCTACTTGATTTGAACAA

>MSTRG.616.1 gene=MSTRG.616

ATATAGATCAATCATTGCATATACATACACACCGTCGATACATGCCTACATATATAATCTTCACGACACA

CACACAATACGGTTGCGCCAAAAGCGGTTAAATCGAAATGAACGTCGGAAATATCGCCTCCAGCCCGGCC

GACCAATCGCCGGAAAAAGGCAAACCCATCGGAGTATCCTCCACTCCGGCGAACAAATCGCCGGGAAGTG

ACGCGATCAAACTACCGCCTTCGCTTGCCGCGAAGAATGACGTTCCGATAAAATCGCCGTTGCCGCGGTT

GCAGACGCAGTCGGAGGCGACGGCGTCGGCGGGAGAAGATGCTCCGGCGCTTCCGCCGCATATAAGGAAT

GCTATGTTCCCGACTGCTGATCCTGGATCCGTTGTTAGCGGGGGAGGCATAAGTTTTCTCACTGGAAACC

GAAACGCAAAGTTCAGCTATGGTTATTCTAGTTTCAAGGGTAAAAGATCTTCAATGGAGGATTTCTATGA

AGCAAGTATATCTGAAGTTGATGGTCAGATGGTTGCCTGCTTTGGCGTCTTTGATGGTCATGGTGGCTCC

AGAACTGCAGAATACCTGAAGAACAATCTTTTCAAAAATCTAAGTAGCCATCCAGATTTTATTAAAGACA

CCAAGTCAGCTATTGTTGATTCATTTCGCCGGACAGATGCGGATTACCTCAATGAAGAAAAAACCCAACA

AAAAGATGCAGGATCAACTGCGGCAGCTGCTGTATTGGTGGGGGATCGGTTGCTTGTAGCTAATGTGGGA

GATTCTAGAGTTGTTGCATCTAGAGCCGGCTCAGCTATACCTCTATCCGTTGATCACAAGCCCGATAGAT

CTGATGAACGTGAGAGGATTGAACAGGCTGGGGGTTTCATCATTTGGGCAGGAACTTGGAGGGTTGGCGG

TGTTCTTGCTGTTTCCCGTGCATTTGGAGATAAACTACTAAAGCCATATGTGGTGGCTGATCCAGAAATT

CAGGAAGAAGAAATTGAGGGTTTTGATTTTTTAATCATTGCAAGTGATGGACTTTGGAATGTCTTTTCGA

ATAAGGATGCTGTGGCTATGGTGCAGGATATTTCTGATGCAGAAGCAGCATCTAGAAAACTCATAGAAGA

AGCTTATGCAAAAGGGAGCTCGGACAATATAACGTGTATGGTTGTCCGATTTGATGGCCAGTGAGAGATG

CATAAAGGTCTTGTATTGCCCACCTTGTATCTTCTTGCATTTGAGATAAATTACTCTTCAAACTACAAAT

ACCAAGGATCTTGCAGGCTTGAAGAACTGGAGTCTTAAAATGGAATCTTGTGATTATTGTAATGAAGAAG

CTTCAGTACATTCCGTGTTTTTTAGGCTAACTAGTAATCAACTTCTTGGTGTTTGTATTATCACATGTCA

CATTTTTTATTGTTTCTTAATAGATGTATCCACACAAATGCTAAATTATGTTTCTGTAGTCTATATGTAT

CGGACTTCTCAAAAAGGTACAAGTATGAGTTGAGGATGAGAGCAAGTTTGGAACTTGTGGGCCAGTGTCA

CTGCTGGCATGTTGTGTTTGTTGTTTCTAAACACATCTGACTTGTAATAATGTGTGCATATTTATAAAGG

CTACTTGATTTGAACAAGGTTCGTCGTTACTTGGCTACTAGCTAAAAGTTTATGCGTGGTAGCATAG

>MSTRG.616.3 gene=MSTRG.616

ATTGCATATACATACACACCGTCGATACATGCCTACATATATAATCTTCACGACACACACACAATACGGT

TGCGCCAAAAGCGGTTAAATCGAAATGAACGTCGGAAATATCGCCTCCAGCCCGGCCGACCAATCGCCGG

AAAAAGGCAAACCCATCGGAGTATCCTCCACTCCGGCGAACAAATCGCCGGGAAGTGACGCGATCAAACT

ACCGCCTTCGCTTGCCGCGAAGAATGACGTTCCGATAAAATCGCCGTTGCCGCGGTTGCAGACGCAGTCG

GAGGCGACGGCGTCGGCGGGAGAAGATGCTCCGGCGCTTCCGCCGCATATAAGGAATGCTATGTTCCCGA

CTGCTGATCCTGGATCCGTTGTTAGCGGGGGAGGCATAAGTTTTCTCACTGGAAACCGAAACGCAAAGTT

CAGCTATGGTTATTCTAGTTTCAAGGGTAAAAGATCTTCAATGGAGGATTTCTATGAAGCAAGTATATCT

GAAGTTGATGGTCAGATGGTTGCCTGCTTTGGCGTCTTTGATGGTCATGGTGGCTCCAGAACTGCAGAAT

ACCTGAAGAACAATCTTTTCAAAAATCTAAGTAGCCATCCAGATTTTATTAAAGACACCAAGTCAGCTAT

TGTTGATTCATTTCGCCGGACAGATGCGGATTACCTCAATGAAGAAAAAACCCAACAAAAAGATGCAGGA

TCAACTGCGGCAGCTGCTGTATTGGTGGGGGATCGGTTGCTTGTAGCTAATGTGGGAGATTCTAGAGTTG

TTGCATCTAGAGCCGGCTCAGCTATACCTCTATCCGTTGATCACAAGCCCGATAGATCTGATGAACGTGA

GAGGATTGAACAGGCTGGGGGTTTCATCATTTGGGCAGGAACTTGGAGGGTTGGCGGTGTTCTTGCTGTT

TCCCGTGCATTTGGAGATAAACTACTAAAGCCATATGTGGTGGCTGATCCAGAAATTCAGGAAGAAGAAA

TTGAGGGTTTTGATTTTTTAATCATTGCAAGTGATGGACTTTGGAATGTCTTTTCGAATAAGGATGCTGT

GGCTATGGTGCAGGATATTTCTGATGCAGAAGCAGCATCTAGAAAACTCATAGAAGAAGCTTATGCAAAA

GGGAGCTCGGACAATATAACGTGTATGGTTGTCCGATTTGATGGCCAGTGAGAGATGCATAAAGGCTTGA

AGAACTGGAGTCTTAAAATGGAATCTTGTGATTATTGTAATGAAGAAGCTTCAGTACATTCCGTGTTTTT

TAGGCTAACTAGTAATCAACTTCTTGGTGTTTGTATTATCACATGTCACATTTTTTATTGTTTCTTAATA

GATGTATCCACACAAATGCTAAATTATGTTTCTGTAGTCTATATGTATCGGACTTCTCAAAAAGGTACAA

GTATGAGTTGAGGATGAGAGCAAGTTTGGAACTTGTGGGCCAGTGTCACTGCTGGCATGTTGTGTTTGTT

GTTTCTAAACACATCTGACTTGTAATAATGTGTGCATATTTATAAAGGCTACTTGATTT

>MSTRG.617.2 gene=MSTRG.617

GTTGTCATTCATGACTAAAACTAAAACAATAATTTGTGACAACAATCTCTGGATTCATTATATTACATAA

ACAAGTAACGTATCTAATCTGCACATATTCTTTATATTCTCTCCGATTATTCTCCCCTCGAAATTAATCT

CCGACTCATAAGTATAGCATGTATGATTCAAATCCGAATAGCACCTTCGATTTTCTCGGACAGACCAAAC

ACAATTGTCATGTTGGGATGGATCCTGGTGTCAGAGAATCAGAGAAGCCTAACAGATTAAGCAACATGAG

TGGTTCGGGTGTTTGTTCAGGGACGGATCTGGTAAGAGGCCCGGGGCATCAGAAACCGTCTTTCAACAAG

GGTAGGAATTCTCCTCCGTCTCTGAGGGTTCAGGCCATTGCAAGAGGCCAGAAGGAGCTTATGGAGATGG

TCAAGGCCATGCCCGAGTCGACGTACGAGCTCTCACTAAAAGACCTCGTTGATCATCACCAGAATGGAAA

TGAGTCGGTAGAAATTGATCAACAGAAGCAAGAAGAGGAAGTAAAAATCGATGAAGAAGAGAGTAATGGT

AATAAAAACAAGGGTGGGGTGAGAGTAAGACAAGGAAGTGTCAGATTAAGACAAGGGAGTGTGAGGAAGC

AAGCAAGTCTCAAGAAAGTGGAAAAGAAGATGGCAAGAAATGGGAGCAATATAAGTGAGAGTGGCAACAA

AGGCTTGTTTTTGAAGATGGTATTTCCCATGCCATTTGGAGGAGGGACCAATAAGAAGGTTGTGAAAGCT

AAGACTAGTAGTACTTCTAAGACAACAGCAAAGGAGAATCCTTATGCTAAAGTGTGTTCAAGTAGTGTTG

GGAATAAGGATCCTAGTATTGGTGATAAATCTTCCAAGAGTAGTAAGCATAAGGAGTGGTGGAAGAGGAG

GTATCCGGTGGCGTCGGACGGGAGTGAGAGCAGTGGATTGAGTAGCAATGGGAGCAGTGGGAGCAGTGGG

AGCTCGGCCAGCAATGACAGCAGTGGAAGCAGCATTAGCAACAAGAGCATCAGGTTTTTTCACTCAATTT

ACTCTGTTTTTCTTTTTCTTCTTCTTCTTTGGTGTCAAAGCTGCTAGAATTGTATTTTTCATGATTTTGA

TTCATTTTACAAGCTCTTAGATTGGTAATAGTCAGATGTTATTTAGAATTTAAGTTCTGAGATTTCACAA

AATACTGGAAAGTCCAAAGGGACGGAATCCTGATTTCTTAAAATTCTGATTTCGATTCTCCCTCGCATGC

TTTGCAATGTGAAACGGAAATCTATAGTCCACAGATATCACCCTCCGGAAGACAAGAGACGTTTAGCATG

CAAGATACTGTTAATAAATATATATTCAGATTTCAATATTGAAATGTTTTAGTTATGTAGTAATATTTTC

TCATAAAAATGACAGTGACTCGTATTCAAAACGGATGAGTTACAGATAATCATGAGTCAGAGATGCTGAG

CTTAAAAAAACAGATTTCTAGAAGGAATATCGAGTTTTAAAATTTAAAGCAAAAAAACGTTTATGTATAT

CTACACGCAAACGTTACAACCCATGCAACTTTAACACAG

>MSTRG.617.1 gene=MSTRG.617

GTTGTCATTCATGACTAAAACTAAAACAATAATTTGTGACAACAATCTCTGGATTCATTATATTACATAA

ACAAGTAACGTATCTAATCTGCACATATTCTTTATATTCTCTCCGATTATTCTCCCCTCGAAATTAATCT

CCGACTCATAAGTATAGCATGTATGATTCAAATCCGAATAGCACCTTCGATTTTCTCGGACAGACCAAAC

ACAATTGTCATGTTGGGATGGATCCTGGTGTCAGAGAATCAGAGAAGCCTAACAGATTAAGCAACATGAG

TGGTTCGGGTGTTTGTTCAGGGACGGATCTGGTAAGAGGCCCGGGGCATCAGAAACCGTCTTTCAACAAG

GGTAGGAATTCTCCTCCGTCTCTGAGGGTTCAGGCCATTGCAAGAGGCCAGAAGGAGCTTATGGAGATGG

TCAAGGCCATGCCCGAGTCGACGTACGAGCTCTCACTAAAAGACCTCGTTGATCATCACCAGAATGGAAA

TGAGTCGGTAGAAATTGATCAACAGAAGCAAGAAGAGGAAGTAAAAATCGATGAAGAAGAGAGTAATGGT

AATAAAAACAAGGGTGGGGTGAGAGTAAGACAAGGAAGTGTCAGATTAAGACAAGGGAGTGTGAGGAAGC

AAGCAAGTCTCAAGAAAGTGGAAAAGAAGATGGCAAGAAATGGGAGCAATATAAGTGAGAGTGGCAACAA

AGGCTTGTTTTTGAAGATGGTATTTCCCATGCCATTTGGAGGAGGGACCAATAAGAAGGTTGTGAAAGCT

AAGACTAGTAGTACTTCTAAGACAACAGCAAAGGAGAATCCTTATGCTAAAGTGTGTTCAAGTAGTGTTG

GGAATAAGGATCCTAGTATTGGTGATAAATCTTCCAAGAGTAGTAAGCATAAGGAGTGGTGGAAGAGGAG

GTATCCGGTGGCGTCGGACGGGAGTGAGAGCAGTGGATTGAGTAGCAATGGGAGCAGTGGGAGCAGTGGG

AGCTCGGCCAGCAATGACAGCAGTGGAAGCAGCATTAGCAACAAGAGCATCAGGAAAAAAGGTGGATTGC

TAAAGGGATGCGCACCCTTCTATTACAACAAGAACAAAACAATAGTGGAGTGAGTTGCGTGTTTGAAAGC

TGCCCAGCTCTGCAATTCATACTTTCTGTATACAGATTCAGCTTCATGTATAATGCCCCCATCTCAATTA

TATGATACATTTTTATACATGGTATTGATAGTTTCGATTTTGTGTAATTTTTGTTTTTGATCTTGTCATT

CTTTTTGTTGTGTTAGAAAATAAATTGATTTTGTTTTGTCTCTAAA

>MSTRG.621.1 gene=MSTRG.621

GTAATACTGAATGTTGTTCATATTGTCATCTCATTTAACTTTTGTTAATATTATATGGCCTTCTAGTCTT

CTGACAGCTTTCCATGGTATCGAGACTCAAGAATTATCATTTCACAGAGTCGATTGGTTATTGATTAGAG

AACAATAATAAAATTTTGGCATATCAATATGCCACTATAGGATCACTACATGACGTATTACACGGGAGGA

AGGGTGTACAAGGGGCTGAACCCGGTCCTGTTCATAGTTGGGTCGAAAGAGTTAAAATTGCTTATGGTGC

AGCGAGAGGCCTTGAGTTTCTACATGAAAAGGTTCAGCCTCCAATTGTACATCGTGATGTCAGATCCAGC

AACGTCCTTCTGTCTGATGATTTTGTACGTAGGATAATGTTGACTTCTCTATTCTGCACATAATAAAACC

ATCCCCTAATTTATTCATATGTGTTTTTGTAGAAGCCTATGATAG

>MSTRG.624.1 gene=MSTRG.624

AATCGCTTATTTATTTTATTTCAGGAAATTGTAGATAAATAAAGAAGGAAGAGAAAATGCAAGAAAAAGA

AGAAAAGGAAAAAGAAAAAGAAAAAAAAAAAGAGTTGGCAACTAAGGGACACATGGAGGGCCACAATCTT

CCCAACACACAAGGCACATGGATGGTCAAGATGGAGCCACCCTACCCCTAAACCCTAATTCTTTAGTTAT

AAATACCCCTCTTCTTTTACTTGTAATCACCAACCTACCAACCTAGTAACCTAGCCACCTACCCACCCTA

CCCCTAAACCCTAATTCTTTAGTTATAAATACCCCTCTTCTTTTACTTGTAATCACCAACCTACCAACCT

AGTAACCTAGCCACCTACCCACATTTTCTACCTAGTCATTTCTATAGTTAGTATCTTAGATAGATTTATA

TTTTGCTAGCCCC

>MSTRG.625.1 gene=MSTRG.625

GAAAAAAGCAATGGTAACTTTTTAGGTTTAATTGAAATGTTAGCTGAGTATGACCCAATTATCCAAGAAC

ATGTTAGTCGTATCACTAATGATGATATTTATATTCATTATCTAGTTAACTCAATTCAAAATGTGTTGAT

AATTTTGCTTGGTTCTGCAATTAAAAGTGAAATCATTAGAAGAGTAAAACGTGCAAAATATTTCTCTGTG

ATACTTGATTGTACACCCGATGTAAGTCACCAAGAGCAAATGTCTTTGATATTAAGATATGTGAATGTCT

CATCGAATCCTATTACTGTTGAAGAATCTTTTTTAGGATTTTTTGGATGTGAATGATACAACTGGACAAG

GACTTTTTGATGTTTTACAAAATGAATTAAAAATGCTTATACTTGATGTGTGAAGACAAGGTTATGATAA

TGGTTCAAATATGAAAGGAAAACATCAAGGAGTACAAAAAAAATTATTGGAAATTAATTCTACAGCTTTT

TATACTCCTTGAGGTTGTCATAGTCTAAACTTGACATTATGTGATACGGCTAATACATGTGGTAAAGCTA

AAGATATTTTTGGAGTTATAGAACGCATATACACAATTTTTTCATGTTCTACAAAAAGATGGTAAATTTT

GAAAGAAAATGTGGGATTGACTTTAAAGTCATTATCATCTACTCGTTGTGAGATTCGTGTTGAGAGTGTC

AAAGCTATAAGATTTCGAATGTTAGATATACGAGAAGCTTTGCTTCAAGTAGCGGATATTGATAATGATT

CAAAAGTGAAGAGTGAATCTAAATCGTTAGCAATGAATGAGCTTGGTGATTTTGAATTTTTAGTTGCAAT

TATTATTTGGTATGAAATATTACATGCTGTTAATATTGTGAGTAAATTTTTGCAATCAAAGGACATGCTT

GTTGATGTAGCTATTGAAAAAATAAAAGGGTTAGTATCATTTTTTGAGGACTATAGAGAAACTGGTTTTA

ACGATGCTTTAAATAGTGCAAAAGAACTTGCTACTGAAAGAATGTTGATCCTGTATTTCCCCAAAAGCGT

GAAATTCGAAGGAAAAGGTTCTTTGATGAAAATTTGGACTCATCATCAAGTGCTCCATTATCCGCAGAGG

AAAAATTTAGAGTTGATTATTTTCTTAACATAGTTGATCAAGCGCTTAGTTCTCTAAATAGAAGATTTGA

GGAATACAAAAATTATGAAAATATTTTTGGGTTCTTGTTTACTTATAAAAAGTTTAAGTCATTGGATGAT

AAGAGTTTGAAAAATTCATGTGTTCAAATAGAAAATGCACTAAAAAATGATGAGTTATCTGATATTGATG

GGAATGACTTATATATGGAATTGAAATTGCTTAGAGACTTTCTTCCTGCTGACATTGTAGGAGCTACTAA

TGTTTTAAAATATTTGAAAGATTTGATTGTTTCCCCAATGGGTTTATTGCTTATAGAATTTTATTGACTA

TTCTTGTGACTGTTGTATCTGCAGAGAGGAGTTTTTCAAAACTGAAGTTGCTGAAGACTTACATGCGCTC

CACAATGCTACAGGAAAGACTTAATGAATTAGCGATGATAGCAATTGAAAGTGATCTCTTACAATATGTA

GAATATGAATATTTAGTTGATGATTTTGCATCGAAAAGTTTTCGAAGGATGTCACTTTTCAAATAAAATA

TGTAAACTATTGTGAAGTTATGATCAATGTAAT

>MSTRG.628.1 gene=MSTRG.628

GATATCTGAGACGAGCATCCGTCCACTGAAAGAAGTTCATTAATATGTTCAAGCCTCAGTGAAGAATAAA

GTTCAATATGTTTCTGCTATCAAGATTGCCTGAAGATCAAGTATCAAAGACCAGAAGATTCCAGTATATT

TAATTTGATTATTTATAATCAAATTTATCGAAGCAACATCTAACCCGGAATGACTGATCAAGTATATTAT

CAAGCAAAGGATTCAAAGAATTATTTCAGTTCGAGTCGTTCGTGAACCAGACCAGTGCACAGGACGTCAG

GACATACGAAAGTATTCAGTGGATTCGATCAACGAATTAATTGATCAAGTCAATCGAGCTACTCCAAAAG

TTATTGTCAAAGAACTCATTGATATGTATATGTATATATATATATTAATTGTGAATTACCTGAATCTAAA

TAATTCAAGTAATTAAATTAACACAATTAATAATATATATATGTATATATATTAGTGCCAATGG

>MSTRG.630.1 gene=MSTRG.630

TGCAAGTCAAGTGGGTGGCGGTTATTGACGGTTTTCGGAAGTTTTATTGAAGGGAAACTGGCAAAGGTCA

AGGCTCCAATTGGCAAGCACAAGCAGGGCAGTTATGGGCAGTTGAGAGCTTCATGGGGCAGTTTTATAAC

TGCCCGGCAGTTAGAAAACTGCCAAGTTGTGGCTGATTCATATTAATGTTAGGCATTTCAGATGTAAACA

TAGAGAGAGAGAGAGAGAGAGTAGAGTTCTATATTGAGGGAATATAGACTAGAGATAGATATATTGTGGT

GTATATCTAGCCTAAGAGAAGAAATTTGGGAGAGTAAAGTGTGTAAACTCTTGCCATAACTCTTGGGTTT

TGTGGGTGTCCTTTGTAATTGTGTGATGCAATAATATAGGTTGGCTTCGGCTATAAATCTGTG

>MSTRG.634.1 gene=MSTRG.634

AAAAGTTACCTGCAGCATTGTATAGTGAGCAGCAGAGGTGATGGGCAGGGGACCAATTGTGGCAGAGTCC

ACTTCACCAGTCGGCAATGGCACTGCCGGGTTGCTGTTTATAAATGGTATGCCTCCACCGGGACTTGAGG

CCGGAAGTAGTGCAGTGGTAGGTGCAGGACCCGGGGATGTCGAATCGCGGGGACGAAAAGGCCATTGGTA

CGATGGGGAGGACGATACTGCCCCACCGGGACTCAGGGGAGGAGATGCCAAGGGTGAGGAGACTTGAGGC

TGGAAAATGGATACTTTGATGGCAAGTTTTTGGCCTTGTTCACATGGTTTTGTGGTGCCATTGTTGAAGG

AGAAATAGAAGTACCCGAGTCTAGATGGAT

>MSTRG.635.1 gene=MSTRG.635

AAGTAAAGTTTCAAGGGTTTGTCTAGCGTGTGCCCATGGGCACATGCTAAGCACTAAAATCTATGCATTT

GGAGGGTTTTGATTGGTGTGGTTGTTGTAAATTCAGGGGGGTCCACCATTATTAGAGAGTGTAAGCCAAT

CAAAAGCTTCCAAATTCATAGATTTTGGCACTTAATGTGTGCCCATGGGCACACCATAGAAAAACCGA

>MSTRG.636.1 gene=MSTRG.636

TCACTTTTGTTCTTTAATTCATCGAGGTTTAGGGCTTCTCTTAAGTGTCAATGGAACAAGGTAACGATTC

AAGTTCAACTCCGAGTGTATCATCAAGTTTGGTTGGTATGATATCAACGAAGTGTGGTGAAAATATCTCT

AAAAGCGCTGATCTGGATTGTGTAATAATTTCGCAAGAAGATAATCTTATTATTGGGCGTGATCAAGCTA

TTCAAGGAAAAGTGCAACCAATCAAAATATAGAAGACCTTGAAGTCATAAGTTTGGAATCATTTTGATCG

GATTGAAAGCGAAATAAAAAGTACAAAAAAATTTGATGAAATTTGTAAGTATTGTCACACTACATTTAAT

GGGGCAAGTGATCAGGGGACGACACATTTAAAAAATCACACAAAAACTTGGCGGATGAAATTAATAAAAG

TTCCTATATCACAAATGTTGTTGGGAAAGCAGGTTAGTGGTGAGGATTCTCGACCAAAAGTAGGAGTTCA

TAAGTTTGATAATGCTTCTAATAATTTAGGGATGCTCTTAGGTATAAAAATTTGTTCAAATGGAAAAAGA

GCACTTGTTCACAATGGTGACATGTTCCATATGCGTTGTGTGCCAAATATTCTTAATTTGATTGTTAAGA

GTGGGTTAGAGATGCTTGATCATTTAGTTGAAAAAATTCAGAAAACTGCAAAATATATAGGATATTCTTC

CCAAAGGGATGAGAAGTTTACCCCTGCTTTGTCTCAAACCAAACTTAATACTAAGAGACGAATCCCTTTT

AACCAATCAATACGACCTCGTAGAGCAGCTTTGATGTTGACACTCGTTGGAACTTAACATATGAAATGAT

TGCGGCATTAATTGAACTCCAACCAGCCATTTGATCGTCTAAATGAATTGGATCCTGATTTTAAATGTTT

CCCTTCAGAACAAGGATGGGAAAATGGAAAGAAAGTGTTTGATTGCTTAAAGATTTTTTCAGATATTACG

AAAAAACATCCTGGTGTGAAATATCCAACTGCAAATCTGTACTTTATTGATGTGATTCAAATTTGTAGAA

GTATTAAAATGTGGGCTGAATCTAGTGATGATTGGATTATTAGTGCAATGGGTAATAAAATGCAACTTAA

ATTTGATAAGTATTGGGGAAAATGCAACAAGTTGTTGACTGTCGCTGTTATTCTTGATCCTAGATATAAA

ATGTCCATCGTCTCTTATGCTTATAAAGGTATCTTTGATATTCATGCTGACTTTCAGGTTAATAAAAATA

CGTGAGTTATTGCTAGAGATTTTTAATGAATATTCTTAGAAATTTGCAAGTAGTAGAGGTTTTGTAGTAA

ATTCTGGTAATGCAAGTTTTGGTTGTTCAAGTTCAGTTGGTGGGGAGTGGTTGAGAGGTTTTCAGGATTT

CATTGCAAGTAGCAATTTGAGGGAAAATACAAGAAAAAGTGAGCTGGATGAATATTTAGAAGAAGGGTTG

TTTTCGATGGATAAAGAAGTGGAGTTTGACATTCTTCATTGGTGGAAACTTCATGGACCTATATTTCAAA

TCTTTGCCCGGATGGCACGAGATATTAGCTATACCTACTTCTTCTGTTGCCTCAGAAAATTCTTTTAGTA

AGTGTCGTAGAATTATAACGGACACTCGGTCTTCTCTAAATGCTGACTCAATTGAAACTTTGATGTGTGT

GAAGGATTGGCTCCCCGAACTCAAGGATGGTAATCAATCTGGAAAAACTTCAGAGGATGATGTTTTTAAT

GATTTGGATAATGAATGGGATTTGGACTTCTAAAAGAATCAGGACTGTGAATCTAATTTAGCAGCTGCAA

GTGGTATTTCACACTCTGTA

>MSTRG.636.2 gene=MSTRG.636

TCACTTTTGTTCTTTAATTCATCGAGGTTTAGGGCTTCTCTTAAGTGTCAATGGAACAAGGTAACGATTC

AAGTTCAACTCCGAGTGTATCATCAAGTTTGGTTGGTATGATATCAACGAAGTGTGGTGAAAATATCTCT

AAAAGCGCTGATCTGGATTGTGTAATAATTTCGCAAGAAGATAATCTTATTATTGGGCGTGATCAAGCTA

TTCAAGGAAAAGTGCAACCAATCAAAATATAGAAGACCTTGAAGTCATAAGTTTGGAATCATTTTGATCG

GATTGAAAGCGAAATAAAAAGTAATCAATCTGGAAAAACTTCAGAGGATGATGTTTTTAATGATTTGGAT

AATGAATGGGATTTGGACTTCTAAAAGAATCAGGACTGTGAATCTAATTTAGCAGCTGCAAGTGGTATTT

CA

>MSTRG.638.1 gene=MSTRG.638

CTTTTTAAGAGAATATTTCTGTTCCTATACATGCAGAATTGAAATGGCTAGTGATATGGAATCAAATAAT

TCATCAGTAGAAAGAGGCGCTGGAAAAAATAAAAGGAAATGGACTGAAGATGAAGATGGAAAACTAGTCG

AAGCTTTGATGAAGACGCTAAAAAAGGATTTCGATGTTGTGTACGACATCTGCTATGGAGCAAACAGTTC

GGGGTTTGGATGGAACGCAGAAGACAATGTTCTTACGGCACCGAGGGATGTCTGGGTTCAATATCTTAAG

GTTCATCCTGGAGCAGCTAAATGACAGAATACTGCCATGCCATCATTCAAAGAACTTTCAGTTATTTTGG

GAAAAGATAGAGCAACCGGAAATATGGTTGAAAATCTTGAAGATGTGGTAGGAGAATTGAACGCTGAAGA

TGCTGATGAATTATCTCTTTAGGAGGACCTGCAACGAAGCACACATTCAGACGAGTCAACAAGCAAGAAA

AGGAAGATAGGAAATGTAGAATCATTGTTGGAAGCTGTGTATGCTGCTTCAGACAGGATTTCCAATCAGT

TTGAGGCGTCAACCAAGTTACTTATTGCAACAGAAGAAGATATGATGCAGAAAAAGAAGCAACTAAATGA

CGAACTATCAAAAATTCCAAATCTCACAGTATTGCAGAAGCTTCAAGTTCCAAAGAAAATAGCCAAGGAC

GAAG

>MSTRG.639.1 gene=MSTRG.639

AAATCTTTGTGTCGATGCAAGTAGATTAGAGACAACTGTATCTTGATTATTAGTAGATGAGTCTTTAAGT

CGTATTTTCCTTAAAAACGCTTTTTGTTAACACCAACGAAATTAGAGTAGAGTCGAGTAGACACTTGCTA

GAATGTCTCGAAGATTTTATGGGTATATCTTCTGTAAACCCTTAGGAGACAAAACTCCTCTTGTAGAGAT

CGTCTACGAGTTTAACGGGTTGGTGAACCTAAAATCACCCGTCACGCCTACGAAAAGGAGCCTAGGAATC

GCATTTAGTTTTCTTAGTTTATTTCTTTTCTTTTGATTTTTACTCGAGGACGAGCAAAAGATAGGTATGG

GGAAGTTTGATTAGTCCATATTTTTGCATATTTAAGTGC

>MSTRG.640.1 gene=MSTRG.640

TGATTGGACTATAAATAGAAAGTGTAACATGTTGAAAAGTAGCAACACAGAAACTTAAACAGAAAAGATA

TAGGAATTAGGAAGCATGAAAGTTCTGAATCGAAACATGCTAATGCTTGTGTTAGTTTTGTCCTTGATAT

GTAATATATCTCACTTGTATGCTGCTGATCCTACTGATGGATTCACCAACATACCACTGACTGAAGCCAA

TTTCAAAATTCAGAGGCCTTATAATTTACCAGTCGAGGCACGATACAGCTTTGAAAATGGCGTTCGCAGG

ATGTGGGTGTACGCTAATGACAAGTCCCACAGCCCAAATAGCCAGACACAACCCCGCACTGAAGTTCGAA

TTATGGGACATGACTACTCATCTGGAGTATGGCAGTTTGAAGGATATGGTTTCGTCCCAAATGGAACCAC

TGGACCTTCAGTTGTGCAGATACATGGTGCCACCCACGAATCGTCTACAATAATTTTAAGGATCTATGAC

GGGGAGCTGAGGTACTATAGTGGTGATCTTATAGCCTCCAATATGTACGATAAGTGGTTCAAGGTTAACT

TAATCCATGATGTGGATCAAGGGAAAGTCACCGTTTTCATTGATAACCAACAAAAGTTTCAGACCAAGGA

CAGAGGGCCTGGAGACCTGTATTTCAAGTGTGGAGTTTATGCTGCTCCCAAAAACATCAGTTACTATATG

GAATCAAGGTGGAGAGACATCAAATTATACAAGAAGTGAACTGCTTAATAGCGAAAACTGTTGAAAATAT

AACCATTGGTGTTTGATATTTGTATCATGTATTTAATCTATCTACAAATCAAGGTTGGTGTGTGAGTATA

GACTTTCGATTTCTGATCAACTTTGGC

>MSTRG.641.1 gene=MSTRG.641

TTTTATTTTGTGCTGAAGCGTTCTCTTCTCTTTTGTATAAAGCAGAGATGGATAAAAAAAAATAAATAGT

TTAAAGTTTGGCAAAAATAGTGTTATGGTGTCCCATTTATTTTTTTGCAGATGATAGATAGCCTGATATT

TATGGAAGGTAACGAGGAGGCATGTAACAATTTCAAGAAAATTGTTGAGAAGTACACTAAAGCATCAGGG

CAATGTATAAATTTTAGCAAGTCTAAATTGTGTGCTGGAAAACTTGTCGGGATTGAAATGCAAGAAATTT

GGCTAAAATTTTGGGGGTGAGGTTAGTTAGACATTTTGAAAAATATTTAGGTTTGCCGACACATGTGGGG

AGAAGTAAGAATGATGTGTTTAGATTTATTAAAGAAAGAGTGTGGAATAAACTCAAAGGATGGAAATCTC

ATCTATTTTCTGTTGCCGGTAGAGAGATACTTATCAAGGCGATCATACAAGCTGTTCCAGTATTCACTAT

GAGCTGTTTTAGAATTCATAAAGGAATTGTTCAAAAAGTTCACAGTATGATGGCTCGATGCTGGTGGGGT

TCAAATGATAAAAAGAGAAGAATTCACTGGTGCAGATGGAATTATTTGTGTGAGCCAAAGGAAAGAGGAG

GATTAGGGTTTAGAAATCTGGAGTTTTTTTTTAATCAGTCCCTCTTGGCGAAACAAATATGGAGAGTCAT

AAGAAATCCTAATTCGCTTTGCAGCAGAGTCTTAAAGGGTTGTTATTTTCCTAACATTTCAATTCTAGAA

GCATCATGTGGTTCAAGTGCATCTTCAGTTTGGAGGAGTCTGATATGGGGGAGTGAGATTATCTTTAAGG

GTTATAGATGGAGAGTGGGTGATGGTAATGATATTAGAATCATAGAAGATTCCTGGATTCCCAGACTGAA

AACTTTCAAAATTTTTGAAAAACGTCATATTCCTCCTAATCTTTATGTAATCGATCTTAAACGAGGAGAT

GGATCATGGGATGAAGAATTTATCAGAATGAACTTTTGCAAGGATGATGCTGAGGTAATCACTCAGATTC

CATGCTGTGATAATGATCTAAAAGATAAGATTATTTGGCATTATTCCAAGGATGTGGAGTATAGTGTGAA

GAGTGGATATAAGTTGGCCATGAAATCGAACAATCAAGCTGAAGCTTCAAATATGAATGAAACTGAGAAT

TGGTGGTCTTCGGTTTGGAAAATGAAAATTCCACAGAAAATCAAATTCTTTATCTGGAAAGTTACTCACA

TGTGGTTGCCTACTAATTTTATTATTTATCGTAAAGGTATCACTAACTCTCCTGAATGTTTGCGATGTAA

CATTGGAGCTAATGAGGATATCATGCATGCATTATGGTCATGCCCCGCTACTTCTAAAATCTAGAAGATT

GTTGGATGGTGGCAGGTGATGAAAAATTATAACGCTTTAGATGTAAGTAGCTTTCTAATTAAAATGAAGC

AAAGATTGACAATGGAAGAATTTGAATTATTTTCTGTCATTAGTTGGCAAGTCTGGTACACGAGGAATAA

TGTTGTTCATTGTGGAATTTGTCCTAAACCTAATGTTCTGGTTAGCTGGTGTTGTGAATATGTCAAAAAT

TTTAAACTTTTAAATGTTTATCACATGAAGGTGGCTGATAGATCAAAATGCAAATGGAAAGTGCCTTCTC

CAGGTATGCTCAAGCTCAATGTAGACGGAAGTATAAATATGAAGAATCCAGGATGTGGTTTAGGTATTGT

GGTGTGCAATGACAAAGGAGATTGTATCGAAGCAAGGTGTGTTTATTTGAACAAATGTAGTTCTCCTTTA

GCTGCTGAATTGTTAGCTATTAAGGAGGGGTTGAAGACTGCCAG

>MSTRG.646.1 gene=MSTRG.646

ATTATTTGGCGGATTGTGGATTTTCAAATAGACTCCAGTTTTTAGCTCCCTTCCGTGGTGTTCGTTATTA

TCTTCAAGATTTTCATGGTCAAGGTCGTCATCCTGAAAATGCCAGAGAATTGTTTAATATTCATCATGAT

TCCTTGAGGAATACAGTGGAACGATTATTCAGAATATTCAAATCACGATTTACAATTTTCAAGACGGCGC

CTCCTTTTTCATTTCAAACACAAGCGGAGATGGTTTTCGTTTGTGCAGGCTTACATAATTTTCTTCGCAA

AGAATGTCGTTCAGATGGATTTCCAGTTGAAGCAGATAATGAAACTTCAGCAACAAAACAAGAAGAGTAT

TTTGAACCACTTATTCATGAAACACAGGAGCAACAACGTGAAAATGCTAATGCGTGAAGAAATGATTTTG

CGCATGAAATGTGGATGAATGTTGAAGATAGGCGGAGCTA

>MSTRG.648.1 gene=MSTRG.648

GATAGATCCGGATAGGTCAATCCGGACCCACCAACTTTCACTATCCGGGTACCTCGCCGGCTTACAACCA

AGGACGTCAACCCTGGAAGGAGACTCAACCCGGGTAGGCACCTAATCCACCCTACCCGGGTACACATGTA

CCGGCCGACAACCACTGCACAAGGCACAGACTGACACGATGAAGACAAATGTAACGGTCAACTACTGACG

ATAGAGACAAGCCAGGGAACATTCCAAAATACTACTCCTACGCTGACACCTGGACACGTGTAGGGGATCG

AACCCCTACGCGTGTAGGACCAGGATCCAGGTACTCACCCTTAAACCCTAATCCTTGGCCTATAAATAGA

CCAAGGATCAAGAGTCTAAGGGTGACTCTCTCT

>MSTRG.649.1 gene=MSTRG.649

CACAAAACCGAGAACTGCATATCCCTCAAGTATTTCATCGAAGAACAGATCAGCAAAGGAAACATGGGGC

AATATGTAGCTCGGAACACAGCCGACAAAACAGGAGGATCCGGGAAGCAGAAAAACATTGTCAACGTAGT

GCTGGGAGGATCCTGTTCCCCTCCCCCTAGCCCGGGCTCCTGTCAAGAAGTAATGTCCATCCAAGCCTAC

CCCGAAC

>MSTRG.650.1 gene=MSTRG.650

GTCTGCGAAAGATTCTTTTGCTTCTGCTCTTCGGAATTTGGGAGGCGTGCATGTGGCCCCGAAGCCTCAA

CCGAAGAAGAAATCAGGGAAGAAAGGGGACTCGAAGAAAGGGGAGTCCGGGTCGAAGAGAGGGGAGTCCG

GGTCTTCCCGTCAAGGGACGGAGGGTACTGGGCATTCCAGTGCTAACCTGGGTGCAACTCCGGATCCGGA

GTTGATCGCTGATGCTGAAGAGGTGGAGAGCCCGGATCAGGGGCCGAAAAGAAAGAGGAAGAAGACTGAT

GCTGATTCTTCTGAAAGGGAAGTCATTGATATGACAGTGGAGGGTCCGGGTAAGGCAGCTATGGAGGTTG

TGGATCTTGGAGTCGATGTTGGGGGCGGTTCCCGGCCGGCTCCTAAGTTTGGCAAGTACCCGGTTAAGAA

AGTGCTCGGGTTGATGTCCGAACTTCCCTCTGACCAAGACTGGGAAATGATGGAGGATGAGGGTCTCGCC

ACCAATTTCAAAGAAATCGGGAACTTGTGGGGTCAGCTCGGTGGTCGTCTGGCCGGGTTCAATACCCATG

CTTTGAACAGCTTGAAGCAGGAGAGGGATCTTTCCGTGAAGAGCCTCGCCCGGGTCACGAAGCTGGAGAA

GGATCTGGATGTGGAGAAGTCAGCCCGGGAGGCTTTGGAGTCCGGGGTAGCCTCTAAGATCAAAGAAGCT

GAGATCCGGAAAGAGGCTGAGCTGAATCAGAAGATTAAGGATGCTGGGACCCGGGCTGACAGCGCTGAGA

AGAAGGTTTCCGGGCTGGAGAAGGAGGTGGCTGATCTGAAGAAGCTCTTGGAAGGGAGAGAGGAACCGGA

GAAGGTTATTGCGGAATTCCAAAAGTCCTCTGCTTATGCGGATGCCTTGGCCGCCGCTGGAGCTCTTGAA

GTCGTCCGGTGTTGGCATGTTGCCGAGCGGCACATCAAGACTGACCCGGAGGCTTCCTTGCAGAGTTTTA

TAGAGCTCTATCTTGCTGCTAAGGATGAGATCAAGGCTGGGAAAGGAGAACCGGAGCCTTACGAAGGCCC

CTCTCCTAGCTTTCTCCCTCCTGCCAATCCGGGTGCTGATGCTGATCTGGGCTCTTCCTCCGATTCTGAA

CCGGTTGATGGGACTCCGGTTGAGAAGACTCCGGCTGATGAAACTCCTGCTGATGAAACTCTTGCTGACT

GATCC

>MSTRG.651.1 gene=MSTRG.651

GATAGATCCGGATAGGTCAATCCGGACCCACCAACTTTCACTATCCGGGTACCTCGCCGGCTTACAACCA

AGGACGTCAACCCTGGAAGGAGACTCAACCCGGGTAGGCACCTAATCCACCCTACCCGGGTACACATGTA

CCGGCCGACAACCACTGCACAAGGCACAGACTGACACGATGAAGACAAATGTAACGGTCAACTACTGACG

ATAGAGACAAGCCAGGGAACATTCCAAAATACTACTCCTACGCTGACACCTGGACACGTGTAGGGGATCG

AACCCCTACGCGTGTAGGACCAGGATCCAGGTACTCACCCTTAAACCCTAATCCTTGGCCTATAAATAGA

CCAAGGATCAAGAGTCTAAGGGTGACTCTCTCT

>MSTRG.652.1 gene=MSTRG.652

TTATAGACGTCGAAATTTCAAGAAGTTGAAAGACCTCATCCCAGTATTGATGGACAACCAATTTCCCCAA

CTTTCATACCAAGATCTTCTGCTTGCTACAAATGAGTTTTCTCCAGATAATATGCTCGGTAGAGGAAGAT

ATGGTTCAGTTTACAAAGGAGTTCTCAAAGCAATGGAGCAAATAGTTGCTGTGAAGGTACTAAACGTTGA

AATACATCGAGCAAAGAAAACTTTTTTGGCAGAGTGTCAAACATTGAGAGAGATTCGACACCGGAATCTT

ATCAAGATCATTACAGCATACTCTACCATTGATTTTAAGGGCAATGACTTCAAGGCATTGGTTTTTGAGT

ACATGGCTAATGGGAGTGTGGACAACTGGTTGCATCCAAGCCCTTCCCATCAGCGGAATGAAAGAAACTT

GACTCTACTACAGAGATTAAATATTTCCATCGATGTTGCAATGGGATTGGACTACCTACATCATCACAGC

CATGAAAGTATCATTCATTGTGACATAAAGCCAAGTAATATTCTTCTTGATGAGGATTTTGTTGCTCATA

TTGGGGATTTCGGTTTCGCGAGGTTTTCTCATGGTACCACAAGTGATGTCAATCAAACACAGACGAGTTC

AACAAGTGTACATGGAACATTTGGATATGTTCCTCCAGAGTATGGAATAGGTGGAGAGGTATCTACAAAG

GGTGATGTGTATAGCTATGGAATTCTTCTACTAGAAATTTTCTCAGGGAAATGCCCTACTGAAAGCAGCA

TATTAAAAGACGGTTATAGTAATCTTCATAATTATGTGAGGGCAGCACTCCCACGAAGAGTGATGGATAT

TGCTGATCCGCAGATTGTACTTGATCAAGAAGAACATGGCTTGACTGTAAATCAATCATACAGCAGGGCT

TCCATGAAAGTATGCCTCACATCAATATTTGAAGTAGGGATATTATGCTCTGAAGAGTTGCCACAAAAAC

GCATAGACATTAATGTTGCTATTAAGAGATTACAGGCAGCACAAGACACAGTTCTGCAGCACAGGCAGTA

AGTTGGGTGAGAGTGGAAATCTCTATACTTTCTAAATAAAGGGATCTATATGTTGATCCTGATGTGAAAA

TAAAGACTTTGTACCTATTTATTTCCCATATTAACAGATTACATGCGGCAGAAGACACAACTCTGCAGCG

CAGGCAGTAAGATGGATGAGAGTGGCAATCTCTATACTTAATAAATAAAGGCATCTCGATATATATGATT

CTAATGTGACAAGAAATAATTTTTACCTATGTATTTCCC

>MSTRG.653.1 gene=MSTRG.653

CCCTGGCTCGTAGAGCCGTGTTGTTGTTGATCAGCCATCGTCATGTGTTTCCTCGTAGAATCTTTAGCAA

GTTTCCCACAGACGGCGCCAATTGTTCGTGCAAGATTTTGCGGGGTTAAATGCTAAAGAATTAAACTAAG

AATAAAATGAACAAGTAACGAGACAAAGATTGGTTACGCGGAAAACCCCCGAAGAGGAAAAAACCGCGGG

AGGGTCGATGCCCTGCCAAATATGTGTTTCACTATGTAGATAATTTGCGTACAATGTTTTTGATATGAGG

CTGCTTATTGCTTGCCCTTTACAGCTTGAATCTAAGCCCTATTTATACAAGTCATATTGTAACTACTACT

CCTACTAATGCTCCACGTGCAGCTGTAACTTATCTCTCCTACTTATCCGCATATGACCACCTCAATGCTT

ATCTTCCGCCTTTGACTCGTTCTCCGCGAATTCAAACAATCGGCATGCCGTCACGTGCACACGTCCCGTC

TTGTCACCCTTCTGTGATGTCAAG

>MSTRG.654.1 gene=MSTRG.654

GATTTTGCGGGGTTAAATGCTAAAGAATTAAACTAAGAATAAAATGAACAAGTAACGAGACAAAGATTGG

TTACGCGGAAAACCCCCGAAGAGGAAAAAACCGCGGGAGGGTCGATGCCCTGCCAAATATGTGTTTCACT

ATGTAGATAATTTGCGTACAATGTTTTTGATATGAGGCTGCTTATTGCTTGCCCTTTACAGCTTGAATCT

AAGCCCTATTTATACAAGTCATATTGTAACTACTACTCCTACTAATGCTCCACGTGCAGCTGTAACTTAT

CTCTCCTACTTATCCGCATATGACCACCTCAATGCTTATCTTCCGCCTTTGACTCGTTCTCCGCGAATTC

AAACAATCGGCATGCCGTCACGTGCACACGTCCCGTCTTGTCACCCTTCTGTGATGTCAAG

>MSTRG.656.1 gene=MSTRG.656

GCCAGTTACACAATCTCATCCAAAGAACTGGACGATTAGATATCAACAAACCCTCCTGTTCGATTTTATA

CCCCCAACCAGGAATCTTACGTATTCTTGCTGCTTTGATTATATATGGACATGGGAATATAGAGTTGTCA

AAGTATCATGTCGTCAAGCTCCAGTACTATACATAATCGACTCATCTTTAACCCATAGGAGTGTGTGAAT

TTGTTTTTTAGAAGTTTTCCCAAAACACTGCAACCAGTGGCAAGTGGATGAGCACATCAGCATTAAAACT

TGATCTGTCAATCAAAAGTCCCTACAGGATCACGAACTGGAGCAGGCCAGGATGTTATTAATCTGCTGGG

CGCTAGCATGTCTGAAGTGTGTCCGAGAAAAAGAGTTATACCACAAGTAAGCTGAGAACTGGGATTAACA

AAAGTGTGTATCCTGCAATCAAAGTAG

>MSTRG.656.2 gene=MSTRG.656

CAATCTCATCCAAAGAACTGGACGATTAGATATCAACAAACCCTCCTGTTCGATTTTATACCCCCAACCA

GGAATCTTACGTATTCTTGCTGCTTTGATTATATATGGACATGGGAATATAGAGTCCCTACAGGATCACG

AACTGGAGCAGGCCAGGATGTTATTAATCTGCTGGGCGCTAGCATGTCTGAAGGCTACGACAGAAAATAT

CATTGTCGGTTCTCATGTTTGGATTGAAGATCCAGAAATAGCTTGGATCGATGGACAAGCTACAAAGATT

AATGGACAAGAAGTTCAGATAGATACTACTAGAGGAAATTCGGTGCTTCTAGCGGAGGACTCGGTGCCCA

TGTGAACTGGCATGTTTGATGCAAATCAAGTGAAAGCTGTAGGAAATGAAAGAGCAAGGTGTTTCTGATG

ATAAACTATTACTTCTCAAGGGCATGAGTGGAGTTTTCAGGTCAGGGGTTCTAACTGCTCTAATGGGTGT

TAGTGGTGCTGGACTGTTGGTAAAACAACTTTGATGGATGTATTGGCTGGTCGGAAACTCGGAATTGGTG

CTGGTTTCATTGCTGATGTTTTGGACATGAGCATTCGTGACAAAGTAATTCAAGTAGGCATTTTGGCAAA

AGCTATAATGTCGTTGACTTCAGTTAATAAATTTTATGGAGAGGCTCTTTCGAGCTTTTATGCACAATTA

TCTGGCAAGATTTTAGTCTAAATTGCATCAAGAGTATGATTACTAAGTAGGTAGTGAATGTTCTATTGAT

TTTTGAGACATCTTGAACAGG

>MSTRG.660.2 gene=MSTRG.660

ACTGTACCACGTGTATCGGTACGTGTATACCTCTGGTTTCTCTCTATCTCTTTTCTCACCCTTGACCTCG

ATCTCTCTCATTTTAATCTCGCTACCAATCTCTCCCGCACTCTCCTTCTCTTTCCCCAATGTGACTCACA

TCTCCTCCTTCAATTTCCCACCAACCGTCAACGAGCCCCATTGCTGCGGTAATCTGATTCAGCATCTTTC

TCCGCGGATTCTGCTCCGTTTCTTCTCCCTGTGTTGTGTCTCTGTGGTACCCTCTGTAGTGTTCTCGATT

CCGTTGCCCAACCACCATCCAACAGCCATTGCTGTCCACTTTTCCGGCAACCCGAGCCTTGCCTCGTCTT

GTGTCTTAATTTCAAATTGAACGAGACGTTGATCGAGGCATTGATCGAGAAGTGATTGAAATGGGTATTC

AGAAATAGCTAAGTTGTGTTGCAAGTTGAAGTTAGGCAGAGATTTGCTATAGATAAAATCTTGTCAGAAG

CTGGCAGAAAGGAATGATATGGCACTACATAGTGTTACTAGAAATCTGAAATAGACACCGG

>MSTRG.660.1 gene=MSTRG.660

ACTGTACCACGTGTATCGGTACGTGTATACCTCTGGTTTCTTCTCATTTTCTTTTCTTCTCTCTCTATCT

CTTTTCTCACCCTTGACCTCGATCTCTCTCATTTTAATCTCGCTACCAATCTCTCCCGCACTCTCCTTCT

CTTTCCCCAATGTGACTCACATCTCCTCCTTCAATTTCCCACCAACCGTCAACGAGCCCCATTGCTGCGG

TAATCTGATTCAGCATCTTTCTCCGCGGATTCTGCTCCGTTTCTTCTCCCTGTGTTGTGTCTCTGTGGTA

CCCTCTGTAGTGTTCTCGATTCCGTTGCCCAACCACCATCCAACAGCCATTGCTGTCCACTTTTCCGGCA

ACCCGAGCCTTGCCTCGTCTTGTGTCTTAATTTCAGTGGAATTGAACGAGACGTTGATCGAGGCATTGAT

CGAGAAGTGATTGAAATGGGTATTCAGAAATAGCTAAGTTGTGTTGCAAGTTGAAGTTAGGCAGAGATTT

GCTATAGATAAAATCTTGTCAGAAGCTGGCAGAAAGGAATGATATGGCACTACATAGTGTTACTAGAAAT

CTGAAATAGACACCGGAGTTCAGGCAAGTATATATCCAACCTACTTTCTCTTTAAAAGATTTTGCTCCTG

CAATGCTTTTGTTATGCTTTGAATATTGCAAAACTCCTTATAGAATTAGACTTCTGCTAATTAACTAGTA

TTCTGGAATTACCCAAGTATGAAATGGAATACCCTTTGTCACTTATATCTTGCAAATATATCAACTTAAT

TATCATCCTTATCTTCTTCAACTGTCAGTTACTTAATATTGAGCTTAGAACCTTGTTTTCTTACCTCCCT

TTTAACTGCAACAAATTGTGTTCTTGCTTATAAACTAGTTCACTTGTTTGTTCCTGACCCTGAAATGAAA

CCCCATTTCTTGAAAGACCTTAGTTATCATCATACAACCTACCCCGCCATCATTATAAAATACATTGATA

CACTTGATTCAGTAACCTATACCCTTGATAAAGAATACTTAATTAGTA

>MSTRG.660.3 gene=MSTRG.660

CGTGTATCGGTACGTGTATACCTCTGGTTTCTCTCTATCTCTTTTCTCACCCTTGACCTCGATCTCTCTC

ATTTTAATCTCGCTACCAATCTCTCCCGCACTCTCCTTCTCTTTCCCCAATGTGACTCACATCTCCTCCT

TCAATTTCCCACCAACCGTCAACGAGCCCCATTGCTGCGGTAATCTGATTCAGCATCTTTCTCCGCGGAT

TCTGCTCCGTTTCTTCTCCCTGTGTTGTGTCTCTGTGGTACCCTCTGTAGTGTTCTCGATTCCGTTGCCC

AACCACCATCCAACAGCCATTGCTGTCCACTTTTCCGGCAACCCGAGCCTTGCCTCGTCTTGTGTCTTAA

TTTCAGTGGAATTGAACGAGACGTTGATCGAGGCATTGATCGAGAAGTGATTGAAATGGGTATTCAGAAA

TAGCTAAGTTGTGTTGCAAGTTGAAGTTAGGCAGAGATTTGCTATAGATAAAATCTTGTCAGAAGCTGGC

AGAAAGGAATGATATGGCACTACATAGTGTTACTAGAAATCTGAAATAGACACCGGAGTTCAGG

>MSTRG.660.4 gene=MSTRG.660

CGTGTATCGGTACGTGTATACCTCTGGTTTCTTCTCATTTTCTTTTCTTCTCTCTCTATCTCTTTTCTCA

CCCTTGACCTCGATCTCTCTCATTTTAATCTCGCTACCAATCTCTCCCGCACTCTCCTTCTCTTTCCCCA

ATGTGACTCACATCTCCTCCTTCAATTTCCCACCAACCGTCAACGAGCCCCATTGCTGCGGTAATCTGAT

TCAGCATCTTTCTCCGCGGATTCTGCTCCGTTTCTTCTCCCTGTGTTGTGTCTCTGTGGTACCCTCTGTA

GTGTTCTCGATTCCGTTGCCCAACCACCATCCAACAGCCATTGCTGTCCACTTTTCCGGCAACCCGAGCC

TTGCCTCGTCTTGTGTCTTAATTTCAAATTGAACGAGACGTTGATCGAGGCATTGATCGAGAAGTGATTG

AAATGGGTATTCAGAAATAGCTAAGTTGTGTTGCAAGTTGAAGTTAGGCAGAGATTTGCTATAGATAAAA

TCTTGTCAGAAGCTGGCAGAAAGGAATGATATGGCACTACATAGTGTTACTAGAAATCTGAAATAGACAC

CGGAGTTCAGGCAAGTATATATCCAACCTACTTTCTCTTTAAAAGATTTTGCTCCTGCAATGCTTTTGTT

ATGCTTTGAATATTGCAAAACTCCTTATAGAATTAGACTTCTGCTAATTAACTAGTATTCTGGAATTACC

CAAGTATGAAATGGAATACCCTTTGTCACTTATATCTTGCAAATATATCAACTTAATTATCATCCTTATC

TTCTTCAACTGTCAGTTACTTAATATTGAGCTTAGAACCTTGTTTTCTTACCTCCCTTTTAACTGCAACA

AATTGTGTTCTTGCTTATAAACTAGTTCACTTGTTTGTTCCTGACCCTGAAATGAAACCCCATTTCTTGA

AAGACCTTAGTTATCATCATACAACCTAC

>MSTRG.661.1 gene=MSTRG.661

ATCTTTCCCAACATCTCACATGAGTCGGCGTCACCTACACTATACATAATTTCTATTTGTACAACAACAT

GAGTGTACATGAATCTTCCTATCCAATTAATCTACGTGATATGCATGGACTTATATGGTTGTCTTTATTT

CCACATTCTATTTAAATAGCTAGCCGGTGCTCGCATAAGTGGAAACTAGAAAACAAAGAGATTGAGGTGA

CTCGAATAAAAATTGTACGAGATCAAAGATGGACAAGAGCAGGTCATTCCCGGAGTACTCATCGTGCGGT

GAATTGGAGTCGGAGAATCGAGCGAATGCTTATTCGTTCAACGGGCCAAACGCCACACCGCCTAGTGATC

CAGAGCTCAAGAGGAGGAAGAGGATTGCTGCATATAACATGTTCACCACTGAAAGAAAACTCAAGTCTAC

TATGAGGGAGAGGCTCAAGTGGATTAAGAATAAGTTCACCGATGTTCGCTATAGTTTCTGAAGTCATTTA

TCAATCTATCTATCTATCTGAGTACAAATAGTTGTGTAACTAAAGAAGCTATATCCTCTGCAATGGATTT

CAGTTCTGCCCTTTGTCGACAAGCTTGTGCCTGTTCAGAGGGATCATTATAAATTATTAAAGTGTGTCGT

TCACGGTACATAAAAATTCCGCATCCGACCGAAGAGTTTTCGGTTTTGAATCATGGTGATCATAAAAATG

TAATCAAGCTTCGGAAATGATATATTCTAAGCGTCCTCCTATTATTTTTCTACATTTGTAAGCATTGTTG

TTCTCGTGTGTGTGTGTTTTTCGATTGTTGGCATCAAGATGCGCCCCTCGGAATCAAACAATCAAATACT

TGATAGTTGAAAACATCTTGTAATGGTTGCCGAGTCGCAATCAATATAACACAAAAGGACAAGTATAGAA

ACTAAAACAATGTTTG

>MSTRG.666.1 gene=MSTRG.666

GTCAAACCCTAATGTAAAATAACTAATCCCTTTCAAATCATTTCAGTTAAACATTCTTATTTATACACAC

ATACACATACCTGTGTCTATCTCCTTGTTTTCACACAAATTCATCGCCAAATCGCAAGAGCTCACACTAC

AAGATCCGAAATTCACACTCATTACACACTTCTAAGCTCCTCACGGAAAACTAAGAGCTAAAGACGGGAG

TTCTGTGTTGTGTTGTTAATGCTGAAAGCAGAAGTTCATTTATGATACAGAAGAGGTAGATATCCTCTTT

TGGCCGAGAATTTGATTTTGAAATAATGGCGCCATCAAGCAAGGCTGATAAAAAGGCTGCAATAGATGCA

GCTGCATGGATGTTTAATGTAGTCACTTCCGTCGGGATCATCATTGTCAATAAAGCCTTAATGGCGACAT

ATGGATTTAGTTTTGCTACAACATTAACTGGTTTGCATTTCCTTACGACAACTTTGATGACGGGTGTCCT

TAGATGGTTGGGATATATCCAGCCTTCTCATTTACCTATTTCAGAGCTGCTCAAATTTGTCCTCTTTGCA

AATTTCTCTATTGTAGGGATGAATGTTAGCTTGATGTTGAACTCAGTCGGATTCTACCAGATCGCAAAGC

TAAGTATGATTCCTGTATCCTGCTTATTGGAAGTTGTCCTAGACAAAATCAGATATTCAAGAGATACAAA

ACTGAGCATCTTAGTCGTTCTGATGGGTGTTGCTGTCTGTACTGTTACTGATGTGAGTGTTAATGGTAGA

GGATTCATTGCTGCTTTTATTGCAGTGTGGAGTACTTCTATGCAGCAATATTATGTTCATTACCTTCAGC

GGAAATACTCACTTAGTTCTTTTAATTTGTTGGGGCACACTGCGCCAGTCCAAGCTGCCTCTCTTTTGTT

AATAGGCCCCTTTCTGGATTATTTATTGACTGACAAGAAAGTATATGAATTTGACTTCAACATGGCATCT

GCGACATTCTTATCACTTTCGTGCATAATCGCAATAGGGACCAACCTGAGCCAGTTCATTTGCATTGGCA

GATTCACAGCAGTGTCATTTCAAGTACTTGGCCATATGAAAACAATCCTTGTTTTGATCTTGGGATTCTT

GTTCTTTGGAAAAGAGGGTCTTAATATACAAGTGGTCATTGGCATGCTCATAGCCATAGTTGGAATGGTC

TGGTATGGGAATGCATCTTCTAAGCCAGGCGGCAAGGAACGCCGCAGCCACTCTATGTCTAGGAGCAGCC

AACAGAAGCTTGTTCCATCTTTAGAACCAACTGAACTTGATGACAAGGTTTAATCAGATCTCAAAATTTA

ATTGGAGCATACAGAGGGTAGCTGATACAATAGGTACCAGGAAAGCTTTAGCAATCCGATTCTAGATACA

AACAGTCAAATACTGCCATATCTTTCCCGATTCTGTAATTCTCATCTTTCATTGTGGAAATTCTTGGGGG

TTTAAAACTCAACTATATAGAGCTCTATGTTTTCCCATTGTTATAATTTATAATAATTTATACTTGTGCA

TTTTTTTATACAATTAAAATATTCTTTCTTCAGGTTGACCTTATATGAGT

>MSTRG.666.2 gene=MSTRG.666

CTTATTTATACACACATACACATACCTGTGTCTATCTCCTTGTTTTCACACAAATTCATCGCCAAATCGC

AAGAGCTCACACTACAAGATCCGAAATTCACACTCATTACACACTTCTAAGCTCCTCACGGAAAACTAAG

AGCTAAAGACGGGAGTTCTGTGTTGTGTTGTTAATGCTGAAAGCAGAAGTTCATTTATGATACAGAAGAG

ATGCAGCTGCATGGATGTTTAATGTAGTCACTTCCGTCGGGATCATCATTGTCAATAAAGCCTTAATGGC

GACATATGGATTTAGTTTTGCTACAACATTAACTGGTTTGCATTTCCTTACGACAACTTTGATGACGGGT

GTCCTTAGATGGTTGGGATATATCCAGCCTTCTCATTTACCTATTTCAGAGCTGCTCAAATTTGTCCTCT

TTGCAAATTTCTCTATTGTAGGGATGAATGTTAGCTTGATGTTGAACTCAGTCGGATTCTACCAGATCGC

AAAGCTAAGTATGATTCCTGTATCCTGCTTATTGGAAGTTGTCCTAGACAAAATCAGATATTCAAGAGAT

ACAAAACTGAGCATCTTAGTCGTTCTGATGGGTGTTGCTGTCTGTACTGTTACTGATGTGAGTGTTAATG

GTAGAGGATTCATTGCTGCTTTTATTGCAGTGTGGAGTACTTCTATGCAGCAATATTATGTTCATTACCT

TCAGCGGAAATACTCACTTAGTTCTTTTAATTTGTTGGGGCACACTGCGCCAGTCCAAGCTGCCTCTCTT

TTGTTAATAGGCCCCTTTCTGGATTATTTATTGACTGACAAGAAAGTATATGAATTTGACTTCAACATGG

CATCTGCGGTAAGTACTGCATACTGTTTTGTTTATTTCCGTGAACTCCGTGAAGTAGCTGAATAGGTTCA

GCTTTAGAAGTGTTTATGTGGCATGTCATGTTGAACTCCTGCTTTTTTCCAAATACTAAGATCTCACAAT

TTTTTCTCGTGCAGACATTCTTATCACTTTCGTGCATAATCGCAATAGGGACCAACCTGAGCCAGTTCAT

TTGCATTGGCAGATTCACAGCAGTGTCATTTCAAGTACTTGGCCATATGAAAACAATCCTTGTTTTGATC

TTGGGATTCTTGTTCTTTGGAAAAGAGGGTCTTAATATACAAGTGGTCATTGGCATGCTCATAGCCATAG

TTGGAATGGTCTGGTATGGGAATGCATCTTCTAAGCCAGGCGGCAAGGAACGCCGCAGCCACTCTATGTC

TAGGAGCAGCCAACAGAAGCTTGTTCCATCTTTAGAACCAACTGAACTTGATGACAAGGTTTAATCAGAT

CTCAAAATTTAATTGGAGCATACAGAGGGTAGCTGATACAATAGGTACCAGGAAAGCTTTAGCAATCCGA

TTCTAGATACAAACAGTCAAATACTGCCATATCTTTCCCGATTCTGTAATTCTCATCTTTCATTGTGGAA

ATTCTTGGGGGTTTAAAACTCAACTATATAGAGCTCTATGTTTTCCCATTGTTATAATTTATAATAATTT

ATACTTGTGC

>MSTRG.668.1 gene=MSTRG.668

GTCAATTTAATTGTCTAGTAATGTTCTTTCTTCTGTTTCCAAGATCTGGACCTGATAGAATTTTCTTGGC

AGTGTGGATTGGACGTTGCAGAACACAGGTGTAAGCACAAGCAATAACTCCTCCGTGGTCACAAGGTCTC

CTGTTGGTGGTGTACACTCTAGCTTGGATGTACAATCAATTTGGTCATATGATTAAAAAGGATGTTAAAA

GGATAAGGAGAACTAATAGTGGACTTGTTAAATATACTTCCTGTTCAGAAGAAAGGAAAAAGGTTTCCTC

TTTTGTCTGGGATTTAGAATACTGGATCCAATATATCGTCCTTGAGTTTATCAGGGAAGGTTTACTTGCA

ATAGCATATATGACTTGCTAGTGCAAGGCATGTGCTAGGAGGCAGTAGGTAACATATCAAGCTTTTTTGG

TATAATATCTTGGTCCGTCTTCCTTGAAAATTGCGTTTATTTTACTTACAAATGAG

>MSTRG.674.1 gene=MSTRG.674

CATTATGATCCGTAGTAGTATCACAATATCATTAACGACGACAACTTAGTTGCGATACATGCATAACGTC

AAACATCTTTATCATGTACAAATTTGCCAGGAAGATGTCTTCAGGACCAATTATTTTGCACTTCTAAGGA

ACTCACATCTTCAAGTTGTGTTTGATGTTGCTGTTGCGTTTCCCTCCCCGTCTCTCTTTTAATTATTTTT

TGGATTTCTGATGCTTTGTGGGTTTATCTGACAGGTGTGCCATGGCACTTGTTCATCAGCGGATTAAGCG

TATATTTTACACTTTTCCTAATCACAATGCTGGGGCGCTTGGAAGTTTGCACAGATTGCAAGGAGAAAAA

AGCTTAAATCACCACTATGCTGTTTTCAGAGTCTTTTTACCAGAATCTATATTGGATAGAGATGATGTTG

TCACTGCTGTTTCTGGAAGTGATAAAAACAAAACACATGTTCTATAGGAGGTATGTACTTGCAATTAATT

ATGCCCCTTGTTTTTTATAGGAGGGAACACTTCCATACTACCATGGTAGCAGATCATCTCCAAGAGACTC

TCTAATTGAGCTCTTAAGTAGAAAAATAAAGAGCCATGTCCAAAATAGAGCTCCAAGAGACTCTTAGAGG

CTTTTTAAAAAGTAAAGAGCTCACTCTCTCTCCTCTCTTTTAGGAGCCGCA

>MSTRG.675.1 gene=MSTRG.675

CACTTTAACCGTACGGGTTAACGTGTAACCTAGTTCTATATGAATTCTTATTACTGTGTAACTATTATAA

TTCTTTTCAACAAAACGAGGCCCAAGAAAACATCAACAGCAGGAGTGGGAGCTTAAATAACCCACCCCCA

ATTGCTTGTATATATCTGTACGCTAAGAAAGGAATGGATATGAATGAGAAAATAGAGATATTGAAATCAG

AATTTCAAAAGCTGTCAAAGGATGGACAAGCTTGGGTGCAACATTTATCAGTGGAGATGAATAAACAAGA

GTGGTTTCAGAAGTTGCCCCCTTTTATTCAAAACATACCTCCACTTCAACTCTATGTTGCACTTGTGGTC

TTGTTCCTCACCATGTTTTTCTTCTTTATAGTTCGGTTATTCAACCGCAAGACATCTAATACCATATTAC

TCACTGGGCTAAGTGGAAGTGGAAAAACTGTCCTATTCTATCAG

>MSTRG.676.1 gene=MSTRG.676

GAGCTGTAGAAGAAGAGTGAAGAAGTAATGGAAAGAGCCAATGATGCAAGTAATGGTGGTGGTGATCTAG

GTGGAGGTCATGTGATCAAGACAGAAGACGATGAGAAGGTAACAATCAAGGTGCAAAAAGAAGGAATGGC

TGATTTCTCCTTCACTATGAACCGTGATCAACCATTAAGAAAGCTTATGATCGCGTTCTGTGAGCGTCTG

AGTCTAGGTGATTATAATAACTTGTGTTACTATTT

>MSTRG.679.1 gene=MSTRG.679

GTAGTGGTTGACAGTAGACTCAAAAAGCTCTTTCTTGCTGTGTAGTAGCTGGGTTCGATTCCTTGGAACT

ATGCAATTTTTTCGTTTCCATGTCACTCGGCTTAAATGGGCTGTACAAATGAGTCCATGAAGTTGGACCA

CTGCTAGCATTGCATATGAAGAAATTGATGAAGCGGCTTTGATAAAGCGGCTTGGATTTGATGGGCCTCC

AAATGGTTAATCTCCTTGGGCCTGGACTGGATCAGAGCAAGTGGAATGGGCTGCAAATGGAATGGGCTTA

GAGCAACTACATAGTAGCGCCTAGAATATTAGGCGGAAGCAGTAATGTGAGAAACTAGTGAAAACTATGG

ACGCAGGTTTAGCTGCTAAATAGGCTTAAGGCCCAGCCCAGCCGAAGAGGGAAACGCTTTAATGCGAGCA

GGTCAGCAACGTGAAGACAGAAGGCTAATACGCCAGTTTAATAAAAACTAGTTAAAAAA

>MSTRG.682.1 gene=MSTRG.682

CATTATAGTTATTTTCATCGAAACACAGAGACCCGTAGGTACAACAAATTTTAACACAGAGATGCTATAA

TTAACTTGTCACTTTCAAGTTTTCAGATGCGTGGTGCATTGTGTTTTACATATTTAAATAAAGATCAAGT

CTTCAGACAAGGTTACAAGTTTAGACATGGGTGCAAAGACTGATCAAAGTTCAAGATACAACAAATATGC

ATATGCTTGTCTCTTGTGTTCTATCCTGGTGAACATATTCTTTTCTTATGATCACTTGGTGAGTAACAAG

TGGAGTTTGAGTTGGAGCACAAGTGCAGCAGCAGAAGCAGAAGCTGTGGCATCGATTTCGTGTTCGGGCC

ACGGGAGAGCTTACTTGGATGGTTTAGTGGTTGATGGTAGGCCAATTTGTGAGTGTAATACTTGTTATGA

TGGCCCTGACTGCTCAAATTATATCCCTGATTGTCCTGCTGATGTAAACAGTGGGGATCCCTTGTTTTTG

GAGCCATTTTGGATGCAAAATGCAGCTAGTAGTGCACTAGTAGTGGCAGGGTGGCATCGAATGAGCTATT

CATTCGGTGACCATACATTTACCTCGCAAGAACTGGAGAAAAGCATCCGTAAATTACATTCTATAGCTGG

AAATGCAGTTGCAGAAGGAAAATATATTGTTTTTGGAACAGGCTCTACCCAATTACTCAATGCAGCAGTC

CATGCCCTCTCCTTGGATAACTCATCTACACCTTCAAAAGTTGTAGTTTCCATCCCATTCTACCCGGCTT

ATGAATTGCAGACTGAATTTTTTGCAACTGAGGACTATGAATTCTATGGCGATACATCGTTGTGCAAAAA

CAGTTCTGAAGACTCTCTGAATGTGATTGAGTTTGTAACTTCACCTAACAATCCTGATGGAAAATTGAGG

AAAGCGGTTCTTAATGGTCCATATGCCAAGTCAATCAATGACCATGCCTATAATTGGCCACATTTCACTG

CAATTCCATCTCCATCAGATGAAGACCTTATGATCTTTACACTTTCCAAGCTCACGGGTCATGCAGGCGT

TCGATTTGGGTGGGCATTGATAAAAGACAAGGATGTATATGAGAGAATGTCAACATATACGGATGACAAT

ACTTTTGGAGTCTCCAGGGACAGTCAGTTACGAGCTTTGAAGCTATTAAAGGTAGTTCTTGAAGGCGATG

GAAGATCAATCTTTAAATACGCTTATGAAACCATGAGAAAACGTTGGGAAGGCTTGAGCCAAGCAGTATC

ATTGTCCACACGCTTCACCATCCAAGAAATCGCGCCCCGCTTCTGTACTTTCTTTCAACATGTCAGAGGA

CCTTCTCCAGCTTATGCATGGCTGAAATGTGAGAGGGAAGAAGATGAACTATGTTACCAAGTCCTCCAAG

CTGGTGGCATACTTGGTCGTGATGGCACTCTATTCGGTGCTAGCAGTCGATATGTTCGCCTCAGCCTCAT

CAAGTCTCAAGATGATTTTGATCAGTTGCTACAACATTTAAACAAATTAGTTCTCCAGGAAGCTGGCAGT

ACTAAAGCAGATATCTGAAATTCTTTTGCATATCAAGGAGAAATACATATTTAATGATGTACATTTACGC

AAGTTTACGAGTCTGCTTATTGGCTCGGTCTATTAGTTCATGTAAATACAATAATAATACATTGTTGCTT

CCTTTGCAACTTGTATAAGTGTTTGTGTAGCTGTGTCTGTAATGTCAACTTGAAGTTGACATGTATTTAT

ACATCGATGATGCATTTTCCAGAGAGATCGACTTGAAG

>MSTRG.685.1 gene=MSTRG.685

GCTCCAACTGCAACTTTATACCTCGTCTCCCAATCCAGATTTCTGTTGCTCTGCAGAAGTTGTTGGAGAT

TCCCATTCGAGATATAGTTGTAGAGCAAGAGTTTGACACTCTTATTGGAACAGTACCCTAAAAGCTTTAC

TATGTTCCTATGCCGAATGTGCCCAAGGATTTGAATTTCTGCAGCAAAAGAGTCTATAGCTGCCTCTTCA

TCTCCCTTTGTTTTCCAAAGCTTCTTTACTGCTATCAACTCTCCATTGGGCATTTCTGCCTTGTAAACAA

CTCCAGAACAGCCTTTCCCGATCACATTTTCATCTTTGAGGCAATCTAGGATGTTGTCGATAGTGAAATT

TAGCTTTTGAAATGGAATGAATGTCCAGGGATAAGAAAAATCTTCTCGGCCCGATGAAGAGGCTAATGTG

CAAGATGATTTGTCTGCCATATACCTGTGATTCCTTGATACGAAAATCCAGAAGGCAACAACTGCTATTG

TCACAGAAGTTAAAATAACAGCTACCAAAGCTACAGTCTTGGCAGATTTTAATCTGTTCTTGCGAAACTG

ATGTGAGGAGCAAGTATAACCGTTAACAGATTGGCAAAGATCTTCATTTTCAAGGAATGAGTCGGAAGAT

AATGTTTTGAAAAAAGGTGTCACGGGTATAGGACCTGAAAAATTATTGTATGAAATATTTAAAGAAGTAA

GGCTTGTGAGGGAACCTAAAATTGCAATCTTTCCGTGGAGCATGTTGTGCGAAAGATCGAGGGCTTGCAA

TTGTGTCAAATCAGACATAGTCTCAGGGATCTCACCTGTAAATCCATTTGAGCTCAAGTCTAGACTAATG

GTTAAGCTTGTAACATCACCAATCTCATCTGGGATTGCACCAGAAAGACTATTGAAGCTCAAATCAAGTA

GAGTTAGTTTCTGAAGATTCCTAATGGACTTTGGTATTGAACCAGTAAGCAAATTGTTGTTGAGAATCAT

TTTATTTAAGTAACTGAAGTTCCCAAAACTCCAAGGAATTTCTCCAGTAAAACCGTTCCTGCTGAGATCA

AGCTGCTCTAAATTCACAAGCTCCCCCAACCGAGATGGTATATCACCACTGATATGGTTATTGTGCACAT

CAAAAAGCTCAAGAACCGTTATATTAGCAATCTCCGCAGGCAAACCACCAGAGAAATGATTTGCATATAG

ATCAAGAAACACAAGATTTTGCAATTGGCCCAACTCCTTTGGAATGTGTCCTGAGAGCTGGTTCTCCCCA

AGTCTTAATCTCACTAAAGACTGGCAATTAGCAACACTTGGAGGCAGCCCTCCCGACAAAGAATTCCCCA

GTAATAGTAGCTTGCTCAGTTTTTTCAAACCAAATAACTCCTCAGGTATACTGCCAGTGAGTTTGTTCTT

AGAAAGATCGACTGAATATAGTTCAGTGCAGTTCCCAAAAGAAGCTGGTATAGTTCCAGAGACCAAATTC

CCCCAAAGAAAGAAACTCTGCAAGAATTTCAGATTTCCAATTTGTGATGGAATTGGACCTGAAAGTTGAT

TCTTGTCAAGCTGAAGAGCTGTAAGACTAGTGCAGTTACTCAACTGCCATGGAATAGACCCTGTAAGAAA

ATTATCAGACAAATGCAGCTCTTCAAGAAGTACTAGCTTCCCCAAATCACCTGGAATTACACCGCTTAAC

TCATTTGCAGAAACATCAAACACTACTAGGGATGAACAATTTGATATTTCACCCGGGATAGGTCCACTAA

GAGAGTTTCCCCACAGGAGCAAGCTAGTAAGTTTCTGTAACTTGCCCAACTGTTGAGGCATTGAACCAGT

GAGCTTGTTCATATGCAAATACAAATTCCTCAGCTCTGAACACAGCCCAAGTTCAGGAGGGATTCTCCCA

GATATTTCAGTGTCGTAAATTGCCAAAGTTTGGAGATTGATCAAGTTTCCAAATGTAGGCGGTATGACAC

CAGATAGTCCAGTAGCTGCCGCGCCAAATGTTGTGAGATTAGTGAGTAATCCCAACTGCAGAGGTATTGC

TCCACTCAAATATGGATTACCCCCAATCCTAAACTGTTGTAAGGAACTTAATGAGCCTAACTGCGACGGA

ATCGACCCATTAAGTAGATTATCTTGAACACAAAACACTTGCAAAGATGAAAGATTCCCAATTTGTGGAG

GTATAGAATCTGTGAGTTGATTAGTATTCAAGAACAGAAACTGCAGTGAACTGAGCTGGCCTAGTTCAGG

AGGGATTGACCCTGACAACGAATTCGAAGACAAGTCCAAAAGATGCAGATGACTAAATGAGCCAAAATAA

GATGGAATATGACCAGAAATATTAGTAGAAGAGAGATTAAGAAGCTGCAGAGATGACAGTGAAGAAAGCT

CTAAAGGCAAACCAGAGAGATTGAGAAAAGTGTTTGGTATAGAAACAGAAATAACCCTGTCTTGTGGAGA

ACAAGTAATACCTTGCCAAGAACATGGAGTTTGGCTTGAAGGGGTCCAAGTAGACAAAACAGAAGATGAA

GATTTTGCATAATGGTCTGCAGTAGAGACCAGAGAGAGCAGAGCTTGGCCATCACTAGAGAGAGATGTCA

CAAGTTCTGGCCTTGTAAAAACTACCAAAAAACAAGAATAAACCAAGAAAAGTGAAGAGACTAAATCAAG

GGTTGCTTTATTTTTCTCCATTTTTTGAGACTTAAAATCAAGAAATGGGCTTACAAGTATGGTGGTGGTG

GTGGTGATGGTGAAAGTGAAAGTCCACTCTAGTAGACAACATGCATGTGAAGAACACAAATGTGAGATAA

TAATAAGAATGCTTAAAACTCACATTTGTGTGTGAATGTGTGTAAATGTTTCATTGCATGTGAACAAAAA

GAGAAACTTCAAAAGGAAACTAGTAGTGTTATTGTTGTTGTGTATCTTGAAGCTATCATCTTACCGGAAA

ATAAAAATGTGTTCTTGAAACTCCGGCAGGCAGGGG

>MSTRG.694.1 gene=MSTRG.694

AACCACCAACATGTTTTTCTTGACATCATGTATGATGAACACTCTGGCTTCGTACTACCTTAACTTTGTG

CTACCTTAAACACCTGATGAAATGAAATGCATCACCACTACACAGCTCTCTTCACACAATGCCTGAAAAC

ACAAAACCTTAACTTGGGAAAACAACTTCACTCCCACCTTATTAAAACATCTCTAACACTAGACACTTTC

TACACTAACCGCCTAATAGATATATATTGTAGATGCAATTCATTAGAATCTGCTCAAAAGGCATTTAATC

AACTTCCAGCAAAGAACACTCATTCATGG

>MSTRG.704.1 gene=MSTRG.704

AAAAAAACAAATTTTGGGTCACTTTTAAGGCCCAAAAGTACTGACCCATAACAAAAAAAAGAATTTTTGG

CCCAAATAAAAAGGCTATATCAGGCTCTCAGCTTTCAACAACTTTAAAAGGGGAAAACCGTAGCCGTTCA

AATAACCCCAATCCTTCAAGTTCAAAACAGAGCAAAGGTAAGAGGCCTCGACGTTTAATAGATGAGGACG

AAGAAGAAGAACGGAATTTGAATTTGAAGAGAACACTTTAATACATGAAGAAGAGTCAAATCAGGATTTT

GAAGATGACTTTGGTGAAGATGATTGATAAAATATCTACCTGTTAAGAGCTGGAAGCCTGAAATTCTGC

>MSTRG.706.1 gene=MSTRG.706

CTGAGATATTATCCTACTATCGTGTCTAATCGATCAAGACGTGTAGATCGAGAATGTTCAGAATTAATCT

GATGGTGTTGCAAGTTGGAGCAAGAATAATCTGAGAAGACAAACGTAGAAAAAGTATTGTCAGAAGCTAC

CTTGGAACATATGGTTGTGCGATATAGTGCTAATAGAATCGTGGTGAGACATCGGAGTTAAGGCA

>MSTRG.707.1 gene=MSTRG.707

AACCCTTTTCAGCTAGGGATAATGATGATTCACTTTAGACTAGGCCAGGATTGCAAGTGGTTTAGTTTAG

GCAGATGGTCAGGTAGAGTATCCAGGACGATATTATTACGACTAGATGCAGTTGGAGTTCTGCAATTCAA

TTCAGTTGGAAATTAGATTTATTTATTTTGTTATCAACTACTCTTCTTATCGAGGTTTTCTTATCGAGGT

TTAGCTGATAATGTAGTTTAATTTGTAATTAGGATTTTATAGTTTTAGTTTCTGATTTCAATGCTCTAAC

CTGTATGCAATCCTGTTTTCAGGGGGT

>MSTRG.709.2 gene=MSTRG.709

GTTTTAACACCCGGTTCTCGGCGGTTAAGAACGAATAAGAACACAACGCGAGATCTGGAAGTAATGTACA

GCTGAAAGATGTTGTCGGCGACGAGCAGAAGCTCCGCATCTTCATTACACTTGAAATCCACCAGACATTT

CCATAGGAAACCATCTCCATACACAATCACGCTTTACATAATCGCCCTATTTGCTCTCTCTATTTTCATT

TTTATTTACTACAGTAAAGATATCTTAGACATTGATGATCAGCACAAGCCCTTTTCGTCTCAATCCCACC

AGCTGTCAGATGAACAAGTCCAGGGAGCTCTCTCGAGCTATGGTTTACATCAATGCGTCGAACCAACTGC

AAAATATAAAGCGGCACTAGGATCGGATCATTATATAACTGTAAGAAGTAATGGAGGACTGAATCAAATG

CGAACTGGTATATCCGATATGGTGGCTATTGCACACATTATGAATGCAACCTTAGTAATTCCACAGCTGG

ATAAGCGCTCATTTTGGCAGGACAAAAGTAAATTTTCTGATGTATTTGACGAGCCACACTTCATTAGAAG

CTTACAAGGAGATATTCGGATTATCAGAGAGCTACCTGGGGAACTTGTATCTCTCCCTCATGCCAGAAAG

CACTTCACCTCCTGGTCTGGAATGGGATACTTTGAGGAGATGAAACAAATATGGAAGGACCACCAGGCAT

TACATGTTCCGAAGTCAGATGCTCGCCTTGCCAACAATGATCTTCCCCTTGATATCCAGAGGTTGAGATG

TAGGTCGCTTTATCATGCTCTTCGCTTCACACCTCCTATAGAGAATCTAGGGAAGAAGCTAGTGGAGCGG

CTGAGATTGCGTGCGGAAAGATATATTGCTCTTCACCTCAGATATGAGAAAGACATGTTATCTTTTACTG

GGTGTACCTATGGTCTTACTGATGCTGAATCCGAAGAGCTGAGAGTGATGAGGGAAAACACCAACCATTG

GAAGATGAAGAAGATAAATTCAACAGAGCAAAGAATTGGAGGGTTTTGTCCACTAACTCCTAAAGAGGTT

GGCATATTTCTTCAAGGTCTTGGTTATACTTCATCGACACTAATATACATTGCGGCTGGGGAGATTTATG

GTGGAAGCACTCACCTTAAAGAGCTTACATCTCGCTTTCCGAATGTTGTTTTCAAGGAAACAATTGCTAC

TGAGGAAGAGCTGAAAGAATTAACCAATCATGCATCTCAAACTGCAGCTCTTGATTATATAATATCAGTA

GAGAGTGACGTATTCATTCCATCGCATTCAGGTAACATGGCAAGAACTGTTGAGGGGCATCGCAGGTTTC

TAGGCCACCGAACAACAATTACCCCTGACAGGAAAGGTCTGGTTGACATATTTGATAAAATGGATAGTGG

ACTGCTTAATGAAAGCTCATCGTTGTCGCGCCTTATAATTAAGCTGCACAAAAACAGGCGTGGTGCTCCC

AGAAAAAGAGGCGGCCCTCCTCCAGGACTCAAAGGCCGAGCAAGGTTAAGGTTTGAAGAACCCTTCTACC

AAAATCCATACCCAGAATGTATATGTGATTCAAAAGTCACTACAAATAGTAGTCATAGTGCGTAGGTCAG

CTTGCACTTATCACTAAAAAAGATGTTCCGAGACATTAGATAATTCAAATTAGAATGACAGTGCTTTTTA

CTGCTGCAAGCCTTTTTGTAATTGAACTGGTTCCTTTTTTTGGTTCTTCGATTCTTTCTAGAATCTGTAT

TTTATGTTAAGATCCTGTACAATCAATACAAGTAGTTATC

>MSTRG.709.1 gene=MSTRG.709

GTTTTAACACCCGGTTCTCGGCGGTTAAGAACGAATAAGAACACAACGCGAGATCTGGAAGTAATGTACA

GCTGAAAGATGTTGTCGGCGACGAGCAGAAGCTCCGCATCTTCATTACACTTGAAATCCACCAGACATTT

CCATAGGAAACCATCTCCATACACAATCACGCTTTACATAATCGCCCTATTTGCTCTCTCTATTTTCATT

TTTATTTACTACAGTAAAGATATCTTAGACATTGATGATCAGCACAAGCCCTTTTCGTCTCAATCCCACC

AGCTGTCAGATGAACAAGTCCAGGGAGCTCTCTCGAGCTATGGTTTACATCAATGCGTCGAACCAACTGC

AAAATATAAAGCGGCACTAGGATCGGATCATTATATAACTGTAAGAAGTAATGGAGGACTGAATCAAATG

CGAACTGGTATATCCGATATGGTGGCTATTGCACACATTATGAATGCAACCTTAGTAATTCCACAGCTGG

ATAAGCGCTCATTTTGGCAGGACAAAAGTAAATTTTCTGATGTATTTGACGAGCCACACTTCATTAGAAG

CTTACAAGGAGATATTCGGATTATCAGAGAGCTACCTGGGGAACTTGTATCTCTCCCTCATGCCAGAAAG

CACTTCACCTCCTGGTCTGGAATGGGATACTTTGAGGAGATGAAACAAATATGGAAGGACCACCAGGCAT

TACATGTTCCGAAGTCAGATGCTCGCCTTGCCAACAATGATCTTCCCCTTGATATCCAGAGGTTGAGATG

TAGGTCGCTTTATCATGCTCTTCGCTTCACACCTCCTATAGAGAATCTAGGGAAGGTTCTGAGCATACAT

GACGGTGACACCACAGAAGCTAGTGGAGCGGCTGAGATTGCGTGCGGAAAGATATATTGCTCTTCACCTC

AGATATGAGAAAGACATGTTATCTTTTACTGGGTGTACCTATGGTCTTACTGATGCTGAATCCGAAGAGC

TGAGAGTGATGAGGGAAAACACCAACCATTGGAAGATGAAGAAGATAAATTCAACAGAGCAAAGAATTGG

AGGGTTTTGTCCACTAACTCCTAAAGAGGTTGGCATATTTCTTCAAGGTCTTGGTTATACTTCATCGACA

CTAATATACATTGCGGCTGGGGAGATTTATGGTGGAAGCACTCACCTTAAAGAGCTTACATCTCGCTTTC

CGAATGTTGTTTTCAAGGAAACAATTGCTACTGAGGAAGAGCTGAAAGAATTAACCAATCATGCATCTCA

AACTGCAGCTCTTGATTATATAATATCAGTAGAGAGTGACGTATTCATTCCATCGCATTCAGGTAACATG

GCAAGAACTGTTGAGGGGCATCGCAGGTTTCTAGGCCACCGAACAACAATTACCCCTGACAGGAAAGGTC

TGGTTGACATATTTGATAAAATGGATAGTGGACTGCTTAATGAAAGCTCATCGTTGTCGCGCCTTATAAT

TAAGCTGCACAAAAACAGGCGTGGTGCTCCCAGAAAAAGAGGCGGCCCTCCTCCAGGACTCAAAGGCCGA

GCAAGGTTAAGGTTTGAAGAACCCTTCTACCAAAATCCATACCCAGAATGTATATGTGATTCAAAAGTCA

CTACAAATAGTAGTCATAGTGCGTAGGTCAGCTTGCACTTATCACTAAAAAAGATGTTCCGAGACATTAG

ATAATTCAAATTAGAATGACAGTGCTTTTTACTGCTGCAAGCCTTTTTGTAATTGAACTGGTTCCTTTTT

TTGGTTCTTCGATTCTTTCTAGAATCTGTATTTTATGTTAAGATCCTGTACAATCAATACAAGTAGTTAT

CATATATCAATTATACATTTTACTTGTA

>MSTRG.716.1 gene=MSTRG.716

TACTTATCATAAAAAGCAAAAGCAAGTGCATCAAAACTCTTCTCACAACCACAATTCTAAACTGCATATG

CATAAAAATGTCACCTCTGCACCAAACACTCAAGCTTCTAGGCACACACACACTCCAAATGCTAAACCTA

CTGTGAATGCTCCTAAAAGTGTGTTAGTTGATCCATCCAATCTGTCTAAGCTTGATAACATGCTCTTGAA

TGTGCAACCTGGTATGATGATCTATGTTTCTCAGAATGGTGTGGTGCATAACATGTTTCTATCTGCTAAG

TGGGAAAACATG

>MSTRG.719.1 gene=MSTRG.719

TGTGTTGTTCTGGTATTGTTAATTGTTATGGATGCAGGAGCTTCTTGCATAACAAGATCGTAACATCTAT

GGTGGTGGTGGAGAGATTCAGAAGGGGCGGAGATGAAGGATGCTAATTTGGCGATAATTGTGCCTCAAGT

AAGGATATTAAATATTCTTAATTAAGGGTTTTTAGTACCATTTATGGCGACCATAATTGGGGCATTTATG

GCCCATAGAGGCATTTAAGTGCCATAATGTTGGCTCATTTATGTCTTTATTTTAATTTTTTTCAAATTTA

CTCATAATTCTTTTATTCGATTTTGTCTTATTTTCAGGATGTTTAAAAAAGATTTTATGAATTTACTTAT

GAAGGCTGCTTCTGAGAAGACGCGAACCTTGGTGTACAAGACATTTGAGCTACAAACATTGATAGCAGGG

AATAAAGAAGACATTTTTAGTCTGCTTTGTAGATGATGTAATTTTGTAGGGCACTCTTCTTTCCCCTTGA

GTTTTTTTCCACAGGGTTTTACTCTTGAAGGCTTTTAACGAGGTCTTTTTCGGTGGGTATCTCTTCTGAC

TTGAAAGGCTATGTTA

>MSTRG.720.1 gene=MSTRG.720

GGGATGAATATGCCGATGAATATGCCTTTCTAAAAAAAAAGAGAGAAAAAAAAAAGTGAAAAAAAAAGAA

TAAAAATTATCAGGTACTCCTTTGAGATCAATGGGTAAAATGCAATGTCATGTGAAAGGGACGATCCATG

TACTTGTGCTGGTATTAAGTGCAAATGTATTAGAATAAGCATATTCTTGGTGACTTTTCACAACCCTTGA

TTACTGGAGAATTACTTGATTAAAAAAAAAAAAAAAAAAAGCGAAGTTGAATGGCGGTCGTGACTCACTT

ACACTGAGAAGCGTCCCAACCTTAGACTTAGCAAGAAAAAAAAAAAAAAAAAAAAAAAAATAGGAGAGGC

ATAGGAATTCTTTAGTGACCCTAAATTGTAGTTGACTCTTTAGTAAAGAAGTGTTTAAACCTGATTCTGA

TTAACGGCTCATAGTGAGGACTCTGTTGAAGTTTGATAGTCTTTGTTTTGTTTCACTAGAGAGCATGCTA

GCACACACGATGCACTTCACACTTGTAGTGGAAGAGAACATGGCTAATATGTGAAAGAGATGAATGCCGA

GAATGTTGCATATTCTGAGGATTCTATGTTAACAAGGATTACACTTCATGATTCGAGTAGACGTAGGCAT

ATAGTTGCATTCATGCATTGCATTAGTAGTTTGTGTGTGTGTCTATGCTTGAGGACAAGCATCGGTTTAA

GTTTGGGGGTGTGATAAGTGGATTTTATATCCACTTGGAATGCCTTCGTTTCGACTTAAATTGATGATTT

GGCCTCAAGTATGTGGTGTTTTTGATGTGTTCTTGTGGTGTTGTGTAGGTTTCCTAGGAGGAGGATAAAG

GGTGGATTAAAGGCTAAAAGGTGAAAGAAACGGCGTAAGGATCGAGCGAGCAAGGAAGGAGAAAAGGGAA

GGAAGGTTCCAAGAGCGTTCGGGGCTGCCCAAATGGGGTCTCCACCCCATTTACGGGGTCTCCACCCCGT

TCTGAAGAAGAAAAATCTGCCAAGATGGGGTCTCCACCCCACTGATGGGGTCTCCACCCCACTTCAGCAG

AATAGAGGCTGCCAAGACAGGGTCTCCACCCCACTGACGGGGTCTCCACCCCACTGACGGGGTCTCCACC

CCATTCTGTTGGGAGAAAAAGCCCGAATTGCCTATTCTTGATCCGTTTGAAGGCCCGATCGCTGTGGGAC

TTAAATCAAAGCTCAAGGGAGAAGCCTAATAACCTAGAAACATTCCAAGGAGCACGTGACGGCTACG

>MSTRG.721.1 gene=MSTRG.721

ATAAAAGTTAGCATTATCAGATTTTTCATAAATATTTTTAATTTTTTATACAAACGGATCAAAAAATATA

GAAGCCGCCTTAACATAAAAGTCGGCTTCTTAGCATAAAAACACAAAACACAGAAGTCTTGTTACCCAAA

TAGGCACCGAAATTTTAAGCATCCTTTCTTAAGTGACCAAGCTCATAGAGTTGCTTAAATTTGTGGTAAA

CAAAGTGTGTATCCATGAAACCTGTGTTGTGTGATCATCAGCTACTTGTGGCGGTGTTTCTCGGTAATAT

TAGCAGCCTGGCAGTTCCTACTTACTCTAGGACAACATCGAGATCAAAGGGACGGAAAATGAAGAGGAAA

AAGAAAAAAGAGTACTAGAGGGGTGCTCAAGAACAAGTAACAAATGCTTTGTTCTTGTAACACCTTTGGC

TTCTAGACGCAACCAGTTTGTATGGCACCAATATCGATCTCCATGTCCTTCTTAATCTTGCATCTTGTTC

TGTTTTCCTTTCCACTTTCCTAGTTGCTTGCTTTCATCTTTGTGTTGCTTTCCTTGGCTTTCATGTTCTT

TTGTTTGGTTTTCATGACTCTGTTATTGCAAGAAACCGGAGTATATAAATAAATTGTGCCAGTTTCTGTT

TTTCGA

>MSTRG.725.1 gene=MSTRG.725

TCCATTGCTGCTCTCAGACAAGGGCGATTCAGAGTTCAGACTCTCGCATCGACTCCACAGATTTCAATAA

GCTCCATGTCCGACGCATCTCAAGGATTCAGTTTACCATTGCAACGATTTTGAAATCTAAAATTATCTAA

GCATACAGAATTGAAATGGCTGGTGATATGGAATCAAATAATTCATCGGTACTAGAAAGAGGCGCTGGAA

AAAATAAAAGGAAATGGACAGAAGAAGAAGATGAAAAACTAGTCGAAGCTTTGATGGAACTTGTGAATAC

CGGAAAGTTCAAAGCGGACAATGGCTTTAAACCTGGATACCTGACATTTCTTGAGAGTTCACTGCAAACC

AAGCTTCCTACAGCACGTATAAAGGGAAAGCCTCATATAGAGTCTCGAATGAAGACACTAAAAAAGGATT

TCAGTGCTGTGTACGACATCAGCTATGGAGCAAACAGTTCGGGGTTTGGATGGAACGCAGAAGACAATGT

TGTTACGGCGCCGAGGGATGTTTGGGTTCAATATCTTAAGGTTCATCCTGGAGCAGCAAAATGGCAGAAT

ACTGCCATGCCATCATTCAAAGAACTTTCAGTTATTTTTGGAAAAGATAGAGCAACCGGAAATATGGCTG

AAAATCTTGAAGATGTGGTAGAAGAATTGAACACTGAAGCTGCTGATGAATTATCTCTTCAGGAGGACCT

GCAACGAAGCACACATTCAGACGAGTCAACAAGCAAGAAAAGGAAGAAAGGAAATGTAGAATCATTGTTG

GAAGCTGTGTATGCTGCTTCGGACAGGATTGCCAATCAGTTTGAGGCGTCAACCAAGTTACTTATTGCAG

CAGAAGAAGATATGATGCAGAAAAAGAAGCAATTAAATGACGAACTATCAAAAATTCCAAATCTCACGGT

ATTGCAGAAGCTTCAAGTTGCAAAGAAAATAGCCAAGGACGAAGATCTTATGATATTGTTTTTTGCAGCA

CCGGCCGAGGAAAAAATCATCTTTGTTCATGCTGTTCTAGATAACCAGATTTGAGCAAACTTTGTTATTA

AGAACCTTTGTTTTTGTTAGGTCCAATCAGACGTAGAAGGGGGGGGTTGAATACGTCGTAC

>MSTRG.725.2 gene=MSTRG.725

ACCAGTTTCTTTTCACGAATACCGAGAGAAACCGAAGTGTGTTATTCGTGTTCTTCGTAATCAAACGAGG

ATTTGGAGGTGTTATTGGAATCCGATGGAGGTGTATGAATAGTCAAAATGAAGCTACCGACGCGTAGAAT

CTAGTTTAATCATCCGTTTGTGTGCAGATTCAACAGAATTGAAATGGCTGGTGATATGGAATCAAATAAT

TCATCGGTACTAGAAAGAGGCGCTGGAAAAAATAAAAGGAAATGGACAGAAGAAGAAGATGAAAAACTAG

TCGAAGCTTTGATGGAACTTGTGAATACCGGAAAGTTCAAAGCGGACAATGGCTTTAAACCTGGATACCT

GACATTTCTTGAGAGTTCACTGCAAACCAAGCTTCCTACAGCACGTATAAAGGGAAAGCCTCATATAGAG

TCTCGAATGAAGACACTAAAAAAGGATTTCAGTGCTGTGTACGACATCAGCTATGGAGCAAACAGTTCGG

GGTTTGGATGGAACGCAGAAGACAATGTTGTTACGGCGCCGAGGGATGTTTGGGTTCAATATCTTAAGGT

TCATCCTGGAGCAGCAAAATGGCAGAATACTGCCATGCCATCATTCAAAGAACTTTCAGTTATTTTTGGA

AAAGATAGAGCAACCGGAAATATGGCTGAAAATCTTGAAGATGTGGTAGAAGAATTGAACACTGAAGCTG

CTGATGAATTATCTCTTCAGGAGGACCTGCAACGAAGCACACATTCAGACGAGTCAACAAGCAAGAAAAG

GAAGAAAGGAAATGTAGAATCATTGTTGGAAGCTGTGTATGCTGCTTCGGACAGGATTGCCAATCAGTTT

GAGGCGTCAACCAAGTTACTTATTGCAGCAGAAGAAGATATGATGCAGAAAAAGAAGCAATTAAATGACG

AACTATCAAAAATTCCAAATCTCACGGTATTGCAGAAGCTTCAAGTTGCAAAGAAAATAGCCAAGGACGA

AGATCTTATGATATTGTTTTTTGCAGCACCGGCCGAGGAAAAAATCATCTTTGTTCATGCTGTTCTAGAT

AACCAGATTTGAGCAAACTTTGTTATTAAGAACCTTTGTTTTTGTTAGGTCCAATCAGACGTAGAAGGGG

GGGGTTGAATACGTCGTAC

>MSTRG.725.3 gene=MSTRG.725

CTTGTTCTGAGACTATTTCTGTTCTTGTATATGCAGAATTGAAATGGCTGGTGATATGGAATCAAATAAT

TCATCGGTACTAGAAAGAGGCGCTGGAAAAAATAAAAGGAAATGGACAGAAGAAGAAGATGAAAAACTAG

TCGAAGCTTTGATGGAACTTGTGAATACCGGAAAGTTCAAAGCGGACAATGGCTTTAAACCTGGATACCT

GACATTTCTTGAGAGTTCACTGCAAACCAAGCTTCCTACAGCACGTATAAAGGGAAAGCCTCATATAGAG

TCTCGAATGAAGACACTAAAAAAGGATTTCAGTGCTGTGTACGACATCAGCTATGGAGCAAACAGTTCGG

GGTTTGGATGGAACGCAGAAGACAATGTTGTTACGGCGCCGAGGGATGTTTGGGTTCAATATCTTAAGGT

AATTAATTATCACATATAATTAATAATACCAGATCTGCTTTTCCTGGCAATTTCTTACACTAACATGGTT

ATCACACTCCATGACTCCACCAAGTCTCCTTCCACCAAGACATTGCAGAATAAAAAAAACAAAAACATGT

ACTTAGCAGTTGCAATGTGCAACTAAATTCCACAATTATGAAAGGATTATATTATTAGGGAAGCCATAGT

AGGTTGTTTGGTTGAGGGAATTGAGTTTTTATTATTAAAAAGTGCTGGATGTAGTGGA

>MSTRG.725.4 gene=MSTRG.725

ACCAGTTTCTTTTCACGAATACCGAGAGAAACCGAAGTGTGTTATTCGTGTTCTTCGTAATCAAACGAGG

ATTTGGAGGTGTTATTGGAATCCGATGGAGGTGTATGAATAGTCAAAATGAAGCTACCGACGCGTAGAAT

CTAGTTTAATCATCCGTTTGTGTGCAGATTCAACAGGATTTGGACGAGACGTGTACGCTCGAAGACGTCA

GGAAGTCGAAATGGTATTGCAAAGCGAATAAGGGATGAATCGAGTAAGCGAAGCGAAGATGTACGGTAGA

TAGAGTATAGCCAGAGATGGTCTAGAGACTATGAGATGCATAGATTGTGAAAGTGGAAAGATGAGATTGA

GATGTGAGACTGGAGTCAGATTTAAGGCAAGCTTAAAGATGAGATGATTGATTGAGATTGTGAATAAGGC

TAATGCTAGGCGAGGAGACAGAGTTGAGAATCCAGTGGTCGAGGAGTGTTCAGGAAAGTTGATGAGGTTG

GTGCAAGCTGAAATAGTTGACAGTGATTCTTCTTATCGAAGATGATCTGATTTGGTAAGAGATGTTGTGT

AATTAATAGTGGTAGATTCTAATTTCAATACTGTAACCTGGATGCGATCCTGTTGCTTTTAGGGGGTTTA

AATGTTCTCTTTCG

>MSTRG.725.5 gene=MSTRG.725

ACCAGTTTCTTTTCACGAATACCGAGAGAAACCGAAGTGTGTTATTCGTGTTCTTCGTAATCAAACGAGG

ATTTGGAGGTGTTATTGGAATCCGATGGAGGTGTATGAATAGTCAAAATGAAGCTACCGACGCGTAGAAT

CTAGTTTAATCATCCGTTTGTGTGCAGATTCAACAGAAAGGCTTAAAGATGAGATGATTGATTGAGATTG

TGAATAAGGCTAATGCTAGGCGAGGAGACAGAGTTGAGAATCCAGTGGTCGAGGAGTGTTCAGGAAAGTT

GATGAGGTTGGTGCAAGCTGAAATAGTTGACAGTGATTCTTCTTATCGAAGATGATCTGATTTGGTAAGA

GATGTTGTGTAATTAATAGTGGTAGATTCTAATTTCAATACTGTAACCTGGATGCGATCCTGTTGCTTTT

AGGGGG

>MSTRG.725.6 gene=MSTRG.725

CGCGTAGAATCTAGTTTAATCATCCGTTTGTGTGCAGATTCAACAGGATTTGGACGAGACGTGTACGCTC

GAAGACGTCAGGAAGTCGAAATGGTATTGCAAAGCGAATAAGGGATGAATCGAGTAAGCGAAGCGAAGAT

GTACGGTAGATAGAGTATAGCCAGAGATGGTCTAGAGACTATGAGATGCATAGATTGTGAAAGTGGAAAG

ATGAGATTGAGATGTGAGACTGGAGTCAGATTTAAGAAAGGCTTAAAGATGAGATGATTGATTGAGATTG

TGAATAAGGCTAATGCTAGGCGAGGAGACAGAGTTGAGAATCCAGTGGTCGAGGAGTGTTCAGGAAAGTT

GATG

>MSTRG.728.1 gene=MSTRG.728

AGCAGATAATGTAACAATTGGAAGGAACAATAACTAGAAGTGTTGTGTGATTTCTGGAGCCCATGATTAA

AAGGAAATTTTATTGACGTGAATGATTAGTCCACTTAAGTGGTATTGCTGTTCAAATTCAGAAAGTGCAG

AATCTGTGTTCCCAACTTACGACTATACTTCAATGGCATTACTGGAAGGAGTCACACTATGGGATGATTA

TCACAAATTTATATTTCAAGTCGGCAGAAATCTGCTTCCTATGCAATGTTAGGATATTT

>MSTRG.737.1 gene=MSTRG.737

TACAACCACAGTTCCCAACCTAATCTCTATTCAAAATTTTTAATCAATCAAAACAAATTCACATTGCCGC

TTCTCCAATTACTTGCGCGCAGATGGAAAGAAAATTCGAGCTGGGTAAAGATGCGGTTGATCTAGGTATT

CAACCACCATTCAGTCGCAATGTTTGTAAGTTGCTCAGATTTTGATACACTCTTCTTGGTTGATTGATTC

ACAACTTTTTACATATACCATTCAGTCGAAATGTTTACAGATTTTGTTACCCTCTTTCCTCTTTTCCGAA

TTGATGGAGAACACACTTTGATAGTAAGTTTGTTTTTGTTGCAGTTTATAGTTTGATGTGAACTGTTTGA
[truncated: 12,254,190 more chars]
