# Supplementary material for: Insights into long non-coding RNA regulation of anthocyanin carrot root pigmentation
Source: Sci Rep. 2021 Feb 18;11:4093. doi: 10.1038/s41598-021-83514-4 (PMC7892999; doi:10.1038/s41598-021-83514-4)
Supplement: Supplementary file 2 — Supplementary Information 2. [file 41598_2021_83514_MOESM2_ESM.pdf]

Supplementary information

Insights into long non-coding RNA regulation of anthocyanin carrot root pigmentation

Constanza Chialva, Thomas Blein, Martin Crespi & Diego Lijavetzky

Supplementary Figure S1

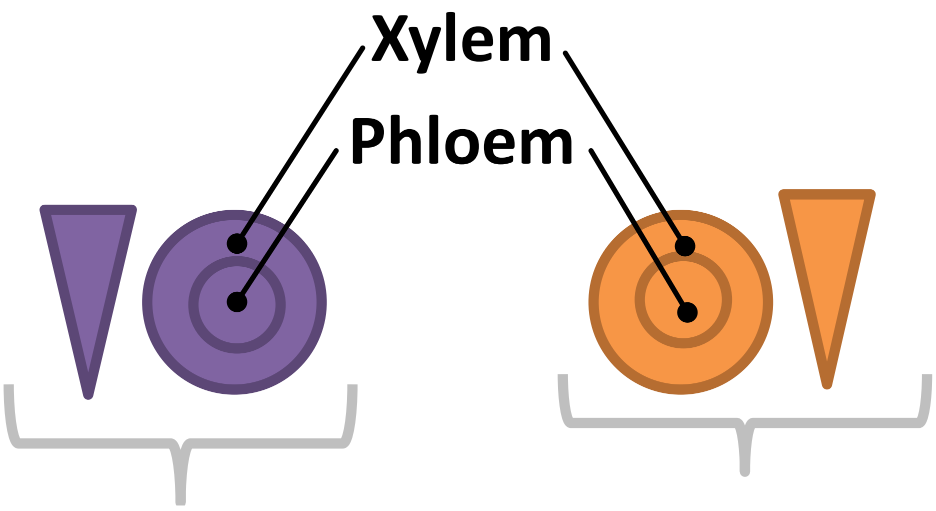

D. carota
cv. Nightbird
(purple)

D. carota
cv. Musica
(orange)

Supplementary Figure S2

a)

geneID	product
DCAR_010745	DcMyb7
DCAR_000385	DcMyb6
DCAR_002739	DcbHLH3
MSTRG.8365	DcSAT1
DCAR_021269	DcUCGXT1
DCAR_001218	Myb_DNA-bind_3 domain-containing protein
DCAR_012866	transcription factor bHLH145
DCAR_019332	transcription factor bHLH93-like
DCAR_005631	transcription factor MYC2-like

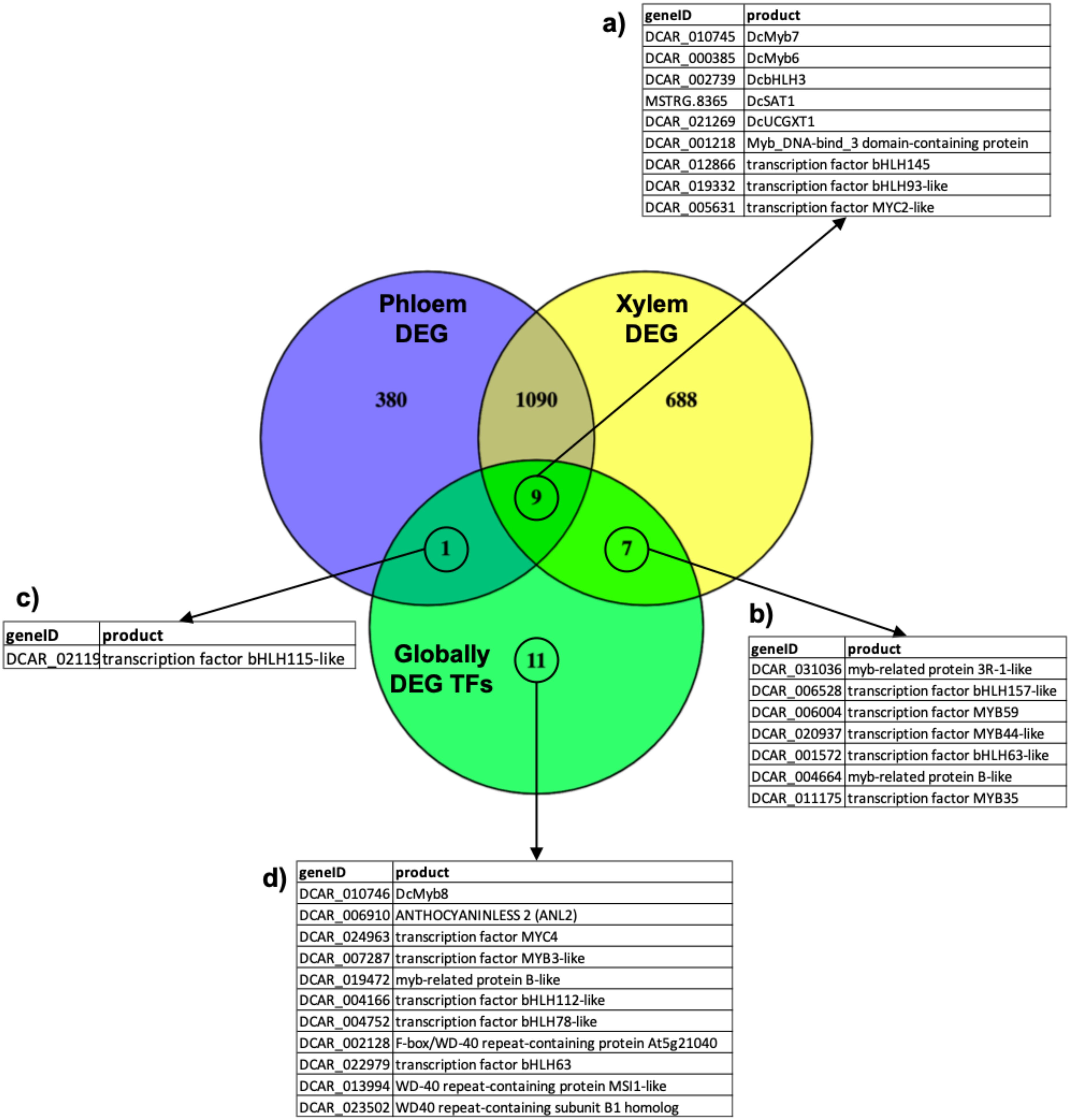

c)

geneID	product
DCAR_02119	transcription factor bHLH115-like

b)

geneID	product
DCAR_031036	myb-related protein 3R-1-like
DCAR_006528	transcription factor bHLH157-like
DCAR_006004	transcription factor MYB59
DCAR_020937	transcription factor MYB44-like
DCAR_001572	transcription factor bHLH63-like
DCAR_004664	myb-related protein B-like
DCAR_011175	transcription factor MYB35

d)

geneID	product
DCAR_010746	DcMyb8
DCAR_006910	ANTHOCYANINLESS 2 (ANL2)
DCAR_024963	transcription factor MYC4
DCAR_007287	transcription factor MYB3-like
DCAR_019472	myb-related protein B-like
DCAR_004166	transcription factor bHLH112-like
DCAR_004752	transcription factor bHLH78-like
DCAR_002128	F-box/WD-40 repeat-containing protein At5g21040
DCAR_022979	transcription factor bHLH63
DCAR_013994	WD-40 repeat-containing protein MSI1-like
DCAR_023502	WD40 repeat-containing subunit B1 homolog

Supplementary Figure S3

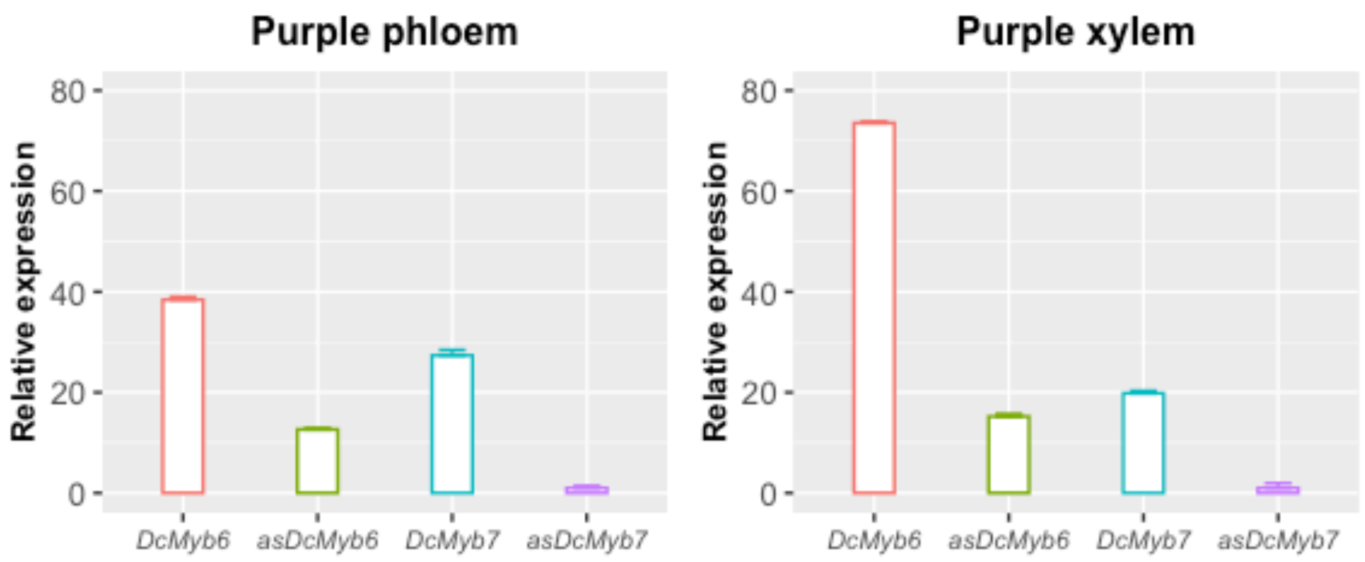

Supplementary Table S1

Library ID	Sample ID	Total read bases (bp)	Total read bases (Gbp)	Total reads	GC(%)	AT(%)	Q20(%)	Q30(%)	Uniquely mapped reads (%)
MORADA_M1F1	Pur_F1	5699079126	5.7	56426526	44.83	55.17	98.1	94.2	90.9
MORADA_M3F2	Pur_F2	5263944462	5.26	52118262	44.81	55.19	98.1	94.1	91.5
MORADA_M3F3	Pur_F3	4935182998	4.94	48863198	45.26	54.74	97.9	93.9	87.8
MORADA_M1X1	Pur_X1	4390776232	4.39	43473032	44.78	55.22	98.1	94.2	92
MORADA_M3X2	Pur_X2	5964969302	5.96	59059102	44.75	55.25	98.1	94.1	91.7
MORADA_M3X3	Pur_X3	4845934146	4.85	47979546	44.91	55.09	98	93.9	86.5
NARANJA_M3F1	Org_F1	4704372748	4.7	46577948	44.67	55.33	98	93.9	91.9
NARANJA_M3F2	Org_F2	4703444760	4.7	46568760	44.79	55.21	97.9	93.7	94.1
NARANJA_M3F4	Org_F4	4779354946	4.78	47320346	44.47	55.53	98	94.1	92.8
NARANJA_M3X1	Org_X1	6093490994	6.09	60331594	45.11	54.89	98	94	92.6
NARANJA_M3X2	Org_X2	5819580004	5.82	57619604	44.67	55.33	98	94.2	86.7
NARANJA_M3X4	Org_X4	5069705100	5.07	50195100	44.28	55.72	98.1	94.3	92.8
		**Average**	5.19	51377752	44.78	55.22	98	94.1	90.9
		**STD**	0.569		0.258	0.258	0.08	0.18	2.53

Supplementary Table S3

Transcript type	Known	New	Total
coding	32109	2095	34204
noncoding	915	6373	7288
NAT	0	1521	1521
lincRNA	915	4852	5767
structural	1239	16	1255
total	34263	8484	42747

Supplementary Table S6

		Correlation			
		Pearson *r*		Spearman's Rho	
coding	lncNAT	*r*	*p*-value	r_s	*p*-value
DcMyb7	***asDcMyb7***	0.81	< 0.01	0.89	<0.001
DcMyb6	***asDcMyb6***	0.86	<0.001	0.79	<0.01
**DCAR_007914**	**MSTRG.6643**	-0.75	<0.01	-0.83	<0.001
**DCAR_010712**	**MSTRG.9085**	0.87	<0.001	0.85	<0.001
**DCAR_014717**	**MSTRG.11960**	0.91	<0.0001	0.89	<0.001
**DCAR_015415**	**MSTRG.11340**	-0.89	<0.0001	-0.84	<0.001
**DCAR_015459**	**MSTRG.11308**	0.83	<0.001	0.85	<0.001
**DCAR_021597**	**MSTRG.18539**	0.71	<0.01	0.74	<0.01
**DCAR_022107**	**MSTRG.18052**	0.84	<0.001	0.91	<0.0001
**DCAR_022871**	**MSTRG.17454**	0.91	<0.0001	0.90	<0.0001
**DCAR_023986**	**MSTRG.20882**	0.71	<0.01	0.81	<0.01
**DCAR_029820**	**MSTRG.26086**	0.75	<0.01	<0.75	<0.01
**DCAR_031718**	**MSTRG.27809**	-0.88	<0.001	-0.88	<0.001
**DCAR_032195**	**MSTRG.28149**	0.75	<0.01	0.80	<0.01
**DCAR_009536**	**MSTRG.8070**	-0.82	<0.01	-0.80	<0.01
**DCAR_006881**	**MSTRG.5651**	0.96	<0 .00001	0.95	<0 .00001
**DCAR_007044**	**MSTRG.5819**	0.98	< 0.00001	0.90	<0.001
**DCAR_004853**	**MSTRG.3846**	0.95	<0.00001	0.92	<0.0001
**DCAR_006773**	**MSTRG.5544**	0.81	<0.01	0.85	<0.001

Supplementary Table S7

Name	Sense	Sequence (5'->3')	Length	Tm	Product length
**DcActin-7**	**Forward**	GCTACTTGGATGTGGCTGT	19	52.63	93
	**Reverse**	CCGACTGAAGAGCTGATCC	19	57.8	
**asDcMyb7-1**	**Forward**	GTGTTGCTGGCAAAGGAGAGT	21	61.09	145
	**Reverse**	AAACAAACAAGCAGTGCGACTA	22	59.32	
**DcMyb7-1**	**Forward**	TGTTGTTAATGTCGTTGCCG	20	57.33	128
	**Reverse**	CCTTCCCCGGACCTTATCAAA	21	59.44	
**asDcMyb6-2**	**Forward**	TGGTCGAAGATAGTTGAGCCAC	22	60.09	146
	**Reverse**	GCAGGTACACAATTCTTCCCACAA	24	61.53	
**DcMyb6-1**	**Forward**	CACCACTTCGGTCTGTCCAT	20	55.00	265
	**Reverse**	CCCTCGGAGAGCAGGGTTG	19	62.02	
