# Supplementary material for: Insights into long non-coding RNA regulation of anthocyanin carrot root pigmentation
Source: Sci Rep. 2021 Feb 18;11:4093. doi: 10.1038/s41598-021-83514-4 (PMC7892999; doi:10.1038/s41598-021-83514-4)
Supplement: Supplementary file 6 — Supplementary Legends. [file 41598_2021_83514_MOESM6_ESM.docx]

**Supplementary Figure S1**. Scheme of the performed dissection between xylem and phloem tissues.

**Supplementary Figure S2**. Tissue specific differential expression of the 26 ‘MBW’ TFs identified in the experiment. a) Genes differentially expressed between purple and orange carrots both in xylem and phloem tissues; b) genes differentially expressed between purple and orange carrots just in xylem; c) gene differentially expressed between purple and orange carrots just in phloem; d) genes differentially expressed between purple and orange carrots detected after the join analysis of phloem and xylem samples.

**Supplementary Figure S3**. Comparative RT-qPCR expression of *DcMyb6*, *DcMyb7* and their corresponding lncNATs in purple phloem and xylem. Data are means ± SD of three biological replicates. Carrot *actin-7* was used as reference gene and *asDcMyb7* as reference sample.

**Supplementary Table S1**. Summary of NGS and quality control data regarding the 12 sequenced libraries.

**Supplementary Table S2**. Genome annotation of the newly identified transcripts.

**Supplementary Table S3**. Known and newly annotated carrot genes classified as coding, noncoding and structural transcripts.

**Supplementary Table S4**. Normalized counts for each of the 12 sequenced libraries.

**Supplementary Table S5**. Overall differentially expressed genes (DEGs) list, including statistical tests, *cis*-located sequences, gene lengths and gene products. The 21 identified lncNAT/coding transcript pairs are sorted on top of the list.

**Supplementary Table S6**. Pearson and Spearman correlation coefficients between the expression levels of the 19 identified lncNAT/coding transcript pairs across the 12 analyzed libraries.

**Supplementary Table S7**. Primers used for RT-qPCR.

**Supplementary File S1.** FASTA sequences of the newly annotated transcripts.
